# 16th European Headache Congress 2022 meeting abstracts: Vienna, Austria. 7-10 December 2022

**DOI:** 10.1186/s10194-022-01527-4

**Published:** 2022-12-07

## A1 Development and pharmacological characterisation of bifunctional CGRP-PACAP receptor antagonists in transfected cells and spinal cord cultures

### D. Hay^1^, Z. Tasma^2^, A. Siow^2^, M. Brimble^2^, P. Harris^2^, C. Walker^2^

#### ^1^University of Otago, Dunedin, New Zealand; ^2^University of Auckland, Auckland, New Zealand

##### **Correspondence:** D. Hay

**Question:** The neuropeptides calcitonin gene-related peptide (CGRP) and pituitary adenylate cyclase-activating peptide (PACAP) are both implicated in migraine. Blocking the activity of these peptides simultaneously may provide a clinical advantage over individual blockade. One strategy is to develop a bifunctional ligand, capable of antagonizing both systems at once. As a starting point we utilized the known antagonism imparted by CGRP and PACAP peptide fragments, exploring different lengths of PACAP. From this, we selected CGRP_8-37_ and PACAP_6-38_ to attach together and assessed these molecules as bifunctional antagonists.

**Methods:** Peptides were synthesized in-house and CGRP_8-37_ was linked to PACAP_6-38_ using 1,3-dipolar cycloaddition at amino acid positions 21, 34 and 38. The potency of these peptides as bifunctional antagonists was then tested, and compared to the parent fragments. We tested antagonism against CGRP at the human CGRP and AMY_1_ receptors and against PACAP-27, PACAP-38 and VIP at the human PAC_1_, VPAC_1_ and VPAC_2_ receptors in Cos7 cells (cAMP production). Translational relevance was assessed by measuring antagonism of agonist-stimulated cAMP production in primary rat spinal cord cultures.

**Results:** The bifunctional antagonists generally displayed similar antagonist activity to CGRP_8-37_ and PACAP_6-38_ in receptor transfected Cos7 cells and spinal cord cultures. Interestingly, linking CGRP_8-37_ to position 38 of PACAP_6-38_ generated a peptide with greater antagonist potency than CGRP_8-37_ at CGRP and AMY_1_ receptors in Cos7 cells.

**Conclusions:** This study provides proof-of-concept that bifunctional antagonists capable of blocking both CGRP and PACAP activity can be generated.

## A2 Crosstalk between cannabinoid and vanilloid systems: role of CB receptors in the capsaicin-induced relaxation responses in human coronary arteries

### E. Rivera- Mancilla^1^, A. van den Bogaerdt^2^, A. H. J. Danser^1^, C. M. Villalón^3^, A. Maassen van den Brink^1^

#### ^1^Erasmus Medical Center, Division of Vascular Medicine and Pharmacology, Department of Internal Medicine, Rotterdam, Netherlands; ^2^ETB-BISLIFE, Heart Valve Department, Beverwijk, Netherlands; ^3^Cinvestav-Coapa, Pharmacobiology, Mexico City, Mexico

##### **Correspondence:** E. Rivera- Mancilla

**Background:** The use of cannabis and its derivatives has increased during the last years due to their therapeutic potential. However, the exact mechanisms of action of cannabinoids are still limited. It has been suggested that cannabinoids can exert their effects via the activation of cannabinoid receptors (*i.e.* CB_1_ or CB_2_ receptors) and/or transient receptor potential vanilloid 1 (TRPV1) channels, suggesting an interaction between both systems. We investigated the role of CB receptors in the vasodilatory effects induced by capsaicin in human isolated coronary arteries (HCAs).

**Methods:** In HCAs (female, n=5; 56±5 years and male, n=4; 57±4 years), the vasodilatory responses to capsaicin (TRPV1 channel agonist) were evaluated in the absence or presence of the antagonists capsazepine (TRPV1, 5 μM); AM6545 (CB_1_ receptor, 1 μM); AM630 (CB_2_ receptor, 1 μM); O-1918 (putative endothelial CB receptor, 10 μM) or cannabidiol (GPR55 receptor, 1 μM) to obtain the maximum contractile response (E_max_).

**Results:** Capsaicin induced concentration-dependent relaxation responses (E_max_ 109±8%), which were significantly reduced by AM6545 (E_max_ 87±4%) or cannabidiol (E_max_ 86±3%), but not by capsazepine (E_max_ 103±6%), AM630 (E_max_ 100±3%) or O-1918 (E_max_ 93±5%). Moreover, pilot experiments (n=2) showed that the maximal response induced by N-arachidonoylethanolamine, (ACEA, a CB_1_ receptor agonist; E_max_ 43±7%) is inhibited by AM6545 or capsazepine: E_max_ 16±3% and 21±4%, respectively.

**Conclusions:** (i) Capsaicin-induced relaxation responses are mediated by CB_1_ and GRP55 receptors; and (ii) TRPV1 channels may be involved in the modulation of the ACEA-induced relaxation responses. Thus, we can suggest that the CB and vanilloid systems may share a signaling pathway to modulate the vascular tone, which may provide novel therapeutic targets for vascular disorders (*e.g.* migraine and its related cardiovascular events). Further studies should elucidate additional mechanisms involved in these responses.

## A3 Second messenger signaling bypasses blockade of the calcitonin gene-related peptide (CGRP) receptor to provoke migraine attacks in humans

### T. P. Do^1^, C. Deligianni^1^, S. Amirguliyev^1^, J. Snellman^2^, C. L. Lopez^3^, M. A. Al-Karagholi^1^, S. Guo^1^, M. Ashina^1^

#### ^1^Danish Headache Center, Glostrup, Denmark; ^2^Novartis, Basel, Switzerland; ^3^Roche Innovation Center Basel, Basel, Switzerland

##### **Correspondence:** T. P. Do

Numerous endogenous molecules trigger migraine attacks when administered to humans. Of therapeutic importance, this has led to the concept of a "migraine attack signaling cascade" with the calcitonin gene-related peptide (CGRP) acting via a downstream second messenger cyclic adenosine monophosphate (cAMP) in intracranial vascular smooth muscle cells and other cells. However, whether intracellular cAMP signaling acts strictly downstream or is dependent on CGRP receptor activation during a migraine attack has never been tested directly in humans. Here, using a human provocation model (CGRP and phosphodiesterase 3 inhibitor, cilostazol, an agent known to accumulate intracellular cAMP by inhibiting its degradation) in a randomized, double-blind, placebo-controlled, parallel trial design, we demonstrate that migraine attacks can be provoked by intracellular cAMP-mediated mechanisms using cilostazol in the presence of CGRP receptor blockade (erenumab). Consistent with these findings, cilostazol-induced dilation of cranial arteries was unaffected by a CGRP receptor blockade. Our work provides clinical evidence that cAMP-evoked migraine attacks act downstream of the CGRP receptor, and that these cAMP-evoked migraine attacks appear independent of CGRP-receptor activation. These findings open new avenues for mechanism-based drug development for migraine.

## A4 Comparison of the primary headache, COVID-19 headache, and COVID-19 immunization headache according to the phenotype in healthcare workers

### A. Gonzalez-Martinez^1^, P. Paños Basterra^1^, M. Domínguez Gallego^1^, A. Somovilla^1^, C. Martín Ramos^1^, Á. Morales Caballero^2^, A. López Guerrero^2^, M. García Cebrián^2^, J. Vivancos^1^, A. B. Gago-Veiga^1^

#### ^1^Hospital Universitario de La Princesa & Instituto de Investigación Sanitaria Princesa (IIS-Princesa), Neurology, Madrid, Spain; ^2^Hospital Universitario de La Princesa, Occupational Medicine Department, Madrid, Spain

##### **Correspondence:** A. Gonzalez-Martinez

**Objective:** Headache is a frequent symptom during SARS-CoV-2 infection and following COVID-19 immunization. We aimed to characterize the semiology of COVID-19 headache and COVID-19 immunization headache, as well as to evaluate the influence of primary headache-migraine or tension-type headache (TTH)-on the COVID-19 headache and COVID-19 immunization headache phenotype.

**Methods:** We performed an observational study through an online survey in healthcare workers who had SARS-CoV-2 infection and were included in the Occupational Medicine´s register of our tertiary hospital. Clinical, demographic and headache variables were collected during infection and immunization.

**Results:** We included 109 participants with COVID-19 headache, 94/109(86,2%) women, 45.1(SD:12.45 years, 24.1(SD:4.3) BMI, 29/109(26.6%) cardiovascular risk factors, 15/112(13.39%) 15/109(13.8%) anxiety, 7/109(6.4%) depression, 22/109(20.18%) migraine as primary headache, 11/109(10,09%) TTH as primary headache. COVID-19 headache was the first symptom in 24/109(22%), appeared in

**Conclusions:** According to our study, both COVID-19 headache and COVID-19 immunization headache frequently follow a TTH-like phenotype more than a migraine phenotype. Moreover, COVID-19 immunization headache is frequently more similar to the COVID-19 headache than to the primary headache.

## A5 Cluster headache genome-wide association study identifies seven loci and implicates smoking as causal risk factor

### B. S. Winsvold^1,2,3^*,* A. Harder^4,5^, C. Ran^6^, M. A. Chalmer^7^, M. C. Dalmasso^8,9^, E. Ferkingstad^10^, A. Belin^6^, M. Matharu^11^, A. van den Maagdenberg^4,5^, T. F. Hansen^7,12^, A. Ramirez^8,13,14,15,16^, J. A. Zwart^1,2,17^, O. B. of the CCG^18^

#### ^1^Oslo University Hospital, Department of Research and Innovation, Division of Clinical Neuroscience, Oslo, Norway; ^2^Norwegian University of Science and Technology (NTNU), K. G. Jebsen Center for Genetic Epidemiology, Department of Public Health and Nursing, Faculty of Medicine and Health Sciences, Trondheim, Norway; ^3^Oslo University Hospital, Neurology, Oslo, Norway; ^4^Leiden University Medical Center, Department of Human Genetics, Leiden, Netherlands; ^5^Leiden University Medical Center, Neurology, Leiden, Netherlands; ^6^Karolinska Institutet, Department of Neuroscience, Stockholm, Sweden; ^7^University of Copenhagen, Rigshospitalet, Neurology, Glostrup, Denmark; ^8^University of Cologne, Division of Neurogenetics and Molecular Psychiatry, Department of Psychiatry and Psychotherapy, Cologne, Germany; ^9^National University A. Jauretche (UNAJ), Neurosciences and Complex Systems Unit (EnyS), CONICET, Hospital El Cruce 'N. Kirchner', Florencio Varela, Argentina; ^10^deCODE genetics / Amgen Inc., Reykjavík, Iceland; ^11^University College London, Headache and Facial Pain Group, London, United Kingdom; ^12^University of Copenhagen, Rigshospitalet, Novo Nordic Foundation Center for Protein Research, Copenhagen, Denmark; ^13^University Hospital Bonn, Department of Neurodegenerative Diseases and Geriatric Psychiatry, Bonn, Germany; ^14^German Center for Neurodegenerative Diseases (DZNE Bonn), Bonn, Germany; ^15^University of Texas Health Sciences Center, Glenn Biggs Institute for Alzheimer's & Neurodegenerative Diseases, San Antonio, TX, United States; ^16^University of Cologne, Cluster of Excellence Cellular Stress Responses in Aging-associated Diseases (CECAD), Cologne, Germany; ^17^University of Oslo, Institute of Clinical Medicine, Faculty of Medicine, Oslo, Norway; ^18^International Consortium for Cluster Headache Genetics, Oslo, Norway

##### **Correspondence:** A. Harder

**Introduction:** Cluster headache is a severe primary headache disorder preferentially affecting men. A high proportion of patients are smokers.

**Methods:** We performed a genome-wide association meta-analysis of 4,043 patients with clinically diagnosed cluster headache and 21,729 controls from ten cohorts of European ancestry.

**Results:** We confirmed the polygenic basis of cluster headache with a SNP-based heritability of 14.5%. We identified seven genome-wide significant loci, of which three are novel (*WNT*2, rs2402176, OR = 1.20; *PLCE1*, rs57866767, OR = 1.18; and *LRP1*, rs11172113, OR = 1.18) and four previously identified (*DUSP10*, rs17011182, OR = 1.38; *MERTK*, rs13399108, OR = 1.41; *FTCDNL1*, rs6714578, OR = 1.53; and *FHL5*, rs9486725, OR = 1.29). The prioritized genes showed enrichment for artery and brain tissue. Cluster headache shared only some genetic risk loci with migraine and is genetically correlated with cigarette smoking, risk-taking behavior, ADHD, depression and musculoskeletal pain. Mendelian randomization analysis indicated a causal effect of cigarette smoking intensity on cluster headache.

**Conclusion:** We identify seven risk loci, of which three are novel. We provide evidence that cluster headache and migraine have a partly distinct and a partly overlapping genetic basis. Mendelian randomization analysis indicates a causal effect of cigarette smoking on the development of cluster headache, which has potential clinical implications.

## A6 Role of the Default Mode Network in Episodic Cluster Headache: Cerebral Connectivity Analysis with Hd-Eeg

### F. Bighiani^1,2^, A. Putortì^1^, R. De Icco^1^, M. Corrado^1,2^, M. Semprini^3^, G. Sances^1^, M. Allena^1^, V. Grillo^1^, C. Tassorelli^1,2^, F. Cammarota^1^

#### ^1^IRCCS Mondino Foundation, Headache Science & Neurorehabilitation Center, Pavia, Italy; ^2^University of Pavia, Brain and Behavioral Sciences, Pavia, Italy; ^3^Istituto Italiano di Tecnologia, Rehab Technologies, Genova, Italy

##### **Correspondence:** F. Cammarota

**Introduction:** The pathophysiological mechanisms underlying episodic cluster headache (eCH), and shift between active and remission phases, are still not fully understood.

**Objectives:** We aimed to define specific internodal connectivity patterns of the default mode network (DMN) in eCH patients, through advanced brain connectivity analyses with high-density EEG (HD-EEG).

**Methods:** Twenty-four patients with eCH and 19 healthy controls (HCs) were enrolled. Patients with eCH were evaluated during both the active (T0) and the remission (T1) phases of disease. Of these 24 patients, 8 were registered only at T0, 10 only at T1, while 6 completed both registrations. The DMN areas considered for the analysis were: the right and left angular gyrus (RANG and LANG), the medial pre-frontal cortex (MPC) and the posterior cingulate cortex (PCC). Results: The study of internodal brain connectivity in patients showed lower connectivity at T1 (remission) when compared to T0 between PCC and MPC (T0=0.078±0.009 vs. T1=0.049±0.006, p=0.022) and between PCC and RANG (T0=0.076 ± 0.008 vs. T1=0.052±0.005, p=0.024). Furthermore, connectivity at T1 was lower when compared to HCs, specifically between PCC and MPC areas (CHe-T1=0.049±0.005 vs. HS=0.067±0.005, p=0.028).

**Conclusion:** eCH patients evaluated during a remission phase of disease showed lower brain connectivity between specific areas of the DMN when compared with either eCH patients tested during an active phase and HCs.

This finding may represent a biological marker of disease, while the fluctuation in PCC connectivity may reflect pathophysiological mechanisms involved in the shift from one phase of disease to the other.

## A7 Expression of vasopressin and its receptors in migraine-related regions in CNS and the trigeminal system: Influence of sex

### A. Maddahi, L. Edvinsson, K. Warfvinge

#### Lunds University, Department of Clinical Sciences, Lund, Sweden

##### **Correspondence:** L. Edvinsson

**Objective;** Hypothalamus is a key region in migraine attacks. In addition, women are disproportionately affected by migraine. The calcitonin gene-related peptide (CGRP) system is an important key player in migraine pathophysiology. CGRP signaling could be a target of hormones that influence migraine. Our aim is to identify the expression of vasopressin and its receptors in the brain and in the trigeminovascular system with focus on the migraine-related regions and, furthermore, to examine the role of sex on expression of neurohormones in the trigeminal ganglion.

**Methods;** Rat brain and trigeminal ganglia were carefully harvested and proteins and genes were analyzed by immunohistochemistry and real-time PCR, respectively. The number of vasopressin and its receptors immunoreactive neurons in male and female TG were calculated.

**Results;** Vasopressin and its receptors immunoreactivity were found in migraine-related areas within the brain and in the trigeminal ganglion, predominantly in neuronal cytoplasm. There were no differences in the number of positive cells expression of CGRP and vasopressin in the trigeminal ganglion between male and female rats. In contrast, the number of RAMP1 (CGRRP receptor) and vasopressin receptors (V1aR and V1bR) immunoreactive cells were higher in female compared to male. Vasopressin and its receptors mRNA were expressed in both hypothalamus and trigeminal ganglion; however, the vasopressin mRNA level was significantly higher in the hypothalamus.

**Conclusions;** A better understanding of potential hormonal influences on migraine mechanisms is needed to improve treatment of female migraineurs. It is intriguing that vasopressin is an output of hypothalamic neurons that influences areas associated with migraine. Therefore, vasopressin might be important hypothalamic components that contribute to migraine pathophysiology.

## A8 Switching OnabotulinumtoxinA and Monoclonal Antibodies Anti-CGRP in Severe, Drugs-Resistant Chronic Migraine

### L. F. Iannone^1^, A. Chiarugi^1^, D. Fattori^1^, F. De Cesaris^1^, P. Geppetti^1^

#### ^1^Foundation Primary Headache and Stress, Health Sciences, Florence, Italy

##### **Correspondence:** L. F. Iannone

**Question:** To assess the long-term therapeutic impact of anti-calcitonin gene related protein (CGRP) monoclonal antibodies (anti-CGRP mAbs) in drugs-resistant patients with chronic migraine (CM) with no or partial response to OnabotulinumtoxinA (BTX).

**Methods:** A retrospective, cohort study, enrolling 78 severe CM patients (>80% with medication-overuse [MO]), resistant to ≥3 preventative treatments, and treated with BTX and then with anti-CGRP mAbs. The study consisted of two observational periods of 9 months. A varying non-observational period of at least 6-months occurred after the last BTX treatment. The primary endpoints were the absolute change from baseline in monthly headache days (MHDs), response rates and persistence in MO at 3-, 6- and 9-months follow-up in the two cohorts separately. The secondary endpoint was the change in acute medications use per month. Finally, we performed a last observation carried forward analysis for primary and secondary endpoints.

**Results:** After nine months of treatment, retention rate ranged from 91.0% to 62.2% in the BTX-A cohort and from 96.2%, to 76.9% in the anti-CGRP mAbs cohort (fig. 1). Approximatively 20% of patients discontinued both treatments due to inefficacy. After 9 months of treatment, 22.4% with BTX-A and 65.0% with anti-CGRP mAbs achieved a ≥50% response (fig. 2). Two patients were migraine-free in the CGRP cohort. BTX-A and anti-CGRP mAbs reduced MHDs at month-9.0 by -5.0 and -12.0, respectively, and decreased the number of MO patients at month-9 (75.5% and 25% persisted in MO, respectively [fig. 3]). Only two patients discontinued treatments due to AEs.

**Conclusions:** Our findings in drugs-resistant CM patients indicate that patients who discontinued BTX-A undergoing anti-CGRP mAbs treatment showed a substantial clinical improvement in migraine related outcomes. Stopping BTX-A in patients with no response/partial response after the first two cycles and switching to an anti-CGRP mAb appears a viable option.

**Fig. 1 (abstract A8).**
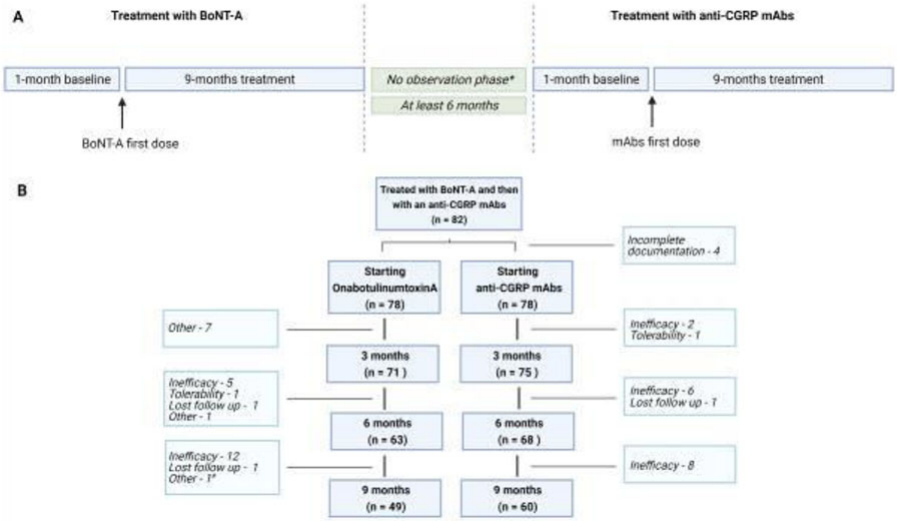
See text for description.

**Fig. 2 (abstract A8).**
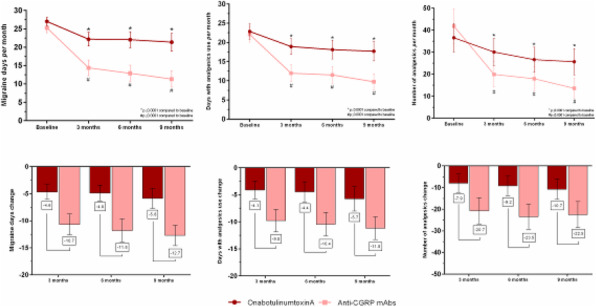
See text for description.

## A9 Placebo effects in clinical trials of anti-CGRP monoclonal antibodies for migraine prevention

### S. Regnier, X. Ying Lee

#### Lundbeck, Copenhagen, Denmark

**OBJECTIVE:** Commonly used indirect treatment comparison (ITC) methods, such as network meta-analyses, assume the presence of a common comparator across trials. For placebo (PBO)-controlled trials, PBO usually serves as the common comparator. Recent literature indicates that differences in route of administration (ROA) across PBO arms of clinical trials in pain disorders may contribute to differences in PBO effect. We conducted a meta-regression on PBO data from anti-CGRP monoclonal antibody (mAb) trials for migraine prevention to quantify the potential impact of ROA on PBO reduction in monthly migraine days (MMDs) across weeks 1–12.

**METHODS**: A systematic literature review was conducted in June 2021 to identify relevant PBO data from randomized clinical trials of anti-CGRP mAbs in migraine prevention. A generalized linear model was fitted to the extracted PBO data, with migraine type (EM/CM) and proportion of patients with ≥2 failed preventives as covariates.

**RESULTS:** An IV ROA for the PBO arm was a predictor for higher MMD reduction while episodic migraine, and a higher proportion of patients having ≥2 failed preventives were predictors of lower MMD reduction over weeks 1–12 in the PBO arms of studies investigating anti-CGRP mAbs.

**CONCLUSIONS:** Our results indicate that PBO ROA differences may warrant the use of ITC methods which do not assume the presence of a common comparator when comparing anti-CGRP mAbs in migraine prevention. Further research should be considered.

## A10 Amylin 1 receptors produce delayed activation and sensitization of trigeminovascular neurons

### A. Labastida-Ramirez, E. Rubio-Beltran, P. Holland, J. Hoffmann

#### King's College London, Headache Group, London, United Kingdom

##### **Correspondence:** A. Labastida-Ramirez

**Objective:** Investigate in an in vivo model the role of amylin 1 (AMY_1_) receptor activation in the modulation of the trigeminal nociceptive system in rats during the different phases of the estrous cycle, and compare it to the responses observed in males.

**Methods:** We recorded neuronal activity in male and female rats with extracellular electrodes placed within the trigeminocervical complex and examined the effects of targeting the AMY_1_ receptor on ongoing spontaneous and dural-evoked firing rates of central trigeminovascular neurons. The selective AMY_1_ receptor agonist pramlintide and AMY_1_ receptor antagonist AC187, were used for the present study. The different stages of the estrous cycle were identified and assigned by a blinded experimenter through Cresyl violet-stained vaginal smears.

**Results:** Compared to males (n=6), intravenous administration of pramlintide (6 μg/kg) significantly augmented the ongoing spontaneous activity and dural-evoked neuronal responses in the trigeminocervical complex, only during the estrus and early diestrus phases of the female estrous cycle, whereas this effect was not observed in the metestrus, proestrus and late diestrus phases (n=4-6 per group). Moreover, compared to vehicle (0.9% NaCl, n=5), intravenous administration of AC187 (6 μg/kg) significantly decreased the ongoing spontaneous and dural-evoked firing rates of central trigeminovascular neurons in males (n=4).

**Conclusion:** Our data support that activation of the AMY_1_ receptor modulates the trigeminal nociceptive system and that this effect is most pronounced during the estrus and early diestrus phases of the mesntrual cycle. The data also support selective AMY_1_ antagonists as novel and potentially effective targets for the treatment of migraine.

## A11 Differential mechanism of action of erenumab and gepants in human isolated coronary arteries

### T. de Vries^1^, A. van den Bogaerdt^2^, A. H. J. Danser^1^, J. Snellman^3^, J. Bussiere^4^, A. Maassen van den Brink^1^

#### ^1^Erasmus Medical Center, Internal Medicine, division of Pharmacology and Vascular Medicine, Rotterdam, Netherlands; ^2^ETB-BISLIFE, Heart Valve Department, Beverwijk, Netherlands; ^3^Novartis, Basel, Switzerland; ^4^Amgen Inc., Thousand Oaks, CA, United States

##### **Correspondence:** T. de Vries

**Objective**

Multiple drugs targeting the calcitonin gene-related peptide (CGRP) pathway have been developed for the acute and preventive treatment of migraine. In this study, the effect of the monoclonal antibody erenumab in combination with the acute anti-migraine medication rimegepant, olcegepant or sumatriptan (5-HT-R agonist), on CGRP-induced vasorelaxation was investigated in human isolated coronary arteries (HCA).

**Methods**

HCA segments from 17 donors (11 female and 6 male, 53±3 years) were incubated overnight with 3 μM erenumab, which is the concentration of erenumab causing a maximum rightward shift of relaxations to CGRP. Next, the segments were mounted on a Mulvany myograph system, in the presence of 3 μM erenumab, and precontracted with 30 mM KCl and subsequently exposed to CGRP. Rimegepant, olcegepant or sumatriptan was added in increasing concentrations to assess whether these compounds did exert additional CGRP-blocking effects on top of erenumab. In addition, full concentration-response curves to CGRP, adrenomedullin or pramlintide were constructed in HCA segments incubated with or without erenumab (3 μM) and/or olcegepant (1 μM).

**Results**

The relaxation caused by CGRP in segments incubated with 3 μM erenumab was reversed by rimegepant (91%), olcegepant (92%) and sumatriptan (134%, physiological antagonism). Olcegepant seemed to further shift the concentration-response curve to CGRP when administered on top of erenumab, while no further shift of the responses to adrenomedullin and pramlintide was observed.

**Conclusion**

Both rimegepant and olcegepant exert additional effects on top of a maximal effect of erenumab, suggesting that the mechanism of action of erenumab and gepants may not be identical. In contrast, olcegepant does not induce additional effects on top of erenumab for the agonists adrenomedullin and pramlintide. This suggests that different receptor populations may mediate responses to CGRP, adrenomedullin and pramlintide in HCA.

## A12 Revisiting RAMP1 expression: Characterisation of antibodies against receptor activity-modifying protein 1 (RAMP1)

### T. Rees^1^, E. Hendrikse^1^, Z. Tasma^1^, M. Garelja^2^, C. Walker^1^, D. Hay^2^

#### ^1^University of Auckland, School of Biological Sciences, Auckland, New Zealand; ^2^University of Otago, Department of Pharmacology and Toxicology, Dunedin, New Zealand

##### **Correspondence:** T. Rees

**Objective:** Calcitonin gene-related peptide (CGRP) is well-established as a key component of migraine pathophysiology, yielding effective migraine therapeutics. Further refinement of these treatments requires a more in-depth understanding of CGRP's actions at its receptors, which contain a core accessory protein subunit: receptor activity-modifying protein 1 (RAMP1). Our understanding of RAMP1 expression at the cellular level is incomplete, partly due to the challenges in the availability and identification of specific and validated antibody tools.

**Methods:** We profiled antibodies for immunodetection of RAMP1 using western blotting,

immunocytochemistry, and immunohistochemistry, including using RAMP1 knockout mouse tissue.

**Results:** Most antibodies could detect RAMP1 protein in western blotting and immunocytochemistry using transfected cells. Selected antibodies were profiled further, and two antibodies (844, ab256575) could detect a RAMP1-like band in western blots of rodent brain but not RAMP1 knockout mice. However, cross-reactivity with other proteins was evident for all antibodies. In immunohistochemistry clear conclusions about RAMP1 anatomical localization were prevented because each antibody unexpectedly detected distinct patterns of immunoreactivity.

**Conclusions:** Although most antibodies were capable of detecting RAMP1, their ability to also detect off-target proteins means that immunoreactivity produced by RAMP1 antibodies (including 844) in nervous tissue cannot be confidently attributed to RAMP1 in immunohistochemical applications. RAMP1 expression in nervous tissue therefore needs to be revisited. Our results have implications for using RAMP1 antibodies for any immunohistochemical investigation in any tissue. Furthermore, as RAMP1 is important for other GPCR/ligand pairings, our results have broader significance beyond the CGRP field.

## A13 One-quarter of individuals with weekly headache have never consulted a medical doctor: A Danish nationwide cross-sectional survey

### T. P. Do^1^, S. Stefansen^1^, M. Dømgaard^1^, T. Steiner^2,3^, M. Ashina^1^

#### ^1^Danish Headache Center, Glostrup, Denmark; ^2^Norwegian University of Science and Technology, Trondheim, Norway; ^3^Imperial College London, London, United Kingdom

##### **Correspondence:** T. P. Do

**INTRODUCTION:** Large numbers of people with headache who would benefit are not reached by headache services. Among the causes are poor or disorganized provision of headache services, but reluctance to seek healthcare has frequently been identified as a significant barrier. We conducted a national survey of people with headache to assess the extent of this problem in Denmark, a country with well organized, highly resourced, and readily accessible services.

**METHODS:** We conducted a nationwide cross-sectional survey of adults ≥18 years old in Denmark reporting at least one headache day in the last year. The survey investigated five items: (1) disease burden, (2) social life, (3) presenteeism, (4) social support, and (5) healthcare utilization.

**RESULTS:** We included 6,567 respondents from May 2021 to June 2021; 70.2% were female, 39.8% male, and mean age was 43.2±13.4 years. Of the respondents, 54.2% reported headache at least once a week, 33.4% reported headache a couple of times a month, and 12.4% reported headache a couple of times a year. Two-thirds of respondents (66.6%) reported that headache limited their social lives occasionally or frequently. Most respondents (86.8%) reported going to work or attending educational activities occasionally or more frequently even though they had headache. Half of the respondents (49.5%) experienced lack of understanding of their headaches from people occasionally or more frequently. Almost half of respondents (43.7%) had never consulted a medical doctor for their headache; even of those with weekly headache, more than a quarter (28.3%) had never done so in their lifetimes.

**CONCLUSIONS:** Headache disorders continue to be a problem, even in a high-income country with free and easily accessible headache services.

## A14 Changes in Acute Headache Medication Use Among Patients With Chronic Migraine and Medication-Overuse Headache: An Exploratory Analysis of PROMISE-2

### R. Cowan^1^, M. Marmura^2^, H. C. Diener^3^, A. Starling^4^, J. Schim^5^, J. Hirman^6^, T. Brevig^7^, R. Cady^8^

#### ^1^Stanford Health Care, Palo Alto, CA, United States; ^2^Thomas Jefferson University, Headache Center, Philadelphia, PA, United States; ^3^Medical Faculty of the University of Duisburg-Essen, Institute for Medical Informatics, Biometry and Epidemiology, Essen, Germany; ^4^Mayo Clinic, Scottsdale, AZ, United States; ^5^Headache Center of Southern California, Carlsbad, CA, United States; ^6^Pacific Northwest Statistical Consulting, Inc, Woodinville, WA, United States; ^7^Lundbeck, Valby, Denmark; ^8^Lundbeck, Deerfield, IL, United States

##### **Correspondence:** H. C. Diener

**OBJECTIVE:** This post hoc analysis evaluated changes in days of acute headache medication (AHM) use among patients with medication-overuse headache (MOH) from the PROMISE-2 trial.

**METHODS:** PROMISE-2 (NCT02974153) was a double-blind, randomized, placebo-controlled phase 3 study evaluating safety and efficacy of eptinezumab (100 or 300 mg) in adults with chronic migraine (CM). In eDiaries, patients indicated daily whether they experienced a headache, used AHM, and type of AHM used.

**RESULTS:** Of the 1072 patients with CM in PROMISE-2, 431 (40.2%) were formally diagnosed with MOH. The 28-day screening/baseline period comprised 18,504 (eptinezumab) and 9,560 (placebo) study days with medication data; Weeks 1-24 comprised 100,390 and 50,632 days, respectively. The proportion of headache days and AHM use decreased –29.1%-points (eptinezumab) *vs* –18.4%-points (placebo), and the proportion reporting no headache or AHM use increased 33.8%-points *vs* 23.6%-points, respectively. The proportion with headache and no AHM use decreased 6.1%-points and –7.1%-points for eptinezumab and placebo, respectively. Triptans were the most used AHMs at baseline (eptinezumab, 20.1%; placebo 19.3%), but triptan use decreased more with eptinezumab *vs* placebo (–11.8 *vs* –7.2%-points).

**CONCLUSIONS:** Eptinezumab was associated with greater declines in headache frequency and days of AHM use *vs* placebo in patients with a dual diagnosis of CM and MOH, especially the subgroup of patients experiencing ≥50% response.

## A15 Antibodies anti-CGRP in patients with fibromyalgia and resistant migraine, are they just as effective?

### C. Nieves Castellanos, M. Olivier, M. I. Fabrich Marín, S. Díaz Insa

#### Hospital Universitari i Politécnic la Fe de Valencia, Headache Unit, Valencia, Spain

##### **Correspondence:** C. Nieves Castellanos

QUESTION

Fibromyalgia is a frequent chronic disease and appears frequently with chronic migraine in our patient. We design this sub-study to evaluate if patients with fibromyalgia and migraine responds to antibodies anti-CGRP (a-CGRP) as well as patients without fibromyalgia.

METHODS

We present a sub-analysis of a prospective study of patients with resistant migraine treated with a-CGRP analyzing days of migraine (MHD), headache (HHD) and symptomatic treatment (MusD) as well as scales (HIT-6, MIDAS, pain catastrophizing scale, quality of life (MsQol)). We compared the response to a-CGRP at 3 ant 6 months in patients with fibromyalgia (Fi) and without it (No-Fi)

RESULTS

We included 53 patients Fi and 283 No-Fi. 78% of women and mean age of 46 years in the group no-Fi and 98% of women and mean age of 53 in the group Fi, 5 treatment failures in both groups.

In the group No-Fi before using the a-CGRP they have 22,6 HHD, 19,4 MHD and 19 MsuD. After 3 months, there is a reduction of 9,7 HHD, 7,8 MHD and 8 MsuD. After 6 months 10,7 HHD, 9,6 MHD, 8,6 MsuD. At 6 months, HIT-6 reduces 9,5 points, MIDAS 46,5 points and MsQol increases 20,5 points.

In the group Fi before they have 23,9 HHD, 19,9 MHD and 18,9 MsuD. After 3 months, there is a reduction of 8,9 HHD, 6,4 MHD and 5,8 MsuD. After 6 months 10,7 HHD, 8,7 MHD, 8,1 MsuD. At 6 months, HIT-6 reduces 8,8 points, MIDAS 63,5 points and MsQol increases 22,3 points.

30% in group Fi reported adverse events and 36% in No-Fi.

CONCLUSIONS

Patients with fibromyalgia responds to a-CGRP too, although, according with our study, the improvement seems to be more delayed compared to patients without fibromyalgia. The quality of life and the disability improve slightly more in the group with fibromyalgia than in the group without it. There is no difference in the rate of adverse events.

We consider that with these results, patients with fibromyalgia and migraine should be treated with a-CGRP as well as patients without it.

## A16 The Idiopathic Intracranial Hypertension Life Long study: Evaluation of prognostic factors and outcomes

### M. Thaller^1,2,3^, V. Homer^4^, S. Mollan^1,5^, A. Sinclair^1,2,3^

#### ^1^University of Birmingham, Institute of metabolism and systems research, Birmingham, United Kingdom; ^2^University Hospitals Birmingham NHS Foundation Trust, Neurology, Birmingham, United Kingdom; ^3^Birmingham Health Partners, Centre for Endocrinology, Birmingham, United Kingdom; ^4^University of Birmingham, Cancer Research (UK) Clinical Trials Unit, Birmingham, United Kingdom; ^5^University Hospitals Birmingham NHS Foundation Trust, Birmingham Neuro-Ophthalmology, Birmingham, United Kingdom

##### **Correspondence:** M. Thaller

**Question**

There is limited longitudinal data evaluating visual and headache outcomes in Idiopathic intracranial hypertension (IIH). We aimed to evaluate the long-term outcomes in a large prospective real-world cohort of patients with IIH and prognostic factors.

**Methods**

A longitudinal clinical examination dataset was analysed from the prospectively collected IIH:Life database 2012-2021. Data included demographics and disease status. Visual outcomes included visual acuity (LogMAR), perimetric mean deviation (MD) (Humphrey 24–2 central threshold) and papilloedema (optical coherence tomography (OCT) imaging measurements). Headache frequency (days per month) and the headache impact test-6 questionnaire (HIT-6) were noted. We analysed the key variables for prognostic outcomes of vision and headache, focusing on the medically treated cohort.

**Results**

490 had a confirmed diagnosis of IIH. 98% were female with a mean body mass index (BMI) of 38 kg/m2. Those with the highest OCT RNFL had the worst visual outcomes, but there was a delay of over 12 months before the visual field and OCT measurements revealed this decline. In the medically managed cohort (n=426) visual outcomes were good. Regression analyses showed change in BMI and disease duration had the most influence on vision.

Those who were managed medically and had active IIH (n=281) there was a high headache burden and risk of high headache frequency was found to be associated with a personal migraine history and daily headache at diagnosis. There was a low relapse rate of 3.7%, which was associated with weight gain.

**Conclusions**

Those with the most elevation of their RNFL had worse long-term visual outcomes which only became apparent in longer term follow-up after 12 months. In a medically managed cohort of people with IIH disease duration and change in BMI were the key factors in influencing visual outcomes. The headache burden was high, and targeted therapy remained an unmet clinical need.

## A17 Dysregulation of multiple metabolic pathways related to amino acid and lipid metabolism in idiopathic intracranial hypertension: A non-targeted case control and longitudinal metabolomic study.

### Z. Alimajstorovic

#### University of Birmingham, Institute of Metabolism and Systems Research, Birmingham, United Kingdom

Idiopathic intracranial hypertension (IIH) is a disease characterised by raised intracranial pressure (ICP) and occurs predominantly in women with obesity; however the underlying molecular pathogenesis is not fully understood. We have applied untargeted metabolomic analysis using ultra high performance liquid chromatography-mass spectrometry to characterise the cerebrospinal fluid (CSF) and serum metabolite profiles in IIH compared to control subjects and to probe underlying disease mechanisms.

CSF and serum were collected from IIH patients (n=66) with active disease (lumbar puncture pressure >25 cmCSF and Frisén papilloedema grade ≥1) at baseline and again at 12 months following therapeutic weight loss. Analogous samples were collected at baseline from gender and body mass index matched healthy controls with obesity (n=20). We identified two annotated metabolite features in CSF; (1) formylpyruvate and (2) maleylpyruvate and/or fumarylpyruvate isomers, which were present at lower concentrations in IIH compared to control subjects and returned to relative values of control subjects following weight loss. These metabolites showed the opposite trend in serum. Several amino acid and fatty acid metabolic pathways were repeatedly perturbed in serum. Arginine metabolism and arginine biosynthesis pathways were also altered in CSF and serum in relation to IIH symptoms and remission. Lipid classes related to obesity were observed as biologically important in serum supporting the link between obesity and lipid metabolism in IIH.

These results support IIH being a systemic metabolic disease, not merely a pathology of the central nervous system and optic nerve. The perturbed pathways were also associated with disease clinical features and normalised over 12 months in line with disease remission. Perturbation of these metabolic pathways provides initial understanding of disease dysregulation in IIH and require further mechanistic evaluation.

## A18 Real-world evidence of galcanezumab for migraine treatment in Japan: A retrospective analysis

### T. Takizawa^1^, S. Ohtani^1,2^, N. Watanabe^1^, N. Miyazaki^3^, K. Ishizuchi^1^, K. Sekiguchi^1^, C. Iba^1^, M. Shibata^4^, R. Takemura^3^, S. Hori^2^, J. Nakahara^1^

#### ^1^Keio University School of Medicine, Neurology, Tokyo, Japan; ^2^Keio University Faculty of Pharmacy, Division of Drug Informatics, Tokyo, Japan; ^3^Keio University Hospital, Biostatistics Unit, Clinical and Translational Research Center, Tokyo, Japan; ^4^Tokyo Dental College Ichikawa General Hospital, Neurology, Ichikawa, Japan

##### **Correspondence:** T. Takizawa

**Question:** Galcanezumab is the first anti-calcitonin gene-related peptide monoclonal antibody approved in Japan. How are the efficacy and safety of galcanezumab in patients with migraine in a real-world setting in Japan?

**Methods:** We retrospectively analyzed patients with migraine who received three doses of galcanezumab between August 2021 and February 2022 at the Keio University Hospital. We assessed changes in monthly migraine days (MMD), responder rate (RR), and migraine-associated and premonitory symptoms. We also investigated injection site reactions and adverse events.

**Results:** Fifty-two patients received three doses of galcanezumab during the study period. Compared to baseline, the MMDs decreased by 5.9 days (95% confidence interval, 4.2–7.7) at 3 months. The 50% RR was 61.5% at 3 months. A total of 64.9%, 50.0%, and 63.9% of patients showed improvement in the severity of photophobia, phonophobia, and nausea/vomiting, respectively. Premonitory symptoms persisted in 62.5% of patients. Moreover, injection site reaction was the most common adverse event (34.6%).

**Conclusion:** This study revealed the efficacy and safety of galcanezumab for migraineurs in Japan. Galcanezumab also improved migraine-associated symptoms. However, despite a reduction in headaches, premonitory symptoms persisted in >50% of the patients at 3 months, possibly due to a peripheral action of anti-calcitonin gene-related peptide monoclonal antibodies.

## A19 Decreased plasma RANTES/CCL5 concentration in headache-free episodic migraine patients

### K. Gecse^1,2^, T. Nagy^1,2,3^, Z. Környei^4^, Á. Dénes^4^, G. Bagdy^1,5,6^, G. Juhasz^1,2^

#### ^1^Semmelweis University, Faculty of Pharmacy, Department of Pharmacodynamics, Budapest, Hungary; ^2^Semmelweis University, SE-NAP2 Genetic Brain Imaging Migraine Research Group, Budapest, Hungary; ^3^Budapest University of Technology and Economics, Department of Measurement and Information Systems, Faculty of Electrical Engineering and Informatics, Budapest, Hungary; ^4^Institute of Experimental Medicine, Momentum Laboratory of Neuroimmunology, Budapest, Hungary; ^5^Semmelweis University, MTA-SE Neuropsychopharmacology and Neurochemistry Research Group, Budapest, Hungary; ^6^Semmelweis University, NAP-2-SE New Antidepressant Target Research Group, Budapest, Hungary

##### **Correspondence:** K. Gecse

**Introduction**: The regulated on activation and normal T-cell expressed and secreted (RANTES/CCL5) is a chemotactic protein that beyond to chemoattraction is involved in nociception and trigeminal pain. Previous study demonstrated an increased serum RANTES/CCL5 concentration in migraineurs during migraine attack. Additionally, higher RANTES/CCL5 concentration distinguish migraine patients compared to tension-type headache patients. However, the RANTES/CCL5 concentration in headache-free period of migraineurs is contradictory in the literature.

**Methods**: Blood samples were collected in two independent time-points to measure plasma RANTES/CCL5 concentration of 21 females with episodic migraine without aura and 22 healthy control females The difference in plasma RANTES/CCL5 between migraine and control group was calculated with Mann-Whitney U-test using SPSS27.

**Results**: RANTES/CCL5 concentration were decreased in migraine patients compared[K1] to healthy controls in both blood samplings (1.BS: U=329, p=0.017; 2.BS: U=312, p=0.049).

**Conclusion**: Previous studies reported that lower RANTES/CCL5 concentration was associated with higher flow-mediated dilation in vessels. Thus, the decreased serum RANTES/CCL5 concentration replicated in two independent measures may be the sign of vascular hypersensitivity in migraine patients between attacks. The decreased RANTES/CCL5 concentration in interictal period may be a predisposing factor for migraine attack, while the increased RANTES/CCL5 concentration might be associated with migraine pain in ictal period. These findings suggest that RANTES/CCL5 might play a complex role in migraine pathophysiology through its pro-inflammatory, nociceptive and vascular effects that should be further explored.

**Funding**: EFOP-3.6.3-VEKOP-16-2017-00009; 2017-1.2.1-NKP-2017-00002, KTIA_13_NAPA-II/14, KTIA_NAP_13-1-2013- 0001, KTIA_NAP_13-2- 2015-0001; 2020-4.1.1.-TKP2020; TKP2021-EGA-25; 2019-2.1.7-ERA-NET-2020-00005.

## A20 Functional connectivity changes in patients with complex migraine aura: beyond the visual network

### A. Russo, M. Silvestro, F. Esposito, M. Cirillo, A. Tessitore, G. Tedeschi

#### University of Campania "Luigi Vanvitelli", Advanced medical and surgical sciences, Naples, Italy

##### **Correspondence:** A. Russo

Background and purpose: Although the majority of migraine with aura (MwA) patients experience simple visual aura, a discrete percentage also report somatosensory, dysphasic or motor symptoms (the so-called complex auras). The wide aura clinical spectrum led to an investigation of whether the heterogeneity of the aura phenomenon could be produced by different neural correlates, suggesting an increased visual cortical excitability in complex MwA. The aim was to explore whether complex MwA patients are characterized by more pronounced connectivity changes of the visual network and whether functional abnormalities may extend beyond the visual network encompassing also the sensorimotor network in complex MwA patients compared to simple visual MwA patients.

Methods: By using a resting-state functional magnetic resonance imaging approach, the resting-state functional connectivity (RS-Fc) of both visual and sensorimotor networks in 20 complex MwA patients was compared with 20 simple visual MwA patients and 20 migraine without aura patients.

Results: Complex MwA patients showed a significantly higher RS-Fc of the left lingual gyrus, within the visual network, and of the right anterior insula, within the sensorimotor network, compared to both simple visual MwA and migraine without aura patients (p < 0.001). The abnormal right anterior insula RS-Fc was able to discriminate complex MwA patients from simple aura MwA patients as demonstrated by logistic regression analysis (area under the curve 0.83).

Conclusion: Our findings suggest that higher extrastriate RS-Fc might promote cortical spreading depression onset representing the neural correlate of simple visual aura that can propagate to sensorimotor regions if an increased insula RS-Fc coexists, leading to complex aura phenotypes.

**Fig. 1 (abstract A20).**
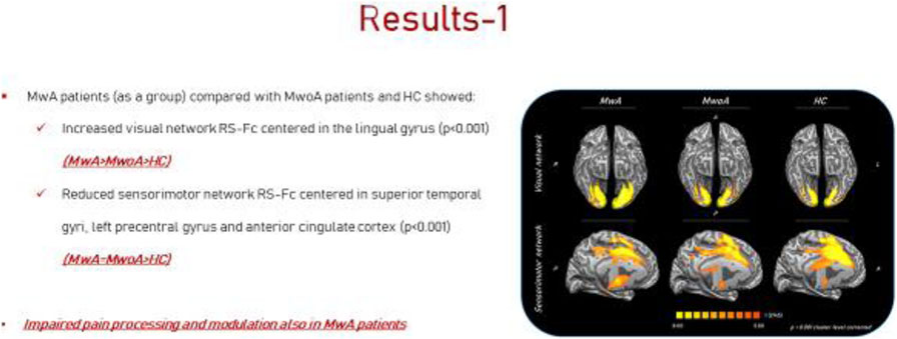
See text for description.

**Fig. 2 (abstract A20).**
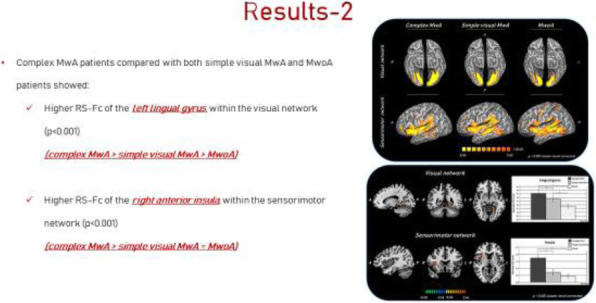
See text for description.

## A21 DNA methylation changes associated with treatment response in chronic migraine

### D. Mehta^1^, I. de Boer^2^, H. Sutherland^3^, J. Pijpers^2^, C. Bron^3^, C. Bainomugisa^1^, L. Haupt^3^, A. van den Maagdenberg^2,4^, L. Griffiths^3^, D. Nyholt^5^, G. Terwindt^2,5^

#### ^1^Queensland University of Technology, Centre for Genomics and Personalised Health, Faculty of Health, Queensland, Australia; ^2^Leiden University Medical Center, Neurology, Leiden, Netherlands; ^3^Queensland University of Technology, Genomics Research Centre, Centre for Genomics and Personalised Health, School of Biomedical Sciences, Brisbane, Australia; ^4^Leiden University Medical Center, Human Genetics, Leiden, Netherlands; ^5^Queensland University of Technology, School of Biomedical Sciences, Faculty of Health, and Centre for Genomics and Personalised Health, Brisbane, Australia

##### **Correspondence:** I. de Boer

**Objective:** The mechanisms behind the transformation of episodic migraine to chronic migraine and *vice versa* have not yet been elucidated. Epigenetic changes are implicated in this process. If treatment results in conversion back to episodic migraine these epigenetic processes might also be reverted. We aimed to identify DNA methylation changes associated with treatment response in chronic migraine patients with medication overuse.

**Methods:** A longitudinal epigenome-wide association study was performed as part of the Chronification and Reversibility of Migraine (CHARM) study. Blood was taken from chronic migraine patients (n = 98) at baseline and after a 12-week withdrawal period. Treatment responders, patients with ≥ 50% reduction in monthly headache days (MHD), were compared with non-responders to identify methylation changes associated with treatment response. Similarly, ≥ 50% versus < 50% reduction in monthly migraine days (MMD) was compared. Sex-specific analyses were performed. Finally, it was evaluated whether DNA methylation status at baseline was predictive of treatment response after t = 12 weeks.

**Results:** At the genome-wide level, a change in DNA methylation at one CpG site within an intron of the *HDAC4* gene was associated with MHD response (*p* = 9.42×10^–8^) (Fig 1A). Sex-specific analyses revealed two CpG sites associated with MHD response, proximal to *DLGAP2* for women (*p* = 1.11x10^-7^) and *STS5/AKIP1* for men (*p* = 8.67x10^-8^). Five CpG sites were associated with MMD response in men: *ZAN* (*p* = 2.41x10^-8^), *ZNF248* (*p* = 2.52x10^-8^), *H4C2* (*p* = 2.87x10^-8^), between *RIT2/SYT4* (*p* = 4.29x10^-8^), and between *NRXN1/ASB3* (*p* = 6.44x10^-8^). Baseline methylation at one CpG within *MARK3* was predictive of MMD response at 12 weeks.

**Conclusion:** Global and sex-specific DNA methylation changes are associated with treatment response in chronic migraine. Moreover, DNA methylation status at baseline might be predictive of treatment response.

**Fig. 1 (abstract A21).**
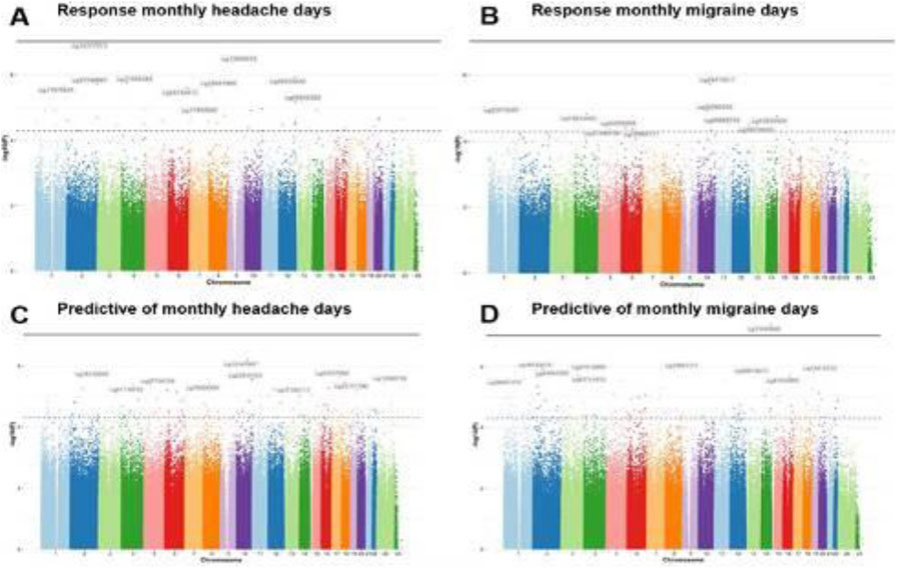
Manhattan plots from the epigenome-wide association study. Manhattan plot showing the -log10 p value for each CpG site. The threshold for genome wide significance (p <9.42 x 10^-1^) is indicated by a continues line. The threshold for suggestive genome wide significance (p < 5 x 10^-1^) is indicated by a striped line. Changes in DNA methylation in monthly headache days (MHD) responders vs non-responders (A): changes in DNA methylation in monthly migraine days (MMD) responders vs non-responders (B). Predictive value of DNA methylation profiles at baseline of favourable treatment response for MHD (C) and MMD (D)

## A22 Putative predictors of super-response to CGRP monoclonal antibodies

### A. R. Pinheiro, A. Rego, P. Neves, S. Delgado, J. Dionísio, B. Madureira, J. Costa, D. Carapinha, Â. Abreu, M. Saraiva, E. Parreira

#### Hospital Professor Doutor Fernando Fonseca, Neurology, Lisbon, Portugal

##### **Correspondence:** A. R. Pinheiro

**Question:** Are there putative predictors of super-response to CGRP monoclonal antibodies?

**Methods:** This was a unicentric prospective study between February 2019 and April 2022. We collected demographic, clinical, comorbid, and therapeutic data. We applied validated clinical scales to assess baseline severity and therapeutic response. We defined super-responders as patients who achieved a consistent ≥75% reduction in migraine frequency after 6 months of treatment and non-responders as patients with reduction <25%. We used SPSS v.23 for statistical analysis and compared groups of super-responders to non-responders using univariate linear logistic regression.

**Results:** From a total of 63 patients and after excluding 8 (12.8%) (short follow-up), we analyzed 42 patients. Median age was 44 years (IQR 52) and 41 (97.6%) were women. Twenty-one (50%) had chronic migraine with median duration of 22.9 years (IQR 56), 17 (40.5%) had medication overuse and 24 (57.1%) responded to triptans. We treated patients with Erenumab (n=31), Fremanezumab (n=7) and Galcanezumab (n=4). We had 29 super-responders and 13 non-responders. We found a statistically significant association between super-responders and lower baseline frequency of migraine (OR=0.901), episodic migraine (OR=0.096) and response to triptans (OR=5.000). After treatment, there was a statistically significant association between reduction of migraine frequency and lower HURT scale score (OR=0.771) and decreased headache intensity (OR=0.644). We noticed a trend towards statistically significant results between super-responders and >3 failed preventives (p=0.072) and lower baseline intense episodes (p=0.085).

**Conclusions:** In CGRP monoclonal antibody treatment, episodic migraine, response to triptans and lower baseline intense episodes may be potential predictors of super-response. HURT scale may be appropriate to monitor these patients. This real-life data may allow better selection and management of patients.

## A23 Pharmacogenetic assessments of treatment response in cluster headache

### A. S. Petersen^1^, M. Barloese^1,2^, N. Lund^1^, A. S. Pedersen^1^, M. L. K. Søborg^1^, M. A. Chalmer^1^, I. Callesen^1^, B. S. Winsvold^3,4,5^, J. A. Zwart^3,4,5^, S. R. Ostrowski^6^, O. B. Pedersen^7^, F. T. Sellebjerg^8^, H. B. Søndergaard^8^, M. B. Hansen^8^, R. H. Jensen^1^, T. F. Hansen^1^

#### ^1^Dansk Hovedpine Center, Rigshospitalet-Glostrup, Neurology, Glostrup, Denmark; ^2^Center of Functional and Diagnostic Imaging and Research, Copenhagen University, Hospital Hvidovre, Department of Clinical Physiology and Nuclear Medicine, Hvidovre, Denmark; ^3^Oslo University Hospital, Department of Research and Innovation, Division of Clinical Neuroscience, Oslo, Norway; ^4^Oslo University Hospital, Department of Neurology, Oslo, Norway; ^5^University of Oslo, Institute of Clinical Medicine, Faculty of Medicine, Oslo, Norway; ^6^University of Copenhagen, Rigshospitalet, Clinical Immunology, Copenhagen, Denmark; ^7^Zealand University Hospital, Department of Clinical Immunology, Køge, Denmark; ^8^Danish Multiple Sclerosis Center, Department of Neurology, Copenhagen University Hospital-Rigshospitalet, Neurology, Glostrup, Denmark

##### **Correspondence:** A. S. Petersen

Background: The response to cluster headache treatments has a high interindividual variation. To date, treatment response has only been assessed by candidate gene approach and no investigations into metabolic pathways have been performed.

Question: To investigate the association between polygenetic risk of cluster headache and treatment response to first line cluster headache treatments. Additionally we investigated known functional variants of *CYP3A4* and the response to verapamil1. Further, to replicate previous single nucleotide polymorphisms found to be associated with treatment response in cluster headache and/or migraine.

Methods: 508 cluster headache patients diagnosed according International Classification of Headache Disorders ere genotyped and participated in a semi-structured interview to evaluate treatment response. Polygenetic risk score was calculated by effect retrieved from meta-analyzing the latest two GWAS on cluster headache.

Results: We confirm previous findings that inferior treatment response associates with chronicity of cluster headache no evidence for response to first-line abortive and preventive treatment was predicted by a high genetic risk of cluster headache or functional variants of *CYP3A4*. We did not found support of the suggested genetic variants previously reported to be associated with treatment response to triptans or verapamil.

Conclusion: The clinically relevant variation in treatment response for cluster headache is unlikely to be influenced by genetic factors and other factors should be searched.

## A24 Salivary CGRP and erenumab response: towards precision medicine in migraine

### A. Alpuente, V. J. Gallardo, L. Asskour, E. Caronna, M. Torres-Ferrús, P. Pozo-Rosich

#### Vall d'Hebron University Hospital, Neurology, Barcelona, Spain

##### **Correspondence:** A. Alpuente

**Question:** It is still to be shown if there is a correlation between baseline CGRP levels and prediction of response to these treatments or if CGRP levels are modified and how with treatment. We aimed (i) to analyze salivary CGRP levels in migraine patients (ii) to predict erenumab response from pre-treatment CGRP levels and (iii) to evaluate CGRP change post-treatment.

**Methods:** This is a prospective observational study that measured salivary CGRP levels in healthy controls (HC), episodic migraine (EM) and chronic migraine (CM) patients. Participants collected saliva samples at baseline and, patients who were candidates to receive erenumab 140 mg, also collected saliva after 3 doses of treatment. We quantified CGRP-like immunoreactivity (CGRP-LI) by ELISA and we performed an analysis at baseline and post-treatment through generalized linear mixed models.

**Results:** At baseline, a higher headache frequency was associated with higher CGRP levels, being those even higher in presence of depressive symptoms. A cut-off point of 103.75 pg/mL was estimated to differentiate migraine from controls with an 80.3% of accuracy. We also found that higher pre-treatment salivary CGRP levels were statistically significantly associated to a higher probability of having 50% or greater reduction in headache frequency in EM patients, but not in CM. After 12-weeks of treatment with erenumab 140, salivary CGRP levels from patients within all spectrum of migraine frequency converged to similar CGRP values. In contrast, in patients with concomitant depressive symptoms, this convergence did not happen.

**Interpretation:** Patients with migraine not only have higher CGRP levels compared to controls, but also the presence of depressive symptoms seems to increase salivary CGRP levels and we have evidence, for the first time, that salivary CGRP concentration is associated with treatment response to erenumab.

**Fig. 1 (abstract A24).**
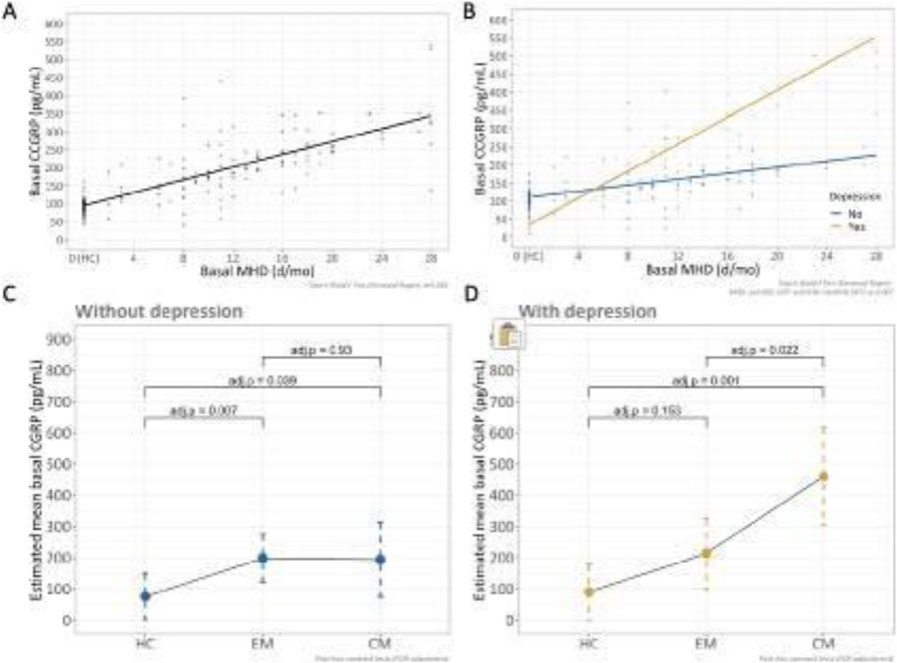
See text for description.

**Fig. 2 (abstract A24).**
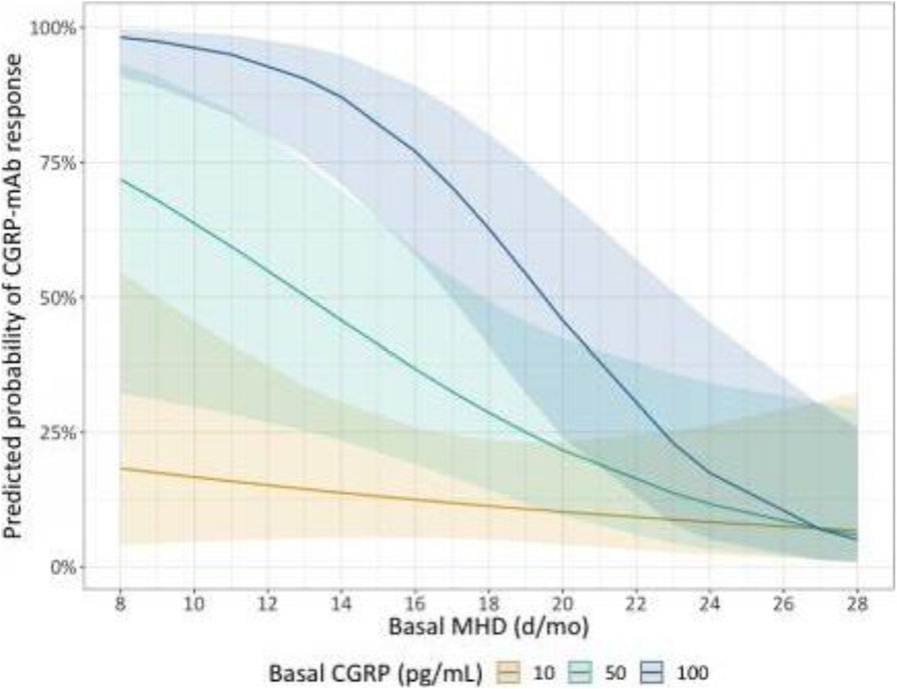
See text for description.

## A25 Effectiveness and safety of surgical treatment of classic and Idiopathic trigeminal neuralgia

### A. Martinez Viguera, J. M. Fernández Vidal, R. Collet Vidiella, G. Olmedo Saura, C. Toscano Prat, R. Sainz Torres, M. Borrell Pichot, T. I. Mederer Fernandez, B. Albertí Vall, R. Rodríguez Rodríguez, J. A. Aibar Duran, R. Belvís Nieto, N. Morollón Sánchez-Mateo, A. Martinez Viguera

#### Hospital de la Santa Creu i Sant Pau, Neurology, Barcelona, Spain

##### **Correspondence:** A. Martinez Viguera

**QUESTION**

Surgical treatment of trigeminal neuralgia (TN) is indicated in refractory cases. Different percutaneous and invasive techniques can be performed. This study aims to analyze the effectiveness and safety of these procedures.

**METHODS**

A retrospective observational single-center study of patients with classic or idiopathic refractory TN (failure to 3 different groups of drugs). Clinical, diagnostic-therapeutic, and clinical evolution data were collected.

**RESULTS**

We included 70 patients (60% female), with a median age at diagnosis of 56 [23-82], 48(69%) had classic TN.

The mean number of previous drugs used was 4.24 (SD±2.25).

47 patients (67%) were treated with microvascular decompression (MVD), being effective in 87% (52% total, 35% partial) and achieving medication withdrawal in 15. 11 (23.4%) presented complications; major, yet reversible, in 3 cases.

In 42 (60%) percutaneous techniques were used. 26 (37.1%) underwent radiofrequency thermocoagulation (1.77 times per patient on average) with a response rate of 60% (12% total, 48% partial), complications appeared in 7 (17.4%), 3 of which were major and persistent. 16 (34.8%) underwent balloon compression (1.25 times per patient) with 84% effectiveness (56% total, 25% partial), 4 (25%) minor complications were registered, 75% of which were persistent. In 17 patients (24%) both techniques were used (47% DCMV as the first option).

2 gangliolysis, 1 stereotactic radiosurgery, and 1 implantation of cortex stimulation device were performed, with no subsequent complications.

**CONCLUSIONS**

Surgical techniques are effective in patients with refractory TN, allowing to reduce medication use. The technique that offers the best results is MVD. Complications may appear in up to a quarter of patients, regardless of the technique used, being more frequent and persistent, contrary to expectations, in percutaneous procedures.

## A26 Effectiveness and tolerance of real-world medical treatment in classical and idiopathic trigeminal neuralgia. A series of 193 patients.

### G. Olmedo Saura, C. Toscano Prat, R. Collet Vidiella, J. M. Fernández Vidal, A. Martinez Viguera, B. Albertí Vall, T. I. Mederer Fernandez, R. Sainz Torres, M. Borrell Pichot, R. Belvís Nieto, N. Morollón Sánchez-Mateo

#### Hospital de la Santa Creu i Sant Pau, Neurology, Barcelona, Spain

##### **Correspondence:** G. Olmedo Saura

**Question**

Medical treatment recommendations in trigeminal neuralgia (TN) are based on few clinical trials with small samples and expert recommendations. Our aim is to describe the long-term effectiveness and tolerability of medical treatment in classical and idiopathic trigeminal neuralgia in real-world conditions.

**Methods**

We performed an observational study in which all patients with classical and idiopathic TN seen in our center were retrospectively collected.

**Results**

We included 193 patients (67% women), with a median age at diagnosis of 59 years [15-93], and a median follow-up of 3 years. 64% had classic TN. The median number of drugs used was 3.23 (SD ±2.26).

Of the 125 patients followed ≥2 years, 74 (59%) achieved sustained control. 66 (53%) with carbamazepine and derivatives, and 23 (18%) with gabapentinoids. 17 (25%) responded to the first drug used, and 19 (15%) responded to combined treatment.

Of the 66 patients followed ≥5 years, 28 (42%) achieved sustained control. 23 with carbamazepine and derivatives, and 7 with gabapentinoids. 17 (25%) responded to the first drug used, and 6 (9%) responded to combined treatment.

A total of 130 patients were treated with carbamazepine, 103 (54%) as first choice. A response was obtained in 44 (35%). 54 (42%) reported adverse effects.

**Conclusions**

Despite the increasing therapeutic offer of neuromodulators, carbamazepine is still the drug of choice, and with better effectiveness data. However, adverse effects are an important limitation. In a high percentage of patients with medical treatment we do not achieve a correct long-term control of TN.

## A27 Predictors of response to medical and surgical treatment in classical and idiopathic trigeminal neuralgia

### R. Collet Vidiella^1^, J. M. Fernández Vidal^1^, G. Olmedo Saura^1^, A. Martínez Viguera^1^, C. Toscano Prat^1^, M. Borrell Pichot^1^, R. Sainz Torres^1^, T. I. Mederer Fernandez^1^, B. Albertí Vall^1^, R. Rodríguez Rodríguez^2^, J. A. Aibar Duran^2^, R. Belvís Nieto^1^, N. Morollón Sánchez-Mateo^1^

#### ^1^Hospital de la Santa Creu i Sant Pau, Neurology, Barcelona, Spain; ^2^Hospital de la Santa Creu i Sant Pau, Neurosurgery, Barcelona, Spain

##### **Correspondence:** J. M. Fernández Vidal

**Question**

The proportion of patients that respond to medical and surgical treatment in trigeminal neuralgia (TN) is variable. We aimed to identify potential predictors of response to medical and surgical treatment in classical and idiopathic trigeminal neuralgia.

**Methods**

We conducted an observational, retrospective, and unicenter study in adults with classical or idiopathic TN. We analyzed the relationship between several epidemiologic, anatomic, and clinical characteristics and outcomes with the use of the chi-square or Fisher's exact test for categorical variables and with ANOVA for continuous variables. P-value ≤0.05 was considered significant. All reported P-values are two-sided.

**Results**

A total of 193 patients (67% women) were included, with a median age at the first visit of 64 [22-93] years.

Medical treatment response for ≥2 years was present in 59% of patients. Older age at the first visit (66 vs 58, p<0.01) and the presence of hypertension (47% vs 26%, p=0.03) were associated with response to medical treatment. No significant differences were found in terms of gender, affected branch, type of TN, or other comorbidities.

The effectiveness of microvascular decompression was 87%. A more significant number of previous drugs (3.7 vs 6.7, p=0.03) was associated with no response. No differences were found regarding other variables.

The effectiveness with thermocoagulation was 60%. V2 involvement (75% vs 17%, p=0.01) and pain in V2-V3 branches (91% vs 40%, p=0.01) were associated with effectiveness, while single branch involvement (27% vs 87%, p<0.01) to no response.

The effectiveness of mechanical compression was 84%. No response predictors were found.

**Conclusions**

Advanced age and hypertension were associated with a sustained response to medical treatment. A greater number of drugs before microvascular decompression was associated with a worse response. The involvement of branches V2 and V2-V3 was associated with effectiveness with thermocoagulation.

## A28 Inhibition of peripheral FAAH alleviates hyperalgesia induced by acute dural inflammation in experimental migraine

### M. Francavilla^1^, R. Greco^1^, C. Demartini^1^, A. Zanaboni^1,2^, C. Tassorelli^1,2^

#### ^1^IRCCS Mondino Foundation, Pavia, Italy; ^2^University of Pavia, Department of Brain and Behavioral Sciences, Pavia, Italy

##### **Correspondence:** M. Francavilla

Peripheral and central sensitization is an important pathophysiological process in migraine and its chronification, but the underlying mechanisms are largely unclear. Trigeminal hyperalgesia and/or facial allodynia have been tested in animal models to simulate clinical symptomatology and to investigate the mechanisms underlying migraine pain. Inhibition of the fatty-acid amide hydrolase (FAAH) activity, the enzyme that deactivates the endocannabinoid anandamide, may exert anti-nociceptive effects via the activation of peripheral cannabinoid (CB1) receptors. **Aim -** Here, we evaluated the effects of FAAH inhibition in a migraine model of peripheral sensitization obtained by evaluating trigeminal hypersensitivity at the orofacial formalin test in animals with dural inflammation. **Methods** – Dural inflammation was induced in male Sprague-Dawley rats with the infusion of an inflammatory soup (IS) onto the dura. Ten min after IS infusion, rats were treated, with the peripheral FAAH inhibitor URB937 (1mg/kg, i.p.) or vehicle. A first set of rats underwent the orofacial formalin test 2h after IS application, while a second experimental set was used to evaluate the expression of CGRP and pro-inflammatory cytokines in specific brain areas in toto. **Results -** IS infusion induced trigeminal hyperalgesia at the orofacial formalin test, associated with an increase in gene expression of CGRP and pro-inflammatory molecules in medulla-pons and trigeminal ganglia. URB937 administration inhibited IS-induced trigeminal hyperalgesia and the prevented the increase in gene expression of CGRP and pro-inflammatory molecules in the nervous tissues. **Conclusions** - The findings suggest that potentiation of the endocannabinoid tone, obtained via peripheral FAAH inhibition, may modulate trigeminal hyperalgesia induced by dural IS through the reduction of pro-inflammatory cytokines and CGRP synthesis at central and peripheral sites of the nervous system.

## A29 Aryl hydrocarbon receptors are involved in the pathogenesis of migraine

### Y. Mrad, A. Raoelina, R. Dallel, I. Ranchon-Cole, C. Alba-Delgado

#### Université Clermont Auvergne, CHU Clermont-Ferrand, Inserm, Neuro-Dol, Clermont-Ferrand, France

##### **Correspondence:** Y. Mrad

**Objective**: Migraine is a prevalent disorder, with episodic attacks. Some patients experience an increase in attack frequency and develop chronic migraine. However, the underlying mechanisms of this progression are uncertain. The noradrenergic locus coeruleus (LC) has been involved in migraine chronicity. Recent studies found that selective activation of LC alleviates pain by reducing neuroinflammation. As the aryl hydrocarbon receptors (AhRs) are key regulators of the brain inflammatory responses, we, therefore, investigate the contribution of LC-expressed AhRs in migraine progression.

**Methods**: Using immunohistochemical approaches, we assessed the intracellular distribution and the expression levels of AhRs in the LC in a mice model of migraine induced by systemic administration of isosorbide dinitrate (ISDN; a nitric oxide donor). We also explored the effect of systemic AhR activation (by the indole agonist ITE) or inhibition (by the pure antagonist TMF) on cutaneous mechanical hypersensitivity (CMH) induced by ISDN.

**Results**: In naïve mice, AhRs were detected in 47.1 ± 3.4 % of LC noradrenergic neurons. Repeated ISDN administration resulted in a significant increase in this *percentage* (56.7 ± 3.4%, P<0.05). Interestingly, morphological alterations of LC neurons together with an increase of the soma and *nucleus sizes* were also observed. Behavioral testing showed that single or repeated TMF or ITE administrations did not affect cephalic mechanical sensitivity in naïve mice. In addition, ITE did not worsen the ISDN-induced CMH. However, a single administration of TMF effectively blocked ISDN-induced acute CMH, while daily administration was unable to prevent long-lasting CMH.

**Conclusions**: These data highlight, for the first time, the involvement of AhRs in the initiation but not in the maintenance of migraine.

## A30 Orexin-A recovers sleep-deprivation induced periorbital allodynia in a preclinical migraine model

### E. Stanyer, J. Hoffmann, P. Holland

#### King's College London, Headache Group, London, United Kingdom

##### **Correspondence:** E. Stanyer

**Questions:** There is a bidirectional link between sleep and migraine with poor sleep reported as both a migraine trigger and symptom. Orofacial allodynia, or hypersensitivity to a non-painful stimulus, is a commonly reported migraine symptom. This study aimed to determine whether sleep deprivation results in orofacial allodynia and whether the arousal promoting neuropeptide orexin-A can recover this.

**Methods:** Mice were sleep deprived for 6 hours using the gentle handling method or allowed to sleep as usual (*n* = 12 per group). Periorbital mechanical withdrawal thresholds were tested pre and post deprivation using the von Frey assay. In a series of experiments, mice were administered either 100μg/kg orexin-A/vehicle or 20mg/kg caffeine/vehicle intraperitoneal and thresholds were tested 30 minutes post injection. To measure arousal, locomotor activity after administration of each drug was tested using infrared sensors. To establish if this effect is specific to sleep deprivation, orexin-A was administered after delivery of the clinical migraine trigger nitroglycerin.

**Results:** Caffeine significantly increased gross movements, and both caffeine and orexin-A significantly increased fine movements compared to vehicle. Sleep deprived mice had significantly lower sensory thresholds than non-sleep deprived mice. Injection of orexin-A, but not caffeine, significantly increased thresholds in sleep deprived mice, despite similar effects on arousal. Nitroglycerin significantly decreased thresholds, however orexin-A did not recover this.

**Conclusions:** These findings demonstrate that sleep deprivation leads to orofacial allodynia in mice. This can be reversed with administration of orexin-A, but not caffeine, indicating that this is unlikely to be driven by arousal. However, orexin-A was not analgesic when delivered after nitroglycerin-induced allodynia. Further research should explore the role of orexin-A in migraine.

## A31 P2Y14 receptor in trigeminal ganglion contributes to neuropathic pain in mice

### J. Lin^1,2^, X. Fang^1,3^, J. Shen^1^

#### ^1^Sichuan University, West China School of Stomatology, Chengdu, China; ^2^University of Barcelona, Department of Pathology and Experimental Therapeutics, Barcelona, Spain; ^3^Zhejiang University School of Medicine, Hospital of Stomatology, School of Stomatology, Hangzhou, China

##### **Correspondence:** J. Lin

**Question:** Trigeminal nerve injury usually induces trigeminal neuropathic pain but lacks effective treatments. Recent reports implied that P2Y14 receptor (P2Y_14_R) activation promoted orofacial inflammatory pain and migraine. However, the role and mechanism of P2Y14R in trigeminal neuropathic pain remain unknown.

**Methods:** Trigeminal neuropathic pain was induced by chronic constriction injury of the infraorbital nerve (CCI-ION). Orofacial mechanical threshold was measured by Von-Frey tests. The ATF3 (a mark of nerve injury) and P2Y14R were detected by immunofluorescence in the trigeminal ganglion (TG). The P2Y_14_R agonist (UDP-glucose) or antagonist (PPTN) was delivered by trigeminal ganglion injection in a stereotaxic apparatus. Furthermore, the expression of P2Y_14_R and its potential downstream cellular signalings were measured by RT-qPCR and/or western blot (WB) in the TG.

**Results:** Firstly, CCI-ION induced orofacial mechanical hypersensitivity. The increased ATF3 expression in the TG confirmed trigeminal nerve injury. P2Y_14_R was expressed in trigeminal ganglion neurons and satellite glial cells. RT-qPCR and WB showed that CCI-ION increased the expression of P2Y_14_R, interleukin-1β, interleukin-6, C-C chemokine CCL2, and tumor necrosis factor-α in TG. Secondly, PPTN alleviated CCI-ION-induced mechanical hypersensitivity and proinflammatory cytokines production. UDP-glucose evoked orofacial mechanical hypersensitivity and upregulated proinflammatory cytokines above. Thirdly, phosphorylated extracellular signal-regulated kinase 1/2 (ERK1/2) and p38 were increased in the TG after CCI-ION, which also were reduced by PPTN. Furthermore, the inhibitors of ERK1/2 (U0126) and p38 (SB203580) decreased these CCI-ION-induced proinflammatory cytokines.

**Conclusions:** The present study demonstrated that P2Y_14_R in the TG contributed to trigeminal neuropathic pain via ERK- and p38-dependent neuroinflammation. P2Y_14_R may be a potential drug target against trigeminal neuropathic pain.

**Fig. 1 (abstract A31).**
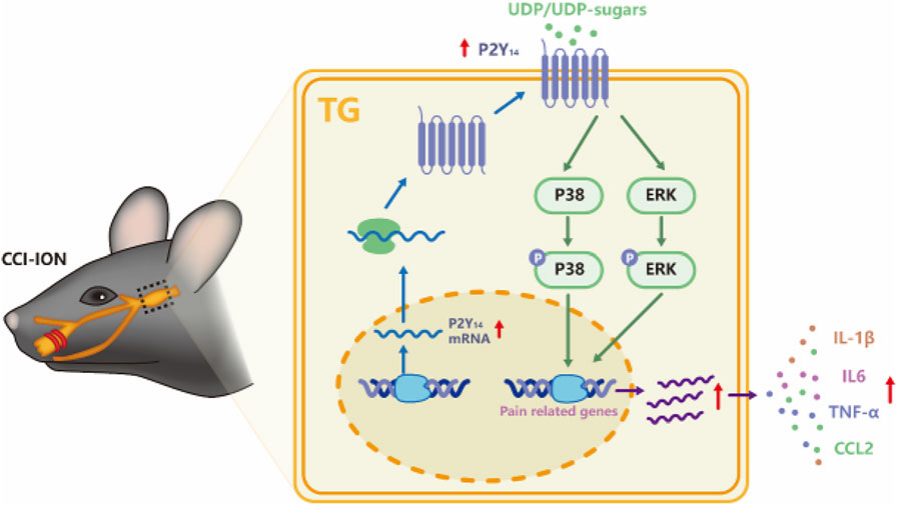
See text for description.

## A32 Kir4.1 in satellite glial cells contributes to trigeminal neuropathic pain

### J. Lin^1,2^, X. Fang^1,3^, J. Shen^1^

#### ^1^Sichuan University, West China School of Stomatology, Chengdu, China; ^2^University of Barcelona, Department of Pathology and Experimental Therapeutics, Barcelona, Spain; ^3^Zhejiang University School of Medicine, Hospital of Stomatology, School of Stomatology, Hangzhou, China

##### **Correspondence:** J. Lin

**Question:** Astrocyte Kir4.1 emerged as a novel therapeutic target for nervous system diseases, such as depression and pain. However, the role and mechanism of Kir4.1 in satellite glial cells (SGCs) in trigeminal neuropathic pain remain unknown. **Methods:** In vivo, chronic constriction injury of the infraorbital nerve (CCI-ION) and Von-Frey tests were used. The ATF3, GFAP, and Kir4.1 were detected by immunofluorescence in the trigeminal ganglion (TG). The AAV2/8 aimed at Kir4.1 was delivered to TG in WT and Kir4.1^f/f^ mice to knockdown or overexpress Kir4.1. In vitro, cultured SGCs were transfected with siRNA to knock down Kir4.1. The expression of Kir4.1 and its potential downstream cellular signalings were measured by RT-qPCR, western blot (WB), and/or ELISA. **Results:** Firstly, CCI-ION induced orofacial mechanical hypersensitivity. The increased ATF3 confirmed trigeminal nerve injury and increased GFAP indicated SGCs activation. Kir4.1 was expressed on

SGCs of TG. RT-qPCR and WB showed that CCI-ION decreased Kir4.1 expression but increased the expression of GFAP, brain-derived neurotrophic factor (BDNF), and glial cell-derived neurotrophic factor (GDNF) in TG. Secondly, knockdown of Kir4.1 in mice evoked orofacial mechanical hypersensitivity and upregulated GFAP, BDNF, and GDNF expression in TG. Overexpression of Kir4.1 alleviated CCI-ION-induced mechanical hypersensitivity and GFAP, BDNF, and GDNF production. In vitro, Kir4.1-siRNA treated SGCs increased GFAP, BDNF, and GDNF expression. Thirdly, phosphorylated extracellular signal-regulated kinase 1/2 (ERK1/2) was increased in TG after CCI-ION, knockdown of Kir4.1 mice, and Kir4.1-siRNA treated SGCs, which also were reduced by Kir4.1 rescue. Furthermore, the inhibitors of ERK1/2 (U0126) decreased these above upregulated BDNF and GDNF. **Conclusions:** This study indicated that Kir4.1 in SGCs contributed to trigeminal neuropathic pain by regulating BDNF and GDNF production via ERK signaling pathway and SGCs activation.

## A33 CGRP-induced relaxation remains unaffected in a porcine acute ischaemic stroke model

### D. Boucherie^1^, J. Bobi^2^, A. Taha^2^, H. M. M. van Beusekom^2^, A. H. J. Danser^1^, A. Maassen van den Brink^1^

#### ^1^Erasmus Medical Center, Internal Medicine, Division of Vascular Pharmacology, Rotterdam, Netherlands; ^2^Erasmus Medical Center, Cardiology, Division of Experimental Cardiology, Rotterdam, Netherlands

##### **Correspondence:** D. Boucherie

**Objective** Calcitonin gene-related peptide (CGRP) plays an important role in migraine attacks and is thought to be protective in ischaemic events. We investigated functional responses to exogenous CGRP of the middle cerebral artery (MCA) harvested from a gyrencephalic model of focal cerebral ischaemia.

**Methods** Female pigs (n=25; weight 51±3kg) underwent a craniotomy to occlude right-sided MCAs with aneurysm clips for 1 (n=5), 2 (n=5), or 4 (n=5) hours followed by 4-h recanalization, or for 8 h without recanalization (n=5). Two animals underwent a sham procedure. After euthanasia, the right-sided MCA was dissected for tension measurements. KCl-induced contraction (30–100 mM) was measured and endothelial function was assessed by bradykinin (BK)- or substance P (SP)-induced relaxation (10–100 nM) after precontraction with thromboxane A_2_ analogue U46619 (10–100 nM). Concentration-response curves were constructed to CGRP (0.1–100 nM) after precontraction with 30 mM KCl.

**Results** Relaxation to SP and BK was significantly reduced after 8 h occlusion (P<0.05), but not after occlusion followed by recanalization. Contraction to KCl was only reduced after 8 h occlusion (P<0.001). No differences were found in response to CGRP between groups (Figure).

**Conclusions** CGRP-induced relaxation remained unaffected after 8 h MCA occlusion, whereas SP- and BK-induced relaxation did not. These results suggest that CGRP acts via an endothelium-independent mechanism and that its potential protective effects after stroke depend on its local release rather than changes at the CGRP receptor level.

**Figure**: (A) No altered CGRP-induced relaxation after (recanalized) MCA occlusion. Reduced endothelial (B) and contractile (C) function after 8 h MCA occlusion. Results depicted as mean ± SEM.

**Fig. 1 (abstract A33).**
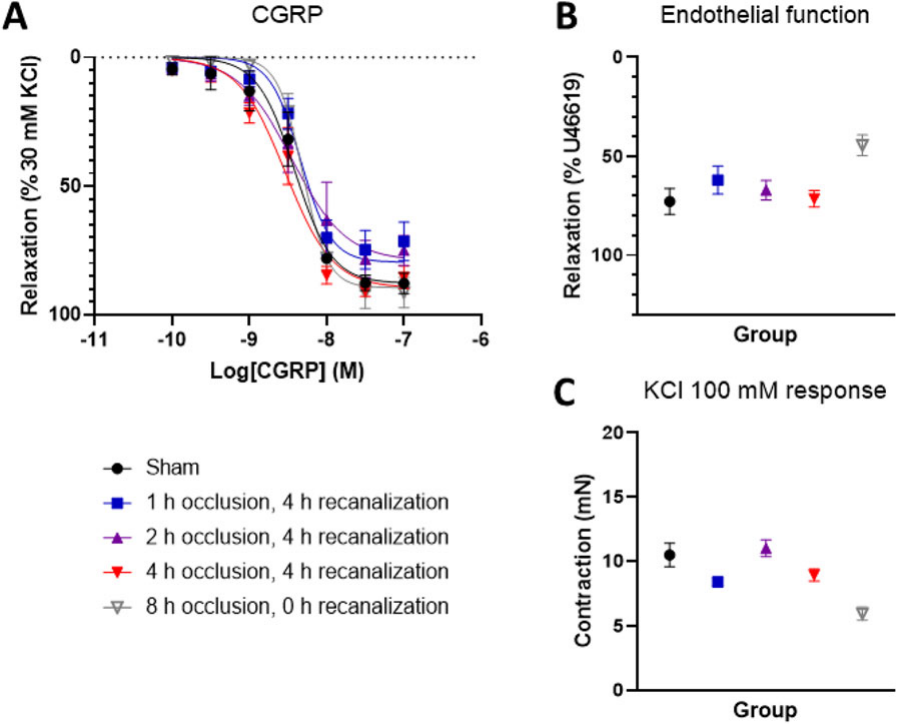
See text for description.

## A34 Involvement of the hypothalamus in migraine-related pathways

### Isabella Mai Christiansen, Anja Holm Nordvang, Lars Edvinsson, Kristian Agmund Haanes

#### Rigshospitalet, Department of Clinical experimental Research, Glostrup, Denmark

##### **Correspondence:** I. Christiansen

**Objective**

According to the current hypothesis of migraine pathophysiology, attacks are initiated in the hypothalamus, whereafter an activation cascade commences, which results in the activation of the trigeminal nucleus caudalis (TNC) and then trigeminal ganglion (TG). Calcitonin gene-related peptide (CGRP) release from the trigeminal system is thought to play a central role in migraine pathology, supported by the fact that currently available antimigraine drugs act by blocking CGRP signalling. The aim was to perform a preliminary examination of the hypothalamus in migraine-related pathways.

**Methods**

CGRP release from hypothalamus-containing acute brain slices was examined using an enzyme-linked immunosorbent assay. In addition, we investigated expression levels of *Calca* (encoding CGRP) and other genes related to dopaminergic pathways in the hypothalamus and the TNC.

**Results**

An extracellular concentration of 60 mM potassium significantly induced CGRP release, as compared to baseline (*p* = 0.01, *n* = 6), whereas application of 100 nM capsaicin did not (*p* = 0.16, *n* = 6). Using quantitative real-time polymerase chain reaction (qRT-PCR), we found that *Calc*a was expressed in the hypothalamus, but no difference was observed between male/female mice. *Drd2* (encoding the D2 receptor) was the most highly expressed dopamine receptor in both the male and female hypothalamus and TNC, indicating a dominantly inhibitory effect of dopamine in these regions. In addition, results from qRT-PCR demonstrated a tendency towards lower expression levels of *Drd2* in the TNC of female mice, as compared to males.

**Conclusion**

Central CGRP release can be detected from brain slices of mice, but the expression of *Calca* did not show any male/female difference. However, we found some sex differences in the dopaminergic pathways, suggesting that lower expression levels of *Drd2* in females as compared to males could contribute to increased excitability of the TNC.

## A35 A powerful dual MAGL/FAAH inhibitor AKU-005 against migraine pain of peripheral meningeal origin

### A. Della Pietra^1^, G. Krivoshein^2^, K. Ivanov^3^, R. Giniatullina^1^, V. Leinonen^4^, M. Lehtonen^5^, A. M. J. Maagdenberg^2^, J. Savinainen^3^, R. Giniatullin^1^

#### ^1^University of Eastern Finland, A.I. Virtanen Institute, Kuopio, Finland; ^2^Leiden University Medical Centre, Human Genetics and Neurology, Leiden, Netherlands; ^3^University of Eastern Finland, Biomedicine, Kuopio, Finland; ^4^Kuopio University Hospital, Kuopio, Finland; ^5^University of Eastern Finland, School of Pharmacy, Kuopio, Finland

##### **Correspondence:** A. Della Pietra

**Question**. Migraine is a neurological multifactorial disease whose worst symptom is pain. To deal with disturbing migraine pain, we propose the engaging of endocannabinoid system (ECS) via inhibition of enzymes monoacylglycerol lipase (MAGL) and fatty acid amide hydrolase (FAAH), which are degrading 2-arachidonoylglicerol (2-AG) and anandamide (AEA), respectively. To this end, we explored the analgesic effect of enhanced endocannabinoids (endoCBs) in peripheral meningeal tissues where pain signaling is of often originating in migraine, leading later to central sensitization.

**Methods**. To develop the functional platform for such purpose, we measured by activity-based protein profiling (ABPP) the activity of the main endoCBs-hydrolases, MAGL and FAAH and by LC-MS/MS the levels of 2-AG and AEA in rat meninges. We explored the analgesic effect of enhanced endoCBs with electrophysiological recordings from rat peripheral meningeal afferents.

**Results**. We found that in the meninges, 2-AG is the main endoCB, much exceeding the level of AEA. However, local depolarization increased the AEA ~2 folds without affecting 2-AG levels. The dual MAGL/FAAH inhibitor AKU-005 slightly increased basal nociceptive activity of trigeminal nerves. Instead, it significantly decreased, through CB1 receptors, meningeal nociceptive activity induced by depolarizing action of KCl, indicating analgesic effect.

**Conclusions**. These results suggest that 2-AG and AEA can differently contribute to counteract migraine pain by tonic or transient activity dependent release from meninges. These findings support the therapeutic perspective for engagement of both endoCBs analgesic molecules at early stages of attack. Thus, our novel dual ultrapotent MAGL/FAAH inhibitor AKU-005 appeared to be a promising tool in reducing migraine nociception originating in the meninges.

**Fig. 1 (abstract A35).**
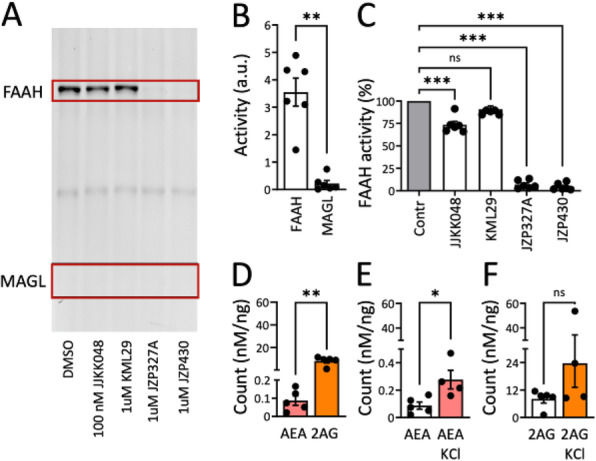
See text for description.

**Fig. 2 (abstract A35).**
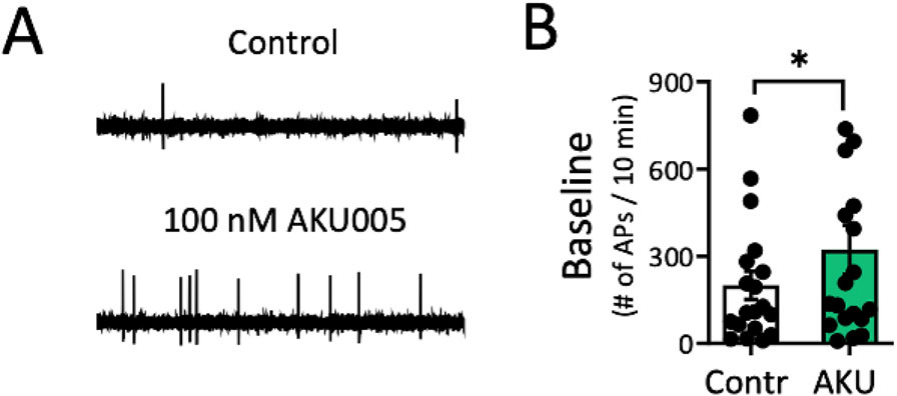
See text for description.

## A36 Whole exome sequencing of hemiplegic migraine patients shows an increased burden of missense variants in *CACNA1H* and *CACNA1I* genes

### O. Ibrahim^1^, A. Harder^2,3^, N. Maksemous^1^, L. Vijfhuizen^2^, H. Sutherland^1^, N. Pelzer^3^, I. de Boer^3^, G. Terwindt^3^, R. Lea^1^, A. van den Maagdenberg^2,3^, L. Griffiths^1^

#### ^1^Queensland University of Technology, Genomics Research Centre, Centre for Genomics and Personalised Health, School of Biomedical Sciences, Brisbane, Australia; ^2^Leiden University Medical Center, Department of Human Genetics, Leiden, Netherlands; ^3^Leiden University Medical Center, Neurology, Leiden, Netherlands

##### **Correspondence:** A. Harder

**Background:** Hemiplegic migraine (HM) is a migraine subtype with aura characterized by attacks associated with motor weakness. Given that causal mutations in the voltage-gated calcium channel a1A subunit gene *CACNA1A* have been found in a subset of HM patients, we investigated whether there is an increased burden in HM of missense variants in other *CACNA1x* genes.

**Methods:** Whole exome sequencing data of a clinically-referred Australian cohort of unrelated HM patients (*n* = 187), along with public data from Genome Aggregation Database (gnomAD v2.1.1) as controls, was used for a comprehensive analysis of missense variants in *CACNA1x* genes that included burden testing with the TRAPD package. Replication was performed in a Dutch clinical HM cohort (*n* = 32).

**Results:** Individual variant analysis of the Australian cohort revealed variants in multiple *CACNA1x* genes. Using TRAPD, we found a significant burden of missense variants in *CACNA1H* (*p* = 8.84 x 10-79) and *CACNA1I* (*p* = 3.00 x 10-169) in the Australian cohort that replicated in Dutch patients (*CACNA1H*, *p* = 0.012 and *CACNA1I*, *p* = 0.044), although *CACNA1I* did not remain significant after correction for multiple testing. The burden effect was slightly higher for *CACNA1I* (OR = 1.43, 95% CI:1.28 - 1.58) than for *CACNA1H* (OR = 1.83, 95% CI:1.54 - 2.12).

**Conclusion:** Our data suggest that HM, in the absence of a single causal mutation in *CACNA1A*, *ATP1A2*, or *SCN1A*, is a complex trait, in which, at least to certain extent, increased burden of missense variants in *CACNA1H* and *CACNA1I* increases the risk of disease.

## A37 Arterial responses to infusion of glucagon-like peptide-1 in humans: A randomized trial study

### R. Christensen^1^, H. Ghanizada^1^, M. Al-Mahdi Al-Karagholi^1^, F. Azzahra Elbahi^1^, H. Coskun^1^, M. Ashina^1,2^

#### ^1^Danish Headache Center, Neurology, Glostrup, Denmark; ^2^Danish Headache Center, Copenhagen, Denmark

##### **Correspondence:** R. Christensen

**Objective:** Glucagon-like-peptide-1 (GLP-1) is an incretin hormone implicated in several metabolic and neurological disorders. GLP-1 induces vasodilation and increases blood flow in the peripheral circulation. Whether GLP-1 alters cerebral hemodynamics in humans is yet to be elucidated.

**Methods:** In a crossover, double-blind, placebo-controlled, and randomized design, 21 healthy volunteers were assigned to receive intravenous GLP-1 infusion (2.5 pmol/kg/min) or placebo over 20 min on two different days separated by at least one week. We used a noninvasive, well-validated transcranial doppler (TCD) and ultrasound dermascan to reveal the effect of GLP-1 on intra- and extracerebral arteries. The mean blood flow velocity in the middle cerebral artery (VMCA), the diameter of the superficial temporal artery (STA) and radial artery (RA), and facial skin blood flow were measured. In addition, we documented headache and its associated symptoms during and after infusion.

**Results:** Twenty participants were included in the final analysis. We found no difference in the VMCA (P = 0.227), diameter of the STA (P = 0.096), the RA (P = 0.221), or facial blood flow (P = 0.814) after GLP-1 compared to placebo. There were no differences in HR, SAT, EtCO2, or RF (P > 0.05) on the GLP-1 day compared to the placebo day. We found no differences in the incidence of headache after GLP-1 (n = 10) compared to placebo (n = 7) (P = 0.250).

**Conclusion:** GLP-1 infusion did not affect cerebral hemodynamics or induce headache in humans. Further preclinical studies with validated methods are required to determine if intra – and extracerebral vasculature express GLP-1Rs in humans.

**Fig. 1 (abstract A37).**
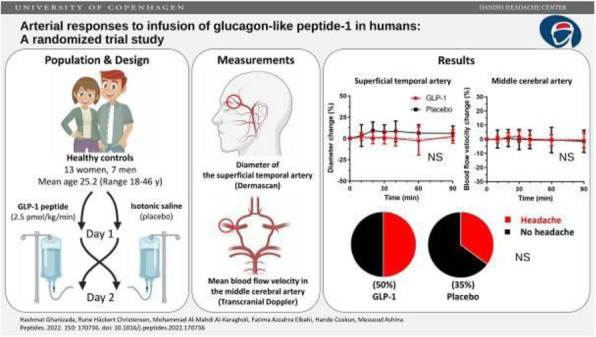
See text for description.

## A38 Prevalence of substance use in a Dutch migraine population

### T. van den Hoek, I. de Boer, I. Verhagen, G. Terwindt

#### Leiden University Medical Center, Neurology, Leiden, Netherlands

##### **Correspondence:** T. van den Hoek

*Objective:* Tobacco, alcohol, and illicit substance use result in substantial morbidity and mortality. Moreover, there is controversy concerning their ability to increase susceptibility to migraine attacks. The current study sought to examine the prevalence of substance use in a large migraine cohort. Prevalence was compared to the general population.

*Methods:* Data on substance use were collected by survey in a large migraine cohort^1^ and from the annual health survey for substances in the general Dutch population (Statistics Netherlands 2016/2017). Associations for substance consumption were standardized for sex, age and educational level and analyzed using chi-square.

*Results:* From the Leiden Headache Center, a standardization a total of 4993 patients with migraine were included. Differences were found between migraine and the general population for illicit drug use (OR 0.53, 95%CI 0.45-0.63, p<0.01), current smoking

(OR 0.52, 95%CI 0.47-0.57, p<0.01), life-time smoking (OR 0.52, 95%CI 0.48-0.55, p<0.01) and current alcohol consumption (OR 0.44, 95%CI 0.40 – 0.47, p<0.01). Prevalences for the use of illicit drugs, current smoking, life-time smoking and current alcohol consumption are shown in **figure 1**.

*Conclusion:* Individuals with migraine are less likely to smoke, drink alcohol or use illicit drugs compared to the general population. Migraine patients might avoid alcohol due to presumed trigger effects.^2^ Differences in smoking prevalence might be due to an increased awareness of an elevated cardiovascular risk among migraine patients.

**References**

^1^ Oosterhout et al Cephalalgia. 2011 Oct;31(13):1359-67

^2^ Onderwater et al Eur J Neurol. 2019;26:588-95

**Fig. 1 (abstract A38).**
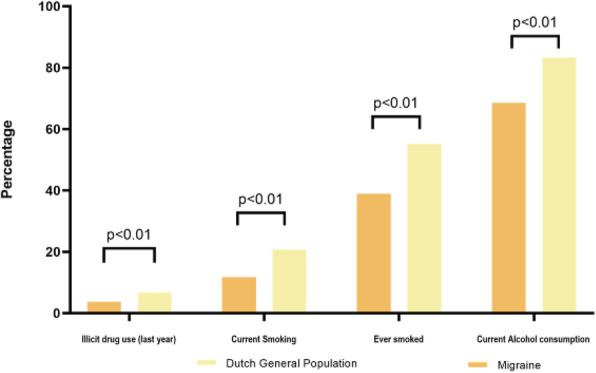
Substance use for Dutch General population and migraine patients after standardization adjusting for age, sex and education.

## A39 A novel approach to identify and classify visual migraine aura: a European multicenter study on 257 patients

### M. Viana^1^, A. Hougaard^2^, I. Grøntveit Winnberg^3^, A. Ambrosini^4^, C. Gobbi^1,5^, A. Perrotta^4^, T. Pho Do^2^, E. Tronvik^3^, C. Zecca^1,5^

#### ^1^Ospedale Regionale di Lugano, Neurology, Lugano, Switzerland; ^2^University of Copenhagen, Rigshospitalet, Neurology, Copenhagen, Denmark; ^3^Norwegian University of Science and Technology (NTNU), Neurology, Trondheim, Norway; ^4^Neuromed, Headache Clinic, Pozzilli, Italy; ^5^Università della Svizzera Italiana, Faculty of Biomedical Sciences, Lugano, Switzerland

##### **Correspondence:** M. Viana

Question: is it feasible to create standardized images representing visual disturbances (VD) of migraine aura (MA)?

Objectives: To test the representativeness of a standardized MA iconography (SMAI) constituted of 25 elementary visual disturbances (EVDs) in a large population of patients suffering from visual MA.

Methods: We prepared descriptions (EVD-texts) and graphical representations on a common background image (EVD-images) of 25 EVDs, that were used to create a web-based survey (WBS). Patients>18years-old, suffering from visual MA and seen consecutively at one of 4 European Headache Centres (Lugano, Switzerland; Copenhagen, Denmark; Trondheim, Norway; Rome, Italy], were invited to participate. They fulfilled the WBS (via computer or mobile devices) where they were asked to choose which among EVDs they had experienced during their MAs and/or to provide a text description of any other EVDs experienced but not included in the WBS. Primary endpoint was the proportion of EVDs experienced by participants which were recognized in the set of 25 EVD-images. The study was approved by competent Ethics committees.

Results: 257 patients with visual MA completed the study: 209 were female (81.3), mean age was 36.7. Patients experienced overall 1741 different VD. Of those, 73% could be recognized as variable combinations of the 25 EVDs in the SMAI. Description of EVSs experienced by pts in their lives are reported in the table.

Conclusion: The majority of VD experienced by patients during their MA were recognized as composed of one or more EVDs in our SMAI. When validated, this this tool will allow a precise VA phenotyping with useful clinical and research applications.

**Table 1 (abstract A39).**
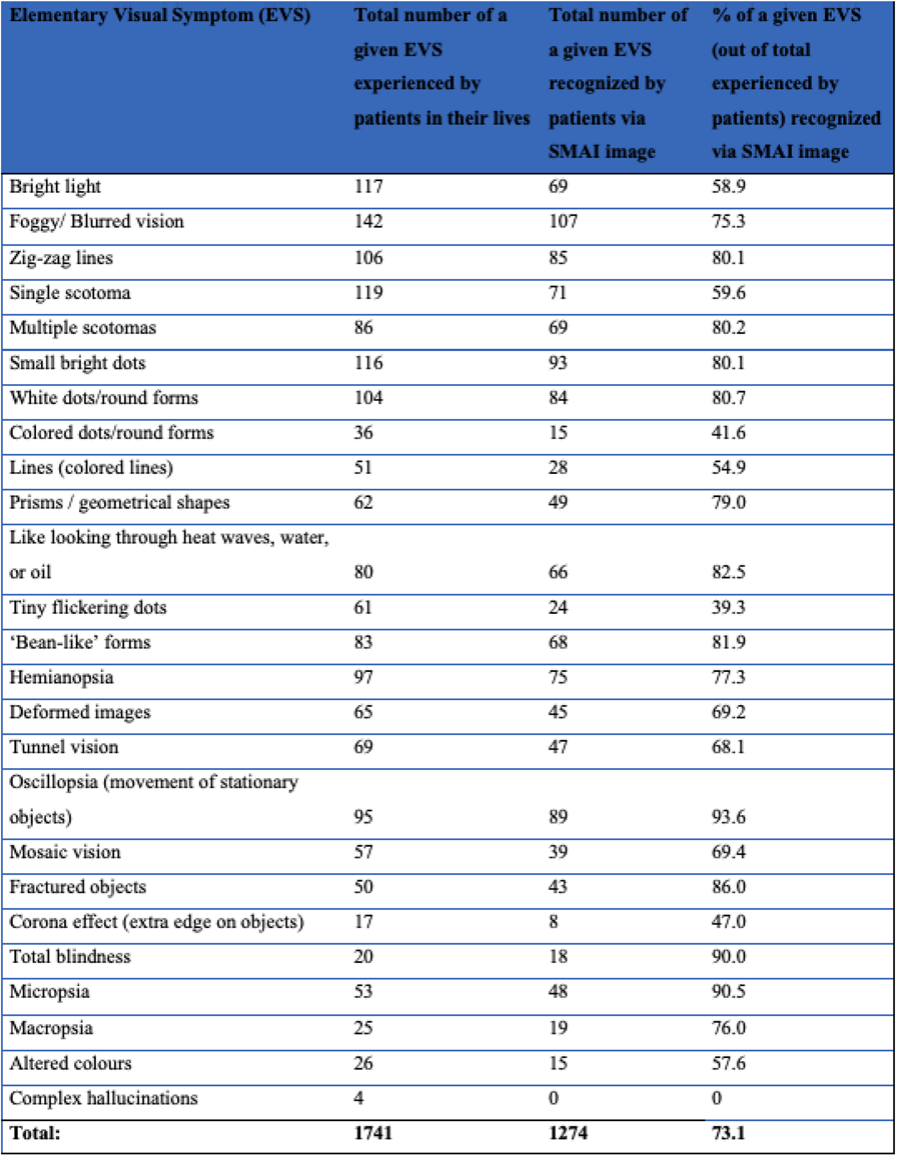
Occurence of each Elementary Visual Symptom (EVS) as experienced by patients in their lives and as recognized by patients via images included in the Standardized Migraine Aura Iconography (SMAI)

## A40 How frequent is visual aura without headache caused by an underlying cause (structural or embolic)?

### H. Koppen^1^, R. van der Zwet^2^, D. Tavy^1^

#### ^1^HagaZiekenhuis, Neurology, The Hague, Netherlands; ^2^Leiden University Medical Center, Neurology, Leiden, Netherlands

##### **Correspondence:** H. Koppen

Question: How frequent is visual aura without headache (VAWOH) caused by an underlying cause?

Methods: Since 2014 subjects with VAWOH were registered in the HagaTeachingHospital registry which now holds 156 consecutive patients seen at the outpatient headache clinic during the timespan of eight years. Subjects underwent standard brain imaging (mainly MRI and in some cases CT) and the first 100 received Transcranial Doppler with emboli detection in medial cerebral artery (TCD-ED). All investigations were performed interictally.

Results: Mean age of 156 subjects in the VAWOH-registry was 59 (range 20-91 years), 107 (70%) were female. Brain imaging showed related lesions in 8 (5%) of 150 subjects. Three were scored as causal: one had an occipital dysembryoplastic neuroepithelial tumor with monthly occuring side-locked aura symptoms for more than 10 years. One subject had an occipital located metastasis of breast carcinoma. One subject had an arterio-venous malformation (AVM) located in the occipital cortex. In four subjects the diagnosis of acute migrainous infarction was made, one of these was caused by de novo trombotic thrombocytopenic purpura. In one subject an older occipital infarction was found. In these 5 ischemic subjects no active emboli were found.

TCD-ED was performed in the first 100 VAWOH subjects. This was technically not possible in 16/100 (16%) due to thick skullbone. Four subjects (5%) showed one or more embolic signals suggesting microemboli during the 30-minute bilateral ACM registration. Two of these emboli positive subjects had recently underwent mitral valve operation or repair respectively. One patient recently underwent ablation for atrial fibrillation with atrialseptal wall puncture.

Conclusions: In 7 (4.6%) of 150 evaluated subjects with VAWOH an underlying cause (structural or embolic) was found.

## A41 Do novel European Headache Federation criteria identify differences in migraine burden? Baseline data of an international real-life study on resistant and refractory migraine (REFINE)

### V. Caponnetto^1^, R. Ornello^1^, C. Rosignoli^1^, N. De Santis^1^, D. Bayar^2^, M. Braschinsky^3^, M. Carnovali^4^, M. Gentile^5^, R. Gil-Gouveia^6^, G. Iaccarino^7^, A. R. Leheste^3^, P. Martelletti^8^, C. Mazzanti^8^, A. Muñoz-Vendrell^9^, R. Oliveira^10^, A. Ozge^2^, I. Pavão Martins^11^, P. Pozo-Rosich^9^, M. P. Prudenzano^5^, K. Ryliskiene^12^, M. Sanchez del Rio^13^, J. Vainauskienė^14^, F. Vernieri^7^, Z. Katsarava^4^, S. Sacco^1^

#### ^1^University of L'Aquila, Department of Applied Clinical Sciences and Biotechnology, L'Aquila, Italy; ^2^Mersin University Faculty of Medicine, Neurology, Mersin, Turkey; ^3^Tartu UnIversity Hospital, Neurology, Tartu, Estonia; ^4^Evangelical Hospital Unna, Unna, Germany; ^5^Centro Cefalee, Clinica Neurologica "L. Amaducci", Azienda Ospedaliero-Universitaria Policlinico Consorziale di Bari, Bari, Italy; ^6^Hospital da Luz and Universidade Católica Portuguesa, Center for Interdisciplinary Research in Health, Lisbon, Portugal; ^7^Cefalee e Neurosonologia, Policlinico Universitario Campus Bio-medico, Rome, Italy; ^8^Sapienza University of Rome, Department of Clinical and Molecular Medicine, Rome, Italy; ^9^Vall d’Hebron University Hospital and Autonomous University of Barcelona, Barcelona, Spain; ^10^Hospital da Luz, Lisbon, Portugal; ^11^Faculdade de Medicine and Hospital Universitário de Santa Maria, Centro Hospitalar - Hospital Cuf Tejo, Lisbon, Portugal; ^12^Vilnius University, Centre of Neurology, Vilnius, Lithuania; ^13^Clínica Universidad de Navarra, Madrid, Spain; ^14^Vilnius University, Vilnius, Lithuania

##### **Correspondence:** V. Caponnetto

**Question.** We evaluated if EHF criteria for resistant (RES) and refractory (REF) migraine identify patients with more severe migraine burden.

**Methods.** We performed an observational, multi center, international study to compare baseline characteristics, comorbidities, and PROMs of non-resistant and non-refractory (NRNR) migraine, RES and REF individuals in the REFINE study.

**Results.** We included 175 individuals with NRNR migraine, 133 (39.7%) with RES and 27 (8.0%) with REF. Individuals with RES and REF migraine as compared to those with NRNR reported higher monthly migraine days (median=8, IQR=5-14 vs. median=13, IQR=10-17 and median=15, IQR=10-20; p≤0.001), months of chronification (median=24, IQR=12-72 vs. median=40, IQR=12-108 and median=60, IQR=18-96; p=0.044), monthly days of symptomatic drugs assumption (median=8, IQR=5-15 vs. median=12, IQR=9-20 and median=15, IQR=10-20; p≤0.001), medication overuse (19.4% vs. 45.9% and 40.7%; p≤0.001). They also had more comorbidities such as depression (18.3% vs. 31.1% and 44.4%; p=0.002) and anxiety (13.7% vs. 21.1% and 37%; p=0.009). In these groups, PROMs also revealed a higher presence of anxiety (p≤0.001) and depression (p≤0.001) symptoms and poorer sleep quality (p=0.006). Regarding specific perceptions about migraine, RES and REF individuals reported higher impact of migraine on daily life (p≤0.001) and work, household work, and social life (p≤0.001), along with a lower perception of the effectiveness of their ongoing treatment for migraine (p≤0.001), when compared to NRNR subjects (Table 1).

**Conclusion.** RES and REF migraine is associated with relevant migraine burden considering migraine features, comorbidities and scores at several scales; the severe burdensome condition of RES and REF is confirmed by the median number of monthly migraine days and PROMs.

**Table 1 (abstract A41).**
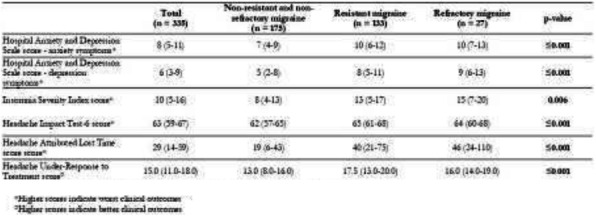
Patient reported outcome measures (PROMs) scores reported as median (IQR)

## A42 OnabotulinumtoxinA in elderly patients with chronic migraine: insights from a Real-Life European Multicenter Study

### C. Altamura^1^, R. Ornello^2^, F. Ahmed^3^, A. Negro^4^, A. Miscio^5^, A. Santoro^5^, A. Apulente^6^, A. Russo^7^, M. Silvestro^7^, S. Cevoli^8^, N. Brunelli^1^, C. Baraldi^9^, S. Guerzoni^9^, A. P. Andreou^10^, G. Lambru^10^, I. Frattale^2^, K. Kamm^11^, R. Ruscheweyh^11^, M. Russo^12^, P. Torelli^13^, E. Filatova^14^, N. Latysheva^14^, A. Gryglas-Dworak^15^, M. Straburzynski^16^, C. Butera^17^, B. Colombo^17^, M. Filippi^17^, P. Pozo-Rosich^6^, P. Martelletti^4^, S. Sacco^2^, F. Vernieri^1^

#### ^1^Fondazione Policlinico Universitario Campus Bio-Medico di Roma, Headache and Neurosonology Unit, Rome, Italy; ^2^University of L’Aquila, L'Aquila, Italy; ^3^Hull UniversityTeaching Hospitals, Hull, United Kingdom; ^4^Sapienza University, Rome, Italy; ^5^Fondazione IRCCS “Casa Sollievo della Sofferenza”, San Giovanni Rotondo (FG), Italy; ^6^Valld’Hebron University, Barcelona, Spain; ^7^University of Campania “Luigi Vanvitelli”, Naples, Italy; ^8^IRCCS Istituto delle scienze Neurologiche di Bologna, Bologna, Italy; ^9^University of Modena and Reggio Emilia, Modena, Italy; ^10^Guy's and St Thomas' NHS Foundation Trust, London, United Kingdom; ^11^Ludwig Maximilians University, Munich, Germany; ^12^Azienda USL-IRCCS di Reggio Emilia, Reggio Emilia, Italy; ^13^University of Parma, Parma, Italy; ^14^Sechenov University, Moscow, Russian Federation; ^15^Headache Center Wroclaw, Wrocław, Poland; ^16^Straburzynski Headache Clinic, Warsaw, Poland; ^17^IRCCS San Raffaele Scientific Institute, Milan, Italy

##### **Correspondence:** R. Ornello

QUESTION The prevalence of migraine decreases after the fifth decade of life. However, when persisting in old age, migraine may be highly disabling, and some patients can still suffer from chronic migraine (CM). This study aimed to investigate the outcome of OBT-A as preventative therapy in elderly CM patients.

METHODS: This is a post-hoc analysis of real-life prospectively collected data at 16 European headache centers on patients treated with OBT-A for CM over the first three treatment cycles. Patients aged ≥65 years were defined as OLD, and those <65-year-old, nonOLD. The primary endpoint was the changes in monthly headache days (MHDs) from baseline to each treatment cycle (i.e., Cy1-3) in OLD compared with nonOLD participants. The secondary endpoints were the frequency of responder rate (RR) ≥50%, conversion to episodic migraine (EM) and the changes in days with acute medication use (DAMs) from baseline to Cy-3.

RESULTS In a cohort of 2831 CM patients, 235 were OLD (8.3%, range 65-91 yrs, 69.6 SD 4.7; 73.2% females) with a migraine history of 47.2 yrs (SD 13.5), of which 15.2 (SD 13.9) with a chronic frequency. After Cy-3, 32.3% of OLD participants discontinued the treatment. We observed a progressive decrease in MHDs from baseline (24.8 SD 6.2) to Cy-1 (17.5 SD 9.1, p<.000001), from Cy-1 to Cy-2 (14.8 SD 9.2, p<.0001), and from Cy-2 to Cy-3 (11.9 SD 7.9, p =.001) and in DAMs from baseline (19.2 SD 9.8) to Cy-1 (11.9 SD 8.8, p<.00001), from Cy-1 to Cy-2 (10.9 SD 8.6, p=.012), and from Cy-2 to Cy-3 (9.6 SD 7.4, p =.049). The percentage of OLD patients with RR ≥50% increased from 30.7% (Cy-1) to 34.5% (Cy-2), to 38.7% (Cy-3). The changes in MHDs and the frequency of RR ≥50% or conversion to EM did not differ in OLD compared with nonOLD patients along with the three cycles.

CONCLUSION In a population of elderly CM patients, OnabotulinumtoxinA provided a significant benefit in the first three cycles of treatment, as good as in non-old patients.

**Fig. 1 (abstract A42).**
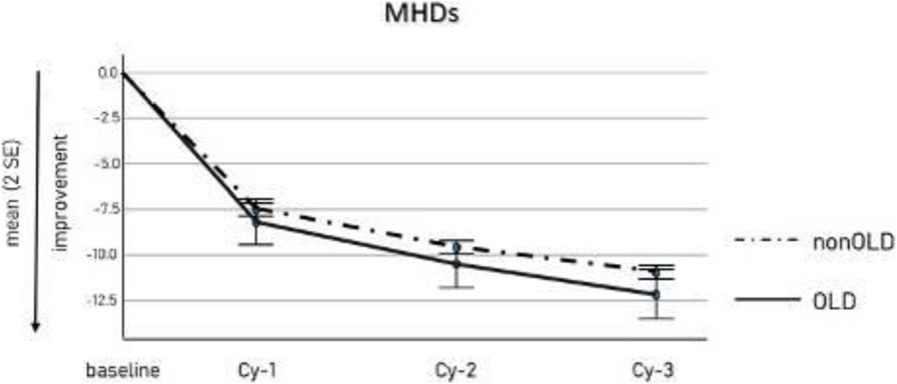
See text for description.

**Fig. 2 (abstract A42).**
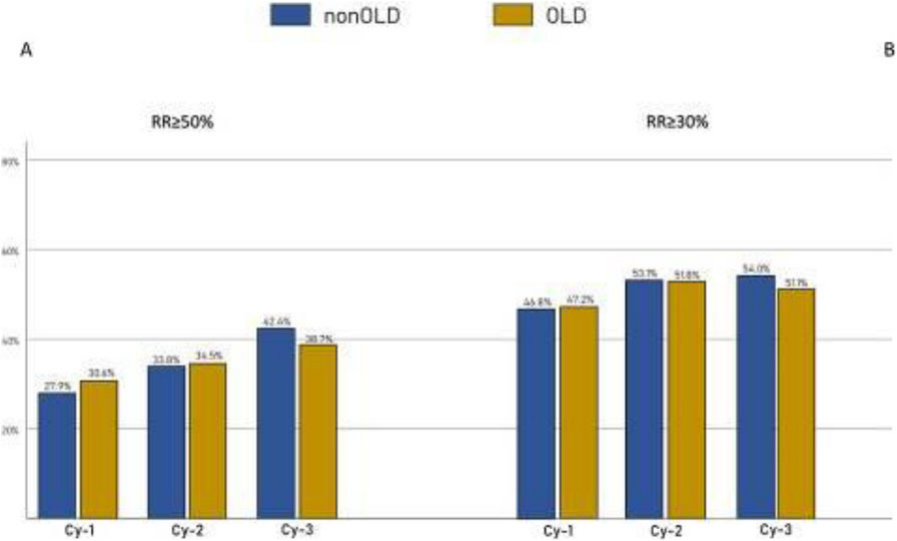
See text for description.

## A43 Pre-attack and pre-episode symptoms in cluster headache: a multicenter cross-sectional study of 327 Chinese patients

### K. Li^1^, S. Sun^1^, Z. Xue^2^, S. Chen^3^, D. Hu^4^, X. Gao^5^, Y. Wang^6^, D. Wang^7^, J. Chen^8^, L. Li^9^, J. Liu^1,10^, M. Zhang^1^, Z. Jia^1^, X. Han^1^, H. Liu^1^, M. He^1^, W. Zhao^1^, Z. Gong^1^, S. Zhang^1^, X. Lin^1^, Y. Liu^1^, S. Wang^1^, S. Yu^1^, Z. Dong^1^

#### ^1^the First Medical Center, Chinese PLA General Hospital, Beijing, China; ^2^Suzhou Blue Cross Brain Hospital, Neurology, Jiangsu, China; ^3^Changsha Central Hospital affiliated to University of South China, Neurology, Changsha, China; ^4^The Second Affiliated Hospital of Shandong First Medical University, Neurology, Shandong, China; ^5^Affiliated Yantai Yuhuangding Hospital of Qingdao University, Neurology, Yantai, China; ^6^Changchun Hospital of traditional Chinese medicine, Neurology, Changchun, China; ^7^Hospital, Neurology, Shenyang, China; ^8^Hospital, Neurology, Lishui, China; ^9^hospital, Neurology, Jincheng, China; ^10^hospital, Beijing, China

##### **Correspondence:** K. Li

**Objective:** There have been a few studies regarding the pre-attack symptoms (PAS) and pre-episode symptoms (PES) of cluster headache (CH), but none have been conducted in the Chinese population. The purpose of this study was to identify the prevalence and features of PAS and PES in Chinese patients, as well as to investigate their relationships with pertinent factors.

**Methods:** The study included patients who visited a tertiary headache center and nine other headache clinics between January 2019 and September 2021. A questionnaire was used to collect general data and information about pre-attack and pre-episode symptoms.

**Results:** Among the 327 patients who met the CH criteria (International Classification of Headache Disorders, 3rd edition), 269 (82.3%) patients experienced at least one PAS. The most common PAS were head and facial discomfort (74.4%). Multivariable logistic regression analysis depicted that the number of triggers (OR = 1.798, *p* = 0.001), and smoking history (OR = 2.067, *p* = 0.026) were correlated with increased odds of PAS. In total, 68 (20.8%) patients had PES. The most common symptoms were head and facial discomfort (23, 33.8%). Multivariable logistic regression analysis showed that the number of triggers were associated with increased odds of PES (OR = 1.372, *p* = 0.005).

**Conclusions:** PAS are quite common in CH patients, demonstrating that CH attacks are not comprised of a pain phase alone; investigations of PAS and PES could help researchers better understand the pathophysiology of CH.

**Fig. 1 (abstract A43).**
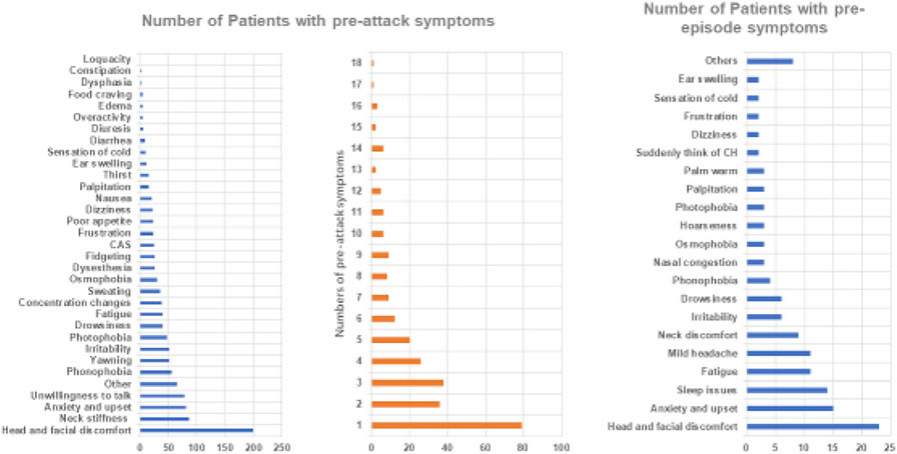
See text for description.

**Fig. 2 (abstract A43).**
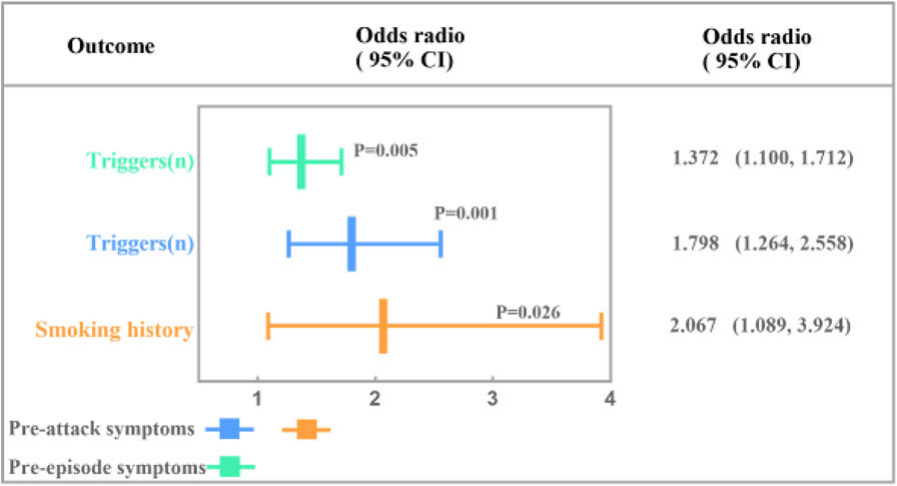
See text for description.

## A44 Persistent headache after first-ever ischemic stroke: clinical characteristics and factors associated with their development

### E. R. Lebedeva, A. V. Ushenin, N. M. Gurary, D. V. Gilev, N. V. Kislyak, J. Olesen

#### The Ural State Medical University, International Headache Center Europe_Asia, Neurology, Yekaterinburg, Russian Federation

##### **Correspondence:** E. R. Lebedeva

**Background** It is poorly described how often headache attributed to stroke continues for more than 3 months, i.e. fulfills the criteria for persistent headache attributed to ischemic stroke. Our aims were: 1) to determine the incidence of these headaches; 2) to describe characteristics and acute treatment; 3) to evaluate risk factors.

**Methods** The study population consisted of 550 patients (mean age 63.1, 54% males) with first-ever ischemic stroke, among them 529 patients were followed up at least three months after stroke. Standardized semi-structured interview forms were used to evaluate these headaches during professional face-to-face interviews at stroke onset and telephone interviews at 3 months.

**Results** At three months, 61 patients (30 women and 31 men, the mean age 60.0) of 529 (11.5%) follow-up patients had a headache after stroke: 34 had a new type of headache, 21 had a headache with altered characteristics and 6 patients had a headache without any changes. Therefore 55 (10.4%) patients had a persistent headache attributed to ischemic stroke. Their clinical features included: less severity of accompanying symptoms, slow decreasing frequency and development of medication overuse headache in one-third of the patients. The following factors were associated with these headaches: lack of sleep (29.1%, p=0.009; ОR 2.3; 95% CI 1.2-4.3), infarct in cerebellum (18.2%, p=0.003; OR 3.0; 95% CI 1.4-6.6), stroke of undetermined etiology (50.9%, p=0.003; ОR 2.3; 95% CI 1.3-4.1), less than 8 points by NIHSS score (90.9%, p=0.007; OR 3.4; 95% CI 1.4-8.6) and low prevalence of large-artery atherosclerosis (12.7%, p=0.006; OR 0.3; 95% CI 0.2-0.80).

**Conclusion** Persistent headache attributed to ischemic stroke is not rare and frequently leads to medication overuse. The problem is often neglected because of other serious consequences of stroke but it actually has considerable impact on quality of life. It should be a focus of interest in the follow-up of stroke patients.

## P1 The TRPA1 pain pathway is a target for multiple environmental pollutants: Possible relevance for migraine

### R. H. Rasmussen^1^, S. L. Christensen^1^, K. Callø^2^, A. Rehfeld^3^, T. E. Taylor-Clark^4^, K. A. Haanes^5^, O. Taboureau^6^, K. Audouze^7^, D. Klærke^2^, J. Olesen^1^, D. M. Kristensen^1^

#### ^1^Dansk Hovedpine Center, Rigshospitalet Glostrup, Glostrup, Denmark; ^2^University of Copenhagen, Rigshospitalet, Department of Veterinary and Animal Sciences, Frederiksberg, Denmark; ^3^University of Copenhagen, Rigshospitalet, Department of Growth and Reproduction, Copenhagen, Denmark; ^4^University of South Florida, Department of Molecular Pharmacology and Physiology, Tampa, FL, United States; ^5^Copenhagen University Hospital - Rigshospitalet, 5Department of Clinical Experimental Research, Glostrup, Denmark; ^6^Paris University, Unité de Biologie Fonctionnelle, Paris, France; ^7^Paris University, INSERM U1124, Paris, France

##### **Correspondence:** R. H. Rasmussen

The prevalence of the disabling pain disorder migraine is rising. Here, we test the hypothesis that ubiquitous environmental pollutants evoke the release of migraine-inducing neuropeptide calcitonin gene-related peptide (CGRP), via the activation of transient receptor potential (TRP) channels, thereby increasing pain.

To understand if environmental pollutants target migraine-associated TRP channels ankyrin 1 (TRPA1) and vanilloid 1 (TRPV1), we used a calcium imaging-based screen of pollutants known to be abundant in industrialized regions. Based on this screen, patch clamping and *in silico* docking, we selected a pesticide (pentachlorophenol; PCP) to perform proof-of-concept experiments. We tested *ex vivo* release of CGRP and *ex vivo* vasodilatory responses of isolated cerebral arteries to PCP. Finally, we tested *in vivo* induction of cutaneous hypersensitivity in wild type and *Trpa1* deficient mice.

16 of 53 screened environmental pollutants activated TRPA1, while none of the investigated compounds activated TRPV1. Focusing on PCP, *in silico* molecular modelling suggested that PCP is stabilized in a known lipid binding pocket of TRPA1. *In vitro*, *ex vivo* and *in vivo* experiments showed that PCP induced calcium influx in neurons, TRPA1-dependent CGRP release from the brainstem, dilation of cerebral arteries, and TRPA1-dependent increased pain response in mice.

These findings establish that abundant pollutants from the environment interact with the TRPA1-CGRP migraine pain pathway. Therefore, exposure to ubiquitous pollutants might be a contributing factor to the increased migraine prevalence.

## P2 Functional responses in a patient-specific iPSC-derived vascular model: a novel approach to study migraine

### T. de Vries^1^, D. Schutter^1^, A. van den Bogaerdt^2^, A. H. J. Danser^1^, A. Maassen van den Brink^1^

#### ^1^Erasmus Medical Center, Internal Medicine, division of Pharmacology and Vascular Medicine, Rotterdam, Netherlands; ^2^ETB-BISLIFE, Heart Valve Department, Beverwijk, Netherlands

##### **Correspondence:** T. de Vries

**Objective** Blood vessels from migraine patients are useful for studying migraine pathophysiology and drug development, but are difficult to obtain. Here, we develop a 3D vessel-on-chip model incorporating induced pluripotent stem cell (iPSC)-derived vascular smooth muscle cells (VSMCs) and endothelial cells, allowing to study patient-specific blood vessels. The vascular responses of the cultured blood vessel model are compared to native human blood vessels to validate the model.

**Methods** In iPSC-derived VSMCs, grown in 2D or in a 3D conformation in a vessel-on-chip model, cAMP responses are measured using the cADDis live cell cAMP assay after stimulation with CGRP and in the presence of phosphodiesterase 3 (PDE3) inhibitors milrinone or cilostazol or the CGRP receptor antagonists rimegepant or olcegepant. Responses to CGRP in the presence of these gepants in iPSC-derived VSMCs were compared to responses in human coronary arteries from heart valve donors (8 F and 7 M, age 48±3 years), as measured in a Mulvany myograph system. Intracellular calcium responses in VSMCs were measured using the calcium dye Cal-520 after stimulation with 10 nM endothelin-1 (ET-1).

**Results** PDE3 inhibitors milrinone and cilostazol significantly augment the cAMP response to CGRP in iPSC-derived VSMCs (p=0.034 and p=0.002, resp.), while rimegepant inhibits the cAMP response to CGRP, similarly as observed in human isolated arteries. In the 3D cultured blood vessels, olcegepant potently blocked the response to CGRP, while ET-1 potently increased the intracellular calcium concentrations. Comparable results were obtained in human isolated arteries (Labruijere et al. 2013).

**Conclusion** Functional measurements can be performed in human IPSC-derived VSMCs, in both 2D and 3D conformation, with comparable results in cultured blood vessels and isolated human arteries. These patient-specific vessel-on-chip models could be used in the future to study migraine pathophysiology and improve drug development.

**Fig. 1 (abstract P2).**
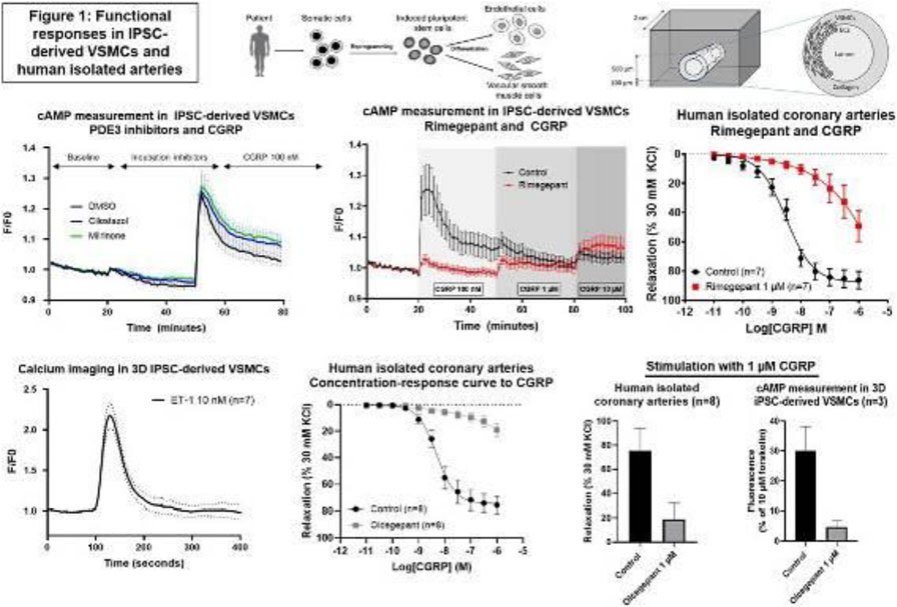
Functional responses in IPSC-derived VSMCs and human isolated arteries

## P3 Relevance of mouse vasculature in investigating side-effects of anti-migraine medications

### S. Kazantzi^1,2^, L. Edvinsson^1^, K. A. Haanes^1,2^

#### ^1^University of Copenhagen, Rigshospitalet, Department of Clinical Experimental Science, Copenhagen, Denmark; ^2^University of Copenhagen, Rigshospitalet, Biology, Copenhagen, Denmark

##### **Correspondence:** S. Kazantzi

Objective

The gold standard treatment in migraine, the triptans, are usually not prescribed to patients with cardiovascular risk factors. Further it has been debated whether, the Gepants and anti-CGRP/CGRP receptor antibodies (collectible named anti-CGRP treatments) poses an additional risk in patients in relation to cardiovascular disease. There are some studies aiming to address this experimentally, particular using mice models. We therefore set out to investigate if the mouse is a good model for cardiovascular effects of anti-migraine treatments.

Methods

∼2mm long isolated mouse coronary (40μm wire) and basilar segments (25 μm wire) were mounted on a Mulvany–Halpern myograph. The smooth muscle cell contractile function was confirmed by challenging the segments two times with K+. We performed a concentration response curve to 5-HT with a maximal concentration of 10 μM. Following an addition of U46619 (~ 0.1 μM), the segments where challenged with CGRP (1 μM) followed by PACAP (0.1 μM).

Results

Mouse coronary arteries contracted to 5-HT (110 ± 13% of the K+), and receptor characterization showed that the 5-HT2A receptor was responsible for the contraction. To ensure visible dilation, we used minimal precontraction for the coronary arteries (62 ± 4.9 % of K+) and basilar arteries (19 ± 6.1 % of K+). The coronary arteries dilated to CGRP (50 ± 7.8 %) but not to PACAP (-6 ± 2.7%). These results contrast to the basilar artery, which did not significantly contract to 5-HT (5.4 ± 1.8%, p=0.06). More important, the basilar artery did not dilate in response to CGRP (9±12%. P=0.46), whereas PACAP had a significant dilatory response (49 ± 4.4%. P=0.002).

Conclusion

These data show that using the mouse as a model organism for side effects of anti-CGRP treatments should be analyzed with care, as the arteries do not have similar expression profile as human arteries. In relation to migraine, it is interesting that cerebral arteries appear to lack CGRP receptors in mice.

## P4 Calcitonin gene-related peptide receptor antagonist BIBN4096BS regulates synaptic transmission in the vestibular nucleus and improves vestibular function via PKC/ERK/CREB pathway in an experimental chronic migraine rat model

### R. Tian, Y. Zhang, Q. Pan, Y. Wang, Q. Wen, X. Fan, G. Qin, D. Zhang, L. Chen, Y. Zhang, J. Zhou

#### The First Affiliated Hospital of Chongqing Medical University, Neurology, Chongqing, China

##### **Correspondence:** Y. Zhang

Questions: Vestibular symptoms are frequently reported in patients with chronic migraine (CM). The neuropeptide calcitonin gene-related peptide (CGRP) and CGRP1 receptor are essential to facilitate central sensitization in CM. However, the role of CGRP/CGRP1 receptor signaling in vestibular dysfunction after CM remains unclear.

Methods: A CM rat model was established by recurrent intermittent administration of nitroglycerin. Migraine- and vestibular-related behaviors were assessed. BIBN4096BS and protein kinase C (PKC) inhibitor-chelerythrine chloride were administered intracerebroventricularly. The expressions of CGRP and CGRP1 receptor components in the vestibular nucleus (VN) were evaluated. Synaptic associated proteins and synaptic morphological characteristics were explored. The expressions of down-stream molecules including PKC, phosphorylated extracellular signal regulated kinase (p-ERK), and phosphorylated cAMP response element-binding protein at serine 133 site (p-CREB-S133) were detected.

Results: The expressions of CGRP and CGRP1 receptor components were significantly upregulated in CM rats. CGRP1 receptor components were expressed mainly in neurons. BIBN4096BS treatment and PKC inhibition alleviated mechanical allodynia, thermal hyperalgesia and vestibular dysfunction in CM rats. Additionally, BIBN4096BS treatment and PKC inhibition markedly inhibited the overexpression of synaptic associated proteins and restored the abnormal synaptic structure in VN after CM. Furthermore, BIBN4096BS treatment dysregulated the expression levels of PKC, p-ERK and p-CREB-S133, and attenuated neuronal activation in VN after CM.

Conclusions: The present study demonstrated that CGRP1 receptor inhibition improved vestibular function after CM by reversing the aberrant synaptic transmission via downregulating PKC/ERK/CREB signaling pathway. Therapeutic interventions by inhibiting CGRP/CGRP1 signaling may be a new target for the treatment of vestibular symptoms in CM.

**Fig. 1 (abstract AP4).**
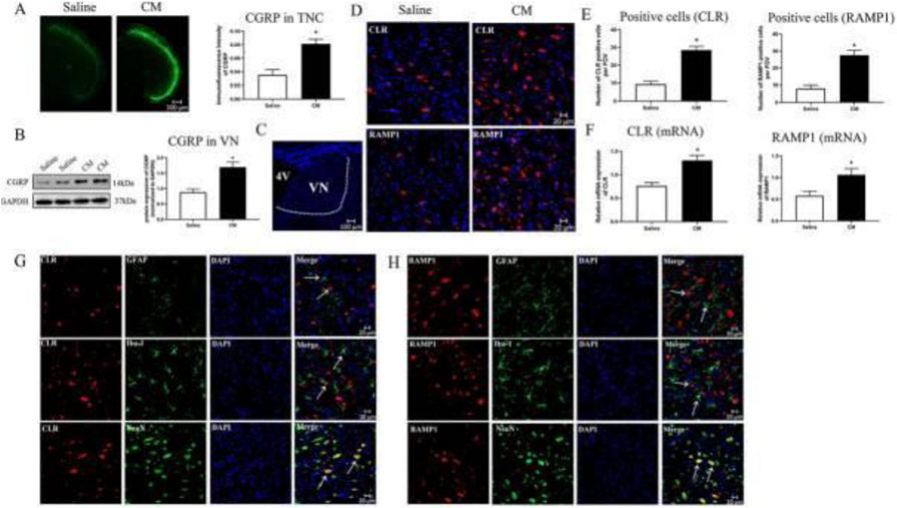
See text for description.

**Fig. 2 (abstract AP4).**
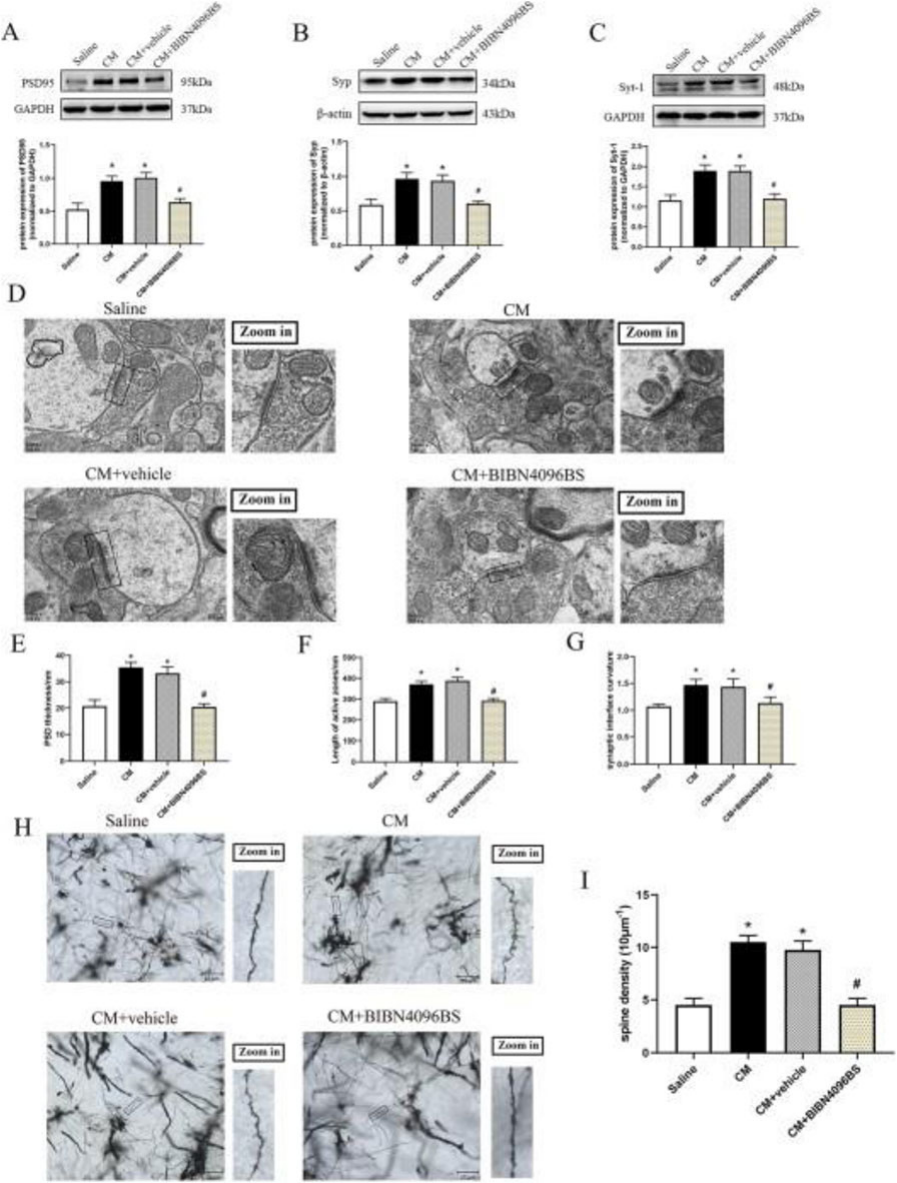
See text for description.

## P5 An open-source electrophysiology system to explore visual evoked potentials in migraine

### H. Zhou Chen^1^, S. Cooke^2^, P. Holland^1^

#### ^1^King's College London, Wolfson Centre for Age Related Disease, London, United Kingdom; ^2^King's College London, Institute of Psychiatry, Psychology and Neuroscience, London, United Kingdom

##### **Correspondence:** H. Zhou Chen

Question: Many studies have highlighted altered sensory processing underlying headache and non-headache symptoms in migraine, however a consensus is yet to be reached due to conflicting clinical data. Further electrophysiological investigation is needed to shed a light on pathophysiology and potential therapeutic targets, but existing tools are limited and expensive. We chose to study visual evoked potentials in a murine model of migraine using an open-source electrophysiology suite (Open-Ephys).

Methods: Visual evoked potentials (VEP) were obtained by LED flash stimulation (100 trials @ 1Hz) in anaesthetised mice (n=8) and acquired into Open-Ephys. Mice were injected with an acute dose of the migraine trigger nitroglycerin (10mg/kg i.p.) and VEPs were measured at 30min and 1hr. Data analysis was performed using custom MATLAB scripts to assess VEP amplitude, latency and spontaneous activity.

Results: We were able to record visual evoked potentials from V1 in response to flash stimuli using Open-Ephys. Preliminary data show NTG resulted in an increased VEP amplitude and a decreased latency of the P1 peak, associated with the extrastriate cortex. Additionally, spontaneous activity is shown to decrease after NTG injection, potentially reflecting vasodilatory effects of the compound.

Conclusion: Open-source electrophysiology systems offer a low-cost alternative to traditional systems and offer customiseable experimental tools tailored to research questions. Its open-source nature enables researchers to share and access neurophysiology tools used in other labs, which is particularly relevant to the headache field, as its pathophysiology most likely involves the interaction of diverse neural circuits which require equally complex tools to unravel. This study demonstrates its feasibility in investigating migraine-relevant cortical areas and further work will be conducted to explore multi-modal thalamocortical circuits in migraine.

## P6 Effects of caffeine on intracranial pressure and pain perception in freely moving rats

### I. M. E. Israelsen^1^, C. S. J. Westgate^1^, R. Højland Jensen^1^, S. Eftekhari^1^

#### ^1^Danish Headache Center, Glostrup Research Institute, Rigshospitalet-Glostrup, University of Copenhagen, Neurology, Glostrup, Denmark

##### **Correspondence:** S. Eftekhari

**Question:** Caffeine, a nonselective adenosine receptor antagonist, is the most commonly consumed psycho-stimulant in the world. Caffeine has been suggested to regulate cerebrospinal fluid (CSF) secretion and is known both to alleviate and to trigger headache. However, its effect on the regulation of intracranial pressure (ICP) is not known. Therefore, we aimed to investigate the effects of caffeine on ICP and pain perception.

**Methods:** Female Sprague Dawley rats (n=21) were implanted with a novel telemetric device for continuous ICP recordings which allowed for continuously recordings in freely moving rats. Single dose of caffeine (30 or 120 mg/kg i.p.) was given. In a second group (non-implanted), the acute effects of 30mg/kg caffeine on periorbital threshold using Von Frey and spontaneous behavior were utilized using an automated behavioral registration platform (LABORAS) in a randomized cross-over study. Immunofluorescence was performed to localize adenosine receptor (ARs) at choroid plexus (CP).

**Results:** Single dose of 30 mg/kg caffeine lowered ICP 5h after administration (saline: 0.16±0.9 vs caffeine: -1.18±0.9ΔmmHg, p=0.0098) and lasted up to 11h. Administration of 120 mg/kg caffeine showed a faster onset of decrease in ICP already within 15min (p=0.0018) and lasted up 12h. The periorbital pain thresholds were higher after 1h (saline: 224.6±15.1 vs caffeine: 289.5±8.7 g, p=0.005) and lasted up to 5h. After 5h of administration, the hind paw threshold was higher relative to vehicle (saline: 200.1±7.7 vs caffeine: 245.7±9.1g, p=0.03). Caffeine treated rats had increased locomotor activity, speed and rearing behavior. Expression of A1 was found at CP.

**Conclusions:** This study demonstrates that caffeine has a lowering effect on ICP as acute treatment and may act on the A1 receptor expressed at CP. Interestingly caffeine caused increased response in cephalic thresholds which was developed in an earlier stage of ICP reduction.

## P7 Capsaicin induced cell death in primary cultured neurons

### J. H. Lee

#### National Health Insurance Service Ilsan Hospital, Neurology, Goyang-si, South Korea

*Purpose*

To determine the effect of capsaicin to central nervous system, we prepared morphologic changes and biochemical assay were investigated in mouse primary cultured CNS neuron.

*Methods*

The susceptability of capsaicin differs for different brain area. Cerebral cortex and hippocampus were more sensitive, and striatum, thalamus and midbrain area were less sensitive to capsaicinsusceptability. After capsaicin treatment, cortical and hippocampal neurons were died in dose-and time-dependent manner. By observation of nuclear fragmentation of capsaicin treated neuron, it is thought that the type of cell death is apoptosis rather than necrosis. The capsaicin receptor immunoreactive cells were observed in the cortex and hippocampus.It is consistent with area of damaged neuron. In case of capsaicin treated neurons, NOS activity stain was positive, the product of nitrite and anti-nitrotyrosineimmunoreactivity were increased, and agmatine, which is a competitive nitric oxide synthases (NOSs) inhibitor significantly protect cortical and hippocampal neurons from capsaicin-induced apoptosis.

*Results*

These results indicated that capsaicin induced influx of cation ions. These results showed that capsaicin induced influx of Ca2+, followed by neuronal NOS is activated by Ca2+ and induced cell death. Also, the activity of caspase 3 was increased after capsacin treatment in the cortical and hippocampal neurons.

*Conclusions*

These results demonstrate that capsaicin induced the apoptosis through acting with capsaicin receptors. Calcium influx due to capsaicin recptor activation may induce apoptosis, which is triggered the formation of peroxynitrite by activating NOS activity or is mediated by activating caspase 3 pathway.

## P8 Src family kinases activity is required for transmitting purinergic P2X7 receptor signaling in contributing to cortical spreading depression propagation

### M. Wang, L. Nie, D. Ma

#### Xi'an Jiaotong-Liverpool University, Biological Sciences, Suzhou, China

##### **Correspondence:** M. Wang

Purinergic P2X7 receptor plays an important role in migraine pathophysiology. Yet precise molecular mechanism underlying P2X7R signaling in migraine remains unclear. This study aims to test the hypothesis that P2X7 receptor transmits signaling to Src family kinases (SFKs) during cortical spreading depression (CSD).

Methods: CSD was recorded using electrophysiology in rats or intrinsic optical imaging in mouse brain slices.

Results: The data showed that deactivation of SFKs by systemic injection of PP2 reduced cortical susceptibility to CSD in rats. Consistently, in mouse brain slices, inhibition of SFKs activity by saracatinib and P2X7 receptor by A740003 similarly reduced cortical susceptibility to CSD. When the interaction of P2X7 receptor and SFKs was disrupted by TAT-P2X7, a marked reduction of cortical susceptibility to CSD were observed in mouse brain slices. The reduced cortical susceptibility to CSD by TAT-P2X7 was restored by NMDA, and disrupting the Fyn-NMDA interaction using TAT-Fyn (39-57) but not disrupting Src-NMDA receptor interaction using TAT-Src (40-49) reduced cortical susceptibility to CSD. Furthermore, activation of P2X7 receptor by BzATP restored the TAT-Fyn (39-57)-reduced cortical susceptibility to CSD.

Conclusion: This study reveals that SFKs activity transmits P2X7 receptor signaling to facilitate CSD propagation via glutamatergic pathway, which is of particular relevance to migraine.

## P9 Evaluation of a human prolonged-release buprenorphine formulation in rats

### K. M. L. Nordahl^1^, S. Kazantzi^1^, L. Edvinsson^1,2^, K. A. Haanes^1^

#### ^1^Glostrup Research Institute, Rigshospitalet Glostrup, Department of Clinical Experimental Research, Glostrup, Denmark; ^2^University Hospital Lund, Department of Medicine, Lund, Sweden

##### **Correspondence:** K. A. Haanes

Objective: The development of novel targets within brain research often requires surgical procedures in rodents, and opioid analgesia is frequently needed postoperatively. Buprenorphine is a partial agonist of the μ-opioid receptors and has a strong analgesic effect when acting on the central nervous system. A long-acting buprenorphine formulation would be highly beneficial to avoid frequent dosing of postoperative animals. However, buprenorphine depot formulations developed for animal use are not available in Europe. The purpose of the present study was therefore to evaluate the effect on rats of a long-acting subcutaneous (s.c) depot injection with buprenorphine available for humans (Buvidal).

Methods: Spraque Dawley rats were used. The depot formulation (Buvidal, 1.5mg/kg s.c, n=24) was evaluated and compared to a short-acting buprenorphine formulation commonly used in laboratory rodents (Temgesic, 0.1 mg/kg s.c, n=18) and negative control (s.c saline, n=24). The analgesic effect was assessed using the von Frey pressure test on the plantar surface of the right hind paw, allowing analgesic efficacy to be evaluated without exposing the animals to any other pain. Post-dose results at 3h, 6h and 24h following injection were compared to pre-dose levels.

Results: At 3h and 6h post-dose both buprenorphine formulations showed a significant analgesic effect compared to baseline. At 6h the effect was more pronounced in rats that had received the depot injection. At 24h, only the depot formulation still showed a significant effect. Saline did not alter the sensitivity to the pressure test at any time-point.

Conclusion: A human prolonged-release buprenorphine formulation (Buvidal) available in Europe has long-term analgesic effect in rats (up to 24h), as evaluated using the von Frey pressure test. This formulation should be considered as an alternative to multiple injections of short-acting buprenorphine formulations in rat studies where opioid analgesia is desired.

## P10 Pharmacological characterization of gepants in human and porcine vasculature

### R. van Drie, D. Boucherie, T. de Vries, A. H. J. Danser, A. Maassen van den Brink

#### ^1^Erasmus Medical Center, Internal Medicine, Rotterdam, Netherlands

##### **Correspondence:** R. van Drie

**Objectives**

We aim to perform an in-depth pharmacological characterization of the potency of several gepants in porcine vasculature, in comparison with the potency in human blood vessels.

**Methods**

Distal coronary artery segments from 6 swine, obtained from the local slaughterhouse, were isolated and mounted in Mulvany myographs for isometric contraction measurements. Concentration response curves to human α-CGRP (10-10 – 3*10-6 M) were constructed in the absence or presence of increasing concentrations of olcegepant, rimegepant, zavegepant and telcagepant. The potency of the antagonists was determined by calculating pKb values. Results were compared to these obtained earlier in human isolated distal coronary arteries.

**Results**

Our results on olcegepant confirm our earlier observations (Gupta*, et al.* Eur J Pharmacol. 2006) of a lower potency in porcine coronary artery compared to human coronary artery (pKb 7.59±0.27 vs 9.13±0.17 respectively at 100 nM olcegepant). Preliminary results on rimegepant point to a similar difference in potency, as exemplified by the pKb values (100 nM: 6.35±0.04 vs 8.71±0.16, 1 μM: 6.35±0.04 vs 8.43±0.25 respectively) (Mulder, *et al.* Ann Neurol. 2020). Similarly, preliminary results on zavegepant again show a lower potency in porcine coronary arteries compared to human coronary artery (unpublished data) (pKb 100nM: 7.00±0.54 vs 9.91±0.15 respectively). For telcagepant an insufficient number of experiments was performed at the time of submission of the abstract.

**Conclusions**

Our initial analyses suggest that the difference in potency between porcine and human vasculature is similar for different gepants. Knowledge on this difference in potency between species, combined with molecular information about the structure of the CGRP receptor and the antagonists, provides information on the implications of the potency of gepants for translational research in experimental animals.

## P11 Spinal manipulation therapy in the therapeutic management of headaches- a systematic review

### M. Maia^1^, J. A. Rodrigues Simoes^1^, J. F. Moreira Craveiro^2^

#### ^1^FCS-UBI, Covilhã, Portugal; ^2^Centro Hospitalar Povoa de Varzim e Vila do Conde, Porto, Portugal

##### **Correspondence:** M. Maia

**Question/Problem statement:** The recommended treatment for headache is generally based on a combination of non-pharmacological and pharmacological approaches, with greater emphasis on the latter. However it doesn"t always allow to achieve a satisfactory symptomatic control. The high prevalence of headaches, associated with the low efficacy of conventional treatments, has led to an increase in the demand for alternative therapies, like spinal manipulation therapy (SMT).

**Objective**:To determine the effectiveness of SMT in the therapeutic management of headache in adults.

**Methods**:A protocol defining the investigation strategy was published on PROSPERO and a research was carried out in MEDLINE/PubMed, Cochrane and Scopus databases for clinical trials published in English and Portuguese, between 2011 and August of 2021. The keywords used were: "spinal manipulation" OR chiropra* AND (headaches OR migraine). The research, selection and analysis of articles was performed independently by three investigators. The main outcomes analyzed were the effectiveness of SMT in therapeutic management of headache (on pain intensity, frequency and duration) and its impact on patients" quality of life.

**Results**:Twelve clinical trials were included in the present review. SMT was apparently able to reduce the frequency and intensity of headache, both in patients with primary headaches and secondary headaches. These results were noticed immediately after the application and long term. On the contrary, there wasn"t enough information allowing to conclude about the effect on headache"s duration or on patients" quality of life.

**Conclusion**:SMT has the potential to become a complementary therapy in the management of headaches, with particular significance in reducing its frequency and intensity. However, the studies" considerable risk of bias compromises its quality and hinders its immediate clinical applicability. We recommend developing new clinical trials with more rigorous methodologies.

**Fig. 1 (abstract P11).**
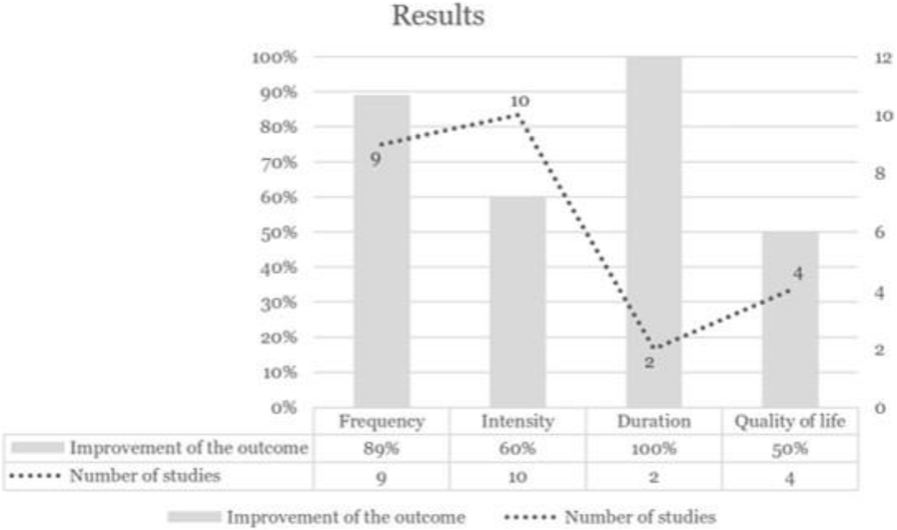
See text for description.

## P12 Hypothalamic paraventricular stimulation inhibits trigeminal nociceptive transmission via oxytocin receptors

### A. González-Hernández, G. Martínez-Lorenzana, M. Condés-Lara

#### Universidad Nacional Autónoma de México, Instituto de Neurobiología, Queretaro, Mexico

##### **Correspondence:** A. González-Hernández

**Question.** Recent data suggest that exogenous oxytocin exerts antinociception at the trigeminal level. Although this peptide is released from the hypothalamic paraventricular nucleus (PVN), little is known about the role of endogenous oxytocinergic neurotransmission modulating trigeminal nociception. This study tested the effect of PVN stimulation on the trigeminal nociceptive responses elicited by activation of the trigeminal nerve.

**Methods.** *In vivo* electrophysiological recordings of trigeminal WDR cells and immunohistological studies were performed in rats. The animals were anesthetized with sevoflurane, assisted with mechanical ventilation, and mounted in a stereotaxic frame. A surgery to access the medullary dorsal horn was performed, and a small craniotomy was made to place a concentric stainless-steel stimulation electrode (1 MW) in the PVN. Under this condition, extracellular unitary recordings of trigeminal WDR cells with input from the first branch of the trigeminal nerve (V1) were made with quartz-Pt-W microelectrodes (4-10 MW). In addition, retrograde neuronal tracing with fluoro-gold® from the spinal trigeminal region to the PVN was assessed in search of oxytocinergic fibers.

**Results.** PVN electrical stimulation (6 sec, 60 Hz, 1 msec pulse duration, 300 mA) inhibited the peripheral evoked trigeminal nociceptive responses. This inhibition was reversed by a peptide OTR antagonist given spinally (dOVT, d(CH_2_)_5_[Tyr(Me)^2^,Thr^4^,Tyr-NH_2_^9^]OVT). Furthermore, the retrograde labeling showed that direct oxytocinergic projections from trigeminal nucleus *caudalis* to PVN exist.

**Conclusion.** Coupled with previous reports showing that exogenous oxytocin administration at the trigeminal level inhibited the periorbital nociceptive responses via OTR, our data strongly support the notion that PVN via oxytocinergic transmission inhibits trigeminal nociception, suggesting that enhancement of oxytocinergic transmission could be used as a potential therapy to treat headaches.

## P13 Long-term effectiveness and safety of occipital nerve stimulation in medically intractable chronic cluster headache: a prospective follow-up study of the randomised controlled ICON trial.

### R. Brandt^1^, L. Wilbrink^2^, W. Mulleners^3^, F. Huygen^4^, E. van Zwet^5^, M. Ferrari^1^, R. Fronczek^1^

#### ^1^Leiden University Medical Center, Neurology, Leiden, Netherlands; ^2^Zuyderland Hospital, Neurology, Heerlen, Netherlands; ^3^Canisius-Wilhelmina Hospital, Neurology, Nijmegen, Netherlands; ^4^Erasmus Medical Center, Anaesthesiology, Rotterdam, Netherlands; ^5^Leiden University Medical Center, Biomedical Data Sciences, Leiden, Netherlands

##### **Correspondence:** R. Brandt

**Question:** We have shown in the ICON study that occipital nerve stimulation (ONS) is safe and effective in medically intractable chronic cluster headache (MICCH). This prospective follow-up evaluates the long-term effectiveness and safety.

**Methods**: Every six months, participants completed questionnaires on the attack-frequency, adverse events, subjective improvement and willingness to recommend this treatment to other patients. Missing values for log-transformed attack-frequency were imputed for up to 5 years of follow-up. Descriptive analyses are presented as (pooled) geometric or arithmetic means and 95% confidence intervals.

**Results:** Of the n=119 eligible participants, n=88 (74%) provided informed consent and were followed ≥2 years unless the device was prematurely removed. There were n=73 (83%) active participants after 2 years, n=60 (68%) after 3 years, n=32 (36%) after 5 years and n=3 (3%) after 8.5 years. Mean follow-up was 4.2 ± 2.2 years for a total of 370 person-years. Of the 49/88 (56%) ≥50% responders at the end of the ICON study, 35/49 (71%) retained this response and 15/39 (38%) of the non-responders became a ≥50% responder for at least half the follow-up period. The pooled geometric mean [95% CI] weekly attack frequency remained considerably lower after one (4,2; 2,8 - 6,3), two (5,1; 3,5 - 7,6) and five years (4,1; 3,0 - 5,5) compared to baseline (16,2; 14,4 - 18,3). Most participants (69/88; 78%) reported a subjective improvement from baseline at the last follow-up and 70/88 (81%) would recommend this treatment to other patients. Additional surgery was required in 112/122 (92%) hardware related events in 44/88 participants (50%), corresponding to a hardware-related additional surgery rate of 0.35 person-year-1 [0.28 – 0.41]. No predictive factors for effectiveness at 2 years after the ICON study, i.e. 3 years after ONS implantation were observed.

**Conclusions:** ONS is a safe, well-tolerated and long-term effective treatment for MICCH.

## P14 Efficacy and safety of Galcanezumab as chronic cluster headache preventive treatment in real world conditions.

### R. Lamas Pérez, M. Millán Vázquez, C. González Oria

#### Hospital Universitario Virgen del Rocío, Neurology, Seville, Spain

##### **Correspondence:** R. Lamas Pérez

**Question:** Calcitonin gene-related peptide (CGRP) has shown to play a pivotal role in cluster headache (CH) pathophysiology. A clinical trial with Galcanezumab has been carried out in chronic cluster headache (CCH) that did not achieve significant reduction of headache attacks. However, its off-label use in patients with CCH refractory to other therapies could be considered.We aim to asses the efficacy and security of Galcanezumab as preventive treatment in a CCH population in a real-life setting.

**Methods**: Observational prospective study. CCH patients who received at least 1 administration of 240mg monthly subcutaneous Galcanezumab. Data were obtained from clinical interviews, headache diaries, dissability scales and PGIC score.

**Results:** 21 patients, 76.2% males, mean age of 47.8 years with 12.2 years of CH. 6.3±1.9 previous preventive therapies, incluiding onabotulinumtoxinA in 90.5%. Furthermore, occipital neuroestimulation in 38.1%, occipital radiofrequency in 9.5% and GON section in 4.8%. The average number of attacks per month was 76.6±61.1 with 8.9±1.5 intensity (NRS) at baseline. After one month of treatment number of attacks reduced to 34.7±25.3 with 8.1±17 intensity; 10(47.6%) patients achieved a reduction of at least 50% in monthly headache attacks, of which 4(19%) achieved a 75% reduction. Triptans abusers reduced from 61.9% to 33.3%. Of the 15 patients of whom we have 3 months follow-up, 7(46.6%) reduced their monthly attacks by 50% and 4(26.6%) 75%, with an average of attacks/month of 35.9±28.1 and an intensity of 7.5±2.3. Triptans abusers were 26.6%. 47.6% considered the improvement as a real difference in their lives (PGIC≥5) after 1 dose of Galcanezumab and 60% after 3 doses. 52% experienced adverse events, mostly mild, most common constipation (19%) leading to discontinuation in 1 patient.

**Conclusions:** Despite how refractory our CCH cohort is, Galcanezumab was effective in nearly 50% patients. This supports individual off-label treatment attempts.

## P15 Intra- and inter-individual attack frequency variability of chronic cluster headache

### R. Brandt^1^, W. Mulleners^2^, L. Wilbrink^3^, P. Brandt^4^, E. van Zwet^5^, F. Huygen^6^, M. Ferrari^1^, R. Fronczek^1^

#### ^1^Leiden University Medical Center, Neurology, Leiden, Netherlands; ^2^Canisius-Wilhelmina Hospital, Neurology, Nijmegen, Netherlands; ^3^Zuyderland Hospital, Neurology, Heerlen, Netherlands; ^4^TU Eindhoven, Electronic systems, Eindhoven, Netherlands; ^5^Leiden University Medical Center, Biomedical Data Sciences, Leiden, Netherlands; ^6^Erasmus Medical Center, Anaesthesiology, Rotterdam, Netherlands

##### **Correspondence:** R. Brandt

**Question:** Little is known regarding the AF variability of CCH, hampering power and sample size calculations, and consensus on the most optimal duration of pre-trial baseline observation periods.

**Methods**: We used detailed data from the 12-week baseline period of the randomized controlled occipital nerve stimulation ICON trial in patients with medically intractable CCH. Participants were post hoc divided into four mean daily AF groups: ≤2; >2-3; >3-4; >4. We analyzed the following four variables for the total and four AF groups: (i) weekly vs. instantaneous recording of the AF; (ii) intra-individual AF variability by using (a) the mean absolute deviation from the mean and (b) the coefficient of variation; (iii) seasonal variability of the AF; (iv) the smallest number of weeks to obtain a reliable estimate of the baseline AF over the entire 12-week period.

**Results:** Weekly median (14.4 [8.2 – 24.0]) and instantaneous (14.2 [8.0 – 24.5]) AF recordings were similar (p=0.20; Bland-Altman plot). The median weekly AF over all 12 weeks was 15.3 (range 4.2-140). Absolute AF variation was lower in the lowest AF group in comparison to the other AF groups (p<0.001). Relative AF variability decreased with increasing AF (p=0.010). During spring AF was higher compared to the other seasons (p=0.001). We tabulated the weekly AF estimation accuracies compared to, and the associated deviations from the 12-week gold standard for different lengths of the observation period.

**Conclusion:** Weekly retrospective recording of the AF is as good as instantaneous recording and more convenient. Participants with ≥3 daily attacks show less AF variability than those with <3 daily attacks. Mean AF is highest in spring. The data suggest that an optimal balance between feasibility and an accuracy of 90% with a deviation of no more than 20% is achieved at an observation period of 7 weeks.

## P16 Galcanezumab for refractory chronic cluster headache in clinical practice

### D. I. Samuel, N. C. Candela, O. Marina Alejandra, F. M. Maribel, M. J. Navarro Muñoz, J. Perez Garcia

#### Hospital Universitari i Politècnic La Fe, Neurology, Valencia, Spain

##### **Correspondence:** D. I. Samuel

QUESTION:

To evaluate the efficacy and safety of galcanezumab for refractory chronic cluster headache (RCCH) patients in clinical practice. RCCH patients usually fail to most of preventive treatments started on them. There"s a need for new drugs to improve their bad quality of life.

METHODS:

We prescribed galcanezumab 360 mg alternating with 240 mg per month in RCCH patients willing to participate in this exploratory study. All of them received at least a 6 month treatment period. They had tested all kind of preventive treatments, including botulinumtoxinA injections. No previous treatment was stopped, galcanezumab was started as add-on therapy. We analyse mean cluster headache attacks (CHA) per week reduction after treatment. Adverse events were also collected. Informed consent was obtained.

RESULTS:

7 patients (6 men / 1 woman) 23-59 years (mean 44,43) were included in the registry, all of them at least > 2 years of RCCH (mean 12,57 years (4-24). Most of them were smokers, just one with alcohol consumption. 3 of them continued with botulinumtoxinA injections each 3 months due to partial response. Mean CHA of 24,14 per week before treatment. 9,29 CHA per week after treatment; this means a reduction of 54,2 % in CHA per week (3,7 – 95 %). In most patients a significant reduction in intensity was found. 3 of them were high responders (reduction more than 75 %) and 3 no responders (25 % or less). No significant adverse events were found, the most usual: local dermal reactions in sites of punction (erythema, swallowing, pruritus)

CONCLUSIONS:

High doses of galcanezumab can be an option for RCCH patients. The treatment is safe and ameliorates at least half of patients in which we prescribed it. In this very difficult to treat population any benefit is welcome, so the use of galcanezumab should be tested in RCCH patients.

## P17 A Systematic Review and Meta-Analysis on the Preventive Treatment of Refractory Chronic Cluster Headache

### J. A. Membrilla, J. Roa, J. Díaz de Terán

#### University Hospital La Paz, Neurology, Madrid, Spain

##### **Correspondence:** J. A. Membrilla

QUESTION- Which is the best preventive treatment strategy for Refractory Chronic Cluster Headache (rCCH) based on current scientific evidence?

METHODS- The review and meta-analysis were performed following PRISMA guidelines. The protocol was registered in PROSPERO (ID CRD42021290983). The search was performed on September 2021 on databases Pubmed, Embase and Cochrane. Studies of preventive strategies for rCCH defined by the European Headache Federation criteria were selected. For risk of bias assessment, the Cochrane Handbook Risk Of Bias tool was used for randomized clinical trials (RCT) and ROBINS-E was used for observational studies (OS).

RESULTS- 43 articles met the inclusion criteria. The largest number of articles studied occipital nerve stimulation (ONS) accounting for 1 ECA and 11 OS for a total of 436 patients, followed by deep brain stimulation (DBS): 1 RCT and 8 OS; 118 patients. All ONS studies reported a significant reduction in attack frequency and the 50% responder rate ranged from 29.4% to 80.0%. Meta-analysis of ONS studies revealed a pooled 50% responder rate of 57.3% (95%CI 0.48-0.67, p<0.001). DBS studies reported a 50% responder rate of 50-100%, with a pooled result of 71.6% (95%CI 0.45-0.978, p<0.001). Reported adverse events (AE) were more serious in DBS studies. The remaining 24 articles (anti-CGRP pathway drugs, ketamine-magnesium infusions, serial occipital nerve blocks, clomiphene, onabotulinum toxin A, ketogenic diet, sphenopalatine ganglion radiofrequency or stimulation, vagus nerve stimulation, percutaneous bioelectric current stimulation, upper cervical cord stimulation and vidian neurectomy) present weaker results or have poorer quality of evidence.

CONCLUSIONS- Considering the quality of the published studies, their results and the profile of AE, ONS could be the first therapeutic strategy for patients with rCCH based on the current evidence.

**Fig. 1 (abstract P17).**
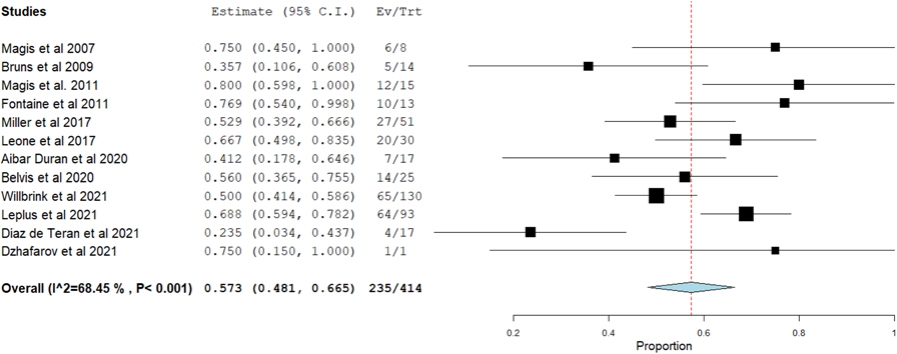
See text for description.

## P18 Side-shift of cluster headache attacks after Greater Occipital Nerve-injection

### W. Naber^1^, R. Brandt^1^, R. L. Ouwehand^1^, J. Haan^1^, M. Ferrari^1^, R. Fronczek^1^

#### ^1^Leiden University Medical Center, Neurology, Leiden, Netherlands

##### **Correspondence:** W. Naber

**Objectives:** Attacks of cluster headache (CH) are usually side-locked in most, but not all patients. In a few patients, the side may alternate between or, rarely, within cluster episodes.

**Methods:** We observed 7 cases in whom the side of CH attacks shifted immediately or shortly after unilateral injection of the greater occipital nerve (GON) with steroids. Side-shift was defined as a temporary or persistent displacement of CH attacks to the contralateral side of the regular headache attack previous to the GON-injection.

**Results:** In five patients with previously side-locked CH attacks and in two patients with previously side-alternating CH attacks, a side-shift occurred immediately (N=6) or shortly (N=1) after GON-injection.

**Conclusion:** Unilateral GON-injections might cause a side-shift of CH attacks, illustrating the complex role of the GON in CH pathophysiology.

**Table 1 (abstract P18).**
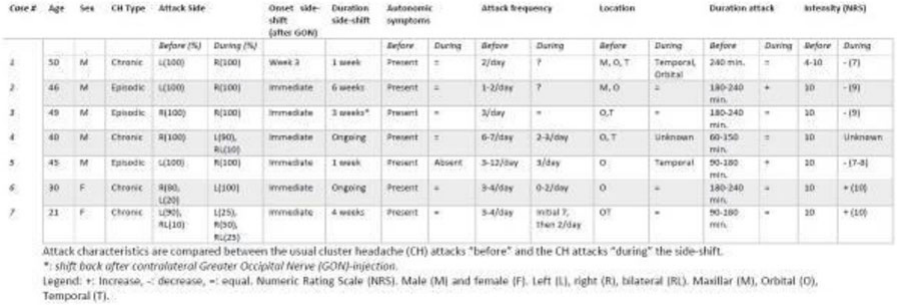
Patient and side-shift characteristics

**Fig. 1 (abstract P18).**
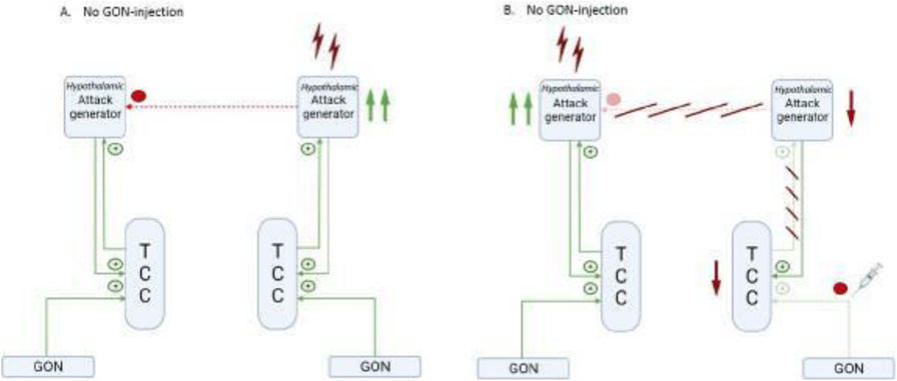
Proposed mechanism of side-shifts after GON injection

## P19 Retrospective Validation of a New Tool for the Identification of Suspected Cluster Headache

### M. Maher, W. Kingston

#### University of Toronto, Neurology, Toronto, Canada

##### **Correspondence: M. Maher**

**Question:** Cluster headache is a disorder often referred to as "suicide headache" and is associated with significantly disabling attacks [Newman et al 2015]. Although it is as prevalent as neurological conditions such as multiple sclerosis [Wei et al.2018, Leray et al. 2016] these patients often go over 5 years before diagnosis [Rosen et al. 2012]. This study aims to determine whether a screening tool can be retrospectively validated to reliably identify patients with a high likelihood of having cluster headache to prioritize rapid consultation with a headache specialist. **Methods**: A screening clinical questionnaire was developed utilizing criteria specific to cluster and to best differentiate it from other headache disorders. The tool was then retrospectively applied to all patients seen at a tertiary care specialty headache clinic between January 2021 and December 2021. Eligibility criteria include adult patients (>18 years), any reason for referral, any referring headache diagnosis and an initial consultation note including all data required to complete the scoring tool. A total score was calculated for each patient and the area under the receiver operating curve (ROC) used to identify the score with the highest sensitivity and specificity for a diagnosis of cluster headache. **Results**: A total of 415 patients were ultimately included in the study, of which there were 25 cluster headache patients. identified with an average screening score of 12.28. In comparison, the 255 migraine patients included had an average score of 1.45 on the screening tool. A ROC analysis indicated that a cutoff score of 8 had a 100% sensitivity and 85-87% specificity for a diagnosis of cluster headache. **Conclusions:** Our study was able to develop and retrospectively validate a cluster headache screening tool that can reliably differentiate cluster headache from other disorders. **Figure 1.** Cluster headache screening tool **Table 1.** Average score by headache disorder **Table 2.** Coordinates of the ROC.

**Fig. 1 (abstract P19).**
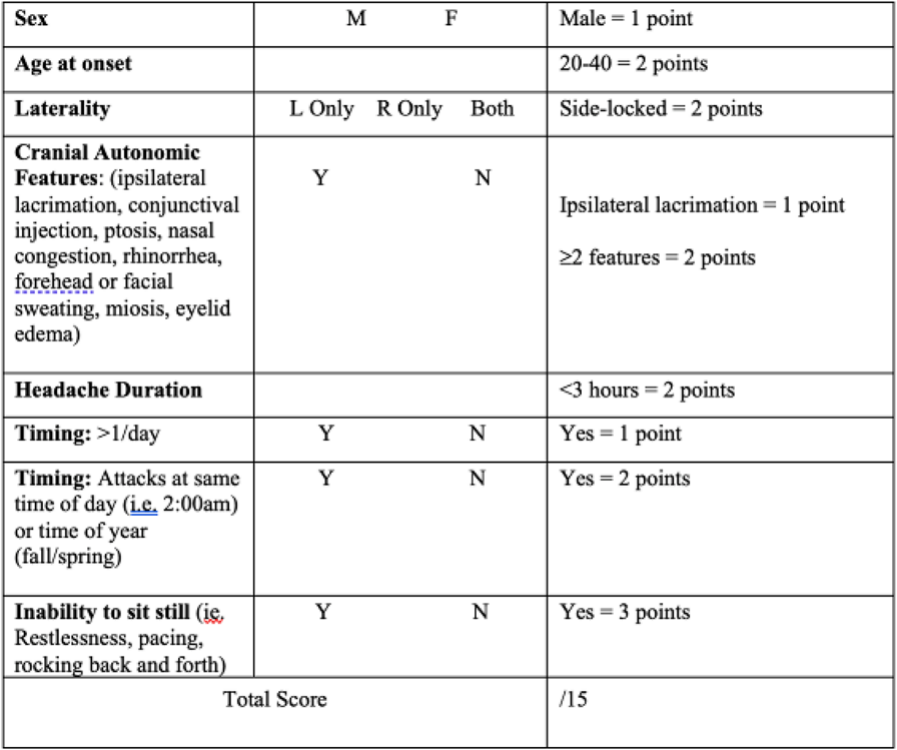
Cluster headache screening tool

**Table 1 (abstract P19).**
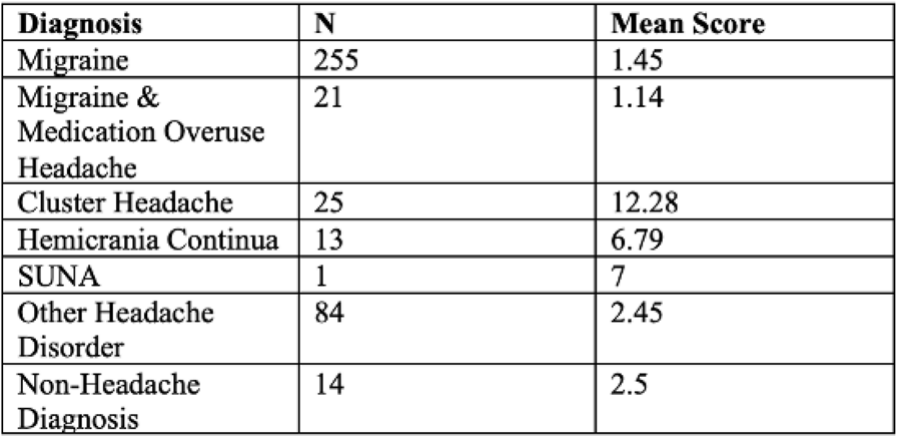
Average core by headache disorder

## P20 Cluster Headache – The Worst Possible Pain on YouTube

### B. A. Chaudhry, T. P. Do, H. Ashina, M. Ashina, F. M. Amin

#### University of Copenhagen, Rigshospitalet, Neurology, Copenhagen, Denmark

##### **Correspondence:** B. A. Chaudhry

**Background:** In clinical practice, patients with cluster headache often ask questions or mention information that they have seen or heard on the Internet. Since YouTube (www.youtube.com) is the second most visited Web site worldwide and offers a plethora of video content, we found it timely to ascertain the quality of information on cluster headache that is freely available on YouTube.

**Methods:** We conducted an inquiry on YouTube on January 24, 2022, with the search term "cluster headache". Eligible YouTube videos included those with ≥10.000 views and content related to cluster headache. We assessed the quality and reliability of the videos with the Global Quality Scale and DISCERN, respectively.

**Results:** The search strategy identified 644 videos of which 134 were eligible for inclusion. The sources of the included videos were categorized as "Healthcare Professional/Institution" (n=45), "Personal Experience" (n=52), and "Other" (n=37). According to the Global Quality Scale, 70 (52%) were low quality, 34 (25%)

were of moderate quality and 30 (22%) were of high quality. According to DISCERN, 104 (78%) were of low reliability, 28 (21%) were of moderate reliability and 2 (1%) were of high reliability.

**Conclusion:** The quality and reliability of cluster headache-related information on YouTube has room for improvement, even the content provided by healthcare providers. These findings should incentivize stakeholders, e.g., governmental services, professional societies, healthcare providers, to provide accessible and better information on cluster headache.

**Fig. 1 (abstract P20).**
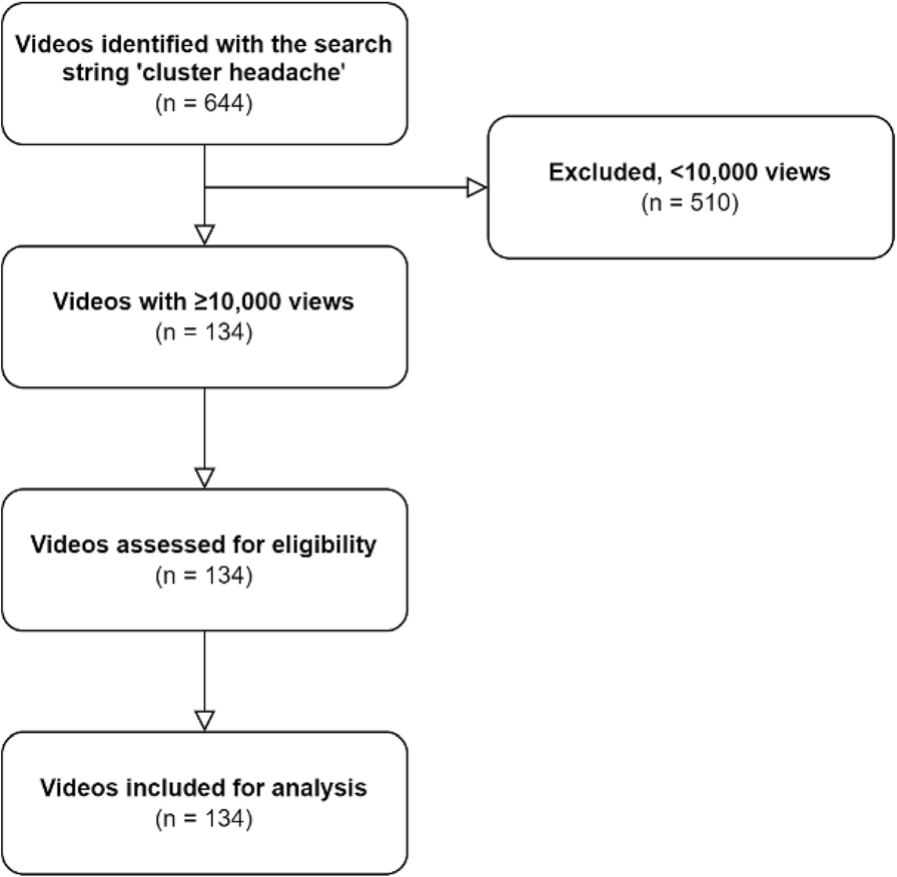
See text for description.

**Fig. 2 (abstract P20).**
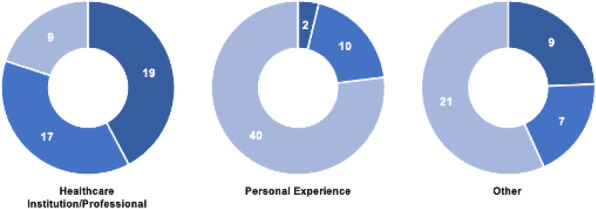
See text for description.

## P21 A retrospective evaluation of use of gammaCore at The Walton Centre NHS Trust

### M. Ghadiri-Sani, C. Bradley, M. Prewett

#### Walton Centre NHS foundation Trust, Neurology, Liverpool, United Kingdom

##### **Correspondence:** M. Ghadiri-Sani

**Introduction**

GammaCore is a non-invasive vagus nerve stimulator (nVNS) which modifies pain pathways and has been approved by NICE for management of cluster headaches. For use in other headache disorders, in the UK, an IGFR form needs to be completed.

Cluster headache (CH) is a trigeminal autonomic cephalalgia characterised by severe, strictly unilateral headache attacks accompanied by ipsilateral autonomic symptoms and restlessness associated with suicide.

**Objective**

To assess the effectiveness of gammaCore in management of headache disorders

**Methods**

Retrospective review of 100 patients who are currently using gammaCore was undertaken

**Results**

Out of 100 patients (F:M 62:38), with an average age of 48, 52 had CH, 39 hemicrania continua (HCC), 3 SUNCT, 4 NDPH and 2 CM.

On Average 6 previous preventatives were tried including Verapamil, Botox and CGRP MABs. Duration of treatment was 1.5 years on average, with one patient on treatment for over 6 years.

Headache diaries was available in 57 patients at start of treatment and 36 at 3 months, 20 at 6 months and 16 at 1 year. At 3 months, there was 6 days reduction in severe days and 7 day increase in clear days for the entire group. Patients with CH continued to show 8 days reduction in severe days at 1 year. Please see attached table.

**Conclusion**

In our experience, GammaCore is effective in management of a variety of headache disorders, particularly CH.

The flaws in our study are limited headache diary data, due to a number of factors including lack of compliance and storage of the information, and lack of data on tolerability.

## P22 Galcanezumab vs Placebo in Cluster Headache Prevention and Treatment: A Systematic Review

### A. Cyntia Lima Fonseca Rodrigues^1^, B. Vieira Nogueira^2^, J. Souto Faria Navarro^3^, L. Lopes Penido de Mendonça^4^, L. Bernardes da Silva Neves^4^, F. Morais Pereira de Medeiros^4^, G. Curvelo Bernardes Silva^5^, V. Marone Barros Lopes^4^, R. Barbosa Mokdeci Surerus^6^, T. Nizoli de Campos^7^, A. S. Crestani^8^, L. Giuliani Schmitt^9^, E. Vieira Nascimento^8^, M. V. Delgobbo Pereira^8^, M. L. Ribeiro Lodo^10^, R. do Couto Soares Caversan^11^, I. Audacio Ramos Fernandez^12^, M. Zago Mazzini^12^, R. Carlos Soares^10^, S. Gonçalves Alencar Rodrigues Furtado Rocha^10^, S. Bazo Diniz^13^, M. Lorini Rodrigues^10^, M. P. Batista Aveaneda^12^, V. Alves da Silva^10^, A. Magna Garib^12^, L. Luminati Picolo de Oliveira^12^, C. E. Bazo Diniz^14^, Y. Ribeiro Basseto^12^, L. Kassburg Mello^10^, I. Biglia Diniz^15^, R. Agustinho Rodrigues^16^, L. Correia da Cruz^15^, N. Mazetto Rocha^15^, V. E. Gomes Lomba^15^, S. Fortes Garib Batista^17^, B. A. da Silva^10^, A. Toyokytty Yoshida^15^, J. Spaggiari Marra^18^, M. A. de Castro Olyntho Júnior^19^, F. Farah Sabe^18^, G. Macedo Goedert^8^, L. Bandeira Rocha^20^

#### ^1^Universidade Positivo, Curitiba, Brazil; ^2^Centro Universitário Serra dos Órgãos, Teresópolis, Brazil; ^3^Universidade Federal de Mato Grosso, Cuiabá, Brazil; ^4^Universidade Estácio de Sá, Rio de Janeiro, Brazil; ^5^Universidade de Vassouras, Vassouras, Brazil; ^6^Universidade Federal de Juiz de Fora, Juiz de Fora, Brazil; ^7^Universidade Iguaçu, Nova Iguaçu, Brazil; ^8^Pontifícia Universidade Católica do Paraná, Curitiba, Brazil; ^9^Universidade Federal de Santa Maria, Santa Maria, Brazil; ^10^Centro Universitário das Américas, São Paulo, Brazil; ^11^Santa Casa de Misericórdia de Birigüi, Birigüi, Brazil; ^12^Universidade de Marília, Marília, Brazil; ^13^São Leopoldo Mandic Araras, Araras, Brazil; ^14^Centro Universitário Redentor, Itaperuna, Brazil; ^15^Fundação Educacional do Município de Assis, Assis, Brazil; ^16^Centro Universitário Católico Salesiano Auxilium, Araçatuba, Brazil; ^17^Faculdade Adamantinense Integrada, Adamantina, Brazil; ^18^Ribeirão Preto Universidade de São Paulo, São Paulo, Brazil; ^19^Olyntho Oftalmo Center, São José do Rio Preto, Brazil; ^20^Hospital Geral de Nova Iguaçu, Nova Iguaçu, Brazil

##### **Correspondence:** A. Cyntia Lima Fonseca Rodrigues

Question: Cluster headache (CH) causes excruciating unilateral temporal or periorbital pain with ipsilateral autonomic symptoms. Classified as primary headache, it is characterized by daily headaches that persist for weeks to months with direct impact on quality life. Treatment with galcanezumab (GZB) is an upcoming option for preventive treatment of CH. What is the impact of this new therapy in preventing and reducing the frequency of CH? Methods: A total of 122 articles were identified in databases: Pubmed, Embase and Cochrane), by two independent researchers. Three randomized controlled trials were included in this systematic review using a PRISMA protocol. The outcome of interest was change in weekly frequency of CH attacks and mean reduction in attack frequency compared with baseline. Results: A total of 449 patients were randomized into 2 groups, the intervention group received the standard dose of 300mg of GZB versus the placebo control group. The studies primarily evaluated the reduction of CH and the mean >= 50% reduction in attack frequency compared with baseline. Goadsby et al. obtained a mean percentage reduction in the weekly frequency of CH attacks in the first 3 weeks of 52% in the intervention group and 27% in the placebo group. Kudrow et al. reported the observed reduction by approximately 43% in the intervention group over the same period. Both studies showed compatible efficacy reducing attacks >= 50% (71% (p=0.046) and 78.3%, respectively). However, Dodick et al. showed no statistically significant effect of GZB in both outcomes: the reduction in weekly attack frequency (p=0.334) and mean reduction in attacks 32.6% (p=0.170). Conclusions: The effects of GZB in reducing the number of seizures, especially in the first few weeks of treatment, are encouraging, although further studies are required to establish the efficacy of GZB in long-term use for CH prevention and treatment.

## P23 Coexistence of migraine and cluster headache - is it more than just a coincidence?

### J. Gulišija, V. Košta

#### University Hospital Center Split, Neurology, Split, Croatia

##### **Correspondence:** J. Gulišija

**Introduction:** Cluster headache (CH) and migraine are recurrent painful primary hedaches. They are typically presented with different clinical appearance, but they show some common features, such as unilateral pain, triggers, pathophysiological mechanisms and response to therapy (triptans and monoclonal antibodies against the calcitonine-related peptide receptors). In the general population, the prevalence of CH and migraine is estimated to 0,1% and 12% respectively. Their coexistence is not common, though ranging from 0-65%^1^. Some data are showing that in 92% of migraine begins first and persists in only 20% of patients after CH began^2^.

**Case report:** A 30-year-old woman presented with typical CH lasting for 3 weeks. Headache was present every day, twice a day - one during a day and once during at night. If untreated duration was 3 h. Rizatriptan coud release her from pain, while ibuprofen and paracetamol were not helpful. She already had a headache with similar characteristics at the age of 17. Her migraine attacks with complex aura started at the age of 13. Attacks were quite infrequent, till age of 28, after she gave birth, and the attacks became much more frequent. In her medical history there were no other diseases and she was not taking any medication.

**Conclusion:** The aim of the present report was to draw attention to coexistence of CH and migraine since it can go unrecognised in clinical practice. Better education of health-care providers and spreading awarness of possibile coexistence of more primary headaches could provide better life quality for our patients. Only population-based studies can confirm or disprove an association between migraine and CH by determing whether they occur together more commonly then expected by chance. Consent to publish had been obtained.

Reference list:

1. Ewans W. et al, Migraine and CH:Coexistence,Laterality and Gender,2004,44;186-188.

2. Solomon S. et al., The time relationship of migrane and CH when occuring in the same patient, Headache, 1986;26:500-502.

## P24 Clinical neurophysiology of hemiplegic cluster headache - case report

### A. Pantovic, Z. Boskovic, A. Markovic, A. Jovanovski

#### Military Medical Academy, Neuroloy clinic, Belgrade, Serbia

##### **Correspondence:** A. Pantovic

Introduction

Cluster headache (CH) is trigeminal autonomic headache presented by the recurrence of unilateral short duration (15-180min) pain attacks with coexisting same-side cranial autonomic symptoms. The atypical form of cluster headache associated with transient hemi-motor, sensory or even visual and aphasic symptoms analogically to migraine is called hemiplegic cluster headache (HCH). This rare form of trigeminal autonomic cephalalgia is not recognised by the International Classification of Headache Disorders (ICHD). Since the first published case series in 2002, only a few cases have been presented so far.

Case presentation

We presented a case of a 50-year-old male that fulfilled the ICHD criteria for episodic CH who experienced atypical attacks characterized by concomitant acute onset of hemi-sensory and hemi-motor symptoms. Using extensive diagnostic panel we excluded the secondary cause of the headache. Exploring the localisation of motor and sensory pathways affectation we used the sensory and motor evoked potentials method.

Conclusion

Neurophysiological parameters show that during the cluster period there is a transient affectation of sensory and motor pathways in the projections of the brain stem and medulla oblongata. This points to potential differences in the mechanism of neural pathway involvement among HCH and different types of primary headaches with coexisting motor and sensory symptomatology. Consent to publish had been obtained.

## P25 Headache is a common aura in patients with generalized seizures at a younger age of onset

### C. Cho, D. W. Kim

#### Konkuk University School of Medicine, Neurology, Seoul, South Korea

##### **Correspondence:** C. Cho

Question: Although the pathophysiology of headache as an epileptic aura is frequently attributed to the excessive neocortical cellular excitability of the parieto-occipital cortex in patients with focal seizures, several studies have documented headache may occur as an aura or isolated epileptic symptom in patients with generalized seizures. Currently, there is limited information on the headache as an aura in patients with generalized seizures.

Methods: We performed a 14-year retrospective study of patients with generalized seizures who received at least 6 months of treatment in our epilepsy clinic. Information on the characteristics of aura and seizure semiology were obtained through review of medical records. The proportion of patients who experienced an aura including headache and the demographic and clinical characteristics of the patients with and without headache as an aura were further analyzed.

Results: We included 102 patients diagnosed with generalized seizures and received treatment for at least 6 months. The patients were 44 males and 58 females. The most frequent seizure types were absence seizures in 8 patients, myoclonic seizures in 54 patients, and generalized tonic clonic seizures in 40 patients. Aura was documented in 45 patients (45/102, 44.1%) and headache was the most common aura in 26 patients (26/102, 25.5%). There was no differences in gender, seizure type, and presence or absence of aura other than headache but patients with headache as an aura had a significantly younger age of onset of seizures than patients without headache (14.8±3.8 vs 24.7±16.2, p=0.003).

Conclusion: Our study shows that headache is the most frequent aura in patients with generalized seizures. Patients with a younger age of onset of seizures are more likely to experience headache as an aura, which may be due to differences in pathophysiology or cortical neuronal network according to the age of onset of seizures in patients with generalized seizures.

**Fig. 1 (abstract P25).**
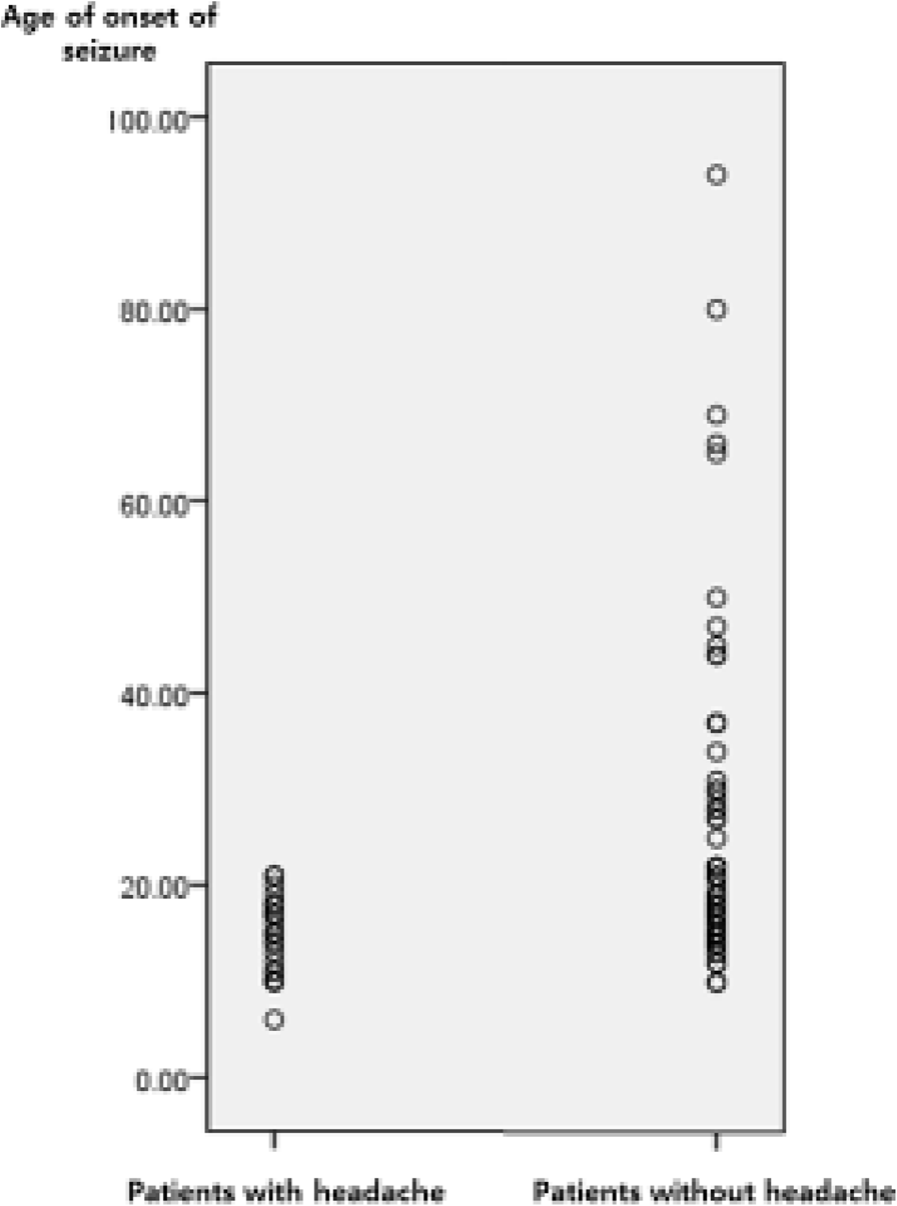
See text for description.

## P26 Headache phenotypes in Idiopathic Intracranial Hypertension and its short-term outcomes

### G. Cabral^1^, M. Serôdio^1^, B. Meira^1^, A. Caetano^1,2^, M. V. Baptista^1,2^

#### ^1^Hospital Egas Moniz, Neurology, Lisbon, Portugal; ^2^Chronic Diseases Research Center (CEDOC) - Nova Medical School, Universidade Nova, Lisbon, Portugal

##### **Correspondence:** G. Cabral

**Question:** Is there any relationship between demographic factors, clinical pattern of headache, treatment response, and headache outcome with the headache phenotype of IIH?

**Methods:** Retrospective analysis of demographic and clinical characteristics of patients with IIH presenting with headache between 01/01/2008-31/12/2021, with the evaluation of headache outcomes in the first 12 months following IIH treatment. Statistical analysis was made using descriptive and non-parametric tests.

**Results**: Headache was present in 32 of 40 patients (80%) with IIH (90% female; mean age 32 years; mean BMI 32,9Kg/m^2^; 88% with papilledema; mean CSF opening pressure 37cmH_2_0). Patients presented commonly with migraine (n=11, 34.4%) and tension-type headache (n=7, 28.1%). A not-classifiable phenotype was present in 12 patients (37.5%). Associated symptoms included photophobia (47%), worsening with Valsalva/recumbency (25%), nausea/vomiting (19%), and phonophobia (9%). 72% of patients had daily pain, but only 9% had a medication-overuse headache; 41% had previous primary headache (mostly migraine). Regarding treatment and short-term follow-up (12months), there was a failure to medical treatment in 44% and a reduction of headaches (≥50%) in 63% of the patients. Among headache phenotypes, there were no significant differences regarding age, race, BMI, or clinical features (symptoms associated with IIH, CSF opening pressure, clinical pattern of headache, time until diagnosis). Also, there were no differences regarding response to treatment or headache outcomes in 12 months follow-up.

**Conclusions:** Headache phenotype does not appear to be an essential factor in allowing clinical distinction, treatment response, or predicting the short-term headache outcome of this intriguing entity.

## P27 Outcome of epidural blood patch for imaging-negative spontaneous intracranial hypotension

### S. Y. Choi^1^, M. Seong^2^, E. Y. Kim^2^, S. Cho^3^, M. Lee^4^

#### ^1^Samsung Medical Center, Neurology, Seoul, South Korea; ^2^Samsung Medical Center, Radiology, Seoul, South Korea; ^3^Uijeongbu Eulji Medical Center, Neurology, Uijeongbu, South Korea; ^4^Seoul National University Hospital, Neurology, Seoul, South Korea

##### **Correspondence:** M. Lee

Background: Spontaneous intracranial hypotension (SIH) is diagnosed based on at least one of abnormal findings in brain MRI, spinal imaging, and lumbar puncture. However, the sensitivity of brain MRI, spinal myelography, and lumbar puncture is low. We questioned if patients with suspected SIH would respond to epidural blood patch (EBP) although they do not have imaging abnormalities.

Methods: We prospectively registered patients with suspected SIH admitted to Samsung Medical Center from 2017 January and 2021 July. For patients whose brain MRI and CT or MR myelography were normal and received EBP for the first time in our hospital, we analyzed their treatment outcome at discharge and 3 months after EBP which was defined as the remission of orthostatic headache and 50% response in maximal headache intensity.

Results: A total of 22 treatment-naïve patients with orthostatic headache and negative brain and spinal imaging who received EBP were identified and included in this study. Spinal imaging was performed with CT myelography in 6 (27%) and MR myelography in 16 (73%). Out of 9 (41%) patients who underwent lumbar puncture, none had an opening pressure lower than normal range (median 13.8 cmH2O, interquartile 9.8 – 16.6). After EBP (mean 1.4 times, range 1–3), orthostatic headache was remitted in 77% and 95% of patients, and 50% response was achieved in 77% and 91% of patients, respectively at discharge and 3 months after treatment.

Conclusion: Our study shows that EBP yielded a high rate of treatment response in imaging-negative patients with suspected SIH. We suggest that the empirical EBP should be considered for the treatment of new onset orthostatic headache although brain and spinal imaging are negative. The necessity of lumbar puncture is questionable in such patients considering the high response rate of EBP and low detection rate of "low pressure".

## P28 Unusual Presentation of Headache Attributed to Airplane Travel - Case Report

### V. Grozeva

#### Private Headache Practice, Sofia, Bulgaria

BACKGROUND: According to ICHD-3 Headache Attributed to Airplane Travel is described as a severe headache, usually unilateral and periocular and without autonomic symptoms, occurring during and caused by aeroplane travel. A recent study reveals some differences in the clinical presentation and suggests a need for refining the criteria. AIM: To describe a case of headache attributed to airplane travel, accompanied by autonomic symptoms and longer duration. CASE PRESENTATION: A 34y woman with severe headache, which developed first on an aeroplane travel during landing and repeats ever since during landing. Pain is of shock-like nature, VAS=10, localized periocularly on the left, accompanied by nausea, vomiting, tearing of the right eye, followed by subsequent tearing of the left one. A discharge of clear secretion from the right nostrail is also present. Headache improves after landing but does not subside. It might contunue 1-3 days with a milder intensity. It is so unpleasant, the patient does not want to travel by plane. Apart from that, a classical clinical characteristics of migraine with visual aura (black dots in the vision field) is reported again on the left side with photo-, phonophobia, and nausea, no other autonomic symptoms, restlessness or aggitation. Patient had concurrent bronchial asthma. Neurological exam-normal. MRI showed small tempo-parietal lesions of a vascular nature; without any sinus pathology. The case most likely refers to both: 1) headache associated with airplane travel and 2) migraine with visual aura. However, the presence of autonomic symptoms is not alligned with the ICHD-3 definition for airplane headache. Additionally, the headache does not remit after landing wich is another discrepancy. The migraine with aura is co-existing. CONCLUSION: Based on the described clinical characteristics, it seems that headache attributed to airplane travel might present also with accompanying autonomic symptoms like lacrimation and rhinorhea. Consent to publish had been obtained.

## P29 Impact of migraine on the presentation of reversible cerebral vasoconstriction syndrome

### K. S. Lange^1,2^, O. Forster^2^, J. Mawet^3^, G. Tuloup^3,4^, C. Burcin^3^, L. Corti^2^, C. Roos^3^, C. Duflos^5^, A. Ducros^2,6^

#### ^1^Charité University Hospital Berlin, Neurology, Berlin, Germany; ^2^University Hospital Montpellier, Neurology, Montpellier, France; ^3^Lariboisière Hospital, APHP Paris, Neurology, Paris, France; ^4^University Hospital Caen-Normandie, Neurology, Caen, France; ^5^University Hospital Montpellier, Clinical Research and Epidemiology Unit, Department of Medical Information, Montpellier, France; ^6^Montpellier University, Charles Coulomb Laboratory, CNRS UMR5221, Montpellier, France

##### **Correspondence:** K. S. Lange

**Question—** Prevalence of migraine among patients with reversible cerebral vasoconstriction syndrome (RCVS) considerably exceeds prevalence in the general population. However, its impact on the clinical and radiological presentation of RCVS remains unknown. We aimed to compare clinical characteristics and complications in RCVS patients with and without a history of migraine.

**Methods—** In a pooled cohort of 345 French patients with RCVS, we compared patients with and without a history of migraine regarding the clinical presentation, rates of neurological complications, and the functional outcome at 3 months.

**Results—** Among 345 patients, 92 (27%) reported a history of migraine. Migraine was independently associated with the absence of thunderclap headache at onset (OR 1.8, 95% CI 1.0-3.3; p=0.049) and with absence of recalled sexual triggers (OR 2.4, 95% CI 1.3-4.7; p=0.008). History of migraine with aura was an independent risk factor for aura during the course of RCVS (OR 6.4, 95% CI 2.0-20.4; p=0.002), while history of migraine without aura was independently associated with the occurrence of subarachnoid hemorrhage (SAH; OR 2.0, 95% CI 1.0-3.7; p=0.037) and multiple cervical artery dissections (mCAD; OR 4.1, 95% CI 1.1-14.6; p=0.032). The functional outcome was equal in both groups, with a modified Rankin scale score of 0-1 in ≥90% of patients.

**Conclusions—** Migraine seems to influence clinico-radiological features of RCVS, predisposing for an atypical clinical presentation and an elevated risk for SAH and mCAD. Larger multi-centric studies are warranted to confirm these findings.

## P30 Extreme intracranial hypertension and severe headache in a immunocompetent man with Cryptococcal meningitis

### F. Farham^1^, A. Naser Moghadasi^2^, H. Marhamati^1^

#### ^1^Tehran University of Medical Sciences, Headache Department, Tehran, Iran; ^2^Tehran University of Medical Sciences, Multiple Sclerosis Research Center, Tehran, Iran

##### **Correspondence:** F. Farham

Cryptococcusis is an infection caused by the fungi Cryptococcus neoformans and gatii.^1^ Cryptococcal meningitis is generally occurred in immunocompromised patients. It may be seen rarely in immunocompetent individuals with nonspecific and more subtle symptoms.^2^

case: A 47 Years old man with history of 6 months headache, diplopia and blurred vision Presented to emergency department because of the worsening of his symptoms. The Headache was severe, generalized with nausea, the characteristics were dullness and postural that the patient could not lay down. Examination was revealed a papillary edema, and decreased visual acuity.

Magnetic resonance imaging of brain showed bilateral basal ganglia signal change. Lumbar puncture was done, and the opening pressure was 120 cmH2O. The analysis results were as follow: Appearance semi-clear, white blood cell: 130 (80% lymphocyte), red blood cell: 580, and the CSF biochemistry results were: Glucose: 28 mg/dl and total protein: 59 mg/dl. The study of CSF showed a positive Cryptococcus neoformans PCR. The patient admitted and treatment with Amphotericin B was started. HIV Ab was negative and patient"s immune system was intact. He just had close-contact with pigeon. After 30 days of treatment, Cryptococcus neoformans PCR was negative in secound SCF study. Patient discharged with oral Fluconazole and the headache was completely resolved but the ophtalmic symptoms had partially improved.

Cryptococcusis Should be considered in the differential diagnosis of immunocompetent patients, presenting with prolonged headache.^2^

Early diagnosis is important, because late treatment may lead to some residual symptoms and sometimes death of patients. Consent to publish had been obtained.

1. Cryptococcal meningitis: epidemiology, immunology, diagnosis and therapy. P.R. Williamson, et al. Nat Rev Neural, 13(2017)

2. Cryptococcus meningitis presented with multiple cerebral infarcts in an immunocompetent patient. Buket Erturk Sengel, et al. IDCases,vol.24(2021)

**Fig. 1 (abstract P30).**
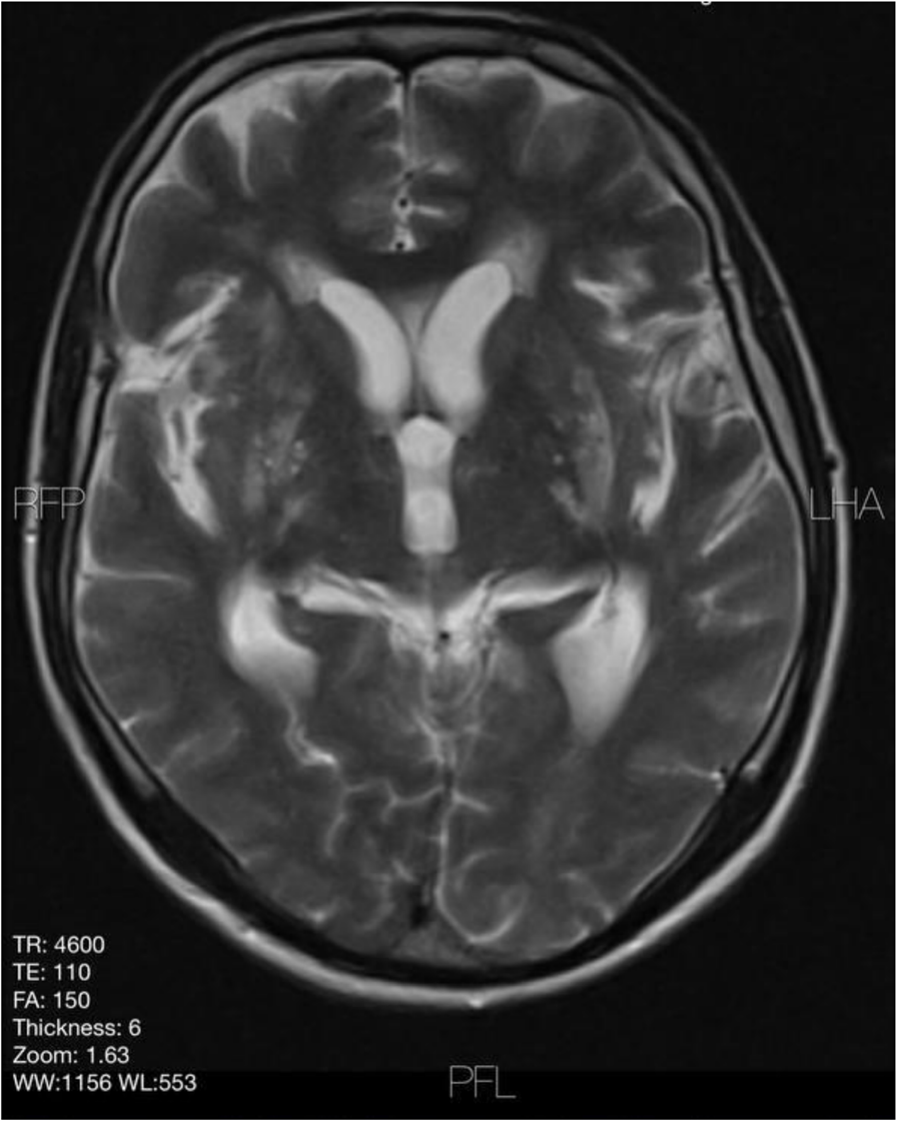
See text for description.

**Fig. 2 (abstract P30).**
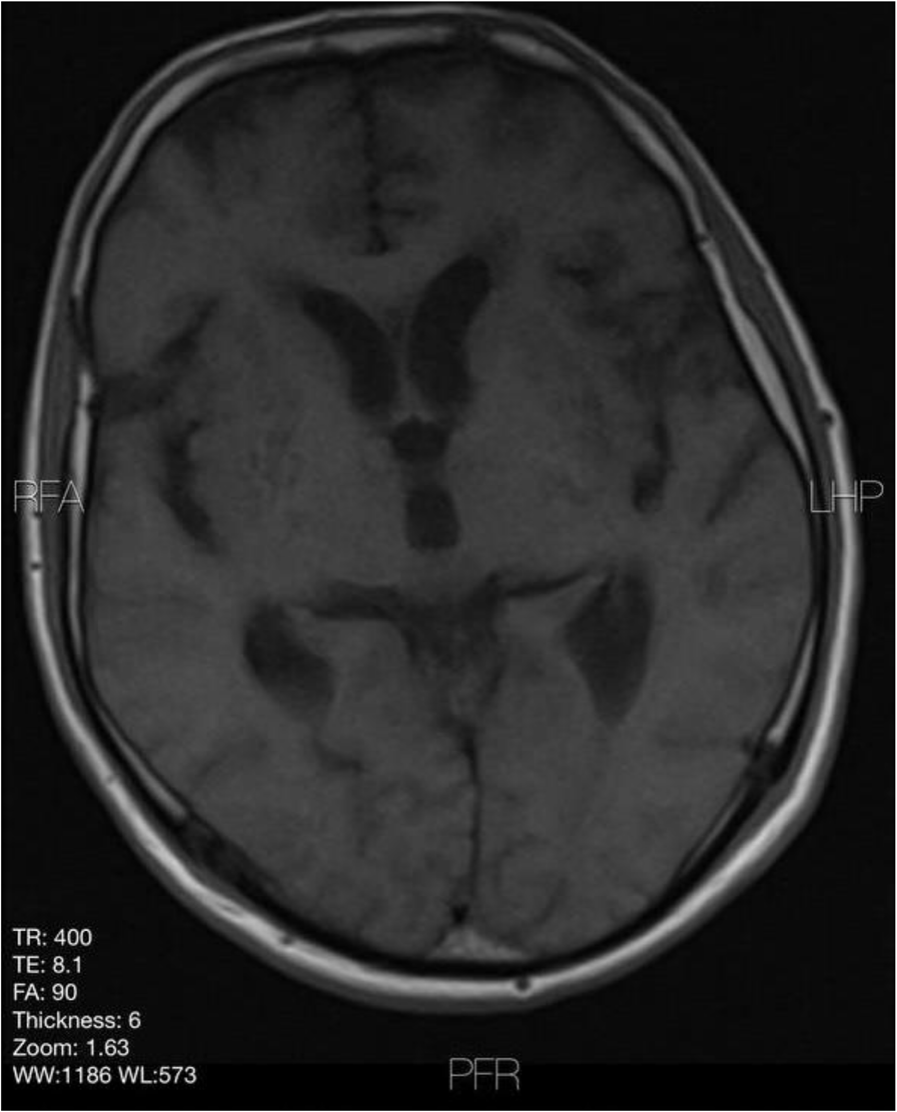
See text for description.

## P31 The features of the course of traumatic brain disease in persons with combat traumatic brain injury

### I. Chernenko

#### Kharkov National University, Neurology, Psychiatry, Narcology, Kharkiv, Ukraine

This article is devoted to the problem of survival and prognosis of treatment outcomes of patients with traumatic brain injury who have sustained craniocerebral trauma according to changes in neurospecificity protein (S100β) levels during the acute period of trauma. An analysis of the course and results of treatment of patients who had sustained severe craniocerebral trauma was carried out. Patients underwent routine biochemical examinations, neuroimaging studies and protein (S100β) levels examination in the acute period of trauma. On the basis of the data obtained, it was found that the level of neurospecificity protein S100β in the blood serum takes a great role in predicting the course and outcome of the disease. Objective: to elucidate the relationship between changes in the level of neurospecific protein (S100β) in the acute period of injury, data from neuroimaging methods (CT and / or MRI) of the study, the course and results of treatment and consequences of traumatic brain injury in combat zone Environmental protection in the East of Ukraine on the basis of the analysis of retrospective research (injury follow-up was 1 year, 3 years, 5 years). Materials and methods of research. 250 participants of hostilities and invalids, after the received craniocerebral trauma in the zone of carrying out anti-terrorist operation, on the basis of neurological department of Regional hospital of war veterans were observed. Patients were divided into groups depending on the severity of the injury (mild, moderate and severe), treatment in the acute period, the course and outcome of the disease. Observations have been conducted since 2015. by 2021, the follow-up was 1 year, 3 years, 5 years. Conclusions. Based on the data we conducted on the basis of the Hospital retrospective analysis of medical documents of the military who received severe trauma in the area of anti-terrorist operation, we can say that the level of neuronspecific protein S100β in serum plays an important role in predicting the course and outcome of the disease.

## P33 Epicrania fugax secondary to multiple sclerosis

### M. D. Calabria Gallego

#### Hospital Universitario de Salamanca, Neurology, Salamanca, Spain

**Background**

Epicrania fugax (EF) is a primary headache of recent description. EF essentially consists of brief paroxysms of pain describing a linear or zigzag trajectory across the surface of one hemicranium, beginning and terminating in the territories of different nerves (1).

Materials and methods

We present a case report that illustrates the possibility of presenting a headache with the characteristics of EF, but which is presumably produced secondary to demyelination plaques due to multiple sclerosis.

**Results**

Patient with a history of multiple sclerosis receiving treatment with glatiramer acetate, with periventricular, subcortical and infratentorial white matter lesions (Figure 1), who reports that for 6 days he has presented pain in the territory of the three branches of the right trigeminal nerve of short duration and electrical characteristic, which is becoming more frequent and longer (4-5 seconds). There is no trigger point. By expressly delimiting the area, he refers that it begins at the level of the vertex, with a rapid path towards the chin. Normal neurological examination.

With these data, a forward EF diagnosis is made. Anesthetic blockade with bupivacaine was performed on both major suboccipital nerves and Lamotrigine 75 mg/day was prescribed (with progressive dose escalation over six weeks).

At the check-up after three months, the patient reports the complete resolution of his symptoms

**Conclusions**

The correct characterization of a secondary headache can improve our management. In the previous case, an anesthetic block is performed and lamotrigine is prescribed. Despite the scant evidence collected to date, this management would be correct for a primary EF, and in the same way it has been useful for the patient. Consent to publish had been obtained.

References

1. Square ML, Guerrero AL, Couple JA. Epicrania Fugax. Curr Pain Headache Rep. 2016 Apr;20(4):21. doi: 10.1007/s11916-016-0557-9. PMID: 26893151.

**Fig. 1 (abstract P33).**
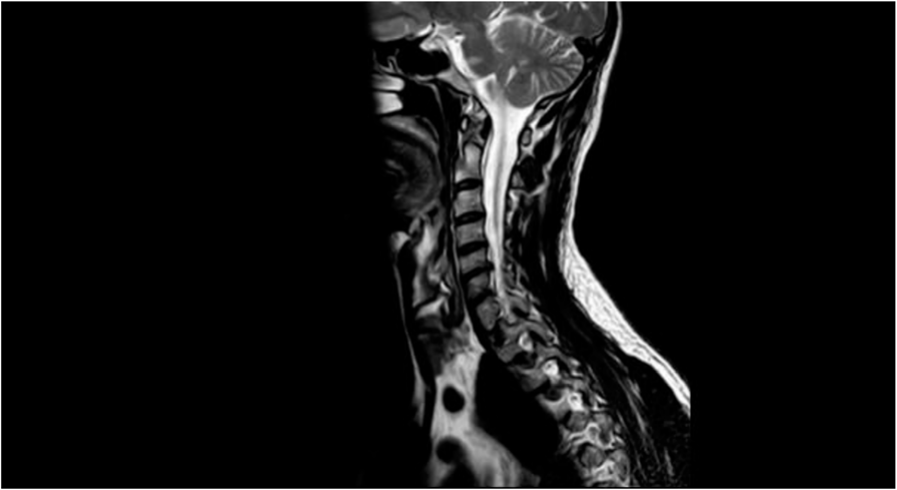
See text for description.

## P34 A Case of Spontaneous Intracranial Hypotension in a Third-trimester Pregnant Woman Resolved by Delivery

### H. S. Lee, H. B. Yang

#### Ansan Hospital Korea University, Neurology, Ansan-si, South Korea

##### **Correspondence:** H. S. Lee

Spontaneous intracranial hypotension (SIH) is characterized by a low cerebrospinal fluid (CSF) volume because of leakage, resulting in an orthostatic headache.1 Little is known about SIH during pregnancy, especially in a third-trimester is very rarely reported.2 We report a SIH case in a third-trimester pregnant woman, which was resolved by delivery. This is the first report to our knowledge.

A 30-year-old woman at 33+6 weeks" gestation presented to our hospital with a headache. She had felt a tearing pain in the upper back while mopping 4 days ago, then an orthostatic headache occurred. Diffuse pachymeningeal thickening/enhancement and fluid collection near the dura showed on brain and spine MRI. (Fig. 1 & 2) We diagnosed her with SIH clinically. She had been treated with bed rest, hydration, and analgesics, but did not improve. We inquired obstetricians about fetal safety for the CT-guided epidural blood patch (EBP), and they recommended a procedure after delivery because the fetus was mature. She had a cesarean section.

The headache rapidly improved after the delivery. On the third day after delivery, the patient could stand for more than an hour. She was discharged on the fifth day after delivery without a headache.

Although the risk or physiologic factors for SIH during pregnancy are unknown, the point that SIH was resolved through delivery in this case suggests that SIH might be related to pregnancy itself. If the symptom does not improve with known treatment, delivery might be considered as the next option for treatment when the fetus matures allow delivery. Consent to publish had been obtained.

1. Mokri B. Spontaneous intracranial hypotension. *Continuum*.2015;21:1086-108

2. Ferrante E, et al. Management of Spontaneous Intracranial Hypotension During Pregnancy: A Case Series*.Headache.*2020;60:1777-87

**Fig. 1 (abstract P34).**
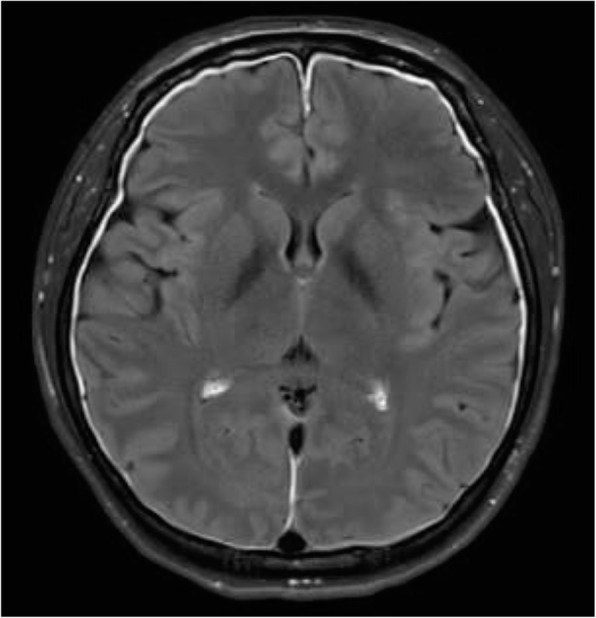
Brain MRI show diffuse pachymeningeal thickening and enhancement

**Fig. 2 (abstract P34).**
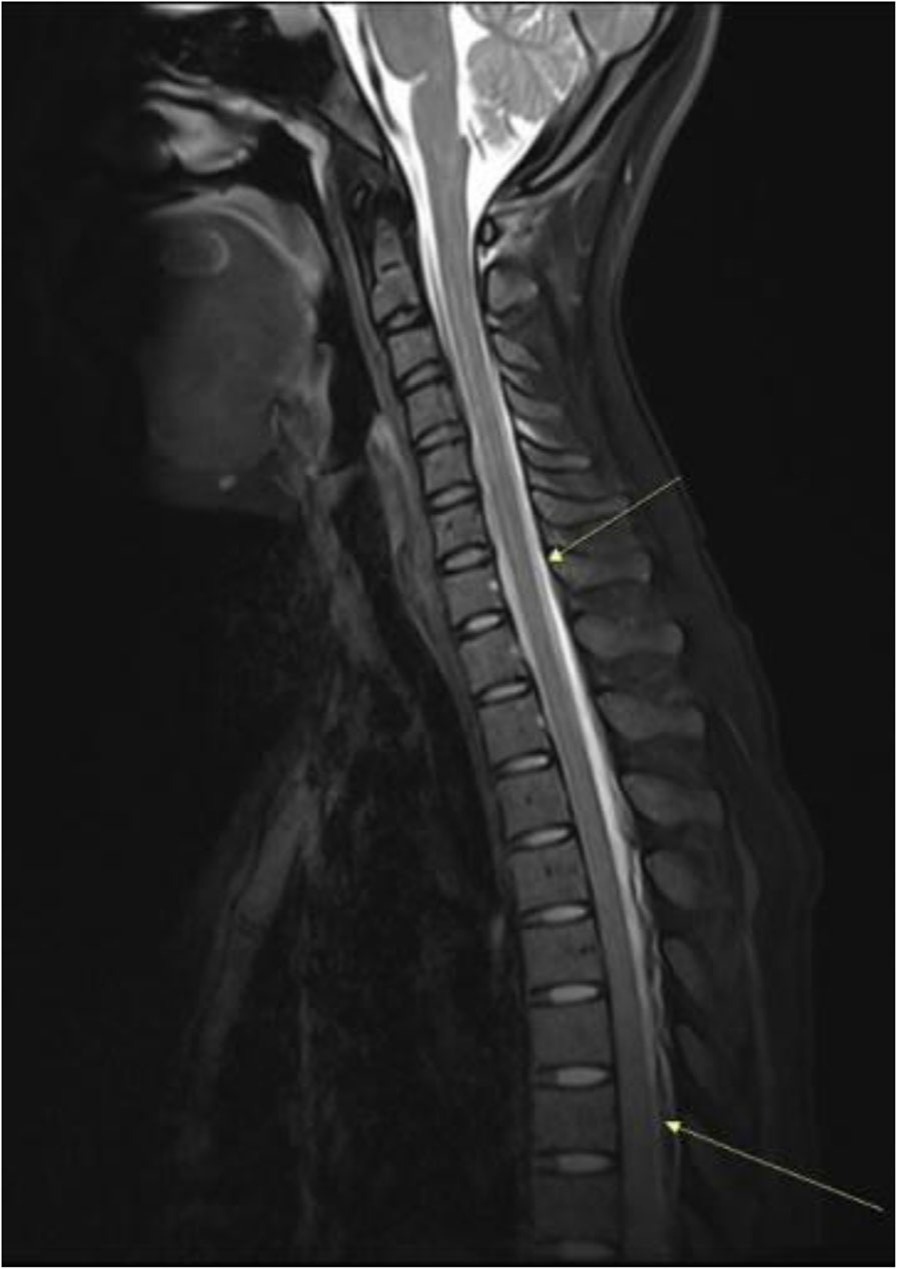
Spine MRI show diffuse fluid collection in the epi and subdural space, posterior to the C7-T7 cord. (arrows)

## P35 Post-traumatic headache responds well to suboccipital block

### M. D. Calabria Gallego

#### Hospital Universitario de Salamanca, Neurology, Salamanca, Spain

**Background:**

Post-traumatic headache, defined as the one that occurs after a traumatic brain injury, can adopt the characteristics of other primary headaches (especially tension-type headache or migraine), and traditionally its management has been that of these headaches. This clinical management lacks solid scientific evidence and is rather based on expert opinions (1).

**Materials and methods:**

Through the exposure of two cases reports, treatment by anesthetic blocks is suggested as a useful therapeutic tool for these cases.

**Results:**

The first patient is a 73-year-old woman, who reported that after a mild head injury, she had intermittent headache of varying intensity, oppressive type, of greater intensity at times of greater psychological stress, and of left parieto-occipital location. Normal examination and CT.

Subcutaneous infiltration with bupivacaine is performed in both major suboccipital and supraorbital nerves, and amitriptyline is prescribed. The patient only consumed one container of amitriptyline by mistake, presenting complete resolution of her symptoms since the blockade was performed.

Secondly, we have a 70-year-old man who had a pulsating right hemicranial headache for 30 years, at which time he suffered a trauma in that area. The headache, in the last months had been accentuated. Normal examination and CT.

Amitriptyline was prescribed, and after two months, the patient reported only a slight improvement, proceeding to perform a bupivacaine blockade of both major suboccipital nerves, giving complete resolution of the condition.

**Conclusion:**

Anesthetic blocks have been positioned as a therapeutic weapon in some types of headaches (migraine, cluster headache, suboccipital neuralgia,...), as we believe that in post-traumatic headache may be. Consent to publish had been obtained.

1. Ashina H, Eigenbrodt AK, Seifert T, *et al*. Post-traumatic headache attributed to traumatic brain injury: classification, clinical characteristics, and treatment. Lancet Neurol. 2021 Jun;20(6):460-469.

## P36 Headache in intracranial arachnoid cysts: implications for management

### J. Carbone

#### WA Health, Neurosurgery, Nedlands, Australia

Question:

With a prevalence of 1.4%, intracranial arachnoid cysts are a frequent incidental finding on MRI and CT. Whilst most cysts are benign in the long term, clinical practice and imaging frequency does not necessarily reflect this. Is headache a useful symptom in stratification of this pathology?

Method:

A literature review was conducted searching the Medline database with MESH terms. This literature was condensed into an article, edited by a consultant Neurosurgeon. This was further condensed, presented to the Neurosurgery department at Princess Alexandra Hospital for final feedback and editing.

Results:

Headache remains a non specific symptom in relation to cysts, however case reports do exist of post traumatic bleed and spontaneous cyst growth. The minority of symptomatic patients or those with cysts in sensitive areas may require referral to a neurosurgeon for clinical follow up, imaging or intervention. This review outlines a treatment algorithm to guide clinicians.

Conclusion:

Greater than 94% of patients are asymptomatic, practitioners can be confident in reassuring patients of the benign nature of a potentially worrying finding. Headaches should be thoroughly explored and cysts in atypical locations or with atypical features may benefit from surgical intervention.

**Fig. 1 (abstract P36).**
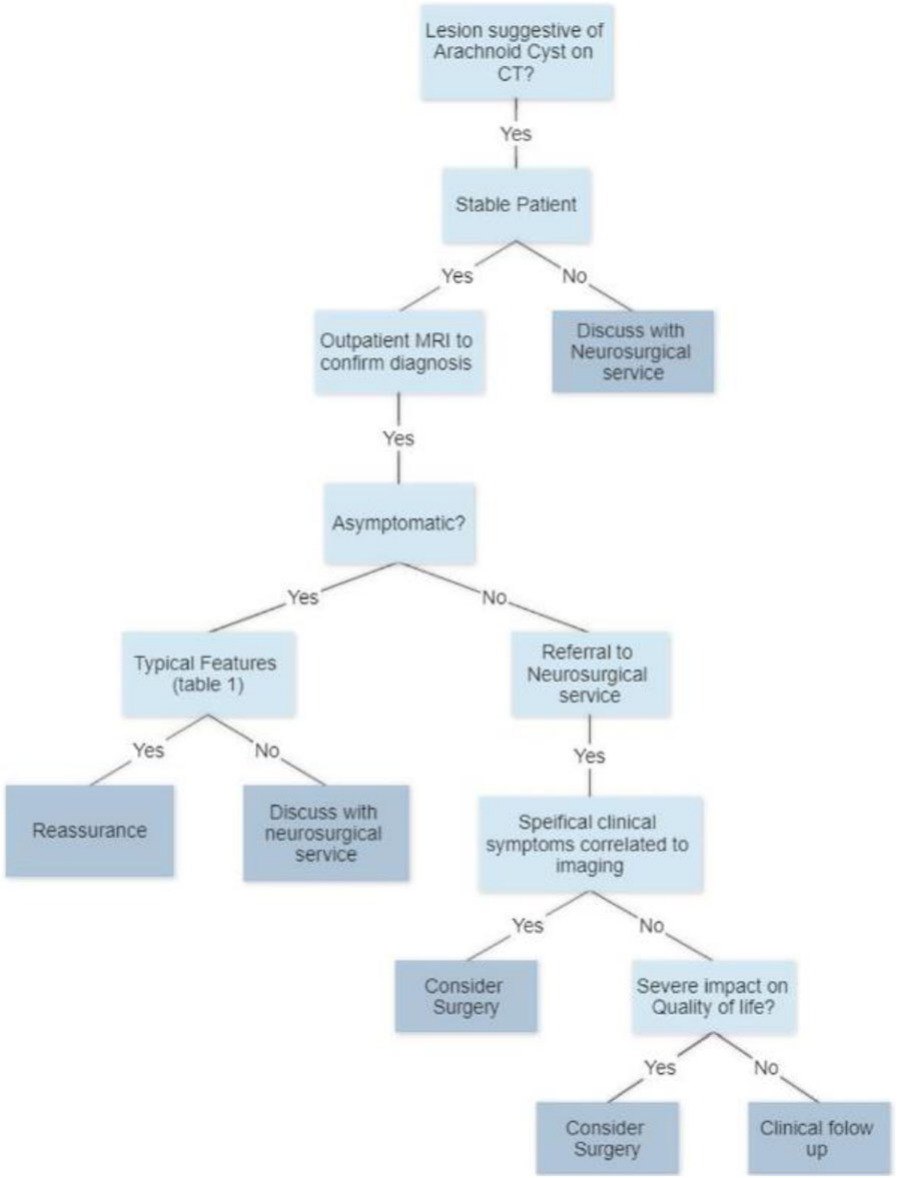
See text for description

**Fig. 2 (abstract P36).**
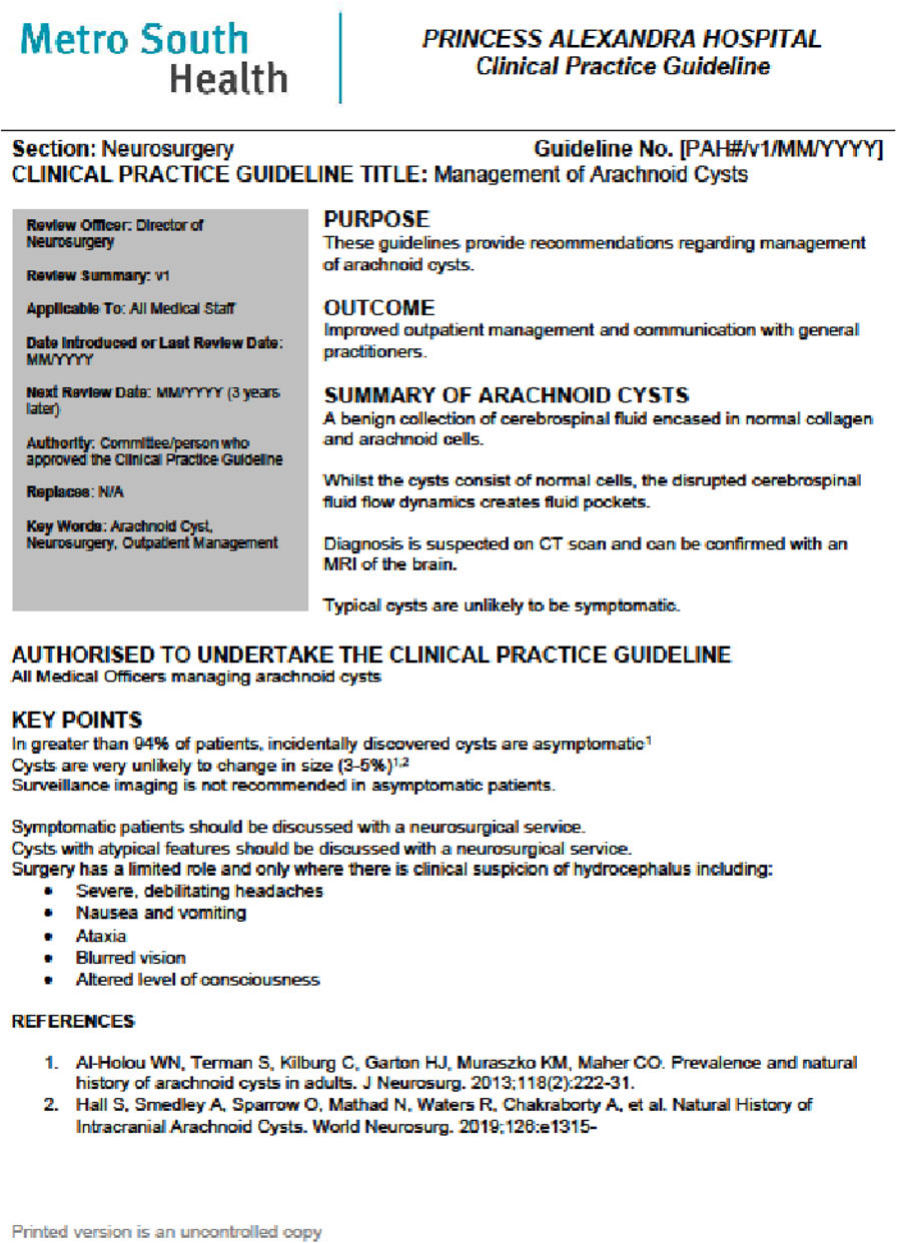
See text for description

## P37 Low adherence to the guideline for the acute treatment of migraine

### A. Olesen ^1^, H. W. Schytz^1^, S. R. Ostrowski^2^, M. Topholm^3^, K. Nielsen^4^, C. Erikstrup^5^, S. Mikkelsen^5^, O. B. Pedersen^6^, J. Olesen^1^, T. F. Hansen^1^, M. A. Chalmer^1^

#### ^1^Rigshospitalet , Danish Headache Center, Neurological Department , Glostrup, Denmark; ^2^Rigshospitalet , Department of Clinical Immunology, Copenhagen, Denmark; ^3^Odense University Hospital, Department of Clinical Immunology, Odense, Denmark; ^4^Aalborg University Hospital, Department of Clinical Immunology, Aalborg, Denmark; ^5^Aarhus University Hospital, Department of Clinical Immunology, Aarhus, Denmark; ^6^Zealand University Hospital, Department of Clinical Immunology, Koege, Germany

##### **Correspondence:** M. A. Chalmer

Introduction: The real-world use of triptans in the treatment of migraine is disappointing. Only 12% of the Danish migraine population purchased a triptan between 2014 and 2019, and only 43% repurchased a triptan after first prescription. The aim of the present study was to assess whether physicians and patients adhere to the therapeutic guideline on acute migraine treatment.

Methods: We interviewed 299 triptan experienced participants with migraine and 101 triptan naïve participants with migraine from the Danish Migraine Population Cohort, using a semi-structured questionnaire. Descriptive statistical analyses were used to study the association with triptan use and the assessed factors.

Results: Among triptan naïve participants with migraine, 64% had consulted their general practitioner about their migraine, of whom only 23% received information about the possibility of triptan treatment. Among triptan experienced participants, 77% had only tried one type of triptan. Only 12% could recall they had been informed by their general practitioner to try each triptan three times before giving up. Twenty percent were informed to try three different triptans in total, if the first did not work. In disagreement with the guideline, participants who reported a low pain reduction by a triptan had only tried one type of triptan.

Conclusion: Our study shows a low adherence to therapeutic guideline for the attack treatment of migraine. There is a need for better education of general practitioners regarding treatment of migraine. Future campaigns should aim to inform both the public and the general practitioner about antimigraine treatments.

## P38 Characterization of adult patients with status migrainosus in a tertiary hospital in Colombia, 2019-2021

### R. Lopez-Gonzalez^1,2,3^, S. Isaza-Jaramillo^1^, M. Ashina^4^

#### ^1^University of Antioquia, Neurology, Medellin, Colombia; ^2^Clinica El Rosario, Neurology, Medellin, Colombia; ^3^Hospital San Vicente Fundacion, Neurology, Medellin, Colombia; ^4^Danish headache center, Rigshospitalet Glostrup, Faculty of Health and Medical Sciences, University of Copenhagen, Department of Neurology, Copenhagen, Denmark

##### **Correspondence:** R. Lopez-Gonzalez

**Objective:** To describe demographic and clinical characteristics, treatment strategies, use of diagnostic tests, evolution in hospitalization and readmissions of patients with status migrainosus (SM) who required inpatient management.

**Methods:** Retrospective and observational study analysing patients who presented SM between 2019 and 2021 at a tertiary hospital in Colombia.

**Results:** We identified 170 SM in 135 subjects. 91% were women with a median age of 34 years. 79,9% had migraine without aura. 79,9% had episodic migraine. 22,9% were taken prophylactic medication. The median duration of headache before admission to emergency department (ED) was 120 hours. 96,5% with level pain ≥7/10. Pulsatile headache (86,5%), worsening of pain with physical activity (87,1%), photophobia (94,7%) and nausea (90%) were the most common symptoms. Pregnancy was present in 8,9%. It was more probable that pregnant women received opioids for their treatment (p=0,0002). 18,2% had prior medication overuse; subjects with medication overuse were less probable to improve with the first line of treatment at ED (p=0.02). Only 34,1% of SM improved with treatment at ED. 81% of SM who improved at ED required at least three medications. Improvement with treatment at ED was less probable if subjects had received opioids before admission (p=0,002). All participants treated by neurologists received a combination of drugs. 52% of them required at least 2 lines of treatment. The most used medications by neurology were magnesium sulfate, ketorolac and triptans. 68,2% received at least one diagnostic test and 94,8% of them were normal. Median length of hospital stay was 1,96 days. 69,4% were discharged pain-free. 12,3% were readmitted to ED due to headache within the next week.

**Conclusion:** SM is a disabling condition. SM requires to be treated with drug combinations with synergic mechanisms of action, which can lead to freedom of pain in most patients. Opioids should be avoided in the treatment of SM.

**Fig. 1 (abstract P38).**
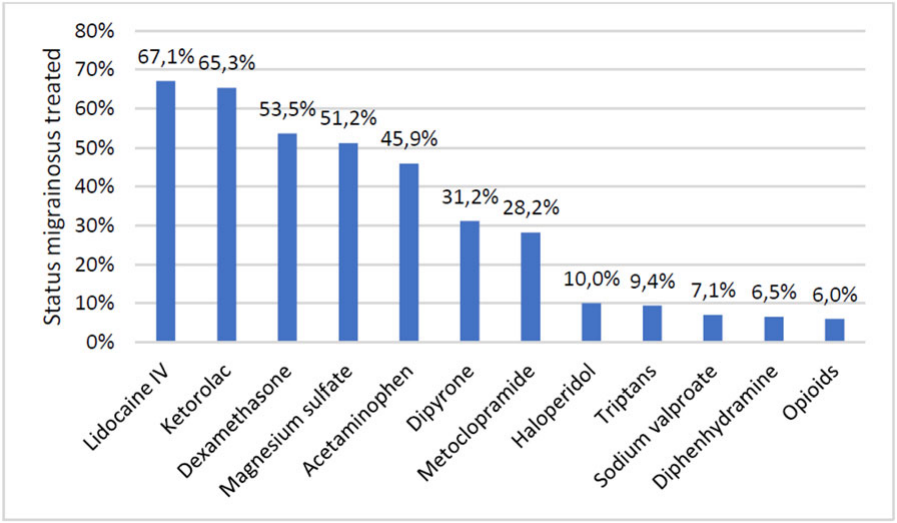
See text for description.

**Fig. 2 (abstract P38).**
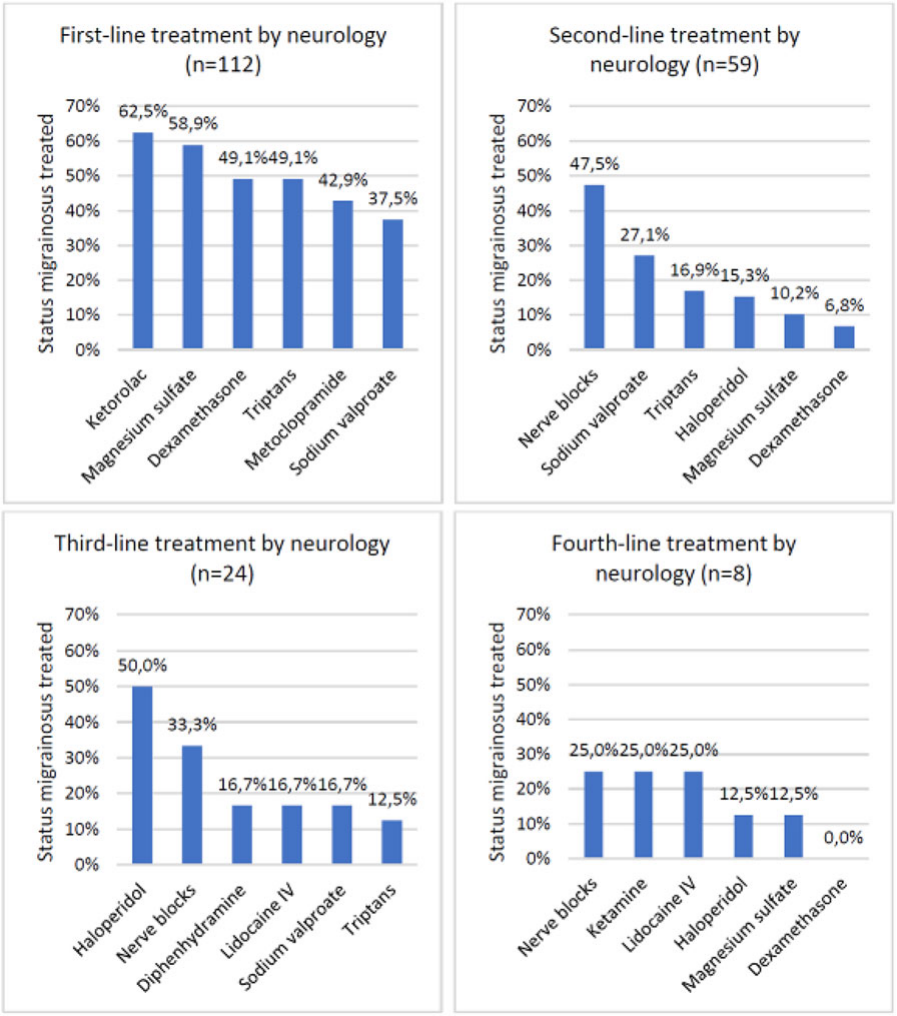
See text for description.

## P39 Implementing a digital treatment solution for headache patients – a pilot study

### T. Niiberg-Pikksööt^1,2^, K. Laas^3^, A. Aluoja^4^, M. Braschinsky^1,2,5^

#### ^1^Tartu University Hospital, Headache Clinic, Department of Neurology, Tartu, Estonia; ^2^Migrevention OÜ, Tallinn, Estonia; ^3^University of Tartu, Institute of Psychology, Tartu, Estonia; ^4^Tartu University Hospital, Psychiatry Clinic, Tartu, Estonia; ^5^University of Tartu, Neurology Clinic, Tartu, Estonia

##### **Correspondence:** T. Niiberg-Pikksööt

**Background**. Migraine is the leading cause of disability worldwide, affecting primarily working-age population. Poor availability of non-pharma cological treatment options and shortage of specialists is a major problem on a global scale. The goal of this pilot study was to create a model that would allow for comparisons between conventional and digitally mediated care while evaluating the cost-effectiveness of digitally mediated care.

**Question**. Is it possible to conduct manual-based digitally mediated non-pharmacological treatment in a way that is comparable to conventional treatment?

**Methods**. The pilot study was approved by the Research Ethics Committee of the University of Tartu. Two groups of patients (n=10 for conventional treatment and n=10 for digitally mediated treatment) participated in an intense 8-week non-pharmacological interdisciplinary treatment program. The patient received nurse counselling, cognitive-behavioural therapy, and physical therapy as part of their treatment. The number of headache days, headache's influence on everyday life, changes in quality of life, anxiety and depression levels were measured. The level of patient satisfaction with the intervention and the amount of work time required by experts were examined.

**Results.** There was no change in the number of days with headaches. In both groups, satisfaction with the intervention was extremely high. Indicators of quality of life improved in both groups. Ten times less time was spent on digitally mediated treatment by specialists.

**Conclusions**. The developed non-pharmacological intervention program and manual-based intervention are appropriate for a wider scale trial. Digitally mediated treatment is just as effective as conventional treatment but permits 10 times as many individuals to be treated without sacrificing quality. There is a need for larger-scale research to demonstrate with greater precision the influence on patients' lives and the cost-effectiveness of the intervention strategy.

## P40 PACAP signaling is not involved in GTN- and levcromakalim-induced hypersensitivity in mouse models of migraine

### S. Guo^1,2^, C. Ernstsen^1^, A. Hay-Schmidt^2^, M. Ashina^1^, J. Olesen^1^, S. S. Christensen^1^

#### ^1^Danish Headache Center, Neurology, Copenhagen, Denmark; ^2^University of Copenhagen, Department of Odontology, Panum Institute, Faculty of Health, Copenhagen, Denmark

##### **Correspondence:** S. Guo

*Question:* Calcitonin gene-related peptide (CGRP) antagonizing drugs represents the most important advance in migraine therapy for decades. However, these new drugs are only effective in 50-60% of patients. Recent studies have shown that the pituitary adenylate cyclase-activating peptide (PACAP38) pathway is independent from the CGRP signaling pathway. Here, we investigate PACAP38 signaling pathways in relation to glyceryl trinitrate (GTN), levcromakalim and sumatriptan.

*Methods: In vivo* mouse models of PACAP38-, GTN-, and levcromakalim-induced migraine were applied using tactile sensitivity to von Frey filaments as measuring readout. Signaling pathways involved in the three models were dissected using PACAP-inhibiting antibodies (mAbs) and sumatriptan.

*Results:* We showed that PACAP mAbs block PACAP38 induced hypersensitivity, but not via signaling pathways involved in GTN and levcromakalim. Also, sumatriptan has no effect on PACAP38-induced hypersensitivity relevant to migraine. This is the first study testing the effect of a PACAP-inhibiting drug on GTN- and levcromakalim-induced hypersensitivity.

*Conclusions:* Based on the findings in our mouse model of migraine using migraine-inducing compounds and anti-migraine drugs, we suggest that PACAP acts via a distinct pathway. Using PACAP38 antagonism may be a novel therapeutic target of interest in a subgroup of migraine patients who do not respond to existing therapies.

## P41 Effect of Topical Clonazepam on the Burning Mouth Syndrome Patients' Functional Connectivity

### H. L. Tan^1,2^, J. Hoffmann^3^, T. Renton^1^, H. Matthew^4^, E. Makovac^4^

#### ^1^King's College London, Center for Oral, Cranial & Translational Sciences, London, United Kingdom; ^2^The National University of Malaysia, Faculty of Dentistry, Kuala Lumpur, Malaysia; ^3^King's College London, Wolfson Centre for Age-Related Diseases, Institute of Psychiatry, Psychology & Neuroscience, London, United Kingdom; ^4^King's College London, Department of Neuroimaging, London, United Kingdom

##### **Correspondence:** H. L. Tan

**Objectives:** Burning mouth syndrome (BMS) is an idiopathic and debilitating burning sensation of the oral mucosa. BMS treatment remains a challenge due to its uncertain aetiopathophysiology, but reports have implied that BMS is a central neuropathic pain disorder. We hypothesised that BMS patients' functional connectivity (FC) was modulated by pain intensity following clonazepam mouthwash (MW) and the difference between treatment responders and non-responders.

**Methods:** 26 BMS patients underwent two sessions. In session 1, they received clinical and neuropsychological assessments. In session 2, pain scores (NRS, 0-10) and resting-state functional MRI scans were acquired before and after mouthwash. Seed-based FC analysis of the right anterior insula (RAI) cortex was performed (pFWE corrected <0.05), given reports of perturbed functioning in this region in chronic pain. Treatment responders were defined as reporting 50% or greater pain reduction from baseline following clonazepam administration.

**Results:** After clonazepam, BMS patients experienced a mean NRS reduction of 2.67 (SD ±2.23), and 15 patients responded to treatment. We observed a decrease of post-MW FC across BMS patients, between the RAI and anterior cerebellum and inferior parietal lobe. At baseline, responders showed lower FC than non-responder between RAI, lateral occipital cortex and parietal lobe. After mouthwash responder showed greater FC network changes (∆FC) than the non-responder between RAI and frontal orbital cortex, frontal medial cortex and paracingulate gyrus but a lesser ∆FC between RAI and prefrontal cortex (Figure 1).

**Conclusion:** This study provides a preliminary insight into the anti-nociceptive mechanism of action of topical clonazepam on brain networks. We demonstrated FC changes between RAI and brain regions involved in pain modulation, which may reflect BMS's ongoing pain symptoms and a valuable marker of treatment response.

**Fig. 1 (abstract P41).**
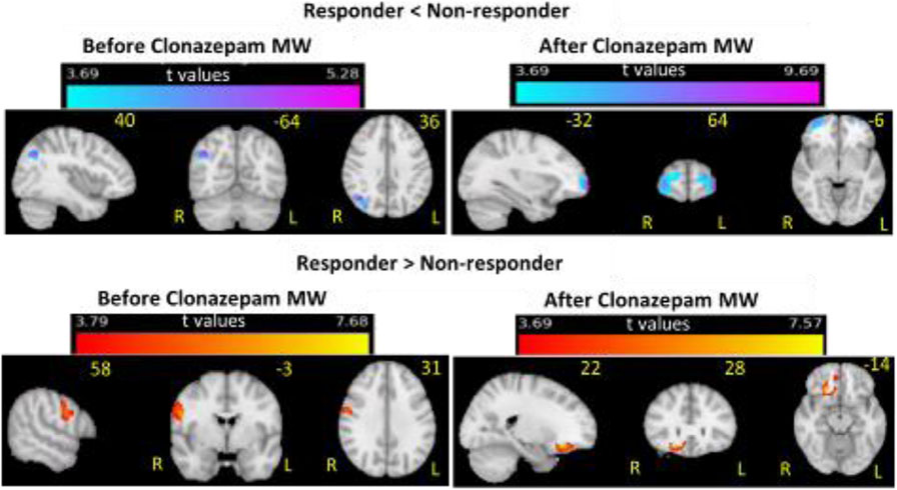
Differences in functional connectivity (FC) between the responder and the non-responder groups of BMS patients at before and after clonazepam mouthwash (MW)

## P42 Prevalence of temporomandibular disorders with comorbid migraine: A retrospective study in a 5-year period

### P. Yakkaphan

#### King's College London, LONDON, United Kingdom

Introduction: Temporomandibular disorders (TMD) is complex and associated with the burden of chronic pain pathologies. Migraine has become one of those constantly found among TMD patients. An association between TMD and migraine has been published. However, no study has reported TMD prevalence according to migraine pain distribution of the somatosensory of the trigeminal nerve (V1, V2, V3). Aim: To investigate the prevalence of TMD with comorbid migraine in Orofacial Pain Clinic, Dental Institute, King's College London Hospital during 2016-2021. Method: The dataset was reviewed for patients diagnosed with TMD during consultation at the OFP Clinic at KCH between January 2016 and December 2021. Data were described by simple statistics, including numbers and percentages. Result: Over five years, we collected data from 1,345 OFP patients. Among 421 TMD patients, 28,26% (n=119) of them presented with comorbid migraine. The majority (94%) had chronic TMD symptoms (duration > 3 months). Myofascial pain was the most prevalent (54.62%), followed by TMD pain due to arthrogenous origin (26.89%). Among TMD patients with comorbid migraine, 60.50% were diagnosed with chronic migraine. Of the TMD patients with migraine, 66.39% suffered from migraine headache (V1 area only), 26.89% suffered from migraine with facial involvement (V1 with V2 and/or V3 area), and 6.72% suffered from orofacial migraine (V2 and/or V3 area). The sole migraine patients were also included in our dataset. Among 242 migraine patients, TMD was found in 57.24% of patients with migraine headache (V1 area only), in 49.23% of patients with migraine with facial involvement (V1 with V2 and/or V3 area), and in 20.51% of patients with orofacial migraine (V2 and/or V3 area). Conclusion: TMD and migraines might commonly occur, especially in individuals with muscle-related chronic TMD and chronic migraine. Although TMD is mostly related to migraine pain in the V1 area, the proportion of TMD with migraine patients reporting pain in the facial region (V2 and/or V3) was not relatively small. Therefore, clinicians should be aware of the presence of migraine headache and orofacial migraine in TMD patients.

## P43 Autonomic Nervous System Disorders in Patients with Burning Mouth Syndrome

### H. L. Tan^1,2^, J. Hoffmann^3^, T. Renton^1^, O. O'Daly^4^, H. Matthew^4^, E. Makovac^4^

#### ^1^King's College London, Center for Oral, Cranial & Translational Sciences, London, United Kingdom; ^2^The National University of Malaysia, Faculty of Dentistry, Kuala Lumpur, Malaysia; ^3^King's College London, Wolfson Centre for Age-Related Diseases, Institute of Psychiatry, Psychology & Neuroscience, London, United Kingdom; ^4^King's College London, Department of Neuroimaging, London, United Kingdom

##### **Correspondence:** H. L. Tan

**Objectives:** Burning mouth syndrome (BMS) is a chronic idiopathic orofacial pain with poorly understood aetiopathogenesis, making treatment challenging. Studies have highlighted central and autonomic nervous system (ANS) dysregulations in BMS, yet studies investigating the interaction between ANS and brain are lacking. The periaqueductal grey area (PAG) has a crucial role in mediating the relationship between ANS and pain, and changes in PAG have been described in other chronic pain conditions. We combined Heart Rate Variability (HRV) as a measure reflective of ANS function, resting-state functional MRI and voxel-based morphometric analysis of brain structure to explore the interaction between ANS and brain mechanisms underpinning BMS pain experiences, focusing on the role of PAG.

**Methods:** 26 BMS patients were assessed in two sessions. In session 1, resting heart rate (HR) was measured for 5 minutes. In session 2, structural and functional MRI scans were acquired. The root mean square of successive differences (a measure of vagal-mediated HRV; RMSSD) was extracted from HR inter-beat. Associations between seed-based PAG functional connectivity (FC) and the interaction between RMSSD and pain scores were investigated (pFWE corrected <0.05). Patients were divided into low and high RMSSD groups for further analysis.

**Results:** The mean pain score (NRS 0-10) was 5.5 (SD ±1.42). Patients with lower RMSSD had higher pain scores in session 1 (p=0.009). RMSSD was positively associated with FC between the PAG and insula and negatively associated with insula grey matter volume. The association between RMSSD and pain was positively mediated by the strength of FC between PAG and thalamus. (Figure 1).

**Conclusion:** BMS brain structure and function changes are associated with parasympathetic tone and perceived pain intensity. These complex relationships provide indications of linkages between the brain and ANS, which may be insightful for developing future therapeutic interventions.

**Fig. 1 (abstract P43).**
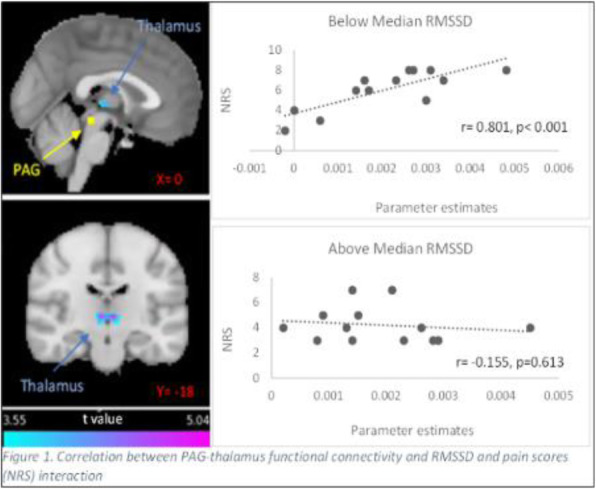
Correlation between PAG-thalamus functional connectivity and RMSSD and pain scores (NRS) interaction

## P44 Two physiotherapy programs in headache attributed to temporomandibular disorder

### P. Moleirinho-Alves^1,2^, P. Cebola^1^, R. Oliveira^2^, P. Pezarat-Correia^2^

#### ^1^Cuf Tejo Hospital, Orofacial pain and Temporomandibular disorders department, Lisbon, Portugal; ^2^Faculty of Human Kinetics, University of Lisbon, Lisbon, Portugal

##### **Correspondence:** P. Moleirinho-Alves

**Objective:** Assess the effects of two 8-week physiotherapy programs on frequency, intensity, and impact of headaches attributed to temporomandibular disorder (TMD). **Methods:** Twenty-four patients diagnosed with headache attributed to TMD were divided into two groups of 12 participants: a therapeutic exercise program (G1, mean age: 26.3±5.6years) and an aerobic and therapeutic exercise program (G2, mean age:26.0±4.6years). Headache frequency and intensity were evaluated using a headache diary, intensity was reported using a numerical pain rating scale (NRS), and headache impact was evaluated using a Headache Impact Test (HIT-6). These parameters were evaluated twice at baseline (A01/A02), at the end of the 8-week intervention period (A1), and 8–12 weeks after the end of the intervention (A2). The study protocol was approved by the ethical committee of the Egas Moniz University Institute on February 13, 2019 (reference number: 675). All individuals provided informed consent in accordance with the Helsinki Declaration and understood that they were free to withdraw from the study at any time. **Results:** None of G2 participants reported having headaches, and in G1, only two participants reported headache, at A1. Scores for headache intensity (0.3 [95%CI: -0,401, 1.068]), (0.0 [95%CI: -0.734,0.734]), significantly decreased in G1/G2 at A1. Score of HIT-6 (50.7 [95%CI:38.008,63.459]), (49.5 [95%CI:36.808,62.259]), significantly decreased in G1 and G2 at A1. Effects obtained immediately after programs completion were maintained until the final follow-up in both groups. **Conclusion:** The programs conducted by G1 (therapeutic exercises) and G2 (therapeutic and aerobic exercise) had significant results at A1 and A2. The physiotherapy programs are important to reduce headache attributed to TMD.

## P45 Orofacial Pain- An Emerging Speciality Bridging the Gap between Medicine and Dentistry

### S. Gupta

#### RAK Dental Care and Implant Centre, ORO Facial Pain, Ras Al Khaimah, United Arab Emirates

Orofacial Pain refers to pain associated with the hard and soft tissues of the head, face, and neck. These tissues, whether skin, blood vessels, teeth, glands or muscles send impulses through the trigeminal nerve to be interpreted as pain by the brain. A vast variety of disorders fall under the umbrella of orofacial pain disorders. There is a bidirectional association between orofacial pain and sleep.

Orofacial pain is a relatively common complaint in general medicine and dental practice. Diagnosis and treatment of pain originating from the head, face, oropharynx, ears, sinonasal area and neck is a complex process compounded by the density of anatomical structures and the prominent psychologic significance attributed to this region. Management of orofacial pain thus demands the service of clinicians from various specializations such as dentistry, otolaryngology, ophthalmology, neurology, neurosurgery, psychiatry and psychology. The quest to better manage pain problems involving the head and neck area has led to the establishment of Orofacial pain as a discipline in the field of dentistry.

Orofacial pain remains a prevalent and debilitating condition with significant social and economic impacts. Clearly the task required is integration of knowledge in this anatomically dense region, traditionally divided between many medical disciplines. Management of orofacial pain requires a professional collaboration between dentists and medical doctors Based on extensive clinical experience with patients suffering from facial pain and headache, an Orofacial pain clinician is well equipped to fulfill this task of giving adequate relief to an orofacial pain patient and improving his/her quality of life.

REFERENCES

1. OroFacial Pain – Guidelines for Assessment, Diagnosis and Management ,The American Academy of OroFacial Pain - Reny de Leeuw, Gary D. Klasser.

2. Bell"s Oral and Facial Pain - Jeffrey P. Okeson

3. The face of Dental Sleep Medicine in 21st century J Oral Rehab Dec 2020

## P46 Association of pain intensity and psychological factors among patients with symptomatic temporomandibular disorders: a cross-sectional study

### S. Martín Pérez^1^, A. Cabrera Fuentes^2^, A. Martín Rivero^2^, G. García Domínguez^2^, J. L. Alonso Pérez^1^, I. M. Martín Pérez^3^

#### ^1^Universidad Europea de Canarias, Musculoskeletal Pain and Motor Control Research Group, Faculty of Health Sciences, Santa Cruz de Tenerife, Spain; ^2^Universidad Europea de Canarias, Musculoskeletal Pain and Motor Control Research Group, Master Degree in Orthopaedic Manual Therapy, Faculty of Health Sciences, Santa Cruz de Tenerife, Spain; ^3^University of La Laguna, Department of Pharmacology and Physical Medicine, Faculty of Health Sciences, Santa Cruz de Tenerife, Spain

##### **Correspondence:** S. Martín Pérez

**Question:** Are pain intensity and psychological variables associated in patients with symptomatic temporomandibular joint disorders?

**Method:** A cross-sectional study was carried out with convenience sampling between February 1, 2022, and May 30, 2022, at the Universidad Europea de Canarias (Spain). Adults with TMJ pain with more than 12 weeks of evolution and who would not be undergone pharmacological or physiotherapeutic treatment were selected. An assessment of pain intensity (*VAS*) as well as anxiety (*STAI*), catastrophizing (*PCS*), perceived stress scale (*PSS*), and sleep quality (*PSQI*) was performed. Moreover, the statistical analysis was carried out using the *Jamovi 2.3.12* software, performing the descriptive analysis, the *Shapiro Wilks* normality test (p<0.05), and the strength of correlational association through the calculation of the Pearson Correlation Coefficient (*Pearson's r).*

**Results:** We recruited 21 subjects (F:17; M:4) aged mean of 41.47 (SD=10.28) suffering from symtomatic TMJ dirsorder with a pain intensity of 4.84 (SD=1.70), anxiety 26.11 (SD=5.22), catastrophism 17.05 (SD=13.05), perceived stress 25.58 (SD= 8.60) and sleep quality 8.26 (SD=4.17). After checking the normality of the data, a weak linear correlation was found between the pain intensity and anxiety (*Pearson's r* = 0.141; r^2^=0.019; p=0.564), pain intensity and catastrophism (*Pearson's r*= 0.180,r^2^=0.032;p=0.462) and pain intensity and perceived stress (*Pearson's r*=0.358;r^2^=0.128;p=0.132). In contrast, moderate and negative strength of association between pain intensity and sleep quality was detected (*Pearson's r*= 0.403,r^2^=0.163;p=0.087).

**Conclusions:** Psychological variables were not associated with pain intensity among TMJ patients. However, sleep quality was the only variable that maintains a moderate linear association with pain intensity.

**Fig. 1 (abstract P46).**
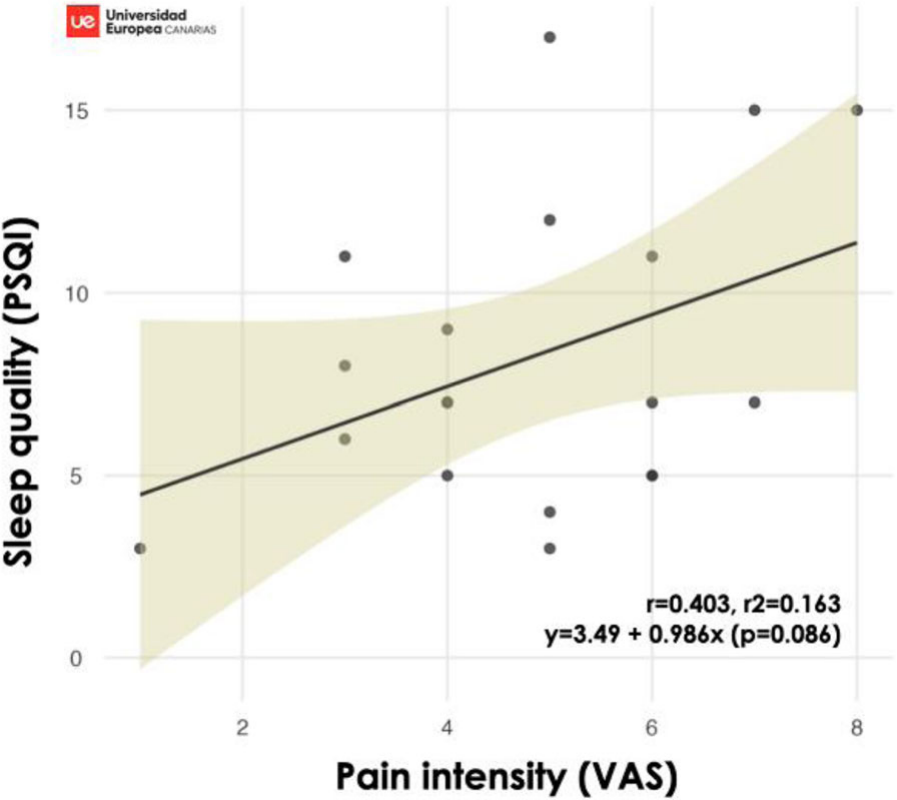
See text for description.

## P47 Neurovascular Orofacial Pain – A Diagnostic Dilemma

### S. Gupta

#### RAK Dental Care and Implant Centre, ORO Facial Pain, Ras Al Khaimah, United Arab Emirates

The sites of the migraine headache are predominantly temporal, supraorbital, frontal, parietal, and occipital. However, they may occur in the orofacial region also. Referral of pain to maxillary teeth is not uncommon. In view of the uncommon pain location, a high number of these patients are misdiagnosed with dental or sinus-related conditions, frequently resulting in inappropriate surgical and medical treatments. If the oro-facial area is the focus of the pain, the newly developed International Classification of orofacial pain refers to "Orofacial Migraine" and "Trigeminal autonomic orofacial pain". Benoliel et al introduced the term Neurovascular oro-facial pain (NVOP), previously also described as lower facial migraine.

Migraine can be localized in the face resembling facial or dental pain, indicating the influence of the trigeminovascular system in the structures innervated by the maxillary and mandibular branches of the trigeminal nerve. The clinical features of NVOP contains a distinctive combination of signs and symptoms common to both migraine and trigeminal autonomic cephalalgias. In contrast to migraine, patients are older at the time of onset and even more predominantly female. Frequently, there is cold allodynia of several teeth. This finding needs to be investigated properly, since it would be an important test and might link this entity to migraine, in which mechanical allodynia is seen during attacks.

NVOP is becoming increasingly recognized in medical and dental clinics. It is important for clinicians evaluating patients with facial pain to show their diligence and identify the associated symptoms of migraine, so as to avoid unnecessary treatments and surgical procedures and deliver appropriate medical therapy. The collaboration between neurologists and facial pain specialists is key to increasing awareness and education on this rare but treatable manifestation of an otherwise very frequent headache disorder.

References

1. Interplay of Oral, Mandibular, and Facial Disorders and Migraine Curr Pain Headache Rep 2022 May

2. Migraine presenting as isolated facial pain: Cephlalgia 2020

3. International Classification of Orofacial Pain. Cephalalgia 2020

## P48 Role of Orofacial Pain Specialists in Diagnosis and Management of Oropharyngeal, Head and Neck Cancer

### S. Gupta

#### RAK Dental Care and Implant Centre, ORO Facial Pain, Ras Al Khaimah, United Arab Emirates

Patients with oropharyngeal, head and neck cancer often experience pain and suffering that reduces quality of life, increases anxiety and depression, affects well being and even compliance with treatment. Also, oral manifestations of hematologic cancers and metastasis to oral tissues may cause pain with similar effects. Orofacial pain can be due to cancer itself, due to cancer therapy or due to noncancerous etiology in cancer patients. Even cancer therapy is well known to frequently induce painful oral complications.

Pathogenesis of oral cancer is not fully understood and various mediators like endothelin-1, proteases and nerve growth factor have been implicated. Effective management of orofacial pain in patients with cancer requires comprehensive assessment of multifactorial etiologies and treatment directed at these causative factors.

Orofacial pain in cancer patients remains a prevalent and debilitating condition with significant social and economic impacts. Clearly the task required is integration of knowledge in this anatomically dense region, traditionally divided between many medical disciplines. Management of orofacial pain requires a professional collaboration between dentists and medical doctors. Dentists, Orofacial pain specialists in particular, play an important role in screening oropharyngeal, head and neck cancer patients, helping in the diagnosis and management by making appropriate referrals. Based on extensive clinical experience with patients suffering from facial pain and headache, an Orofacial pain clinician, being part of the multidisciplinary team, is well equipped to fulfill the task of giving adequate relief to orofacial pain in cancer patients and thereby improving quality of life.

Orofacial pain in patients with cancer can be managed by using topical therapy, non-opioid and strong opioid analgesics, adjuvant and centrally acting analgesics as well as adjunctive or complementary management strategies.

## P49 Use of anti-CGRP monoclonal antibodies in pediatric migraine: first evaluations of a phase 3, randomized, double-blind, placebo-controlled study

### F. Ursitti, L. Papetti, G. Sforza, M. A. N. Ferilli, G. Monte, R. Moavero, M. Valeriani

#### Ospedale Pediatrico Bambino Gesù, Neurologia, Rome, Italy

##### **Correspondence:** F. Ursitti

Objective: to date, prophylactic therapies for migraine include the use of antiepileptic drugs, calcium antagonists or antidepressants. In recent years, studies have been conducted on adults with the monoclonal antibody that binds the receptor of the peptide related to the calcitonin gene (GCRP), which competes specifically with the binding of CGRP to its receptor by inhibiting its function. CGRP modulates the nociceptive signal and is associated with the pathophysiology of migraine. Therapies currently available in children have limited efficacy. There is therefore a need for additional drugs. Methods: 8 patients with chronic migraine and 1 patient with episodic migraine were enrolled in the study according to the criteria of the International Classification of Headaches (ICHD-III). Patients with chronic migraine are in the following phases: 1 finished the study, 1 dropped out, 2 in the double-blind phase, 3 in the open-label and dose-blind phase, and 1 moved from the episodic migraine study to chronic. Results: among the 8 patients with chronic migraine, it can be stated that 4 patients reported a reduction in the frequency and intensity of monthly migraine attacks, 2 patients reported the ineffective therapy and 2 patients, in whom the double-blind phase was not started, are being evaluated. In three of the four patients with chronic migraine, who had a good response to therapy, a reduction in the frequency and intensity of monthly attacks was observed starting from the double-blind phase. Conclusion: to date our preliminary data on the efficacy of anti-CGRP antibodies in pediatric age, even if under evaluation, confirm what has been found in double-blind studies on the adult population, or the possibility of having a prophylactic drug specific and efficacy for migraine. However, more pediatric studies will be needed to confirm these preliminary results.

## P50 Real-world Reductions in Monthly Migraine Days and Migraine-Related Healthcare Resource Utilization in UK Patients Using Fremanezumab

### S. Afridi^1^, T. Totev^2^, L. J. Krasenbaum^3^, E. Terasawa^4^, H. Akcicek^5^, D. Dhiraj^6^, R. Sun^2^, B. Yilma^4^, M. T. Driessen^5^

#### ^1^Headache Service, Department of Neurology, Guy’s and St Thomas’ NHS Trust, London, United Kingdom; ^2^Analysis Group, Inc., Boston, MA, United States; ^3^Teva Branded Pharmaceutical Products R&D, Inc., West Chester, PA, United States; ^4^Analysis Group, Inc., New York, NY, United States; ^5^Teva Pharmaceutical B.V., Amsterdam, Netherlands; ^6^Teva UK Ltd., Castleford, United Kingdom

##### **Correspondence:** H. Akcicek

**Objective:** To assess the real-world effectiveness and healthcare resource utilization (HCRU) of fremanezumab in adults with episodic/chronic migraine (EM/CM) treated by multiple physicians from the UK.

**Methods:** This UK panel-based online physician chart review used electronic case report forms. Patient inclusion criteria included a physician diagnosis of CM/EM in Scotland and CM in England, Wales, and Northern Ireland; first fremanezumab treatment initiation (index date) at aged ≥18 years from June 2020–October 2021; ≥3 months of continuous treatment after index date; monthly migraine days (MMD) and monthly headache days (MHD) assessments 1 month before (pre-index) and 3 months (±15 days) after (post-index) initiation; and ≥3 months of information about migraine treatments prior to index date. Migraine-related HCRU was compared for 6 months pre- and post-index. *P*

**Results:** Data were included from 42 neurologists and 183 patients (mean age[SD], 40.5[11.3]; female, 129[70.5%]; CM diagnosis, 174[95.1%]). 95(51.9%) patients initiated monthly fremanezumab dosing, and 88(48.1%) initiated quarterly dosing. Reductions in MMD were observed across dosing subgroups after 3 months of treatment (percent reduction from baseline: overall, 51.9[25.7]; monthly, 52.9[28.3]; quarterly, 50.8[22.7]; all *P*<0.001). Reductions in MHD were also observed (percent reduction from baseline: overall, 47.3[27.0]; monthly, 49.2[28.3]; quarterly, 45.0[25.5]; all *P*≤0.002). Reductions in HCRU were observed from 6 months pre-index to 6 months post-index: outpatient office visits (*P*=0.005); urgent care/ER visits (*P*=0.001); inpatient admissions (*P*=0.028). Reductions, not statistically significant, were observed in the number of telehealth consultations.

**Conclusions:** Fremanezumab reduced MMD and MHD after 3 months and reduced migraine-related HCRU after 6 months in a real-world UK population of patients with migraine from multiple physicians.

## P51 Anti-CGRP mAbs in a difficult to treat migraine population. Efficacy and safety

### D. I. Samuel, N. C. Candela, O. Marina Alejandra, J. Perez Garcia, M. J. Navarro Muñoz

#### Hospital Universitari i Politècnic La Fe, Neurology, Valencia, Spain

##### **Correspondence:** D. I. Samuel

QUESTION

To evaluate efficacy and safety of erenumab (E), Galcanezumab (G) and fremanezumab (F) as preventive treatment in a difficult to treat migraine population in our headache unit.

METHODS

Prospective and descriptive study of the use of E, G and F in our headache unit since their approval. Measures of efficacy: migraine days per month (MMD), triptans days per month (TDM) and overuse of symptomatic medication. We use scales as Patient Related Outcome (PROs): MIDAS, HIT-6, Pain Catastrophizing scale (PCS) and migraine-specific quality of life questionnaire (MsQol). We evaluate each patient at baseline and every 3 months. Follow up of adverse events also every 3 months.

RESULTS

336 patients reached at least 3 months of treatment and 233 patients 6 months. 140 patients were treated with E, 139 with G and 57 with F. Mean age was 46,78 years, 81,55% were women and 88,09% were diagnosed of chronic migraine. They had failed an average of 5,39 previous preventive treatments.

At baseline they had: 19,50 MMD; 13,78 TDM; 69% overuse of triptans; MIDAS: 90,62, HIT-6: 68,75, PCS: 31,77, MsQol: 31,23.

MMD with E at baseline, 3 and 6 months was: 20,53; 13,48 y 10,73.

With G: 19,33; 11,34 y 9,83.

With F: 17,40; 9,47 y 8,41.

PROs changed in the same way with all the anti-CGRP mAbs. MIDAS scale was reduced to 53,69 points at 3 months and to 40,66 at 6 months; HIT-6 reduced to 60,37 points at 3 months and 58,33 at 6 months and the overuse of triptans reduced to 31,54% at 3 months and 29,18% at 6 months.

Regarding adverse events (AE), constipation is the most frequent AE, reaching 19% of all patients. It trends to reduce its prevalence as time goes by.

CONCLUSION

All anti-CGRP mAbs are effective in patients with difficult to treat migraine. Constipation is the most frequent AE, greater than in clinical trials, not clinically significant and improving over time with treatment. The results of efficacy and safety are similar among the 3 a-GCRP mAbs.

## P52 The efficacy of CGRP monoclonal antibodies – an Australian experience

### J. Ray^1,2,3^, L. Dalic^2^, S. Cheng^1^, E. Hutton^1,3^

#### ^1^Alfred Health, Neurology, Melbourne, Australia; ^2^Austin Health, Neurology, Melbourne, Australia; ^3^Monash University, Neurosciences, Melbourne, Australia

##### **Correspondence:** J. Ray

Objective: To assess the real-world efficacy of CGRP monoclonal antibodies (mAb) in an Australian setting.

Methodology: A retrospective cohort study was undertaken of all patients commenced on a CGRP monoclonal antibody at two Victorian tertiary hospitals over the first 12-months of listing of the medication on the Pharmaceutical Benefits Scheme (PBS).

Results: Over the study period, 163 patients were commenced on either galcanezumab or fremanezumab. The study population had a median age of 44 (IQR 16), was 71.3% female, had failed a median of 5 previous migraine preventers (IQR 4) and a baseline mean monthly headache day (MHD) of 23.9 (SD 7.7). Amongst patients who were CGRP mAb naïve, the 50% responder rate was 55.9%, with a mean reduction of MHD of 10.4 (SD 9.7). A total of 25 patients were transitioned to a CGRP mAb from onabotulinumtoxinA (onaB) for incomplete response, with a baseline median MHD of 18 (IQR 23). The 50% responder rate was 40%, with a mean reduction of MHD of -3 (-7.8-1.7) beyond the effect of onaB. A Kaplan-Meier test was run to determine if there were differences in the survival distribution between galcanezumab and fremanezumab. The survival distributions for these interventions were not statistically significantly different, X2(2)=0.673, p=0.412.

Conclusion: CGRP monoclonal antibodies were effective treatments of migraine in an Australian population of migraine who had failed multiple preventative medications, including in patients who had sub-optimal responses to onaB.

## P53 Use of non-pharmacological therapies in individuals with migraine eligible for treatment with monoclonal antibodies targeting calcitonin gene-related peptide (CGRP)-signaling: a single-center cross-sectional observational study

### L. Rundblad^1^, C. K. Cullum^1^, S. Sacco^2^, R. Gil-Gouveia^3^, D. Uludüz^4^, T. P. Do^1,5^, F. M. Amin^1,6^

#### ^1^Danish Headache Center, Glostrup, Denmark; ^2^University of L'Aquila, L'Aquila, Italy; ^3^Hospital da Luz, Lisbon, Portugal; ^4^Istanbul University Cerrahpaşa School of Medicine, Istanbul, Turkey; ^5^Danish Headache Center, Glostrup, Denmark; ^6^University of Copenhagen, Rigshospitalet, Department of Neurorehabilitation/Traumatic Brain Injury, Copenhagen, Denmark

##### **Correspondence:** C. K. Cullum

**Introduction:** Treatment with monoclonal antibodies targeting the calcitonin gene-related peptide (CGRP) signaling pathway is impeded by regulatory restrictions. Affected individuals may seek out other services including non-pharmacological therapies. Thus, we found it timely to ascertain the use of non-pharmacological therapies in individuals with treatment-resistant migraine eligible for and naïve to treatment with CGRP-signaling targeting monoclonal antibodies (mAbs).

**Methods:** Single-center cross-sectional observational study of patients eligible for and naïve to treatment with mAbs targeting CGRP or its receptor. We recorded demographical information, frequency of headache and migraine days, previous use of preventive pharmacological medications for migraine, and use of non-pharmacological therapies the past 3 months including frequency of interventions, costs, and patient-reported assessment of efficacy on a 6-point scale.

**Results:** We included 122 patients between June 17, 2019, and January 6, 2020; 101 (83%) were women and the mean age was 45.2±13.3 years. One-third (n=41 [34%]) had used non-pharmacological therapy within the past 3 months. Among these participants, median frequency of different interventions was 1 (IQR: 1-2), median number of monthly visits was 2 (IQR: 1-4), mean and median monthly costs were 1086±1471 and 600 (IQR: 0-1200) DKK (1 EUR = ~7.5 DKK), respectively, and median patient-reported efficacy of interventions was 2 (IQR: 0-3).

**Conclusions:** Even in a high-income country with freely accessible headache services and universal healthcare coverage, there was a non-negligible direct cost in parallel with a low satisfaction for non-pharmacological therapies amongst patients at a tertiary headache center.

## P54 Role of Monoclonal Antibodies Against the Calcitonin Gene-Related Peptide or Receptor (CGRP-Mabs) for Chronic Migraine Prevention: A Critical Review

### S. Chowdhury, N. Rajpal

#### Vivekanand Polyclinic and Institute of Medical Sciences, General Medicine, Lucknow, India

##### **Correspondence:** S. Chowdhury

**Objective:** We aimed to critically review the role of CGRP-mAbs (monoclonal antibodies against the calcitonin gene-related peptide or receptor) namely erenumab, galcanezumab, fremanezumab and eptinezumab for chronic migraine (CM) prevention.

**Methods:** We searched PUBMED for all CGRP-mAbs trials conducted for CM prevention in adults. We analyzed the pivotal double-blind (DB) placebo-controlled trials of at least 12 weeks duration for efficacy and safety, post-hoc studies for subgroup analysis, patient-reported outcomes (PRO) for meaningful differences, and long-term trials for safety and effectiveness.

**Results:** We analyzed a total of 61 studies. The results are summarized in table 1. In the DB trials, the difference in reduction of migraine headaches days between CGRP-mAbs and placebo ranged from 1.7 to 2.6 days. ≥50% responder rate varied from 27.6% to 61.4%. The best results for both these outcomes were obtained by 300mg quarterly (12 weekly) intravenous eptinezumab. The difference in reduction of acute medication days between CGRP-mAbs and placebo ranged from 1.2 to 2.6 (the best result was obtained by monthly subcutaneous140mg erenumab). Mild, self-limiting adverse effects were reported. Post-hoc analyses showed that these drugs were effective in patients with coexistent medication overuse and prior failure to multiple preventives. PRO such as disability, functionality, and quality of life also showed significant and meaningful improvements. Long-term trials (1to 5years) showed consistent efficacy, no significant immunogenicity and no new safety concerns. References: Figure 1 and 2.

**Conclusion:** Erenumab, galcanezumab, fremanezumab and eptinezumab showed statistically superior and clinically meaningful efficacy compared with placebo for the prevention of CM with good long-term tolerability and safety profile.

**Fig. 1 (abstract P54).**
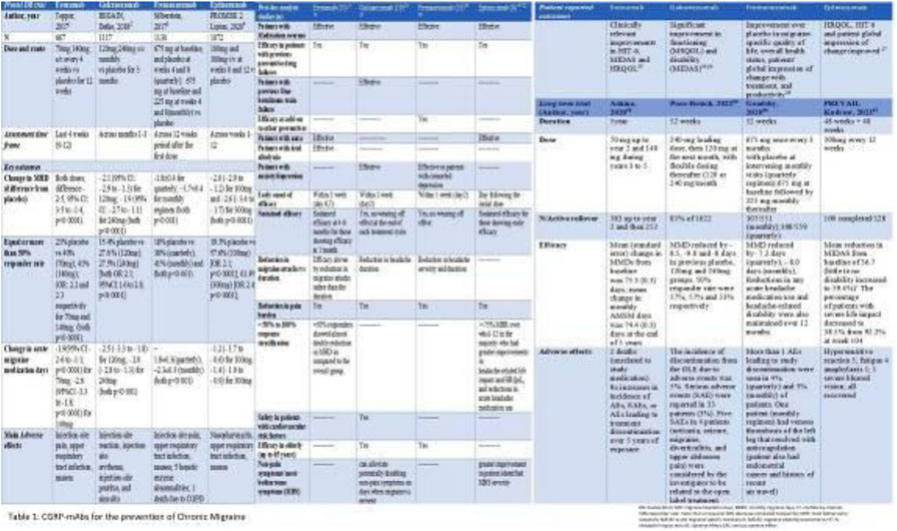
See text for description.

**Fig. 2 (abstract P54).**
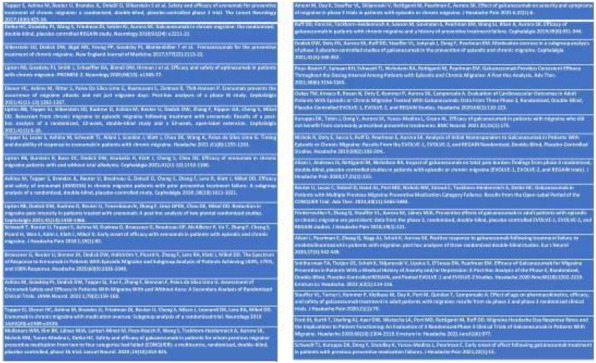
See text for description.

## P55 Real Life Experience: Use of CGRP Inhibitors in Patients Older than 55 Years Old, are they Safe and Effective?

### M. V. Castro Sanchez^1^, H. Antoli Martinez^1^, A. Sanchez Guijo Benavente^1^, L. Rodriguez Jimenez^1^, L. Garcia Trujillo^1^

#### ^1^Regional University Hospital of Malaga, Neurology, Malaga, Spain

##### **Correspondence:** M. V. Castro Sanchez

QUESTION: At 2018 European Medicines Agency authorized CGRP inhibitors as a treatment for episodic and chronic migraine. Erenumab and Galcanezumab included in its clinical essays patients until 65 years old. Fremanezumab included patients until 70 years old, but did not specify the percentage of older patients or their comorbidities. There are few data of this group who usually has other comorbidities and vascular risk factors. Our objective is to describe the efficacy and security of CGRP inhibitors in clinical practice.

METHODS: We have retrospectively reviewed 32 patients with ages between 55 and 77 years old treated with CGRP inhibitors. We included patients who began CGRP inhibitors between 2019 and 2022. We considered CGRP inhibitors were effective if patients achieved a decrease of 50% of monthly migraine days or a decrease higher than 5 points in MIDAS or HIT-6 scales.

RESULTS: 3 patients were male and 25 female. 4 patients had episodic migraine and 28 chronic migraine with an average MIDAS of 106.5 and HIT-6 of 67.5. Patients had tried an average of 6 preventive medications, including botulinum toxin 31 of them. 15 patients had vascular risk factors, 6 had high blood pressure, 11 hypercholesterolemia, 1 diabetes. Efficacy was of 40.6%, 12/24 patients responded to erenumab, 1/4 to galcanezumab and 0/4 to fremanezumab. 11 patients who did not respond to a first CGRP inhibitor switched to another and 7/11 responded. Adverse effects appeared in 46.9% of the patients. The most common was constipation in 9 patients, followed by articular pain and local erythema in 2 patients. There were 3 significant adverse effects: a paralytic ileus, hypertensive emergency and a patient who reported worsening of her inflammatory arthritis.

CONCLUSIONS: In our series the efficacy of CGRP inhibitors in older patients were similar to the efficacy reported in clinical essays. Most adverse effects were minor, only 3 leaded to discontinuation of the treatment.

## P56 Real life experience and learning curve of Galcanezumab in migraine. Galca-only Consortium

### V. Obach^1^, S. Fernandez Fernandez^1^, N. Fabregat^1^, T. Marco^1^, I. Martin^1^, F. Velasco^2^, M. Martin Bujanda^3^, D. Garcia-Azorin^4^, E. Cuadrado^5^, D. Guisado^5^, A. Moreira^5^, A. Suarez^5^, S. Aranceta^6^, A. Ruisanchez^7^, J. C. Garcia Monco^8^, N. Roncero^8^, A. Minguez^9^, M. Ruibal^9^, I. Kortazar^10^, A. Echeverria^10^, A. Lopez Bravo^11^, R. Alvarez Escudero^12^, N. Riesco Perez^12^, L. Gonzalez-Fernandez^12^

#### ^1^Hospital Clinic of Barcelona, Neurology, Barcelona, Spain; ^2^Hospital Cruces, Bilbao, Spain; ^3^Hospital Navarra, Pamplona, Spain; ^4^Hospital Valladolid, Valladolid, Spain; ^5^Hospital del Mar, Barcelona, Spain; ^6^Hospital Tauli, Sabadell, Spain; ^7^Hospital Galdako, Bilbao, Spain; ^8^Hospital Basurto, Bilbao, Spain; ^9^Hospital Donostia, San Sebastian, Spain; ^10^Hospital Txagorritxu, Alava, Spain; ^11^Hospital Reina Sofia, Tudela, Spain; ^12^Hospital Central de Asturias, Oviedo, Spain

##### **Correspondence:** V. Obach

**QUESTION**

Safety and efficacy of galcanezumab at 12 months from a multicentre registry

**METHODS**

Pharmacy Commission of 12 centers with Headache Unit or monographic headache neurologist, approved Galcanezumab use in 2020 as the first line MAB for high frequency (>7 attacs/month, refractory to 3 oral preventive treatments) or chronic migraineurs also refractory to BOTOX.

Consecutive candidates were interviewed for demographics, monthly headache days (MHDs) and previous BOTOX use.

Patients were grouped into Q1 to Q4 according to the quartile time of inclusion in each center to assess a learning curve effect.

Satisfactory response was considered when reduction of more than 50% in MHDs was achieved (SR50) at 12 months.

**RESULTS**

One thousand and four patients received galcanezumab. Q1(n=257), Q2(n=252), Q3(n= 248) and Q4(n=247). Mean age was 50 years old (SD 12), female gender 83.1%, median MHDs was 20 [12-30].

According to the quartile distribution, the prevalence of chronic migraine was 80.9%, 80.6%, 76.2%, 67.6%; duration of migraine chronification 7, 9, 5 and 4 years; median HIT6 was 69 [64-72], 68 [66-72], 69 [66-74] and 70 [66-74]; Anxiety and mood disorders 39%, 34%, 48.1% and 39%; and Fibromyalgia 11.3%, 10.5%, 16.6% and11.8%, respectively. Concomitant Botox Use (MAB add-on) at baseline was 26.2%, 27.5%, 24.7 and 27.8%.

At 12 month, SR50 was 55.3%, 41.1%, 40.4% and 45.5% (p=0.01). Galcanezumab was withdrawn due to improvement in 22.9%, 25.5%, 23.2% and 19.6%.

SR50 in patients with treated mental disorder was 37.8% (vs 50.2%, p=0.01) and with fibromyalgia 23.8% (vs 47.7%, p=0.001)

**CONCLUSION**

We do not detect any learning curve in Galcanezumab efficacy in migraine therapy and our first treated patients seemed to have been more accurately selected therapy and our first treated patients were more accurately selected to treatment. Anxiety and mood disorders t and fibromyalgia reduce Galcanezumab efficacy.

## P57 Real-world Experience with Galcanezumab for the Preventive Treatment of Cluster Headache

### S. J. Cho^1^, H. Mo^1^, H. S. Moon^2^, B. K. Kim^3^

#### ^1^Dongtan Sacred Heart Hospital, Neurology, Hwaseong, South Korea; ^2^Kangbuk Samsung Hospital, Neurology, Seoul, South Korea; ^3^Eulji Hospital, Eulji University, Neurology, Seoul, South Korea

##### **Correspondence:** S. J. Cho

Galcanezumab of 300 mg monthly is the FDA approved preventive medication for cluster headache (CH). Compared to the 120 mg galcanezumab syringe for the treatment of migraines, the 100 mg syringe for CH has globally not been as widely available. We evaluated patients with CH who received at least 1 dose of 240 mg (2 prefilled syringe of 120 mg) of galcanezumab in the 3 university hospitals from February 2020 to September 2021. In the patients with episodic CH, the efficacy and safety data of galcanezumab were analyzed regarding to the presence of the conventional preventive therapy at the timing of therapy of galcanezumab. The data of other subtypes of CH were separately described. Results: In 47 patients with episodic CH, galcanezumab was started median 18 days after the onset of current bout (range 1-62 days) and 4 patients (10.8%) received second dose of galcanezumab. The median time to the first occurrence of 100% reduction from baseline in CH attacks per week after galcanezumab therapy was 17 days (25% to 75% quartile range: 5.0~ 29.5) in all patients with episodic CH, 15.5 days (3.8~ 22.1) in 36 patients with galcanezumab therapy adding on conventional preventive therapy, 21.0 days (12.0~ 31.5) in 11 patients started galcanezumab as initial preventive therapy. Among 33 patients with headache diary, the proportion of patients with 50% reduction at week 3 from baseline 78.8% about the numbers of CH attacks per week and 79.3% about the days with acute medications per week. Patient global impression of improvement was reported as feeling "very much better" or "much better" in 80.9% of patients with episodic CH and all 3 patients with chronic CH or the first episode of cluster bout. One 240 mg dose of Galcanezumab with/without conventional therapy for the prevention of CH is considered effective and safe in clinical practices, as seen in the clinical trial of galcanezumab.

## P58 Recurrent painful ophthalmoplegic neuropathy treated with erenumab: A case report

### D. Mahović^1^, M. Bračić^2^

#### ^1^University Hospital Center Zagreb, School of Medicine, University of Zagreb, Department of Neurology, Zagreb, Croatia; ^2^Andrija Štampar Teaching Institute of Public Health, Department of School and Adolescent Medicine, Zagreb, Croatia

##### **Correspondence:** D. Mahović

Background and objective: Recurrent painful ophthalmoplegic neuropathy (RPON), formerly known as ophthalmoplegic migraine, is a rare type of cranial neuralgia characterized by attacks of unilateral headache with ipsilateral ophthalmoplegia due to paresis of one or more ocular cranial nerves. The exact pathophysiology behind RPON is unclear and the clinical presentation often resembles that of migraine disorders. The objective of this paper is to present the first reported use of erenumab in a patient with RPON.

Methods: Case description.

Results: A 31-year-old woman with a 3-year history of recurrent unilateral headache, ipsilateral ptosis, nausea, and photo- and photophobia was referred to our clinic due to suspected dural carotid-cavernous fistula observed on brain magnetic resonance imaging. Neurological examination revealed left-sided ptosis and mydriasis with a sluggish reaction to light. After excluding the presence of a dural fistula on digital subtraction angiography, the patient was diagnosed with RPON. Her symptoms subsided after receiving pulse corticosteroid therapy. She was discharged with rizatriptan for acute attacks and propranolol as prophylaxis. Over the course of the following 5 years, the patient didn"t experience a significant decrease in either intensity or frequency of her symptoms in spite of adjustments in prophylactic therapy. After numerous therapeutic failures with different classes of prophylactic drugs, including beta blockers, antidepressants and antiepileptics, erenumab was introduced in the prophylactic regimen (140 mg subcutaneously once every 28 days). While on erenumab, the patient experienced a 75% reduction in monthly headache days and this effect was sustained for 18 months.

Conclusion: The results of our case support the argument that RPON should be reclassified as a migraine variant, which would enable the use of specific prophylactic medication in patients suffering from this disorder. Consent to publish had been obtained.

## P59 Investigating the Long-Term Efficacy of Calcitonin Gene Related Peptide Monoclonal Antibodies

### A. Salim^1,2^, T. Peixoto Leal^1^, I. Mata^1,2^, Z. Ahmed^3,2^

#### ^1^Cleveland Clinic, Genomic Medicine Institute, Cleveland, United States; ^2^Case Western Reserve University, School of Medicine, Cleveland, United States; ^3^Cleveland Clinic, Center for Neuro-Restoration, Cleveland, United States

##### **Correspondence:** A. Salim

**Question:** Calcitonin gene related peptide monoclonal antibodies (CGRP mAbs) are a promising treatment for episodic and chronic migraine. Though many real-world studies have shown benefit at an average treatment time of 3 or 6 months, little is known about its long term efficacy. The objective of this study is to examine the efficacy of CGRP mAb after 1, 2, and 3 years. This longitudinal study will provide insight on the long-term treatment use of CGRP mAb.

**Methods**: We extracted Electronic Medical Records (EMR) containing migraine frequency data for Cleveland Clinic patients (n =2025) with 6 consecutive months of positive treatment to a CGRP mAb between June 2018 and December 2021. This cohort's (87.5% female, 47.0 ± 13.5 years old, 1586 with chronic migraine) monthly migraine days (MMD) were examined at 1 (n = 822), 2 (n = 407), or 3 (n = 101) years of CGRP mAb treatment. The responses were differentiated into Non-responders (MMD reduction of 25% or less), Responders (MMD reduction between 26-74%) and Super-responders (MMD reduction of 75% or greater).

**Results**: After 1 year of treatment, 55.5% were Super-responders, 38.4% Responders, and 6.2% became Non-responders. Following 2 years of CGRP mAb use, 53.6% continued a Super-response, 37.6% responded, and 9.1% stopped responding positively. After 3 years, 59.5% maintained a Super-responder status, 29.7% were Responders, and 10.8% had a negative response.

**Conclusions**: We have seen great benefits in the short-term treatment of this recent agent, and our results show that CGRP mAbs do maintain efficacy after extended periods of treatment, with only a minority losing benefit over time. This longitudinal study provides some clarity for the preserved long term benefit of the use of CGRP MAB for the treatment of migraine.

## P59a Cognition in Menstrually Related Migraine:neural correlates of working memory along the cycle

### A. Ruiz-Tagle^1^, A. Fouto^1^, G. Caetano^1^, C. Domingos^1^, I. Esteves^1^, R. Gil-Gouveia^2^, R. Nunes^1^, I. Pavão Martins^3^, P. Figueiredo^1^

#### ^1^IST-ID, Lisbon, Portugal; ^2^Universidade Católica Portuguesa, Center for Interdisciplinary Research in Health, Lisbon, Portugal; ^3^Universidade de Lisboa, Centro de Estudos Egas Moniz e Instituto de Medicina Molecular João Lobo Antunes, Faculdade de Medicina, Lisbon, Portugal

##### **Correspondence:** A. Ruiz-Tagle

QUESTION

Hormones play a preponderant role in triggering migraine attacks, with women having higher prevalence and severity of migraine due to their influence along the reproductive cycle^1^. The preictal, ictal and postictal phases tend to include cognitive executive difficulties along with the rest of the attack symptoms^2^. Fluctuations in neural sensitivity observed in migraine could underlie such difficulties^3^. On the other hand, functional and structural changes in brain structures related to cognitive processes along the menstrual cycle have also been documented^4^.

We aim to use functional Magnetic Resonance Imaging (fMRI) to evaluate working memory at different stages of the migraine cycle and compare to a non-migraine population while controlling for their menstrual phases.

METHODS

A clinical sample of 15 women suffering from episodic migraine with menstrual-related attacks were recruited. They underwent fMRI sessions with a verbal N-back task in different phases of the migraine cycle, namely, preictal, ictal, postictal and interictal phase. 15 non-migraine controls matched for gender and age were assessed during premenstrual and post ovulation phase. A neuropsychological battery and questionnaires quantifying clinical symptoms and attack description at the time of the exam were also applied.

RESULTS

We report results for 70 sessions of acquisition in whole brain group analysis using a cluster threshold of z > 2.3. We observed left orbital prefrontal areas with significantly higher activation during preictal (z =3.44), ictal (z=3.49) and interictal (z=3.3) phases compared to postictal phase.

CONCLUSIONS

The brain activation observed in prefrontal regions during the migraine attack phases could be related to cognitive inhibition while performing a working memory task.

REFERENCES

^1^ Vetvik & MacGregor 2017, Lancet Neurol

^2^ Vuralli et al 2018, The Journal of Headache and Pain

^3^ Schulte & May 2016, Brain

^4^ Dubol et al 2020, Frontiers in neuroendocrinology

## P60 Brain connectivity modifications induced by monoclonal antibodies targeting the CGRP pathway in migraine patients: a prospective HD-EEG, open-label, study

### R. De Icco^1^, M. Corrado^1^, F. Bighiani^1^, G. Vaghi^1^, V. Grillo^1^, A. Putortì^1^, D. Martinelli^1^, M. Semprini^2^, M. Allena^3^, G. Sances^1^, C. Tassorelli^1^

#### ^1^IRCCS Mondino Foundation, University of Pavia, Pavia, Italy; ^2^Istituto Italiano di Tecnologia, Genova, Italy; ^3^IRCCS Mondino Foundation, Pavia, Italy

##### **Correspondence:** R. De Icco

Question: Monoclonal antibodies targeting the CGRP pathway (mAbs) proved effective and safe as migraine preventive treatment. Due to their molecular weight, mAbs act outside of the blood brain barrier, namely in the peripheral component of the trigeminovascular system. Nonetheless, a reduced sensitization of the first order neuron in the trigeminal ganglion may induce secondary effects at central level. Here we aim to study the changes induced by mAbs in cortical brain connectivity recorded by means of high-density electroencephalography (HD-EEG).

Methods: We plan to perform 5 resting state HD-EEG recordings, at baseline (before mAbs treatment), and then every 3 months for one year. Here we present data regarding 16 migraine patients (age 44.7±10.6, 14 females, 11 with CM) who completed the first three months of mAbs treatment (T3). We aim to study the connectivity changes in the nodes of the default mode network (DMN): the right and left angular gyrus (RANG and LANG), the medial pre-frontal cortex (MPC) and the posterior cingulate cortex (PCC).

Results: At T3, mAbs treatment induced an inter-nodal connectivity reduction between MPC-PCC (p=0.025), MPC-LANG (p=0.020), MPC-RANG (p=0.043), and PCC-LANG (p=0.005). By contrast, the connectivity was enhanced between PCC-RANG (p=0.005) and LANG-RANG (p=0.003). At T3, 7 patients qualified as "Responder" to mAbs (reduction in monthly migraine days of at least 50% when compared to baseline). Responders were characterized by a baseline enhanced connectivity between MPC-PCC (p=0.042) and MPC-RANG (p=0.032), and by a reduced connectivity between LANG-RANG (p=.016).

Conclusions: We described brain connectivity modifications in the DMN of migraine patients after three months of mAbs treatment. We hypothesize that a reduced sensitization of the peripheral component of the trigeminovascular system may account for the observed findings. In addition, Responder patients showed a specific baseline brain connectivity pattern.

**Fig. 1 (abstract P60).**
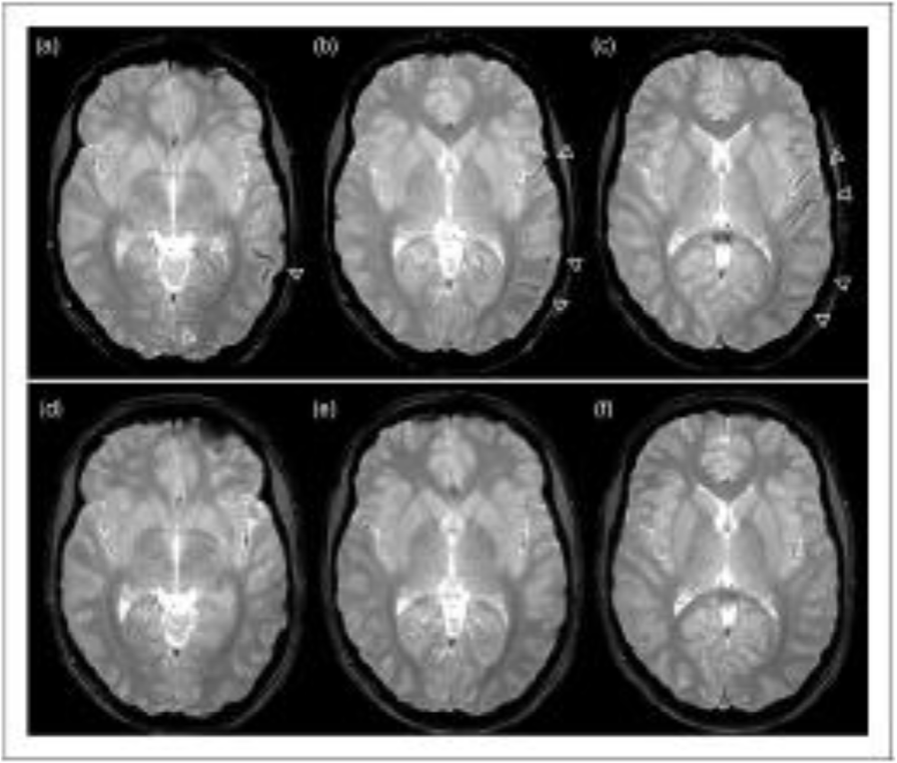
See text for description.

## P61 Diagnostic utility of T2*-weighted GRE in migraine with aura attack. The cortical veins sign

### C. Hirtz^1^, A. Viguier^2^

#### ^1^CHU Timone, Neurology, Marseille, France; ^2^Toulouse Hospital - Purpan, Toulouse, France

##### **Correspondence:** C. Hirtz

**Objective**: To evaluate the frequency, distribution, and clinical associations of the dilated appearance of cerebral cortical veins, termed cortical veins sign on T2*-weighted gradient recalled-echo (T2*-GRE) in the acute setting of migraine with aura attack in adult patients.

**Methods**: We conducted a retrospective analysis of 60 consecutive patients admitted for acute neurological symptoms with a final diagnosis of migraine with aura (42%) or probable migraine with aura (58%) who underwent emergency brain magnetic resonance imaging and 60 non-migrainous control adults. The cortical veins sign was defined as a marked hypointensity and/or an apparent increased diameter of at least one cortical vein. We examined the prevalence, the spatial distribution, and the associations of cortical veins sign with clinical characteristics of migraine with aura.

**Results**: We detected the cortical veins sign in 25 patients (42%) with migraine with aura, compared to none in the control group (p < 0.0001). The spatial distribution of cortical veins sign was characterised by the predominantly bilateral and posterior location. Presence of cortical veins sign was associated with increased severity of aura (p =0.05), and shorter delay to MRI (p =0.02).

**Conclusion**: In the setting of acute neurological symptoms, the presence of cortical veins sign is frequent in patients with migraine with aura and can be detected with good reliability. This imaging marker may help clinicians identify underlying migraine with aura.

**Table 1 (abstract P61).**
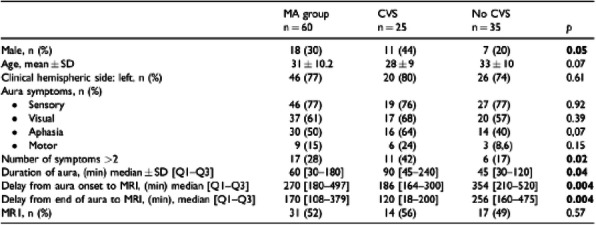
Clinical characteristics of the entire migraine aura (MA) group and acording to the presence of the cortical veins sign (CVS). Statistically significant results in bold

## P62 Emotional processing differences between migraine and tension-type headache subjects – an fMRI study

### D. Dobos^1,2^, K. Gecse^1,2^, E. Szabo^2,3,4^, D. Baksa^1,2^, N. Kocsel^2,4^, A. Galambos^2,4^, T. Zsombok^2^, G. Kokonyei^1,2,4^, G. Juhasz^1,2^

#### ^1^Semmelweis University, Department of Pharmacodynamics, Budapest, Hungary; ^2^Semmelweis University, SE-NAP 2 Genetic Brain Imaging Migraine Research Group, Hungarian Brain Research Program, Budapest, Hungary; ^3^Boston Children’s Hospital, Harvard Medical School, Center for Pain and the Brain (PAIN Research Group), Department of Anesthesiology, Critical Care and Pain Medicine, Boston, MA, United States; ^4^ELTE Eotvos Lorand University, Institute of Psychology, Budapest, Hungary

##### **Correspondence:** D. Dobos

**Objective:** The diagnosis of migraines and tension-type headaches is based on phenotypic characteristics. We currently do not know any marker in the nervous system along which we could separate the two diseases. The emotional processing of migraineurs has been proved to be altered in comparison with that of people without headaches. We wondered whether alterations would also be present when comparing migraineurs to subjects with tension-type headaches.

**Methods:** 45 episodic migraine (41 females) and 34 episodic tension-type headache subjects (24 females) performed an implicit face emotion processing fMRI task. After preprocessing raw images, individual contrast maps were created and used in a full factorial design to detect between-group differences in association with the average monthly headache frequency. The initial significance threshold was p<0.001 but only results surviving family-wise error correction (pFWE<0.05) were considered statistically significant. Both preprocessing procedure and statistical analysis of fMRI scans were performed in SPM12.

**Results:** At the sight of sad faces, migraine subjects showed less activation in the left supplementary motor area compared to tension-type headache subjects in association with the average monthly headache frequency (pFWE<0.05, voxel threshold=0).

**Conclusion:** Although both headache disorders are associated with negative mood, neural responses yielded to a negative emotion were different in migraine and tension-type headache subjects having similar headache frequency. Since the affected cortical region plays a role in emotional processing and cognitive control, we can speculate that the difference in its reaction might contribute to the differences in processing the affective component of pain.

**Funding:** 2017-1.2.1-NKP-2017-00002; KTIA_NAP_13-2- 2015-0001; 2020-4.1.1.-TKP2020; TKP2021-EGA-25; 2019-2.1.7-ERA-NET-2020-00005, and ÚNKP-20-3-II-SE-51.

## P63 Dynamic functional connectivity in migraine during the interictal phase: a resting-state fMRI study

### I. Esteves^1^, C. Fonseca^1^, M. Xavier^1^, A. Fouto^1^, A. Ruiz-Tagle^1^, G. Caetano^1^, R. Nunes^1^, R. Gil-Gouveia^2,3^, J. Cabral^4^, I. Pavão Martins^5^, A. Rosa^1^, P. Figueiredo^1^

#### ^1^ISR-Lisboa and Department of Bioengineering, Instituto Superior Técnico – Universidade de Lisboa, Lisbon, Portugal; ^2^Center for Interdisciplinary Research in Health, Universidade Católica Portuguesa, Lisbon, Portugal; ^3^Hospital da Luz, Neurology, Lisbon, Portugal; ^4^Life and Health Sciences Research Institute, University of Minho, Braga, Portugal; ^5^University of Lisbon, Centro de Estudos Egas Moniz e Instituto de Medicina Molecular João Lobo Antunes, Faculty of Medicine, Lisbon, Portugal

##### **Correspondence:** I. Esteves

**Question:** Migraine is a cyclic and complex disorder, characterized by attacks of headache, sensory and cognitive disturbances^1^. Thalamocortical connectivity in migraine has been found to be transiently abnormal^2^. Our aim was to assess if the dynamical properties of the migraine brain are affected during the interictal phase.

**Methods:** Resting-state functional MRI data was collected from 14 menstrual migraine patients without aura (interictal phase) and 12 healthy controls (menstrual post-ovulation phase). fMRI data processing included^3^: motion and distortion correction, temporal highpass filter, regression of motion and physiological confounds, spatial smoothing, and parcellation with the Desikan atlas. Dynamic functional connectivity (dFC) between regions was computed using phase coherence, and recurrent dFC states were identified by k-means clustering (k ranging between 3 and 15) of the leading eigenvectors of dFC in each time point^4^. Permutation tests were performed to evaluate statistically significant differences between patients and controls in the probability of occurrence and the mean lifetime of the dFC states.

**Results:** Similar dFC states were found consistently across different numbers of clusters, k, which resembled the canonical resting-state networks as expected. Compared to healthy controls, migraine patients show a significantly lower mean lifetime in one dFC state, when grouping in 4, 5 and 6 clusters. No differences were found for the probability of occurrence.

**Conclusions:** Migraine may be linked to a disruption of brain networks dynamics. This emphasizes the need to adopt time-resolved methods, in addition to static, to study functional connectivity, to better understand the mechanisms of migraine. Our next step will be to assess the dynamics of the migraine brain throughout the migraine cycle.

1.Goadsby et al., Physiological reviews, 2017

2.Tu et al., Neurology, 2019

3.Jenkinson et al., NeuroImage, 2012

4.Cabral et al., Scientific reports, 2017

## P64 Reliable posterior insula–operculum region gray matter volume alterations in vestibular migraine

### L. Dong, H. Li, J. Zhou

#### The First Affiliated Hospital of Chongqing Medical University, Neurology, Chongqing, China

##### **Correspondence:** L. Dong and J. Zhou

**Question**

Vestibular migraine (VM) is one of the most prevalent causes of episodic vertigo. Neuroimaging offers the possibility to investigate and localize the responsive brain areas in patients with VM. Voxel-based morphometry (VBM) has been generally considered as a reliable technique to analyze structural alterations, especially the gray matter volume (GMV) across neurological diseases. Despite all imaging data accumulated on GMV across the past decades, an overview of the imaging evidence of GMV differences in VM is still missing.

**Methods**

The coordinate based meta-analysis (CBMA) is a novel method to identify consistent and reliable brain alterations among individual neuroimaging studies. This study was performed under the latest algorithm of CBMA, seed-based d mapping with a permutation of subject images (SDM-PSI).

**Results**

5 studies were included after systemic review (103 patients and 107 healthy controls). Main CBMA showed significantly decreased GMV in the left rolandic operculum (SDM-Z value=-3.68, p=0.004, Voxels=629) with a peak MNI coordinate (-44, -12, 16) located in Brodmann area (BA) 48 and the two largest voxels belonging to the insula and rolandic operculum were consistently reported in VM patients compared to healthy controls. When removing a study with most patients (14/20) had predominantly left-sided headaches in sensitivity analysis, decreased GMV in the right Heschel gyrus (SDM-Z value=-3.83, p=0.003, Voxels=504) with a peak MNI coordinate (48, -12, 8) located in BA 48 was detected, which is symmetrical to the results reported in the main CBMA.

**Conclusions**

Our CBMA demonstrated the involvement of the posterior insula–operculum region in VM. The lateralization of the headache attack may determine the lateralization of the GMV alteration. Further longitudinal neuroimaging studies are necessary to draw more precise conclusions and the headache side may need to be taken into account when designing migraine-related neuroimaging studies.

## P65 Relation of post-stroke headache to cerebrovascular pathology and hemodynamics

### E. Abed

#### Al-Azhar University, Neurology, Cairo, Egypt

**Background:** Despite the high prevalence of cerebrovascular stroke, headache attributed to ischemic strokes is often undertreated and overlooked. The aim is to detect the relation of a post-stroke headache to cerebrovascular pathology and changes in hemodynamics through a high-resolution duplex ultrasound examination. **Methods:** This is a case-control study that was conducted on 239 patients who presented with an acute ischemic stroke. Patients were subdivided into two groups; Group I included patients with headache attributed to ischemic stroke (cases) and Group II included headache-free stroke patients (controls). History included headache characteristics and risk factors. Clinical and radiological examination were preformed to detect the type of stroke. Ultrasound duplex examination of the extracranial and intracranial cerebrovascular system was carried for both groups. **Results:** Group I included 112 patients (mean age 57.66 ±6.59 years), Group II included 127 patients (mean age 57.73±7.89 years). Post-stroke headache was more frequent in patients with posterior circulation infarction (58%). Post-stroke headache was reported within 7 days post-stroke in (61.6%) of patients. Pre-stroke headache was an independent predictor for post-stroke headache occurrence (OR=28.187, 95%CI; 6.612-120.158, P<0.001). Collateral opening and various degrees of intracranial vascular stenosis were strong predictors of headache occurrence (OR=25.071, 95% CI; 6.498-96.722, P<0.001). **Conclusion:** Post-stroke-headache is a common phenomenon especially in patients with pre-stroke headache, history of old stroke, posterior circulation infarction, and large artery disease. This headache was of moderate-intensity with clinical characteristics of tension-type. The intracranial cerebrovascular pathological changes including opening of the collateral channels and variable degrees of stenosis of cerebrovascular systems were implicated in the production of that headache. **Keywords:** Post-stroke headache; cerebrovascular; hemodynamics; duplex ultrasound.

**Fig. 1 (abstract P65).**
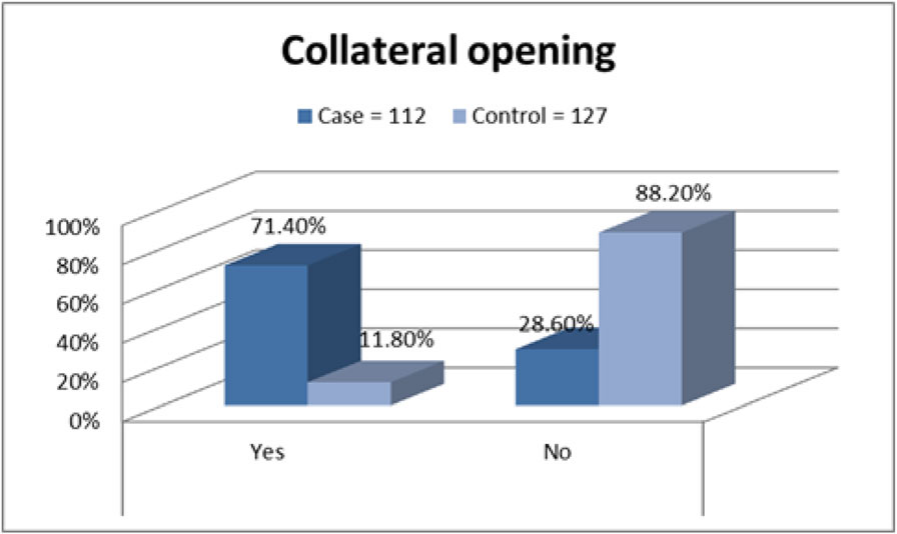
See text for description.

**Fig. 2 (abstract P65).**
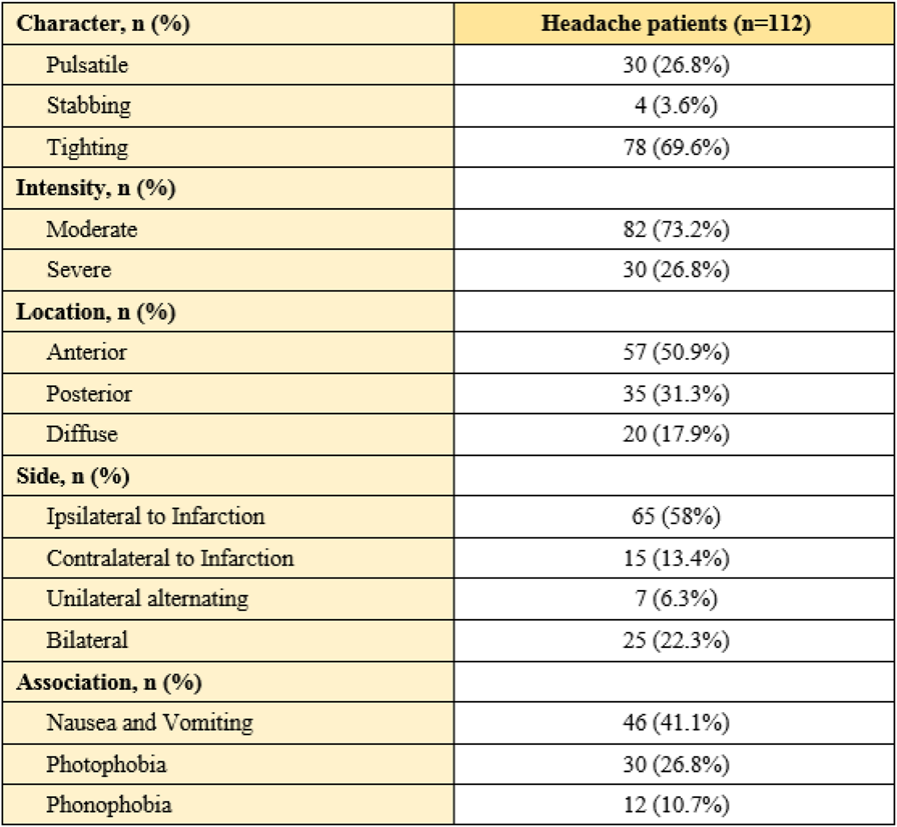
See text for description.

## P66 Differences of resting-state functional connectivity between patients with cluster headache and episodic migraineurs

### N. Imai^1^, A. Moriya^1^, E. Kitamura^2^

#### ^1^Japanese Red Cross Shizuoka Hospital, Neurology, Shizuoka-Shi, Japan; ^2^Kitasato University, Neurology, Sagamihara-shi, Japan

##### **Correspondence:** N. Imai

[Objectives] To investigate the differences in pathophysiology between cluster headache (CH) and episodic migraine (EM), we studied static and dynamic resting-state functional connectivity (RSFC) between patients with CH and EM. [Methods] Nineteen patients with CH and 19 sex- and age-matched episodic migraineurs were selected for the study. All patients fulfilled the International Headache Society criteria 3 CH or EM. High-resolution structural magnetic resonance imaging (MRI) and resting-state functional MRI (RS-fMRI) were performed in both groups. [Results] Region of interest (ROI)-to-ROI analyses in static RS-fMRI revealed that patients with CH showed 13 higher connectivity pairs mainly between the right parahippocampal gyrus and other brain lesions and 2 lower connectivity pairs than patients with EM (Fig. 1). ROI-to-ROI analyses in dynamic RS-fMRI showed that t patients with CH showed 8 higher connectivity pairs than patients with EM (Fig. 2). All 8 pairs in dynamic RS-fMRI were different from 15 pairs in static RS-fMRI. [Conclusions] Our study showed some differences in RSFC between CH patients and episodic migraineurs. Our data also revealed that patients with CH had some higher connectivity than those with EM.

**Fig. 1 (abstract P66).**
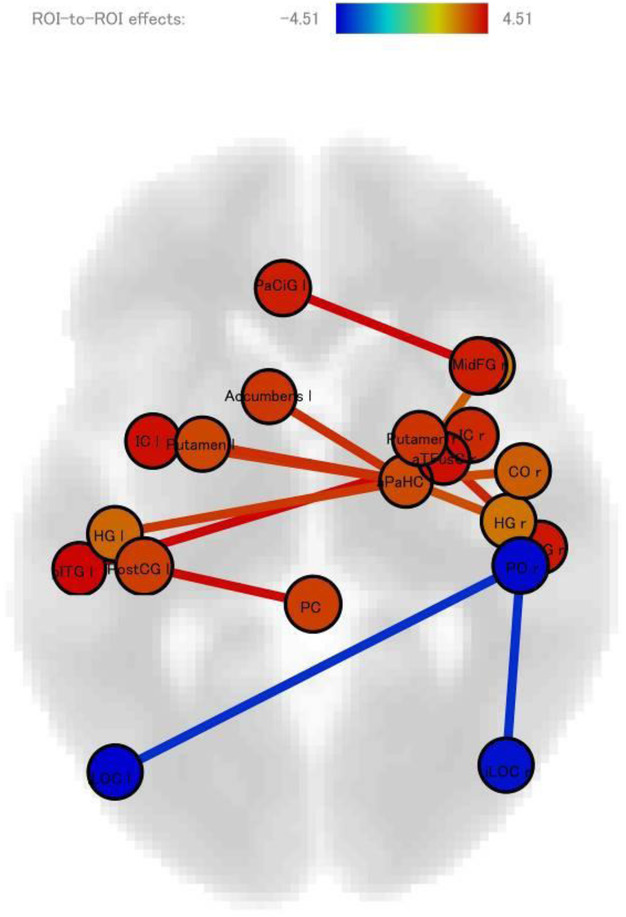
See text for description.

**Fig. 2 (abstract P66).**
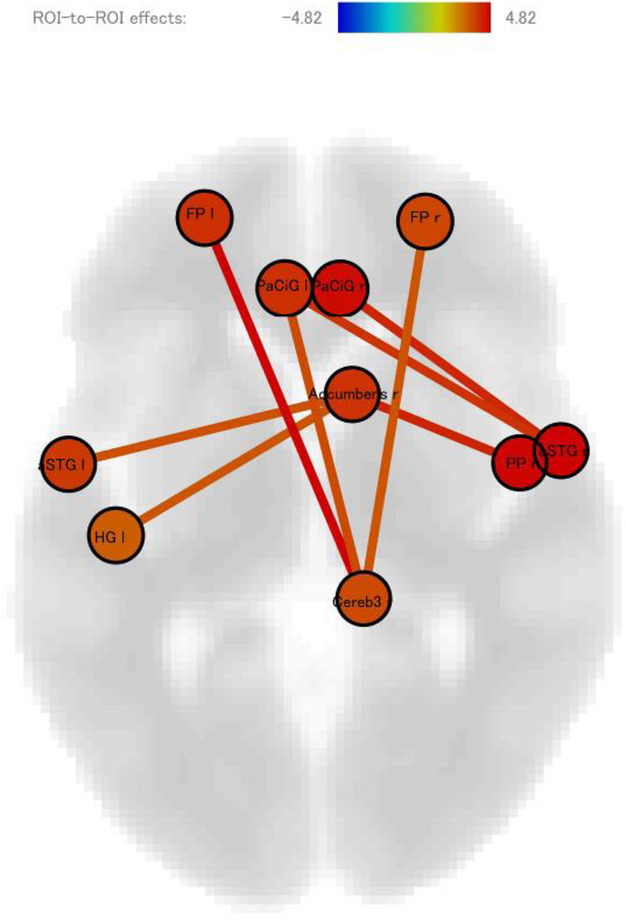
See text for description.

## P67 White-matter microstructural changes in episodic menstrual migraine compared with hormonal controls

### A. Fouto^1^, R. Nunes^1^, A. Ruiz-Tagle^1^, I. Esteves^1^, G. Caetano^1^, N. A. Silva^2^, P. Vilela^3^, R. Gil-Gouveia^4,5^, P. Figueiredo^1^

#### ^1^Institute for Systems and Robotics - Lisboa and Department of Bioengineering, Instituto Superior Técnico, Universidade de Lisboa, Lisbon, Portugal; ^2^Hospital da Luz, Learning Health, Lisbon, Portugal; ^3^Hospital da Luz, Imaging Department, Lisbon, Portugal; ^4^Hospital da Luz, Neurology, Lisbon, Portugal; ^5^Center for Interdisciplinary Research in Health, Universidade Católica Portuguesa, Lisbon, Portugal

##### **Correspondence:** A. Fouto

**Question:** Do patients with episodic menstrual migraine exhibit white-matter microstructural changes?

**Methods:** 14 women with episodic menstrual migraine (35±8yrs) were assessed during interictal phase together with 11 healthy women (29±10yrs) during a matching phase of their menstrual cycle (post-ovulation). 2D-EPI multi-shell DWI data were acquired on a 3T Siemens Vida (64-ch coil) and preprocessed using DESIGNER [1]. Diffusion tensor / kurtosis imaging (DTI/DKI) parameter maps were estimated and skeletonised [2] and histogram-metrics were computed for each subject: median, peak height, width, and value.

**Results:** Voxelwise statistical analysis [3] revealed multiple white-matter regions with lower MD and AD in patients, with no differences in FA and RD. Interestingly, migraineurs showed increased MK, AK and RK. Moreover, significant groups differences (Mann-Whitney test with Bonferroni correction) were found in histogram-metrics MD peak value, AD median and peak height and AK median. Median AK was positively associated (Spearman correlation) with disease duration but not with attack frequency and pain intensity.

**Conclusion:** Our findings extended previous reports of white-matter microstructural changes in migraineurs across multiple brain regions [4, 5]. DKI histogram-metrics showed potential as disease biomarkers.

References:

[1] Ades-Aron *et al.*, *Neuroimage*, 183, 532–543, 2018.

[2] S. M. Smith *et al.*, *Neuroimage*, vol. 31, no. 4, pp. 1487–1505, 2006.

[3] A. M. Winkler, *et al.*, *Neuroimage*, vol. 92, pp. 381–397, 2014.

[4] D. Yu *et al.*, *Cephalalgia*, vol. 33, no. 1, pp. 34–42, Jan. 2013.

[5] B. Ades-Aron *et al.*, in *Proc. Intl. Soc. Mag. Reson. Med. 27*, 2019, p. 0293.

**Fig. 1 (abstract P67).**
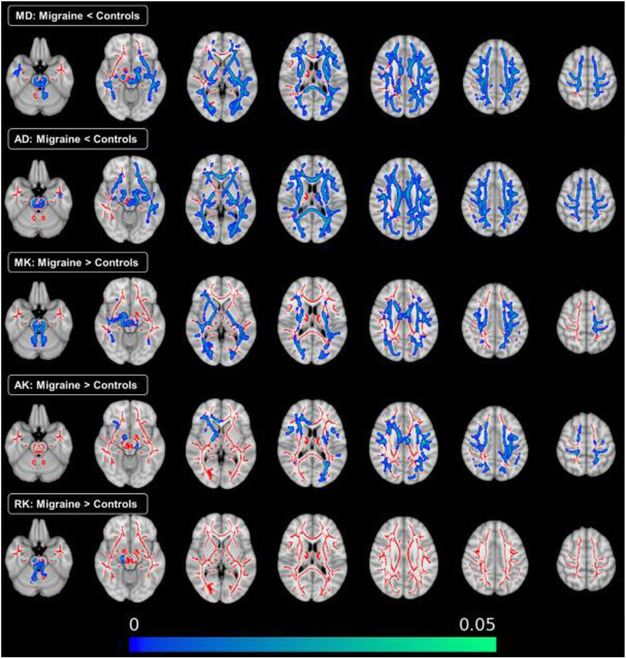
Results from voxelwise analysis of mean diffusivity (MD), axial diffusivity (AD), mean kurtosis (MK), axial kurtosis (AK) and radial kurtosis (RK) maps between controls and patients (p-value in blue-green); red represents mean FA skeleton of all subjects

## P68 Neuroimaging Utilization in Telemedicine Relative to In-Person Initial Visits for Migraine and Headache at a Tertiary Headache Center: A One-Year Analysis

### B. Torphy, M. Smith, B. Ranchero

#### Chicago Headache Center & Research Institute, Chicago, United States

##### **Correspondence:** B. Torphy

**Objective:**

During the past two and 1/2 years there has been a marked increase in the use of telemedicine in the treatment of migraine. The purpose of this study was to assess whether there was greater utilization of neuroimaging when initial patient visits for migraine and other headache conditions were conducted via synchronous video telemedicine compared to when such initial visits were conducted in-person.

**Methods:**

We performed a retrospective chart review of all initial patient visits from September 1, 2021 to August 31, 2022 at a tertiary headache center in the United States (U.S.). We compared the percentage of visits conducted via telemedicine which resulted in an order for neuroimaging to the percentage of visits conducted in-person which resulted in an order for neuroimaging.

**Results:**

A total of 398 new patient visits were conducted at the tertiary headache center, 109 (27%) of which were telemedicine, and 289 (73%) of which were in-person. Neuroimaging studies were ordered during 19.3% of visits conducted via telemedicine and during 27.3% of in-person visits.

**Conclusions:**

Neuroimaging studies were ordered 41.5% more frequently during in-person initial visits than during telemedicine initial visits for migraine and other headache conditions at a tertiary headache center. More research is warranted to determine if this phenomenon is due to selection bias, with more severe cases having a greater potential to be secondary headaches being seen in-person rather than via telemedicine, or if other factors are involved. More research is also needed to assess if this phenomenon is unique to tertiary headache centers or if it is also applicable to general neurology and primary care practice settings. As more specialties, inlcuding primary care, are caring for even more patients via telemedicine, a clearer understanding of the utilization of neuroimaging in this setting can have important clinical as well as economic implications.

## P69 Short-lasting Unilateral Neuralgiform Headache Attacks in China: A Multicenter Study of 76 Patients

### S. Zhang^1^, Z. Dong^1^, Y. Cao^1^, H. Zhao^1^, F. Yan^2^, S. Chen^3^, W. Gui^4^, D. Hu^5^, H. Liu^1^, H. Li^1^, R. Yu^6^, D. Wei^7^, X. Wang^1^, R. Wang^1^, X. Chen^1^, M. Zhang^1^, Y. Ran^1^, Z. Jia^1^, X. Han^1^, M. He^1^, J. Liu^1^, S. Yu^1^

#### ^1^the First Medical Center, Chinese PLA General Hospital, Beijing, China; ^2^Hospital, Medicine, Shandong, China; ^3^Hospital, Medicine, Hunan, China; ^4^Hospital, Medicine, Anhui, China; ^5^Hospital, Medicine, Shandong, China; ^6^Hospital, Medicine, Henan, China; ^7^Hospital, Medicine, Wuhan, China

##### **Correspondence:** S. Zhang

**Background:** Short-lasting unilateral neuralgiform headache attacks with conjunctival injection and tearing (SUNCT) and short-lasting unilateral neuralgiform headache attacks with cranial autonomic symptoms (SUNA), collectively known as short-lasting unilateral neuralgiform headache attacks (SUNHA), has hitherto not been studied sufficiently due to limited data, particularly in China. This study aimed to characterize and compare SUNCT and SUNA, as well as to aid in the identification of appropriate diagnostic and therapeutic strategies.

**Methods**: Between April 2009 and December 2021, individuals visiting a tertiary headache center or seven other headache clinics in China who were diagnosed with SUNCT or SUNA were included, compared its demographics and clinical characteristics.

**Results**: In total, 45 individuals with SUNCT and 31 individuals with SUNA were included in the study. SUNCT had a mean onset age of 37.22 ± 14.54 years while SUNA had a mean onset age of 42.45 ± 14.72 years. Both SUNCT and SUNA had a female preponderance (M:F 1:1.14 vs 1:2.10). Headache severity was moderate or severe[m1] . Qualitative descriptions of attacks were stabbing pain (44.7%), electric shock-like pain (36.8%), shooting pain (25.0%), and slashing pain (18.4%). Two individuals had an attack duration of more than 600 sec, and two had a self-reported duration of less than 1 second. No significant variations in demographic or clinical parameters were detected between the two, except for attack areas (temporal area in SUNCT, *p* = 0.017; parietal area in SUNA, *p* = 0.002).

**Conclusions**: SUNCT and SUNA should be classed as a single clinical entity, however this will require more research.

**Fig. 1 (abstract P69).**
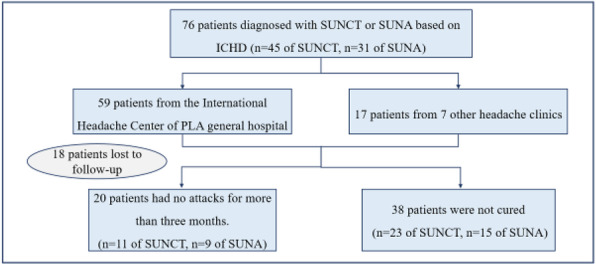
See text for description.

**Fig. 2 (abstract P69).**
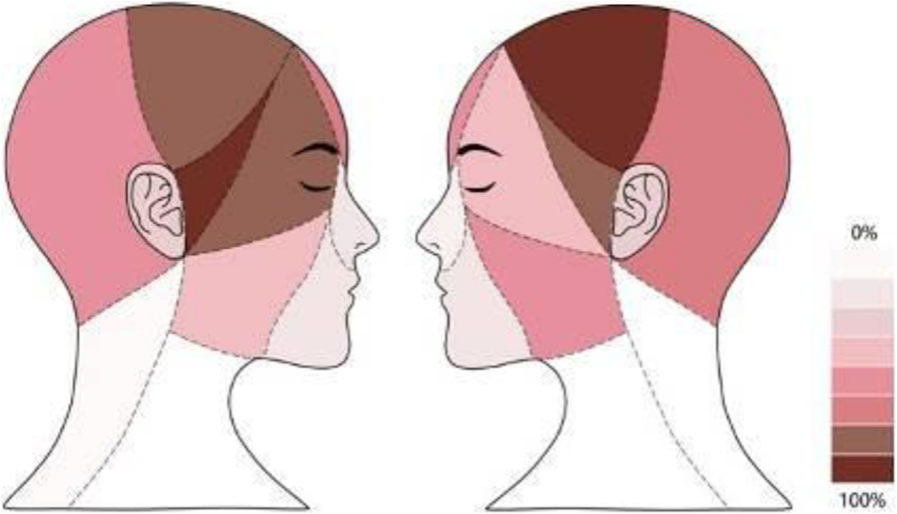
See text for description.

## P70 Primary thunderclap headache caused by micturition: a challenging diagnosis

### A. Quka^1^, S. Grabova^1^, A. Kuqo^1,2^, J. Tana^1,2^, J. Kruja^1,2^

#### ^1^University Hospital Center Mother Teresa, Neurology, Tirana, Albania; ^2^Faculty of Medicine, University of Medicine, Tirana, Albania

##### **Correspondence:** J. Tana

Introduction: Thunderclap headache (TCH) is an hiperacute and severe headache, which needs a comprehensive differential diagnosis. Primary TCH has been reported rarely, associated to cough, sexual intercourse. We report a case of a patient otherwise healthy, with recurrent episodes of Primary TCH triggered by micturition.

Case presentation: A 55 years old woman was admitted in the ER because of episodes which fulfilled TCH clinical criteria, associated to micturition. These episodes started spontaneously, in the absence of arterial hypertension or any previous medical illness and occurred several times during the day, lasting for up to ten minutes, starting upon micturition. Her physical exam was normal. Her brain CT scan, CTA and MRA were normal. At first, she was treated with anxiolytics, antidepressants, with no improvement. Her blood lab tests were unremarkable. Normal urinary metanephrines and abdominal CTscan excluded urinary bladder pheochromocytoma. She was treated with nimodipine and her situation improved visibly since the first day after starting therapy.

Discussion: The causes of such disorder, are still not well understood, but the typical clinical history of the patient and all negative tests for other causes of TCH, including urinary bladder pheochromocytoma are the main clues to the right diagnosis and treatment. Treatment was based on similar cases in the literature.

Conclusions: Primary TCH triggered by micturition is a challenging and rare diagnosis. Nimodipine seems to be a good treatment option for this rare type of primary TCH. Consent to publish had been obtained.

## P71 Suggesting a Mechanism for "Long COVID-19" Associated Headaches (Fascial Armoring as a Chronic Compartment-Like Syndrome of the Whole Body)

### S. Plaut

#### University of Nicosia, Primary Care and Population Health, Nicosia, Cyprus

**Objective**: The Coronavirus pandemic has impact on our community far beyond the acute phase, Long COVID-19 is recognized as a new medical entity and resembles fibromyalgia which, likewise, lacks a clear mechanism. Headaches and myofascial pain are common manifestations of COVID-19 and its post-acute sequalae. This work suggests a theoretical model with an organic mechanical mechanism to help explain long COVID-19 headaches and the headaches of functional psychosomatic syndromes such as fibromyalgia, based on cross-disciplinary empirical studies.

**Methods**: Systematically searched multiple keywords in MEDLINE, EMBASE, COCHRANE, PEDro, and medRxiv, inclusion/exclusion based on title and abstract, then full-text inspection. Additional literature added on relevant side topics.

**Results**: 831 records included. The theory of "facial-armoring" suggests long COVID-19 and fibromyalgia-like entities may be a disease of connective-tissue driven by myofibroblast-generated-biotensegrity-tension. This mechanism may explain fibromyalgia's pain, distribution of pain, close association with primary headache disorders, decreased pressure-pain threshold, tender spots, fatigue, autonomic abnormalities, absence of clear inflammation, silent imaging investigations, and other phenomena. "Long-COVID-19" is predicted by the model to involve fascial armoring, whereby headaches may arise in part due to a disorder of myofascial tissue, at least in a subset of patients.

**Conclusions**: long COVID-19 and fibromyalgia-like syndromes resemble a chronic-compartment-like-syndrome-of-the-whole-body and can lead to headaches due to a network of contractile fascial myofibroblasts. Treatments focusing on lifestyle modification and non-pharmacological modalities may be more beneficial in the long term. The body and the mind are one being.

Reference: Plaut S. Scoping review and interpretation of myofascial pain/fibromyalgia syndrome: An attempt to assemble a medical puzzle. PLoS One. 2022;17(2):e0263087.

## P72 Postcovid Headache after the First Wave of the Covid-19 in a Tertiary Care Headache Outpatient Clinic in Spain

### C. Trevino-Peinado, M. Babiano-Nodal

#### Hospital Universitario Severo Ochoa, Neurology, Madrid, Spain

##### **Correspondence:** C. Trevino-Peinado

Question

To describe the clinical characteristics of headache, that persists after acute SARS-Cov-2 infection in a sample that belongs to a tertiary outpatient clinic in Spain

Methods

This is a cross-sectional descriptive study. The study population were patients who had been diagnosed with COVID-19, either by PCR or by serology in the first wave. Demographic variables, history of previous headaches, pain characteristics, symptomatic and preventive treatment, COVID and post-COVID symptoms, and psychiatric comorbidity were collected.

Results

Twenty patients were included, 90% were women and the mean age was 48.5 years. 60% of the patients had a previous history of headache, being episodic migraine the most prevalent (35%). The accompanying symptoms that stood out during the acute phase were: anosmia/hyposmia 45% and pneumonia 45%. The pain that appeared during the postcovid headache was daily (60%) moderate (70%), bilateral (60%), frontal (30%) and oppressive (50%). The patients associated photophobia 90%, phonophobia 85%, osmophobia 35%. The most used preventive treatment was amitriptyline (55%) . Greater occipital nerve block with anesthetics was beneficial in 50% of the sample. 15% of the patients were treated with corticotherapy. The most reported post-COVID symptoms were anosmia (25%) and cognitive alterations (20%) and fatigue 20%. Four patients met clinical *ICHD* criteria for NDPH, and these patients had higher scores on the Hamilton anxiety scale, especially in somatic anxiety 13/28 in patients with NDPH vs 8/28 (mean of the rest of the patients). 85% of patients were responders to preventive treatment.

Conclusion: Patients who meet the NDPH criteria showed higher levels of anxiety compared to the rest of the patients. Anxiety and other psychiatric comorbidities related to the pandemic may help perpetuate the pain. Although most patients improve over time, in our sample 15% remain with persistent headache.

**Fig. 1 (abstract P72).**
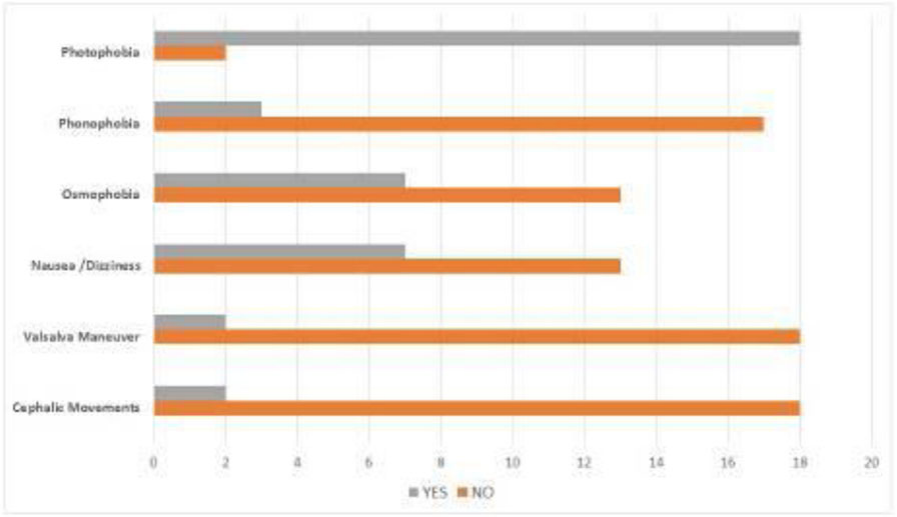
See text for description.

**Fig. 2 (abstract P72).**
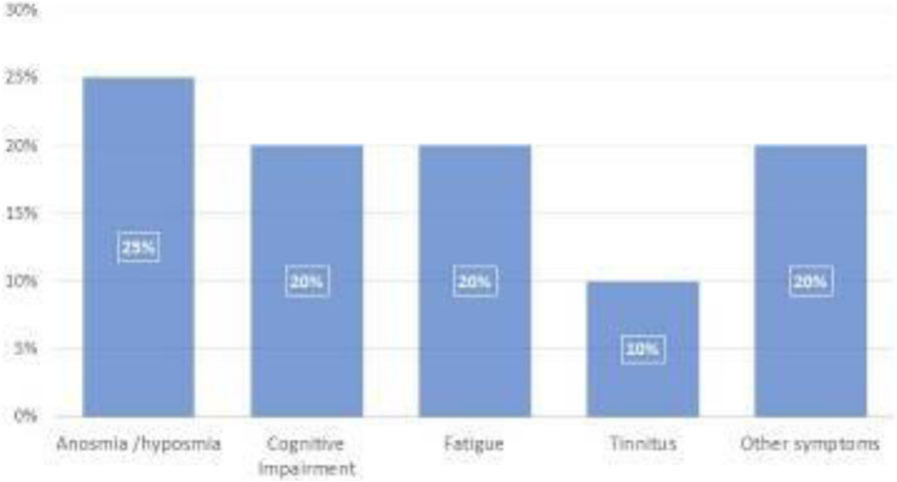
See text for description.

## P73 Clinical manifestations and impact of SARS-CoV-2 infections and vaccines in patients with migraine

### E. Caronna, L. Malgarejo, I. Elosua-Bayes, A. de la Torre Suñe, A. Alpuente, M. Torres-Ferrús, P. Pozo-Rosich

#### Vall d'Hebron University Hospital, Neurology, Barcelona, Spain

##### **Correspondence:** E. Caronna

Objective: To evaluate the clinical manifestations and the impact of (1) the infection caused by different SARS-CoV-2 variants and (2) different SARS-CoV-2 vaccines in patients with migraine.

Methods: Cross-sectional study of a cohort of patients with migraine, followed up in a Spanish Headache Unit. Through a digital questionnaire, we collected data on the characteristics of SARS-CoV-2 infection, clinical manifestations associated with vaccination against SARs-CoV-2, and their impact on migraine. We compared participants according to (1) SARs-CoV-2 variant (native strain, Alpha, Delta, Omicron) (2) type of vaccine (Pfizer, AstraZeneca, Moderna).

Results: 428 participants. COVID-19: 43.69% (187/428) had at least one infection. 83.42% (156/187) reported headache, being severe in 51.18% (81/187). The native strain and the Alpha variant were associated with a longer duration of headache compared to Delta and

Omicron (15.17, 13.11, 9.16 and 6.84 days, respectively, p=0.0003). 9% (17/187) had a reinfection, all due to Omicron, and 75% (9/12) reported headache, less intense than the one of the first infection. In 28.20% (44/156) of patients their migraine got worse since COVID-19. Vaccination: 79.43% (340/428) were vaccinated, 51.47% (175/340) reported headache. There were no differences in the proportion of patients experiencing headache between different vaccines. More severe headache was observed with AstraZeneca (p=0.0041). 21.14% (37/175) of patients reported worsening of their migraine since the vaccination.

Conclusions: SARS-CoV-2 infection and vaccines are a frequent cause of headache in people with migraine, however reinfections (with or without previous vaccination), new variants and mRNA vaccines are associated with more benign forms. SARS-CoV-2 infection and vaccines are also factors responsible for migraine worsening.

## P74 Neuralgic pain after Mucormycosis infection

### E. Muharremi, P. Djamandi, J. Kruja

#### University Hospital Center Mother Teresa, Neurology, Tirana, Albania

##### **Correspondence:** E. Muharremi

We present the case of a previously healthy 53 year old man who presented in the emergency department with drooping of the upper eyelid and difficulty seeing clearly. These symptoms started 5 days ago. The patient has diabetes type 2 and was started on steroids because he was diagnosed with COVID 2 weeks before.

On examination there was proptosis of the right eye, the pupil was not reactive to light and there was ophthalmoplegia of 3rd and 6th cranial nerves. The patient also feels paresthesias and a neuralgic pain in the V1 and V2 territory. On visual testing he can only see shadows with the right eye. The rest of the physical exam was normal.

On blood work, except for a HbA1C at 9.5 % and HSV1 IgG (+) and VZV IgG (+), other results were within the normal range. The imaging studies revealed no vascular lesions on CT angiography and brain MRI showed retroorbital inflammation on the right side, no signal restriction and the venous system appeared normal, ruling out venous sinus thrombosis.

Tissue biopsy confirmed the diagnosis of mucormycosis and treatment with Amphotericin B for 5 days followed by fluconazole. The inflammation seemed to subside, the patient still had some limitations in eye movement and the vision got better but he developed neuralgic pain that was stabbing in character in the V1 and V2 territories. We discharged the patient and started outpatient Carbamazepine therapy for a month which was unsuccessful and then switched to Pregabaline 150 mg/daily. We followed up in 3 months and then in 6 months and the patient is doing better and Pregabalin was slowly tapered. Consent to publish had been obtained.

## P75 Case report: Headache and COVID-19

### M. Xhelili, I. Zekja, J. Kruja, A. Rroji

#### University Hospital Center Mother Teresa, Neurology, Tirana, Albania

##### **Correspondence:** M. Xhelili

**Introduction**: Neurological complications are not rare in patients who survived COVID-19. On the other hand, ophthalmologists say that ocular manifestations should not be neglected.

**Case report**: We report the case of a 45- year-old male patient COVID-19 positive one month ago, without any other comorbidities, who presents in the Emergency Room in a stuporous state and bilateral midriasis after a tonic bilateral epileptic seizure. Two hours later he was lucid and oriented, without any focal neurological deficit but bilateral midriasis persisted. The patient complained severe, holocranial throbbing headache with dizziness, nausea and significant visual blurring. Ophthalmological examination reveals bilateral optic disc oedema, peripapillary hemorrhagic petechiae and venous tortuosity. Brain MRI, Angio- MRI and EEG resulted normal. The patient is treated with a high-dose of corticosteroids for three days and acetazolamide. After treatment he has no other complaints and the headache is less severe. We scheduled a follow-up with fundoscopy, after being treated with acetazolamide for 10 days.

**Discussion:** Headache is one of the frequent neurological symptoms associated with COVID-19. In the absence of evidence of infectious or vascular disease, pseudotumor cerebri should be considered. Several studies suggest that patients with COVID-19 have vascular retinal lesions ,including flame shaped haemorrhages, peripapillary petechie and acute retinal ischaemia.

**Conclusion**: Further research is needed for COVID-19 and the possible neurological or ocular complications. It is important to consider pseudotumor cerebri in a patient with severe headache after COVID-19 and to perform a fundoscopy if indicated. Consent to publish had been obtained.

## P76 Amitriptyline in the preventive treatment of persistent COVID-19 related headache: 50% responder rate and response predictors in a series of 66 patients

### D. García-Azorín, C. García-Ruiz, A. Echavarría-Íñiguez, Á. Sierra-Mencía, A. Recio García, Y. González-Osorio, C. García Iglesias, A. González-Celestino, Á. Planchuelo-Gómez, Á. L. Guerrero Peral

#### University Hospital of Valladolid, Neurology, Valladolid, Spain

##### **Correspondence:** D. García-Azorín

**Background**

Headache is a frequent symptom of coronavirus disease 2019 (COVID-19). In most cases, it is transient, but in an 8-16% it adopts a chronic pattern. Herein we describe our experience in a real-world setting with amitriptyline (AMT) and we explore the possible response predictors.

**Methods**

Patients with confirmed COVID-19 that were referred due to headache were included. The 50% responder rate of AMT was determined as the proportion of patients who presented a 50% reduction in the number of headache days per month between weeks 8-12 of treatment, compared to the month prior to the AMT onset. We conducted a regression model to evaluate which variables were associated with a higher probability of response.

**Results**

Sixty-six patients had used AMT, 92.4% female, aged 48.11 (standard deviation (SD): 11.5) years, 34.8% with prior history of migraine and 15.2% with prior history of tension-type headache. Patients had prior history of anxiety (43.9%), depression (28.8%), sleep disorders (37.9%), and other painful conditions (27.3%).

Median time between the COVID-19 infection and the AMT use was 6.1 (inter-quartile range (IQR): 4.1-9.6) months.

The median monthly frequency of headache at the moment of AMT use was 30 (IQR: 28-30). AMT was the first preventive treatment in 80.3%.

The 50% responder rate between weeks 8-12 of AMT use was 45.5%. The variables that remained associated with a 50% responder rate in the multivariate analysis were prior history of other painful conditions (odds ratio (OR): 0.142; 95% confidence interval (CI): 0.027-0.755); number of prior preventive medications (OR: 0.349; 95% CI: 0.158-0.771) and hemicranial headache (OR: 0.159; 95% CI: 0.32-0.798).

**Conclusion**

Amitriptyline was effective in treating patients with persistent post-covid-19 headache six months after the acute phase. Prior history of painful conditions, the number of preventive medications and hemicranial headache were associated with a lower probability of response.

## P77 Effect of Covid 19 Infection on Episodic Migraine Patients in Saudi Populations and Role of Vaccination.

### F. Al-Hamaid^1^, H. Younis^1^, M. Meshref^2,3^

#### ^1^King Fahad Armed Forces Hospital, Jeddah, Saudi Arabia., Neuroscience department, Jeddah, Saudi Arabia; ^2^Al-Azhar University, Neurology, Cairo, Egypt; ^3^King Saud Medical City, Riyadh, Saudi Arabia., Neurology, Riyadh, Saudi Arabia

##### **Correspondence:** H. Younis and M. Meshref

**Background:** Migraine is one of the most common medical disorders, it affects almost 1 billion patients worldwide and it is a chronic disabling disease affects women more than men. Few neurological disorders have been reported to be a possible complication of COVID 19 infection.

**AIM OF THE STUDY**: to evaluate and assess the effect of covid 19 infection on the episodic migraine patients and if there were any differences either the patients are vaccinated or not.

**STUDY DESIGN**: our study was prospective and retrospective observational study and has been done at Neuroscience department, King Fahad Armed Forces Hospital (KFAFH) in Jeddah, Saudi Arabia. We have two group of episodic migraine patients each was 30 patients; the first group was the non-vaccinated while the second one was the vaccinated group, and we assessed all the patients and reviewed their daily headache at periods of 3 months and 6 months from COVID 19 infection which was confirmed with PCR. Also, we compared it with their last daily headache before the infection.

**Results**: total 11 patients (36.6%) from the non-vaccinated group developed chronic migraine after 3 months (7 females, 4 males), The vaccinated group only 5 patients (16.6 %) developed chronic migraine (3 females, 2 males) **(p value 0.0014)**. while only 5 patients (16.6%) developed chronic migraine after 6 months (3 females, 2 males) in the non-vaccinated group while the vaccinated one only 3 patients (10 %) developed chronic migraine (2 females, 1 males) (**p valuen0.0001**).

**Conclusion:** COVID 19 infection has negative effects on the episodic migraine patients. However, the covid vaccination has a protective role against its conversion to chronic migraine.

**Key Words**: Episodic migraine, Chronic migraine, COVID 19 infection, COVID 19 Vaccination.

**Table 1 (abstract P77).**
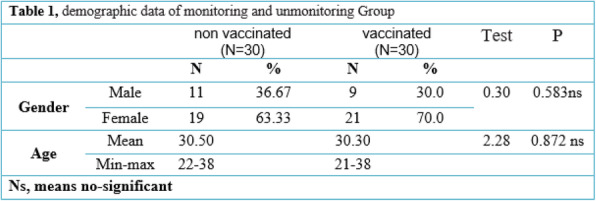
Demographic data of monitoring and unmonitoring group

**Table 2 (abstract P77).**
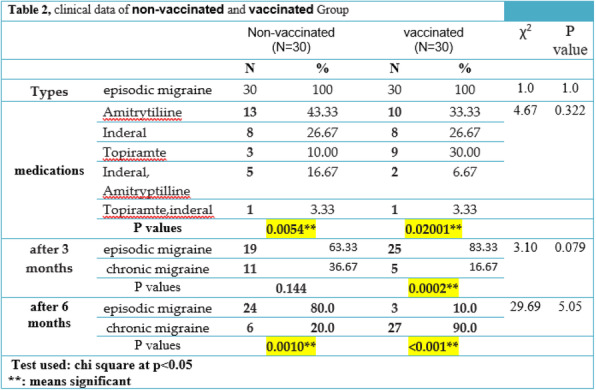
Clinical data of non-vaccinated and vaccinated group

## P78 Occipital neuralgia after COVID-19 vaccination: a case report

### S. Malheiro, D. Costa, R. Varela

#### University of Porto, Neurology, Porto, Portugal

##### **Correspondence:** S. Malheiro

**INTRODUCTION**

Headache is the most commonly reported neurological adverse effect after COVID-19 vaccination, with mild to moderate headaches being reported in 25-52% of patients after BNT162b2. Herein, we describe the first case reported of an occipital neuralgia after BNT162b2.

**CLINICAL CASE**

A 39-year-old caucasian woman with no previous story of headache, was admitted to our hospital with two weeks of a bilateral paroxysmal stabbing shock-like pain beginning in the occipital zone. Six days before, she received the first dose of Pfizer-BioNtech vaccine against SARS-CoV-2, with fever reported in the first day after the administration of this vaccine. She had never experienced any kind of headaches after other vaccinations. The pain was a bilateral stabling shock-like pain, severe (intensity of 8/10), with a short duration (few seconds) and spontaneously initiated or triggered by touching or brushing the hair, appearing many times per day and, over the time, in the period between the shocks, she starts to feel a dull pain in the vertex and nuchal region, with concomitant dysesthesia in these zones. On examination, pressure over the occipital nerves revealed local tenderness and elicited a paroxysm of pain. A probable occipital neuralgia was considered, and bilateral occipital blockage had been performed in the emergency department, with significant relief of the pain. It was started gabapentin (up to 300 mg) and amitriptyline (up to 25 mg). Neuroimagiology (brain CT, brain and cervical MRI) was unremarkable. Since then, the patient has been re-evaluated in consultation, with 3-month intervals, with administration of large occipital nerve blockages, with significant improvement in shocking pain, and with progressive improvement in constant dull pain over the months.

**CONCLUSION**

Two cases of trigeminal neuralgia after COVID-19 vaccination had already been reported, however this is the first time that a case of occipital neuralgia after this vaccine is described. Consent to publish had been obtained.

## P79 Impact of SARS-CoV2 vaccine on migraine course in patients receiving CGRP monoclonal antibodies

### G. Vaghi^1,2^, B. Guindani^3^, R. De Icco^1,2^, F. Cammarota^1,2^, C. Tassorelli^1,2^, F. S. Robustelli Della Cuna^3,4^, L. Gervasio^4^, G. Sances^2^

#### ^1^University of Pavia, Department of Brain and Behavioral Sciences, Pavia, Italy; ^2^IRCCS Mondino Foundation, Headache Science & Neurorehabilitation Center, Pavia, Italy; ^3^University of Pavia, Department of Drug Sciences, Pavia, Italy; ^4^IRCCS Mondino Foundation, Pharmacy Service, Pavia, Italy

##### **Correspondence:** G. Vaghi

**Questions** COVID-19 vaccines reduce the risk of death and major sequelae related to SARS-CoV2. Despite proven safety, they present adverse events among which headache is one of the most frequently reported. We aim to evaluate vaccine impact on headache frequency in migraine patients receiving monoclonal antibodies targeting CGRP pathway (anti-CGRP mAbs)

**Methods** We enrolled 139 migraine patients actively treated with one of the 3 anti-CGRPmAbs currently available in Italy (erenumab, galcanezumab or fremanezumab). We collected: i) clinical and demographic data; ii) self-perceived headache changes after the 1^st^ and 2^nd^ vaccine doses (frequency, intensity and acute drug's efficacy); iii) monthly headache and migraine days (MHD and MMD), days and doses of acute drug intake in the month before(m_pre_) and after(m_post_) vaccine administrations through paper diaries

**Results** The dataset is formed by 100 migraine patients who received COVID-19 vaccine(fig. 1) during mAbs treatment (73% females, migraine history 36.7±12.2yrs). At baseline 96% of patients had a diagnosis of chronic migraine (86.5% of them also had medication overuse headache). 13% of the patients reported a *subjective* worsening of headache frequency and intensity. Still, headache diaries demonstrated an *objective* reduction in MHD, days and doses of acute drug intake after the 1^st^ and 2^nd^ vaccine doses. All parameters showed a reduction trend without reaching significance, except for MHD after the 1^st^vaccine dose (MHD1^st^dose m_pre_ 14.6±9.8;m_post_13.2±9.7, *p=0.01;* 2^nd^dose m_pre_14.0±10.9; m_post_13.0±9.7,p=0.15)(fig. 2). No correlation was found between demographic or baseline headache features and subjective headache worsening

**Conclusions** In our cohort of migraine patients treated with anti-CGRPmAbs, COVID-19 vaccination did not induced any worsening in migraine characteristics. Our data suggest that mAbs treatment may prevent headache worsening frequently reported in patients with migraine exposed to COVID-19 vaccination.

**Fig. 1 (abstract P79).**
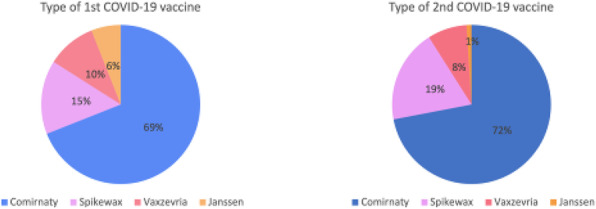
Type of COVID-19 vaccine administered as 1^st^ and 2nd dose

**Fig. 2 (abstract P79).**
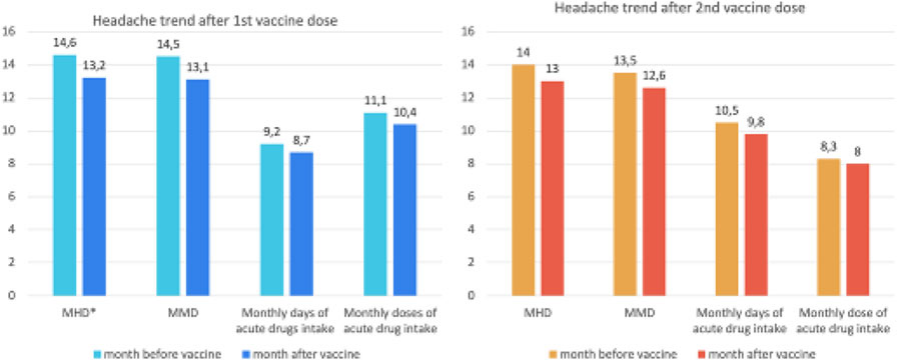
Headache features in the month before and after the 1^st^ and ^2nd^ dose of COVID-19 vaccine. MHD: monthly headache days, MMD: monthly migraine days

## P80 The Regional Outreach Programme of the International Headache Society in sub-Saharian Africa: partnership with the DREAM program and results from the first survey on headache training.

### D. Martinelli^1,2^, C. Tassorelli^1,2^, R. H. Jensen^3^, M. Matharu^4^, V. Tolno^5^, D. Thole^6^, G. Guidotti^7^, M. C. Marazzi^8,9^, M. Leone^10,9^

#### ^1^IRCCS Fondazione Mondino, Headache Science and Rehabilitation Center, Pavia, Italy; ^2^University of Pavia, Brain and Behavioral Science Department, Pavia, Italy; ^3^University of Copenhagen, Rigshospitalet, Neurology, Copenhagen, Denmark; ^4^UCL Queen Square institute of Neurolog, headache and facial pain group, London, United Kingdom; ^5^DREAM Program, Blantyre, Malawi; ^6^DREAM Program, Balaka, Malawi; ^7^Azienda Sanitaria Locale Roma 1, Rome, Italy; ^8^Libera Università Maria SS Assunta, Rome, Italy; ^9^DREAM Program, Rome, Italy; ^10^IRCCS Besta Foundation, UO Neuroalgology, Milan, Italy

##### **Correspondence:** D. Martinelli

**Background**

Neurology is one of the least represented medical specialties in the African continent. In Sub-Saharan Africa (SSA) neurologic care is mostly delivered by non-physician healthcare professionals (np-HCPs). Here we report the results of a survey conducted to evaluate training, needs, and knowledge about headache of a representative group of np-HCPs in Malawi.

**Methods**

The Regional Outreach Programme of the International Headache Society (ROPE-IHS) promotes headache education worldwide. In SSA ROPE-IHS is developing an education program in collaboration ith the Disease Relief through Excellent and Advanced Means (DREAM) program, a recognized primary care health programme that has activated healthcare facilities in 10 SSA countries. In order to customize the training program on the specific needs of local HCPs, we performed a survey among np-HCPs working in the different DREAM centres in MW. For this purpose, we created a specific questionnaire to test the level of preparation and interest on headache.

**Results**

Fifty-one healthcare workers (23 women) participated (median age 37 years; median duration of education 3 years): 26 were clinical officers, 6 nurses, 5 clinicians, and 14 had other roles. All respondents agreed on the importance to receive education and training on headache; 84% never attended a full headache course. Past headache courses were mainly delivered by np-HCPs.

Only 2% of headache patients are referred to doctors while the vast majority (84%) are seen by clinical officers or local healers (57 and 27%, respectively).

**Conclusions**

Insufficient education among healthcare providers is the main barrier to care for headache patients in SSA. A partnership between international societies and recognized local providers is a valid tool to provide bottom-up tailored education on headache at primary care level in difficult contexts as in SSA. The partnership also accomplishes the WHO *Intersectoral Global Action Plan.*

## P81 A Case of Migrainous Infarction in a 40-Year-Old Male with a History of Spontaneous Coronary Artery Dissection

### E. Troy, S. Quigley, A. M. Ryan

#### Cork University Hospital, Neurology, Cork, Ireland

##### **Correspondence:** E. Troy

**Objective**

Spontaneous coronary artery dissection (SCAD) is associated with a history of migraine, though this association is poorly understood. It classically affects young and middle-aged women and is rare in men.(1) SCAD patients with migraine tend to be younger at the time of SCAD. Migrainous infarction is an infrequent, but specific type of ischemic stroke developing during an attack of migraine with aura.(2) We present a case of a migrainous infarction in a man with a history of SCAD a decade earlier.

**Background**

A 40-year-old man developed episodes of visual aura which was associated with slurred speech and left-sided limb weakness, lasting 3-4 minutes before resolving. A second episode occurred and symptoms persisted for over an hour. A unilateral throbbing headache followed. His medical history was significant for migraine with aura and a spontaneous coronary artery dissection age 29-years. There was a family history of migraine and TIA. He Blood pressure, neurological and cardiac examinations were normal.

**Results**

Infectious and inflammatory markers were normal, and electrocardiogram showed normal sinus rhythm. CT brain was unremarkable. MRI Brain revealed a small area of diffusion restriction in the right parietal lobe along the postcentral sulcus consistent with an acute infarction. Carotid dopplers, echocardiogram and holter monitor were unrevealing. CT Angiogram found no evidence of vessel abnormality.

**Conclusions**

Migrainous infarction has not previously been described in a patient with a history of SCAD. This occurred in a male, which is also unusual as both SCAD and migraine predominately affect women. Consent to publish had been obtained.

References

1.Kok SN, Hayes SN, Cutrer FM, Raphael CE, Gulati R, Best PJM, et al. Prevalence and Clinical Factors of Migraine in Patients With Spontaneous Coronary Artery Dissection. J Am Heart Assoc. 2018;7(24):e010140

2.Laurell K, Lundström E. Migrainous infarction: aspects on risk factors and therapy. Curr Pain Headache Rep. 2012;16(3):255-60

**Fig. 1 (abstract P81).**
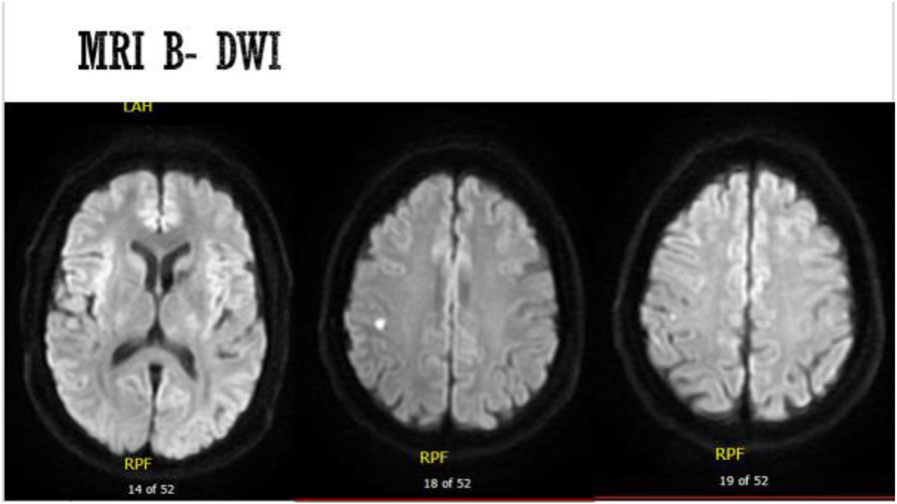
See text for description.

**Fig. 2 (abstract P81).**
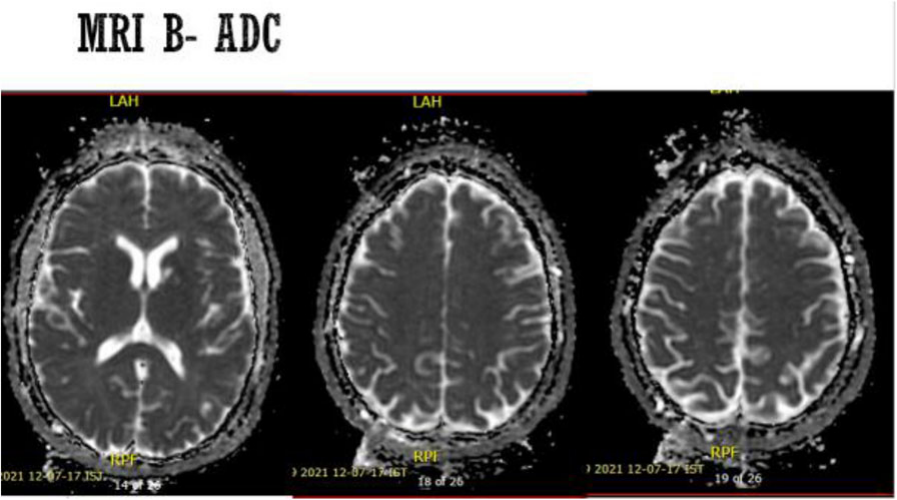
See text for description.

## P82 Efficacy of surgical treatment in patients with trigeminal neuralgia secondary to multiple sclerosis – a prospective study of 18 cases with evaluation of outcome and complications by independent evaluators

### J. Worm^1^, N. Noory^1^, E. A. Smilkov^2^, T. B. Heinskou^1^, A. S. S. Andersen^1^, J. B. Springborg^3^, P. Rochat^3^, J. L. Frederiksen^4,5^, L. Bendtsen^1^, S. Maarbjerg^1^

#### ^1^Danish Headache Center, Department of Neurology, Rigshospitalet, Glostrup, Denmark; ^2^Rigshospitalet Glostrup, Department of Radiology, Glostrup, Denmark; ^3^Rigshopitalet Blegdamsvej, Department of Neurosurgery, Copenhagen, Denmark; ^4^University of Copenhagen, Copenhagen, Denmark; ^5^Rigshospitalet Glostrup, Department of Neurology, Glostrup, Denmark

##### **Correspondence:** J. Worm

**Background**

Patients with multiple sclerosis (MS) are at increased risk of developing trigeminal neuralgia. Medical treatments for trigeminal neuralgia (TN) secondary to MS (MS-TN) is based on weak evidence and have low efficacy and tolerability issues. Patients are often referred to neurosurgery, but the scientific evidence regarding efficacy and complications in these patients is scarce and inconsistent. We aimed to assess outcome and complications after neurosurgical intervention for MS-TN.

**Methods**

Patients with MS-TN who underwent microvascular decompression (MVD), glycerol rhizolysis or balloon compression were prospectively included from 2012 to 2019. Preoperatively, we obtained clinical characteristics and performed a 3.0 Tesla MRI in all patients. Postoperative follow-up visits at 3, 6 and 12 months were conducted by independent assessors. Efficacy was assessed using a modified version of the Barrow Neurological Institute score and complications were graded into major and minor complications.

**Results**

We included 18 patients with MS-TN. Of seven patients treated with MVD, 5 (71%) patients had an excellent or good outcome, one (14%) patient had treatment failure and one (14 %) patient had a fatal outcome. Three (43%) patients had major complications and two (29%) patients had minor complications. Of eleven patients treated with percutaneous procedures, 6 (55%) patients had an excellent or good outcome. Major complications were seen in two (18%) patients and two (18%) patients had minor complications.

**Conclusions**

Our findings shows that surgical treatment with MVD in MS-TN has a higher complication rate and an outcome inferior to MVD in primary TN. Hence, we recommend the use of percutaneous procedures in MS-TN patients and MVD only in selected patients using advanced neuroimaging to maximize the probability of an excellent outcome.

Figure. Outcome (top) and complications (bottom) in primary trigeminal neuralgia (TN) and TN in multiple sclerosis (MS-TN).

**Fig. 1 (abstract P82).**
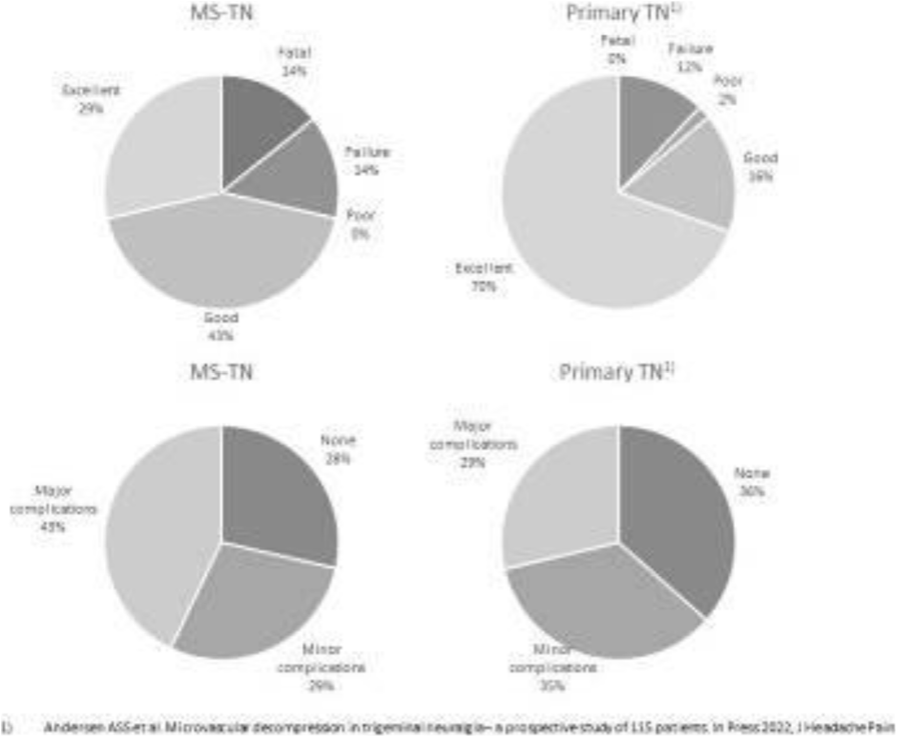
See text for description.

## P83 Acute treatment of classic, secondary and idiopathic trigeminal neuralgia in the emergency room. 43 patients series

### T. Mederer, M. Borrell-Pichot, R. Sainz-Torres, G. Olmedo-Saura, A. Martinez-Viguera, R. Collet-Vidiella, J. M. Fernandez-Vidal, C. Toscano Prats, R. Belvís, N. Morollón

#### Hospital de la Santa Creu i Sant Pau, Neurology, Barcelona, Spain

##### **Correspondence:** T. Mederer

OBJECTIVES

The aim is to describe the acute management of status due to neuralgia in patients with trigeminal neuralgia (TN) who come to the emergency room.

METHODS

Observational, descriptive, and retrospective study in which all visits to the emergency room due to *neuralgia status* of all patients with TN followed up in a tertiary hospital were collected. *Neuralgia status* was defined as TN flares in patients receiving specific pharmacological treatment. For each status episode, the rescue treatment received, the modifications in the basal treatment and the need for hospitalizations and its duration were collected.

RESULTS

From a sample of 231 patients diagnosed with TN, 43 presented at least one status episode: 16 patients diagnosed with idiopathic TN, 16 with classic TN and 11 with secondary TN (classified according to the ICHD3). The mean age was 44 years old, and the total number of statuses was 89.

Concerning the rescue treatment used, 40% of patients received first-step analgesia, 45% opioids, 18% corticosteroids, 11% antiepileptics and 3% gabapentinoids. Opioids seemed to be the more effective ones (75%), followed by antiepileptics (60%) and first-step analgesia (50%).

Hospitalization was required in 20% of status patients, with a mean stay of 8.1 days, during which optimization of the basal treatment was performed: the most used drugs being carbamazepine, lamotrigine, lacosamide and gabapentinoids. 8 patients underwent invasive procedures (3 microvascular decompression, 2 percutaneous surgeries, and 3 trigger-point blocks).

CONCLUSIONS

18.6% of patients presented at least one status episode. The most administered rescue drugs were first-step analgesia and opioids and the most effective ones seemed to be opioids and antiepileptics. 20% of status required hospitalization and 8 patients required invasive procedures.

## P84 Oral lacosamide for the treatment of refractory trigeminal neuralgia: a retrospective analysis of 86 patients

### R. Tena-Cucala^1^, A. Muñoz-Vendrell^1,2^, S. Campoy^1,3^, J. Prat^1^, S. Martinez-Yelamos^1,2^, M. Huerta-Villanueva^1,3^

#### ^1^Neurology Department, Headache Unit, Hospital Universitari de Bellvitge-IDIBELL, Universitat de Barcelona, Neurology, Barcelona, Spain; ^2^University of Barcelona, Neurology, Barcelona, Spain; ^3^Neurology Department, Hospital de Viladecans, Viladecans, Spain

##### **Correspondence:** R. Tena-Cucala

**Introduction & Objectives**

First-line treatment in trigeminal neuralgia (TN) is limited to carbamazepine (CBZ) and oxcarbazepine (OXC), but inefficacy or intolerability is frequent and valid alternatives are scarce. Lacosamide (LCM), a novel sodium channel blocker, has been proposed as an alternative option in a few case reports. Our objective is to describe a series of patients who received oral LCM after first-line treatment failure.

**Methods**

In this retrospective analysis, we included patients who were prescribed LCM for TN pain control and were followed at our tertiary hospital. We recorded demographic information, TN characteristics and treatment data. Primary endpoints were pain relief and adverse events. Secondary endpoints were absence of pain and time to pain worsening.

**Results**

86 patients were included, with a mean age of 61.9 ±15.6 years, 62.8% women. Median time since TN diagnosis was 4.44 years (range 48 years). Pain was purely paroxysmal in 66.3%. Etiology was secondary in 18.6%. 88.37% of patients had previously been treated with CBZ or OXC. Mean daily initial LCM dose was 143 ±63.32mg, and mean daily maintenance dose was 228.5 ±113.64mg. 53.49% of patients concomitantly received CBZ or OXC. Pain relief was accomplished in 74.4% of cases, with a 31.4% of adverse events (mainly dizziness in 18.6%, somnolence in 4.65% and instability in 3.48%). One patient presented a first-degree cardiac blockade. Absence of pain was reported in 33.7%. Pain worsening was recorded in 43% or patients, with a mean time of 441.26 ±478.93 days after LCM initiation.

**Conclusions**

LCM could be an effective and relatively safe treatment of refractory pain in TN, after first-line treatments failure. Further prospective studies are needed.

## P85 Post COVID-19 Vaccine Trigeminal Neuralgia in a Patient with Melkersson-Rosenthal Syndrome

### B. G. Turk^1^, A. Mengi^2^, U. Uygunoglu^1^

#### ^1^Istanbul University Cerrahpaşa School of Medicine, Neurology, Istanbul, Turkey; ^2^Istanbul University Cerrahpaşa School of Medicine, Algology, Istanbul, Turkey

##### **Correspondence:** B. G. Turk

Introduction

Melkersson-Rosenthal syndrome (MRS) is a disease characterized by recurrent peripheral facial paralysis, orofacial edema and fissured tongue triad. Cranial neuropathies have also been reported after COVID vaccines. Here, we present a 53-year-old female patient with this rare syndrome who had new-onset trigeminal neuralgia after COVID vaccination.

Case Report

53-year-old female patient presented with itchy, burning like pain over the right mandibular region of her face for ten days. Pain was occurring as 10-20 attacks per day lasting seconds. She reported that the new onset pain had started 2 days after the mRNA COVID vaccine. Concerning her medical history, she was diagnosed with MRS which had three previous episodes of peripheral facial paralysis and recurrent orofacial edema. Cranial imaging, serum and cerebrospinal fluid analysis were normal. She was diagnosed as trigeminal neuralgia (TN) and symptomatic treatment with gabapentin 1200 mg/day had started. She was pain-free for the past month.

Discussion

Cranial nerve involvement in MRS can be seen and the facial nerve is the most commonly involved one. Rarely, other cranial nerves may also be affected with complaints of impaired taste and hearing or trigeminal neuralgia. Cranial neuropathies have also been reported after COVID vaccines, and there are a few cases of post-vaccination trigeminal neuralgia in the current literature. The time interval between vaccination and TN in the reported cases was approximately 7-14 days. However, in our case, the onset of TN was quite rapid, and we interpreted this situation as the combination of MRS and COVID vaccine may facilitate the onset of TN. Consent to publish had been obtained.

References

1- Kaya A, Kaya SY. A case of trigeminal neuralgia developing after a COVID-19 vaccination. J Neurovirol. 2022 Feb;28(1):181-182. doi: 10.1007/s13365-021-01030-7. Epub 2021 Dec 6. PMID: 34870807; PMCID: PMC8647511.

## P86 Vagoglossopharyngeal neuralgia as cause of syncope due to atrio-ventricular block: one case report

### O. García Pazos^1^, L. Ramos Rúa^1^, J. Armesto Rivas^2^, N. Rojo Prieto^2^, C. Tuñas Gesto^1^, P. Santamaria Montero^1^, R. Alonso Redondo^1^, L. Álvarez Fernández^1^, F. Brañas Fernández^1^, C. F. Da Silva França^1^, A. Doporto Fernández^1^, A. Fernández Cabreira^1^, M. Guijarro del Amo^1^, T. Rodríguez Ares^1^, M. Rodriguez Rodríguez^1^, G. Vicente Peracho^1^, R. Pego Reigosa^1^

#### ^1^Lucus Augusti University Hospital, Neurology, Lugo, Spain; ^2^Lucus Augusti University Hospital, Cardiology, Lugo, Spain

##### **Correspondence:** O. García Pazos and L. Ramos Rúa

Question

Glossopharyngeal neuralgia is a rare facial pain syndrome characterized by painful paroxysms in the sensory distribution of the glossopharingeal nerve. Association with cardiac syncope is even rarer and when it happens it is termed vagoglossopharyngeal neuralgia (VN). We present a case of idiopatic VN with secondary complete atrio-ventricular (AV) block and symptomatic syncopes

Methods

A 66-year-old man with no medical history of interest, presented to Emergency department with high intensity pain in left preauricular region and behind mandibular angle. He described the pain as electrical discharges. These attacks were followed by syncopes of 20-30 seconds duration. Furthermore, during some of these episodes he presented limb shaking without tongue bite or sphincter relaxation.

Cardiac monitoring showed complete AV block followed by sinus bradycardia at 30 beats per minute and subsequent recovery of heart rate.

Between these episodes the patient was asymptomatic. His general and neurological examination was normal.

Results

He was admitted to Intensive Care Unit for monitoring and treatment with aleudrine was started. Chest radiography, echocardiogram, brain magnetic resonance imaging, computed axial tomography of the neck and electroencephalogram were performed with normal results.

Structural heart disease was ruled out. AV-block secondary to increased vagal tone due to NV was diagnosed. Treatment with aleudrine was stopped and carbamazepine was started, after which the patient had no new episodes of neuralgia or cardiac block.

He was discharged and after five months he has kept on treatment and has had no new episodes of neuralgia or syncope.

Conclusions

VN is a rare syndrome. It could be associated with syncopes due to AV-block. Pharmacological therapy is the initial step to the VN treatment, but surgery could be needed in refractory cases. Pacemarker could be required to treat AV-block in selected patients. Consent to publish had been obtained.

## P87 Intravenous lidocaine infusions for trigeminal neuralgia acute exacerbations – preliminary results from a prospective study

### A. L. Neves^1^, M. J. Pinto^1^, S. Silva^2^, A. Gomes^3^, A. Costa^1^, P. Abreu^1^

#### ^1^Centro Hospitalar Universitário de São João, Neurologia, Porto, Portugal; ^2^University of Porto, Porto, Portugal; ^3^Centro Hospitalar Universitário de São João, Anestesiologia - Dor Crónica, Porto, Portugal

##### **Correspondence:** A. L. Neves

**Question:** Trigeminal neuralgia (TN) is a chronic neuropathic pain disorder. Some patients experience severe acute exacerbations, often requiring therapeutic escalation. The efficacy of intravenous (IV) lidocaine infusions in this setting was reported in few small studies, however, there is not a uniform protocol of administration. Our aim was to evaluate the efficacy of IV lidocaine infusion for TN acute exacerbations based on a predefined protocol in a Chronic Pain Outpatient Clinic.

**Methods:** We included all adult patients admitted to our Outpatient Clinic for treatment of acute exacerbation of TN with IV lidocaine. IV lidocaine (1mg/kg) was infused over 60 minutes in each session. Vital signs were measured during and after each infusion. Pain was evaluated before initiating treatment and after the last infusion using the Numeric Pain Scale (NPS), the Pain Catastrophizing Scale (PCS), the Pain Disability Index (PDI) and the Hospital Anxiety and Depression Scale (HADS). Patient scores were compared using the paired-samples t-test and Wilcoxon. P-values <0.05 were considered statistically significant.

**Results:** Eight patients completed 12 sessions (3/week), 5 (62.5%) female, with a mean age of 61 ± 14 years. Significant differences were found between baseline and the end of the IV lidocaine treatment in NPS (before 9 ± 5 vs after 4.13 ± 2.42, p=0.02). No significant differences were found in PCS (40.88 ± 9.08 vs 38.88 ± 6.79, p=0.41), PDI (45 ± 24 vs 30.88 ± 22.71, p=0.40) and HADS (anxiety 10.50 ± 4.24 vs 9.50 ± 3.59, p=0.51; depression 11.50 ± 2.56 vs 10 ± 4.44, p=0.26). No adverse effects were reported.

**Conclusion:** Patients showed improved scores in all applied scales after IV lidocaine treatment, although not statistically significant except for the NPS. In order to confirm these preliminary results we are still recruiting patients to enlarge our sample.

## P88 Descriptive study of a series of patients diagnosed with secondary trigeminal neuralgia. 39 patients series.

### R. Sainz-Torres, T. Mederer, M. Borrell-Pichot, A. Martinez-Viguera, G. Olmedo-Saura, R. Collet-Vidiella, J. M. Fernandez-Vidal, C. Toscano Prats, R. Belvís, N. Morollón

#### Hospital de la Santa Creu i Sant Pau, Neurology, Barcelona, Spain

##### **Correspondence:** T. Mederer

Objectives

The aim of the study is to describe the most frequent etiologies and the pain qualities in a sample of patients with secondary trigeminal neuralgia (TN).

Methods

Observational, descriptive, retrospective, and single-center study that includes all secondary TN followed up for a mean of 5 years in the Headache Unit of a tertiary hospital.

Results

39 patients diagnosed with secondary TN with a mean age of 54 years were included. The main etiologies found in our sample were tumors 41% (meningioma 38%, squamous cell tumor 25%, schwannoma 25% and glioma 13%) and multiple sclerosis (MS) 41% (88% due to pontine plaques, only 8% active plaques). The remaining 18% were related to nonspecific demyelinating lesions without MS criteria, ischemic lesions, post-surgery, pachymeningitis, hemangioma, and Arnold-Chiari malformation.

Regarding the pain pattern, most of the patients suffered Burchiel type 1 neuropathic pain (77%) and the involvement of more than one branch predominated: 2 branches in 41% of the patients and three branches in 21%. The most affected branch was V3 (77%), followed by V2 (67%).

The most used drugs were carbamazepine and its derivatives (79%), with an effectiveness of 45% for more than two years, followed by gabapentinoids (67%), with an effectiveness of 11%, and lacosamide (41%), with an effectiveness of 0%. 28% of the patients underwent percutaneous surgery and 10% underwent microvascular decompression surgery.

Conclusions

The most frequent causes of secondary TN were intracranial tumors and MS. Secondary TN more often affects 2 or more trigeminal branches. The most effective drugs were carbamazepine and its derivatives.

## P89 Medical Cannabis in Senior Patients: Therapeutic Effects/Side Effects. Behavioral and Imaging Correlations

### M. Nicolodi^1^, L. Sicuteri Di Puccio^2^, M. S. Pinnaro^3^, M. Cerboneschi^4^

#### ^1^Foundation Primary Headache and Stress, Research, Florence, Italy; ^2^University of Padua, Neuropsychology, Padua, Italy; ^3^University of Florence, Science of Nutrition, Florence, Italy; ^4^University of Florence, Biology, Florence, Italy

##### **Correspondence:** M. Nicolodi

**BACKGROUND**

Cannabinoid receptors CB1 and CB2 upregulation occurs in pain processing, CB2 agonists suppress neuropathic symptoms. Trigeminal neuralgia (TN), a pain syndrome of neuropathic origin, can be or become refractory to anticonvulsants. Observation concerns cannabinoids in refractory TN of senior patients.

**METHOD**

**Drug:** fixed combination 22%Tetrahydrocannabinol (THC), a CB1 CB2 receptor partial agonist +>1%Cannabidiol (CBD) trafficking with CB1 and CB2 receptors

**THC/CBD Dose finding:** 200 mg/day effective dose

**Administration Route**: Sublingual to circumvent gastrointestinal adaptation, hepatic first-pass metabolism and producing higher plasma concentration. Non decarboxylated form was chosen to allow entourage effect

**Plan**: Explanatory-Controlled partly Covered, 3-months treatment, 1-month follow-up

**Participants** suffered from TN refractory (less than 25% relief) to anticonvulsants. They are part of an observation regarding refractory TN. Hereinafter observation concerns Group A (23 males, 5 females; 57.3±2.2SD) receiving fixed combination THC-/CBD and Group B (20 males, 6 females; 56.9±1.9SD) re-testing carbamazepine 900mg/day. Rescue treatment: lidocaine 2% 10 ml i.v.

**RESULTS**

**Theraputic Effects, Side effects**

Pain relief THC/CBD vs Baseline and Carbamazepine treatment p<0.0001

Rescue n=13 Carbamazepine, n=1 THC/CBD

Withdrawal n=7 Carbamazepine, n=2 THC/CBD

Relapse during follow-up n=4 THC/CBD, n=8 Carbamazepine

Somnolence, Drowsiness n=9 Carbamazepine, n=11 females THC/CB first week

Memory (Randt Memory Battery) impairment n=20 Carbamazepine p< 0.5 vs baseline Retrieval amelioration: THC/CBD n=27 p>0.01 vs baseline

Cerebral fMRI changes within the temporal lobe n=3 males Carbamazepine

Sexual intercourses increase n=3 males THC/CBD

**CONCLUSIONS**

Outcomes suggest either effectiveness and safety of 22%THC+>1%CBD in refractory TN sufferers 50-65 years old or a role for CB1, CB2 receptors in TN. Larger casuistry is needed to guarantee efficacy

## P90 Differential expression of the calcitonin receptor, CGRP and amylin in the trigeminal and dorsal root ganglia

### T. Rees^1^, Z. Tasma^1^, C. Walker^1^, D. Hay^2^

#### ^1^University of Auckland, School of Biological Sciences, Auckland, New Zealand; ^2^University of Otago, Department of Pharmacology and Toxicology, Dunedin, New Zealand

##### **Correspondence:** T. Rees

**Objective:** The trigeminal ganglia (TG) and dorsal root ganglia (DRG) are anatomically important sites for pain, containing neuropeptides and receptors that modulate pain transmission. The neuropeptides, calcitonin gene-related peptide (CGRP) and amylin have been linked to migraine. They are potent agonists of the AMY1 receptor, a heterodimer of the calcitonin receptor (CTR) and RAMP1. Co-expression of the CTR and CGRP has been reported in the TG, with little or no amylin observed. In the DRG, both peptides have been reported; however, their distribution relative to the CTR is unknown. This suggests that there may be differences in the relative abundance of each peptide between these two ganglia and which peptides might signal via CTR in each location. This study aimed to determine the relative distribution of the CTR with CGRP and amylin in the DRG and compare this to the TG.

**Methods:** In combination with neural markers, specific antibodies against CTR, CGRP and amylin were applied to mouse, rat, and human C1/2 DRG to investigate distribution. Data were compared to our prior TG data using the same conditions.

**Results:** In the DRG, CGRP-like immunoreactivity (LI) and amylin-LI were present in distinct and overlapping neurons, indicating occasional co-expression of the peptides. CTR-LI was present in neurons which expressed CGRP or amylin alone, as well as neurons which expressed both peptides. Co-staining was uncommon with an A-fibre marker, NF200, indicating that CTR-LI, CGRP-LI and amylin-LI were primarily in C-fibre neurons.

**Conclusions:** The expression of CGRP and CTR were similar between the DRG and TG. However, unlike the TG, abundant amylin expression and co-localisation with CTR was observed in the DRG. These data suggest that distinct local agonists may activate CTR-based receptors, such as the AMY1 receptor, in C-fibre neurons in the DRG and TG. This highlights that local amylin may play a more important role in DRG-mediated pain responses than in the TG.

## P91 Glibenclamide posttreatment does not inhibit levcromakalim induced headache: A randomized clinical trial

### L. Kokoti, M. A. Al-Karagholi, C. A. Waldorff Nielsen, M. Ashina

#### Danish Headache Center, Neurology, Glostrup, Denmark

##### **Correspondence:** L. Kokoti

**Objective**: ATP-sensitive potassium (K_ATP_) channel opener levcromakalim causes headache in humans. Whether K_ATP_ channel blocker glibenclamide inhibits levcromakalim-induced headache has not yet been elucidated.

**Methods**: In a double blind, randomized, three-arm, placebo-controlled study, 20 healthy participants were assigned to receive 20 mL of levcromakalim (0.05 mg/min (50 mg/mL) or placebo (saline) intravenously over 20 minutes followed by oral administration of 10 mg glibenclamide or placebo. The primary endpoint was the difference in incidence of headache (0–12 hours) between glibenclamide and placebo.

**Results**: Fifteen participants completed all three study days. More participants developed headache on levcromakalim-placebo day (15/15, 100%) and levcromakalim-glibenclamide day (13/15, 86%) compared to placebo-placebo day (7/15, 46%) (P < 0.05). We found no difference in headache incidence between levcromakalim-placebo day and levcromakalim-glibenclamide day (P > 0.05). The AUC_0-12h_ for headache intensity was significantly larger in levcromakalim-placebo day and levcromakalim-glibenclamide day compared to placebo-placebo day (106.3± 215.8) (P < 0.01). There was no difference in the AUC_0-12h_ for headache intensity between the levcromakalim-placebo (494 ± 336.6) day and the levcromakalim-glibenclamide day (417 ± 371.6) (P > 0.05).

**Conclusion**: Non-specific K_ATP_ channel inhibitor glibenclamide did not attenuate levcromakalim-induced headache in healthy volunteers. Future studies should clarify the involvement of the distinct isoforms of sulfonylurea receptor subunits of K_ATP_ channels in the pathogenesis of headache and migraine.

## P92 Monthly occipital nerve blocks in chronic tension-type headache – a tertiary centre results

### B. Martins, A. Fernandes, M. Pinto, A. Costa

#### Centro Hospitalar Universitário de São João, Neurology, Porto, Portugal

##### **Correspondence:** B. Martins

**Question:** Peripheral nerve blocks are used in the acute and preventive treatment of primary headaches. Few studies have studied its effectiveness in tension-type headache (TTH), with contradictory results.

**Methods:** Observational study of patients followed in a headache-intervention consultation of a tertiary hospital (01/2021-04/2022), submitted to monthly greater and lesser occipital nerve blocks (GON, LON) with lidocaine 2% (2.5mL GON, 1.0mL LON).

**Results:** Peripheral nerve blocks were performed in 75 patients, median of 46 years (P25=36.0, P75=57.0), 63 (84.0%) were female. The most prevalent type of headache was migraine (38.7%), followed by cTTH (20.0%). Of the patients with cTTH (n=15), 13 (86.7%) were female and 10 (66.7%) were older than 50 years-old. Five (33.3%) had concomitant medication-overuse headache. Twelve patients (80.0%) were receiving concomitant prophylactic treatment. In 33.3% the block was performed due to refractory course, and in 33.3% due to treatment intolerance. Overall, in this group, there was a decrease in the number of headache days with treatment [p<.001; 23.9 (sd 8.6) pre-treatment vs. 10.0 (sd 11.1) post-one treatment]; only 4 (14.8%) had no response. An interaction with age (≤50/>50) (p=.001) was observed, with a higher reduction in headache frequency in patients >50y (Mprevious=22.7±9.1; Mpost-blockade=8.1±9.5), compared to ≤50y (Mprior=15.4±10.0; Mpost-block=9.2±9.6). There was an interaction with follow-up time (0-6/7-24/>24) (p=.029), with a greater reduction in frequency in the group with a follow-up time of up to 6months.

**Conclusions:** Patients with cTTH had a good response to monthly occipital nerve blocks. Treatment responsiveness seemed to be better in older patients and those that started this intervention earlier.

## P93 Recording pre-ictal migraine changes in cortical responsivity using EEG in a patient's home environment

### T. van den Hoek^1^, B. van der Arend^1^, E. Tolner^2^, M. van de Ruit^3^, A. van den Maagdenberg^2^, G. Terwindt^1^

#### ^1^Leiden University Medical Center, Neurology, Leiden, Netherlands; ^2^Leiden University Medical Center, Department of Human Genetics & Neurology, Leiden, Netherlands; ^3^Delft University of Technology, Department of Biomedical Engineering, Delft, Netherlands

##### **Correspondence:** T. van den Hoek

Brain excitability appears a critical biosignature of migraine leading up to attacks that can help to monitor a participant's susceptibility to develop an attack. We have shown – in our clinical EEG laboratory – that differences in cortical responsivity (as measure of brain excitability) in relation to the initiation of a migraine attack can be identified using longitudinal measurements of electroencephalographic (EEG)^1^ responses to flashing light, using the 'Chirp' paradigm^2^ (**fig. 1c**). For the current study we have moved out of the lab to the patient's home environment.

We will record EEG changes toward the (pre-)ictal phase of individual attacks using visual evoked potential EEG (VEP-EEG) (**fig. 1a**). Participants fill out our validated headache E-Diary, providing daily information about headache, medication use and menstrual bleeding.^3,4^ Recordings will start six days prior to an expected (menstrual) migraine attack. In the majority of cases 3-7 days of daily sessions (~30 min each) will be performed, but if needed recordings will be extended until an attack occurs. VEP-EEGs will be measured using a mobile EEG-system and flashing lights presented by LED goggles (**fig. 1b**).^1^ The flash light sequences consist of a short pulse-train over a broad frequency range of 10-40 Hz ('chirp' stimulation).^2^

We have successfully set up a home-based neuromonitoring system that will help us understand brain excitability changes in a patient's home environment by capturing daily fluctuations as possible early warning signs of upcoming migraine attacks.

We have set up a neuromonitoring system to perform home-based VEP-EEG measurements in a large group of migraine patients. For the future, picking up early changes in EEG signals in relation to attacks may form the basis for guiding early interventions and monitoring treatment effects.

1. Perenboom et al. 2020 Cephalalgia
2. Gantenbein et al. 2014 Cephalalgia
3. van Casteren et al. 2021 Cephalalgia
4. van Casteren et al. 2021 Neurology

**Fig. 1 (abstract P93).**
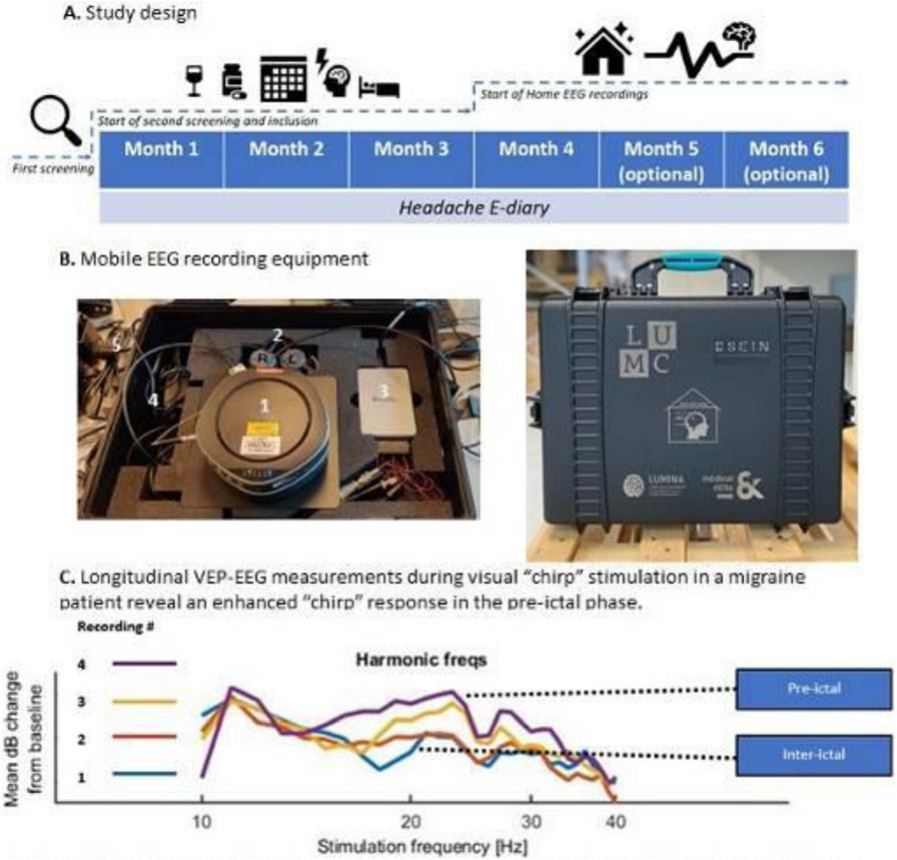
**A** Study design **1B.** Suitcase for mobile EEG equipment for recordings at patient’s home containing: 1) EEG device 2) LED goggles 3) Triggerbox 4) Headcaps in different sizes 5) Power supply. **1C.** Unpublished data of migraine patient that underwent our longitudinal study design to assess cortical EEG responses to visual ‘chirp’ stimulation (i.e. short 10-40 Hz pulse train of light flashes), during different phases of the migraine attack. VEP-EEG analyses reveal a visible difference in the harmonic frequency responses between the 1^st^ recording (interictal) and the 4^th^ recording (pre-ictal), after which a migraine attack occured within 24 hours

## P94 Vestibular signs in experimentally induced migraine attacks: a post-hoc, exploratory analysis

### M. Corrado^1^, F. Bighiani^1^, C. Demartini^1^, R. Greco^1^, A. Zanaboni^1^, V. Grillo^1^, G. Sances^1^, M. Allena^1^, C. Tassorelli^1^, R. De Icco^1^

#### ^1^IRCCS Mondino Foundation, Headache science and Neurorehabilitation Center, Pavia, Italy

##### **Correspondence:** M. Corrado

**Question:** Vestibular migraine (VM) as defined in ICHD-3 represents one of the most common vestibular syndromes, although its pathophysiology is not fully understood. The acute phase of VM is characterized by transitory oculo-vestibular signs (OVSs) that usually disappear outside of the VM attack. The difficulty to study spontaneous migraine attacks led to inconsistent results, and we believe that the adoption of human migraine models can help overcome this issue.

**Methods:** In this post-hoc analysis, we investigated the incidence of OVSs during experimentally induced migraine attacks in 24 episodic migraine patients without VM and 19 healthy controls exposed to sublingual nitroglycerin (NTG 0.9 mg). A comprehensive oculo-vestibular examination was performed at baseline, at migraine-like onset and before hospital discharge (180 minutes after NTG).

**Results:** Sixteen out of the 24 migraine patients developed a migraine-like attack (66.7%). Three of them (12.5%) developed OVSs during the migraine-like attack. In line with previous literature, we described a combination of central (down-beating nystagmus) and peripheral (bilateral deficit of vestibulo-ocular reflex) vestibular signs. Noteworthy, no patients with a negative induction test developed OVSs. No OVSs were detected in healthy subjects at any timepoints. Noteworthy, no subjects complained of vestibular symptoms throughout the study procedures.

**Conclusions:** Human migraine models may indeed be appropriate tools to evaluate the vestibular dysfunction in migraine and in VM under well-controlled experimental conditions. The present findings represent a starting point to design future ad-hoc and well-powered studies to deepen our knowledge on this topic.

## P95 NMDA, AMPA, Kainate Receptors in Medication Overuse Headache - Focus on Correlations of the Therapy with the HPA-Axis and Pineal Gland Activities

### M. Nicolodi^1^, M. S. Pinnaro^2^, L. Sicuteri Di Puccio^3^

#### ^1^Foundation Primary Headache and Stress, Research, Florence, Italy; ^2^University of Florence, Science of Nutrition, Florence, Italy; ^3^University of Padua, Neuropsychology, Padua, Italy

##### **Correspondence:** M. Nicolodi

**BACKGROUND** Observation examined effects of dextromethorphan, a manageable N-methyl-D-aspartate receptor antagonist involved with AMPA and kainate receptors trafficking, for medication overuse headache (MOH) and correlations of its effect with variables of patients. In 2006 we wrongly indicated the drug ineffectiveness. It was due to treatment shortness. The drug was shown needing a longer time for sensory remapping.

**METHOD** Participants 576 MOH. Presented data regard 378 (34.6±7.9SD 284 females) divided in 2 matched groups.

**Procedure**: dextromethorphan 1.5 mg/kg/PO vs amitriptyline 1 mg/kg day/PO, 2 months treatment-period. Baseline evaluation of: Adrenocorticotropic hormone (ACTH), cortisol, L-citrulline co-product in nitric oxide synthesis, microbiome, methylation, patterns, pineal gland volume (MRI), melatonin, psychometric tests.

**RESULTS**

*Dextromethorphan Therapeutic and Side Effects*

**Drop-out** n= 1

**Effect** decrease pain severity (87%), hrs./pain (72%) p>0.0001 vs amitriptyline (42%, 39% respectively)

**Less than 35% benefit**

Post-traumatic stress disorder n= 9 females,2 males

Opioids abusers n=25 females

**Absolute Refractoriness** n=2 females surgically treated pyneocitoma

**Side effects**

Drowsiness n=135 first week

*Dextromethorphan: Therapeutic Effect Correlations*

**Positive**

Cortisol abnormal pattern and high values p< 0.02

or low values p <0.01

ACTH abnormal pattern and value p>0.02

L-citrulline high p>0.009

Sleep rhythm alteration p<0.009

Depression Hamilton D p>0.01

Anxiety Hamilton A p<0.05

Microbiome disturbances p<0.001

Melatonin abnormalities p<0.02

Pineal dimensions RMN p<0.05

**No/Poor**

Methylation patterns NS

Social stress test TSST p<0.6

**CONCLUSION**

Hypothalamic-pituitary-adrenal axis function and pineal gland may play role in MOH mechanism and in dextromethorphan effectiveness

## P96 Elucidating the relationship between brain structure and migraine risk using genetic data

### B. L. Mitchell^1^, S. Díaz-Torres^1^, S. Bivol^1^, G. Cuéllar-Partida^2^, Z. F. Gerring^1^, N. G. Martin^1^, S. E. Medland^1^, K. L. Grasby^1^, D. Nyholt^3^, M. E. Renteria^1^

#### ^1^QIMR Berghofer Medical Research Institute, Mental Health and Neuroscience Program, Brisbane, Australia; ^2^The University of Queensland, The University of Queensland Diamantina Institute, Brisbane, Australia; ^3^Queensland University of Technology, School of Biomedical Sciences, Faculty of Health, Centre for Genomics and Personalised Health, Brisbane, Australia

##### **Correspondence:** M. E. Renteria

Migraine risk is associated with both genetic and brain morphometry differences. Yet, the relationship between migraine, brain morphometry and genetics has not been studied concurrently. Here we have used summary statistics from the largest available genome-wide association studies to examine the genetic overlap between migraine and brain volumes (i.e., intracranial volume and regional volumes of nine subcortical brain structures). We further focused on identifying and annotating genomic regions with a shared aetiology between brain imaging measures and migraine risk. Finally, we examined whether the size of any of the examined brain regions was causally associated with an increase in migraine risk using a Mendelian randomisation approach.

At the genome-wide level, we observed a significant negative genetic correlation between migraine risk and intracranial volume (rG = -0.11, p = 1x10-3) but not with any subcortical region. However, at the regional level, we identified jointly associated genomic loci shared between migraine and every brain structure. Gene enrichment in these shared genomic regions suggested possible links with neuronal signalling and vascular regulation. Finally, we provide evidence of a possible causal relationship between smaller total brain, hippocampal and ventral diencephalon volume, and increased migraine risk as well as a causal relationship between increased risk of migraine and a larger volume of the Amygdala.

In summary, we leveraged the power of large genome-wide association studies to show evidence of shared genetic pathways that jointly influence migraine risk and several brain structures, suggesting that altered brain morphometry in individuals with high migraine risk may be genetically mediated. Further interrogation of these results showed support for the neurovascular hypothesis of migraine etiology and shed light on potentially viable therapeutic targets.

## P97 Reversible cerebral vasoconstriction syndrome and polymorphisms of thrombophilia genes

### S. Zamanian

#### Social security organization, Mashhad, Iran

Background and aims:

(RCVS)characterized by the acute intense headache, focal and/or universal cerebral symptoms, epileptic paroxysms,accompanied by reversible segmental multifocal cerebral vasospasm, which disappears in three months.Aim of our study was to identify gene polymorphisms predisposing to hereditarv thrombophilia in REVS patients

Methods: 24 patients (age 38+11 years) with RCVS were examined: 19 women (79.1%) aged 38.0+11.4 years,5 men (20.8%) aged 38.2+11.3 years. There didn't find significant gender difference in age. Investigation included routine clinica and neurological examination,neurolayme research methods (brain MRI on 1.5T or 3T, MR arterio-graphy) and molecular genetic study of polymorphisms predisposing to thrombophilia: G20210A of prothrombin gene, C677T methylenetetrahydrofolate reductase gene, 675 4G/5G gene of endothelial plasminogen activator inhibitor (PA1-1, SERPINE1), 455 G/A gene of the beta-polypeptide chain of fioninogen

Results: Polymorphism G20210A in the prothrombin gene was not detected in the examined patients. Heterozygous carriage in the methylenetetrahydrofolate reductase gene was observed in 10 patients - in nine women and one man (43.5%), homozygous-in two women (8.3%). Polymorphism of the endothelial plasminogen activator inhibitor gene was detected in 16 patients (12 women and four men) in the heterozygous state (66.7%) and in three - in the homozygous

state (12.5%). Polymorphism 455 G/A was detected in heterozygous state in six patients (25%): five women and one man and in homozygous state in four patients 16.7%- two women and two men

Conclusion: The role of the revealed changes in the development of the complicated course of CVS requires further study.

**Fig. 1 (abstract P97).**
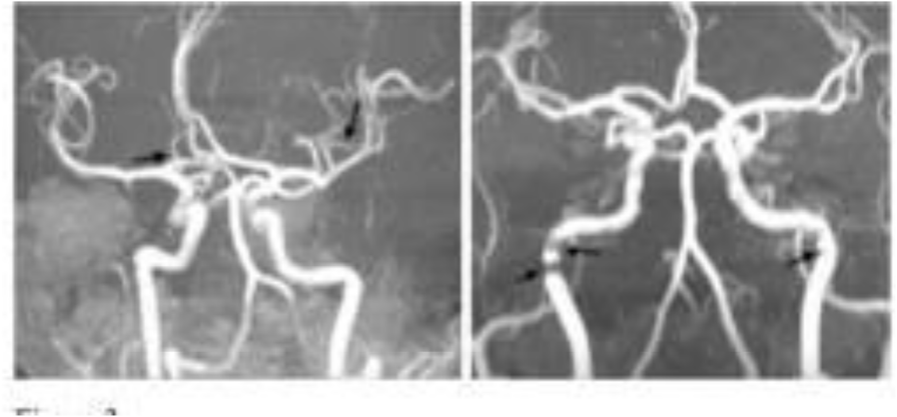
See text for description.

## P98 A system-wide retrospective cohort analysis toward an epidemiology of persistent post-concussive symptoms

### P. Brown^1,2^, D. Ali^2^, S. Olet^3^, J. Posas III^2^

#### ^1^The University of Queensland - Ochsner School of Medicine, Brisbane, Australia; ^2^Ochsner Xavier Institute for Health Equity and Research, Neurology, New Orleans, LA, United States; ^3^Ochsner Xavier Institute for Health Equity and Research, New Orleans, LA, United States

##### **Correspondence:** P. Brown

Question

The purpose of this study was to evaluate the prevalence of persistent post-concussion symptoms (PPCS; traditionally termed post-concussion syndrome, or PCS), associated risk factors (demographics, clinical presentation, premorbidity, and geographical location), and to determine the risk of developing psychiatric or nonpsychiatric disorders following mild traumatic brain injury (mTBI).

Methods

A state-wide, Ochsner Health (OH) hospital data collection was conducted between 2010 and 2020 to identify patients diagnosed with mTBI– based on "ICD-10 criteria" as stated by the electronic medical record. The results of this study indicate there is a significant association between patient characteristics and the development of PPCS.

Results

1481 (13.9%) patients developed PPCS following mTBI. Patient demographics including race and ethnicity demonstrated significant associations with PPCS (p<0.0001; p=0.0001). The presence of somatic, emotional, and cognitive symptoms following mTBI all demonstrated significant associations to the development of PPCS (p<0.0001). Somatic symptoms following mTBI was the single most influential factor identified towards the development of PPCS (OR 26.64, 95% CI [22.03-29.84], p<0.0001). Geographic location also demonstrated significant associations, with isolated communities at almost four times the likelihood of developing PPCS compared to urban communities (OR 3.72, 95% CI [1.97-7.01], p<0.0001). Lastly, our study showed an increased probability of psychiatric and non-psychiatric disorders when developed in congruence with PPCS (p<0.0001).

Conclusion

Prior to this research, no study to our knowledge has applied most recent consensus guidelines to prevalence studies of PPCS, nor to identifying demographic, neuropsychiatric risk and prognostic factors. Our findings provide the most up to date evidence of the association between patient characteristics and the development of persistent post-concussion symptoms.

**Fig. 1 (abstract P98).**
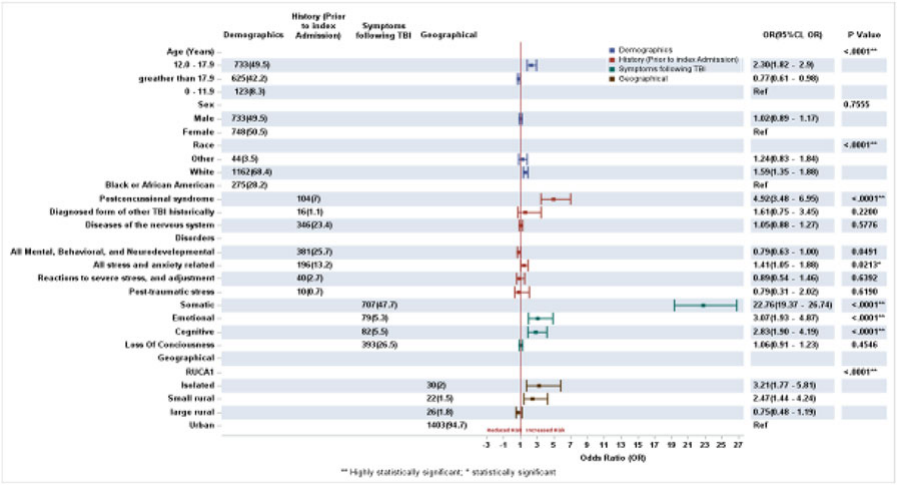
See text for description.

**Fig. 2 (abstract P98).**
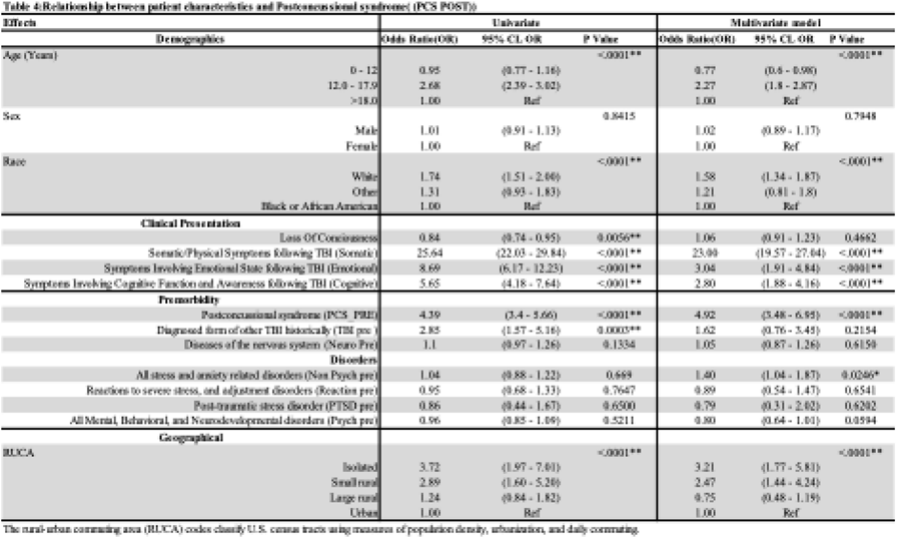
See text for description.

## P99 Painful trigeminal neuropathy. A series of 30 cases

### M. Borrell-Pichot, T. Mederer-Fernandez, R. Sainz-Torres, J. M. Fernandez-Vidal, C. Toscano-Prat, R. Collet-Vidiella, A. Martinez-Viguera, G. Olmedo-Saura, R. Belvís, N. Morollón

#### Hospital de la Santa Creu i Sant Pau, Neurology, Barcelona, Spain

##### **Correspondence:** M. Borrell-Pichot and T. Mederer-Fernandez

QUESTION

Painful Trigeminal Neuropathy (PTN) is an entity which affects the trigeminal nerve axonally and has not yet been well studied. We present our series of the last five years.

METHODS

Observational, descriptive and retrospective study from a database consisting of patients with trigeminal pain followed up in a tertiary hospital for the last five years. We include patients with PTN (ICHD3 criteria: code 13.1.2). We analyzed epidemiological, etiological, clinical and therapeutic variables.

RESULTS

We found 30 patients with PTN (4%) out of 262 patients with trigeminal pain (classic TN: 33%, secondary: 15%, idiopathic: 40%). Women: 67%, mean age of debut: 61.1y [21-92] with an evolution of 3.6y and mean follow-up of 18 months.

Symptoms: right side 48%, left side 31%, bilateral 21%. Affected branches: V1 31%, V2 17%, V3 7%, V1+V2 21%, V2+V3 14%, V1+V2+V3 10%. Burchiel type 2: 94%, Burchiel type 1+2: 7%.

Causes: post-herpetic 28%, post-surgery 24%, post-traumatic 10%, oral infection 3%, idiopathic 34%.

Need for therapy: 90%. Most frequently used drug at start: PGB 38%, GBP 35%, others 27%. Average number of therapies: 2. Monotherapy efficacy: 73%; Polytherapy: 27%. Most effective drug: GBP 30.7% (mean dose: 1212mg/d); CBZ/OXC/ESLI 23%; PGB 19%; AMT 19%; transdermal lidocaine 15%; Duloxetine 8%, LCS 8%, anaesthetic blockade of affected branch 8%.

Most frequent associations: post-herpetic + V1: 100%. Response to transdermal lidocaine in herpetic causes: 50%. Bilateral involvement + idiopathic cause: 100%.

CONCLUSIONS

PTN is an infrequent entity in trigeminal pain (4%) and it is clinically and etiologically heterogeneous. Bilateral affection is associated with an idiopathic cause, and herpetic causes always affect V1. The most effective drug was gabapentin and 50% of post-herpetic cases showed a good response to transdermal lidocaine.

## P100 Pressure Pain Threshold over Neural Structures in Patients with Acute Whiplash Associated Disorders with and without Headache: A Case-Control Study

### E. Anarte-Lazo^1^, C. Bernal-Utrera^1^, D. Falla^2^, C. Rodriguez-Blanco^1^

#### ^1^University of Seville, Department of Physiotherapy, Seville, Spain; ^2^University of Birmingham, School of Sport, Exercise and Rehabilitation Sciences, Birmingham, United Kingdom

##### **Correspondence:** E. Anarte-Lazo

**Objective:** To evaluate whether there are differences in pressure pain thresholds (PPT) over neural structures in the cranio-cervical region and upper limbs between patients who present with headache and those who do not shortly after a whiplash injury

**Methods:** A case-control study was conducted from September 2020 to February 2021 in a Traumatology Clinic in Madrid, Spain. PPT (N/cm2) were evaluated with an algometer twice over the median, ulnar, radial, supra-orbitaire and greater occipital nerves bilaterally. The evaluator was blinded to the group allocation.

**Results:** Among 49 consecutive patients who were assessed, 41 eligible patients were included in this study, 22 and 19 with and without headache, respectively.

Baseline differences between groups were found in relation to sex; there were more women in the group with headache (73.7% vs 50% in the non-headache group). No baseline differences were found in age, height, weight and days from the accident to the evaluation. When compared to the non-headache group, significantly lower PPT (mean ± SD; p-value) were found in the headache group for left (20,92±7,4; p=0,031) and right (20.90±8,5; p=0,028) radial nerves, right ulnar nerve (16.64±5,52; p=0,016) left (8.47±2,62; p=0,008) and right (8.79±3,15; p=0,008) supra-orbitaire nerves and left (8.79±3,10; p<0,001) and right (8.64±2,83; p<o,001) greater occipital nerves.

**Conclusion:**

People who present with headache soon after a whiplash injury show lower PPT over neural structures when compared to those patients who did not develop headache. These findings suggest the presence of greater sensitization in those who develop headache following a whiplash trauma.

## P101 Post-traumatic headache, a narrative review and a report of the latest therapeutic and management guideline (EHF abstract)

### M. Babaei, M. Togha

#### Tehran University of Medical Sciences, Headache Department, Tehran, Iran

##### **Correspondence:** M. Babaei

Post traumatic headache (PTH) is referred to any newly developed or worsened previously existing headache, occurring within 7 days after trauma or regaining consciousness post-trauma. Headaches resolving within 3 months after onset are called acute and persisting beyond 3 months are called persistent.

A prevalence of 33-92% has been reported for PTH. Considering the significant increase in the amount of traumatic injuries, PTH as the most prevalent sequel of trauma is also believed to rapidly increase. Interestingly, there is no dose-response relationship between the injury and headache severity. PTH mostly resembles migraine, tension type, and cervicogenic headaches as well as trigeminal autonomic cephalalgias.

PTH has a benign course with complete symptom resolution. It usually resolves within 3-6 months after its onset, though it might persist for one year or even longer. In a minority of patients headache have a prolong course and sometimes resistant to different treatment modalities.

Risk factors for PTH include age, sex, headache at emergency department at first admission, psychological disorders and medications, substance abuse, history of pre-injury headaches, history of physical or sexual abuse, low educational achievements, medication overuse and associated factors including dizziness, fatigue, decreased concentration, psychomotor slowing, memory problems, insomnia, anxiety, personality changes are addressed for PTH.

Pathophysiology of PTH is mainly related to inflammatory markers increasing in CNS after trauma and increased permeability of blood brain barrier to immune agents and pathogens.

Work up includes comprehensive patient evaluation and tests to rule out serious conditions. Medication usually begins with analgesics and NSAIDs with management of comorbidities and psychological support. If the pain didn"t get resolved, further pharmacologic medication will be prescribed according to the therapeutic management of the nearest headache phenotype.

## P102 Differences on the Flexion-Rotation Test in Patients ith Acute Whiplash Associated Disorders with and without Headache: A Case-Control Study

### E. Anarte-Lazo^1^, C. Bernal-Utrera^1^, D. Falla^2^, C. Rodriguez-Blanco^1^

#### ^1^University of Seville, Department of Physiotherapy, Seville, Spain; ^2^University of Birmingham, School of Sport, Exercise and Rehabilitation Sciences, Birmingham, United Kingdom

##### **Correspondence:** E. Anarte-Lazo

**Objective:** To assess if subjects who develop headache shortly after a whiplash injury show less range of motion on the flexion-rotation test (FRT) than those who do not develop headache

**Methods:** A case-control study was conducted on patients between 18-65 years old diagnosed with whiplash associated disorders (WAD) grade II according to the Quebec Task Force. Patients were excluded if they had previous headache prior to the whiplash injury, were evaluated more than 30 days after the whiplash injury and/or who had a serious disease or congenital condition. Range of motion (°) on the FRT was evaluated bilaterally in consecutively recruited patients. The evaluator was blinded to the headache status.

**Results:** 41 patients were included in this study, 22 and 19 with and without headache, respectively. Baseline differences between groups were found only in relation to sex; there were more women in the group with headache (73.7% vs 50% in the non-headache group). Statistical analysis revealed that range of motion (°) on the FRT was significantly reduced in patients with headache on both the left (mean±SD: 28,98±7,76 vs 35,29±4,92; p=0,002) and right side (29,73±7,7 vs 36,84±4,05; p<0,001) when compared to the non-headache group.

**Conclusion:**

A decreased range of motion was observed on the FRT in patients who have developed headache shortly after a whiplash injury when compared to those patients who did not develop headache. These findings suggest that the upper cervical structures may be involved in the presence of headache in patients with acute WAD.

## P103 Atogepant for the preventive treatment of chronic migraine: results from the PROGRESS phase 3 trial

### P. Pozo-Rosich^1^, J. Ailani^2^, M. Ashina^3^, P. Goadsby^4^, R. Lipton^5^, U. Reuter^6^, H. Guo^7^, B. Schwefel^7^, R. Boinpally^7^, E. McCusker^7^, S. Yun Yu^7^, M. Finnegan^7^, J. Trugman^7^

#### ^1^Vall d’Hebron University Hospital and Autonomous University of Barcelona, Barcelona, Spain; ^2^Medstar Georgetown University Hospital, Georgetown Headache Center, Washington, DC, United States; ^3^University of Copenhagen, Rigshospitalet, Danish Headache Center, Copenhagen, Denmark; ^4^NIHR-Wellcome Trust King's Clinical Research Facility, King's College London; University of California, London; Los Angeles, United Kingdom; ^5^Albert Einstein College of Medicine, Bronx, NY, United States; ^6^Department of Neurology, Charité - Universitätsmedizin Berlin; Universitätsmedizin Greifswald, Berlin, Germany; ^7^AbbVie, Madison, NJ, United States

##### **Correspondence:** P. Pozo-Rosich

**Objective:** To evaluate the efficacy, safety, and tolerability of atogepant, an oral CGRP receptor antagonist, for the preventive treatment of chronic migraine (CM).

**Methods**: PROGRESS (NCT03855137) was a 12-week phase 3 trial in adults with CM, randomized 1:1:1 to atogepant (30mg twice daily [BID], 60mg once daily [QD]) or placebo. Primary efficacy endpoint was change from baseline in mean monthly migraine days (MMDs) over 12 weeks. A key secondary endpoint was proportion of participants with ≥50% reduction in 3-month average of MMDs.

**Results**: Of 778 participants (89.2% completed double-blind treatment period), 773 were in safety population (average age=42.1 years; average BMI=25.5 kg/m2; 87.6% female; 59.4% White; 36.4% Asian), and 755 in modified intent-to-treat (mITT) population. Baseline mean MMDs (mITT population) were 18.6 – 19.2 across groups. Mean change from baseline over 12 weeks was −7.5 days for atogepant 30mg BID, −6.9 for atogepant 60mg QD, and -5.1 for placebo (atogepant 30mg BID vs. placebo, *p*<0.0001, atogepant 60mg QD vs. placebo, *p*=0.0009). Reduction of ≥50% in 3-month average of MMDs was achieved by 42.7% of participants in the atogepant 30mg BID group, 41.0% in the atogepant 60mg QD group and 26.0% in the placebo group (30mg BID vs. placebo, *p*=0.0003, 60mg QD vs. placebo, *p*=0.0009). Treatment-emergent adverse events (TEAEs) were reported by 56.4% (atogepant 30mg BID), 63.2% (atogepant 60mg QD) and 49.4% (placebo) of participants. Most frequent TEAEs (≥5% any group): constipation (10.9% atogepant 30mg BID, 10.0% atogepant 60mg QD, 3.1% placebo); nausea (7.8% atogepant 30mg BID, 9.6% atogepant 60mg QD, 3.5% placebo). Serious TEAEs were reported by 1.6% (atogepant 30mg BID), 2.7% (atogepant 60mg QD) and 1.2% (placebo) of participants; none treatment-related.

**Conclusion**: Atogepant showed significant reductions in MMDs in participants with CM and was safe and generally well-tolerated.

## P104 A Phase 2/3 Open-label, Long-term, Safety Trial of Zavegepant 10 mg Nasal Spray for the Acute Treatment of Migraine

### R. Croop^1^, J. Madonia^1^, J. Hould^1^, L. Mosher^1^, M. Lovegren^1^, V. Coric^1^, R. Lipton^2^

#### ^1^Biohaven Pharmaceuticals, New Haven, CT, United States; ^2^Albert Einstein College of Medicine, Bronx, NY, United States

##### **Correspondence:** R. Croop

Objective

Evaluate the safety of zavegepant 10 mg nasal spray, the only small molecule CGRP receptor antagonist (gepant) for intranasal administration in late-stage development for the acute treatment of migraine.

Methods

This was a Phase 2/3, 1-year open-label safety study (NCT04408794) of zavegepant nasal spray for the acute treatment of migraine. Adults aged ≥18 years with a history of 2 to 8 moderate-severe monthly migraine attacks were eligible. Use of another gepant was prohibited. Subjects self-administered 1 dose of zavegepant 10 mg nasal spray per calendar day as needed to treat migraine attacks of any severity, up to 8 times per month, for 52 weeks. Months were defined as 4-week intervals. Safety assessments included adverse events (AEs), vital signs, ECG, nasal inspection, and clinical laboratory tests. Subjects who took ≥1 dose of zavegepant were included in the analysis.

Results

Of 608 subjects who entered the long-term treatment phase, 603 were treated with zavegepant 10 mg nasal spray. At baseline, the mean (SD) number of attacks per month was 5.0 (1.89), and 18.1% of treated subjects used preventive migraine medication. Treatment-emergent AEs reported in ≥ 5% of subjects (Figure) were dysgeusia (39.1%); nasal discomfort (10.3%); COVID-19 (7.5%); nausea (6.1%); nasal congestion and throat irritation (5.5% each); and back pain (5.3%). In total, 6.8% of subjects discontinued due to AEs; 1.5% discontinued due to dysgeusia. The majority of AEs (96.4%) were mild to moderate. Of the 7 serious AEs reported, none was considered related to treatment by the investigators. Aminotransferases >3x the upper limit of normal (ULN) occurred in 2.6% of subjects, none of whom had concurrent elevations in bilirubin >2x ULN. Subjects used a mean (SD) of 3.1 (1.6) zavegepant doses per month.

Conclusion

Favorable safety and tolerability profiles were observed with 1 year of open-label zavegepant 10 mg nasal spray for the acute treatment of migraine.

**Fig. 1 (abstract P104).**
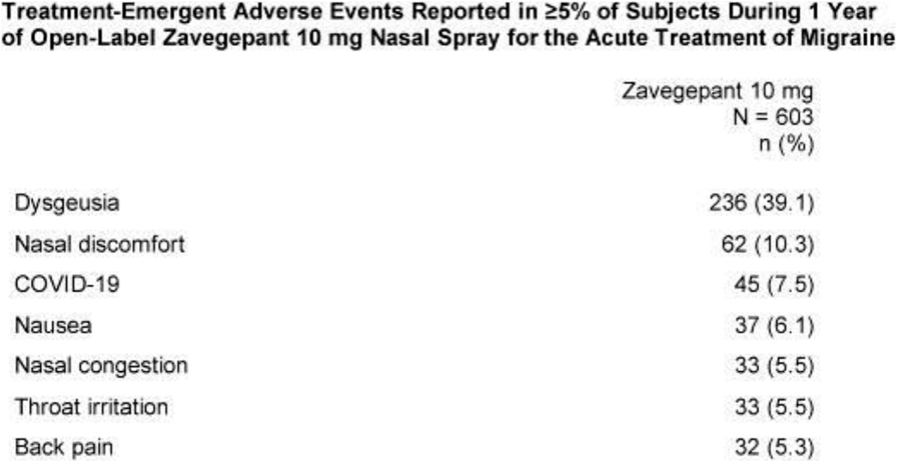
See text for description.

## P105 Sustained Response to Atogepant in Individuals with Episodic Migraine: Post Hoc Analyses of 12- and 52-Week Phase 3 Trials

### R. Lipton^1^, S. Nahas^2^, P. Pozo-Rosich^3^, T. Bilchik^4^, P. McAllister^5^, M. Finnegan^6^, J. Ma^6^, T. Chalermpalanupap^6^, B. Dabruzzo^6^, D. Dodick^7^

#### ^1^Albert Einstein College of Medicine, Bronx, NY, United States; ^2^Thomas Jefferson University, Philadelphia, PA, United States; ^3^Vall d’Hebron University Hospital and Autonomous University of Barcelona, Barcelona, Spain; ^4^Yale School of Medicine, Nerw Haven, CT, United States; ^5^New England Institute for Neurology & Headache, Stamford, CT, United States; ^6^AbbVie, Madison, NJ, United States; ^7^Mayo Clinic, Scottsdale, AZ, United States

##### **Correspondence:** R. Lipton

**Objective**:To evaluate proportion of participants who sustained initial responses of ≥50%, ≥75%, or 100% reduction in mean monthly migraine days (MMDs) over 12 and 52-weeks of atogepant treatment for episodic migraine.

**Methods**:Post-hoc analyses of 2 phase 3 trials: ADVANCE (NCT03777059), 12-week placebo-controlled trial of atogepant (10, 30, 60mg); and a separate 52-week open-label, long-term safety (LTS) trial (NCT03700320) of 60mg atogepant. Participants had initial response if they achieved ≥50% reduction from baseline in MMDs in month 1 (4 weeks) for ADVANCE or quarter 1 (3-month average) for the LTS trial. Participants achieving initial response were categorized by response threshold (≥50%, ≥75%, 100%). Proportion of participants sustaining the same initial response or ≥50% through each subsequent month, or quarter, was calculated.

**Results**:In ADVANCE (Table 1), 70.8–81.1% of participants who achieved a response of ≥50% in MMDs in month 1 sustained their response (ie, a ≥50% response in month 2 and 3) throughout the treatment period. Between 47.3–61.9% of those who were ≥75% responders in month 1 sustained their response, while 79.2–86.9% maintained ≥50% response in month 2 and 3. Of participants achieving initial 100% treatment response, 34.8–41.7% remained migraine-free over 3 months (86.4–95.0% sustained responses of ≥50%, and 66.7–69.6% sustained responses of ≥75%). During the LTS trial (Table 2), 84.7%, 72.6%, and 42.2% of participants who achieved an initial response of ≥50%, ≥75%, or 100% in quarter 1, sustained these responses through quarter 2, 3, and 4. Of those who were initial ≥75% and 100% responders, >90% maintained ≥50% response in each subsequent quarter*.* Few participants with initial response were non-responders (<25% reduction in MMD) at the end of ADVANCE or LTS trials.

**Conclusion**:Findings demonstrate the majority of participants who achieved initial atogepant treatment response sustained it with continued treatment over a 52-week period.

**Table 1 (abstract P105).**
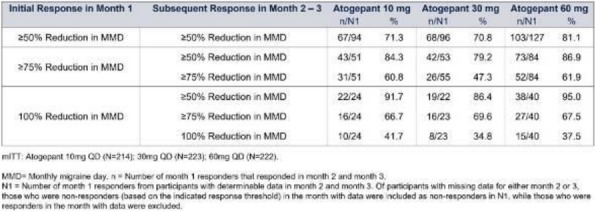
Proportion of Participants in ADVANCE with an Initial Response ≥50%, ≥75% or 100% Reduction in MMD with Sustained Response through to Month 3

**Table 2 (abstract P105).**
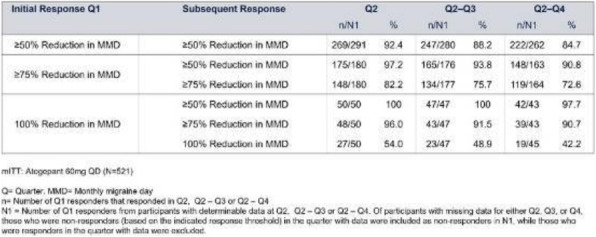
Proportion of Participants in the long-term safety trial with an Initial Response of ≥50%, ≥75% or 100% Reduction in MMD with Sustained Response through to Q4

## P106 Post-hoc Analysis Evaluating Safety of Atogepant in ADVANCE & Open-Label Extension Participants with Cardiovascular Risk Factors

### A. Blumenfeld^1^, J. Pavlovic^2^, A. Harriott^3^, P. Best^4^, T. Montheith^5^, R. De Abreu Ferreira^6^, J. Ma^6^, J. Smith^6^, B. Dabruzzo^6^, S. Nahas^7^

#### ^1^Headache Center of Southern California, Carlsbad, CA, United States; ^2^Albert Einstein College of Medicine, Bronx, NY, United States; ^3^Massachusetts General Hospital, Boston, MA, United States; ^4^Mayo Clinic, Rochester, MN, United States; ^5^University of Miami, Miami, FL, United States; ^6^AbbVie, Madison, NJ, United States; ^7^Thomas Jefferson University, Philadelphia, PA, United States

##### **Correspondence:** A. Blumenfeld

**Objective:** ADVANCE (NCT03777059) was a phase 3, 12-week trial in episodic migraine participants. ADVANCE trial completers were eligible to roll over into a 40-week, open-label, extension trial (309-OLEX, NCT03939312). The objective was to evaluate safety of atogepant in participants with cardiovascular risk factors (CV-RFs) from ADVANCE and 309-OLEX.

**Methods:** Post-hoc analysis of participants receiving placebo or 60mg atogepant from ADVANCE and 309-OLEX trials were evaluated for CV-RFs: age [men ≥45 years; women ≥55 years], BMI ≥25 kg/m2, cardiovascular disease (CVD) [including history of myocardial infarction, stroke, or transient ischemic attack], diabetes, dyslipidemia, hypertension, sleep apnea, smoking; or taking concomitant medications for CVD, diabetes, or hypertension. (Goff et al. Circulation 2014;129:S49–S73) Participants were stratified into 3 groups: 0, 1, or ≥2 CV-RFs.

**Results:** Percentage of participants with 0, 1 or ≥2 CV-RFs were similar across treatment arms and trials (Table 1); 87% of ADVANCE and 88% of 309-OLEX participants treated with atogepant had at least 1 CV-RF. Most common CV-RFs in both trials included BMI ≥25 kg/m2 (73.9-74.1%), hypertension (35.6-38.6%) and dyslipidemia (36.9-37.9%). CV treatment-emergent adverse events (CV-TEAEs) were infrequent (<5%, Table 1). Treatment-related CV-TEAEs included atrioventricular block in 1 placebo participant (0.4%), and palpitations in 2 atogepant participants (one in each trial [0.1%-0.5%]). No CV serious adverse events (CV-SAEs) were observed in either trial (Table 1). Treatment emergent hypertension events were reported in 0.4% of atogepant participants and 0.5% of placebo participants in the ADVANCE trial, and in 1.6% of the 309-OLEX trial participants.

**Conclusions:** Participants with baseline CV-RFs were well represented in ADVANCE and 309-OLEX. Low incidence of CV-TEAEs were noted. These data provide evidence supporting the safety profile of atogepant, specifically in those with CV-RFs.

**Table 1 (abstract 106).**
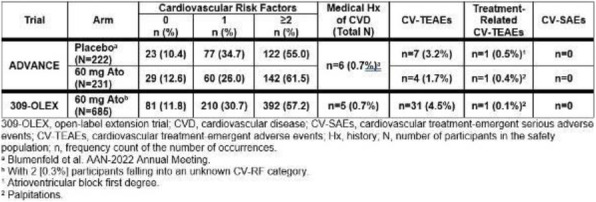
See text for description.

## P107 Cut-off value for Tampa Scale for Kinesiophobia in migraine patients

### G. A. Tolentino^1^, C. F. Pinheiro-Araujo^2^, L. L. Florencio^3^, J. Martins^1^, A. C. C. Norato^1^, F. D. Sambini^1^, F. Dach^1^, D. Bevilaqua Grossi^1^

#### ^1^University of São Paulo, Ribeirão Preto, Brazil; ^2^Federal University of Alfenas, Alfenas, Brazil; ^3^Universidad Rey Juan Carlos, Alcorcón, Spain

##### **Correspondence:** G. A. Tolentino

**Objective:** Identify the optimal cut-off value for kinesiophobia in migraine patients using the Tampa Scale for Kinesiophobia (TSK).

**Methods:** Fifty women aged between 18 and 55 years (mean 33.9; SD 9.69) with migraine were evaluated. Migraine diagnosis followed the third edition of the International Headache Society criteria. All participants completed the questionnaires TSK and Migraine Disability Scale (MIDAS). The disability is a variable associated with kinesiophobia. The MIDAS was used as a binary variable and the TSK as a continuous variable. Thus, receiver operating characteristic analyses were conducted to identify a clinically relevant cut-off score capable of distinguishing kinesiophobia in migraine patients. The diagnostic accuracy was interpreted as follows: 0.9- 1, excellent; 0.8- 0.9, very good; 0.7- 0.8, good; 0.6- 0.7, sufficient; and 0.5- 0.6, bad, and <0.5 not useful. The local ethics committee approved the study (6862/2016).

**Results:** The cut-off value for kinesiophobia in migraine individuals is > 34 points. This tool presented sensibility of 74.3% (95% CI 57.87% to 86.96%) and specificity of 63,6% (95% CI 30.79% to 89.07%), with good accuracy of 72% (95% CI 57.51% to 83.77%) to differentiate kinesiophobia in individuals with migraine. Furthermore, there was a low diagnostic value positive likelihood ratio of 2.04 (95% CI 0.92 to 4.57) and a negative likelihood ratio of 0.40 (95% CI 0.20 to 0.81), positive predictive values ranged from 76.46% to 94.18% and negative predictive values from 25.86% to 58.42%.

**Conclusion:** The optimal TSK cut-off of 34 points in migraine patients has been established with good accuracy. This cut-off is beneficial for clinicians to assess the presence of kinesiophobia in patients with migraine. The cut-off score can help identify patients who need additional attention and treatment, such as pain neuroscience education and cognitive-behavioral therapy.

## P108 A retrospective real-life multicenter study on concurrent oral preventives in patients with chronic migraine treated with botulinum toxin

### L. H. Overeem^1,2^, R. Ornello^3^, M. M. Pocora^4,5^, A. N. Dueland^6,7^, S. Sacco^3^, C. Tassorelli^4,5^, U. Reuter^1,8^, B. Raffaelli^1,9^, D. Martinelli^4,5^

#### ^1^Charité University Hospital Berlin, Neurology, Berlin, Germany; ^2^Humboldt Graduate School, International Graduate Program Medical Neurosciences, Berlin, Germany; ^3^University of L'Aquila, Neuroscience Section, Department of Biotechnological and Applied Clinical Sciences, L'Aquila, Italy; ^4^IRCCS Mondino Foundation, Headache science and rehabilitation center, Pavia, Italy; ^5^University of Pavia, Department of Brain and Behavioral Sciences, Pavia, Italy; ^6^Sandvika Nevrosenter, Sandvika, Norway; ^7^Oslo University Hospital, Neurology, Oslo, Norway; ^8^Universitätsmedizin Greifswald, Greifswald, Germany; ^9^Berlin Institute of Health at Charité (BIH), Clinician Scientist Program, Berlin, Germany

##### **Correspondence:** L. H. Overeem

**Objective**

Onabotulinumtoxin A (BoNTA) is a safe and effective treatment for chronic migraine (CM). The local action of BoNTA favors the combination with oral treatments with systemic action. However, little is known about the possible interactions with other preventives. We aimed to describe pharmacological patterns in patients with CM treated with BoNTA in routine clinical care and discuss safety and efficacy according to the presence or absence of concomitant oral treatments.

**Methods**

In this multi-center, observational, retrospective, cohort study, we collected data from patients with CM receiving prophylactic treatment with BoNTA. We documented concomitant migraine prophylactic treatments (CcMP) and their side effects during four BoNTA treatment cycles. Additionally, we collected monthly headache days (MHDs) and monthly acute medication days (AMDs) from the patients' headache diaries. Patients with a CcMP treatment were compared with those without using a nonparametric approach.

**Results**

We analyzed data from 181 patients, of whom 77 (43%) received a CcMP treatment. The most prescribed concomitant treatments were antidepressants and antihypertensive drugs. Side effects caused by the CcMP treatments occurred in 18% (n=14) of the patients. Only in 4% (n=3), these adverse events (all caused by topiramate) had a significant interference with the patients" functioning. Both patients with and without CcMP treatment had a significant reduction of MHDs and AMDs (p<0.001). The reduction of MHDs, however, was significantly lower in patients with CcMP treatment (p=0.018) during the fourth treatment cycle compared to patients without any concomitant treatment.

**Conclusion**

In our cohort, we did not identify any unexpected safety issues in patients treated with BoNTA and a CcMP treatment. Patients with a CcMP treatment might have a smaller reduction in MHDs than those without CcMP treatments, possibly due to the high resistance to treatments in that subgroup of patients.

## P109 Impact of Eptinezumab on Patient-Reported Outcomes in Patients With Prior Preventive Treatment Failures

### P. Goadsby^1^, P. Barbanti^2^, G. Lambru^***3***^, A. Ettrup^4^, C. L. Christoffersen^4^, M. K. Josiassen^4^, R. Phul^4^, B. Sperling^4^

#### ^1^King's College London, NIHR-Welcome Trust King's Clinical Research Facility, London, United Kingdom; ^2^IRCCS San Raffaele, Headache and Pain Unit, Rome, Italy; ^3^Guy's and St Thomas' NHS Foundation Trust, Headache Centre, London, United Kingdom; ^4^Lundbeck, Copenhagen, Denmark

##### **Correspondence:** G. Lambru and A. Ettrup

**OBJECTIVE:** In DELIVER, eptinezumab reduced migraine frequency in patients with migraine and prior preventive treatment failures. This analysis evaluates patient-reported outcomes (PROs) over 24 weeks.

**METHODS:** DELIVER (NCT04418765) randomized and treated patients with intravenous eptinezumab 100mg, 300mg, or placebo every 12 weeks. The assessed PROs include EuroQol 5-Dimensions 5-Levels visual analogue scale (EQ-5D-5L VAS; higher scores better); 6-item Headache Impact Test (HIT-6; lower scores better), Patient Global Impression of Change (PGIC; lower scores better), most bothersome symptom (MBS; lower scores better), and Migraine-Specific Quality of Life Questionnaire (MSQ, v2.1; higher scores better).

**RESULTS:** Patients received eptinezumab 100mg (n=299), 300mg (n=294), or placebo (n=298). Mean changes from baseline to Week 12 (Wk12) in EQ-5D-5L VAS scores were 2.0 (100mg, *P*=0.0007) and 4.4 (300mg, *P*<0.0001) versus -3.1 (placebo), and were maintained or improved to Wk24 (2.0, 5.2, -2.8, respectively). Mean baseline HIT-6 total scores were ~66.4, with mean changes at Wk12 of -6.9 (100mg, *P*<0.0001) and -8.5 (300mg, *P*<0.0001) versus -3.1 (placebo) that were improved through Wk24 (-8.9, -9.9, -3.9). PGIC, MBS, and MSQ scores showed greater improvement for eptinezumab than placebo.

**CONCLUSION:** In adults with migraine and prior preventive treatment failures, eptinezumab robustly improved health-related quality of life and reduced migraine-related burden over 24 weeks versus placebo.

## P110 Subsequent Response to Atogepant in Individuals with Episodic Migraine after an Initial Inadequate Response: Post Hoc Analysis of a 12-week Phase 3 Trial

### D. Dodick^1^, S. Nahas^2^, P. Pozo-Rosich^3^, T. Bilchik^4^, P. McAllister^5^, M. Finnegan^6^, J. Ma^6^, T. Chalermpalanupap^6^, B. Dabruzzo^6^, R. Lipton^7^

#### ^1^Mayo Clinic, Scottsdale, AZ, United States; ^2^Thomas Jefferson University, Philadelphia, PA, United States; ^3^Vall d’Hebron University Hospital and Autonomous University of Barcelona, Barcelona, Spain; ^4^Yale School of Medicine, New Haven, CT, United States; ^5^New England Institute for Neurology & Headache, Stamford, CT, United States; ^6^AbbVie, Madison, NJ, United States; ^7^Albert Einstein College of Medicine, Bronx, NY, United States

##### **Correspondence:** D. Dodick

**Objective**: Post hoc analysis of ADVANCE (NCT03777059) to determine the proportion of participants who did not achieve initial response of ≥25% or ≥50% reduction in mean monthly migraine days (MMDs) that subsequently responded with continued atogepant treatment.

**Methods**: ADVANCE was a 12-week, phase 3 trial, evaluating the safety and efficacy of atogepant for preventive treatment of migraine. This analysis calculated the proportion of atogepant-treated participants who achieved <25% or <50% reduction from baseline in mean MMDs in month 1 that subsequently achieved at least that response in month 2 and in either month 2 or month 3, and the proportion of atogepant-treated participants who achieved <25% or <50% reduction in MMDs in month 1 and month 2 that achieved at least that response in month 3.

**Results**: Few atogepant-treated participants (6.1- 8.4%) achieved <25% reduction in MMDs monthly (atogepant 10mg 17/203; 30mg 17/220; 60mg 13/212). Of those with <25% response in month 1 (Table 1), 36.2 – 48.3% achieved ≥25% reduction in MMDs in month 2 and 66.7 – 71.7% in month 2 or 3 Of participants with <50% reduction from baseline MMD in month 1 (Table1), 33.8 – 41.3% achieved ≥50% reduction from baseline MMDs in month 2 and 52.8 – 61.4% in month 2 or 3. Some participants who did not achieve ≥25% or ≥50% reductions in MMDs in months 1 or 2 (Table2) were able to achieve at least these responses. 31.6 – 48.5% achieved ≥25% reduction in MMDs in month 3 and 16.7 – 37.2% achieved ≥50% reduction in MMDs in month 3.

**Conclusion**: While the majority of atogepant-treated participants in the ADVANCE trial responded to treatment within the first month, of those who did not, a substantial number achieved at least a 25% or 50% reduction in monthly migraine days in the second or third month.

**Table 1 (abstract P110).**
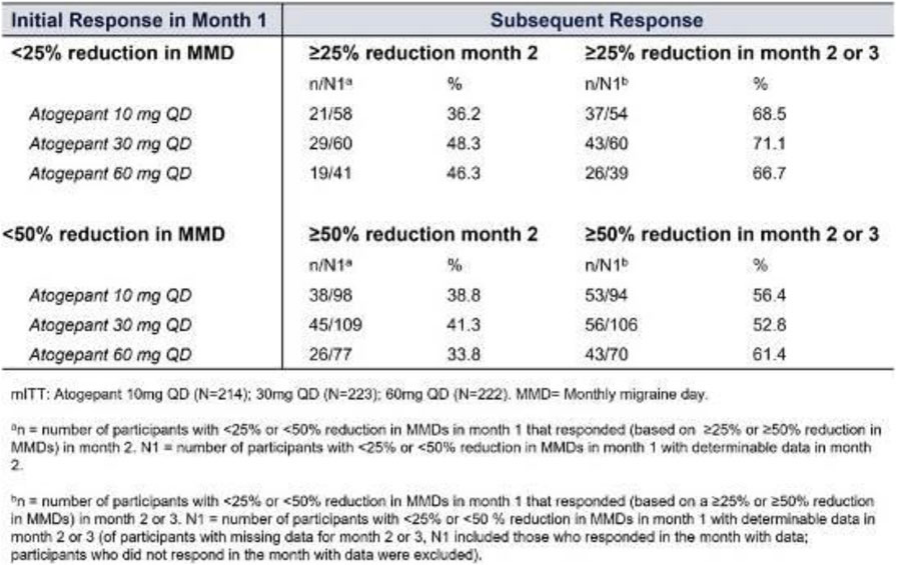
Proportion of participants with an initial inadequate response in month 1 that achieved a subsequent response of ≥25% or ≥50% reduction in MMDs in month 2 or 3

**Table 2 (abstract P110).**
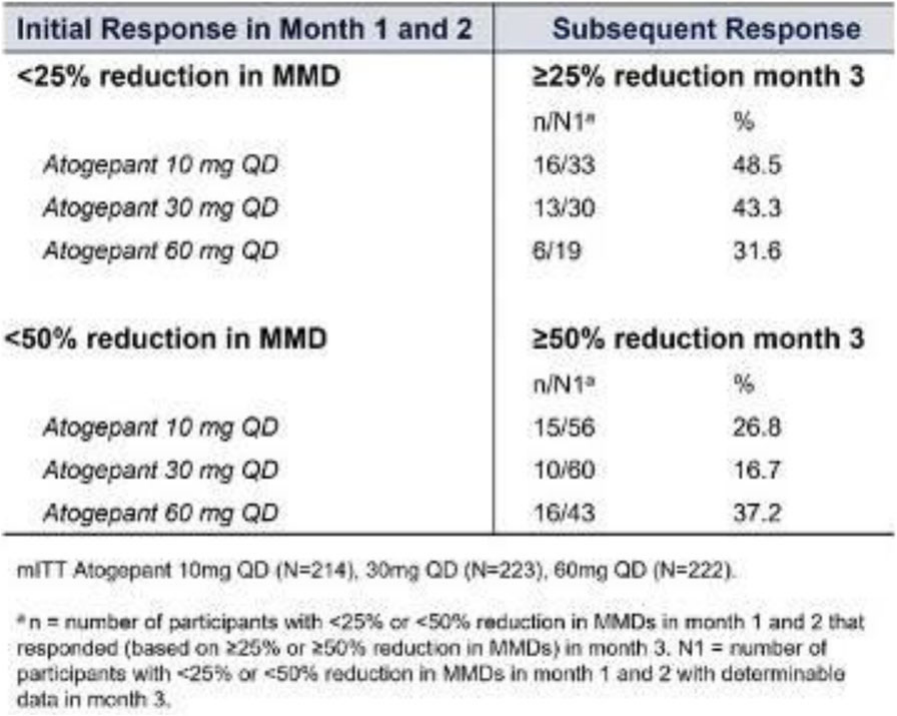
Proportion of participants with an initial inadequate response in month 1 and 2 that achieved a subsequent response of ≥25% or ≥50% reduction in MMDs in month 3

## P111 Eptinezumab for Migraine Prevention in Patients with 2-4 Prior Treatment Failures: DELIVER Subpopulation Analysis

### M. Ashina^1^, M. Lanteri-Minet^2^, P. Pozo-Rosich^3^, A. Ettrup^4^, C. L. Christoffersen^4^, M. K. Josiassen^4^, R. Phul^4^, B. Sperling^4^

#### ^1^University of Copenhagen, Rigshospitalet, Danish Headache Center, Copenhagen, Denmark; ^2^Pain Department and FHU InovPain, Centre Hospitalier Universitaire de Nice and Université Côte Azur, Nice, France; ^3^Vall d’Hebron University Hospital and Autonomous University of Barcelona, Barcelona, Spain; ^4^Lundbeck, Copenhagen, Denmark

##### **Correspondence:** P. Pozo-Rosich

**OBJECTIVE:** In DELIVER, eptinezumab reduced migraine frequency in patients with episodic (EM) or chronic migraine (CM) and 2-4 documented prior preventive treatment failures versus placebo. This analysis evaluated the preventive migraine efficacy of eptinezumab in specific subgroups of patients.

**METHODS:** DELIVER (NCT04418765) randomized patients to eptinezumab 100mg, 300mg, or placebo (administered intravenously every 12 weeks). Change from baseline in monthly migraine days (MMDs) over Weeks 1-12 was analyzed in patient subgroups, including sex, disease classification, medication-overuse headache (MOH) diagnosis, and number of previous treatment failures.

**RESULTS:** Eptinezumab-treated patients demonstrated greater MMD reductions over Weeks 1-12 than placebo across all subgroups, with larger numerical advantages in patients with MOH versus the general population, CM versus EM, high-frequency versus low-frequency EM, and >2 prior preventive treatment failures versus 2 prior failures. The 95% confidence intervals for mean differences from placebo in change from baseline did not cross 0 for any subgroups except men and patients with low-frequency EM (both had ≤40 patients per treatment arm).

**CONCLUSIONS:** Across all explored subgroups of adults with migraine and prior preventive treatment failures, greater reductions in MMDs over Weeks 1-12 were observed with eptinezumab versus placebo in patients with MOH, CM, and >2 documented prior treatment failures.

## P112 Efficacy and Safety of Eptinezumab for Migraine Prevention in Patients With 2–4 Prior Preventive Treatment Failures

### M. Ashina^1^, M. Lanteri-Minet^2^, P. Pozo-Rosich^3^, A. Ettrup^4^, C. L. Christoffersen^4^, M. K. Josiassen^4^, R. Phul^4^, B. Sperling^4^

#### ^1^University of Copenhagen, Rigshospitalet, Danish Headache Center, Copenhagen, Denmark; ^2^Pain Department and FHU InovPain, Centre Hospitalier Universitaire de Nice and Université Côte Azur, Nice, France; ^3^Vall d’Hebron University Hospital and Autonomous University of Barcelona, Barcelona, Spain; ^4^Lundbeck, Copenhagen, Denmark

##### **Correspondence:** P. Pozo-Rosich

**Objective:** DELIVER evaluated the efficacy and safety of eptinezumab for migraine prevention in patients with migraine and prior preventive treatment failures.

**Methods:** DELIVER (NCT04418765) is a phase 3b, randomized clinical trial evaluating eptinezumab (100mg and 300mg administered intravenously every 12 weeks) for migraine prevention. The study includes a 24-week double-blind, placebo-controlled period and a 48-week dose-blinded extension conducted in adults (18-75y) with episodic or chronic migraine and 2-4 preventive treatment failures within the past 10y. The primary endpoint in the placebo-controlled period was change from baseline in monthly migraine days (MMDs) over Weeks (Wks) 1-12.

**Results:** A total of 891 individuals received ≥1 dose of study drug, and 865 completed the placebo-controlled period. Eptinezumab achieved statistically significant reductions in MMDs versus placebo over Wks1-12 (100mg, -4.8; 300mg, -5.3; placebo, -2.1; *P*<0.0001), which was maintained over Wks13-24. Over Wks1-12, more eptinezumab-treated patients achieved ≥50% and ≥75% MMD reduction vs placebo (*P*<0.0001). Additionally, efficacy endpoints on patient-reported outcomes were achieved. Incidence of treatment-emergent adverse events was 42.5% (100mg), 40.8% (300mg), and 39.9% (placebo).

**Conclusions:** In adults with migraine and prior preventive treatment failures, eptinezumab robustly decreased MMDs across Wks1-12 and Wks13-24 compared to placebo, with a favorable safety and tolerability profile.

## P113 Psychopathological disorders in chronic migraine: is there an association with the endocannabinoid system?

### S. Bottiroli^1,2^, R. Greco^1^, A. Zanaboni^1^, M. Allena^1^, E. Guaschino^1^, N. Ghiotto^1^, R. De Icco^1,3^, G. Sances^1^, C. Tassorelli^1,3^

#### ^1^IRCCS Mondino Foundation, Pavia, Italy; ^2^Giustino Fortunato University, Benevento, Italy; ^3^University of Pavia, Pavia, Italy

##### **Correspondence:** S. Bottiroli

**Objectives**: The understanding of factors involved in the prognosis of chronic migraine (CM) has become a topic of interest in the current debate. Compelling evidence has suggested a negative prognostic value for psychopathological disorders. Dysfunctions of the endocannabinoid system can underlie several psychiatric disorders. To date, no data is available for CM. Hence, the present study aims to evaluate the association existing between psychopathological disorders and endocannabinoid system in CM.

**Method:** Thirty-four patients (mean age=44.9±11.9) with CM (operationally defined according to ICHD-III) who failed at least three preventive therapies were enrolled and received full psychological evaluation according to DSM-V criteria for mood, anxiety, and personality disorders. Gene expression of enzymes involved in the synthesis and degradation of endocannabinoids and their receptors (CB1 and CB2) was assessed in peripheral blood mononuclear cells.

**Results**: Among enrolled patients, 53% (n=18) presented mood disorders (MD), 79% (n=27) anxiety disorders (AD). In addition, 53% (n=18) resulted positive for personality (PD) disorders (Cluster C - predominantly obsessive-compulsive disorder). Interestingly, different associations between these psychopathological disturbances and mRNA levels of cannabinoid receptors were found. Specifically, higher CB1 (2.97 ± 2.05 vs 1.66 ± 1.00, p=.018) and NAPE (2.09 ± 0.62 vs 1.63 ± 0.50, p=.04) receptor gene expression was found in MD when compared to non-MD. A tendency to higher FAAH values was found in AD when compared with non-AD (2.09 ± 0.62 vs 1.63 ± 0.50, p=.04). Finally, lower CB1 gene expression (1.48 ± 0.78 vs 2.52 ± 1.76, p=.027) was detected in PD when compared to non-PD.

**Discussion:** These preliminary findings provide knowledge regarding the association existing between psychopathological disorders and alterations in the endocannabinoid activity in CM.

## P114 Real-World Persistence and Costs Among Patients With Chronic Migraine Treated With OnabotulinumtoxinA or CGRP mAbs: A Retrospective Claims Analysis Study

### T. Schwedt^1^, J. Lee^2^, K. Knievel^3^, J. McVige^4^, W. Wang^5^, Z. Wu^5^, P. Gillard^2^, D. Shah^6^, A. Blumenfeld^7^

#### ^1^Mayo Clinic, Scottsdale, AZ, United States; ^2^AbbVie, Irvine, CA, United States; ^3^Barrow Neurological Institute, Phoenix, AZ, United States; ^4^DENT Neurologic Institute, Amherst, NY, United States; ^5^Genesis Research LLC, Hoboken, NJ, United States; ^6^AbbVie, Madison, NJ, United States; ^7^Headache Center of Southern California, Neurology, Carlsbad, CA, United States

##### **Correspondence:** T. Schwedt

**Objective:** Evaluate real-world persistence rates and costs among patients with chronic migraine (CM) treated with onabotulinumtoxinA (onabotA) or calcitonin gene–related peptide monoclonal antibody (CGRP mAb).

**Methods:** This retrospective, longitudinal, observational study analyzed the IBM MarketScan® Commercial and Medicare Supplemental databases (7/1/17-2/29/20). Adults treated with onabotA or CGRP mAbs (based on overall migraine ICD-10 codes) and having continuous coverage ≥6 months prior and ≥12 months after treatment initiation were included. Persistence to treatment was assessed at 6, 9, and 12 months, and all-cause and migraine-related costs were evaluated during the 12-month follow-up period. Persistence and costs were adjusted for potential confounders (demographics, comorbidities, oral migraine preventive medication [OMPM] use) using generalized linear model regression.

**Results:** Of 66,303 patients with onabotA or CGRP mAb claims, 2697 CM patients met inclusion/exclusion criteria. In the total population, patients were primarily female (86%) with a mean age of 44 y, which was consistent among the individual CGRP mAbs. Persistence was higher among those receiving onabotA vs the combined CGRP mAbs group at 6 (67% vs 47%; *P*<.001), 9 (51% vs 37%; *P*<.001), and 12 (40% vs 27%; *P*<.001) months. OnabotA and CGRP mAbs were associated with comparable 12-month all-cause ($16,681 vs $16,666) and migraine-related ($8198 vs $8518) costs. Compared to CGRP mAbs, onabotA was associated with lower 12-month acute medication ($763 vs $1240; *P*<.001), OMPM ($685 vs $993; *P*<.01), and migraine-related inpatient ($224 vs $728; *P*<.01) costs. Migraine-related emergency department costs were comparable between both groups ($149 vs $129). Findings were sustained after regression adjustment for confounders.

**Conclusions:** Patients with CM initiating onabotA had higher persistence and comparable all-cause and migraine-related costs over 12 months compared to those taking CGRP mAbs.

## P115 Rimegepant for the Acute Treatment of Migraine: Subgroup Analyses From 3 Phase 3 Clinical Trials by Triptan Treatment Experience

### C. M. Jensen^1^, R. Lipton^2^, A. Blumenfeld^3^, R. Croop^1^, A. C. Thiry^1^, G. L’Italien^1^, B. Morris^1^, V. Coric^1^, P. Goadsby^4,5,6^

#### ^1^Biohaven Pharmaceuticals, New Haven, CT, United States; ^2^Albert Einstein College of Medicine, Bronx, NY, United States; ^3^Headache Center of Southern California, Carlsbad, CA, United States; ^4^King's College London, NIHR-Wellcome Trust King’s Clinical Research Facility, London, United Kingdom; ^5^King’s College Hospital/SLaM Biomedical Research Centre, London, United Kingdom; ^6^University of California, Los Angeles, CA, United States

##### **Correspondence:** C. M. Jensen

Objective

Assess the efficacy of rimegepant — an oral small molecule calcitonin gene-related peptide receptor antagonist — for the acute treatment of migraine in subjects with and without a history of insufficient response to triptans.

Methods

Three double-blind, placebo-controlled trials of similar design randomized adults with migraine to rimegepant 75 mg tablet (NCT03235479, NCT03237845) or ODT (NCT03461757) or placebo to treat 1 migraine attack of moderate to severe pain intensity. Subgroups with a history of insufficient response with 1 or ≥2 triptans and those without a history of insufficient response, including triptan-naïve and current triptan users, were analyzed. Triptan insufficient response was defined as self-reporting a history of discontinuing ≥1 triptan due to inadequate efficacy and/or poor tolerability. The co-primary endpoints were 2-hour freedom from pain and the most bothersome symptom (MBS).

Results

In the pooled population (N=3507: rimegepant n=1749, placebo n=1758), 2272 (64.8%) subjects had no history of triptan insufficient response and 1235 (35.2%) had a history of insufficient response with ≥1 triptan. Results for the co-primary endpoints in each triptan subgroup are shown in Figure 1. No differences in co-primary endpoints were found in pairwise comparisons of triptan subgroups in rimegepant-treated subjects (Figure 2).

Conclusions

Rimegepant was effective for the acute treatment of migraine in subjects with and without a history of triptan insufficient response. The efficacy of rimegepant was consistent among those with insufficient response to 1 or ≥2 triptans and those who were triptan-naïve or currently using triptans.

**Fig. 1 (abstract P115).**
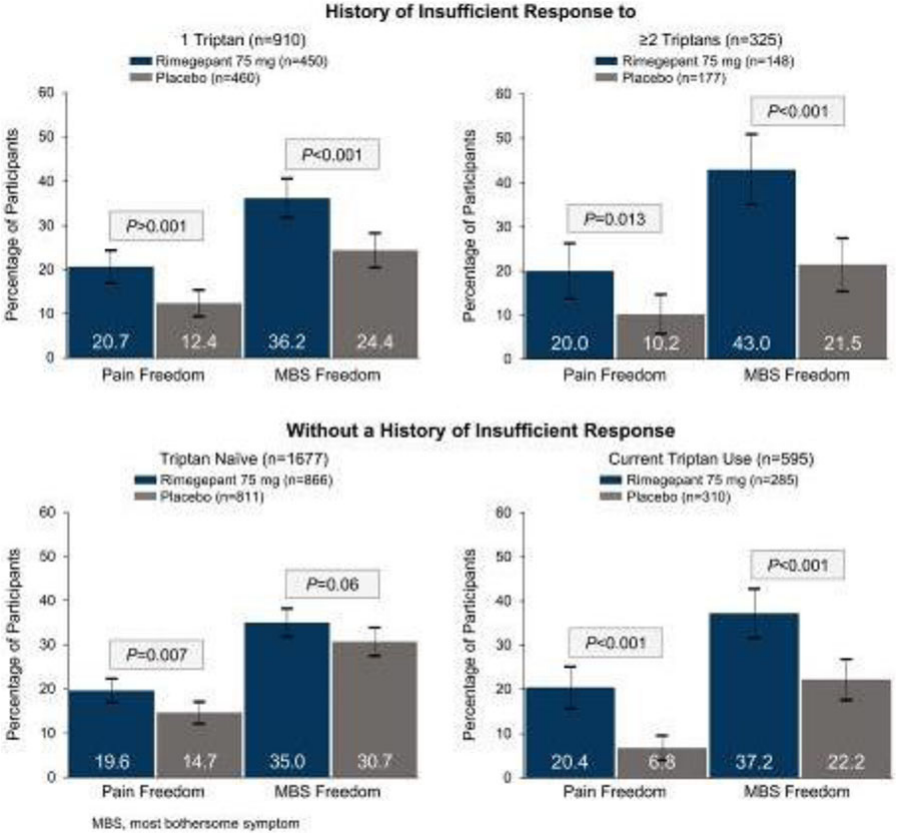
Co-primary Efficacy Endpoints at 2 Hours Postdose by Triptan Experience Subgroups

**Fig. 2 (abstract P115).**
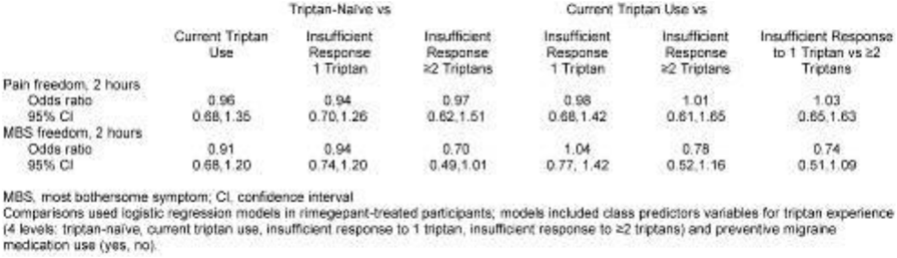
Coprimary Efficacy Endpoints Compared Pairwise between Triptan Experience Subgroups Using Logistic Regression Models in Rimegepant-Treated Subjects

## P116 Adenosine causes short-lasting vasodilation and headache, but not migraine attacks in migraine patients: A randomized clinical trial

### J. Thuraiaiyah, M. A. Al-Karagholi, F. Azzahra Elbahi, Z. A. Zhuang, M. Ashina

#### Danish Headache Center, Glostrup, Denmark

##### **Correspondence:** J. Thuraiaiyah

**Question**

Does adenosine infusion induce migraine attack?

**Methods**

In a randomized, double-blinded, placebo-controlled, crossover study, 18 participants diagnosed with migraine without aura were allocated to receive 120 μg/kg/min adenosine or placebo over 20 minutes. Headache intensity, migraine associated symptoms, vital signs, the diameter of the superficial temporal artery (STA) and blood flow velocity in the middle cerebral artery (V_MCA_) were measured at baseline and every 10 minutes until two hours post-infusion start.

The primary endpoint was the difference in incidence of migraine attacks after adenosine compared to placebo.

**Results**

Eighteen participants completed the study. We found no difference in incidence of migraine following adenosine (7/18, 39%) compared to placebo (3/18, 17%) (*P* = 0.29). Fourteen of 18 (78%) participants reported headache after adenosine compared to placebo (6/18, 33%) (*P* < 0.01). Adenosine increased heart rate (*P* < 0.001), facial skin blood flow (*P* < 0.05) and STA diameter (AUC_T0-20min_, *P* = 0.01), and decreased VMCA (AUC_T0-20min_, *P* < 0.001) compared to placebo.

While mean arterial blood pressure (*P* = 0.96) remained unaltered.

**Conclusion**

Adenosine induced headache accompanied by a short-lasting (< 30 min) dilation of intra- and extracerebral arteries. However, adenosine is a less powerful migraine inducer compared to other migraine inducing substances.

## P117 Treatment Responder Rates of Oral Atogepant for the Preventive Treatment of Chronic Migraine: Results From the PROGRESS Trial

### R. Lipton^1^, M. Ashina^2^, C. Tassorelli^3^, V. Martin^4^, S. Y. Yu^5^, K. Nagy^6^, B. Schwefel^7^, J. Trugman^5^

#### ^1^Albert Einstein College of Medicine, Bronx, NY, United States; ^2^University of Copenhagen, Rigshospitalet, Neurology, Copenhagen, Denmark; ^3^Headache Science Centre, C. Mondino Foundation and University of Pavia, Pavia, Italy; ^4^University of Cincinnati, Cincinnati, OH, United States; ^5^AbbVie, Madison, NJ, United States; ^6^AbbVie, Budapest, Hungary; ^7^AbbVie, North Chicago, IL, United States

##### **Correspondence:** R. Lipton

**Objective:** To evaluate mean monthly migraine day (MMD) responder rates to characterize the efficacy profile of atogepant in the preventive treatment of chronic migraine (CM).

**Methods:** PROGRESS was a multicenter, randomized, double-blind, placebo-controlled phase 3 study that assessed the safety, tolerability, and efficacy of atogepant 30 mg twice daily (BID) and 60 mg once daily (QD) compared with placebo for the preventive treatment of CM. Adults (18-80 years) with a ≥1-year history of CM and confirmation of ≥15 monthly headache days and ≥8 MMDs during the baseline period were randomized 1:1:1 to receive placebo, atogepant 30 mg BID, or atogepant 60 mg QD. These analyses evaluated ≥30%, ≥50%, ≥75%, and 100% reductions in mean MMDs across 12 weeks and at 4-week intervals. All reported *P* values are nominal; there was no adjustment for multiplicity.

**Results:** A total of 778 participants were randomized to treatment. The modified intent-to-treat population included 755 participants: placebo: n=246; atogepant 30 mg BID: n=253; and atogepant 60 mg QD: n=256. Atogepant-treated participants (30 mg BID and 60 mg QD) were significantly more likely than placebo-treated participants, respectively, to experience a ≥30% (62.1% and 59.0% vs 43.1%; *P*<0.001), ≥50% (42.7% and 41.0% vs 26.0%; *P*<0.001), or ≥75% (21.3% and 18.8% vs 5.7%; *P*<0.001) reduction in mean MMDs across 12 weeks. During weeks 1-4 and 5-8, the proportion of participants experiencing a ≥30% or ≥50% reduction in mean MMDs was significantly greater for both atogepant doses vs placebo and during weeks 9-12 for atogepant 30 mg BID vs placebo (**Figure**). The proportion of participants experiencing a ≥75% or 100% response was higher for both doses of atogepant in each 4-week interval assessed.

**Conclusions:** Both atogepant dosing regimens increased the proportions of participants with CM achieving ≥30%, ≥50%, ≥75%, or 100% reduction in mean MMDs across 12 weeks.

**Fig. 1 (abstract P117).**
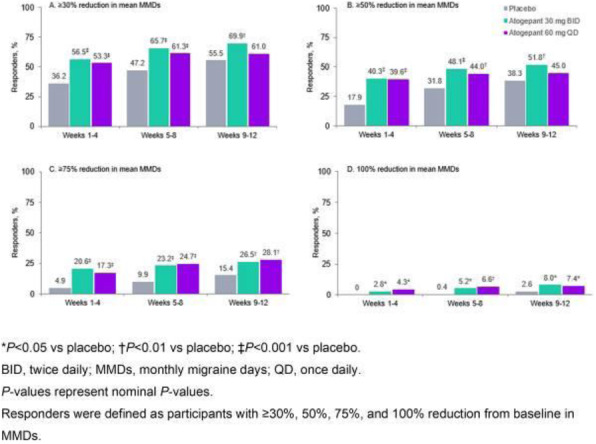
Reduction of (A) ≥30%, (B) ≥50%, (C) ≥75%, and (D) 100% in Mean MMDS BY 4-Week Intervals

## P118 Chronic Migraine Epidemiology and Outcomes – International (CaMEO-I) Study: methods and global findings for diagnosis rates and care

### D. C. Buse^1^, E. Leroux^2^, M. Lanteri-Minet^3^, F. Sakai^4^, M. Matharu^5^, Z. Katsarava^6^, M. Reed^7^, K. Fanning^8^, A. Manack Adams^9^, K. Sommer^9^, R. Lipton^1^

#### ^1^Albert Einstein College of Medicine, Bronx, NY, United States; ^2^Canadian Headache Society, Brunswick Medical Center, Montreal, Canada; ^3^Centre Hospitalo-Universitaire de Nice, Nice, France; ^4^Saitama International Headache Center, Chuo-ku, Saitama City, Japan; ^5^Institute of Neurology, London, United Kingdom; ^6^Evangelical Hospital Unna, Unna, Germany; ^7^Vedanta Research, Chapel Hill, NC, United States; ^8^MIST Research, Wilmington, NC, United States; ^9^AbbVie, Irvine, CA, United States

##### **Correspondence:** D. C. Buse

**Objective:** To describe the methodology and present findings on migraine diagnosis, consulting, and current medication use for migraine across 6 countries.

**Methods:** CaMEO-I was a cross-sectional, observational, web-based study in 2021 in 6 countries: US, Canada, UK, Germany, France, and Japan. A validated questionnaire identified patients with migraine based on modified *International Classification of Headache Disorders*, 3rd ed (mICHD-3) criteria. Qualified respondents provided sociodemographic background, headache features, migraine disability based on the Migraine Disability Assessment Scale (MIDAS), and history of consulting, diagnosis, and treatment patterns.

**Results:** A total of 14,492 individuals met criteria for migraine (approximately 2400 from each country) and were included in this analysis. The mean age among migraine respondents ranged from 40.3-42.3 years and the majority were female (68.7-73.8%). Median monthly headache days (MHDs) ranged from 2.3 to 3.3 days, with between 5.4% (France) to 9.5% (Japan) of respondents reporting ≥15 MHDs. Moderate-to-severe migraine-related disability was reported between 30.3% (Japan) to 52.0% (Germany) of migraine respondents (**Figure**). Self-reported medical diagnosis (SRMD) rates for migraine, chronic/transformed migraine, or menstrual migraine among those meeting the ICHD-3 case definition ranged from 42.8% (Japan) to 49.3% (US). The SRMD rates for chronic/transformed migraine ranged from 0.7% (Japan) to 4.8% (US) of respondents with migraine. In the overall migraine population, rates of current preventive use ranged from 6.4% (Japan) to 16.8% (US).

**Conclusions:** Between one-third and one-half of respondents who met mICHD-3 criteria for migraine reported moderate to severe migraine-related disability as measured by MIDAS. While there were between-country differences in the proportion of CaMEO-I respondents with an SRMD of migraine and chronic migraine, underdiagnosis of migraine was a concern in each country studied.

**Fig. 1 (abstract P118).**
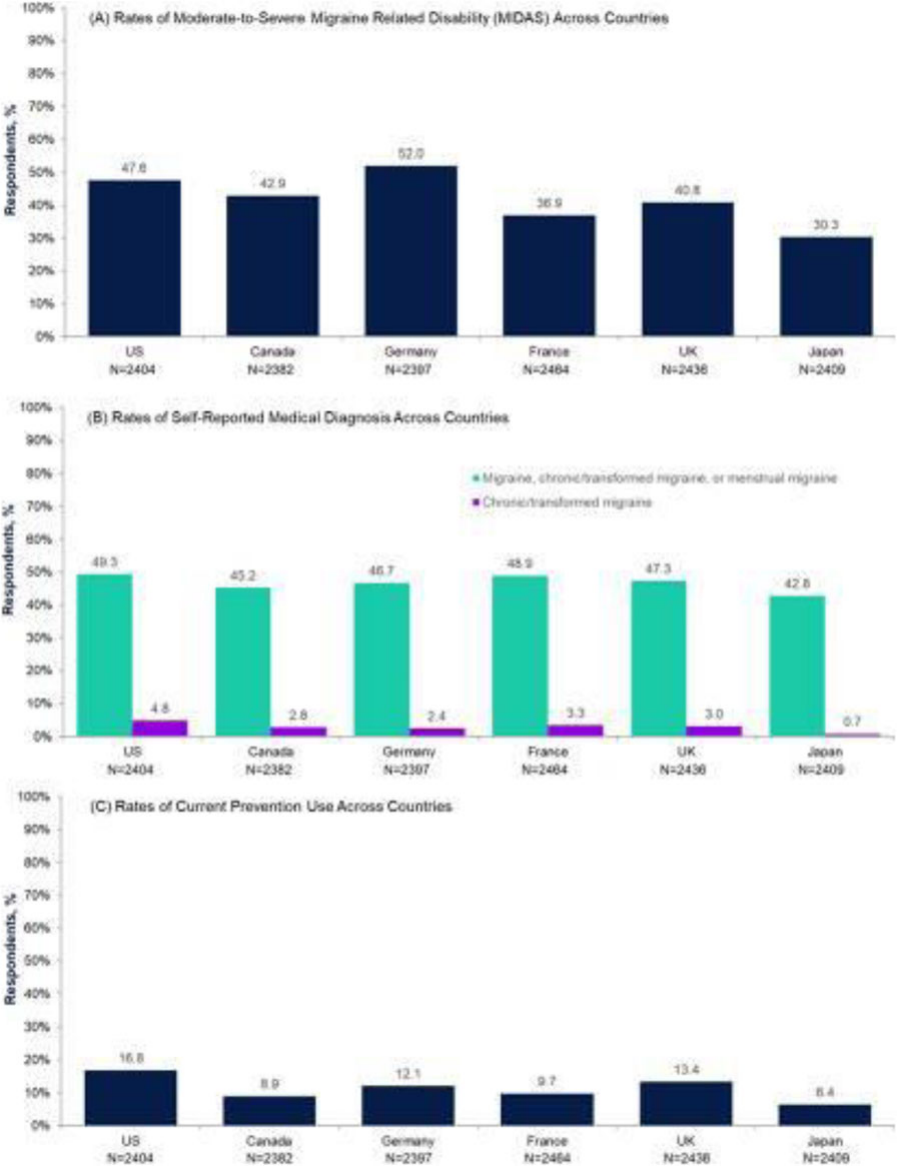
Rates of Moderate-to-Severe Migraine-Related Disability (A), Self-Reported Medical Diagnosis (B), and Current Prevention Use (C) Across Countries in CaMEO-I Respondents With Migraine

## P119 Eptinezumab Improved Work Productivity in Adults With Migraine and Prior Preventive Treatment Failures: Results From the Randomized, Double-Blind, Placebo-Controlled DELIVER Study

### P. Goadsby^1^, P. Barbanti^2^, G. Lambru^3^, A. Ettrup^4^, C. L. Christoffersen^4^, M. K. Josiassen^4^, R. Phul^4^, B. Sperling^4^

#### ^1^King's College London, NIHR-Welcome Trust King’s Clinical Research Facility, London, United Kingdom; ^2^Headache and Pain Unit, IRCCS San Raffaele, Rome, Italy; ^3^Guy's and St Thomas' Hospitals NHS Trust, The Headache Group, London, United Kingdom; ^4^Lundbeck, Copenhagen, Denmark

##### **Correspondence:** G. Lambru and A. Ettrup

**OBJECTIVE:** This analysis reports the impact of eptinezumab, an anti-calcitonin gene-related peptide monoclonal antibody, on work productivity and daily activities in patients with migraine and prior preventive treatment failures.

**METHODS:** The DELIVER study (NCT04418765) randomized adults (18-75y) with migraine and documented evidence of 2-4 prior preventive treatment failures to receive eptinezumab 100mg, 300mg, or placebo (IV every 12 weeks). At baseline and every 4 weeks, patients completed the migraine-specific 6-question Work Productivity Activity Impairment (WPAI:M) questionnaire (7-day recall). Changes from baseline in WPAI subscores were predefined secondary endpoints and analyzed without control for multiplicity.

**RESULTS:** The full analysis set included 890 patients (100mg, *n*=299; 300mg, *n*=293; placebo, *n*=298). Mean baseline WPAI subscores indicated a negative impact of migraine on work productivity and normal daily activities. Beginning at first post-baseline assessment at Week 4 and through Week 24, eptinezumab demonstrated larger reductions than placebo in absenteeism (*P*<0.05), presenteeism (*P*<0.001), work productivity loss (*P*<0.001), and activity impairment (*P*<0.001) subscores.

**CONCLUSIONS:** In adults with migraine and prior preventive treatment failures, eptinezumab treatment robustly improved migraine-related absenteeism, presenteeism, work productivity loss, and activity impairment as early as Week 4 and throughout the study.

## P120 Monthly Migraine Days, Acute Medication Use Days, and Migraine-Specific Quality of Life in Responders to Atogepant: A Post Hoc Analysis

### D. Dodick^1^, R. Lipton^2^, S. Nahas^3^, P. Pozo-Rosich^4^, P. McAllister^5^, L. Mechtler^6^, J. Ma^7^, B. Dabruzzo^7^, M. Dufek^7^, L. Severt^7^, M. Finnegan^7^, J. Trugman^7^

#### ^1^Mayo Clinic, Scottsdale, AZ, United States; ^2^Albert Einstein College of Medicine, Bronx, NY, United States; ^3^Thomas Jefferson University, Philadelphia, PA, United States; ^4^Vall d’Hebron University Hospital and Autonomous University of Barcelona, Barcelona, Spain; ^5^New England Institute for Neurology & Headache, Stamford, CT, United States; ^6^DENT Neurologic Institute, Buffalo, NY, United States; ^7^AbbVie, Madison, NJ, United States

##### **Correspondence:** D. Dodick

**Objective:** To characterize the magnitude of treatment effect in atogepant responders and nonresponders. In the phase 3 ADVANCE trial, treatment with atogepant 60mg reduced mean monthly migraine days (MMDs) from 7.8 days at baseline to 3.0 at weeks 9-12 (∆=-4.7) in the overall episodic migraine population, which included treatment responders and nonresponders (ie, participants with marked benefit and those with minimal benefit). This approach obscures clinically relevant information regarding the magnitude of treatment effect in these two populations.

**Design/Methods:** This post hoc analysis used data from participants who completed the 12-week ADVANCE trial. Mean MMDs, acute medication use days, and Migraine-Specific Quality of Life-Role Function-Restrictive (MSQ-RFR) scores were calculated in treatment responders (based on a percentage reduction in MMDs) and nonresponders.

**Results:** During weeks 9-12, a ≥50% improvement (ie, a 50%-100% reduction in MMDs from baseline) was achieved by 71% (139/195) of participants. In these responders, MMDs were reduced from 7.6 at baseline to 1.3 at weeks 9-12 (∆=-6.3). A ≥75% response was achieved in 50% (97/195) of participants. In this group, MMDs were reduced from 7.7 at baseline to 0.6 at weeks 9-12 (∆=-7.1). Atogepant 60mg nonresponders (<25% reduction in MMDs) comprised 15% (30/195) of participants and showed an MMD change from 7.7 at baseline to 9.1 at weeks 9-12 (∆=+1.4). Acute medication use days in ≥50% MMD responders decreased from 7.1 at baseline to 1.6 at weeks 9-12 (∆=-5.5). In treatment nonresponders (<25% reduction in MMDs), acute medication use days were 7.3 at baseline and 7.2 at weeks 9-12 (∆=-0.1). Similar results were observed for mean MSQ-RFR score changes in responders and nonresponders.

**Conclusions:** For the 71% of participants who experienced a ≥50% reduction in MMDs, a substantial treatment effect (∆MMD =-6.3) was observed, which represents an 83% reduction in MMDs.

## P120a Evaluation of PREEMPT fixed-dose, fixed-site and follow the pain treatment paradigms in the PREDICT Study

### C. Graboski^1^, M. Ong-Lam^2^, W. Becker^3^, J. Ma^4^, K. Sommer^5^, I. Finkelstein^6^

#### ^1^Island Health, Brentwood Bay, Canada; ^2^St Paul Hospital, Vancouver, Canada; ^3^University of Calgary, Calgary, Canada; ^4^AbbVie, Madison, NJ, United States; ^5^AbbVie, Irvine, CA, United States; ^6^Toronto Headache & Pain Clinic, Toronto, Canada

##### **Correspondence:** C. Graboski

**Objective**: To analyze the real-world effectiveness and safety of 155U, 156-195U and 195U onabotulinumtoxinA (onabotA) in patients with chronic migraine (CM) from the PREDICT study. The phase 3 PREEMPT clinical trials established the safety and efficacy of 155-195U onabotA in adults with CM.

**Methods**: PREDICT (NCT02502123) was a Canadian 2-year, prospective, observational study in adults with CM. Patients received onabotA approximately every 12 weeks (≤7 treatment cycles [Tx]) per the Canadian product monograph. The primary endpoint was mean change from baseline in Migraine-Specific Quality of Life (MSQ) at Tx4. Headache days (daily headache diary), physician and patient satisfaction were evaluated throughout the study. This analysis stratified the safety population (≥1 onabotA dose) into 3 groups (155U,156-195U and 195U) by the dose receivedon ≥3 of the first 4 treatment cycles.

**Results**:Of 184 patients that received ≥1 onabotA dose, 68 received 155U, 65 received 156-195U and 13 received 195U on ≥3 treatments. Baseline characteristics were similar between groups. Baseline mean (SD) headache days/month 21.6(6.4) 155U;20(7) 156-195U; and 21.7(6) 195U decreased over time (Tx4: -7.1[6.7] 155U; -6.5[6.7] 156-195U;-11.2[6.4] 195U versus baseline). Improvements in all MSQ domains were observed across groups at Tx4 and the final visit. Physicians rated most patients as improved, and the majority of patients were satisfied at the final visit (80.8% 155U; 83.6% 156-195U; 90% 195U). Treatment-emergent adverse events (TEAEs) were reported in 18/68 patients (26.5%) in the 155U group, 41/65 (63.1%) in the 156-195U group and 10/13 (76.9%) in the 195U group; treatment-related TEAEs were 9(13.2%), 10(15.4%) and 3(23.1%) respectively; serious TEAEs were 0, 3(4.6%), and 1(7.7%), and not treatment-related.

**Conclusion**:Long-term treatment with 155U, 156-195U, and 195U onabotA in PREDICT was safe, well-tolerated, and effective in the treatment of CM. No new safety signals were identified.

## P121 The virtual "Enfacement Illusion" on pain perception in patients suffering from chronic migraine: preliminary data from a randomized controlled trial

### S. Bottiroli^1,2^, M. Matamala-Gomez^3^, M. Allena^1^, E. Guaschino^1^, N. Ghiotto^1^, R. De Icco^1,4^, G. Sances^1^, C. Tassorelli^1,4^

#### ^1^IRCCS Mondino Foundation, Pavia, Italy; ^2^Giustino Fortunato University, Benevento, Italy; ^3^University of Milan-Bicocca, Milan, Italy; ^4^University of Pavia, Pavia, Italy

##### **Correspondence:** S. Bottiroli

**Background**: Given the limited efficacy of pharmacological treatments for chronic migraine (CM), new non-pharmacological strategies have gained increasing attention. Body ownership illusions have been proposed as a non-pharmacological strategy for pain relief. Here we report the preliminary data from a randomized controlled trial (RCT) evaluating the efficacy in reducing pain perception of the enfacement illusion created through an immersive virtual reality (VR) system in CM

**Method:** Data are taken from a double-blind RCT, involving CM patients randomly assigned to the experimental or the control group. The experimental group was exposed to the enfacement illusion; whereas the control group to a pleasant immersive VR environment. Both conditions consisted in three VR sessions (20 minutes) during a one-week period. At the baseline (T0) and at the end of the intervention (T1), the patients filled in behavioral measures related to their emotional and psychological state, and body image perception. Before and after each VR session, we assessed the level of pain and the affective state of the patients.

**Results**: Twenty-five CM patients received the experimental (n=11, mean age=39.5±12.6) or the control (n=14, mean age=44.3±10.7) condition. Patients were comparable from the clinical and psychological point of view at T0. Data showed a comparable effect between the two groups in terms of pain reduction following the intervention: both the experimental and control groups achieved a significant reduction on the VAS scale within each VR session and when comparing sessions 1 and session 3. More pronounced benefits were found for the experimental group than the control group in terms of changes in the affective state between T0 and T1.

**Discussion:** These preliminary results seem to support the effectiveness of body ownership illusions as a cognitive behavioral intervention acting not only on pain relief but also on the affective state in patients with CM.

## P122 Oral Rimegepant 75 mg is Safe and Well Tolerated in Adults With Migraine and Cardiovascular Risk Factors: Results of a Multicenter, Long-Term, Open-Label Safety Study

### S. Hutchinson^1^, J. Schim^2^, R. Lipton^3^, R. Croop^4^, C. M. Jensen^4^, A. C. Thiry^4^, E. G. Stock^4^, C. M. Conway^4^, M. Lovegren^4^, V. Coric^4^, M. Hanna^4^

#### ^1^Orange County Migraine & Headache Center, Irvine, CA, United States; ^2^Headache Center of Southern California, Carlsbad, CA, United States; ^3^Albert Einstein College of Medicine, Bronx, NY, United States; ^4^Biohaven Pharmaceuticals, New Haven, CT, United States

##### **Correspondence:** S. Hutchinson and C. M. Jensen

Objective

Evaluate the safety and tolerability of rimegepant in adults with cardiovascular (CV) risk factors.

Methods

This was a multicenter, long-term, open-label safety study (NCT03266588) in adults with a history of 2-14 monthly migraine attacks of moderate to severe pain intensity. Subjects used rimegepant 75 mg up to once daily for up to 52 weeks. For this analysis, subjects were organized into subgroups by number of baseline CV risk factors (0, 1, ≥2) and Framingham 10-year risk of developing a CV condition (low = <10%, moderate to high = ≥10%).

Results

Of the 1800 rimegepant-treated subjects, 735 (40.8%) had CV risk factors (518 [28.8%] had 1 and 217 [12.1%] had ≥2]) and 126 (7.0%) had a moderate to high risk 10-year CV risk. The most common adverse events (AEs) regardless of relationship to treatment were upper respiratory tract infection (8.8%), nasopharyngitis (6.8%), and sinusitis (5.1%), and the proportion of subjects reporting ≥1 AE was similar across all subgroups (Table). No serious AEs were considered by the investigator to be related to rimegepant. Only 1 subject out of 1800, a 53 year-old male with a history of CV disease (angina pectoris), experienced an ischemic Cardiac Disorder SOC AE (angina pectoris) deemed by the investigator to be not related to rimegepant.

Conclusion

Rimegepant dosed up to once daily for up to 1 year showed favorable safety and tolerability in adults with migraine with CV risk factors, including adults with moderate to high CV risk.

**Table 1 (abstract P122).**
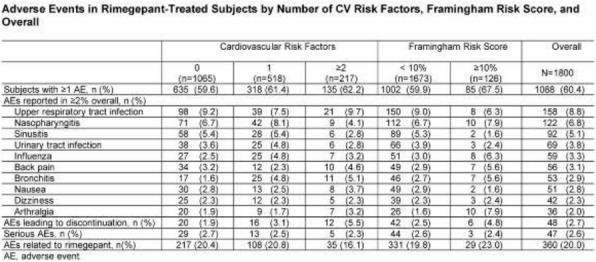
See text for description.

## P123 Medication Preference, Satisfaction, and Clinical Improvement Among Adults Receiving Long-Term Treatment With Rimegepant for Migraine

### K. Mullin^1^, J. Pavlovic^2^, S. Hutchinson^3^, R. Lipton^2^, C. M. Jensen^4^, A. C. Thiry^4^, L. Kamen^4^, V. Coric^4^, R. Croop^4^

#### ^1^New England Institute for Neurology and Headache, Stamford, CT, United States; ^2^Albert Einstein College of Medicine, Bronx, NY, United States; ^3^Orange County Migraine & Headache Center, Irvine, CA, United States; ^4^Biohaven Pharmaceuticals, New Haven, CT, United States

##### **Correspondence:** C. M. Jensen

**Objective** Assess preference for and satisfaction with open-label rimegepant as well as CGI-C over 1 year of use as a preventive treatment and acute treatment for migraine.

**Methods** This 1-year open-label extension phase of a 12-week, randomized, double-blind, placebo-controlled study (NCT03732638) included adults aged ≥18 years with a history of 4-18 moderate-severe monthly migraine attacks. Subjects completing 12 weeks of double-blind treatment with rimegepant 75 mg or placebo every other day could continue with open-label treatment with rimegepant 75 mg every other day for preventive treatment of migraine for 52 weeks; on nonscheduled dosing days, they could take rimegepant 75 mg up to once daily as needed for acute treatment. Exploratory objectives evaluated rimegepant on Preference of Medication (PoM), Satisfaction with Medication (SM), and CGI-C scales at Weeks 12 and 52 of the open-label extension phase (ie, overall study Weeks 24 and 64).

**Results** Of 741 subjects treated in the double-blind treatment phase, 603 (81.4% [rimegepant n=301, placebo n=302]) were treated in the open-label extension phase (mean age 42.6 years, 82.7% female, hx of 7.9 monthly mod-sev attacks). Percentages (95% CI) of subjects preferring rimegepant to prior migraine treatments at Week 12 (n=357) and Week 52 (n=246) were 82.6% (78.3, 86.2) and 85.2% (80.4, 89.0), respectively. Percentages (95% CI) completely satisfied with rimegepant were 32.7% (28.2, 37.6) at Week 12 and 47.2% (41.5, 53.0) at Week 52; most of the other subjects reported being very satisfied. Percentages (95% CIs) improved on the CGI-C at Weeks 12 and 52, respectively, were 95.1% (92.9, 96.7) and 98.3% (96.4, 99.2). Week 52 results are shown in the Figure.

**Conclusion** Large majorities of subjects who used open-label rimegepant for both preventive treatment and acute treatment of migraine over 1 year preferred rimegepant to prior migraine medications, were satisfied with rimegepant, and experienced clinical improvement.

**Fig. 1 (abstract 123).**
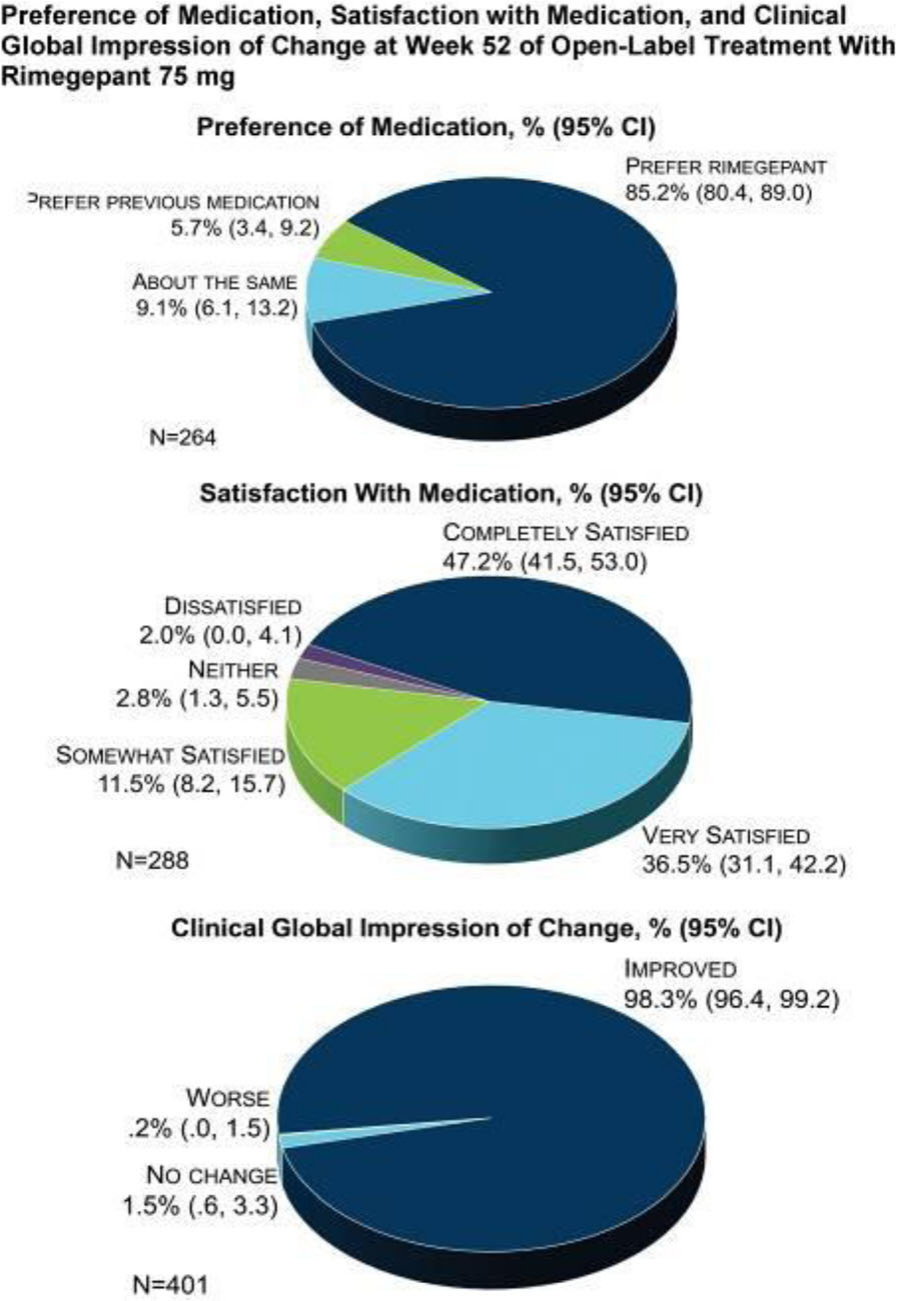
See text for description.

## P124 Characterizing gaps in preventive treatment of migraine: global results from the CaMEO-International study

### D. C. Buse^1^, F. Sakai^2^, M. Matharu^3^, M. Reed^4^, K. Fanning^5^, B. Dabruzzo^6^, R. Lipton^1^

#### ^1^Albert Einstein College of Medicine, Bronx, NY, United States; ^2^Saitama International Headache Center, Chuo-ku, Saitama City, Japan; ^3^University College London, Neurology, London, United Kingdom; ^4^Vedanta Research, Chapel Hill, NC, United States; ^5^MIST Research, Wilmington, NC, United States; ^6^AbbVie, Madison, NJ, United States

##### **Correspondence:** D. C. Buse

**Objective:** To assess self-reported use of preventive medications for migraine available in 2021 and analyze potential treatment gaps among individuals who are candidates for preventive treatment based on the American Headache Society (AHS) Consensus Statement (ie, ≥3 monthly headache days [MHDs] with severe disability, ≥4 MHDs with some disability, or ≥6 MHDs regardless of disability level).

**Methods:** CaMEO-I was a cross-sectional, observational, web-based cohort study conducted in 2021 in the US, Canada, UK, Germany, France, and Japan. Respondents who met *International Classification of Headache Disorders*, 3rd ed symptom criteria were eligible to participate in the survey assessment.

**Results:** Of 14,492 respondents with migraine included in this analysis (≈2400 from each country), 31.5-42.1% qualified for migraine preventive treatment based on the AHS Consensus Statement (US: n=976; Canada: n=794; UK: n=767; Germany: n=1010; France: n=802; Japan: n=897). In the overall sample, respondents who reported ever using a preventive medication for migraine ranged from 9.7% (Japan) to 28.9% (US). Among those who reported ever using a preventive medication, 51.1-65.8% were current users. Among current users, 70.6-97.1% used an oral preventive medication, 1.7-16.5% used an injectable preventive medication, and 1.3-20.9% used both. The majority of respondents with migraine who qualified for preventive treatment did not report currently using a preventive (77.6-89.9%); 30.0-40.8% of respondents who were not currently using a preventive qualified for preventive treatment. Of respondents who were currently using a preventive, 41.1-59.1% still qualified for preventive treatment.

**Conclusions:** More than 75% of individuals with migraine who are candidates for a preventive treatment are not currently taking a medication to prevent migraine. Of those who are currently using a preventive medication for migraine, roughly 50% are not receiving adequate benefit from their current medication.

## P125 Concomitant treatment of anti-CGRP and botulinum toxin in resistant migraine, is it useful for our patients?

### C. Nieves Castellanos, M. Olivier, M. I. Fabrich Marín, S. Díaz Insa

#### Hospital Universitari i Politécnic la Fe de Valencia, Headache Unit, Valencia, Spain

##### **Correspondence:** C. Nieves Castellanos

QUESTION

An important percentage of our patients are using monoclonal antibodies against CGRP or its receptor (a-CGRP) concomitantly with botulinum toxin. It is important to analyze how these patients evolve with both treatments.

METHODS

We investigate patients with resistant migraine treated with a-CGRP and botulinum toxin ant compare with patients with a-CGRP only. We analyzed: days of migraine (MHD), headache (HHD) and triptanes per month (MtuD) as well as scales (HIT-6, MIDAS, and quality of life (MsQol)). We compared the data at 3 and 6 months. We analyzed the wearing off from botulinum toxin in patients who are with this treatment and how many of them stopped the treatment with toxin after initiated a-CGRP.

RESULTS

336 patients were included, 215 with both treatments (64%), 121 patients with a-CGRP but without toxin (36%). Comparing results, in the group with toxin, at baseline 19 MHD with reduction of 7 MHD at 3 months and 8,3 MHD at 6 months. In the group without toxin, they had 20,3 MHD at baseline with reduction of 8,5 MHD at 3 months and 11,8 MHD at 6 months.

In the group with toxin, HIT-6 was reduced an average of 6,3 points at 6 months and MIDAS 40,2 points at 6 months. In the group with toxin, HIT-6 was reduced 12,9 points and MIDAS 58,8 points at 6 months.

At 6 months, 119 patients are with both treatments (botulinum toxin and a-CGRP). 79 of them (66%) presented wearing off from the toxin: 32 patients had more frequent migraines, 13 more intense them, and 24 patients both things.

45 patients (20,9%) stopped the treatment with botulinum toxin after 3-6 months after initiating the a-CGRP.

CONCLUSIONS

Although both groups have a significant response, the group without botulinum toxin presents better results. However, the wearing off in the group with botulinum toxin supports the use of both treatments in these patients to optimize their therapy.

## P126 The effect or resveratrol in *ex vivo* and behavioral rat models of migraine

### P. Reducha^1,2^, K. A. Haanes^1^, J. Bömers^1^, A. Holm^1^, S. Kazantzi^1,2^

#### ^1^University of Copenhagen, Rigshospitalet, Department of Clinical Experimental Research, Copenhagen, Denmark; ^2^University of Copenhagen, Rigshospitalet, Biology, Copenhagen, Denmark

##### **Correspondence:** P. Reducha

**Objective:** The activation of the trigeminovascular system (TGVS), nociception, neurogenic neuroinflammation, as well as the release of the neuropeptide calcitonin gene-related peptide (CGRP) from C-fibers in the meninges and trigeminal ganglion (TG) have been proposed to be part of migraine pathophysiology. Resveratrol is a polyphenol with therapeutic effects on various conditions and diseases, however little research has been conducted of this compound in the context of migraines, which was therefore the purpose of this study.

**Methods:** The effect of resveratrol on CGRP release was investigated in the TG and dura mater of rats, where we applied the following stimuli: KCl induced depolarization, TRPV1 activation (capsaicin) and TRPM3 activation (CIM0216), which are all CGRP release stimulants. Finally, resveratrol was tested in an *in vivo* inflammatory model, where rats were administered with Complete Freund"s Adjuvant (CFA) to their dura, followed by periorbital allodynia testing using an electronical von Frey.

**Results:** Resveratrol did not stimulate CGRP release per se. Further, resveratrol reduced capsaicin induced CGRP release in the TG by 29.9±12.1% (p=0.02), and reduced CIM0216 and KCl induced CGRP release in the dura by 32.2±2.8% (p=0.02) and 29.8±6.3% (p=0.01), respectively. Despite inhibitory effects on CGRP, two days of intraperitoneal injection of 100 mg/kg resveratrol did not alleviate periorbital allodynia in the inflammation model.

**Conclusion:** The *ex vivo* data provides arguments and encouragement for further migraine studies to investigate resveratrol as a therapeutic agent, and we postulate that this could be caused by the ability of resveratrol to potentially interact with the function of TRPV1 channels and TRPM3 channels, and thereby reduce membrane excitation in the TGVS. Although we did not observe positive effect in the *in vivo* model, we believe that with the optimal dosing resveratrol could show positive effects.

## P127 Is migraine causally linked to inflammatory bowel disease or coeliac disease? A Mendelian randomisation study

### N. Welander^1^, G. Rukh^1^, M. Rask-Andersen^2^, A. Harder^3,4^, A. van den Maagdenberg^3,4^, H. Schiöth^1^, J. Mwinyi^1^

#### ^1^Uppsala University, Department of Surgical Sciences, Uppsala, Sweden; ^2^Uppsala University, Department of Immunology, Genetics and Pathology, Uppsala, Sweden; ^3^Leiden University Medical Center, Department of Human Genetics, Leiden, Netherlands; ^4^Leiden University Medical Center, Neurology, Leiden, Netherlands

##### **Correspondence:** N. Welander

**Question:** Migraine has been linked to inflammatory bowel disease (IBD) and coeliac disease. This paper assesses whether the link may be explained by a shared genetic basis or could be causal.

**Methods:** Linkage disequilibrium score regression and two-sample bidirectional Mendelian randomisation (MR) analyses were performed using summary statistics from genome-wide association studies of migraine (59,674 cases; 316,078 controls), IBD (25,042 cases; 34,915 controls) and coeliac disease (11,812 or 4533 cases; 11,837 or 10,750 controls). Migraine with and without aura (MA and MO) were analysed separately, as were the two IBD subtypes Crohn"s disease and ulcerative colitis. Positive control analyses and conventional MR sensitivity analyses were performed.

**Results:** Migraine was not genetically correlated with IBD or coeliac disease. No evidence was observed for IBD or coeliac disease causing migraine or vice versa when all migraineurs were analysed jointly (*p* > 0.05 for all). There was some indication of causality between coeliac disease and MA (odds ratio 1.04, 95% confidence interval 1.00–1.08, *p* = 0.045) and between coeliac disease and MO (0.95, 0.92–0.99, *p* = 0.006), as well as between MO and ulcerative colitis (1.15, 1.02–1.29, *p* = 0.025). The results were, however, not significant after multiple testing correction.

**Conclusions:** We found no evidence of a shared genetic basis or of a causal association between migraine and either IBD or coeliac disease, although we obtained some indication of causality with migraine subtypes.

**Fig. 1 (abstract P127).**
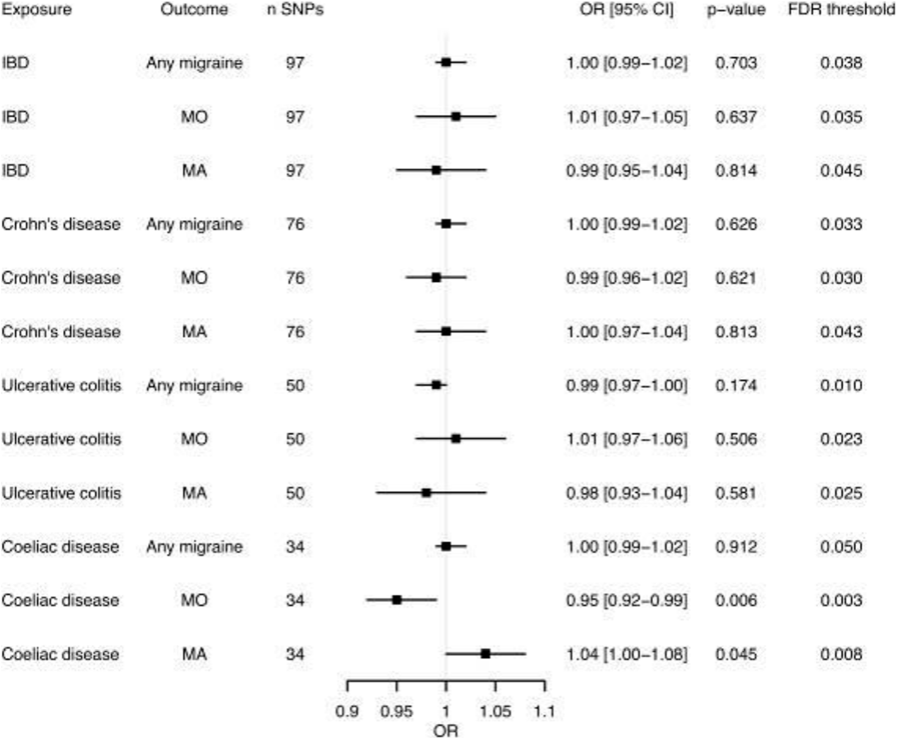
Effects of genetic liability to gastrointestinal conditions on migraine**.** Forest plot of two-sample MR effect estimates for IBD and coeliac disease on migraine based on the inverse-variance weighted method

**Fig. 2 (abstract P127).**
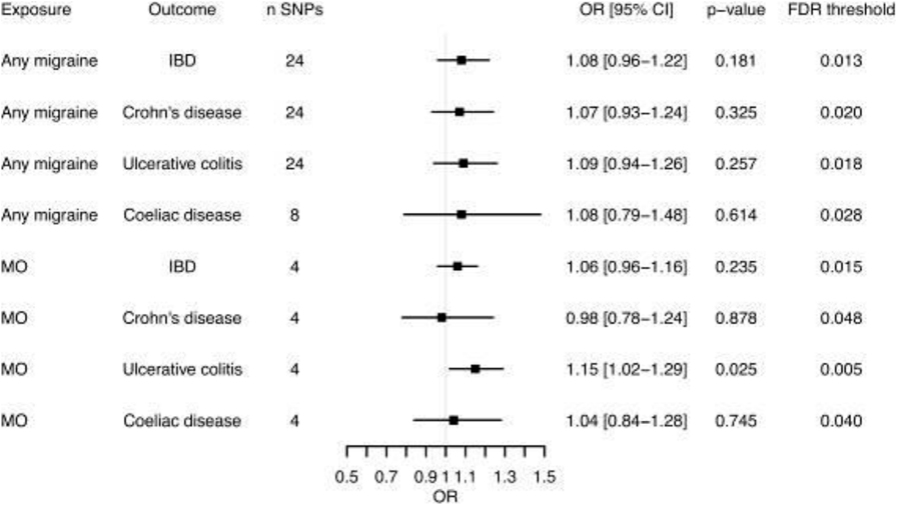
Effects of genetic liability to migraine on gastrointestinal conditions. Forest plot of two-sample MR effect estimates for migraine on IBD and coeliac disease based on the inverse-variance weighted method

## P128 Medication overuse and patient-reported outcome of *OnabotulinumtoxinA* treatment in Chronic Migraine

### C. Fernandes^1^, B. Silva^2^, J. Lopes^3^, I. Luzeiro^1,4^

#### ^1^Hospitalar and University Center of Coimbra, Neurology, Coimbra, Portugal; ^2^Hospitalar Center of Leiria, Neurology, Leiria, Portugal; ^3^Hospitalar Center of Baixo Vouga, Neurology, Aveiro, Portugal; ^4^The School of Health Technology of Coimbra, Sleep Medicine, Coimbra, Portugal

##### **Correspondence:** C. Fernandes

**Question:**

The onabotulinumtoxinA (onabotA) is an injectable preventive treatment of chronic migraine (CM), in 12 week"s intervals. The aim of our study was to evaluate the parient-reported outcome of off onabotA treatment and to study the influence of medication overuse.

**Methods**

We designed a cross-sectional study of patients with CM and at least two treatments with onabotA from August 2021 until March 2022. We proceeded to demographic and clinical characterization and evaluation of medication overuse and patient-reported outcome of onabotA response with the Patients' Global Impression of Change scale (PGICS).

**Results**

We included 60 patients (95,1% female) diagnosed with CM with a mean age of chronic migraine diagnosis of 31,8±14,2 years. In our cohort, 12 patients (21,8%) had evidence of current medication overuse and 25 (55,6%) had anxiety problems. On average, before onaBotA treatment patients had around 20,0 attacks per month. In 45,3% we noticed a therapeutical response after the first treatment and 86,2% showed a decrease in duration of headache attacks and a mean intensity of attacks decrease of 3 points in pain visual analog scale (VAS). The wearing-off effect was noticed in 36 patients (66,7%) before the next injection of onabotA and the majority between the 10th to 12th week post treatment. At the evaluation of PGICS, 20 patients (66,7%) reported be "better" or "much better" after onabotA treatment. There was no correlation between the presence of medication overuse and onabotA response (p=0,758) and between wearing-off and perception of onabotA therapeutical response according to PGICS (p=0,097).

**Conclusion**

To summarize, the presence of medication overuse does not seem to influence the onabotA response and the patient-reported outcome. Also, the wearing-off phenomena, that were noticed in the most patients, does not influence the perception of onabotA therapeutical response.

## P129 Atogepant for the Preventive Treatment of Chronic Migraine in Europe: Results From the PROGRESS Study

### P. Goadsby^1,2^, P. Pozo-Rosich^3,4^, M. Ashina^5^, U. Reuter^6,7^, X. Moisset^8^, J. Trugman^9^, H. Ha^10^, B. Schwefel^11^, K. Nagy^12^

#### ^1^King's College London, London, United Kingdom; ^2^University of California, Los Angeles, CA, United States; ^3^Vall d'Hebron University Hospital, Barcelona, Spain; ^4^Autonomous University of Barcelona, Barcelona, Spain; ^5^University of Copenhagen, Rigshospitalet, Neurology, Copenhagen, Denmark; ^6^Charité University Hospital Berlin, Berlin, Germany; ^7^Universitätsmedizin Greifswald, Greifswald, Germany; ^8^Université Clermont Auvergne, CHU Clermont-Ferrand, Inserm, Neuro-Dol, Clermont-Ferrand, France; ^9^AbbVie, Madison, NJ, United States; ^10^AbbVie, Toronto, Canada; ^11^AbbVie, North Chicago, IL, United States; ^12^AbbVie, Budapest, Hungary

##### **Correspondence:** P. Goadsby

**Objective:** Present the primary and key secondary endpoints for the Europe subpopulation in the PROGRESS trial.

**Methods:** Phase 3 multicenter, randomized, double-blind, placebo (PBO)-controlled trial (RCT) evaluated the efficacy and safety of atogepant (ATO) for prevention in people with chronic migraine (CM). Participants with ≥1-year CM history, ≥15 headache d/mo in the past 3 months, and ≥15 headache days (≥8 days qualified as migraine) during the 28-day screening period were randomized to receive ATO 30mg twice daily (BID), ATO 60mg once daily (QD), or PBO in the 12-week treatment period. In this analysis we examined a Europe subpopulation. Primary outcome was change from baseline in monthly migraine days (MMDs) across the 12-week treatment period, and the key secondary outcome was proportion of participants with ≥50% reduction in 3-month MMD average.

**Results:** From the safety population (n=773; female, 87.6%, mean age, 42.1y), 760 individuals were included in the off-treatment hypothetical estimand population and 269 were included in the Europe subpopulation (PBO n=88, ATO 30mg BID n=91, ATO 60mg QD n=90). Least square (LS) mean change in MMDs was −8.44 in the ATO 30mg BID and −8.00 ATO 60mg QD groups compared to −5.42 in the PBO group. LS mean difference [95% CI] vs PBO was greater in both groups (ATO 30mg BID: −3.02 [−4.82, −1.22]; ATO 60mg QD: −2.59 [−4.39, −0.79]). A higher proportion of ATO 30mg BID (48.4%; OR [95% CI]: 1.87 [1.01, 3.44]; nominal *P*=0.0457) and ATO 60mg QD (46.7%; OR [95% CI]: 1.84 [1.00, 3.41]; nominal *P*=0.0511) participants had a ≥50% reduction in 3-month average of MMDs compared to PBO (33.0%).

**Conclusions:** In the Europe subpopulation, both ATO doses demonstrated significantly higher reductions in mean MMDs and proportions of ≥50% responders in MMD reduction over 3 months vs PBO.

## P130 Additional effects of pain neuroscience education combined with physiotherapy on the headache frequency of adult patients with migraine – a randomized controlled trial

### R. Meise^1^, G. F. Carvalho^1^, C. Thiel^2^, K. Luedtke^1^

#### ^1^University of Luebeck, Department of Physiotherapy, Pain and Exercise Research Luebeck (P.E.R.L), Luebeck, Germany; ^2^University of Applied Sciences, Department of Applied Health Sciences, Bochum, Germany

##### **Correspondence:** R. Meise

Aim:To assess the efficacy of pain neuroscience education (PNE) combined with physiotherapy (PT) compared to physiotherapy alone for the management of migraine.

Background: Physiotherapy can significantly reduce the intensity and frequency of migraine, but the level of evidence is low. PNE might pose a promising treatment for migraine patients, as it addresses migraine as a chronic pain disease.

Methods: In this randomized controlled trial, patients with migraine received PT+PNE or PT alone. The primary outcomes were reduction of headache frequency (days/month) migraine days and migraine associated disability. Secondary outcomes included *migraine* specific quality of life, depression, neck pain and the acquired knowledge on the neurophysiology of pain. The treatments were preceded by a three-month waiting period during which a headache diary was kept. A two-way repeated ANOVA was used to assess between- and within-subjects factors and interactions, including group and time for baseline, post-treatment and 3-month follow-up.

Results: 82 patients participated in the study and showed a significant decrease of headache frequency post-treatment and at 3-months follow-up (F2,158 = 4.12, *p* = 0.02) (effect size d= 0.46). There was no difference between groups (F2,158 = 1.35, *p* = 0.26). Frequency of migraine days, only, showed a significant difference between groups (F2,158= 5.04, *p* = 0.008) with a greater reduction in the PT+PNE group (medium effect size d= 0.5). Migraine associated disability showed a significant decrease at 3-months follow-up (strong effect size d= 1.15) (F1,80 = 24.08, *p* < 0.001 (d= 1.15) and no difference between groups (F1,80 = 0.30, *p* = 0.583). Secondary outcomes demonstrated a significant effect of time with no interaction between time and group.

Conclusion: PNE does not significantly add to the effect of physiotherapy regarding the reduction of headache frequency and migraine associated disability but may reduce the number of migraine days.

**Fig. 1 (abstract P130).**
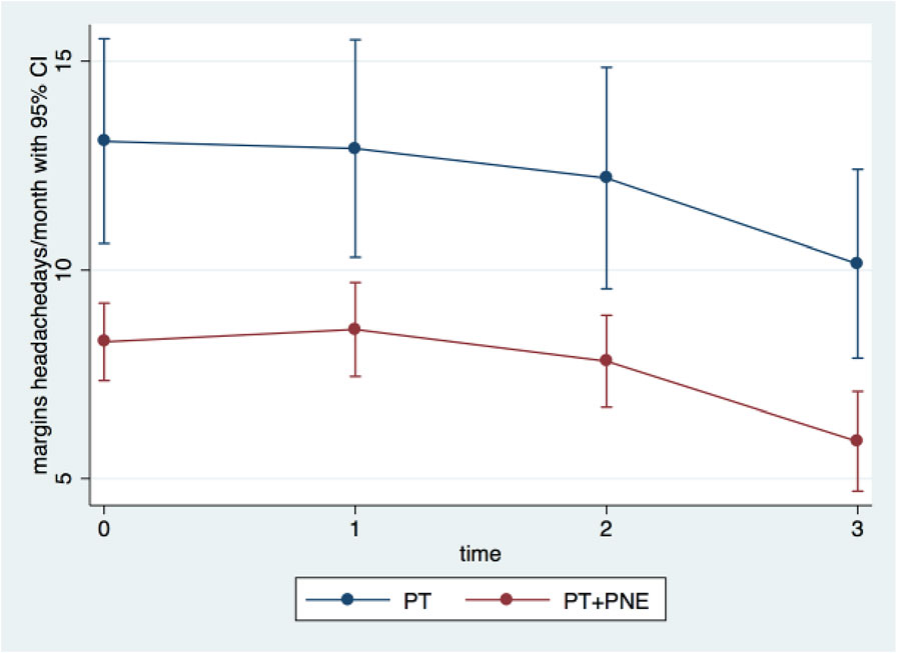
See text for description.

**Fig. 2 (abstract P130).**
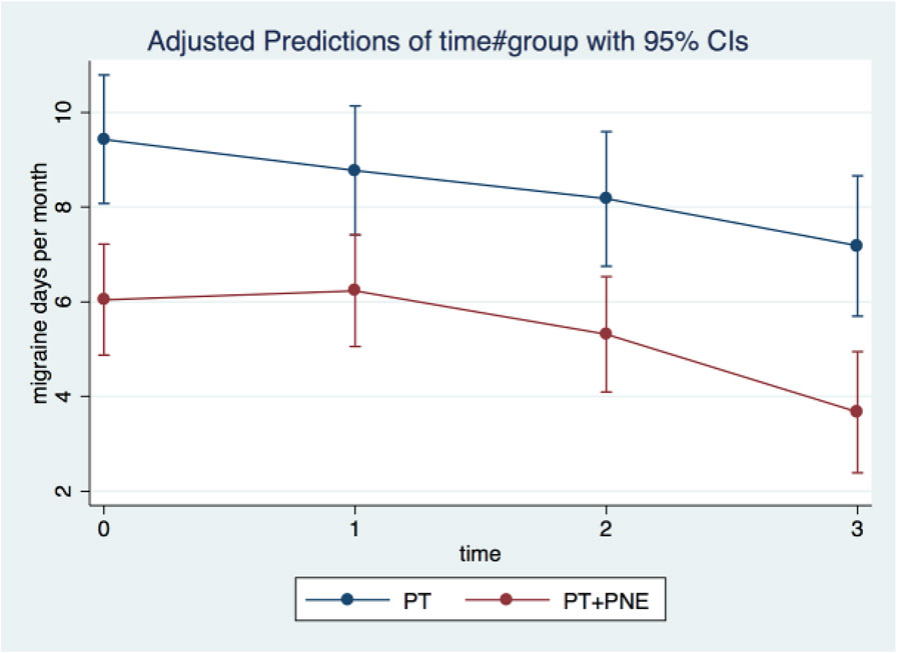
See text for description.

## P131 Assessing Hypersensitivity, Cortical Hyperexcitability, and Habituation in Migraine, according to Age and Disease Severity, using Visual Evoked Potentials during Pattern-Reversal Stimulation

### A. Marti-Marca^1^, A. Vilà-Balló^1^, X. Cerdá-Company^1^, N. Ikumi^1^, M. Torralba^2^, M. Torres-Ferrús^1,3^, E. Caronna^1,3^, V. J. Gallardo^1^, A. de la Torre Suñe^1^, A. Alpuente^1,3^, S. Soto-Faraco^2,4^, P. Pozo-Rosich^1,3^

#### ^1^Vall d'Hebron Research Institute, Headache and Neurological Pain, Barcelona, Spain; ^2^Center for Brain and Cognition, Universitat Pompeu Fabra, Multisensory Research Group, Barcelona, Spain; ^3^Vall d'Hebron University Hospital, Neurology, Barcelona, Spain; ^4^Institució Catalana de Recerca i Estudis Avançats (ICREA), Barcelona, Spain

##### **Correspondence:** A. Marti-Marca

**Objective:** Our goal was to test whether hypersensitivity, cortical hyperexcitability, and hyperresponsivity (lack of habituation) are typical of migraine interictally and whether inconsistencies might be attributed to age or disease severity.

**Method:** Two experiments were carried out on: (1) 18 young patients (22.8±1.89 years) with low-frequency episodic migraine (3.4±3.35 headache days/month) and 27 matched controls (21.8±2.03 years); and (2) 16 middle-aged patients (41.8±9.21 years) with high-frequency episodic migraine (12.4±4.30 headache days/month) and 29 matched controls (39.2±8.84 years). A neurologist confirmed the diagnoses. We obtained migraine phase (using eDiaries), Sensory Perception Quotient (SPQ) scores, and PR-VEPs (N1, P1). Interictal data was analysed; initial sample: 23(1) and 57(2) patients. The SPQ was used to measure hypersensitivity, Group differences in P1-N1 amplitude denoted cortical hyperexcitability, and habituation was defined as a decrease of P1-N1 amplitude across Blocks/Trials. P1-N1 Peak-to-Peak amplitudes were analysed with linear mixed models considering Block (100 trials/Block) or Trial (all trials) and Group.

**Results:** Patients reported increased sensitivity to visual stimuli on the SPQ ((1)p=0.010; (2)p=0.017) compared to controls. Regarding P1-N1 data, there was no significant main effect of Group in either (1) or (2), ruling out cortical hyperexcitability. Significant interactions between Block-x-Group ((1)p<0.012;(2)p=0.005) and Trial-x-Group ((1)&(2)p<0.0001) were observed. Post-hoc tests indicated habituation both in patients, regardless of age and headache frequency ((1)&(2)p<0.0001), and controls ((1)p=0.001;(2)p<0.0001). Patients showed a sharper habituation slope than controls ((1)p=0.0001;(2)p<0.0001).

**Conclusion:** Hypersensitivity to visual stimuli was not related to cortical hyperexcitability or interictal habituation using PR-VEPs; these findings did not vary based on age or disease severity in episodic migraine.

## P132 Effects of vitamin B1, B6, and B12 on serum levels of CGRP, endothelial nitric-oxide synthase, homocysteine, and headache characteristics in women with episodic migraine

### S. Nematgorgani^1^, M. Togha^1^, S. Razeghi Jahromi^2^, E. Jafari^1^

#### ^1^Tehran University of Medical Sciences, Headache Department, Tehran, Iran; ^2^Shahid Beheshti University of Medical Sciences, Department of Clinical Nutrition and Dietetics, Tehran, Iran

##### **Correspondence:** S. Nematgorgani

**Question:** Migraine is a common, painful, and debilitating disease. Previous studies have shown that B vitamins especially vitamin B2 are beneficial in treating pain and migraine; however, the mechanism of their actions, and also the effects of other vitamin B supplements remain unclear. The purpose of the present study was to examine the effects of vitamin B1, B6, B12 supplementation versus placebo on serum levels of CGRP, endothelial nitric-oxide synthase, homocysteine and headache characteristics in women with episodic migraine (EM).

**Methods:** This double-blind, placebo-controlled, randomized clinical trial study included 80 episodic migraineurs who randomly assigned into four equal groups to receive either daily dose of vitamin B6 (80 mg), B12 (500 μg), B1 (300 mg) or placebo for 12 weeks. At baseline and after the trial, general characteristics, biochemical factors, anthropometric measurements, dietary intake, physical activity and headache diaries form were collected. CGRP, eNOS, and homocysteine levels were measured using an ELISA kit before and after the intervention.

**Results:** 64 patients completed the study. After controlling baseline values and confounders supplementation with vitamin B6 reduced serum levels of CGRP compared to placebo (P <0.047) and B12 groups (P <0.008). Each of the B1, B12 and B6 supplements resulted in a decrease in the mean severity of headache attacks compared to the placebo group (P<0.001, P<0.006, P<0.001). The number of headache days went down significantly in only groups B1 and B12 (P<0.022, P<0.004). In contrast, the duration of headache attacks did not differ significantly among the groups.

**Conclusion:** We found that supplementation with vitamins B1, B12, and B6 improved migraine characteristics. The effect of vitamin B6 at least partly seems to be due to decreasing serum levels of CGRP in patients with episodic migraine. Further research is needed to determine the mechanisms of action of other vitamins on migraine.

## P133 Quantifying aversion thresholds to light, sound, smell, and touch in migraine: A longitudinal study in migraine and non-headache controls

### N. Ikumi^1^, A. Marti-Marca^1^, A. Vilà-Balló^1^, X. Cerdá-Company^1^, A. Torre-Suñe^1^, V. J. Gallardo^1^, A. Alpuente^2^, E. Caronna^1^, P. Pozo-Rosich^1,2^

#### ^1^Headache and Neurological Pain Research Group, Vall d'Hebron Research Institute, Department of Medicine, Universitat Autònoma de Barcelona, Barcelona, Spain; ^2^Vall d'Hebron University Hospital, Neurology, Barcelona, Spain

##### **Correspondence:** N. Ikumi

**Question**

To prospectively and longitudinally quantify sensory aversion thresholds to light, sound, smell (smoked, lavender, vanilla), and brush touch in migraine patients and investigate whether they are modulated by headache intensity and phase of the migraine cycle.

**Methods**

In the laboratory, we precisely quantified sensory aversion thresholds on a daily basis over the course of 27 days. A 2AFC (decision whether the presented stimulus was perceived as bothersome or not) using an adaptive procedure or a rating scale, was used to estimate the aversion of each stimulus (white light, 1000 Hz sounds, smoked, lavender, and vanilla smells, and cutaneous light brush). Besides headache intensity, we also controlled for various factors daily such as menstruation, medication intake, sleep quality, and participant anxiety.

**Results**

We included six episodic migraine patients (between 2 and 13 headache days/month) and two headache-free controls that were gender-(100% females) and age-(W=1, p=0.12) matched. We found that aversion to light (p.adj<0.01), sound (p.adj<0.01), smell (smoked; p.adj=0.01, vanilla; p.adj<0.01, lavender; p.adj=0.01), and touch (p.adj<0.01) increased with headache intensity in migraine. However, aversion thresholds in migraine compared to controls were only differentially modulated at certain phases of the migraine cycle for the tested sensory modalities.

**Conclusions**

Aversion thresholds of various sensory modalities change alongside headache intensity in patients with migraine; enhancing our understanding of the presence of multiple sensory modality fluctuations throughout the migraine cycle.

Figure 1. Example of one participant's data. Z-scores of the measured aversion thresholds/scores, anxiety-state, and sleep quality over 27 days. Missing data was filled in black, while presence of menstruation/medication intake were filled in grey. Colours of the z-scores for the auditory/visual thresholds were inverted to ease interpretation.

**Fig. 1 (abstract P133).**
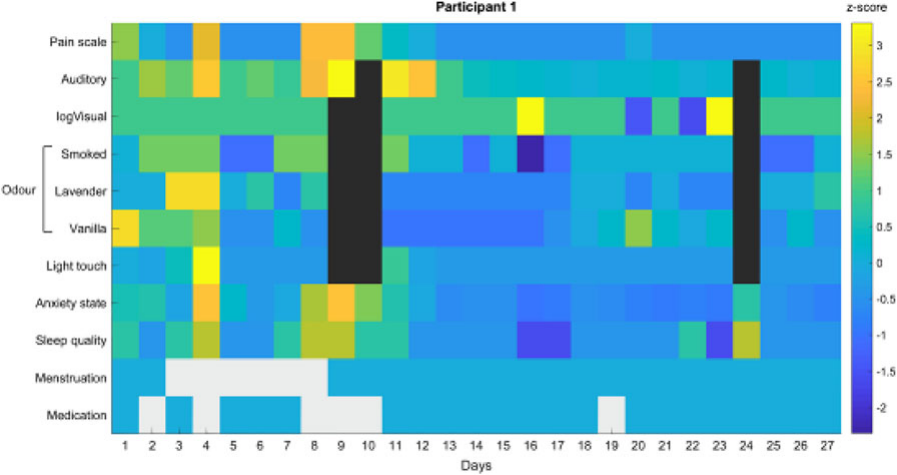
See text for description.

## P134 Negative impact of under-diagnosed migraine in university students in Slovakia

### Duraníková O, Horváthová S, Valkovič P

#### 2nd Department of Neurology, Comenius University Faculty of Medicine and University Hospital in Bratislava, Bratislava, Slovakia

##### **Correspondence:** O. Duraníková and S. Horváthová

**Introduction:** Migraine is prevalent and disabling disorder affecting more than 1 bilion people worldwide. Despite its increasing prevalence, socioeconomic impact and modern prophylactic treatment, migraine remains under-diagnosed and under-treated. Acording to the Consensus statement endorsed by the EHF and EAN, preventive treatment for migraine is recommended for patients adversely affected in ≥2 days per month despite optimized treatment. The aim of our study was to determine the proportion of undiagnosed and under-treated patients with prophylactic therapy among migraine sufferers in university students. **Method:** We screened 472 university students (356 women,age 22.0 ± 2.4 years) of Comenius University in Bratislava via an online questionnaire for any type of headache. Subsequently, we searched for migraine using diagnostic criteria according to ICHD-3. In positive patients we evaluated their average number of days with migraine per month and we asked if they have ever seen any specialist because of migraine. **Results:** 29.5% (n=139) of students fulfilled migraine criteria. 56.9% of (n=79) students have never been examined by specialist, 28.1% (n=39) were examined by neurologist, 5.8% (n=8) by GP, 9.4% by more than one specialist. We identified 85.6% (n=119) of students with ≥2 days of migraine per month, 25.2% with 2-7 days, 15.8% with 7-14 days, 38.1% with >14 days. In our cohort no patient was treated for migraine prophylaxis. **Conclusion:** We confirmed under-diagnosed migraine in more than half of students, less than 1/3 underwent neurological examination. We found more than 2/3 of students with ≥2 days with migraine per month. Despite fulfilling criteria, none of the students were using prophylactic treatment. Therefore screening of migraine patients by neurologists can improve accurate diagnosis and immediate initiation of migraine prophylaxis can lead to reduction of monthly migraine days, reduced need for acute medications and improve quality of their lives.

## P135 Change in Migraine Diagnosis After Preventive Treatment With Eptinezumab: Post Hoc Analysis of the PROMISE Studies

### P. Pozo-Rosich^1^, D. Dodick^2^, A. Ettrup^3^, J. Hirman^4^, R. Cady^5,6^

#### ^1^Vall d’Hebron University Hospital and Autonomous University of Barcelona, Barcelona, Spain; ^2^Mayo Clinic, Scottsdale, AZ, United States; ^3^Lundbeck, Copenhagen, Denmark; ^4^Pacific Northwest Statistical Consulting, Inc, Woodinville, WA, United States; ^5^Lundbeck, Bothell, WA, United States; ^6^RK Consults, Ozark, MO, United States

##### **Correspondence:** P. Pozo-Rosich

**OBJECTIVE:** To identify the proportions of patients shifting from higher to lower levels of headache frequency over Months 1-6 of treatment in the PROMISE studies.

**METHODS:** Headache frequency was categorized into 4 groups: chronic migraine (CM; ≥15 monthly headache days [MHDs]), high-frequency episodic migraine (HFEM; 10–14 MHDs), low-frequency episodic migraine (LFEM; 4–9 MHDs), and very-low-frequency episodic migraine (VLFEM; ≤3 MHDs). Outcomes included the percentage of patients within each MHD group and the percentage of patients improving by ≥1 diagnostic category.

**RESULTS:** At PROMISE-1 baseline, most patients were classified as having HFEM (100mg, 46.2% [102/221]; 300mg, 48.2% [107/222]; placebo, 51.4% [114/222]) or LFEM (100mg, 46.6% [103/221]; 300mg, 42.8% [95/222]; placebo, 42.3% [94/222]). In total, 35.7% (100mg, 79/221), 37.4% (300mg, 83/222), and 30.6% (placebo, 68/222) of patients had 6 months with reduction of ≥1 diagnostic category.

At PROMISE-2 baseline, all patients treated with eptinezumab 100mg (356/356) and placebo (366/366) groups experienced ≥15 MHDs, as did 99.4% (348/350) of patients treated with 300mg. In total, 43.0% (100mg, 153/356), 48.3% (300mg, 169/350), and 31.7% (placebo, 116/366) of patients had 6 months with reduction of ≥1 diagnostic category.

**CONCLUSIONS:** Patients treated with eptinezumab reported more downward shifts in diagnostic frequency category in Month 1 and sustained or improved this shift through Month 6 of treatment than placebo.

## P136 Hormonal treatment for menstrual migraine: rationale and protocol of the WHAT!-Trial

### B. van der Arend^1,2^, I. Vehagen^1,2^, D. van Casteren^1^, A. Maassen van den Brink^2^, G. Terwindt^1^

#### ^1^Leiden University Medical Center, Neurology, Leiden, Netherlands; ^2^Erasmus Medical Center, Internal Medicine, Rotterdam, Netherlands

##### **Correspondence:** B. van der Arend

**Introduction** – Currently, there is no evidence-based hormonal treatment for migraine in women. Several small studies suggested a beneficial effect of hormonal contraceptives, but no large randomized controlled trial has been performed. As proof of efficacy is lacking and usage may be accompanied by potentially severe side effects, there is a great need for research on this topic. In a small study a beneficial effect of vitamin E with respect to pain severity and functional disability was described, which was suggested to be mediated by a reduction of prostaglandin production in the endometrium.

**Objectives** – To study the efficacy of continuous daily use of ethinylestradiol/levonorgestrel (30/150 μg/day) compared to vitamin E (400 IU/day) in the treatment of menstrual migraine.

**Methods** – Women with menstrual migraine (n=180) are randomly assigned (1:1) to ethinylestradiol/levonorgestrel 30/150μg or vitamin E 400IU. The study is open-label since we consider it practically and ethically not feasible to blind participants. Vitamin E is chosen as an active comparator. Participants start with a baseline period of 4 weeks, which is followed by a 12-week treatment period. During the study period, participants fill out our headache E-Diary, which is time-locked and includes an automated algorithm differentiating headache and migraine days based on ICHD-3 criteria. The Stanford Expectations of Treatment Scale (SETS) will be used to help assess expectancy effects of both interventions.

**Results** – Primary outcome will be change in monthly migraine days (MMD) from baseline (week -4 to 0) to the last 4 weeks of treatment (weeks 9-12). Secondary outcomes will be change in monthly headache days (MHD), and 50% responder rates of MMD and MHD.

**Conclusion** – The WHAT!-Trial aims to investigate superiority of continuous oral contraceptive treatment for menstrual migraine. Results may be implemented in clinical practice at short notice.

Trial registration: Clinical trials.gov NCT04007874

## P136a Efficacy and Safety of Zavegepant Nasal Spray for the Acute Treatment of Migraine: Results of a Phase 3 Double-Blind, Randomized, Placebo Controlled Trial

### K. Mullin^1^, R. Croop^2^, J. Pavlovic^3^, L. Mosher^2^, T. Smith^4^, J. Madonia^2^, M. Lovegren^2^, V. Coric^2^, R. Lipton^3^

#### ^1^New England Institute for Neurology and Headache, Stamford, CT, United States; ^2^Biohaven Pharmaceuticals, New Haven, CT, United States; ^3^Albert Einstein College of Medicine, Bronx, NY, United States; ^4^Study Metrix Research, Saint Peters, MO, United States

##### **Correspondence:** R. Croop

Objective

Compare the efficacy and safety of zavegepant nasal spray with placebo in the acute treatment of migraine.

Methods

In this phase 3, double-blind, randomized, placebo-controlled trial (NCT04571060), adults with a history of 2-8 moderate or severe monthly migraine attacks self-administered 1 dose of zavegepant 10 mg nasal spray or placebo to treat 1 migraine attack of moderate or severe pain intensity. The co-primary endpoints were 2-hour freedom from pain and the most bothersome symptom (MBS).

Results

Of 1405 randomized subjects, 1269 (mean age 41 years, 83% female) were evaluable for efficacy (zavegepant n=623, placebo n=646). Zavegepant was superior to placebo for 2-hour freedom from pain (23.6% vs 14.9%, *P*<.0001) and 2-hour MBS freedom (39.6% vs 31.1%, *P*=.0012). Secondary endpoints included pain relief at 15 minutes (15.9% vs 8.0%, *P*<.0001) and 2 hours (58.7% vs 49.7%, *P*=.0012); return to normal function at 30 minutes (10.5% vs 6.1%, *P*=.0059) and 2 hours (35.8% vs 25.6%, P=.0001); and sustained pain relief 2 to 48 hours (36.1% vs 29.6%, *P*=.013) postdose. Figure 1 summarizes outcomes for the coprimary and secondary endpoints; Figure 2 presents pain relief from 15 minutes through 2 hours postdose. The most common (≥2%) adverse events (zavegepant vs placebo) were dysgeusia (20.5% vs 4.7%), nasal discomfort (3.7% vs .8%), and nausea (3.2% vs 1.1%). Most adverse events were mild or moderate; none were serious.

Conclusions

Zavegepant nasal spray was effective for the acute treatment of migraine, achieving its coprimary endpoints and providing a rapid onset of pain relief as early as 15 minutes postdose, sustained benefits to 48 hours postdose, and favorable safety and tolerability.

**Fig. 1 (abstract P136a).**
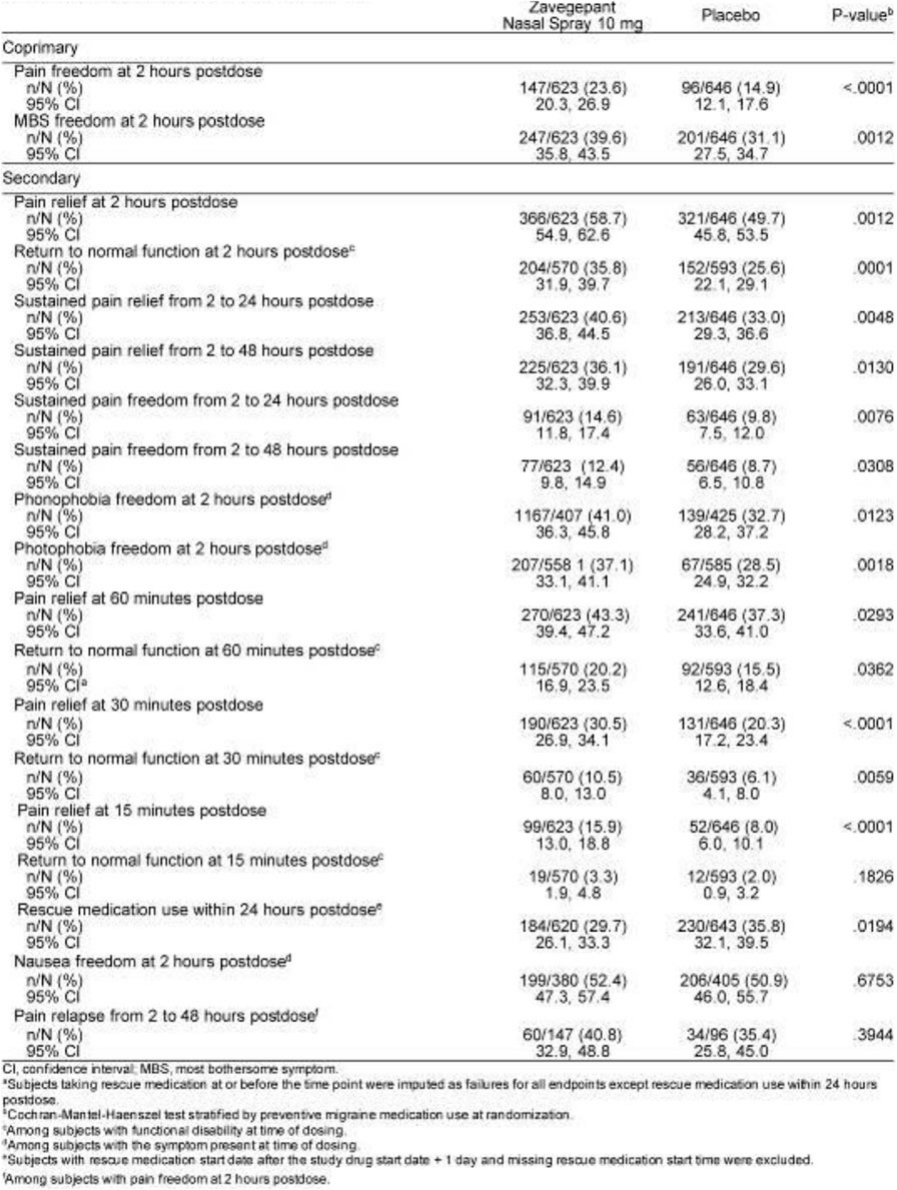
Zavegepant Nasal Spray versus Placebo in the Acute Treatment of Migraine: Summary of Results on Coprimary and Secondary Efficacy Endpoints.^*^

**Fig. 2 (abstract P136a).**
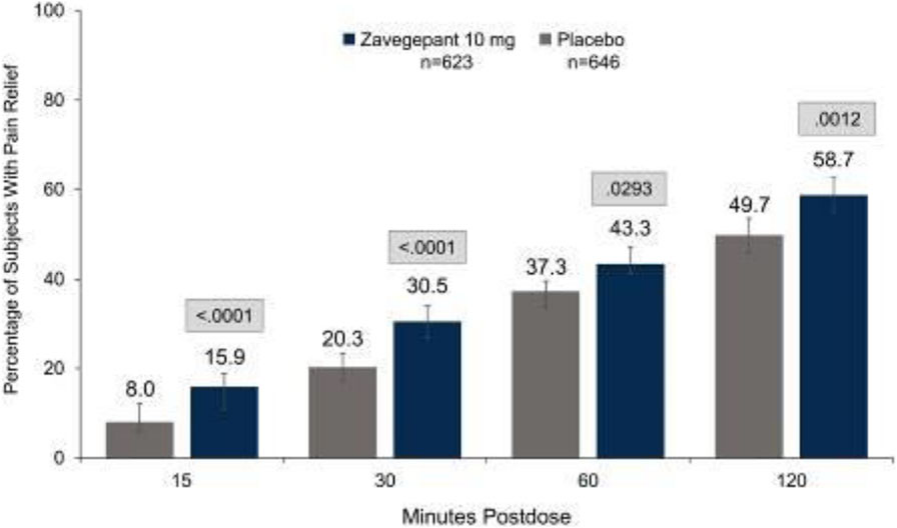
Zavegepant Nasal Spray Versus Placebo in the Acute Treatment of Migraine: Pain Relief at 15, 30, 60, and 120 Minutes Postdose

## P137 Identification of crystal-clear days in migraine using data of a nation-wide population-based study

### W. Lee^1,2^, S. J. Cho^3^, H. Hwang^4^, M. Lee^5^, E. H. Kim^5^, K. M. Kim^2^, K. Heo^2^, K. Jang^2,6^, M. K. Chu^2^

#### ^1^Yongin Severance Hospital, Neurology, Yongin, South Korea; ^2^Yonsei University College of Medicine, Neurology, Seoul, South Korea; ^3^Hallym University College of Medicine, Neurology, Hwaseong, South Korea; ^4^Yonsei University Wonju College of Medicine, Neurology, Wonju, South Korea; ^5^Yonsei University College of Medicine, Biomedical Systems Informatics, Seoul, South Korea; ^6^Jangseong Hospital, Neurology, Jangseong-gun, South Korea

##### **Correspondence:** W. Lee

**Question:** Headache-free days are not equal to migraine symptom-free days because migraine affects individuals during the headache-free period. We tried to investigate and differentiate them. Crystal-clear days can be characterized by days without headache and having minimal or no migraine symptoms. In contrast, days without headache, but with more than minimal migraine symptoms, can be defined as unclear days.

**Methods:** Participants with migraine and non-migraine headache were investigated, using the data of the Circannual Change in Headache and Sleep study, a nation-wide population survey on headache and sleep. Cross-sectional and case–control analyses were done. The number of crystal-clear days per 30 days was assessed by asking "How many days have you had crystal-clear days without headache during the previous 30 days?" We defined headache-free, but not crystal-clear days, as unclear days. The number of unclear days per 30 days was calculated as follows: 30 – the number of headache days per 30 days – the number of crystal-clear days per 30 days.

**Results:** Of 170 participants with migraine, 165 (97.1%) had unclear days. The numbers of crystal-clear days (median and interquartile range, 20.0 [15.0 – 25.0] vs. 25.0 [20.0 –29.0], *p*<0.001) and unclears days (4.0 [0.0 – 8.0] vs. 1.0 [0.0 – 7.0], *p*<0.001) per 30 days in participants with migraine were significantly lower and higher, respectively than in those with non-migraine headache. Headache days (incident rate ratio and 95% confidence interval, 0.94 [0.90 – 0.97], *p*<0.001) and weekly average sleep duration (0.95 [0.91 – 1.00], *p*=0.035) were significant factors for crystal-clear days in participants with migraine.

**Conclusions:** The number of crystal-clear days were different from that of headache-free days. Almost all participants with migraine had unclear days. Our findings will facilitate understanding the symptoms and burden of migraine.

**Fig. 1 (abstract P137).**
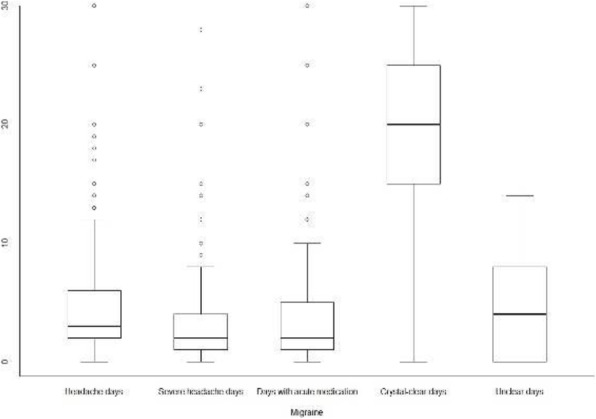
See text for description.

**Fig. 2 (abstract P137).**
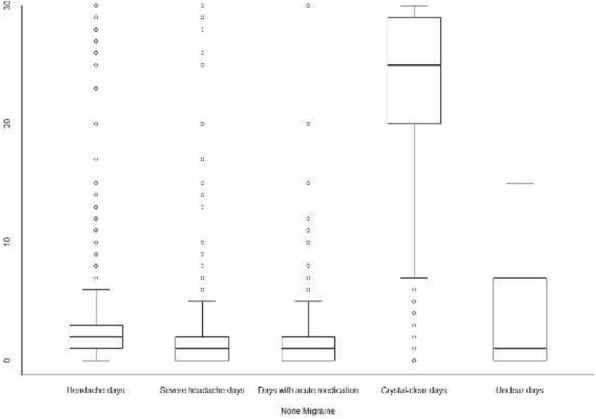
See text for description.

## P137a Long-Term Treatment With Lasmiditan in Patients With Migraine: Results From the Open-Label Extension of the CENTURION Study

### M. Ashina^1^, C. Roos^2^, D. Ayer^3^, D. Ruff^3^, J. Krege^3^, L. Q. Li^3^, M. Komori^4^

#### ^1^University of Copenhagen, Rigshospitalet, Dept. of Neurolog, Copenhagen, Denmark; ^2^Lariboisiere Hospital, Assistance Publique - Hôpitaux de Paris, Paris, France; ^3^Eli Lilly and Company, Indianapolis, IN, United States; ^4^Eli Lilly and Company, Hyogo, Japan

##### **Correspondence:** M. Ashina

**Background:** This OLE collected data for ≤1 year about dose optimization, patterns of use, migraine-related disability, and quality of life during lasmiditan treatment.

**Methods:** Patients (pts) who completed the CENTURION study (EUDRACT: 2018-001661-17 / NCT: NCT03670810) started lasmiditan 100 mg; dose may adjust to 50/200 mg. Migraine Disability Assessment

(MIDAS) and Migraine Specific Quality of Life Questionnaire (MSQ) were used.

**Results:** In all, 445 (intention-to-treat) treated ≥1 attack with lasmiditan. 8654 of 11327(76.4%) attacks were treated with lasmiditan (84.9% were moderate/severe pain). Reasons for not treating with lasmiditan were planning to drive or operate machinery (8% of attacks) or thought another medication would work better (6% of attacks). Most pts (47.0%) remained on 100 mg. Mean improvements in MIDAS Total Score was -13.0(24.9) at month 12. Mean improvement in the MSQ total score was 11.3(19.4) at month 12. Treatment-emergent adverse events (TEAEs) reported in ≥5% of pts included dizziness, paresthesia, fatigue, nausea, vertigo, somnolence, and asthenia. Most TEAEs were mild/moderate in severity. Four (0.9%) pts reported a serious TEAE; 1(0.2%) self-reported case of serotonin syndrome lasting 1 hr:40 min not requiring intervention was considered related to lasmiditan.

**Conclusions:** In the relatively real-world conditions, lasmiditan therapy was associated with a high completion rate (72.1%). Most attacks were treated with lasmiditan and remained on 100 mg throughout. Pts showed improvements in migraine-related disability and quality of life. There were no new safety findings.

## P138 Patterns of use of monoclonal antibodies for the preventive treatment of migraine: Results from the OVERCOME (EU) study

### J. Pascual^1^, D. Novick^2^, T. Panni^3^, G. Dell Agnello^4^, S. Evers^5^, S. Gonderten^6^

#### ^1^Hospital Universitario Marqués de Valdecilla and University of Cantabria, Santander, Spain; ^2^Eli Lilly and Company Ltd., Bracknell, United Kingdom; ^3^Eli Lilly Deutschland GmbH, Bad Homburg, Germany; ^4^Eli Lilly Italia SpA, Sesto Fiorentino, Italy; ^5^University of Münster, Münster, Germany; ^6^Eli Lilly and Company, Dubai, United Arab Emirates

##### **Correspondence:** S. Evers and S. Gonderten

**Question**

The aim of this analysis was to investigate the reasons for starting, stopping or switching treatment with calcitonin gene-related peptide (CGRP) monoclonal antibodies (mAbs) for migraine prevention in the European ObserVational survey of the Epidemiology, tReatment and Care of MigrainE (OVERCOME [EU]) study.

**Methods**

Data were obtained from a cross-sectional web-based survey (2020-2021). Adult respondents fulfilled International Classification of Headache Disorders (ICHD)-3 criteria for migraine or had a self-reported physician diagnosis. Respondents who ever used mAbs (erenumab, fremanezumab, galcanezumab) were considered in this analysis. Reasons to start, stop, or switch treatment were collected and summarised using descriptive statistics.

**Results**

Of 20,756 respondents, 2167 (10.4%) had used one or more mAbs. Among users of mAbs, the mean (standard deviation [SD]) age was 32.9 (10.4) years, 38.8% were female, and mean (SD) headache days per month was 3.4 (4.4). A total of 333 (15.4%) had switched and 1189 (54.9%) had stopped. No dominant reasons for starting mAbs could be identified (all reported as 15-20%). The 3 most common reasons for switching were recommendation from the doctor (27.0%) or a friend/family member (26.7%), and preference for the injector/needle used (26.7%). Reasons for stopping included improvement in headaches, recommendations from others, dosage, or tolerability. Only 11.5% stopped their medication because it was not working.

**Conclusions**

Reasons for starting mAbs were multiple, including physician recommendation and patient efficacy expectations. The finding that recommendation from others was the most frequent reason for switching highlights the importance of the patient-physician relationship and family support in the management of migraine.

## P139 Cardiovascular risk factors and migraine: Results from the population-based Rotterdam study

### L. Al-Hassany^1^, C. Acarsoy^2^, M. K. Ikram^2,3^, D. Bos^2,4^, A. Maassen van den Brink^1^

#### ^1^Erasmus Medical Center, Internal Medicine, Rotterdam, Netherlands; ^2^Erasmus Medical Center, Department of Epidemiology, Rotterdam, Netherlands; ^3^Erasmus Medical Center, Neurology, Rotterdam, Netherlands; ^4^Erasmus Medical Center, Department of Radiology & Nuclear Medicine, Rotterdam, Netherlands

##### **Correspondence:** L. Al-Hassany

**Objective** Migraine is associated with cardiovascular (CV) events. Interestingly, less is known about the link between CV risk factors and migraine, and the role of sex herein. Therefore, we conducted this study to investigate the association between the lifetime prevalence of migraine and CV risk factors in both sexes.

**Methods** In 7266 participants from the population-based Rotterdam Study (median age 66.6 [IQR 56.4−74.8], 57.5% female), we assessed migraine using a structured interview. Migraine patients were matched by age to individuals without migraine (ratio 1:3). We performed univariable and multivariable conditional logistic regression analyses on the association of CV risk factors and migraine, stratified for sex. In the first model we included clinical risk factors: current smoking, obesity, hypercholesterolemia, hypertension, and diabetes mellitus (DM). The second model aimed to provide insights into the contribution of separate components of the CV system, including smoking status (former/current), total cholesterol, high-density lipoprotein, triglycerides, systolic and diastolic blood pressure (BP), body mass index, and DM. Both models were additionally adjusted for alcohol intake and physical activity.

**Results** From the 7266, 1085 had active or a history of migraine. We found that current smoking was related to a lower migraine prevalence in females (Odds Ratio (OR) 0.72, 95% CI 0.58-0.90). Also, a higher diastolic BP related to a slightly higher prevalence of migraine in females only (OR 1.11, 95% CI 1.02-1.20). No associations were observed for other factors in both sexes.

**Conclusions** Traditional CV risk factors are unrelated to migraine, except for smoking. While underlying mechanisms are not clarified yet, our study contributes to the hypothesis that migraine is associated with non-traditional CV risk factors, which may relate to microvascular dysfunction, as reflected by the slightly increased diastolic BP. These mechanisms may differ among sexes.

## P140 A prospective evaluation of neurological presentations to a National Neuro-Behçet Clinic in the UK- Migraine is the commonest neurological complaint in Behçet"s disease

### M. Ghadiri-Sani^1,2^, S. Broadhurst^1^, J. Nair^2^, R. Moots^2^

#### ^1^Walton Centre NHS foundation Trust, Neurology, Liverpool, United Kingdom; ^2^Aintree University Hospital, Rheumatology, Liverpool, United Kingdom

##### **Correspondence:** M. Ghadiri-Sani

**Introduction**

Behçet"s disease (BD) is a relapsing multisystem inflammatory condition. It manifests as recurrent oral and genital aphthous ulceration, skin lesions, ocular disease (uveitis), arthritis and neurological deficits.

Neuro-Behçet"s Disease (NBD) affects avout 10% of patients and can be associated with high morbidity and mortality and is classified into parenchymal (brainstem, multifocal, myelopathy, cerebral or optic neuropathy), and non-parenchymal (cerebral venous thrombosis, intracranial hypertension, acute meningeal syndromes) disease

**Objectives**

To evaluate patients seen in the NBC at the national Behçet"s centre in Liverpool, UK.

**Methods**

A prospective review of all patients in the NBC from December 2021 to June 2022.

**Results**

24 patients (10%, F: 19, M:5) were seen in the NBD clinic, of the total of 249 patients. 4 patients (2%) fulfilled the criteria for NBD, 3 of which had parenchymal disease, 1 with inflammatory cerebellar lesions, 1 with an inflammatory CSF but normal imaging and one with optic neuropathy. One patient had non-parenchymal disease with cerebral sinus venous thrombosis (CSVT).

20 (83%) patients presented with migraine, 15 (75%) of whom had associated medication overuse headaches (MOH). Only 13 patients had received prior preventatives.

18 (75%) patients were already on immunosuppression initiated and 1 patient was started on immunosuppression following the diagnosis of CSVT.

**Conclusion**

Based on our findings, NBD remains a rare complication of BD (2%) with majority of patients seen in the NBC presenting with migraine (83%); only 65% (13) of whom had received on average 2 prior preventative treatment (Propranolol, 5 and Pregabalin, 6). MOH is a common co-morbid condition. Given the prevalence of migraine in the general population, further studies would be required to ascertain the correlation to BD.

Other complications of NBD were much rarer but associated with significant morbidity.

## P141 Safety and tolerability of atogepant: a post hoc analysis of pooled data from four clinical trials

### P. Rizzoli^1^, M. Marmura^2^, J. Robblee^3^, J. McVige^4^, S. Sacco^5^, R. De Abreu Ferreira^6^, L. Rekeda^7^, J. Ma^7^, B. Dabruzzo^7^, M. Ashina^8^

#### ^1^Brigham and Women’s Hospital, Boston, MA, United States; ^2^Thomas Jefferson University, Philadelphia, PA, United States; ^3^Barrow Neurological Institute, Phoenix, AZ, United States; ^4^DENT Neurologic Institute, Amherst, NY, United States; ^5^Carolinas Headache Clinic, Matthews, NC, United States; ^6^AbbVie, Chicago, IL, United States; ^7^AbbVie, Madison, NJ, United States; ^8^University of Copenhagen, Rigshospitalet, Neurology, Copenhagen, Denmark

##### **Correspondence:** P. Rizzoli

**Objective:** To characterize the safety and tolerability profile of atogepant for the preventive treatment of migraine, using pooled data across 4 clinical trials.

**Methods:** Data were pooled from 2 randomized double-blind, placebo-controlled (RPC) trials (a phase 2b/3 trial, and a phase 3 trial [ADVANCE]), and 2 open-label, long-term safety (LTS) trials of atogepant in participants with episodic migraine (EM). The phase 2b/3 trial evaluated atogepant 10, 30, and 60 mg once daily and 30 and 60 mg twice daily vs placebo. The ADVANCE trial evaluated the efficacy and safety of atogepant 10, 30, and 60 mg once daily vs placebo. Participants who completed the phase 2b/3 trial and the ADVANCE trial were eligible to participate in the 52-week and 40-week LTS trials, respectively. Both LTS trials assessed the safety and tolerability of atogepant 60 mg once daily. Here, we focus on the US-approved atogepant dosing of 10, 30, and 60 mg once daily.

**Results:** A total of 1550 participants from RPC trials and 1424 participants from LTS trials were pooled for this analysis. Of the 1142 participants who received atogepant once daily in the RPC trials, 643 (56.3%) experienced ≥1 treatment-emergent adverse event (TEAE) vs 218 (53.4%) in the pooled placebo group, and serious TEAEs were reported in 7 (0.6%) participants who received atogepant vs 4 (1.0%) in the pooled placebo group (**Table**). The most commonly reported TEAEs (>5% in either arm) in the RPC trials (atogepant vs placebo) were nausea (6.6% vs 3.2%), constipation (6.1% vs 1.2%), and upper respiratory tract infection (5.3% vs 6.1%). Of the 1228 participants who received atogepant in the LTS trials, 792 (64.5%) experienced ≥1 TEAE, and 47 (3.8%) reported serious TEAEs. The most commonly reported TEAEs (>5%) among those who received atogepant in the LTS trials were similar to those reported in the RPC trials.

**Conclusions:** The daily administration of atogepant in adults with EM up to 52 weeks was safe and generally well tolerated.

**Table 1 (abstract P141).**
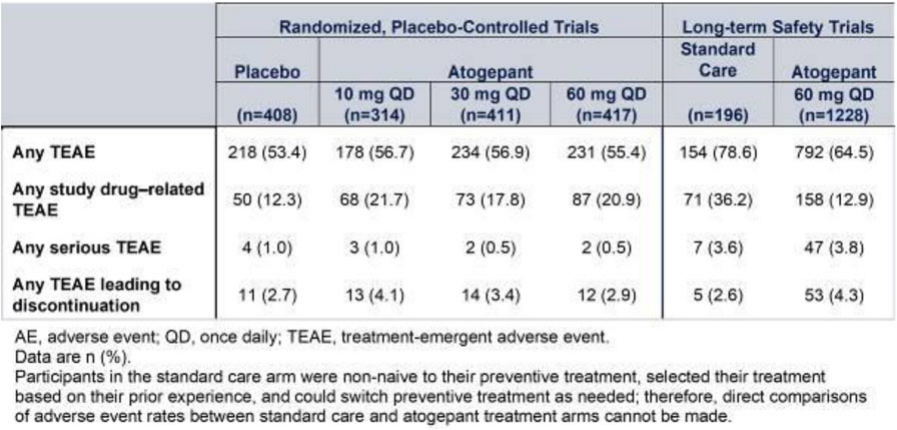
Summary of Treatment-Emergent Adverse Events for the Once-Daily Dose Groups

## P142 Effect of atogepant on Migraine-Specific Quality of Life Questionnaire and Headache Impact Test-6 in a 12-week, double-blind, randomized, phase 3 (PROGRESS) trial for preventive treatment of chronic migraine (CM)

### R. Lipton^1^, P. Pozo-Rosich^2^, D. Dodick^3^, S. Christie^4^, J. Ailani^5^, K. Nagy^6^, J. Stokes^7^, H. Guo^7^, P. Gandhi^7^

#### ^1^Albert Einstein College of Medicine, Bronx, NY, United States; ^2^Vall d’Hebron University Hospital and Autonomous University of Barcelona, Barcelona, Spain; ^3^Mayo Clinic, Neurology, Scottsdale, AZ, United States; ^4^University of Ottawa, Ottawa, Canada; ^5^MedStar Georgetown University Hospital, Washington, DC, United States; ^6^AbbVie, Budapest, Hungary; ^7^AbbVie, Madison, NJ, United States

##### **Correspondence:** R. Lipton

**Objective:** To evaluate impact of atogepant (ATO) on key secondary and exploratory patient-reported outcomes (PROs) for measures of functioning and headache-related impact among individuals with CM.

**Methods:** Phase 3, multicenter, randomized, double-blind, placebo (PBO)-controlled trial. Participants with ≥1-year history of CM, ≥15 headache d/mo in the past 3 months, and ≥15 headache days (with ≥8 days qualifying as migraine days) during the 28-day screening period were randomized to receive ATO 30mg twice daily (BID), ATO 60mg once daily (QD), or PBO during the 12-week treatment period. PROs included the Migraine-Specific Quality of Life Questionnaire v2.1 (MSQ) and Headache Impact Test-6 (HIT-6). Change from baseline in MSQ Role Function-Restrictive (RFR) domain and HIT-6 scores at week 12 were key secondary endpoints in Europe and Canada (MSQ RFR was also a key secondary endpoint in the United States). A graphical approach with weighted Bonferroni test procedure was used to control the overall type I error rate at the two-sided α=.05 level for key secondary endpoints. For exploratory endpoints, nominal *P* values were provided without adjusting for multiplicity.

**Results:** Of 778 participants randomized, 773 received study drug (mean age: 42.1y; 87.6% female), and 755 were included in the modified intent-to-treat population (ATO 30mg BID, n=253; ATO 60mg QD, n=256; PBO, n=246). At all assessed timepoints, increases from baseline (improvements) in all MSQ domain scores were significantly greater in both ATO doses vs PBO (*P*<.01) (**Figure**). Both doses demonstrated significant improvement in HIT-6 scores vs PBO at all assessed time points (*P*<.001). Significantly greater proportions of ATO- vs PBO-treated participants were HIT-6 responders (reduction ≥5 points) at all time points and doses (nominal *P*<.001).

**Conclusions:** ATO demonstrated statistically significant improvements in PRO measures of functional ability and impact of headache.

**Fig. 1 (abstract P142).**
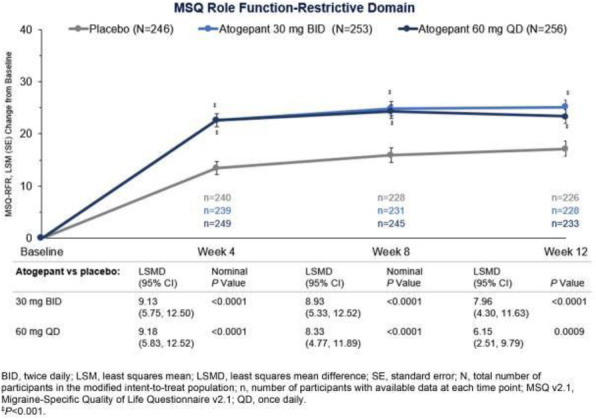
Change From Baseline in MSQ Role Function-Restrictive Domain at Weeks 4, 8, and 12

## P143 Effect of atogepant on the Activity Impairment in Migraine–Diary and Work Productivity and Activity Impairment Questionnaire in a 12-week, double-blind, randomized, phase 3 (PROGRESS) trial for preventive treatment of chronic migraine (CM)

### R. Lipton^1^, P. Pozo-Rosich^2^, D. Dodick^3^, D. I. Friedman^4^, J. Ailani^5^, J. Smith^6^, J. Stokes^7^, B. Schwefel^8^, P. Gandhi^7^

#### ^1^Albert Einstein College of Medicine, Bronx, NY, United States; ^2^Vall d’Hebron University Hospital and Autonomous University of Barcelona, Barcelona, Spain; ^3^Mayo Clinic, Neurology, Scottsdale, AZ, United States; ^4^University of Texas Southwestern Medical Center, Departments of Neurology and Ophthalmology, Dallas, TX, United States; ^5^MedStar Georgetown University Hospital, Washington, DC, United States; ^6^AbbVie, Irvine, CA, United States; ^7^AbbVie, Madison, NJ, United States; ^8^AbbVie, North Chicago, IL, United States

##### **Correspondence:** R. Lipton

**Objective:** To evaluate impact of atogepant (ATO) on key secondary and exploratory patient-reported outcomes (PROs) for measures of daily functioning and work productivity among individuals with CM.

**Methods:** A phase 3, multicenter, randomized, double-blind, placebo (PBO)-controlled, parallel-group trial. Participants (≥1-year history of CM, ≥15 headache d/mo in the past 3 months, and ≥15 headache days [≥8 migraine days] during the 28-day screening period) were randomized to receive ATO 30mg twice daily (BID), ATO 60mg once daily (QD), or PBO for 12 weeks. PROs included the Activity Impairment in Migraine–Diary (AIM-D) and Work Productivity and Activity Impairment Questionnaire (WPAI): Migraine. Improvements across the 12-week period in AIM-D Performance of Daily Activities (PDA) and Physical Impairment (PI) domains were key secondary endpoints in all regions except Europe and Canada. A graphical approach with weighted Bonferroni test procedure was used to control the overall type I error rate at the 2-sided α=.05 level for key secondary endpoints. For exploratory endpoints, nominal *P* values were provided without adjusting for multiplicity.

**Results:** Of 773 participants (mean age: 42.1y; 87.6% female) from the safety population, 755 were included in the modified intent-to-treat population (ATO 30mg BID, n=253; ATO 60mg QD, n=256; PBO, n=246). Both ATO groups had statistically significant improvements from baseline across the 12-week treatment period in AIM-D PDA (**Figure**) and PI domain scores (*P*<.01), and nominally significant improvements at weeks 1-4, weeks 5-8, and weeks 9-12 (only for 30mg BID) for both AIM-D domains vs placebo. Nominally significant improvements were seen in presenteeism, overall work productivity loss (**Figure**), and activity impairment at all time points, and in absenteeism at weeks 4 and 12, for both doses vs PBO (*P*<.05).

**Conclusions:** ATO demonstrated statistically significant improvements in PRO measures of daily functioning and work productivity.

**Fig. 1 (abstract P143).**
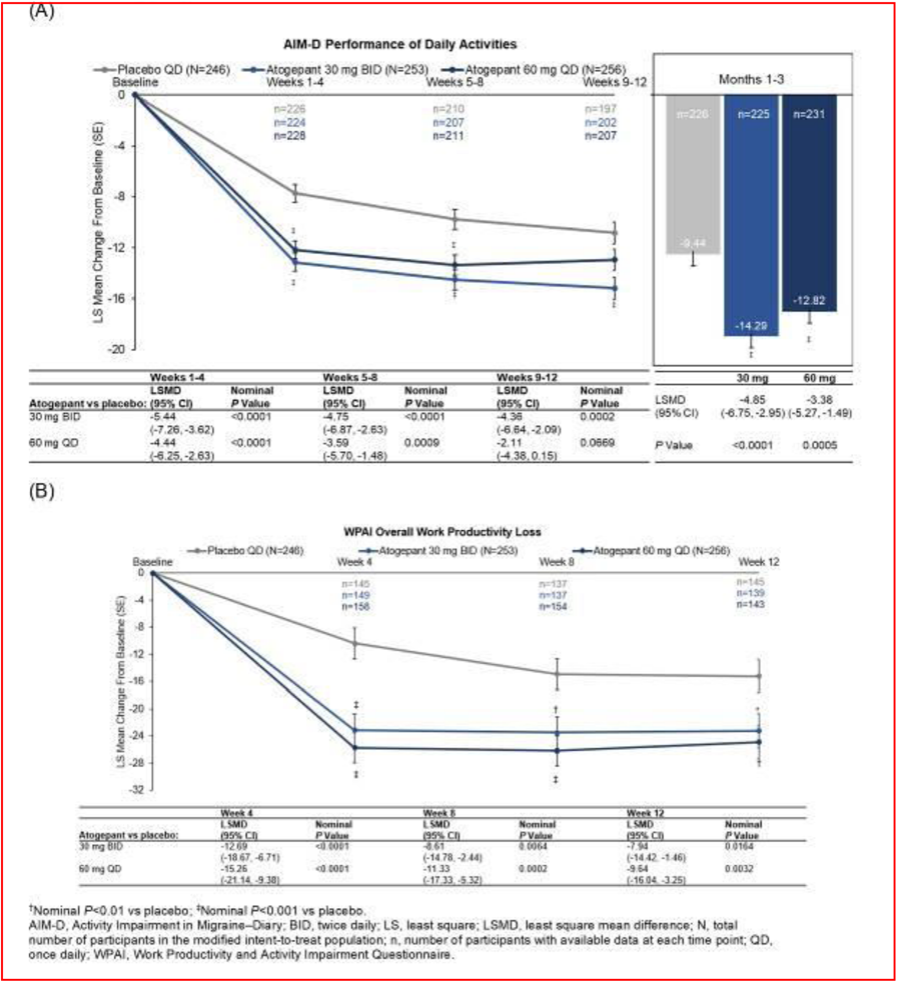
Change From Baseline in (A) AIM-D Performance of Daily Activities at Weeks 1-4, 5-8, and 9-12 and Across 12 Weeks and (B) WPAI Overall Work Productivity Loss at Weeks 1-4, 5-8, and 9-12

## P144 Erenumab as a first line preventive treatment for episodic migraine in Spain: a cost-effectiveness analysis.

### P. Irimia^1^, S. Sánchez^2^, C. Crespo^2^, M. Martínez^3^, P. Pozo-Rosich^4,5^

#### ^1^Clínica Universidad de Navarra, Neurology, Pamplona, Spain; ^2^Axentiva Solutions S.L., Barcelona, Spain; ^3^Novartis, Barcelona, Spain; ^4^Vall d'Hebron University Hospital, Neurology, Barcelona, Spain; ^5^Autonomous University of Barcelona, Research Group, VHIR, Barcelona, Spain

##### **Correspondence:** P. Irimia

**OBJECTIVE:** To perform a cost-effectiveness analysis of erenumab 140 mg vs topiramate for the prophylaxis of episodic migraine in preventive treatment naïve patients in Spain.

**METHODS:** We built a cost-effectiveness analysis using a Markov model with 12-week cycles based on responders from the societal perspective. A responder was defined as having a minimum 50% reduction in the number of monthly migraine days (MMDs). The hypothetical cohort were patients with episodic migraine (4+ MMD), preventive treatment naive (80.5% women and mean age of 41 years). We estimated quality-adjusted life years (QALY) and MMD over a 5-year time horizon. Incremental cost-effectiveness ratios based on QALYs and MMD avoided were performed. Resource use and costs (2022) were obtained from official data sources and were validated by an expert panel. Sensitivity analysis was performed to validate the robustness.

**RESULTS:**

At 5 years, QALYs were 3.23 for topiramate and 3.35 for erenumab. Erenumab showed an incremental cost per patient of 2.986€ vs topiramate. Incremental cost per QALY gained with erenumab was 24.859€, below the Spanish efficiency threshold. Patients treated with erenumab improved mean MMDs over time, from 9.05 MMDs at baseline to 6.03 MMDs at 5 years, while topiramate patients improved to 7.71. Given the total reduction of migraine days with erenumab, [MMD1] the cost per MMDs avoided with erenumab was 33€.

**CONCLUSION:** Erenumab (Aimovig®) is a cost-effective alternative vs topiramate for episodic migraine from the societal perspective. Our findings suggest that erenumab (Aimovig®) is cost-effective in preventive treatment naïve patients.

## P145 Impact of migraine attack accompanying symptoms in treatment response to anti-CGRP monoclonal antibodies treatment and their evolution after 6 months

### A. Alpuente, A. Torre-Suñe, E. Caronna, M. Torres-Ferrús, P. Pozo-Rosich

#### Vall d'Hebron University Hospital, Neurology, Barcelona, Spain

##### **Correspondence:** A. Alpuente

**Question:** Studies with anti-CGRP monoclonal antibodies (mAbs) poorly investigated their impact on migraine accompanying symptoms. We aimed to evaluate whether accompanying symptoms at baseline influenced mAb treatment response and their evolution after 6 months.

**Methods:** Prospective study. Patients with migraine diagnosis seen in the Headache Clinic and treated with erenumab, galcanezumab or fremanezumab were recruited. They completed a daily electronic diary providing data on migraine frequency and accompanying symptoms in every attack (photophobia, phonophobia, nausea, dizziness and aura). Patients were classified as responders or non-responders based on 50% or greater reduction in HDM at 6 months (≥50% RR). Accompanying symptoms ratios based on headache days per month (HDM) were assessed per patient at baseline and after 6 months. Comparisons for baseline characteristics, for accompanying symptoms ratios between responders and non-responders and for symptomatology between baseline and 6 months were performed. A generalized Poisson mixed-effects regression model was estimated to assess the potential effect of accompanying symptoms at baseline on HDM evolution.

**Results:** 106 patients were included, 48/106 (45%) had ≥50% RR. No statistically significant differences in accompanying symptoms at baseline were found between groups. A significant reduction in HDM in both groups after 6 months was found (-11.0 days/month responders,-3.2 days/month non-responders;p<0.001). Significant decreases in photophobia (-16.0%,p=0.008) and phonophobia ratios (-10.6%,p=0.037) were additionally found for responders. Accompanying symptoms at baseline did not reach a significant effect on HDM reduction over time.

**Conclusions:** Photophobia and phonophobia ratios based on HDM were significantly reduced in the responder group, indicating that the presence of these accompanying symptoms improve due to mAbs treatment and decrease to a higher rate than HDM does for responders after 6 months.

**Fig. 1 (abstract P145).**
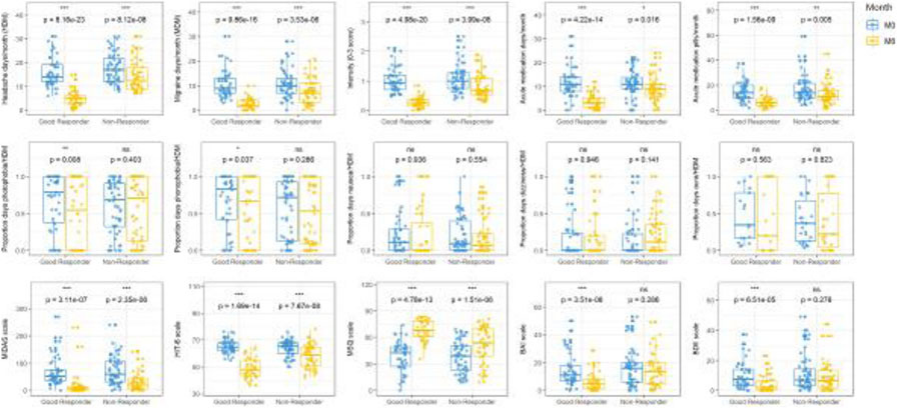
See text for description

**Fig. 2 (abstract P145).**
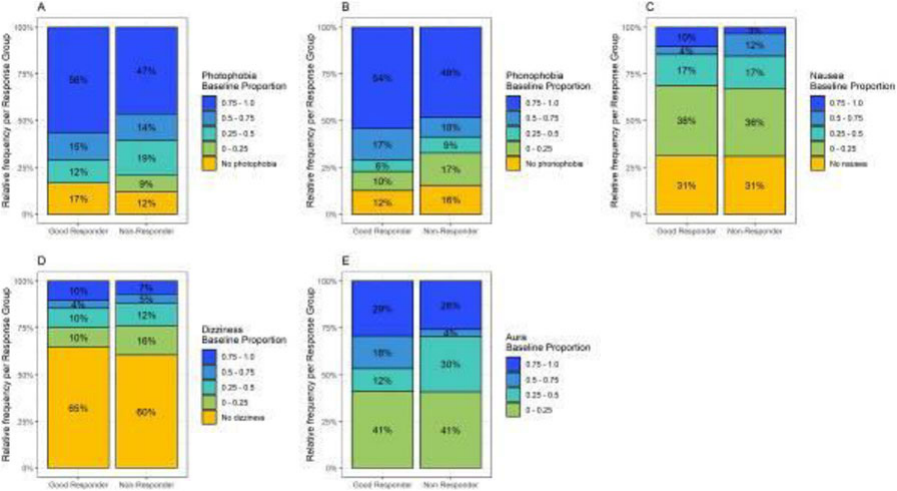
See text for description

## P146 Mapping Migraine Minds: A cross-sectional survey to compare the difference in the level of treatment expectations and satisfaction for migraine among Indian male & female patients

### S. Singh^1^, R. L. Narasimhan^2^, A. Gupta^3^, J. Sharma^4^, U. Sundar^5^, S. Thakur^6^, A. Thorat^6^

#### ^1^Artemis Hospital, Neurology, Gurgaon, India; ^2^Madras Medical College, Neurology, Chennai, India; ^3^Army Hospital, R&R New Delhi, New Delhi, India; ^4^Base Hospital, Neurology, New Delhi, India; ^5^Sion Hospital, Neurology, Mumbai, India; ^6^Novartis, Medical, Mumbai, India

##### **Correspondence:** S. Thakur

**Objectives:** To assess the difference in the level of treatment expectations and satisfaction for migraine among Indian male(M) and female(F) patients.

**Methods:** A survey was conducted from 20th April 2022 – 21st June 2022 in 300 adult male and female (1:1) migraine patients. Survey questionnaire was validated by a steering committee of 10 Indian neurologists. Data was collected by using telephonic and face to face interview mode.

**Results:** On an average, female migraine patients had higher expectations from migraine treatment compared with males [60%(F); 51%(M)]. Higher proportion of females wanted aggressive therapy for rapid relief [68%(F); 52%(M)]. Higher proportion of females expected symptom relief [53%(F); 41%(M)] & more females did not want their migraine to worsen [48%(F); 36%(M)]. Overall average treatment satisfaction level was lower in females than that in males for both acute [73%(F); 77%(M)] & preventive therapies [81%(F); 87%(M)].

**Conclusion:** This study has demonstrated that there is a difference in the level of treatment expectations & satisfaction with both acute & preventive therapies with female patients demanding more from their current migraine therapies. An individualized approach towards migraine care for both male & female patients comprising of realistic expectations from therapy, lifestyle modification, trigger management & early use of targeted advanced pharmacotherapy would improve clinical outcomes. A focused attention towards female migraine patients in India is warranted where females are also the caregivers, & their migraine could impact their families too

**Key words**: Migraine; Treatment satisfaction; Treatment expectation; Genders

## P147 Migraine, chronic neck pain and endurance muscle cervical test - a controlled study

### A. Rodrigues^1^, M. Mendes Bragatto Scornavacca^1^, L. Lima Florencio^2^, L. Bigal^3^, M. Bigal^4^, D. Bevilaqua Grossi^1^

#### ^1^University of São Paulo, Health Sciences, Ribeirão Preto, Brazil; ^2^Universidad Rey Juan Carlos, Madrid, Spain; ^3^University of North Carolina, Chapel Hill Gillings School of Global Public Health, North Chicago, IL, United States; ^4^Ventus Therapeutics, Montreal, Canada

##### **Correspondence:** A. Rodrigues

**Objective:** To verify if the cervical pain observed in patients with migraine may occur due to cervical muscle dysfunction, the presence of pain during the cervical muscle endurance test or a combination of both. **Methods:** Sample consists of 100 women, stratified by diagnosis (migraine, cervical pain, both and none) and self-reported pain during the cervical muscle endurance test (with or without headache and / or cervical pain during the endurance test). The resistance test for cervical flexion and extension was evaluated and immediately after each resistance test, the participants were asked if they had neck and / or head pain during the test. Pain was classified according to the numerical pain rate scale (NPRS, 0-10). **Results:** As for the diagnosis, during the endurance test in flexion, migraine patients with cervical pain presented less endurance when compared to the control (p = 0.02). In the extension endurance test, the cervical pain groups with or without migraine, had a shorter sustaining time than the control group (p <0.01). As for the report of pain during the endurance test in flexion and extension, those who had headache sustained less time than those without headache during the test. Similar results were seen when comparing those with head and neck pain versus no pain during the test (p <0.05). **Conclusion**: The clinical diagnosis was not decisive for the performance of muscular endurance. Instead, the presence of headache associate or not neck pain during the test is what caused the endurance time to decrease.

## P148 Effects of Rimegepant 75 mg on Monthly Migraine Days: a 52-Week, Open-Label Extension Study

### J. Ailani^1^, D. Kudrow^2^, T. Smith^3^, R. Lipton^4^, A. C. Thiry^5^, C. M. Jensen^5^, L. Kamen^5^, V. Coric^5^, R. Croop^5^

#### ^1^Medstar Georgetown University Hospital, Washington, DC, United States; ^2^California Medical Clinic for Headache, Santa Monica, CA, United States; ^3^Study Metrix Research, Saint Peters, MO, United States; ^4^Albert Einstein College of Medicine, Bronx, NY, United States; ^5^Biohaven Pharmaceuticals, New Haven, CT, United States

##### **Correspondence:** C. M. Jensen

**Objective**Assess the effects of rimegepant 75 mg on monthly migraine days (MMDs) through 52 weeks of open-label treatment when dosed every other day (EOD) for preventive treatment plus as needed (PRN) for acute treatment on nonscheduled dosing days.**Methods**Open-label extension phase of a 12-week, randomized, double-blind, placebo-controlled study evaluating rimegepant 75 mg EOD for preventive treatment of migraine in adults aged ≥18 years with a history of 4-18 moderate-severe monthly migraine attacks. Subjects completing a 4-week observation period and 12 weeks of double-blind treatment could continue with open-label rimegepant 75 mg EOD for preventive treatment for 52 weeks. On nonscheduled dosing days, subjects could take rimegepant 75 mg up to once per day PRN for acute treatment. **Results**Of 741 subjects who received double-blind treatment, 603 (81.4% [rimegepant n=301, placebo n=302]) were treated in the open-label phase (mean age 42.6 years, 82.7% female, hx of 7.9 monthly mod-sev attacks). Mean (SD) number of rimegepant doses per month was 14.6 (2.45). The most common adverse events were upper respiratory tract infection (7.1%), nasopharyngitis (6.3%), and back pain (4.3%). Through 52 weeks of open-label rimegepant (Figure A), the frequency of MMDs consistently declined; mean (95% CI) changes from the observation period in MMDs were −5.1 (−5.49, −4.74) in Weeks 1-4 and −6.9 (−7.31, −6.56) in Weeks 49-52. The percentage of subjects with ≥50% reduction in moderate-severe MMDs from the observation period ranged from 63.6% (Weeks 1-4) to 80.9% (Weeks 49-52), ≥75% reductions ranged from 44.1% (Weeks 1-4) to 65.8% (Weeks 49-52), and 100% reductions ranged from 25.6% (Weeks 1-4) to 49.3% (Weeks 49-52; Figure B).**Conclusion**Scheduled EOD preventive treatment with rimegepant 75 mg plus PRN acute treatment on nonscheduled days consistently reduced MMDs over 52 weeks. More than 80% of subjects had ≥50% reduction in moderate-severe MMDs; ~50% had a 100% reduction by Week 52.

**Fig. 1 (abstract P148).**
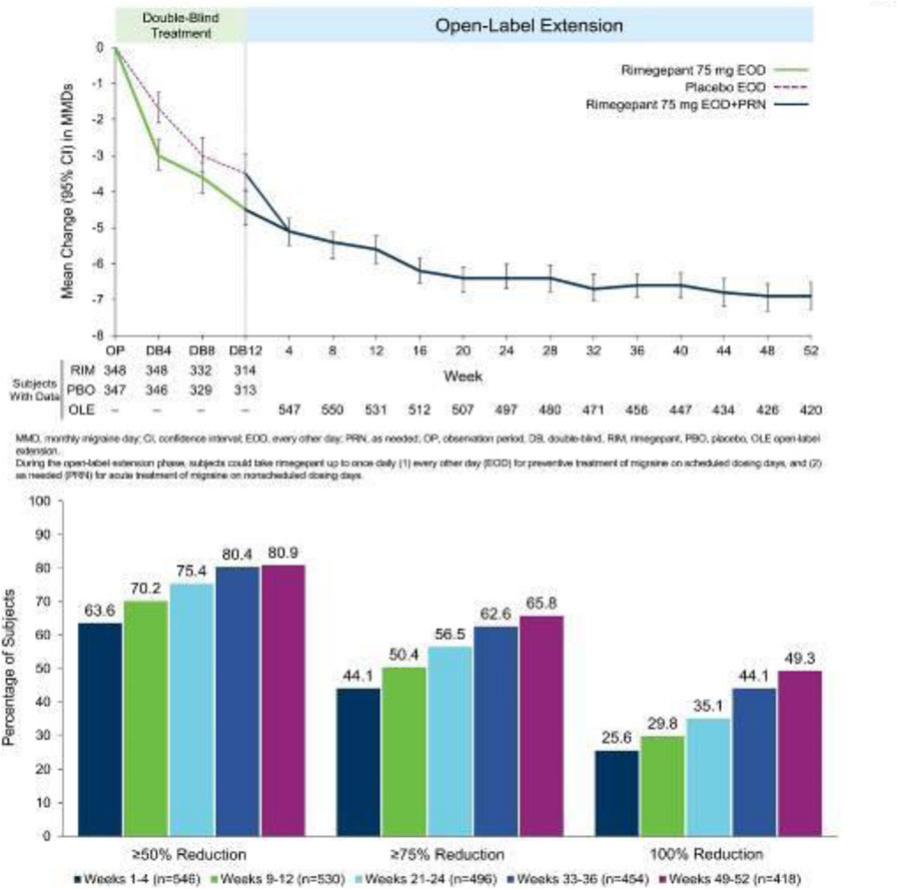
See text for description

## P149 Safety and Tolerability of Rimegepant Every Other Day for Preventive Treatment of Migraine Plus As-Needed for Acute Treatment of Migraine: Results from A 52-Week, Open-Label Extension Study

### R. Lipton^1^, D. Kudrow^2^, T. Smith^3^, J. Ailani^4^, L. Kamen^5^, A. C. Thiry^5^, C. M. Jensen^5^, V. Coric^5^, R. Croop^5^

#### ^1^Albert Einstein College of Medicine, Bronx, NY, United States; ^2^California Medical Clinic for Headache, Santa Monica, CA, United States; ^3^Study Metrix Research, Saint Peters, MO, United States; ^4^Medstar Georgetown University Hospital, Washington, DC, United States; ^5^Biohaven Pharmaceuticals, New Haven, CT, United States

##### **Correspondence:** C. M. Jensen

Objectives

Assess safety and tolerability of rimegepant 75 mg every other day (EOD) for the preventive treatment of migraine and as-needed (PRN) for acute treatment on nonscheduled dosing days.

Methods

This 1-year open-label extension phase of a 12-week, randomized, double-blind, placebo-controlled study (NCT03732638) of rimegepant for the preventive treatment of migraine included adults aged ≥18 years with a history of 4-18 moderate-severe monthly migraine attacks. Subjects completing 12 weeks of double-blind rimegepant 75 mg or placebo EOD could continue with rimegepant 75 mg EOD for 52 weeks. On nonscheduled dosing days, subjects could take rimegepant 75 mg up to once per day PRN. Safety assessments were adverse events (AEs) and clinical laboratory tests, including liver function tests. Subjects who took ≥1 dose of open-label rimegepant were analyzed. Months were 4-week intervals.

Results

Of 741 subjects who received double-blind treatment, 603 (81.4% [rimegepant n=301, placebo n=302]) were treated in the open-label extension (mean age 42.6 years, 82.7% female, hx of 7.9 monthly mod-sev attacks). The most common AEs (Figure) were upper respiratory tract infection (7.1%), nasopharyngitis (6.3%), and back pain (4.3%). The discontinuation rate due to AEs was 2.8%. Serious AEs (2.2%) were unrelated to rimegepant. Two deaths (0.3%), 1 due to aortic dissection related to Marfan syndrome and 1 due to sepsis, were also unrelated to rimegepant. Aminotransferases >3x the upper limit of normal (ULN) occurred in 3.4% of subjects; none had elevations in bilirubin >2x ULN. Mean (SD) number of rimegepant doses per month was 14.6 (2.45); 81.4% of subjects used ≤16 tablets per month.

Conclusion

One year of open-label rimegepant 75 mg EOD for preventive treatment of migraine plus PRN on nonscheduled dosing days for acute treatment up to once daily was safe and well tolerated with no liver safety concerns. Use of PRN treatment was limited, and >80% of subjects took ≤16 tablets per month.

**Fig. 1 (abstract P149).**
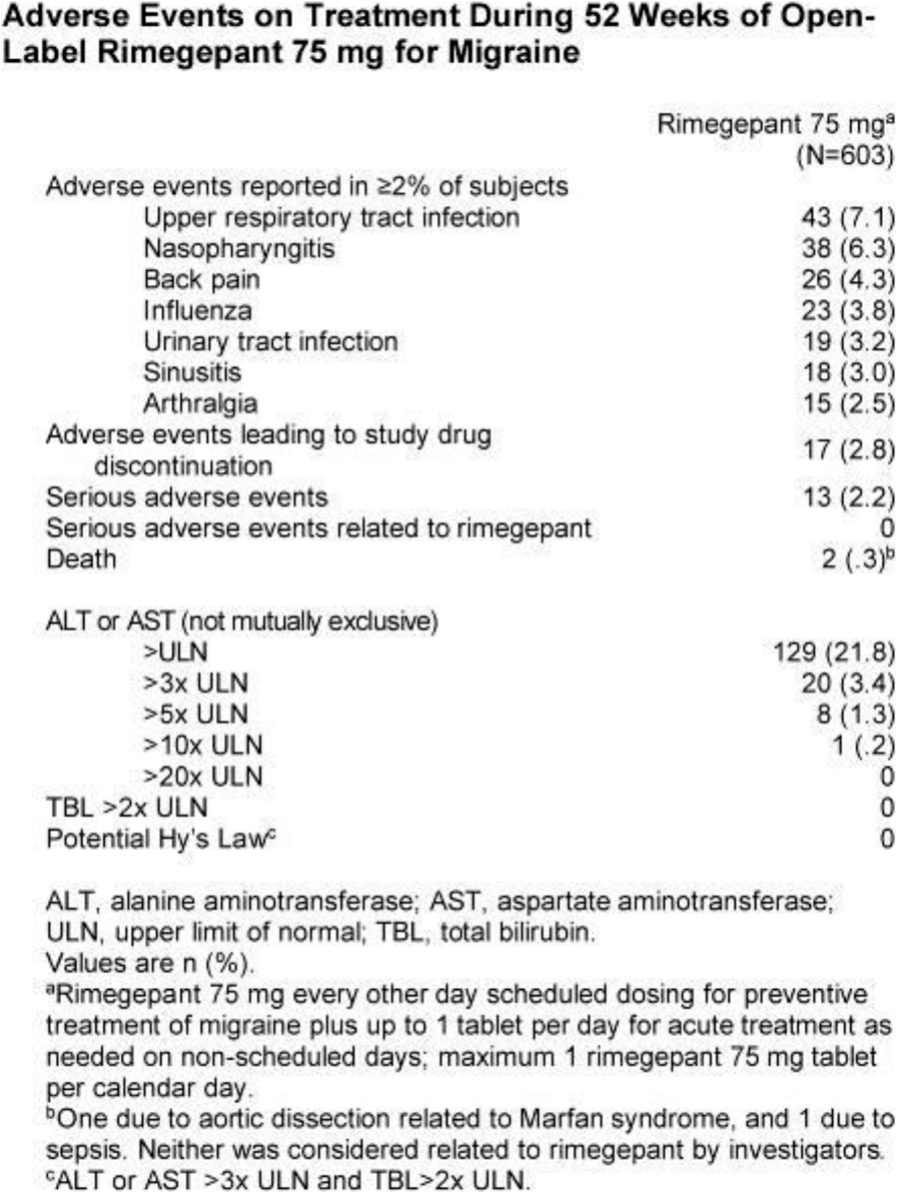
See text for description.

## P150 Onabotulinum toxinA for unremitting chronic migraine: assessment of muscle function and strength, efficacy and safety after 10 years of continuous treatment

### G. P. Boudreau

#### Clinique des Céphalées et de Recherches de Montréal, Headache clinic, Montreal, Canada

OBJECTIVES

Assess the impact of the injection paradigms on muscle function and strength, assess the efficacy and safety over 10 years of repeated treatments every 3 months, identify risk factors maintaining chronicity.

METHOD:

One hundred patients were injected with a dilution ratio of 1:1 with a sterile 0.9 % saline solution, 50 patients with a 100u vial in one, 1cc tuberculin syringe, and 50 patients with a 200u vial of onabotulinum toxinA in two, 1cc tuberculin syringe during 10 years.

RESULTS

The strength of the paracervical and first portion of the trapezius muscle was altered in 6% (100u) and 18% (155u) of subjects. The second portion of the trapezius muscle was altered in 2% (100u) and 10% (155u) of subjects. Muscle function of the paracervical and first portion of the trapezius muscles was altered in 34% (100u) and 28% (155) of subjects, for the second portion of the trapezius muscle 58% (100u) and 52% (155) of subjects. The efficacy of onabotulinum toxinA was constantly maintained during the 10 years of treatment. 72% (100u), and 74% (155u) of subjects had less than 7migraine days /month (77% improvement). Early onset of migraine, comorbid emotional burden and chronic neck pain, should be considered as risk factors for the unremitting condition.

CONCLUSION

Muscle strength, and function alteration did not have an impact on esthetics of the face and on normal daily muscle function in both cohorts. In both cohorts more than 70% of patients had more than 75% improvement in monthly migraine days. Depth of the toxin injection, diffusion and presence of adipose tissue (lean versus obese patients) may be responsible for muscle strength and function alteration.

## P151 Long-term Effectiveness and Safety of Erenumab in patients with Migraine: a Systematic Review and Single-Arm Meta-analysis

### F. Ferreira Bomtempo^1^, J. P. Mota Telles ^2^, G. Isadora Cenci ^3^, G. Borges Nager^4^, R. Bustamante Rocha ^5^

#### ^1^Faculdade Ciências Médicas de Minas Gerais (CMMG), School of Medicine, Belo Horizonte, Brazil; ^2^Hospital das Clínicas da Faculdade de Medicina da Universidade de São Paulo (HC-FMUSP), Department of Neurology, São Paulo, Brazil; ^3^Faculdade Meridional (IMED), School of Medicine, Passo Fundo, Brazil; ^4^Universidade Federal do Estado do Rio de Janeiro (UNIRIO), School of Medicine, Rio de Janeiro, Brazil; ^5^Universidade Federal do Amazonas (UFAM), School of Medicine, Manaus, Brazil

##### **Correspondence:** F. Ferreira Bomtempo

**Background:** Several studies on use of erenumab for migraine treatment have been published over the last years. This study aims to estimate the safety and effectiveness of erenumab on the long-term basis (established as ≥ 1 year of exposure).

**Methods:** PubMed, Embase and Cochrane were systematically searched randomized clinical trials (RCTs) phase extensions and real-world studies through June 2022. Risk of bias was assessed using the Newcastle-Ottawa Scale.

**Results:** 14 studies comprising 3,574 patients met the inclusion criteria. Total follow-up period ranged from 48 up to 268 weeks (i.e., 1 year to 5.6 years). The pooled estimate rates for all adverse events (AEs) were 63% (CI: 46-78% - see Figure 1A); for serious AEs, 3% (95% CI: 1-7% - see Figure 1B); and for AEs leading to discontinuation of erenumab, 3% (95% CI: 2-5% - see Figure 1C). AEs corresponded to the minority (15.8%) of all reasons to discontinuation from reported data. Reduction in monthly migraine days (MMDs) was -6.98 (95% CI: -8,90; -5.05 - see Figure 2A) and in migraine specific medication days (MSMDs), -6.09 (95% CI: -9.43; -2.75 - see Figure 2B). More than half (57%; 95% CI: 51-63% - see Figure 3A) and around one-third (35%; 95% CI: 28-42% - see Figure 3B) of patients presented reductions of ≥ 50% and ≥ 75% in MMDs, respectively. Headache Impact Test-6 (HIT-6) score was decreased in -9.68 points (95% CI: -12.03; -7.34 - see Figure 2C).

**Conclusions:** Cumulative analysis of data revealed a consistent favorable safety profile and a sustained effectiveness of erenumab with long-term exposure in the treatment of migraine.

**Figure 3 –** Percentage reduction in MMDs

**3 (A)** – ≥50%

**3 (B) -** ≥75%

**Fig. 1 (abstract P151).**
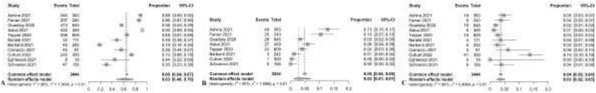
Incidence of AEs. **1(A)** – All AEs. **1(B) -** Serious AEs. **1(C)** - AEs leading to discontinuation

**Fig. 2 (abstract P151).**
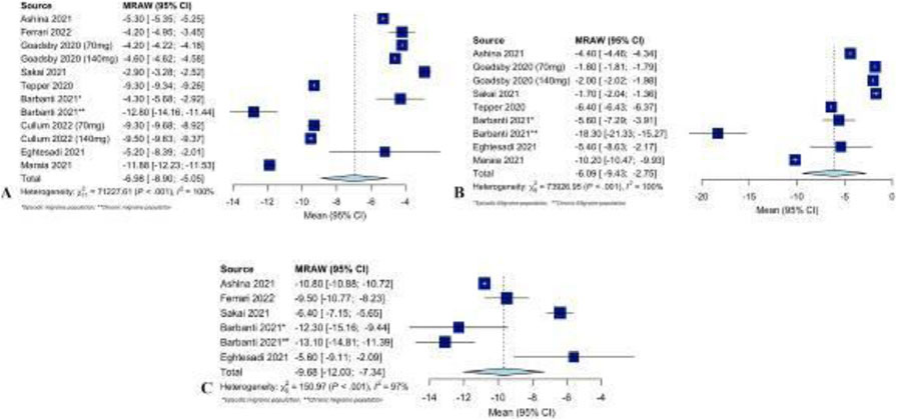
Change from baseline. **2(A)** – in MMDs. **2 (B) -** in MSMDs. **2 (C)** - in HIT-6

## P152 Prospective evaluation of migraine premonitory symptoms during the 30 days period

### K. Skorobogatykh, J. Azimova, D. Korobkova, N. Vashchenko, A. Uzhakhov, S. Kornienko, E. Mamkhegov

#### University Headache Clinic, Moscow, Russian Federation

##### **Correspondence:** K. Skorobogatykh

The objective was to evaluate the symptoms of the migraine premonitory phase prospectively.

Methods: We used Migrebot headache diary database to select subjects with migraine features and headache frequency 3-8 days per month. Selected subjects proceeded to complete a specially designed version of the diary (ProdromaBot) to assess the characteristics of migraine attacks and interictal symptoms for at least 30 days. Participants completed 3 time points (TP) daily (9am,15pm,21pm). At each TP, participants answered 51 questions about potential triggers, overall wellbeing, premonitory symptoms, and presence of a headache and its characteristics.

Results: 98 subjects entered the study, 71 subjects completed at least 30 days period with at least 80% compliance. 59 subjects visited the clinic to confirm migraine. Patients completed not all TPs, so we selected only TP with new headache episodes (N=682) which were preceded or followed by the fully completed TP. Thus, we had 581 premonitory TPs and 640 postdrome TPs for further analysis. We analyzed the frequency of premonitory symptoms depending on the headache attack characteristics.

Phonophobia was the symptom which has the greatest number of premonitory symptoms (excess of light p<0,04, light sensitivity p<0,0001, excess of noise p<0,001, sound sensitivity p<0,0001, odor sensitivity p<0,047, hunger p<0,003, dehydration p<0,0001, feeling anxious p<0,0001 or depressed p<0,004,yawing p<0,0001, eye strain p<0,014,scalp allodynia p<0,001,unilateral lacrimation or nasal congestion p<0,007,frequent urination p<0,005). Allodynia was the most frequent premonitory symptom followed by the light sensitivity, feeling anxious, dehydration, unilateral lacrimation or nasal congestion and frequent urination.

Conclusions: This is the first study in which migraine premonitory symptoms were analyzed prospectively for at least 30 days 3 times daily. Premonitory symptoms vary significantly depending on the migraine attack characteristics.

**Fig. 1 (abstract P152).**
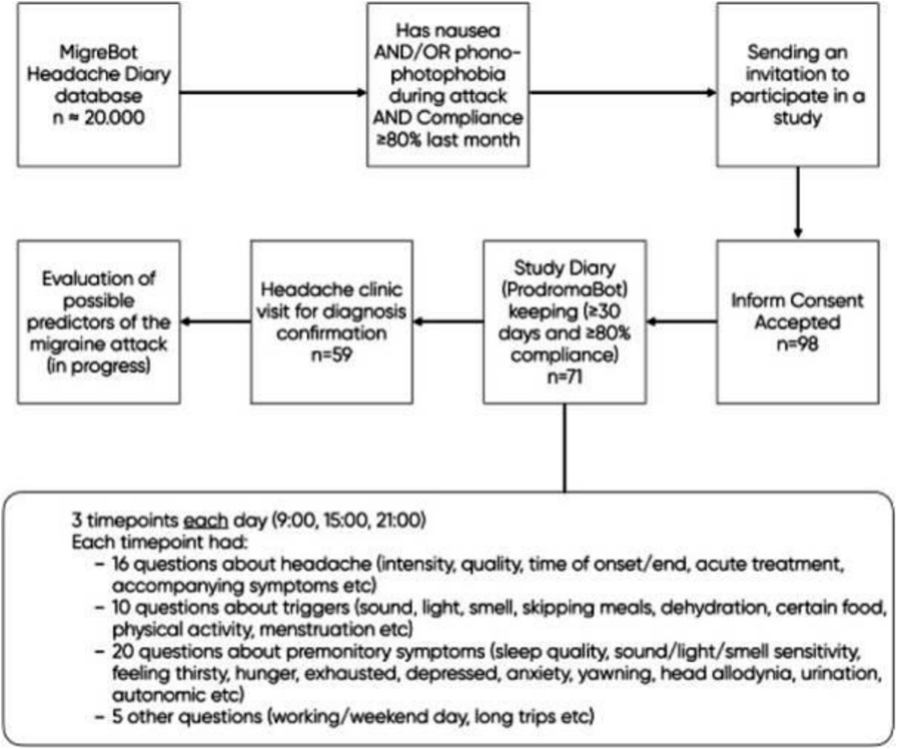
See text for description.

**Fig. 2 (abstract P152).**
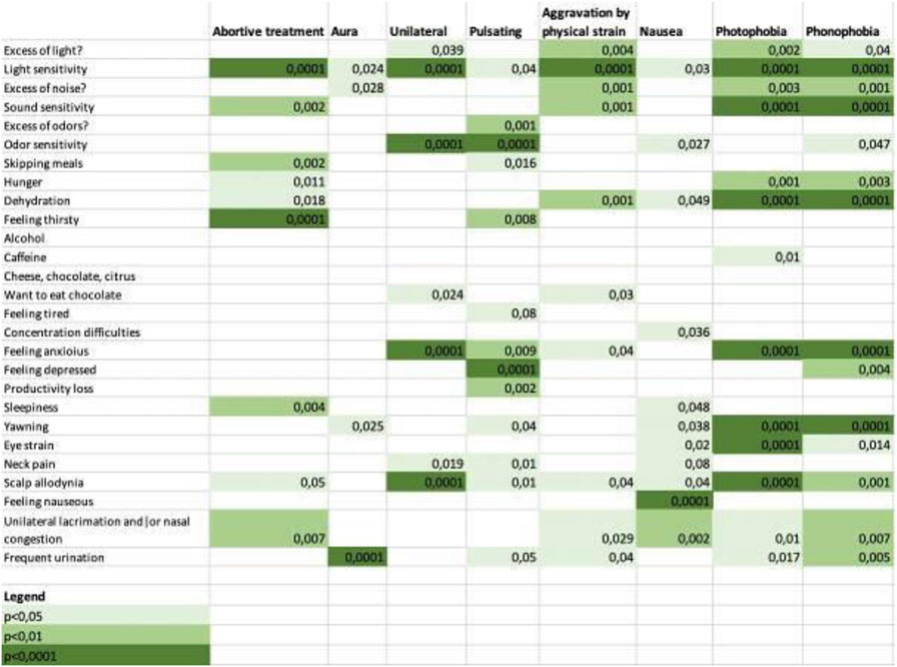
See text for description.

## P153 Prevalence of sinonasal symptoms in migraine without aura (results from *Migraine in Poland -* a nationwide cross-sectional survey)

### M. Straburzynski^1^, M. Waliszewska-Prosół^2^, S. Budrewicz^2^, E. K. Czapińska-Ciepiela^3^, M. Nowaczewska^4^, A. Gryglas-Dworak^5^, R. B. Lipton^6^

#### ^1^University of Warmia and Mazury, Department of Family Medicine and Infectious Diseases, Olsztyn, Poland; ^2^Wroclaw Medical University, Department of Neurology, Wrocław, Poland; ^3^Epilepsy and Migraine Treatment Centre, Kraków, Poland; ^4^Nicolaus Copernicus University in Toruń, Ludwik, Rydygier Collegium Medicum in Bydgoszcz, Department of Otolaryngology, Head and Neck Surgery, and Laryngological Oncology, Bydgoszcz, Poland; ^5^Headache Center, Wroclaw, Poland; ^6^Albert Einstein College of Medicine, Department of Neurology, Bronx, New York, United States

##### **Correspondence:** M. Straburzynski

Question: Assessing prevalence of sinonasal symptoms in migraine.

Methods: The *Migraine in Poland* study is a nation-wide cross-sectional online survey, conducted from August 2021 to June 2022. Survey protocol included questions assessing diagnostic criteria for migraine without aura (MwoA) according to International Classification of Headache Disorders-3, rhinosinusitis (European Position on Rhinosinusitis and Nasal Polyps 2020 – EPOS2020) and sinonasal/cranial autonomic symptoms (CAS) in relation to headache attacks.

Results: Among 1679 subjects meeting criteria for MwoA 602 (35.85%) of participants confirmed having at least one episode of self-described "sinus headache" in the last year (*n*=520 - 30.97% in the 3 months before the study). At least one nasal symptom was accompanying headache in 1004 (59.8%) respondents. During headache attacks, 315 (18.76%) subjects met symptomatic criteria for rhinosinusitis diagnosis according to EPOS2020. These symptoms were accompanied by other (non-nasal) CAS in *n*=251 (42.40%). Osmophobia was reported by 66.29% MwoA subjects. Hyposmia was present during headache attacks in 10.84%, with the majority of these respondents reporting co-existent osmophobia.

Conclusions: Sinonasal symptoms commonly occur in MwoA subjects, with 1/5 having symptoms indicating rhinosinusitis and many more reporting at least one rhinologic symptom during headache. Osmophobia and hyposmia are not mutually exclusive in MwoA, which limits their value in differentiating between rhinosinusitis and migraine. A comprehensive, multidisciplinary workup still remains the baseline in discerning between migraine and rhinosinusits.

## P154 Evaluation of a mandatory drug holiday during treatment with monoclonal antibodies targeting the cgrp pathway: a patient survey

### J. Versijpt^1^, E. Boon^2^, S. L. Sava^3^, F. Debruyne^4^, C. Van Humbeeck^5^, K. Delmotte^6^, J. Schoenen^7^

#### ^1^UZ Brussel, Neurology, Brussel, Belgium; ^2^Neurologiecentrum Vlierbeek, Kessel-Lo, Belgium; ^3^Clinique des Céphalées du Valdor, Liège, Belgium; ^4^GZA Sint-Augustinus , Antwerpen, Germany; ^5^Private practice, Herent, Belgium; ^6^Jessa Hospital, Neurology, Hasselt, Belgium; ^7^Citadelle Hospital, Headache Research Unit, Liège, Belgium

##### **Correspondence:** J. Versijpt

**Question**: how do patients evaluate a mandatory drug holiday during treatment with monoclonal antibodies targeting the CGRP pathway?

**Methods**: patients under treatment with monoclonal antibodies targeting the CGRP pathway were given a survey on how they evaluated this mandatory yearly drug holiday of 2 (erenumab) to 3 months (fremanezumab or galcanezumab).

**Results**: 79 adult patients either under treatment with erenumab, fremanezumab or galcanezumab were included. 75% deteriorated subjectively during the drug holiday. For 10% of the patients, this deterioration even required a quicker re-introduction of their preventive treatment. 22% had a stable disease or even a further improvement. 13% of the patients prolonged injection intervals in order to shorten the drug holiday. As for the timing of the deterioration, 47% had a worsening of their migraine already during the first month, while for 10% this only happened during the 2nd or 3rd month. For 11% of the patients the deterioration did only take place after 3 months, therefore the re-introduction of their preventive treatment could be postponed.

For 81% of the patients the mandatory drug holiday led to anxiety, of which 44% rated this anxiety as at least 'a lot'. On the other hand more than half of the patients (54%) found this drug holiday useful in order to assess the need for the continuation of their treatment, of which 23% even rated this as 'very useful'.

**Conclusions**: although a mandatory drug holiday is feasible and is considered useful for the majority of patients, it leads to both a swift aggravation of their migraine and substantial anxiety. A rigid and mandatory yearly drug holiday of 2-3 months seems not feasible for all migraine patients under treatment with a monoclonal antibody targeting the CGRP pathway.

## P155 Use of CGRP monoclonal antibodies and patient-reported improvement: Results from the OVERCOME (EU) study

### S. Evers^1^, G. Dell Agnello^2^, T. Panni^3^, D. Novick^4^, J. Pascual^5^, S. Gonderten^6^

#### ^1^University of Münster, Münster, Germany; ^2^Eli Lilly Italia SpA, Sesto Fiorentino, Italy; ^3^Eli Lilly Deutschland GmbH, Bad Homburg, Germany; ^4^Eli Lilly and Company Ltd., Bracknell, United Kingdom; ^5^Hospital Universitario Marqués de Valdecilla and University of Cantabria, Santander, Spain; ^6^Eli Lilly and Company, Dubai, United Arab Emirates

##### **Correspondence:** S. Evers and S. Gonderten

**Question**

Real-world data are limited for people who use a calcitonin gene-related peptide (CGRP) monoclonal antibody (mAb) for preventive treatment of migraine. We assessed respondent characteristics and patient-reported improvement among current CGRP mAb users in the OVERCOME (EU) study.

**Methods**

Data were obtained from a cross-sectional web-based survey (2020-2021). Adult respondents fulfilled International Classification of Headache Disorders (ICHD-3) criteria for migraine or had a self-reported physician diagnosis. This analysis assessed clinical and demographic characteristics in those who had ever used erenumab, fremanezumab or galcanezumab. Moreover, current users of a single mAb completed the Patient Global Impression of Improvement (PGI-I) to assess improvement in current migraine condition. Analyses were descriptive.

**Results**

Of 20,756 respondents, 2167 (10.4%) reported ever using a CGRP mAb. Among these, the mean (standard deviation [SD]) age was 32.9 (10.4) years, 38.8% were female, and the mean (SD) headache days/month (HD/m) was 3.4 (4.4). More than 1/3 of respondents (34.1%) had severe migraine-related disability (MIDAS score >=21), 30.8% moderate (11-20), 21.8% mild (6-10), and 13.3% little to none (<=5). The vast majority had used at least 1 additional traditional preventive medication. A total of 940 respondents (43.4%) had used a single CGRP mAb within the past 3 months. Among them, most respondents (77.3%) reported their migraine condition as "better" based on the PGI-I since starting the CGRP mAb. This was consistent across HD/m categories.

**Conclusions**

Most respondents taking a CGRP mAb for the preventive treatment of migraine reported their migraine as better since starting the medication.

## P156 Total tau concentrations are increased in blood serum of migraine patients: A cross-sectional case-control study

### L. H. Overeem^1,2^, B. Raffaelli^3,2^, R. Fleischmann^4^, A. Maleska^5^, K. Ruprecht^2^, W. Su^6^, M. Koch^7^, M. Arkuszewski^8^, N. Tenenbaum^6^, K. Jens^5^, U. Reuter^2,4^

#### ^1^Humboldt Graduate School, Doctoral Program, International Graduate Program Medical Neurosciences, Berlin, Germany; ^2^Charité University Hospital Berlin, Neurology, Berlin, Germany; ^3^Berlin Institute of Health at Charité (BIH), Clinician Scientist Program, Berlin, Germany; ^4^Universitätsmedizin Greifswald, Klinik und Poliklinik für Neurologie, Greifswald, Germany; ^5^University Hospital Basel, University of Basel, Neurologic Clinic and Policlinic, Department of Medicine, Biomedicine and Clinical Research, Basel, Switzerland; ^6^Novartis, East Hanover, NJ, United States; ^7^Novartis, Global Medical Affairs Neuroscience, Basel, Switzerland; ^8^Novartis, Clinical Development Neuroscience, Basel, Switzerland

##### **Correspondence:** L. H. Overeem

**Objective**

The pathomechanisms of the most common neurological disorder, migraine, are not fully understood. This may explain why a stable biomarker for the diagnosis of the disease does not exist. Imaging studies have shown structural changes in the gray and white matter of individuals with migraine. Therefore, we aimed to compare markers associated with structural changes or cell damage in the central nervous system or blood-brain barrier disruption in the blood serum from patients with migraine and healthy controls.

**Methods**

In this cross-sectional study, we assessed blood samples from 92 patients with episodic migraine (EM), 93 with chronic migraine (CM), in the interictal phase, and 42 age-matched healthy controls (HC). Serum total-tau protein (t-tau), neurofilament light polypeptide (NFL), glial fibrillary acidic protein (GFAP), and ubiquitin carboxy-terminal hydrolase L1 (UCH-L1) concentrations were studied. We obtained headache characteristics from headache diaries during the 28 days before blood sampling. Samples were analyzed with a Neurology 4-plex assay kit, on a single molecule array HD-1 Analyzer. Non-parametric tests were used to compare groups and assess correlations.

**Results**

Serum t-tau concentrations were elevated in patients with migraine versus healthy controls (p < 0.05). EM and CM groups were both different from HC (p = 0.002 and p = 0.025, respectively). Migraine aura did not have an effect on t-tau concentrations. The stratification for prophylaxis in CM, showed elevated t-tau concentrations in CM patients without prophylaxis and HC (p = 0.009). No differences between EM and CM, versus HC for NFL, GFAP, and UCH-L1 were observed (p = 0.507, p = 0.850, and p = 0.195).

**Conclusion**

This study did not find biochemical evidence for cell damage in the central nervous system in patients with migraine. The increase of t-tau concentrations in serum may be associated with the disruption of the blood-brain barrier in migraine.

## P157 Disconnectome of the Migraine Brain: A Model of Migraine as "Connectopathy"

### A. Russo, M. Silvestro, M. Cirillo, F. Esposito, A. Tessitore, G. Tedeschi

#### University of Campania "Luigi Vanvitelli", Advanced medical and surgical sciences, Naples, Italy

##### **Correspondence:** A. Russo

Background

Structural brain connectome, characterized by higher stability and reproducibility, have not been investigated in migraine by means of graph analysis approach. We hypothesize a rearrangement of the brain connectome with an increase of both strength and density of connections between cortical areas involved in pain perception, processing and modulation in migraine patients. The connectome rearrangement, misbalancing competing parameters of network efficiency and segregation, may underpin the energetically dysfunctional migraine brain.

Methods

We investigated, using diffusion-weighted MRI imaging tractography-based graph analysis, the graph-topological indices of the brain "connectome", a set of grey matter regions (nodes) structurally connected by white matter paths (edges) in 94 patients with migraine without aura (MwoA) compared to 91 healthy controls (HC).

Results

We observed in MwoA patients compared to HC: i) higher local and global network efficiency (*p* < 0.001) and ii) higher local and global clustering coefficient (*p* < 0.001). Moreover, we found changes in the hubs topology in MwoA patients with: i) posterior cingulate cortex and inferior parietal lobule (encompassing the so-called neurolimbic-pain network) assuming the hub role and ii) fronto-orbital cortex, involved in emotional aspects, and visual areas, involved in migraine pathophysiology, losing the hub role. Finally, we found higher connection (edges) probability between cortical nodes involved in pain processing as well as in cognitive and affective attribution of pain experiences, in migraine patients when compared to healthy controls (*p* < 0.001). No correlations were found between imaging and clinical parameters of disease severity.

Conclusion

The imbalance between the need of investing resources to promote network efficiency and the need of minimizing the metabolic cost of wiring probably represents the mechanism underlying migraine patients" susceptibility to triggers.

**Fig. 1 (abstract P157).**
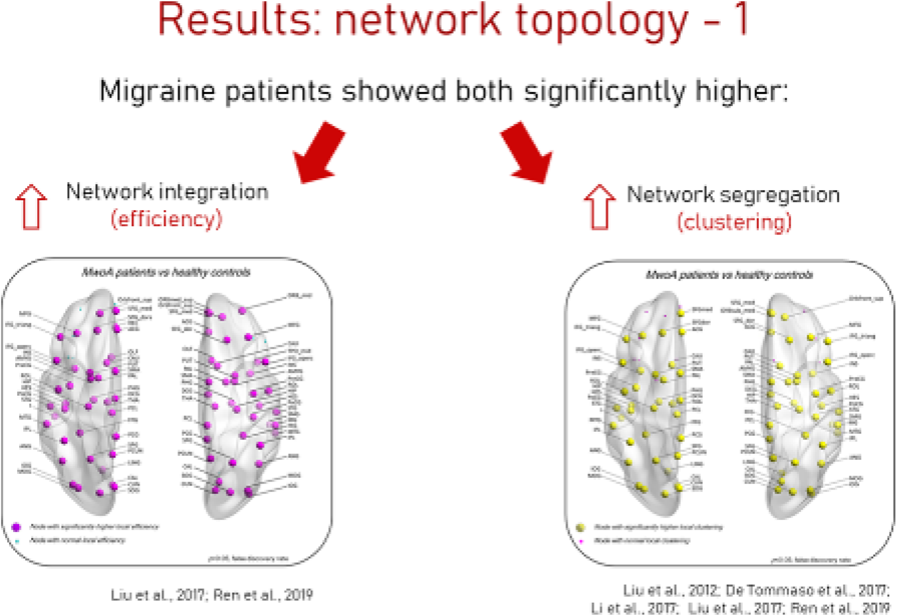
See text for description.

**Fig. 2 (abstract P157).**
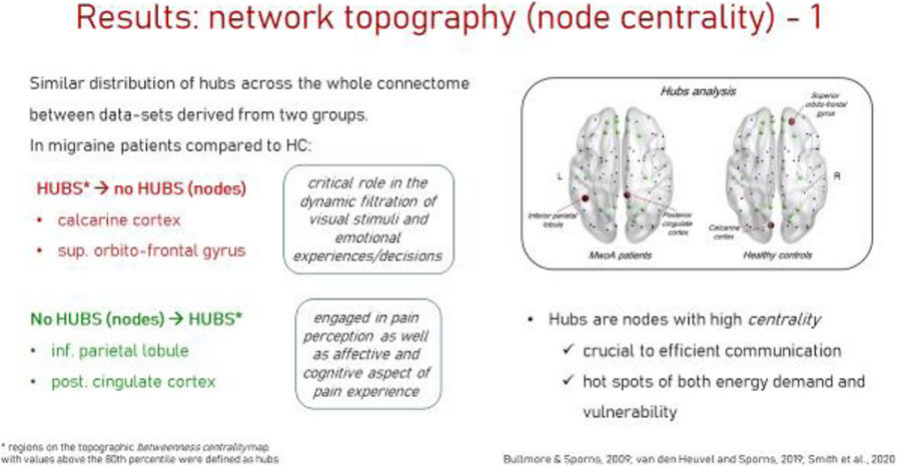
See text for description.

## P158 Changes in Sensitization and Habituation Measured by Algometry in Chronic Migraine Patients Treated with Onabotulinumtoxin A

### Á. L. Guerrero Peral, A. Serra-Mecína, B. Martínez Rodríguez, Y. González-Osorio, A. Recio García, A. Echavarría-Íñiguez, D. García-Azorín

#### University Hospital of Valladolid, Headache Unit, Valladolid, Spain

##### **Correspondence:** Á. L. Guerrero Peral

**OBJECTIVES:** Our aim is to evaluate if changes in sensitization and habituation measured with algometry might be a biomarker for response to OnabotulinumtoxinA (OnabotA) in patients with chronic migraine (CM).

**METHODS**: Observational prospective cohort study. OnabotA therapy (PREEMPT) was initiated in CM patients accordingly with local guidelines. An algometry study was performed prior to OnabotA treatment and after each of three sessions. Response to OnabotA was defined as a decrease of more than 50% of migraine days, compared to the baseline. A mechanical algometer was used to determine Pressure Pain Thresholds (PPT). They were measured on 21 points over the scalp (Fp1, F3, F7, C3, T3, P3, T5, O1, Fp2, F4, F8, C4, T4, P4, T6, O2, Fpz, Fz, Cz, Pz, Oz), based on the normalized positions for electroencephalogram recordings. In each point three measures were performed with a 30-second resting period between them. We considered decreasing or increasing between PPTs (kPa) in these three measures, to establish respectively a pattern of sensitization or habituation. We evaluated change of these PPTs patterns between basal and third determination.

**RESULTS:** 35 patients (91.4% females) were included. Mean age was 43.3 ± 9.1 years (26-60). Twenty-six (74.3%) responded to OnabotA. Basal PPTs showed in 20 points a pattern of sensitization, whilst when measured after 3 OnabotA procedures in 14 points habituation was observed. When comparing responders with no responders, differences between basal and third determination were obtained in T4 (0.05 vs -0.44, p:0.035), F3 (0.21 vs – 0.1, p:0.033) and FP2 (0.33 vs – 0.13, p:0.005)

**CONCLUSION:** Algometry pattern turns from sensitization to habituation in CM patients treated with OnabotA, which, in some points, correlates with response to treatment.

## P159 Mapping Migraine Minds: A cross-sectional survey to compare the difference in burden of migraine among Indian male & female patients

### S. Singh^1^, R. L. Narasimhan^2^, A. Gupta^3^, J. Sharma^4^, U. Sundar^5^, S. Thakur^6^, A. Thorat^6^

#### ^1^Artemis Hospital, Neurology, Gurgaon, India; ^2^Madras Medical College, Neurology, Chennai, India; ^3^Army Hospital, R&R New Delhi, New Delhi, India; ^4^Base Hospital, Neurology, New Delhi, India; ^5^Sion Hospital, Neurology, Mumbai, India; ^6^Novartis, Medical, Mumbai, India

##### **Correspondence:** S. Thakur

**Objectives:** To compare the difference in burden- symptom, functional, social, economic & quality of life (QoL) of migraine among Indian male & female patients.

**Methods:** A cross-sectional survey was conducted from 20th April 2022 – 21st June 2022 in 300 adult male and female (1:1) migraine patients. Survey questionnaire was validated by a steering committee of 10 Indian neurologists. Data was collected by using telephonic and face to face interview mode. The results were analyzed using descriptive statistics

**Results:** Average number of migraine symptoms was higher in females (F) Vs males (M). [5.73 (F); 4.73%(M)]; fatigue [59% (F); 37%(M)] abdominal pain [29% (F); 11%(M)] & stomach upset [21% (F); 11%(M)]. Higher proportion of females had triggers like stress [80% (F); 76%(M)] & physical activity [56% (F); 44%(M)]. Average duration of migraine attack was higher in females [5.8 hours (F); 5.3 hours (M)] with higher comorbidities like anxiety [24% (F); 18%(M)] & obesity [23% (F); 17%(M)]. Higher proportion of females reported that migraine impacted their social & personal life [80.6% (F); 30.6 (M)]. While there was no difference in the direct costs associated with migraine; indirect costs were higher in females (INR 9100) Vs males (INR 8367). More number of working days were lost due to migraine in females than that in males. [3.7 (F); 2.2 (M)].

**Conclusion:** Magnitude of migraine burden in terms of symptoms, functional, social and economic burden among females is higher than that in males in India. Customized approach towards migraine care for females comprising of counselling, lifestyle modification, trigger management & early use of targeted pharmacotherapy would improve clinical outcomes.

**Key words**: Migraine; Burden; Gender; Targeted approach

## P160 Whole-brain functional connectome alterations in patients with migraine

### C. H. Lee^1^, M. J. Lee^2^, B. Y. Park^3,4^

#### ^1^Inha University, Statistics, Incheon, South Korea; ^2^Seoul National University Hospital, Neurology, Seoul, South Korea; ^3^Inha University, Data Science, Incheon, South Korea; ^4^Center for Neuroscience Imaging Research, Suwon, South Korea

##### **Correspondence:** C. H. Lee

Previous neuroimaging studies have examined alterations in brain function in patients with migraine, but the whole-brain investigation is relatively scarce. Here, we aim to assess atypical whole-brain organization of brain function in patients with migraine using functional MRI and dimensionality reduction techniques.

We recruited 50 patients with migraine, and sex- and age- matched healthy controls from Samsung Medical Center. Imaging data were preprocessed using fusion of neuroimaging preprocessing (FuNP) surfaced-based pipeline [Park, 2019, Front. Neuroinform.]. Functional connectivity matrix was constructed by calculating Pearson"s correlation of time series between different brain regions and Fisher"s r-to-z transformed. We generated low-dimensional representations of functional connectivity (i.e., eigenvectors) across the cortex [Margulies, 2016, PNAS], and assessed between-group differences in the eigenvectors between patients with migraine and healthy controls using multivariate analysis with controlling for age and sex. The subcortical alterations were assessed using the nodal degree values of subcortical weighted manifolds, defined by a subcortico-cortical connectivity multiplied by cortical eigenvectors [Park, 2021, Nat. Commun.]. The multiple comparisons were corrected using false discovery rate (FDR)< 0.05.

The eigenvectors showed significant between-group differences in early visual, somatomotor, and temporal pole, as well as amygdala. Stratifying the effects according to seven intrinsic functional communities [Yeo, 2011, J. Neurophysiol.], dorsal attention, visual and limbic network revealed strong effects.

The current study found that migraine is associated with altered brain function in low-level sensory and higher-order limbic systems, including an associated subcortical structure. Our findings may provide insights for understanding whole-brain alterations in migraine.

## P161 Interictal IgE and tryptase levels in episodic and chronic migraine

### S. Cho^1^, S. J. Kim^1^, H. J. Lee^1^, S. H. Lee^1^, W. Lee^2^, M. K. Chu^1^

#### ^1^Yonsei University College of Medicine, Neurology, Seoul, South Korea; ^2^Yongin Severance Hospital, Neurolohy, Yongin, South Korea

##### **Correspondence:** M. K. Chu

**Questions:** A close association between migraine and allergic diseases has been reported. IgE and mast cells play key roles in the development of allergic diseases. Tryptase has been used a marker of mast cell activation. Although altered levels of IgE in migraine was reported, no study separately evaluated the IgE and tryptase levels in episodic migraine (EM) and chronic migraine (CM).

**Methods:** The IgE and tryptase levels were measured by fluorescence enzyme immune assay method on a ThermoFisher Phadia 250 system. We collected plasma ≥ 48 h having passed after the cessation of a typical migraine attack, being headache-free (for participants with EM), and having mild or less headache intensity (for participants with CM). We also evaluated the history of allergic disease among participants.

**Results:** This study enrolled 95 and 96 participants with EM and CM, respectively and 56 controls. 88 of participants (42, 40, and 8 of EM, CM and controls) had allergic diseases. Among participants with allergic diseases, IgE levels were significantly different among participants with EM, CM and controls (81.6 [42.0-248.3] vs. 46.5 [15.9-116.0] vs. 195.0 [78.2-301.0] KU/L, p=0.025). Nevertheless, tryptase levels did not significantly differ among three groups (3.4 [2.3-4.1] vs. 3.3 [2.3-4.2] vs. 3.7 [2.8-3.8] ng/ml, p=0.625). IgE levels among participant with allergic diseases, headache frequency was inversely associated with IgE levels (Pearson"s correlation coefficient = - 0.261, p=0.019). Among 109 participants without allergic diseases, IgE (43.5 [27.8-99.8] vs. 45.1 [23.2-99.0] vs. 50.7 [24.2-114.0] KU/L, p=0.832) and tryptase (3.4 [2.3-4.1] vs. 3.3 [2.7-3.9] vs. 3.3 [2.5-4.3] ng/ml, p=0.862) levels did not significantly differ among three groups.

**Conclusions:** IgE levels were significantly differ in participants with allergic diseases among those with EM, CM and controls.

## P162 Efficacy and safety of once monthly subcutaneous erenumab 70 mg in adult chronic migraine patients (primary analysis): Indian sub analysis from Global DRAGON study

### D. Chowdhury^1^, R. Baviskar^2^, S. Thakur^3^, A. Thorat^3^

#### ^1^GB Pant Hospital, Neurology, Delhi, India; ^2^Neurocare, Neurology, Nashik, India; ^3^Novartis, Medical, Mumbai, India

##### **Correspondence:** S. Thakur

**Objectives:** DRAGON (CAMG334A2304),12-week, double-blind randomized study evaluated efficacy & safety of erenumab (70 mg) in adult Chronic migraine (CM) patients from China, Taiwan, Korea, Southeast Asia & India. This is India sub-set analysis of the global DRAGON study.

**Methods:** Patients (N=30) were randomized to placebo or erenumab 70 mg (1:1). Primary endpoint was change from baseline in monthly migraine days (MMD). Secondary endpoints were ≥50% reduction in MMD, changes in modified Migraine Disability Assessment (mMIDAS), changes in monthly acute headache medication days (MHMD), & safety/tolerability.

**Results:** Mean (SD) age was 34.5 (10.8) years, 73.3 % were women; mean MMD was 15.14 (4.93) & only 53.3% had prior preventive treatment failure. Similar change in MMD from baseline at week 12 was observed in erenumab 70 mg group & placebo [-8.37 and -8.62 respectively) (*p*=0.913)]. Patients achieving ≥50% reduction in MMD was higher in erenumab 70 mg vs placebo (82.4% vs 69.2%; *p*=0.379) Change in mMIDAS was -11.13 with placebo & -11.92 with erenumab 70 mg (*p*=0.496). Change in MHMD was -3.90 with placebo & –3.48 for erenumab 70 mg (*p*=0.556). No AEs leading to discontinuation nor SAEs or deaths were reported in either group. Safety & tolerability of erenumab was comparable to placebo with no new safety signals.

**Conclusion:** While Indian subset study was not powered to detect statistically significant differences, Erenumab (70mg) s.c QM Vs placebo showed numerical superiority for achieving ≥50% reduction in MMDs. Clinically meaningful reduction in mMIDAS, & MHMD at 12 weeks with favourable safety profile in Indian CM patients & no new safety signals were detected

**Key words:** India; Chronic Migraine; erenumab; anti-CGRP

**Table 1 (abstract P162).**
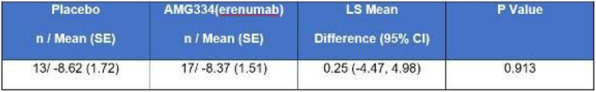
Change from baseline in Monthly Migraine Days at Week 12

**Table 2 (abstract P162).**
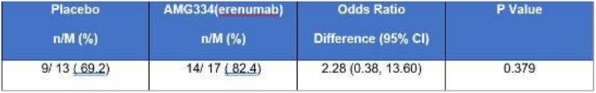
Proportion of subjects with at least a 50% reduction in Monthly Migraine Days at Week 12

## P163 Subjective Tinnitus in Pediatric and Adolescent Migraine Versus Other Primary Headaches – a Prospective, Comparative Study

### T. Eidlitz Markus^1,2^, Y. Levinsky^1,2^, A. Brameli^1,2^

#### ^1^Tel Aviv University, Sackler Faculty of Medicine, Tel Aviv, Israel; ^2^Schneider Children's Medical Center, Day Hospitalization Department, Petach Tikva, Israel

##### **Correspondence:** T. Eidlitz Markus

Background: Subjective tinnitus is perception of sound in the ear, or in the head occurring without an outside acoustic stimulus. Headache and especially migraine has been reported as associated with tinnitus in adults but not in children. The study aimed to investigate the prevalence of tinnitus and its associated clinical parameters in pediatric and adolescent migraine versus other primary headaches. Methods: In the pediatric headache clinic of a tertiary hospital, patients aged 8-18 years and their parents were interviewed regarding their headache symptoms and according to a validated tinnitus questionnaire. Patients with tinnitus were referred for audiometry. Results: Of 153 patients, 90 (58.5%) were females; the mean age was 7.9±2.74 years. Ninety-four (61.4%) were diagnosed with migraine and 59 (38.6%) with primary headaches. The rate of tinnitus was significantly higher among patients

with migraine than among patients with other primary headaches (47.9% vs 10.2%, p

**Fig. 1 (abstract P163).**
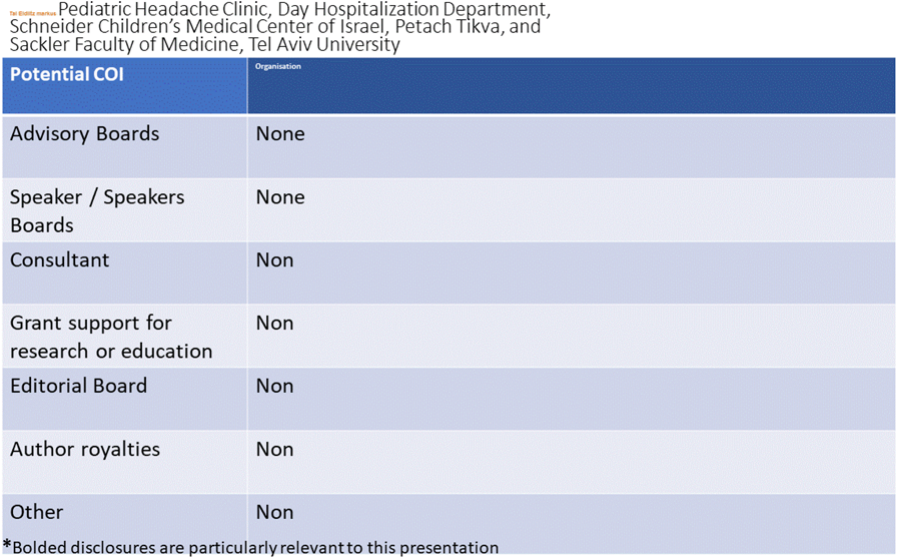
See text for description.

**Fig. 2 (abstract P163).**
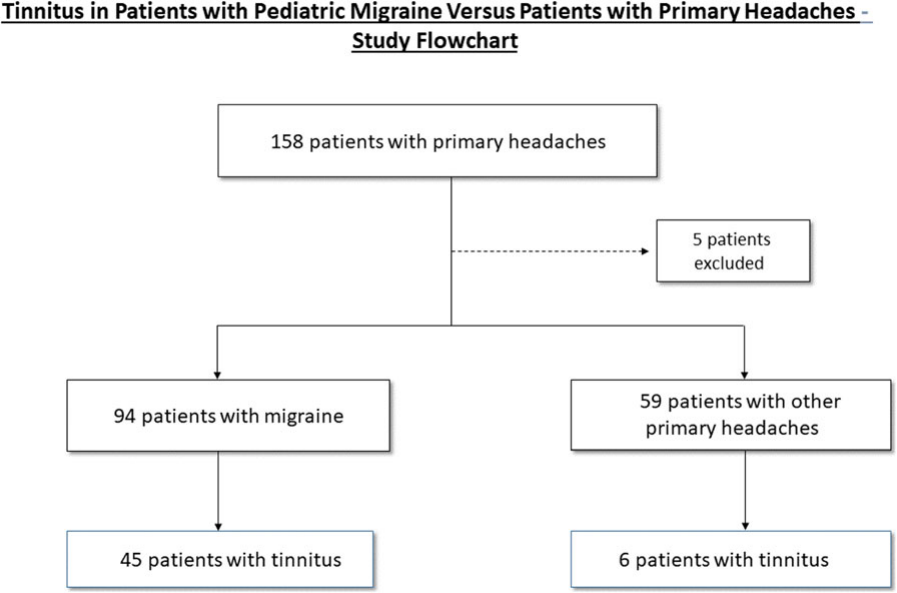
See text for description.

## P164 Influence of Neck Pain in Response to Cervical Spine Manual Palpation on Neck Proprioception and Muscle Endurance of Migraine Patients and Controls

### G. F. Carvalho^1^, T. M. Szikszay^1^, W. M. Adamczyk^2^, A. Schwarz^3^, D. Bevilaqua Grossi^4^, A. May^5^, K. Luedtke^1^

#### ^1^University of Luebeck, Institute of Health Sciences, Luebeck, Germany; ^2^The Jerzy Kukuczka Academy of Physical Education, Laboratory of Pain Research, Katowice, Poland; ^3^Hochschule Bremen, Bremen, Germany; ^4^Ribeirao Preto Medical School, Institute of Health Sciences, Ribeirão Preto, Brazil; ^5^University of Hamburg-Eppendorf, Department of Systems Neuroscience, Hamburg, Germany

##### **Correspondence:** G. F. Carvalho

The aim of this study was to investigate the cervical joint position sense (JPSE) and the neck muscle endurance among migraineurs and controls following stratification according to pain response over the cervical spine. Thirty-two headache-free subjects and 57 migraineurs were included. The sample was stratified according to the presence of pain during the manual palpation of the upper cervical spine: no neck pain (P0, n=23), local neck pain (P1, n=37) and pain referred to the head (P2, n=29). All subjects were instructed to perform the cervical JPSE for extension, right and left rotation. All subjects also underwent the muscle endurance test of the neck flexors and extensors. A significant effect of neck dysfunction for the JPSE was found (P0 versus P2 mean difference=1.93 cm, F2=4.85, p=0.008). No diagnosis effect was verified for all movement directions (p>0.39), and no neck dysfunction effect was verified for the remaining right (p>0.83) and left (p>0.46) directions. For the muscle endurance test, a significant effect was found for diagnosis (migraine vs no migraine) and neck dysfunction. Compared to migraineurs, controls exhibited greater muscle endurance of neck flexors (mean difference 15 sec, F1=10.54, p=0.001) and extensors (mean difference 48 sec, F1=4.93, p=0.02). According to the stratification, subjects of the P0 group exhibited greater muscle endurance compared to P1 for neck flexors (mean difference 27 sec, F2=16.21, p<0.001) and extensors (mean difference 46 sec, F2=3.20, p=0.04). The P0 group also was different from the P2 group regarding flexion (mean difference 29 sec, F2=16.21, p<0.001) and extension (mean difference 68.4 sec, F2=3.20, p=0.04) endurance. No differences between P1 and P2 groups were found. The presence of neck pain referred to the head is related to a greater JPSE for neck extension. The presence of migraine and/or any neck dysfunction is related to reduced endurance of neck flexor and extensor muscles.

## P165 Association Between Migraine and Transient Global Amnesia: An Inpatient Sample Analysis

### C. Lampl, K. Aschauer, B. Juranek, N. Haselgruber

#### Konventhospital Barmherzige Brüder Linz, Neurologie, Linz, Austria

##### **Correspondence:** C. Lampl

Introduction: Transient global amnesia (TGA) constitutes an enigmatic amnestic syndrome characterized by temporary memory dysfunction of abrupt onset and total resolution within 24 h from emergence. Among the most prevailing theories implicates migraine and the neurophysiologic substrate of aura, which is caused by the release of massive glutamate and a subsequent wave of short-lasting cortical depolarization (CSD). Given this relationship between migraines and transient hippocampal dysfunction, it is feasible to consider the possibility of an etiological relationship between migraines and the transient memory problems in TGA individuals. The main focus of the study was to investigate the comorbidity of migraine among the TGA study population to analyze a potential association with TGA. Methods: Data extraction war performed between January 2007 and March 2021. Descriptive statistics were displayed as mean ± standard deviation for continuous data and frequencies with percentages for categorical variables. For group comparisons we used Chi-Quadrat-Test, Cramer’s V was performed to measure the relative strength of an association. The Mann–Whitney U test was used as analog nonparametric test. Results: From the study period of 14 years and 3 months 641 persons (62,1% women;37,9% men) with TGA were analyzed and evaluated. Mean age at the time TGA was diagnosed was 66,1 years of age (SD=10.02). Overall, 5,9% of the TGA persons preported a history of migraine. Furthermore, women with a history of migraine were 3,75 times more likely to suffer from TGA than men (p

## P166 Gradually shifting clinical phenomics in migraine spectrum: A Cross-sectional, Multicenter study of 5438 patients

### Y. Ran^1^, Z. Yin^2^, Y. Lian^3^, Y. Xu^4^, Y. Li^5^, J. Liu^5^, Q. Gu^6^, F. Yan^7^, Z. Ge^8^, Y. Lian^9^, D. Hu^10^, S. Chen^11^, Y. Wang^12^, X. Wang^1^, R. Wang^1^, X. Chen^1^, J. Liu^1^, M. Zhang^1^, X. Han^1^, W. Xie^1^, Z. Yu^1^, Y. Cao^1^, Y. Li^1^, K. Li^1^, Z. Dong^1^, S. Yu^1^

#### ^1^Chinese PLA General Hospital, Neurology, Beijing, China; ^2^University of Shanghai for Science and Technology, Shanghai, China; ^3^The first affiliated hospital of Zhengzhou university, Zhengzhou, China; ^4^Dingyuan general hospital, Chuzhou, China; ^5^The centre hospital of jilin city, Jilin, China; ^6^Huzhou first peolple’s hospital, Huzhou, China; ^7^Linyi Hinluo Hospital, Linyi, China; ^8^Shenzhen second people’s hospital, Shenzhen, China; ^9^Inner Mongolia xing’an league people’s hospital, Hinggan, China; ^10^The second affiliated hospital of Shandong first medical university, Shandong, China; ^11^Changsha Central Hospital affiliated to University of South China, Changsha, China; ^12^Chinese PLA General Hospital, Pediatric Center, Beijing, China

##### **Correspondence:** Y. Ran

*Objective*: The aim of the study was to investigate whether MwoA and MwA are different manifestations of a single disease, distinct clinical entities, or located at two poles of a spectrum.

*Methods*: In this cross-sectional study, 5438 patients from 10 hospitals in China were included: 4651 were diagnosed with migraine without aura (MwoA) and 787 with migraine with aura (MwA). We used a validated standardized electronic survey to collect multidimensional data on headache characteristics and evaluated the similarities and differences between migraine subtypes. To distinguish migraine subtypes, we employed correlational analysis, factor analysis of mixed data (FAMD), and decision tree analysis.

*Results*: Compared to MwA, MwoA had more severe headaches, predominantly affected females, were more easily produced by external factors, and were more likely to have accompanying symptoms and premonitory neck stiffness. Patients with MwA are heterogeneous, according to correlation analysis; FAMD divided the subjects into three clear clusters. The majority of the differences between MwoA and MwA were likewise seen when typical aura with migraine headache (AWM) and typical aura with non-migraine headache (AWNM) were compared. Furthermore, decision trees analysis revealed that the chaotic MwA data reduced the decision tree"s accuracy in distinguishing MwoA from MwA, which was significantly increased by splitting MwA into AWM and AWNM.

*Conclusions*: The clinical phenomics of headache phenotype varies gradually from MwoA to AWM and AWNM, and AWM is a mid-state between MwoA and AWNM. We tend to regard migraine as a spectrum disorder, and speculate that different migraine subtypes have different "predominant regions" that generate attacks.

**Fig. 1 (abstract P166).**
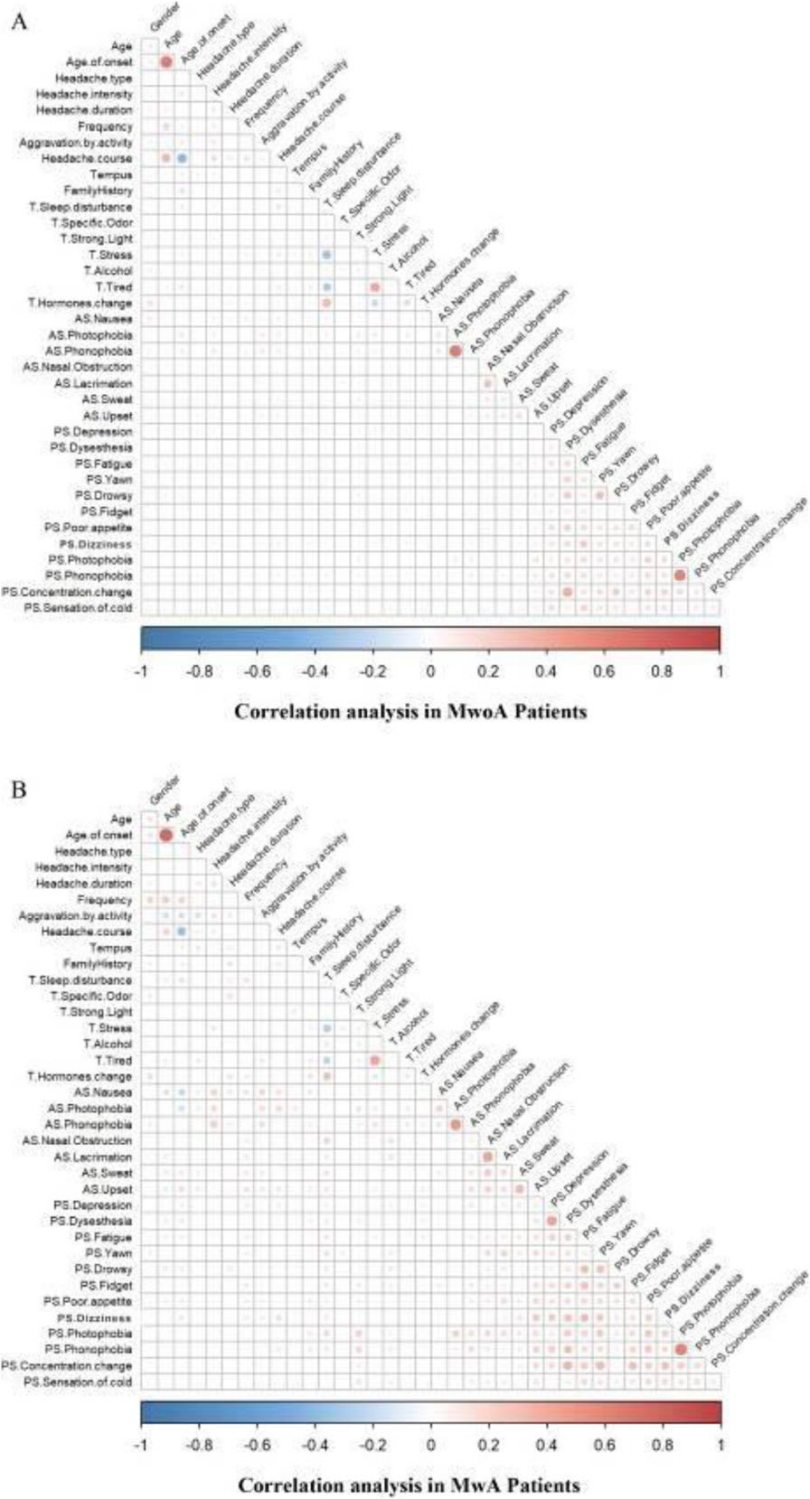
See text for description.

**Fig. 2 (abstract P166).**
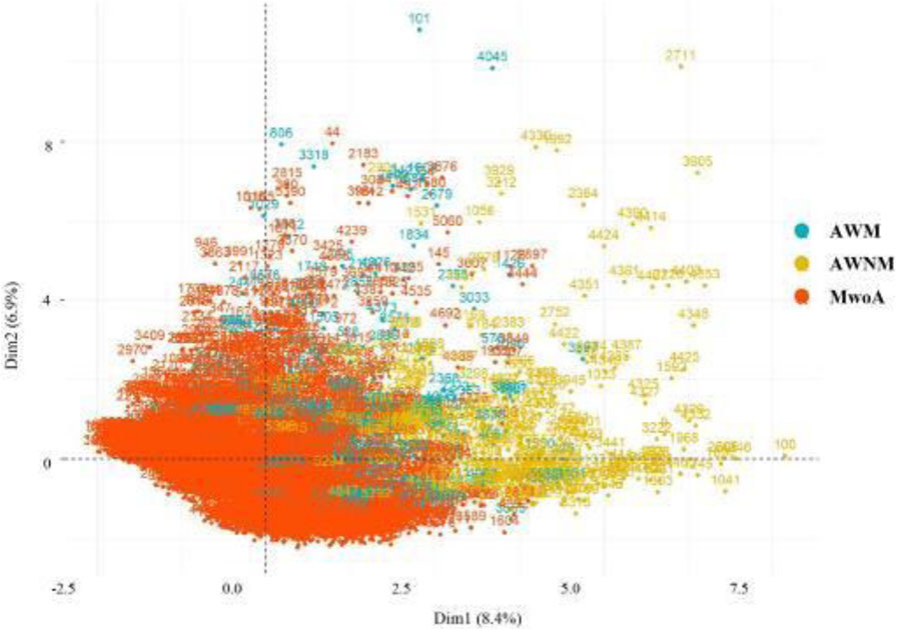
See text for description.

## P167 Characterization of the spectral content of resting-state electroencephalographic activity in chronic migraine female patients

### V. Gutiérrez-de Pablo^1^, Á. L. Guerrero Peral^2^, D. García-Azorín^2^, Á. Sierra-Mencía^2^, J. Gómez-Pilar^1^, J. Poza^1^, R. Hornero^1^, C. Gómez^1^

#### ^1^University of Valladolid, Biomedical Engineering Group, Valladolid, Spain; ^2^University Hospital of Valladolid, Headache Unit, Valladolid, Spain

##### **Correspondence:** V. Gutiérrez-de Pablo

**Objective.** Previous studies have reported neurophysiological differences between chronic migraine (CM) and healthy controls (HC). The aim of the current study is to evaluate how the CM condition affects the brain activity in women using spectral measures.

**Methods.** We have included 62 female subjects: 32 CM patients (age 34.50 (27.50, 39.00)) and 30 HC subjects (age 29.00 (26.00, 35.00)). Ten minutes of eyes-closed resting-state electroencephalographic (rsEEG) activity were acquired using a Brain Vision® equipment. The power spectral density (PSD) of rsEEG recordings was computed to assess the spectral content of the brain electrical activity. Nine spectral parameters were computed from the PSD: individual alpha frequency, transition frequency, median frequency (MF), spectral edge frequency, relative power (RP) in the conventional frequency bands, spectral entropy, Rényi entropy, Tsallis entropy, and Escort-Tsallis entropy.

**Results.** Statistically significant differences (*p* < 0.05, Mann-Whitney *U*-test) were found in the spectral content of PSD in terms of MF, and RP in beta 1 and beta 2 frequency bands. In addition, PSD irregularity, assessed by means of spectral entropy, Tsallis entropy, and Rényi entropy, showed differences between both groups. Furthermore, CM patients exhibited significantly higher values for MF, RP in beta 1 and beta 2 bands, spectral entropy, Tsallis entropy, and Rényi entropy than HC subjects, which suggests that CM induces an increase in the oscillatory activity in high-frequency bands and irregularity in rsEEG activity.

**Conclusions.** Our analyses showed that CM is associated with an increase in both high-frequency oscillatory activity and irregularity in rsEEG activity compared with HC. These findings could be exploited to provide further understanding on CM.

## P168 Impact of air pollution exposure on headache onset in migraine patients

### A. Alpuente, A. Torre-Suñe, J. Cloquell, V. J. Gallardo, P. Pozo-Rosich

#### Vall d'Hebron University Hospital, Neurology, Barcelona, Spain

##### **Correspondence:** A. Alpuente

**Question:** Air pollution has a clear impact on people health"s increasing the risk of suffering from several diseases. We aimed to analyze whether if ambient air pollution triggers migraine attacks

**Methods:** This is a prospective longitudinal study. Headache daily status (headache free vs headache day) and GPS coordinates were collected using a custom-developed Smartphone App. Patients with migraine diagnosis seen in the Headache Clinic were recruited. Daily maximum 1-hour nitric oxide (NO), carbon monoxide (CO), sulfur dioxide (SO2), fine particulate matter (PM10) and ozone (O3) levels in the previous four days before headache onset were considered as possible explanatory variables for a binary outcome variable describing the potential daily headache status. A mixed-effects logistic regression model was performed. The model was adjusted by patient characteristics at baseline and meteorological parameters (daily mean temperature, relative humidity, accumulated precipitation, radiation and wind speed levels). It was validated using repeated 10-fold cross-validation.

**Results:** Sixty-six patients (80.3% women, mean age 48.7±9.2 years) contributed to 12,233 days of data, from which 1,668 days (13.6%) were headache days. Statistically significant differences in levels of daily maximum 1-hour NO (p=0.041), SO2 (p=0.010) and O3 (p=0.010) were found over the previous four days before headache onset. An increase in daily maximum 1-hour levels of NO2 of 35 μg/m³ (0.5·IQR, Interquartile Range) in the previous 48 hours was found to increase a 5.6% the probability of a headache onset (p=0.035). The presence of an attack on previous days was also associated with potential headache risk.

**Conclusions:** Headache onset in migraine patients might be influenced by greater air pollution on previous days. Air pollution, combined with other external and individual internal factors, has an impact on health status and might contribute to triggering migraine attacks.

## P169 A Cohort Study Comparing Melatonin-users with Non-users in the treatment of Migraine Headaches

### S. Singhvi^1^, G. Bhandari^1^, P. Singh^2^, S. Lakhotia^3^

#### ^1^Manidhari Hospital, Jodhpur, Rajasthan, India, Department of Medicine, Jodhpur, India; ^2^Smt.NHLMMC and SVP Hospital, Ahmedabad, India; ^3^BJMC and Civil Hospital Ahmedabad, Department of Psychiatry, Ahmedabad, India

##### **Correspondence:** S. Singhvi

**Question**

Melatonin improves sleep quality and severity of headaches, when compared to baseline studies. It is a safe drug with minimal side effects and is well tolerated. This research will further highlight the potential of Melatonin for treatment of migraine headaches.

**Method**

A cross sectional study was performed on 120 participants in a hospital setting where the users and non-users of Melatonin were divided into groups of 60 each. A MIDAS (Migraine Disability Assessment Scale) questionnaire was used to assess the symptoms. To establish a correlation between different factors, the chi-square test and p-value were used. A null hypothesis was formulated with no association taken into account, and the chi-square and p-values were computed to find possible association between the considered factors. The confidence interval for p-value was taken at 95% with a 0.05 level of significance.

**Results**

It was found that there was a reduction in the intensity of pain and improvement in quality of sleep in the Melatonin user against the non-user cohort with an average age of 30, as evident from the results of chi square and p value of 7.5481 and 0.006007 for number of night time awakenings (relative risk reduction= 0.539 and number needed to treat= 4.291) and chi-square value of 36 and p-value of 0.00001 for intensity of pain reduction (relative risk reduction= 0.473, number needed to treat=1.897). The values for mean frequency headache reduction went down from 3.23±0.26 in the non-user cohort to 2.1±0.67 in the user cohort.

**Conclusions**

Migraine is a condition that causes recurring headaches with severity ranging from mild to moderate in intensity. It is a chronic, debilitating condition that reduces the quality of life. Melatonin has drawn attention for its anti-migraine action, owing to its structural similarity to Indomethacin and free-radical neutralizing property. Therefore, Melatonin can be a promising agent for treatment of migraine headaches.

## P170 Epilepsy in Migraine Aggravates the Cognitive Disorders

### M. Mavlanov, F. Saidvaliyev

#### Tashkent Medical Academy, Neurology, Tashkent, Uzbekistan

##### **Correspondence:** M. Mavlanov

**Purpose**: To study the features of cognitive impairment in patients with migraine and migraine comorbid epilepsy.

**Materials and methods:** 47 patients with migraine comorbid epilepsy were examined. Of these, there were 16 men (34%), women - 31 (66%), the average age of which was 27.4±2.2 years. For the control group, 128 patients with migraine were examined, of which 82 (64%) were women, 46 (36%) were men, the average age of which was 27.5±2.1. Cognitive function was studied using the MMSE test, MSCT/MRI of the brain.

**Results:** Migraine with aura was diagnosed in 20 (42.6%) patients, migraine without aura 27 (57.4%) caused an epileptic attack. In 5 (10.6%) cases, one seizure was detected, in the remaining 42 (89.4%) cases, two or more episodes of seizures were detected. In the control group, 26 (20.3%) patients were diagnosed with migraine with aura, 95 (74.2%) patients with migraine without aura. Studying cognitive function, it was found that 85.1% (n=40) of patients with migraine comorbid epilepsy had moderate and mild cognitive impairment. In the control group, only 27.3% (n=35) of cases had moderate cognitive impairment. Neuroimaging revealed ischemic changes in the cerebral cortex, periventricular and subcortical white matter in 87.2% (n=41) of cases in the main group and in 38.3% (n=49) of patients in the control group. MMSE data had a direct correlation with MRI/MSCT data with P<0.001 changes.

**Conclusions:** Accession of an epileptic seizure in migraine is combined with a decrease in cognitive function and is associated with vascular complications of the brain. Cognitive impairment in migraine comorbid epilepsy requires correction of this condition.

## P171 Impact of migraine in Indian housewives: A subset analysis from Mapping Migraine Minds study- A cross-sectional study to compare the difference in burden of migraine among Indian males & females

### S. Singh^1^, R. L. Narasimhan^2^, A. Gupta^3^, J. Sharma^4^, U. Sundar^5^, S. Thakur^6^, A. Thorat^6^

#### ^1^Artemis Hospital, Neurology, Gurgaon, India; ^2^Madras Medical College, Neurology, Chennai, India; ^3^Army Hospital, R&R New Delhi, New Delhi, India; ^4^Base Hospital, Neurology, New Delhi, India; ^5^Sion Hospital, Neurology, Mumbai, India; ^6^Novartis, Medical, Mumbai, India

##### **Correspondence:** S. Thakur

**Objective:** To highlight migraine impact in terms of symptom, functional, social & economic burden among Indian housewives.

**Methods:** A cross sectional survey was conducted from 20th April 2022 – 21st June 2022 in 300 adult male and female (1:1) migraine patients. Survey questionnaire was validated by a steering committee of 10 Indian neurologists. Data was collected using telephonic & face to face interview mode. Results were analysed using descriptive statistics

**Results:** Our study included 120 housewives; of which 34% reported 4-7 monthly migraine days (MMDs), 40% 8-14MMDs & 26% had 15 days or more. Major symptoms observed were headache (88%), fatigue (62%), Blurred vision (60%), loss of appetite (57%) & dizziness (47%). Average duration of migraine episode was 5.9 hours. Migraine resulted in productivity loss of 3.8 days/month and 2.3 hours/day. 85.8% of housewives reported that migraine has impacted their social life. Of these, 74% felt guilty, 63% felt isolated & 47% felt depressed / helpless. 69% felt that migraine may damage their relationship with spouse. 100% housewives with children (n=95) reported that migraine has affected their children; 62% reported compromised academics, 57% with reduced ability to parent, 69% for anxiety in children, 67% for frustration & 45% for mood change / irritability. Indirect cost of migraine was INR 8958/6 months in addition to direct medical costs which was less than other females.

**Conclusion:** Migraine impact among Indian housewives is significantly high. Total cost incurred for housewives is the least, highlighting healthcare neglect. Holistic approach of patient education, lifestyle interventions & target specific pharmacotherapy may improve their quality of life.

**Key words:** Migraine Burden; India; Housewives; Quality of life

## P172 The effect of botulinum toxin on anxiety and depression scales in chronic and high frequency migraneurs

### J. Moniz Dionísio, A. R. Pinheiro, Â. Abreu, S. Machado, E. Parreira

#### Hospital Prof. Doutor Fernando Fonseca, Neurology, Lisbon, Portugal

##### **Correspondence:** J. Moniz Dionísio

**Objective**: To evaluate the impact of botulinum toxin (BoNT) on depression and anxiety scales in patients with migraine.

**Methods**: Unicentric prospective study, with data analysis of 40 patients with chronic and high frequency migraine, refractory to several oral migraine-preventive medications, from 2016 to 2022. Data concerning demographic, clinical, comorbid, and therapeutic data was obtained. Validated clinical questionnaires were applied regarding pain characteristics (*Head Impact Test-6* – HIT-6, *Headache Under-Response to Treatment* – HURT, and *Migraine Disability Assessment* - MIDAS), and psychological comorbid states (*STAI Form Y-1* and *Form Y-2* and *Zung Depression Scale*). SPSS® 28 was used for statistical analysis.

**Results**: Eighty-seven-point five percent were female, with a mean age at the end of treatment of 46 years (σ=12.52). 30% of patients were previously diagnosed with an anxiety disorder and 37.5% had been diagnosed with depression. At 6 months of treatment, all scales showed a consistent reduction, especially HIT-6 (R^2^=0.932) and HURT (R^2^=0.956) scores. On linear regression analysis, a positive relation was found between both STAI Y-1 and STAI Y-2 and MIDAS scales (R^2^=0.785, R^2^=0.878, p2 = 0.213, R^2^=0.266, p<0.05), where STAI Y-2 (anxiety state) seemed to have a better correlation with pain variation. No significant statistical relation was found when analyzing the groups that had been previously diagnosed with anxiety and/or depression. No significant correlations were found when analyzing Zung Depression Scale with pain scales.

**Conclusions**: BoNT seems to have a positive effect on anxiety. This effect is possibly related with treatment efficacy, as measured by MIDAS and HIT-6, and it does not seem to be affected by previous diagnosis of anxiety disorders.

## P173 Intravenous lidocaine and ketamine infusions for headache disorders: a retrospective cohort study

### J. Ray^1,2,3^, S. Cheng^1^, K. Tsan^4^, H. Hussain^4^, R. Stark^1,3^, M. Matharu^5^, E. Hutton^1,3^

#### ^1^Alfred Health, Neurology, Melbourne, Australia; ^2^Austin Health, Neurology, Melbourne, Australia; ^3^Monash University, Neurosciences, Melbourne, Australia; ^4^Monash University, Melbourne, Australia; ^5^Queen Square Institute of Neurology and The National Hospital for Neurology and Neurosurgery, London, United Kingdom

##### **Correspondence:** J. Ray

Objective: To assess the safety of real-world efficacy and safety of intravenous lidocaine and ketamien for headache disorders.

Methodology: Patients admitted between 01/01/2018 and 31/07/2021 were identified by ICD code and electronic prescription. Efficacy of infusion was determined by reduction in visual analogue score (VAS), and patient demographics were collected from review of the hospital electronic medical record.

Results: Through the study period, 83 infusions (50 lidocaine, 33 ketamine) were initiated for a headache disorder (77 migraine, 3 NDPH, 2 SUNCT, 1 cluster headache). In migraine, lidocaine infusion achieved a ≥50% reduction in pain in 51.1% over a mean 6.2 days (SD 2.4). Ketamine infusion was associated with a ≥50% reduction in pain in 34.4% over a mean 5.1 days (SD 1.5). Side effects were observed in 32% and 42.4% respectively. Infusion for medication overuse headache (MOH) led to successful withdrawal of analgesia in 61.1% of lidocaine, and 41.7% of ketamine infusions.

Conclusion: Lidocaine and ketamine infusions are an efficacious inpatient treatment for headache disorders, however associated with prolonged length-of-stay and possible side-effects.

## P174 HEADWORK as innovative tool for monitoring MABs efficacy in migraine and their influence on work activity

### D. A. Montisano^1^, G. Vaghi^2^, D. D'Amico^3^, A. Raggi^3^, G. Sances^2^, C. Tassorelli^2,4^, L. Grazzi^3^

#### ^1^University of Milan-Bicocca, Milano, Italy; ^2^Fondazione Istituto Neurologico Mondino di Pavia, Pavia, Italy; ^3^IRCCS Foundation “Carlo Besta” Neurological Institute, Milano, Italy; ^4^Università degli studi di Pavia, Department of Brain and Behavioural Sciences, Pavia, Italy

##### **Correspondence:** G. Vaghi and L. Grazzi

QUESTION:Monoclonal antibodies (MABs) have been a game changer in the treatment of migraine since their approval. Their efficacy is generally assessed with disease related metrics, but there is an increasing need to evaluate the impact of disease treatment on the global burden on patients and society. HEADWORK (HW) is a new evaluation tool, developed specifically to assess the impact on work tasks and reduced productivity of migraineurs. The aim of this study was to test the performance of HW on migraine patients treated with MABs.

METHODS:We enrolled 56 patients receiving treatment with MABs at the Headache Centres of IRCCS "C. Besta" (Milan) or IRCCS "C. Mondino" (Pavia). They were assessed with the HW questionnaire at baseline and at the 3rd (M3) and 6th month (M6) of treatment. HW questionnaire consists of two sections: "Work-related difficulties" (HW1),11 items dealing with the degree of difficulty in general skills, problems solving or starting new task; "Factors contributing to work-related difficulties" (HW2), 6 items to address the degree to which some factors, such as noise and brightness of the workplace, negatively impact work-related tasks.

RESULTS:Population: 10M and 46F, mean age (49.5y±8.7), mean age at onset of disease (18y±8),mean duration of disease (34y±11.2). We observed a marked and consistent reduction in "classical" indicators: monthly migraine days (15±5.9 at baseline, 5±6.27 at M3, 7±6.8 at M6), medications per month (15±9.3 at baseline, 5±13.7 at M3, 6±7.4 at M6), MIDAS (48±36.8 at baseline, 8.5±17.4 at M3, 5±11 at M6), HIT-6(66±3 at baseline, 62±8.3 at M3, 59±8.2 at M6).HW scores paralleled the above parameters: HW1(20±8.3 at baseline, 10.5±5.8 at M3 , 7±8.5 at M6), HW2(10.5±5.8 at baseline,6±4.4 at M3, 4±4.1 at M6).

CONCLUSION:Our findings confirm the effectiveness of MABs. HW1 and HW2 also show an extremely positive impact on work related activities. HW appears a suitable tool to assess migraine-related work disability in these patients.

**Fig. 1 (abstract 174).**
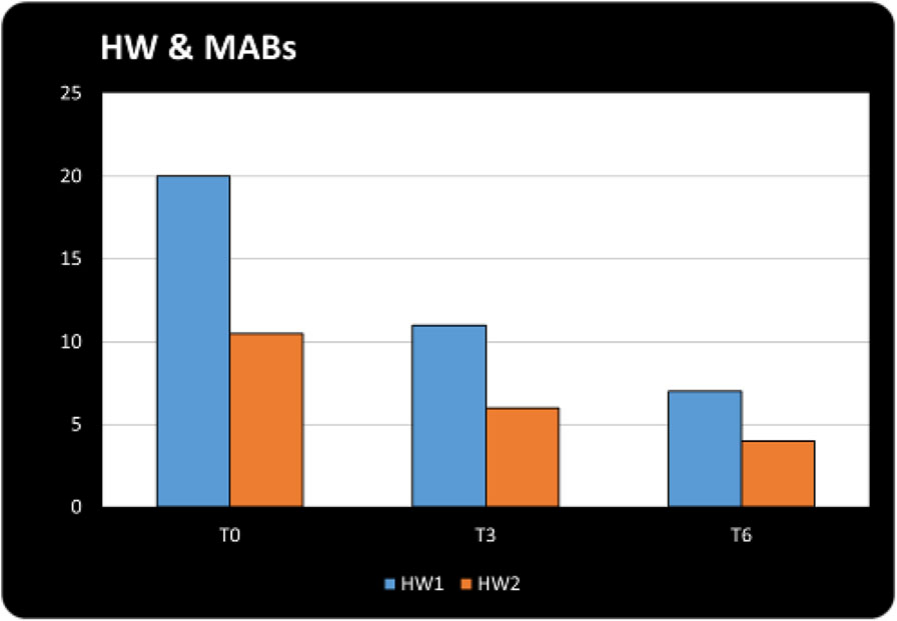
See text for description.

**Fig. 2 (abstract 174).**
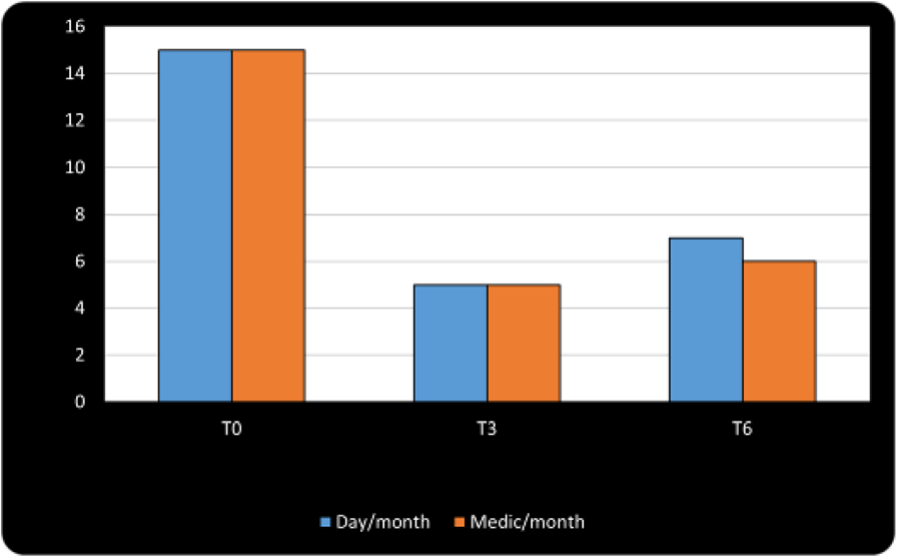
See text for description.

## P175 Optimization of Acute Treatment in Migraine Patients Treated with Monoclonal Antibodies Acting on the CGRP Pathway

### C. Rosignoli^1^, R. Ornello^1^, V. Caponnetto^1^, A. Onofri^1^, L. Tartaglione^2^, A. Russo^2^, M. Silvestro^2^, S. Sacco^1^

#### ^1^University of L'Aquila, Neuroscience Section, Department of Applied Clinical Sciences and Biotechnology, L'Aquila, Italy; ^2^University of Campania “Luigi Vanvitelli”, Department of Advanced Medical and Surgical Sciences (DAMSS), Naples, Italy

##### **Correspondence:** C. Rosignoli

**Question:** We aimed at assessing the impact of monoclonal antibodies targeting the calcitonin gene-related peptide pathway (CGRP-mAbs) on migraine outcomes that are not usually captured by literature, including the optimization of acute treatments.

**Methods:** Consecutive patients with chronic or episodic migraine from the Headache Centers of Avezzano-L'Aquila and Naples, were included from March 2021 to June 2022. We included and followed up to 3 months patients starting treatment with any CGRP-mAb (erenumab, fremanezumab, or galcanezumab) at the baseline visit. All patients filled out the Migraine Treatment Optimization Questionnaire (MTOQ) at the start and 3 months after the start of treatment with CGRP-mAbs. During the study period, they completed a headache diary, where they reported the number of migraine days, and acute drug intakes.

**Results:** We included 41 patients, (87.5% women; 72.5% with chronic migraine), with a median age of 46 [interquartile range (IQR) 42.25–55] years. At baseline – i.e., during the 3 months before treatment start –, median MTOQ score was 6 (IQR 3-8), with 30 median monthly migraine days (IQR 20-53) and a median drugs intake equal to 30 doses (IQR 20.25-60). At the 3-month follow-up, median mTOQ scores increased to 10 (IQR 7-13; pvs. baseline), indicating better optimization of treatment during, while median monthly migraine days decreased to 20 (IQR 9-27.5; p=0.002 *vs.* baseline). The median number of acute treatment monthly doses decreased from 30 to 20 (IQR 5.75-29.25, p=0.010 *vs* baseline), during the 3 months of follow-up. Finally, higher scores on the mTOQ negatively correlated with lower use of acute treatments (p=0.028).

**Conclusion:** Our study shows that, 3 months of preventive treatment with CGRP-MoAbs led to a significant increase in mTOQ scores, meaning improved effectiveness of acute treatments, paralleled by decreased monthly migraine days and acute treatment use.

## P176 Personalized Low-Glycemic Nutrition for the Prophylaxis of Migraine: Real World Data from two Prospective Studies using a Digital Therapeutic

### T. Schroeder^1^, V. Lelleck^2^, C. Sina^2^, F. Schulz^1^, G. Kuehn^1^, S. Evers^3^, O. Witt^1^, C. Gaul^4^, D. Thaci^5^, D. Klein^1^, A. Gendolla^6^

#### ^1^Perfood GmbH, R&D, Luebeck, Germany; ^2^University Hospital of Schleswig-Holstein, Institute of Nutritional Medicine, Luebeck, Germany; ^3^University of Muenster, Muenster, Germany; ^4^Headache Center Frankfurt, Frankfurt, Germany; ^5^University of Luebeck, Institute and Comprehensive Center for Inflammation Medicine, Luebeck, Germany; ^6^Medical Practice for Neurology and Pain Therapy Essen, Essen, Germany

##### **Correspondence:** T. Schroeder

Question:

Migraine is a headache disorder associated with a high socioeconomic burden. We developed a digital therapeutic that provides an individualized low-glycemic diet based on continuous glucose measurement. We aimed to find out if this digital therapeutic can serve as a non-pharmacological migraine prophylaxis.

Methods:

We have performed two prospective studies with migraine patients who used our digital therapeutic over a period of 16 weeks. The patients used a headache diary and recorded their migraine-related daily life impairment using the assessment tools HIT-6 and MIDAS for a pre versus post comparison. In addition, continuous glucose data of patients were compared to healthy controls.

Results:

In both studies, patients reported a reduction of headache and migraine days as well as reductions in HIT-6 and MIDAS scores. More specifically, migraine days decreased by 2.40 days (95 % CI [-3.37; -1.42]), HIT-6 improved by 3.17 points (95 % CI [-4.63; -1.70]) and MIDAS by 13.45 points (95 % CI [-22.01; -4.89]). Glucose data suggest that migraine patients have slightly increased mean glucose values as compared to healthy controls but drop into a glucose range that is below one"s individual standard range before a migraine attack.

Conclusion:

In conclusion, our digital therapeutic is a non-pharmacological migraine prophylaxis that induces a therapeutic effect within the range of pharmacological interventions.

**Fig. 1 (abstract P176).**
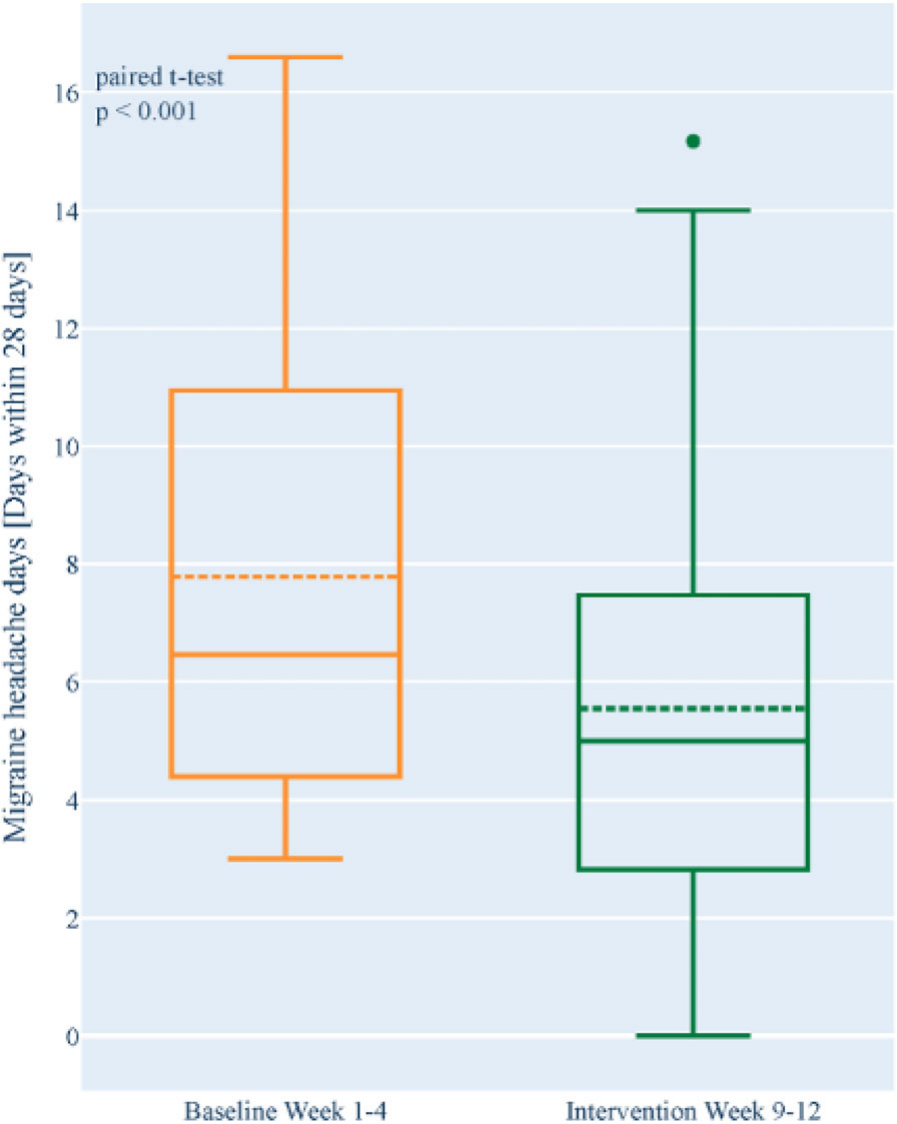
Change in the number of monthly migraine headache days between the four-week baseline phase and the last four weeks of the intervention phase in the complete data set analysis (n=62). Number of headache days was assessed by daily headache diary. Boxplots show 1st quartile, median (solid line), mean (dashed line), and 3rd quartile. Outliers are marked with dots

## P177 Iprazochrome and codeine – unlikely winners in migraine treatment in Poland. Targeting areas for improvement in migraine treatment (results from *Migraine in Poland* - a nationwide cross-sectional survey)

### M. Waliszewska-Prosół^1^, M. Straburzyński^2^, S. Budrewicz^1^, E. Czapińska-Ciepiela^3^, M. Nowaczewska ^4^, A. Gryglas-Dworak^5^, R. B. Lipton^6^

#### ^1^Wroclaw Medical University, Neurology, Wroclaw, Poland; ^2^University of Warmia and Mazury, Family Medicine and Infectious Diseases, Olsztyn, Poland; ^3^Epilepsy and Migraine Treatment Centre, Kraków, Poland; ^4^Nicolaus Copernicus University in Toruń, Ludwik, Rydygier Collegium Medicum, Otolaryngology, Head and Neck Surgery, and Laryngological Oncology, Bydgoszcz, Poland; ^5^Headache Center, Wrocław, Poland; ^6^Albert Einstein College of Medicine, Neurology, New York, United States

##### **Correspondence:** M. Waliszewska-Prosół

**Question**: Defining migraine patterns of care in Poland.

**Methods:** The *Migraine in Poland* study is a nation-wide cross-sectional online survey conducted from August 2021 to June 2022. Participants were recruited through various channels, targeting mostly persons suffering from headaches. Survey protocol included questions allowing for diagnosis of migraine without aura (MwoA). Moreover, the questionnaire assessed consultation rates with a medical professional, as well as the use of abortive or preventive treatment, including non-pharmacological methods.

**Results:** 3225 respondents aged 13 to 80 (mean age 38.94) submitted their answers via online questionnaire (87.10% were women). MwoA diagnosis was confirmed in 1679 (52.73%) of subjects, and 1571 (93.57%) of them consulted their headache with a medical professional in the past.

1553 (92.50%) of MwoA participants declared the current use of some form of treatment. Combination analgesics (especially containing codeine) were the most frequently used (n=991, 59.02%) abortive medications. These were followed by non-steroidal anti-inflammatory drugs and acetaminophen (n=1318, 78.50%). Triptans/ergots were used by 959 (57.12%) respondents. 383 (22.81%) resorted to abortive treatment with frequency indicating medication-overuse.

Prophylactic treatment was at some point used by 599 (35.68%), while 193 (11.49%) were currently on preventive medications. The most frequently prescribed migraine prophylaxis was iprazochrome 151 (8.99%), followed by flunarizine 136 (8.10%) and topiramate 99 (5.90%). 23.28% used nutraceuticals for prevention (most frequently magnesium).

**Conclusions:** The consultation rate for migraine patients in Poland was relatively high, and most of the subjects received the correct diagnosis. However, there is a need for improving standards of care, especially in regard to choice of prophylaxis. There is also a need to raise public awareness of the dangers of codeine-based medications (available OTC in Poland).

## P178 Alternative Indirect Treatment Comparisons for Eptinezumab in Migraine Prevention

### C. Fawsitt^1^, H. Thom^1^, S. Regnier^2^, X. Ying Lee^2^, S. Kymes^3^, L. Vase^4^

#### ^1^Clifton Insight, Bristol, United Kingdom; ^2^Lundbeck, Copenhagen, Denmark; ^3^Lundbeck, Deerfield, IL, United States; ^4^Sciences Aarhus University, Department of Psychology and Behavioural, Aarhus, Denmark

##### **Correspondence:** X. Ying Lee

**OBJECTIVE**: To explore impact of delivery mechanism on 12-week change from baseline in monthly migraine days (MMDs) for indirect treatment comparisons (ITCs) of eptinezumab and other aCGRP mAbs. Intravenous (IV) eptinezumab was investigated for migraine prevention in episodic (EM) and chronic migraine (CM). The comparator aCGRP mAbs are delivered subcutaneously (SC). Influence of delivery mechanism on placebo (PBO) response may bias ITCs.

**METHODS**: Evaluated 3 methodologies: (1) standard Bayesian network meta-analysis (NMA) of phase 3 clinical trials assuming PBOs are identical; (2) network meta-regression (NMR) regressing treatment effect on PBO response; and (3) unanchored simulated treatment comparison (STC) using only active arm data.

**RESULTS**: NMA results favored eptinezumab 300mg over fremanezumab in CM but favored erenumab 140mg and galcanezumab 120mg over eptinezumab 100mg in EM. NMR found all treatments were similar. Unanchored STC in EM favored eptinezumab for most comparisons, while all comparisons in CM favored eptinezumab (one-sided p<0.05). See Tables 1 and 2.

**CONCLUSIONS**: Assumptions about delivery mechanism have a large impact on ITCs. NMA and NMR results are mixed and rarely differentiate treatments. The unanchored STC strongly and consistently favored eptinezumab over other migraine preventive treatments. Consideration of which approach best reflects drug-placebo interactions and real-world outcomes would be beneficial to clinical and formulary decision makers.

**Table 1 (abstract P178).**
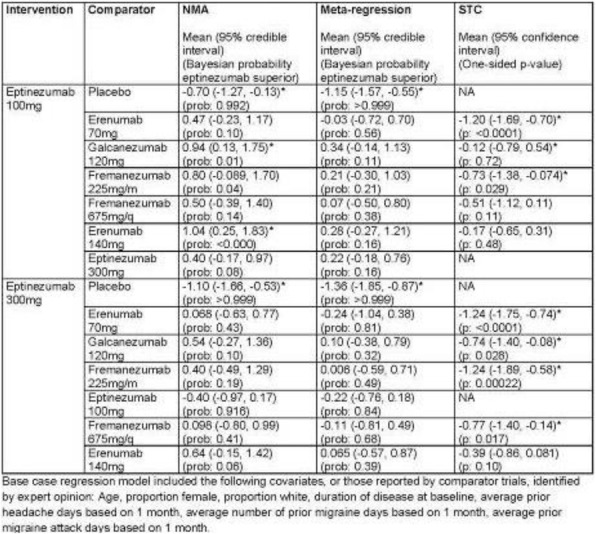
Estimated differences in CFB MMD at 12 weeks in episodic migraine. Values <0 favor eptinezumab. ‘Significance’ at 0.05 threshold should be judged by 95% intervals not crossing 0 and is indicated by an asterisk (*)

**Table 2 (abstract P178).**
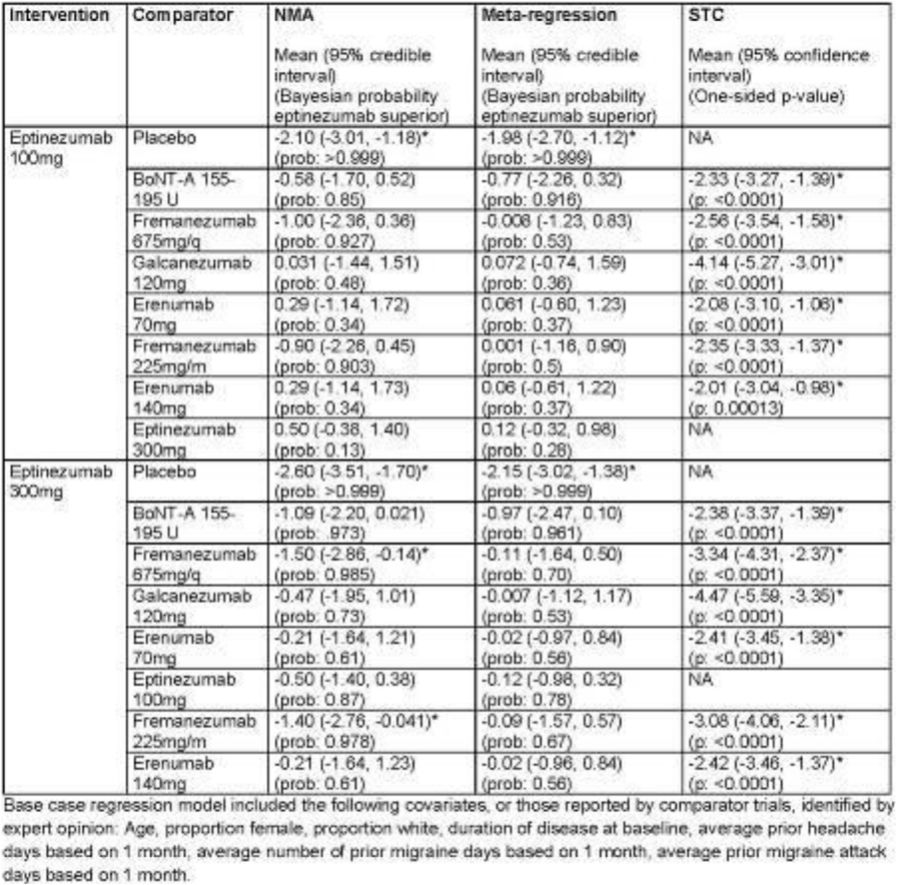
Estimated differences in CFB MMD at 12 weeks in chronic migraine. Values <0 favor eptinezumab. ‘Significance’ at 0.05 threshold should be judged by 95% intervals not crossing 0 and is indicated by an asterisk (*)

## P179 Clinical pattern and response to treatment of predominant posterior migraine

### G. Cabral^1^, C. Gonçalves^1^, A. Caetano^1,2^, R. Pelejão^1^, M. V. Baptista^1,2^

#### ^1^Hospital Egas Moniz, Neurology, Lisbon, Portugal; ^2^Chronic Diseases Research Center (CEDOC) - Nova Medical School, Universidade Nova, Lisbon, Portugal

##### **Correspondence:** G. Cabral

**Background**: There are few data about patients whose migraine headaches predominantly affect the head's back.

**Methods:** Retrospective analysis of patients with the diagnosis of episodic or chronic migraine (according to the ICHD-3 classification) with posterior (occipital and back of neck) onset and predominant location from 2013 to 2020.

**Results**: We identified 60 patients (mean age of headache onset: 27 years old; 81.6% were female) with episodic (78.3%) or chronic migraine (21.7%) with the posterior onset and predominant location. We identified 3 patterns of pain: patients with only posterior localized migraine (25; 41.7%); posterior and temporal or parietal migraine (posterior plus migraine; 16; 26.7%); and posterior onset with holocranial irradiation of pain (19; 31.6%). Regarding the headache characteristics, the most frequent pattern was bilateral or alternating pain (40% each); 63,4% described photophobia or phonophobia plus nausea or vomiting; 28.3% reported aura. The duration of each attack was mostly <48hours (61.8%). Most patients had <5 episodes of migraine attacks/month (51.8%). Only 10 patients (16.7%) reported modification of pain pattern since the onset of migraine. Regarding the treatment, 53.3% of the patients reported failure of at least one drug for the acute phase and 20% had a failure with at least one prophylactic drug. We identified that 18.4% of the patients also had a medication-overuse headache. Forty-three patients (71.7%) started a prophylactic drug. During follow-up, only 14 patients had treatment response (defined as a reduction in ≥50% of monthly headaches).

**Conclusions:** In our cohort, patients with posterior predominant migraine have a young-onset headache, mostly localized pain, a short duration of each attack, and a low frequency of monthly headaches. In the future, it would be important to understand if there are clinical and therapeutic differences between patients with posterior versus anterior predominant migraine.

## P180 Classification of the pain curve and semiology of a migraine crisis

### A. Gonzalez-Martinez^1^, J. Gálvez-Goicurría^2^, J. Pagán^2^, S. Quintas^1^, A. Vieira^1^, J. L. Ayala^3^, A. Sanz^2,4^, M. Sobrado^1^, J. Vivancos^1^, A. B. Gago-Veiga^1^

#### ^1^Hospital Universitario de La Princesa & Instituto de Investigación Sanitaria Princesa (IIS-Princesa), Neurology, Madrid, Spain; ^2^Universidad Politécnica de Madrid, Electronic Engineering Department, Madrid, Spain; ^3^Computer Architecture and Automation Department, Universidad Complutense de Madrid, 16734 Madrid, Comunidad de Madrid, Spain 4CCS: Center for Computational Simulation, Universidad Politécnica de Madrid, Madrid, Spain; ^4^Unidad de Análisis de datos, Instituto de Investigación Sanitaria (IIS-Princesa), Hospital Universitario de la Princesa, Madrid, Spain

##### **Correspondence:** A. Gonzalez-Martinez

**Objective:** Previous studies have identified clinical characteristics of a migraine crisis; however, the evolution of the pain has been little studied. Our objective was to evaluate the semiology of a migraine crisis according to the type of pain curve.

**Methods:** We analyzed the pain curve of patients with episodic migraine according to the current criteria (ICHD-III) included in a prospective real time study using a smartphone application. The follow-up period was one month, the patient marked start/end and evolution of the pain in at least 5 points depending on the intensity. Symptomatic treatment was allowed. At the end of the crisis, the patient completed the characteristics of the episode in the smartphone application. To generate the model K-means and a supervised validation technique using a logistic model tree was used.

**Results:** A total of 344 migraine crisis from 51 patients (mean age 39 years, 90.2% women) were analyzed. Using the maximum pain intensity, time to achieve the maximum pain intensity, the relationship between both parameters and the total duration, all episodes were categorized in four types according to the main characteristics: 1(high intensity), 2(acute onset), 3(prolonged and intense) y 4(low intensity). Univariate analysis found statistically significant differences in the type of curve regarding the presence of nauseas (p<0.001), sonophobia(p<0.001), osmophobia(p<0.03) and the number of simultaneous symptoms (p<0.001); there were also differences in prodromic symptoms such as speech difficulties (p=0.029), dizziness (p=0.015), and postdrom symptoms such as appetite (p=0.029), whim (p=0.031), cognitive (p=0.019) and sleepiness (p=0.013).

**Conclusion:** The evolution of the pain crisis can be successfully categorized in 4 types of curves, which represent different semiology characteristics of a migraine crisis

## P181 Evolution of practice guidelines for the treatment of acute migraine with Paracetamol (Acetaminophen)

### M. Lanteri-Minet^1^, R. Pegahi^2^

#### ^1^CHU de Nice, Nice, France; ^2^UPSA, Medical Affairs, Rueil-Malmaison, France

##### **Correspondence:** M. Lanteri-Minet

**Objective:** Qualitative assessment of evolution over time in different guidelines for paracetamol use in acute migraine

**Methods:** The assessment was performed on 10 published guidelines from 5 scientific societies, American Headache Society (AHS), formerly American Academy of Neurology (AAN), French headache society *("Société Française d'Etudes des Migraines et Céphalées"* - SFEMC), Canadian Headache Society (CHS), European Federation of Neurological Societies (EFNS), and European Headache Federation (EHF).

**Results:** The comparison between earlier (1997-2014) and current (2009-2021) guidelines showed an evolution in the level of recommendations for paracetamol use in three of five scientific societies. There was a shift from no recommendation or recommendation only for mild attacks to recommendation for mild to moderate attacks without restriction in updated guidelines. Four of the five scientific societies (AHS, SFEMC, CHS, EFNS) now recommend Paracetamol for mild to moderate attacks. The 5th scientific society (EHF) recommends paracetamol when NSAIDs are contraindicated.

**Conclusion:** This qualitative analysis emphasizes evolutions in practice guidelines over time in different scientific societies regarding the place of paracetamol in acute migraine. There was a positive evolution in paracetamol recommendations in the latest guidelines. Most guidelines now recommend it for mild to moderate pain.

## P182 Craniocervical Flexion Test in patients with migraine: discriminatory validity and accuracy

### A. Rodrigues^1^, L. Lima Florencio^2^, J. Martins^1^, M. Mendes Bragatto Scornavacca^1^, C. Fernández-de-las-Peñas^2^, F. Dach^3^, D. Bevilaqua Grossi^1^

#### ^1^University of São Paulo, Health Sciences, Ribeirão Preto, Brazil; ^2^Universidad Rey Juan Carlos, Madrid, Spain; ^3^University of São Paulo, Ribeirão Preto, Brazil

##### **Correspondence:** A. Rodrigues

**Objectives:** To evaluate the discriminatory validity and provide a clinical cut-off of the craniocervical flexion test (CCFT) in migraineurs stratified by the report of neck pain, headache-related disability and neck disability.

**Methods**: This study enrols 50 women without headache and 102 women with migraine recruited by convenience from a local tertiary care setting. The standard reference for migraine diagnosis was the International Classification of Headache Disorders. All volunteers underwent the CCFT and the patients with migraine patients answered the Migraine Disability Assessment (MIDAS) and Neck Disability Index (NDI) questionnaires. Discriminatory validity was verified by groups comparison. The clinical cut-off was obtained and classified according to the diagnostic accuracy of the CCFT.

**Results:** The CCFT presented discriminative validity for comparing control (median = 28, IRQ = 6) with migraine (median = 26, IRQ = 4, p = 0.01) and migraine with neck pain (median = 26, IRQ = 4, p = 0.01), but not among the migraine subtypes with disability by migraine or neck pain related disability on the MIDAS and NDI. The diagnostic accuracies were classified between poor and not discriminating with the area under the receiver operating characteristic curve ranging from 57% to 69%, and non-acceptable values of sensitivity, specificity and positive and negative likelihood ratios.

**Conclusion:** The CCFT is able to discriminate asymptomatic controls from migraine patients with and without neck pain. However, it cannot discriminate patients with migraine according to their pain-related disability. In addition, the CCFT does not offer a cut-off value adequate to identify the deficit of function of the deep flexor muscles in migraine patients.

## P183 Prevalence, disease profile, treatment patterns and burden of migraine in India: An internet-based survey

### D. Chowdhury^1^, A. Krishnan^2^, A. Duggal^1^, D. Datta^1^, A. Mundra^1^, V. Deorari^1^, A. Tomar^1^, A. Koul^1^

#### ^1^GB Pant Institute of Post Graduate Medical Education and Research, Neurology, Delhi, India; ^2^All India Institute of Medical Sciences, Community Medicine, Delhi, India

##### **Correspondence:** D. Chowdhury

**Objective:** To study the prevalence, disease profile, and disease burden of migraine patients in India using an internet-based survey.

**Methods:** This is the second part of an internet-based survey using a structured questionnaire conducted from 27th April to 31st July 2020. The first part assessed the impact of the COVID-19 lockdown on Indian migraine patients (1). Persons aged 18 years and above were invited to participate. Previously known migraine patients being treated by physicians or those fulfilling 2 out of 3 criteria (limitation of activities for a day or more, associated nausea or vomiting, and photophobia or phonophobia) were diagnosed as migraine patients.

**Results:** 5694 persons registered and 4078 completed the full survey. Migraine was diagnosed in 984 (24.1%) participants [(635 females); mean age 35.32 ±11.16]. The mean migraine days per month was 7.24±5.84 (median=5; range=0.5-30). 109 (11.1%) had ≥ 15 headache days per month. Moderate to severe pain was reported by 923 (93.8%). Acute drug treatment was used by 877(89.1%) and 10.9% used non-pharmacological measures. Preventive medications were used by 569 (57.8%). Of patients who required preventive medications (≥ 4 attacks per month), 18.5% did not receive them. Headache yesterday was reported by 571 (58%). 52% were willing to spend from INR 1000 to 10000 (13.6 USD to 136 USD) per month to cure their headaches completely.

**Conclusion:** Approximately 1 out of 4 persons suffers from migraine in India. More than half of the migraine patients have a significant burden as reflected by their willingness to pay for its cure.

Reference:

1. Chowdhury D, Krishnan A, Duggal A, Datta D, Mundra A, Deorari V, Tomar A, Koul A. An Internet-based study on the impact of COVID-19 pandemic-related lockdown on migraine in India. Acta Neurol Scand. 2021;144(6):706-716.

## P184 Use of Carbamazepine in Patients with Migraine-Comorbid Epilepsy in The Uzbek Population

### M. Mavlanov, F. Saidvaliyev

#### Tashkent Medical Academy, Neurology, Tashkent, Uzbekistan

##### **Correspondence:** M. Mavlanov

**Purpose**: Choice of the most effective anticonvulsant in patients with migraine comorbid epilepsy (MCE).

**Materials and methods:** In the period from 2018 to 2021, 18 patients with a diagnosis of migraine comorbid epilepsy applied to the department of the consultative polyclinic of the Tashkent Medical Academy. The patients underwent examination, ID-Migraine test, VAS, MIDAS, IPSS-QoL and EEG (again in a month), MRI of the brain.

**Results:** The diagnosis was made according to the ID-Migraine criteria and the International Headache Classification 3-Revision (ICHD-3). Women 12 (66.7%), men 6 (33.3%), mean age 27.4±0.4. Neurologic examination without features. The mean VAS was 6.9, which indicates a pronounced intensity of headaches. According to the MIDAS scale, the average value is 15.6 - in the main number of patients, headache makes everyday activity difficult. The quality of life was reduced according to the IPSS-QoL test - global index = 39.9. In 14 (77.7%) cases, convulsions had a primary generalized character, in 4 (22.3%) patients, partial convulsions with transitions to generalization were determined. On the EEG foci were determined. Patients were divided into equal 2 groups, while the difference between the VAS, MIDAS and IPSS-QoL scores of the 2 groups was not statistically significant. In order to prevent migraine and relieve seizures, the first group of patients was given Carbamazepine 400-600 mg/day, the patients of the second group received Valproic acid 1000 mg/day. Upon re-examination, in all patients, the frequency and intensity of headaches decreased, daily activity and quality of life improved with a predominance in the first group. The EEG also showed positive dynamics with prevalence in the first group.

**Conclusions:** Patients with MCE Carbamazepine gave a better result compared to valproic acid. This conclusion is not a criterion for choosing the drug and requires further research.

## P186 Treatment of Migraine in children and adolescents

### J. Genizi

#### BNAI ZION, PEDIATRICS, Haifa, Israel

Migraine headaches in children may cause attacks that require abortive treatment. This study evaluated the incidence and efficacy of medications used for relieving migraine headache attacks in the pediatric population in Israel. Children 6–18 years of age who were diagnosed in our pediatric neurology clinic as having migraine headaches were enrolled into the study. Children and their parents recorded the children response to abortive treatment during consecutive migraine attacks. Fifty children, with 116 migraine attacks, were included in the study (30 females; mean age 12; range 6–18). Forty-seven (94%) reported on abortive treatment on the first migraine attack, 43 (86%) on a second migraine attack and 26 (52%) on a third migraine attack. During the first recorded migraine attack, 41 children (87.5%) reported taking only one type of medication for each headache episode, mainly ibuprofen or acetaminophen; less than a quarter used dipyrone (metamizol). Overall the improvement rate after two hours was 65.4% ± 27 for ibuprofen, 59.8 ± 35.3 for acetaminophen and 50.9 ± 27.4 for dipyrone without statistical difference. However, in the first recorded headache episode, males had a significantly better response to acetaminophen, compared to ibuprofen (95% ± 28 vs 75 ± 20). In conclusion, Children with migraine in Israel mainly use a single medication for each headache episode. Ibuprofen is the most commonly used abortive treatment; however, acetaminophen was associated with a better response among some of our patients.

## P187 Vitamins and migraine? Is it a matter? The possible effects and suggested pathophysiology

### M. Togha

#### Tehran University of Medical Sciences, Headache Department, Tehran, Iran

Objective: This review aims to look at the possible effects of vitamins on migraine in terms of pathophysiology and clinical presentation. In the last years, there are several articles indicative of the positive role of food supplements in migraine.

Methods: Our research team has had some studies on the effects of vitamins on migraine. Here we are going to present the result of our studies on Vitamin B groups and Vitamin D.

Result: It seems that the positive effect of Vitamin D3 on migraine is through suppressing neuroinflammation, such as its effect on CGRP, TGF-β, IL-17 levels and Th17/Treg-related cytokines balance. Supplementing migraineurs with vitamin D reduced the frequency of attacks, according to our study. In another study, we found that CGRP level was significantly lower following vitamin D supplementation than patients in the placebo arm (*P*-value = 0.022). Energy-deficit syndrome with mitochondrial dysfunction should be considered an upstream disorder in migraine pathophysiology. A majority of vitamin B group including Thiamine, Riboflavin, Niacin, and Pantothenic Acid are involved in metabolic and energy production pathways. Besides vitamin B12 is thought to be involved in important pathways that seem to be related to the pathogenesis of migraine including scavenging against NO and prevention of hyperhomocysteinemia. The findings of our study on migraine patients suggest that participants with lower vitamin B12 and higher MMA levels that are considered to lower functional activity of B12 had higher odds of migraine. Our team also found that supplementation with folic acid, vitamin B6, vitamin B12, and vitamin B1 significantly reduced headache frequency and intensity.

Conclusion: It seems that deficiency of vitamin D3 and vitamin B group might lead to initiation or aggravation of migraine and supplementation of these vitamins could improve it possibly through their effects in the neuroinflammation pathway or/and energy generation in the mitochondria.

## P188 Vestibular Migraine: Management differences between General Neurology and Neuro-Otology

### M. D. Villar-Martinez^1,2^, D. Moreno-Ajona^1^, A. Bronstein^2^, P. J. Goadsby^1^

#### ^1^King´s College London, London, United Kingdom; ^2^Imperial College London, Neuro-Otology, London, United Kingdom

##### **Correspondence:** M. D. Villar-Martinez

**Question:** Patients with a diagnosis of vestibular migraine (VM) are frequently referred to Neurology. Here we assessed if the management received is similar comparing a general with a specialised clinic.

**Methods:** We audited clinical notes of patients seen in Neuro-Otology clinics (NO) and General Neurology clinics (GN) from the same tertiary hospital, from January 2021 to January 2022 diagnosed with VM. Demographics, treatment and outcomes were analysed with Jamovi.

**Results:** Of VM patients (*n* = 85), 22 were seen in GN, and 63 in NO. Sixty-seven (77%) were females, and sex distribution was similar. Age (median, IQR) was 48.7, (38.8-56.6) in the NO group, and 35.8 (27.3-50.8) in the GN (*P*=0.031). Preventives were advised in 77% in NO in comparison with 59% in GN (*P*=0.107) and there were no differences in the choice of preventive between groups. Most patients were on tricyclics (38.8%) followed by beta-blockers (15.3%), supplements (14.1%), candesartan (11.8%) and antiepileptics (3.5%). In the GN group, 2 patients were on pizotifen, and in the NO, one patient was on sertraline, prochlorperazine, botulinum toxin A and perimenstrual naproxen, respectively. In the NO group, 17.5% of patients were discharged, in comparison with 54.5% in the GN (*P*<0.001).

**Conclusion:** VM patients seen in NO are older, require more preventive medication and follow-up than those seen in GN. This could be due to a more complex clinical presentation, delay in diagnosis or failure of previous management. Patients would possibly benefit from an earlier referral to a clinic with expertise in VM.

## P189 Population-based prevalence of cranial autonomic symptoms in migraine and proposed diagnostic appendix criteria

### C. G. Christensen^1^, T. R. Techlo^1^, L. J. A. Kogelman^1^, L. W. Thørner^2^, J. Nissen^2^, E. Sørensen^2^, J. Olesen^1^, T. F. Hansen^1^, M. A. Chalmer^1^

#### ^1^Rigshospitalet, Danish Headache Center, Neurological Department, Glostrup, Denmark; ^2^Rigshospitalet, Department of Clinical Immunology, Copenhagen, Denmark

##### **Correspondence:** M. A. Chalmer

Background: Migraine with cranial autonomic symptoms (MwCAS) is well described in the literature, but its prevalence in previous studies varies enormously. A precise estimate of the prevalence in a population-based material is important because MwCAS might represent an endophenotype, in which genetic and pathophysiological features differ from those without cranial autonomic features. The aim of the present study was hence to estimate the prevalence in a big population-based sample using both questionnaire-based diagnosis (N=12,620) and interview-based diagnosis (N=302). We validate questionnaire-based diagnosis of MwCAS and develop the first diagnostic criteria for future research of this possible endophenotype.

Methods: 62,677 participants answered a diagnostic questionnaire of whom 12,620 had migraine. The diagnostic migraine questionnaire included questions about cranial autonomic symptoms. Validation was performed by a follow-up semi-structured interview of 302 participants with migraine.

Results: The questionnaire-based prevalences of one, respectively two cranial autonomic symptoms were 57% and 31%. The semi-structured interview-based prevalences of one, respectively two symptoms were 44% and 22%. The most common symptoms were facial/forehead sweating (39%) and lacrimation (24%). The specificity of the questionnaire was 80% and the sensitivity was 68%. Correlation analysis showed a weak correlation between symptoms ranging from 0.07 – 0.41, and no clear clustering of symptoms was detected. We suggest the first diagnostic appendix criteria for genetic and epidemiological studies and tighter criteria for clinical and pathophysiological studies. We encourage further studies of severity and consistency of symptoms.

Conclusion: MwCAS is prevalent in the general population. Suggested diagnostic appendix criteria are important for future studies of this possible migraine endophenotype.

## P190 Prediction of a migraine crisis in real-time using a wearable device

### A. Gonzalez-Martinez^1^, J. Gálvez-Goicurría^2^, J. Pagán^2^, S. Quintas^1^, A. Vieira^1^, C. A. Ramiro^2^, M. Sobrado^1^, J. L. Ayala^3^, J. Vivancos^1^, A. B. Gago-Veiga^1^

#### ^1^Hospital Universitario de La Princesa & Instituto de Investigación Sanitaria Princesa (IIS-Princesa), Neurology, Madrid, Spain; ^2^Universidad Politécnica de Madrid, Electronic Engineering Department, Madrid, Spain; ^3^Universidad Complutense de Madrid, Computer Architecture and Automation Department, Madrid, Spain

##### **Correspondence:** A. Gonzalez-Martinez

**Objective:** Previous research carried out in our group demonstrated that patients with migraine exhibit changes in hemodynamic variables, suggesting a dysregulation of the autonomic nervous system (ANS). We aim to evaluate whether hemodynamic variables measured by cutting-edge wearable devices can predict migraine pain onset.

**Methods:** We performed a prospective study including patients with migraine in which we recorded real-time hemodynamic signals, including skin distal temperature (T), heart rate (HR) and electrodermal activity (EDA), obtained from a 24-hours wrist wearable device. Personalized prediction models were generated using the Artificial Recurrent Neural Networks long short-term memory (LSTM) to compute on a one-minute basis if the pain was going to appear in the next 120 minutes. Data were balanced in time periods of pain-no pain to train the models.

**Results:** A total of 8 patients with episodic migraine were included in the study. Most patients were women 7/8 (87.5%) and median age was 46 (IQR:34-48) years. Median duration of migraine was 27 (IQR: 18-35) years. The algorithm was able to predict migraine attacks with 95% sensitivity in the whole sample. The model predicted 23/24 (95%) of the pain attacks. In 7/8 (85.7%) of patients" migraine attacks were predicted, based on the 60-minute model, with no false negatives among them.

**Conclusions:** This study confirms that it is possible to predict a migraine attack using hemodynamic variables recorded by an easy-to-use wrist wearable. This research opens new possibilities to study the effect of early treatment on the evolution of the pain in a migraine crisis.

## P191 Utility of machine-learning based models to predict 30%, 50% and 75% response to anti-CGRP response in patients with migraine: a multicenter Spanish study

### A. Gonzalez-Martinez^1^, J. Pagán^2^, A. Sanz^3^, D. García-Azorín^4^, J. Rodríguez-Vico^5^, A. Jaimes^5^, A. Gómez García^5^, J. Díaz de Terán^6^, M. Sastre Real^6^, N. González-García^7^, J. Porta-Etessam^7^, S. Quintas^1^, R. Belascoaín^1^, J. Casas Limón^8^, C. Calle^9^, G. Latorre^9^, Á. Sierra-Mencía^10^, Á. L. Guerrero Peral^10^, C. Trevino-Peinado^11^, A. B. Gago-Veiga^1^

#### ^1^Hospital Universitario de La Princesa & Instituto de Investigación Sanitaria Princesa (IIS-Princesa), Neurology, Madrid, Spain; ^2^Universidad Politécnica de Madrid, Electronic Engineering Department, Madrid, Spain; ^3^Unidad de Análisis de datos, Instituto de Investigación Sanitaria (IIS-Princesa), Hospital Universitario de la Princesa, Madrid, Spain; ^4^University Hospital of Valladolid, Neurology, Madrid, Spain; ^5^Fundación Jiménez Díaz, Neurology, Madrid, Spain; ^6^University Hospital La Paz, Neurology, Madrid, Spain; ^7^Hospital Universitario Clínico San Carlos, Neurology, Madrid, Spain; ^8^Hospital Universitario Fundación de Alcorcón, Neurology, Alcorcón, Spain; ^9^Hospital Universitario de Fuenlabrada, Neurology, Fuenlabrada, Spain; ^10^University Hospital of Valladolid, Neurology, Valladolid, Spain; ^11^Hospital Severo Ochoa, Neurology, Leganés, Spain

##### **Correspondence:** A. Gonzalez-Martinez

**Objective:** To date, several variables have been associated with anti-CGRP receptor or ligand-antibody response with disparate results. Our objective is to determine whether machine learning (ML)-based models can predict 6, 9 and 12 months response to anti-CGRP receptor or ligand therapies among migraine patients.

**Methods:** We performed a multicenter analysis of a prospectively collected data cohort of patients with migraine from 8 tertiary hospitals receiving anti-CGRP therapies. Demographic and clinical variables were collected. Response rate defined in the 30% to 50% range-or at least 30%-, in the 50% to 75% range-or at least 50%-, and response rate over 75% reduction in the number of headache days per month at 6, 9 and 12 months. A sequential forward feature selector was used for variable selection and ML-based predictive model response to anti-CGRP therapies at 6, 9 and 12 months, with models" accuracy not less than 70%, were generated.

**Results:** A total of 712 patients were included,93% women, aged 48 years (SD=11.7). Eighty-three percent had chronic migraine. ML models using headache days/month, migraine days/month and HIT-6 variables yielded predictions with a F1 score range of 0.70-0.97 and AUC (area under the receiver operating curve) score range of 0.87-0.98. SHAP (SHapley Additive exPlanations) summary plots and dependence plots were generated to evaluate the relevance of the factors associated with the prediction of the above-mentioned response rates.

**Conclusions:** According to our study, ML models can predict anti-CGRP response at 6, 9 and 12 months using commonly collected clinical variables. This study provides a useful predictive tool to be used in a real-world setting.

## P192 A prospective observational real-world evidence (RWE) study to describe the impact of OTC headache treatments on headache intensity and the association of headache intensity on functional and cognitive parameters

### L. Constantin^1^, C. Amand-Bourdon^1^, V. Polivka^1^, M. K. Margolis^2^, C. Colby^2^, A. Stewart^3^, P. J. Goadsby^4,5^

#### ^1^Sanofi, Gentilly, France; ^2^Evidera PPD, part of Thermo Fisher Scientific, Wilmington, United States; ^3^Sanofi, Cambridge, United States; ^4^NIHR King’s Clinical Research Facility, King’s College, London, United Kingdom; ^5^University of California, Los Angeles, United States

##### **Correspondence:** L. Constantin

**Objective:** Headache can negatively impact cognitive parameters such as concentration, attention, or the ability to focus. This prospective observational real-world evidence study was conducted in Germany and Japan to describe the impact of over the counter (OTC) headache treatments on headache pain intensity and to assess the association of pain intensity with functional and cognitive impact.

**Methods:** Panel members who used Sanofi OTC headache treatments were invited to participate. Only attacks treated with that product were assessed. The primary endpoint was change in pain intensity assessed via 11-point Numeric Rating Scale (NRS; 0=no pain to 10=worst pain imaginable) from baseline to 2 hours after treatment, using the first headache episode treated. Secondary endpoints were changes from baseline to 2 hours post-treatment in concentration, attention, ability to focus, coordination, productivity, clear thinking, and energy – all assessed via 11-point NRS (0=no impact and 10=severe impact) on all headache episodes treated.

**Results:** In Germany and Japan, respectively, 426 and 452 participants were enrolled; of which 293 and 326 reported a headache and among them 202 and 196 used OTC treatment only. (Mean±SD age was 42.8±12.0 and 41.9±10.2 years; 52% and 72% were female). Mean NRS pain score 2 hours post-treatment decreased from baseline by 3.4 points (CI 95% 3.1 to 3.7) in Germany and 3.0 points (2.7 to 3.3) in Japan, both *p*<0.0001. All secondary endpoints with functional and cognitive parameters were improved with this pain relief (*p*<0.0001).

**Conclusion:** Participants in both countries reported significant reductions in pain 2 hours post OTC treatment, which also correlated with reduction in impact of all cognitive and functional parameters evaluated. Cognitive and functional impairment due to headache impacts patients´ quality of life and should be considered in addition to pain reduction in optimizing headache treatment.

## P193 Searching for the predictors of response to BoNT-A in migraine using a machine learning approach

### D. Martinelli^1,2^, M. M. Pocora^1,2^, R. De Icco^1,2^, M. Allena^1^, G. Sances^1^, G. Castellazzi^1^, C. Tassorelli^1,2^

#### ^1^IRCCS Fondazione Mondino, Headache Science and Rehabilitation Center, Pavia, Italy; ^2^Pavia University, Brain and Behavioral Science Department, Pavia, Italy

##### **Correspondence:** D. Martinelli

OnabotulinumtoxinA (BoNT-A) reduces the frequency of migraine but the clinical profile of those patients who might benefit from it is still missing.

**Objective:** In this single-centre, real-life study, we applied machine learning (ML) algorithms to a database of patients who underwent treatment with BoNT-A to identify baseline clinical characteristics capable to predict response to treatment.

**Methods**: We collected baseline demographic and clinical data of consecutive patients who started BoNT-A at IRCCS Mondino Foundation from January 2017 to March 2022. All patients had a diagnosis of chronic migraine or high-frequency episodic migraine and underwent at least one treatment cycle with BoNT-A according to the PREEMPT paradigm. Patients were primarily classified according to the monthly migraine days reduction in the 12-week period after the fourth BoNT-A treatment, as compared to a 28-day baseline period. Patients who early terminated the treatments after 1 or 2 consecutive administrations without any effect were profiled as well. Other classifications were obtained using secondary endpoints like migraine disability assessment test (MIDAS) and abortive drug use reduction.

Collected data were used as input features to run different kinds of supervised and unsupervised ML algorithms.

**Results**: Of the 212 patients included in the evaluation, 35 qualified as responders to BoNT-A administration and 91 as non-responders. Not a single, or panel, of anamnestic characteristics, proved capable to discriminate responders from non-responders. All ML models coherently reached good accuracy but underperformed and lacked specificity.

**Conclusions:** Overall, ML findings suggest that routine anamnestic features acquired in real-life settings cannot accurately predict the patients that will benefit from BoNT-A treatment. A deeper phenotyping of patients" features, possibly combined with multimodal parameters, is probably required to identify features predictive of response to BONT-A.

**Fig. 1 (abstract P193).**
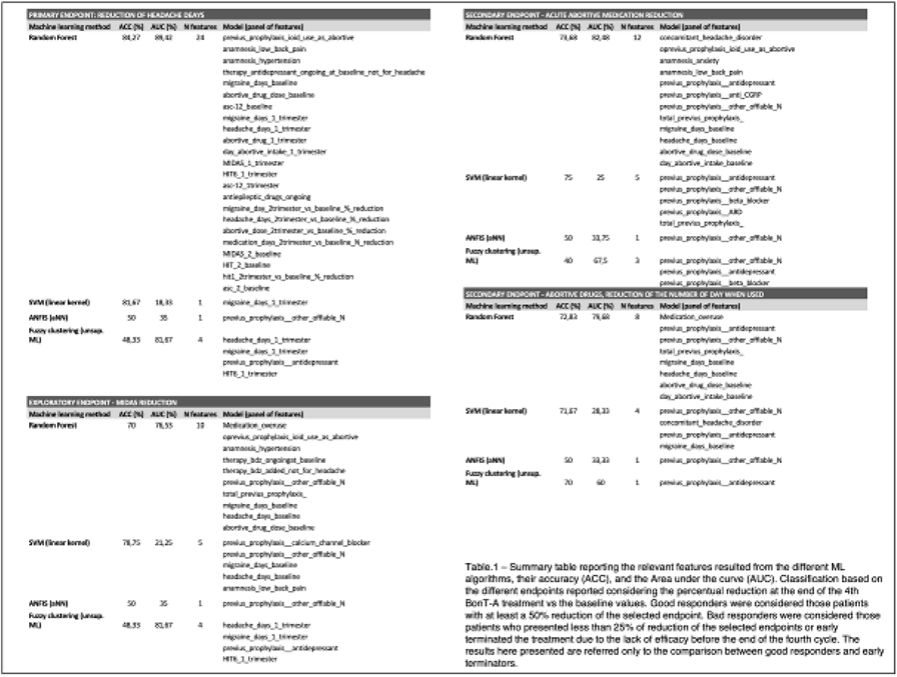
See text for description.

## P194 An Exploratory Study Evaluating the Thirty Medications Most Commonly Associated with Headaches in the FDA Adverse Event Reporting System

### B. Musialowicz, B. Kamitaki, P. Zhang

#### Rutgers Robert Wood Johnson Medical School, Neurology, New Brunswick, NJ, United States

##### **Correspondence:** P. Zhang

Background:

Headache secondary to medication use is a well-known entity. However, which medications are most likely to cause headaches on a global scale is unknown.

Objective:

This project seeks to identify the top thirty drugs most commonly associated with headaches in the Food and Drug Administration Adverse Event Reporting System (FAERS), as well as their respective reporting odds ratio (ROR).

Methods:

We extracted case identifiers (IDs), adverse events (AEs), and attributed medications for entries in the FAERS database from July 1, 2018, to March 31, 2020. Entries were split into two files based on whether each contained the word "headache(s)." Non-medication words are excluded. Medications were ranked by frequency of occurrence in the database, and their ROR values were calculated.

Results:

We extracted 2,673,081 entries, of which 86,086 contain the word "headache(s)." The thirty most frequently appearing medication were then ranked by ROR value and a 95% confidence interval. Immunosuppressants, antivirals, as well as pulmonary hypertension medication classes were most commonly associated with headache.

Conclusion:

Our study offers a potential list of the medication classes commonly associated with iatrogenic headaches.

## P195 Machine prescription for chronic migraine

### A. Stubberud^1^, R. Gray^2^, E. Tronvik^1^, M. Matharu^3^, P. Nachev^2^

#### ^1^Norwegian University of Science and Technology (NTNU), Department of Neuromedicine and Movement Science, Trondheim, Norway; ^2^UCL Queen Square Institute of Neurology, High Dimensional Neurology Group, London, United Kingdom; ^3^University College London, Headache and Facial Pain Group, London, United Kingdom

##### **Correspondence:** E. Tronvik

Objectives: To quantify the individualized treatment effects of major categories of prophylactic treatment in chronic migraine; and quantify the time-to-response of machine prescription and compare it against established heuristic treatment policies.

Methods: Examining a richly phenotyped cohort of 1446 consecutive unselected patients with chronic migraine, we use causal multitask Gaussian process models to estimate individual treatment effects across 10 classes of preventatives. Such modelling enables us to quantify the accessibility of heterogeneous responsiveness to high-dimensional modelling, to infer the likely scale of the underlying causal diversity. We calculate the treatment effects in the overall population, and the conditional treatment effects among those modelled to respond and compare the true response rates between these two groups. Identifying a difference in response rates between the groups supports a diversity of causal mechanisms. Moreover, we propose a data-driven machine prescription policy, estimating the time-to response when sequentially trialling preventatives by individualized treatment effects and comparing it to expert guideline sequences.

Results: We identify significantly higher true response rates among individuals modelled to respond, compared with the overall population (mean difference of 0.034; 95% CI 0.003–0.065; P = 0.033), supporting significant heterogeneity of responsiveness and diverse causal mechanisms. The machine prescription policy yields an estimated 35% reduction in time-to-response (3.750 months; 95% CI 3.507–3.993; P < 0.0001) compared with expert guidelines, with no substantive increase in expense per patient.

Conclusion: We conclude that the highly distributed mode of causation in chronic migraine necessitates high-dimensional modelling for optimal management. Machine prescription should be considered an essential clinical decision-support tool in the future management of chronic migraine.

## P196 Implementing a digital treatment solution for headache patients – study protocol for developing a methodology for interventional studies.

### T. Niiberg-Pikksööt^1,2^, K. Laas^3^, A. Aluoja^4^, M. Braschinsky^5,1,2^

#### ^1^Tartu University Hospital, Headache Clinic, Tartu, Estonia; ^2^Migrevention OÜ, Tallinn, Estonia; ^3^University of Tartu, Institute of Psychology, Tartu, Estonia; ^4^Tartu University Hospital, Psychiatry Clinic, Tartu, Estonia; ^5^Tartu University Hospital, Neurology, Tartu, Estonia

##### **Correspondence:** T. Niiberg-Pikksööt

**Background**. The most effective treatment of migraine is interdisciplinary. However it has very limited availability. This study is the first interventional study to test the operation and effectiveness of digital interventions compared to the usual interdisciplinary treatment (treatment standard).

**Question**. Is digitally mediated treatment as effective as the conventional one and does it allow to treat more patients while saving the workload of professionals?

**Methods**. The study is approved by the Research Ethics Committee of the University of Tartu and registered at ClinicalTrials.gov. This is a multi-centre, open-label, prospective, randomized study. According to power analysis, 600 migraine patients are randomized to receive the interdisciplinary intervention for migraine in parallel groups: the study group (digital treatment group) and the control group (standard interdisciplinary treatment group or treatment standard group). Interventions are held by same specialists in 3 different areas (headache nurse counselling, cognitive-behavioural therapy, physiotherapy). Pharmacological treatment is prescribed by patient's treating neurologist, who is blinded of patients' allocation to groups.

**Outcomes**. The primary outcome measure is a reduction of headache days after three months of the interdisciplinary intervention program compared to baseline. Secondary outcome measures will measure changes in: number of headache days six and nine months after the intervention (follow-up); consumption of migraine acute medications; pain intensity; depression and anxiety; pain acceptance; health relates quality of life; sick-leaves; impact on the burden of migraine.

**Discussion**. The main importance of this innovative study design and approach is the possibility to provide evidence about effective digitized migraine treatment to much more patients with a smaller workload. This study is laying the foundation for planning further research in the area and for clinical implementations.

## P198 @TrialMigraine: A Daily Migraine Clinical Trials Tracker

### P. Zhang^1,2^

#### ^1^Rutgers Robert Wood Johnson Medical School, Neurology, New Brunswick, NJ, United States; ^2^Cymbeline LLC, Dayton, NJ, United States

Background

Keeping abreast of on-going headache researches is an important task for both clinicians, researchers, and patient advocates. However, this task can be time consuming especially as on-going clinical trials are un-finished and therefore unpublished. Using clinicaltrials.gov's application programming interface, this project builds a Twitter robot, @TrialMigraine, which alerts subscribers of newly registered clinical trials on a daily basis

Methods

Using Haskell and Clojure, two functional programming languages, clinicaltrial.gov is accessed and searched on a daily basis for any clinical trials containing the word "headache". Resultant clinical trials are downloaded and recorded on a daily basis. The title of new clinical trials registered are reported on a daily basis on @TrialMigraine.

Results

@TrialMigraine has been implemented on a RaspberryPi unit since May 2020. A total of 1181 clinical trials has been reported from its inception until January 2022. Since @TrialMigraine is a Twitter account, it is available to the public without cost.

Conclusion

Daily reporting of on-going clinical trials through social media robot is possible and may offer various stakeholders a readily accessible in tracking the latest developments in headache medicine.

## P199 No machine learning required: a number theory diagnostic methods for primary headache disorders

### P. Zhang^1*,*2^

#### ^1^Rutgers Robert Wood Johnson Medical School, Neurology, New Brunswick, NJ, United States; ^2^Cymbeline LLC, Dayton, NJ, United States

Background

Significant interests have been generated in the past years in applying machine learning techniques to headaches diagnosis. Here we propose that headaches disorder diagnosis using ICHD3 can be translated to a prime factorization problem.

Methods

We assigned each statement of headache characteristic in the ICHD3 criteria to a unique prime number. (Table 1). Each ICHD3 diagnosis criterion can be decomposed to logic statements, which then in turn can be used to generate a list of composite numbers (Table 2) through the following rules: 1) If the logic AND is required, we multiply the two numbers. 2) If the logic OR is required, we document both numbers in a list.

To use the algorithm, the user answers yes or no to each of the headache characteristics in Table 1; then the user raises the corresponding prime numbers to 1st power if the answer is yes and 0th power if the answer is no. These numbers are then multiplied together; if this number can be divided by a number in Table 2 without remainder, then the corresponding ICHD3 diagnosis is diagnostic.

For example, a patient with greater than 5 episodes (19) of pulsating (71), unilateral headaches (73) lasting 5 hours each (17) with nausea (37) but no photo/phonophobia (43*47) can be represented by a score of 19*17*37*43*47*71*73 =

125184848693. This number can be divided by 61942033 without remainder. Since 61942033 corresponds to migraine without aura (Table 2), the diagnosis is therefore migraine without aura.

Results

We applied the above method to migraine without aura as well as infrequent, frequent, and chronic tension type headaches as a pilot study. For migraine without aura, infrequent, frequent, and chronic tension type headaches, 22 composite numbers represent each of the diagnostic criteria.

Conclusion

Applying headache diagnosis according to the ICHD3 criteria can be translated to a prime factorization problem. Our method represents a bridge between number theory and clinical medicine.

**Table 1 (abstract P199).**
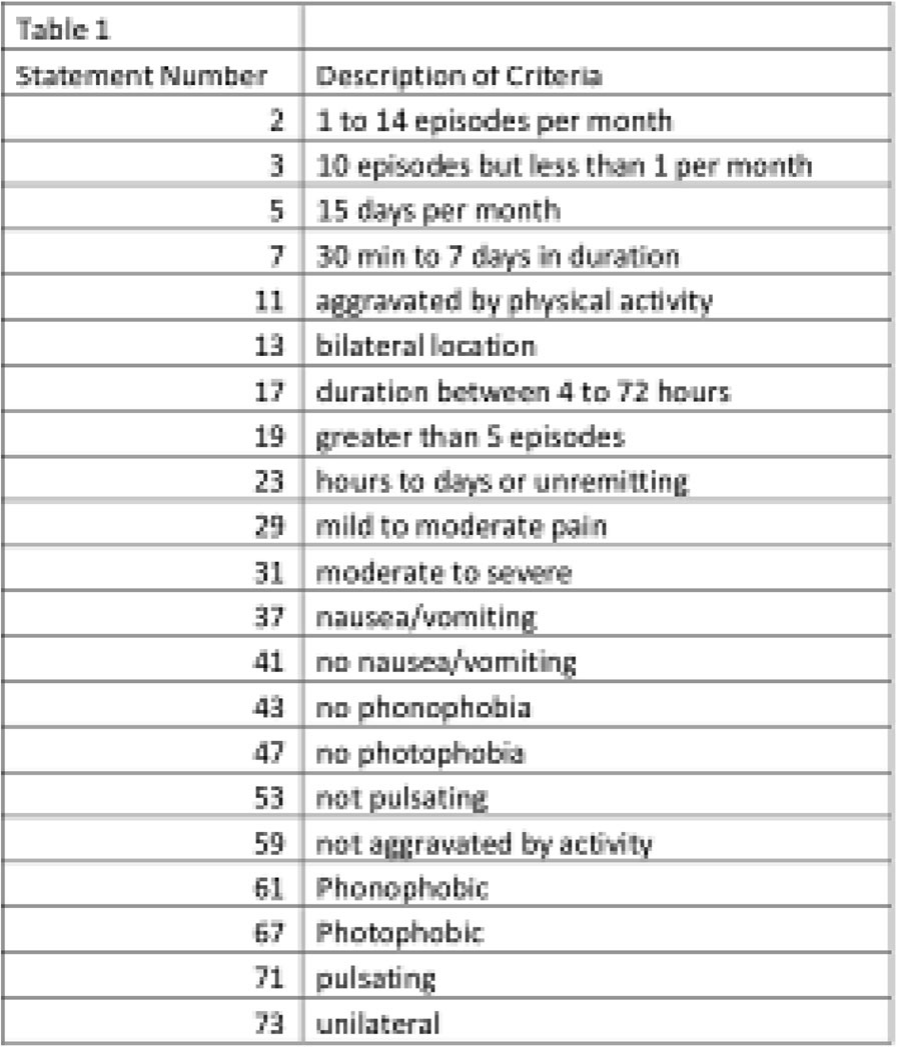
See text for description.

**Table 2 (abstract P199).**
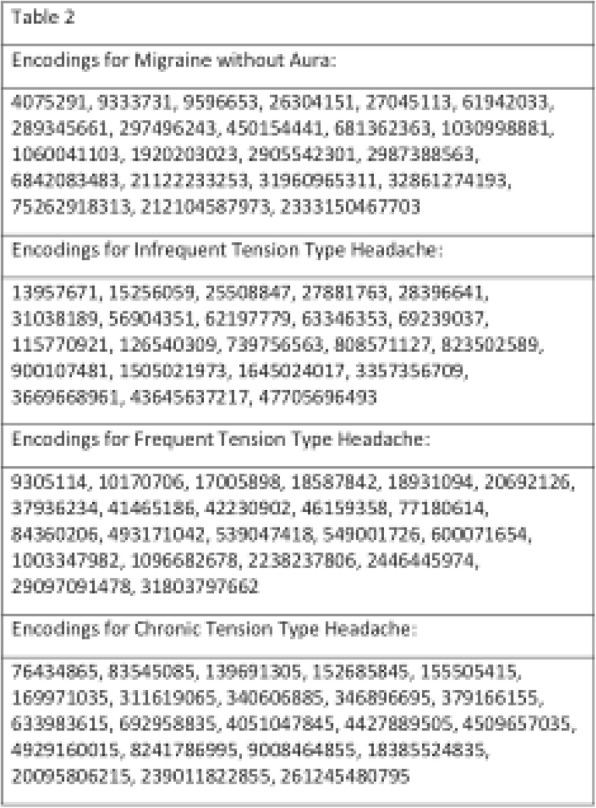
See text for description.

## P200 Title: Health care indicators for patients with headache at the emergency department during the COVID pandemic

### A. Boix Moreno^1^, A. J. Moreno Rojas^1^, M. J. Corujo Suarez^1^, T. Mateos Salas^1^, J. Camiña Muñiz^1^, F. J. Molina Martinez^1^

#### ^1^Hospital Universitario Son Espases, Palma de Mallorca, Spain

##### **Correspondence:** A. Boix Moreno

**Objective**: Analysis of health care indicators in headache patients at the emergency department (ER) to assess potential improvement areas.

**Methods**: Retrospective analysis of health care indicators of the business intelligence tool Discern Analytics 2 of Millenium (Cerner) and PowerBi (Microsoft), in the period 01/01/2019 to 02/28/2022.

**Results**: During the study period, 9,512 episodes of 7,427 patients (1.28 visits per patient) were recorded.

The total number of annual headache visits fell by 20% in 2020 compared to 2019. Similar drop was seen in global visits to the ER (26%). In the first month of the pandemic in our country (March 2020), there was a 46.75% decrease in visits for headaches compared to the previous month. In April 2020, the minimum number of monthly visits was reached with 59.96% reduction compared to the previous year.

Despite the decrease of total patients attended at the ER, the total number of patients admitted for headache was maintained. The 2% of all visits in ER were due to headache. Out of these, 70% were discharged with a diagnosis of nonspecific headache and 20% with a diagnosis of migraine. A brain CT scan was requested in about 40-45% of patients. The 10% were assessed by neurology. Out of these, 53% end up being admitted. 14.70% of patients who go to the emergency department for headaches also consume outpatient resources in neurology.

**Conclusion**: The impact of the pandemic has led to a decrease in the number of patients visited in the ER but the total number of patients admitted for headache has remained practically the same. The 2% of the emergency visits are due to headache. We should work on improving the headache coding at discharge. The number of admissions could be reduced by incorporating new alternative circuits of ambulatory care

## P201 The structure and organization of headache differential diagnoses: A Pilot Study of Subset Relationships between Differentials in ICHD3

### P. Zhang

#### Rutgers Robert Wood Johnson Medical School, Neurology, New Brunswick, NJ, United States

**Objective** Differential diagnosis is fundamental to medicine. Using DiffNet, a differential diagnosis generator, as a model we studied the structure and organization of how collections of diagnose (i.e. sets of diagnoses) are related in the ICHD3. Specifically, we asked: Which sets of differential diagnoses are subsets of each other? What is the minimum number of sets of differential diagnoses that encompass all ICHD3 codes? Furthermore, we explored the clinical and theoretical implication of these answers.

**Methods** DiffNet is a freely distributed differential diagnosis generator for headaches using graph theoretical properties of ICHD3. For each ICHD3 diagnosis, we generated a set of differential diagnoses using DiffNet. We then determined algorithmically the set/subset relationship between these sets. We also determined the smallest list of ICHD3 diagnosis whose differential diagnoses would encompass the totality of ICHD3 diagnoses.

**Results** All ICHD3 diagnoses can be represented by a minimum of 92 differential diagnosis sets. Differential diagnosis sets for 10 of the 14 first digit subcategories of ICHD3 are represented by more than one differential diagnosis sets. Fifty-one of the 93 differential diagnosis sets contain multiple subset relationships; the remaining 42 do not enter into any set/subset relationship with other differential diagnosis sets. Finally, we included a hierarchical presentation of differential diagnosis sets in ICHD3 according to DiffNet.

**Conclusion** We propose a way of interpreting headache differential diagnoses as partial ordered sets (i.e. poset). For clinicians, fluency with the 93 diagnoses and their differential put forth here implies a complete description of ICHD3. On a theoretical level, interpreting ICHD3 differential diagnosis as poset, allows researchers to translate differential diagnoses sets topologically, algebraically, and categorically.

## P202 A comprehensive list of prevention medications without drug-drug interactions can be generated from DrugBank and FAERS.

### J. Dave, I. Hakkinen, P. Zhang

#### Rutgers Robert Wood Johnson Medical School, Neurology, New Brunswick, NJ, United States

##### **Correspondence:** P. Zhang

Background

Preventive medications are key in migraine prevention. In cases of refractory migraine headaches, multiple medications may be required. We seek to identify a comprehensive list of preventive migraine headache medications that can be used in two, three, or four combinations without drug-drug interactions.

Methods

We compiled a list of prevention medications from Szperka et al's "Migraine Care in the Era of COVID-19" as well as American Headache Society's 2018 and 2021 consensus statements on integrating new migraine treatments into clinical practice. We obtained all possible two to four combinations of prevention medications through this list. We then filtered out all combinations containing at least one interaction based on DrugBank or FAERS database.

Results

A total of 27 unique prevention medications are identified. There are a total of 351 combinations of two preventives, 2925 combinations of three preventives, and 17550 combinations of four preventives. When screened using DrugBank, there are a total of 115, 113, and 0 non-interacting two, three, and four preventive combinations, respectively. All non-interacting medications can be represented by a condensed list of 147 unique combinations of medications. When screened using FAERS, there are a total of 288, 1742, and 6875 non-interacting two, three, and four preventive combinations. The non-interacting medications can be represented by a condensed list of 6902 unique combinations of medications.

Conclusion

This list of migraine preventive medications with out drug-drug interactions is an useful tool for clinicians seeking to manage refractory headaches more effectively by implementing an evidence-based polypharmacy.

## P203 Which Co-diagnoses are Possible in the ICHD3? A Number Theoretic Approach

### P. Zhang

#### Rutgers Robert Wood Johnson Medical School, Neurology, New Brunswick, NJ, United States

Background:

In clinical practice, headache presentations may fit more than one ICHD3 diagnoses. This project seeks to exhaustively list all logically consistent "codiagnoses" according to ICHD3 criteria. We limit our project to cases where only two diagnoses are involved.

Methods:

We included the ICHD3 criterias for "Migraine" (1.1, 1.2, 1.3), "Tension-type headache" (2.1, 2.2, 2.3, 2.4), "Trigeminal autonomic cephalalgias" (3.1, 3.2, 3.3, 3.4, 3.5), as well as all "Other primary headache disorders". We excluded "Complications of migraine"(1.5) and "Episodic syndrome that may be associated with migraine" (1.6) since these diagnoses require codiagnoses of migraine as first assumption. We also excluded "probable" diagnosis criteria.

Each phenotype in the above criteria is assigned an unique prime number. We then encoded each ICHD3 criteria into integers, call "criteria representations", through multiplication in a list format. (See Abstract ID-9) "Codiagnoses representations" are generated by multiplying all possible pairings of criteria representations.

To eliminate logical inconsistent codiagnses, we manually encode a list of logically inconsistent phenotypes through multiplication: For example, headache lasting "seconds" would be logically inconsistent with "headache lasting hours"; the prime representation for both are multiplied together. We called this list the "inconsistency representations".

All codiagnoses representation divisible by any inconsistency representations are filtered out, generating a list of codiagnoses represenation that are logically consistent. This list is then translated back into ICHD3 diagnoses.

Results:

A total of 103 phenotypes are encoded with 99 pairs being inconsistent. There are 128 possible codiagnoses. We will present these in the meeting.

Conclusions:

Codiagnoses are possible but uncommon. Prime representation of ICHD3 criteria provides a powerful way to analyze ICHD3 as numerical data.

## P204 Changes in Eeg Microstates after Alpha-Training in Boys and Girls-Adolescent with High Level of Aggression

### T. Kachynska^1^, I. Kuznietsov^1^, I. Khachidze^2^, O. Zhuravlov^1^, O. Abramchuk^1^

#### ^1^Lessya Ukrainka Volyn National University, Human and animal physiology, Lutsk, Ukraine; ^2^Beritashvili Centre of Experimental Biomedicine, LABORATORY OF HUMAN PSYCHOPHYSIOLOGY, Tbilisi, Georgia

##### **Correspondence:** T. Kachynska

The problem of aggression and aggressive behavior in science becomes relevant when a society experiences critical periods of development. Therefore, it is necessary to search for methods of psycho-correction to reduce the level of aggression and improve the psycho-emotional state of adolescent, to prevent the formation of personality with deviant behavior. Neurofeedback is non-invasive and captivating tool for the regulation of aggressive behavior may play a great role for subject's successful social adaptation. Microstate analysis is a prospective method frequently used for assessing brain dynamics reflected in the EEG under varying conditions and brain states. Of specific interest is the application of microstates approach in a neurofeedback domain.

It is still under consideration, which specific microstates are more efficient for persons with high level of aggression during alpha-training. In our study we examined the resting state EEG of 20 subjects (age - 13-14 yy) before and after 5th and 10th session of alpha- training. The EEG microstates were calculated for the whole EEG spectra using eLoreta software.

It was found that electrical activity in adolescents with high levels of aggression before alpha-training can be explained by 4 microstates. In girls 47% of the time was described by microstate A, 13% - B, 12% - C, and 28% - D. In boys 29 % of time was described microstate A, 23 % - В, 20 % - С, 31 % - D. After 10th session of alpha-training in adolescent-girls, microstate A (auditory network) decreases and microstate D (attention network) and C (saliency network) increase. Boys-adolescent were characterized by decrease in microstate A (visual network) and increase microstate C. As a result of alpha training, in boys compared with girls build neural networks with the involvement of the frontal cortex, which leads to more conscious control of aggression and the choice of a behavioral response plan with less involvement of the psycho-emotional component.

## P205 A Study of MeSH Terms in PubMed Headache Case Reports and Clinical Trial Abstracts

### P. Zhang^1,2^

#### ^1^Rutgers Robert Wood Johnson Medical School, Neurology, New Brunswick, NJ, United States; ^2^Cymbeline LLC, Dayton, NJ, United States

Background

Case Reports and clinical trial research represent two important categories of literature in headaches research. MeSH terms are controlled vocabulary used to document medical literature. This project seeks to document the demographic of case reports and clinical trial publication in PubMed through the use of MeSH keywords.

Methods

Using PubMed API, a search is done for "headache" case reports from 1966 to 2022. Basic information, including article abstract, article ID, and article MeSH keywords for each PubMed entry is then downloaded. The frequency of all unique MeSH keywords are tallied. The same method is applied for a search of "headache" clinical trials publications from 1991 to 2022. Both searches, download, as well as analysis are done through Python and Haskell languages.

Results

We downloaded 22,658 case reports and 9,897 clinical trial abstracts as well as their MeSH terms between January 8th, 2022 and January 12th, 2022. As expected, among both case reports and clinical trials, some of the most common MeSH terms are "Human", "Male", "Female", "Adult" and "Headache". For case reports, "Brain Neoplasm", "Meningitis", "Intracranial Aneurysm", "Hematoma", and "Subarachnoid hemorrhage" are the top 5 most frequently reported diseases. Among clinical trials, "Migraine Disorders", "Hypertension", "Tension Type Headaches", "Neoplasm", and "Depressive Disorders" are the top 5 most frequently reported diseases.

Conclusion

A survey of topics of PubMed Case Reports and Clinical Trials in Headaches is possible through the use of MeSH terms. Case reports in headaches appear to focus on secondary headache conditions. Clinical trial literature in headaches, with the exception of neoplasm, appears to involve migraine, tension type headaches as well as their associated co-morbidities.

## P206 How to sequentially try all possible "pick two" combinations of abortive and/or prevention medications: an exploration in hamiltonian cycles in headache

### P. Zhang^1,2^

#### ^1^Rutgers Robert Wood Johnson Medical School, Neurology, New Brunswick, NJ, United States; ^2^Cymbeline LLC, Dayton, NJ, United States

Introduction:

Trying combinations of medications in headaches is often inevitable in clinical practice. However, switching multiple medications concurrently may expose patients to unwanted side effects and introduce confusion in determining efficacies of individual medications. This concern raises the question: Is there a way of systematically trying all possible "pick two" combinations of abortive/preventives while adjusting only one medication at a time?

Methods:

We take as inputs non-interacting lists of abortive medications based on DrugBank from Kayter and Zhang's "Non-interacting, Non-opioid, and Non-barbiturate Containing Acute Medication Combinations in Headache". We generate a mathematical graph using the following definitions: 1) Nodes are all "pick two" medication combinations. 2) An edge exists between two nodes if there is only 1 drug difference between two nodes. We then look for a "Hamiltonian path" for this graph using brute force strategy. (A Hamiltonian path is a way of visiting each nodes in a graph only once by traversing through edges.)

A graph is generated using the same definition for "pick two" prevention medications using non-interacting data from Dave et al (AHS Scientific 2022).

Results:

Hamiltonian path exists for prevention/abortive medications. Table 1 and 2 shows the Hamiltonian path for "pick two" abortives as well as preventive, respectively. (These table results are simplified by condensing some similar medication classes.) Every element in each table differs from the next by only 1 medication. Notice that the last element in the prevention list forms an edge with the first element, therefore forming a cycle. Detailed versions of hamiltonian paths for individual medications will be presented in the meeting.

Conclusion:

It is possible to try all possible non-interacting "pick two" medication combinations by adjusting only one medication at a time. This project is also the first known use of Hamiltonian path in medicine.

**Table 1 (abstract P206).**
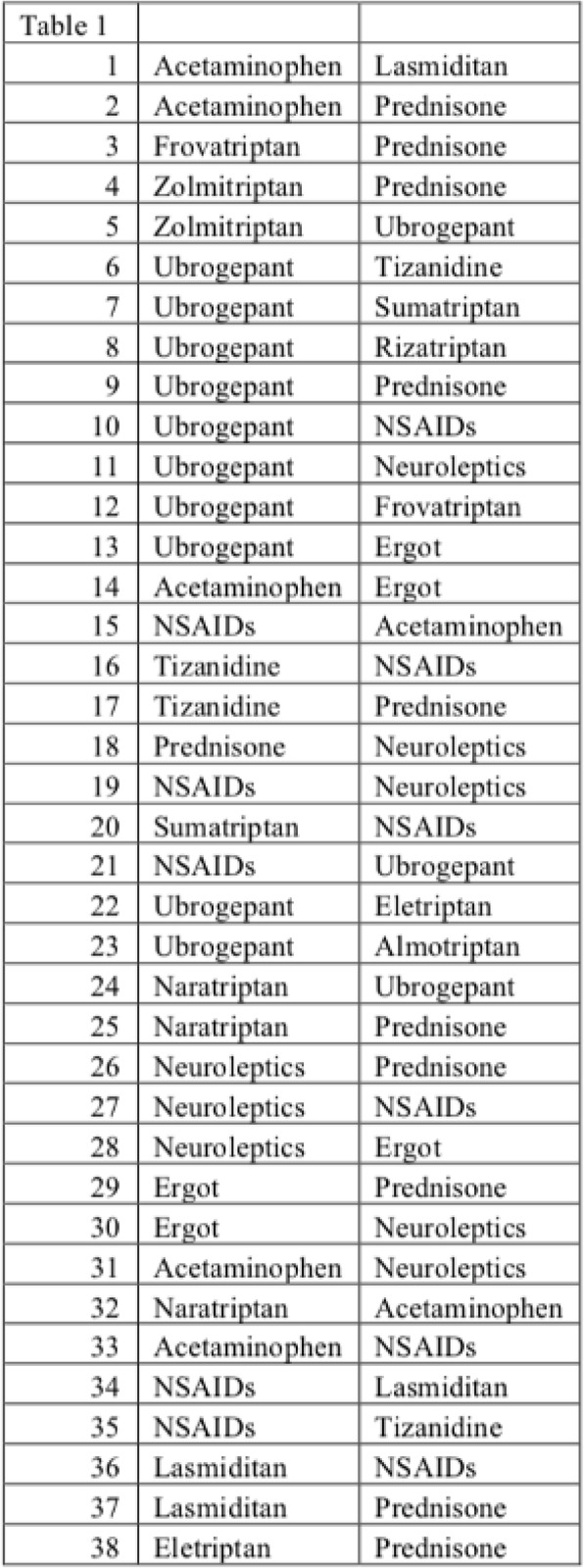
See text for description.

**Table 2 (abstract P206).**
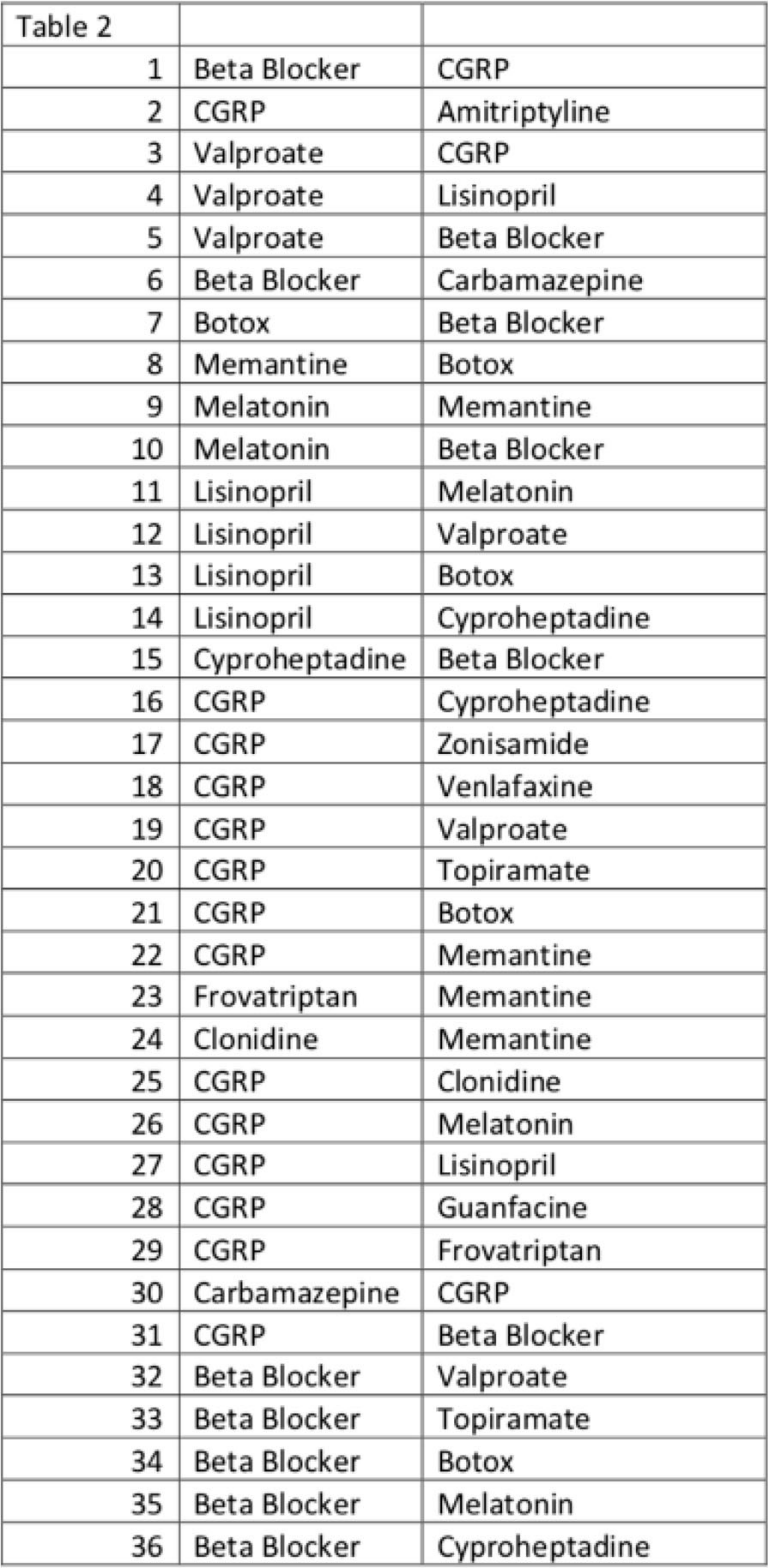
See text for description.

## P207 Health related Quality of life among patients with MOH: One year experience of University Headache Centre

### S. Ljubisavljevic

#### University of Nis, Neurology, Nis, Serbia

*Background:* The negative impact of medication overuse headache (MOH) on the quality of life (QoL) of the patient is undoubted.

*Objective:* The aim of this study was to identify clinical and headache related parameters that directly affect HRQoL of MOH patients.

*Patients and Methods:* 83 patients (11 men and 72 women) firstly diagnosed as having MOH and 81 healthy subjects (22 men and 59 women) in control group (CG) were enrloed in this study. The age of the study subjects range from 18 to 71 years. HRQoL was assessed using a Short Form - 36 questionnaire (SF-36), measuring its Physical Composite Score (PCS), Mental Composite Score (MCS) and Total score (TS).

*Results:* All HRQoL domeins (PCS, MCS, TS) were lower in MOH compared to the CG (p <0.001). In MOH, the depression itself is a risk factor for all aspects of HRQoL, for PCS (B = -0.70, 95% CI -1.32 - - 0.08, p = 0.027); for MCS (B = -0.71, 95% CI -1.14 - - 0.29, p = 0.001); for TS (B = -0.69, 95% CI -1.16 - - 0.22, p = 0.005)), with female gender being an associated risk factor only for PCS (B = -15.47, 95% CI -26.79 - - 4.14, p = 0.008). The results did not find a predictive role of anxiety, stress, and ruminative style of thinking for HRQoL in MOH patients (p>0.05).

*Conclusions:* Screening for depression among MOH patients and its treatment could be useful for improving their HRQoL.

*Keywords*: Medication overuse headache, Health related quality of life

## P208 Outcomes following Ventriculoperitoneal shunts for sight threatening Idiopathic Intracranial Hypertension: a prospective longitudinal cohort study

### Y. Hyder^1^, V. Homer^2^, M. Thaller^1,3^, R. Piccus^4^, G. Tsermoulas^5^, S. Mollan^1,4^, A. Sinclair^1,3,4,6^

#### ^1^University of Birmingham, Institute of Metabolism and Systems Research (IMSR), Birmingham, United Kingdom; ^2^University of Birmingham, Cancer Research UK Clinical Trials Unit, Birmingham, United Kingdom; ^3^University Hospitals Birmingham NHS Foundation Trust, Neurology, Birmingham, United Kingdom; ^4^University Hospitals Birmingham NHS Foundation Trust, Birmingham Neuro-Ophthalmology, Birmingham, United Kingdom; ^5^University Hospitals Birmingham NHS Foundation Trust, Department of Neurosurgery, Birmingham, United Kingdom; ^6^Birmingham Health Partners, Centre for Endocrinology, Diabetes and Metabolism, Birmingham, United Kingdom

##### **Correspondence:** Y. Hyder

**Question:** Idiopathic Intracranial Hypertension (IIH) is a rare neurological disorder characterised by raised intracranial pressure (ICP). Severe cases can manifest with papilloedema and rapidly deteriorating vision. Reduction of ICP can be achieved through surgical procedures such as ventriculoperitoneal shunt (VPS) insertion. We aimed to establish detailed characteristics of patients with IIH who required surgical intervention. Secondly, we sought to describe their long-term recovery.

**Methods:** Data was collected prospectively at clinical visits. This included headache frequency, measures of papilloedema and ganglion cell layer volume (GCLV) on optical coherence tomography (OCT) imaging, perimetric mean deviation (MD) in people with IIH requiring VPS at a large regional neuroscience centre. Loess smoothers were used to characterise outcomes following surgery.

**Results:** 51 patients underwent VPS insertion (92% female [47/51], age 28.1 [SD 8.4], BMI 37.4 [SD 9.7], mean follow up 330 days [SD 290]). Characteristics of the worst eye at baseline included MD of -11.4 dB [SD 9.7], retinal nerve fibre layer thickness 381.9 μm [SD 116.6] and Frisen grade papilloedema 4.3 [SD 0.9].

Post-operatively, markers of papilloedema showed complete resolution by 4 months. Alarmingly, mean GCLV steadily declined from 1.05 μm3 at baseline to 0.95μm3 12 months post-operatively. Headache frequency fell from 12.7 days per month to 3.4 days at 3 month post-operatively, before increasing to 13.8 days by 12 months.

**Conclusions:** VPS insertion leads to a dramatic and sustained reduction in papilloedema by 4 months in IIH. However, macular ganglion cell layer loss continued at 12 months following surgery which may predispose to future sight loss. Whilst headache severity often improves following VPS, this is inconsistent and not sustained.

## P209 The Idiopathic Intracranial Hypertension Life Long Asymptomatic study: Evaluation of the impact of an asymptomatic IIH presentation on outcomes

### M. Thaller^1,2,3^, V. Homer^4^, S. Mollan^1,5^, A. Sinclair^1,2,3^

#### ^1^University of Birmingham, Institute of metabolism and systems research, Birmingham, United Kingdom; ^2^University Hospitals Birmingham NHS Foundation Trust, Neurology, Birmingham, United Kingdom; ^3^Birmingham Health Partners, Centre for Endocrinology, Birmingham, United Kingdom; ^4^University of Birmingham, Cancer Research (UK) Clinical Trials Unit, Birmingham, United Kingdom; ^5^University Hospitals Birmingham NHS Foundation Trust, Birmingham Neuro-Ophthalmology, Birmingham, United Kingdom

##### **Correspondence:** M. Thaller

Question

Papilloedema can be an incidental finding at a routine optician review or neurological examination, and some of these would be asymptomatic. The long-term prognosis of these patients in comparison to the more common symptomatic population needs to be investigated.

Methods

Evaluate key outcomes such as vision (LogMAR visual acuity; Humphrey visual field perimetric mean deviation (PMD) and optical coherence tomography (OCT)) and headache in a prospectively collected cohort within the IIH Life database (2012-2021). Comparison was made according to whether they were symptomatic at diagnosis or not.

Results

Truly asymptomatic presentations are uncommon (10%, 36/343), with incidental papilloedema more common at 35% (121/343). Asymptomatic IIH patients had similar visual outcomes compared to those with symptomatic disease, with as expected lower headache frequency outcomes.

Conclusions

Asymptomatic IIH can pose a challenge to the neurology and ophthalmology teams managing IIH. We have shown that prognosis is similar to that of the more "typical" IIH and there should be managed as per standard IIH guidelines.

## P210 Metabolomic Analysis Reveals Remodelling of central and systemic metabolite pathways in Idiopathic Intracranial Hypertension linked to disease activity and headache generation

### O. Grech^1^, S. Seneviratne^1^, Z. Alimajstorovic^1^, A. Yiangou^1^, J. Mitchell^1^, T. Smith^2^, S. Mollan^3^, G. Lavery^4^, C. Ludwig^1^, A. Sinclair^1^

#### ^1^University of Birmingham, Institute of Metabolism and Systems Research, Birmingham, United Kingdom; ^2^University of Cambridge, Addenbrooke’s Hospital, Cambridge, United Kingdom; ^3^University Hospitals Birmingham, Birmingham Neuro-Ophthalmology, Birmingham, United Kingdom; ^4^Nottingham Trent University, School of Science and Technology, Nottingham, United Kingdom

##### **Correspondence:** O. Grech

Question

The pathogenesis of Idiopathic Intracranial Hypertension (IIH) remains poorly understood and this lack of knowledge hinders advances in IIH. Mounting evidence indicates that IIH is no longer considered exclusively a disease of the central nervous system, but instead involves systemic metabolic perturbation. We sought to determine if metabolic disturbances are evident in IIH and if they are ameliorated by disease remission.

Methods

A case control study utilised proton nuclear magnetic resonance spectroscopy for metabolomic profiling of CSF, serum and urine in IIH patients (n=84) compared to age, gender and body mass index matched controls (n=20). Assessments included intracranial pressure (ICP), headache and papilledema measurements, and were repeated following a 12-months weight loss intervention n IIH patients (n=50).

Results

We identified a distinct metabolic profile in IIH featuring 4 predominant metabolites. Urea was lower in IIH (CSF p<0.001, urine p=0.009) correlated with ICP (p=0.019) and headache severity (p=0.031) and increased by 12 months (CSF p=0.004, urine p=0.043). The lactate:pyruvate ratio was increased in IIH (CSF p=0.023, serum p=0.004) and decreased at 12 months (p<0.001). Acetate was higher in IIH (p=0.008), correlated with headache severity and disability (p = 0.030, p= 0.003) and decreased at 12 months (p = 0.007). Ketones 3-hydroxybutyrate and acetoacetate were altered in IIH CSF and normalized at 12 months (p = 0.019, p = 0.015).

Conclusion

This IIH metabolomics study demonstrates systemic metabolic disturbances evident in CSF, serum and urine. Urea, an osmolar metabolite, was reduced in IIH and normalised as ICP improved. Perturbed lactate:pyruvate ratio, a marker of respiratory chain function, suggests dysregulation of systemic and central metabolic flux. Elevated acetate was associated with headache morbidity. These alterations of metabolic pathways provides biological insight and warrants mechanistic evaluation.

## P211 The Idiopathic Intracranial Hypertension Life Long Pregnancy study: Evaluation of the impact of pregnancy on outcomes

### M. Thaller^1,2,3^, V. Homer^4^, S. Mollan^1,5^, A. Sinclair^1,2,3^

#### ^1^University of Birmingham, Institute of metabolism and systems research, Birmingham, United Kingdom; ^2^University Hospitals Birmingham NHS Foundation Trust, Neurology, Birmingham, United Kingdom; ^3^Birmingham Health Partners, Centre for Endocrinology, Birmingham, United Kingdom; ^4^University of Birmingham, Cancer Research (UK) Clinical Trials Unit, Birmingham, United Kingdom; ^5^University Hospitals Birmingham NHS Foundation Trust, Birmingham Neuro-Ophthalmology, Birmingham, United Kingdom

##### **Correspondence:** M. Thaller

Question

IIH is a known metabolic neuro-ophthalmologic disorder which is associated with body weight gain. One of the known commonest reasons why a woman of childbearing age would gain weight is pregnancy. What is the long-term impact of IIH diagnosed during pregnancy? Does pregnancy affect the visual and headache outcomes?

Methods

Evaluate key outcomes such as vision (LogMAR visual acuity; Humphrey visual field perimetric mean deviation (PMD) and optical coherence tomography (OCT)) and headache in a prospectively collected cohort within the IIH Life database (2012-2021). Comparison was made to those with a subsequent pregnancy, and those whom never become pregnant.

Results

377 patients had pregnancy data recorded. IIH diagnosed in pregnancy is rare. People diagnosed with IIH in pregnancy had worse structural visual outcomes (Mean OCT total retinal thickness), although comparable visual fields and acuity, compared to those who had a pregnancy during the disease course. None took intracranial pressure lowering medicines, few required a temporising lumbar puncture in the first trimester and less required sight saving surgery. Pregnancy during the IIH disease course did not adversely affect visual or headache outcomes. Headache outcomes showed variability reflecting the IIH cohort as a whole.

Conclusions

IIH patient monitoring during pregnancy is important, not only for maternal health but physician communication. Medical intervention is limited due to risk of teratogenicity. Those diagnosed with IIH in pregnancy, or those in whom IIH is exacerbated by pregnancy are more challenging to manage and require individualised care plans.

## P212 The Idiopathic Intracranial Hypertension Life Long PCOS study: Prevalence of comorbid PCOS and evaluation of the impact of PCOS on outcomes

### M. Thaller^1,2,3^, V. Homer^4^, S. Mollan^1,5^, A. Sinclair^1,2,3^

#### ^1^University of Birmingham, Institute of metabolism and systems research, Birmingham, United Kingdom; ^2^University Hospitals Birmingham NHS Foundation Trust, Neurology, Birmingham, United Kingdom; ^3^Birmingham Health Partners, Centre for Endocrinology, Birmingham, United Kingdom; ^4^University of Birmingham, Cancer Research (UK) Clinical Trials Unit, Birmingham, United Kingdom; ^5^University Hospitals Birmingham NHS Foundation Trust, Birmingham Neuro-Ophthalmology, Birmingham, United Kingdom

##### **Correspondence:** M. Thaller

Question

Idiopathic intracranial hypertension (IIH) and Polycystic ovary syndrome (PCOS) affect women of reproductive age with obesity but have different hyperandrogenic profiles. The prevalence of comorbid PCOS in IIH patients is highly variable in the literature; and the longitudinal impact on visual and headache outcomes are unknown.

Methods

Assess the prevalence in a prospective IIH cohort (IIH Life database (2012-2021)) based on Rotterdam criteria from questionnaire and routine clinical practice data. Secondary aim to evaluate the impact of PCOS on IIH outcomes (visual and headache).

Results

398 females with IIH were followed up for a median of 10 months (range 0-87) and had presence or absence of PCOS documented. Prevalence of PCOS in IIH was 19.6% (78/398) by the Rotterdam criteria, with additional 14.6% (58) describing hyperandrogenic symptoms alone. There was a 3.2-fold increased risk for self-reported fertility problems and 4.4-fold for requiring medical help if comorbid PCOS was reported.

Females with IIH and comorbid PCOS did not have significantly different visual outcomes from those without PCOS, although the total retinal thickness improved more rapidly following baseline review in the PCOS cohort. Headache outcomes were variable and similar between the groups, with a worse initial headache frequency but more rapid improvement in the PCOS cohort.

Conclusions

Symptomatic hyperandrogenism is common in IIH patients who have previously been noted to have elevated levels of testosterone. Diagnosing co-morbid PCOS is important as it can impact fertility and long-term cardiovascular risk. From an IIH management aspect, comorbid PCOS does not confer worse visual or headache outcomes.

## P213 Impact of rater experience on detecting MRI features of idiopathic intracranial hypertension

### G. Bsteh^1^, W. Marik^2^, S. Macher^1^, V. Schmidbauer^2^, N. Krajnc^1^, P. Pruckner^1^, C. Mitsch^3^, K. Novak^4^, C. Wöber^1^, B. Pemp^3^

#### ^1^Medical University of Vienna, Neurology, Vienna, Austria; ^2^Medical University of Vienna, Neuroradiology, Vienna, Austria; ^3^Medical University of Vienna, Ophthalmology, Vienna, Austria; ^4^Medical University of Vienna, Neurosurgery, Vienna, Austria

##### **Correspondence: G. Bsteh**

*Question:* In idiopathic intracranial hypertension (IIH), certain MRI features are promising diagnostic markers, but impact of radiologist"s experience on identifying these features correctly is unknown. Therefore, we compared ratings in daily routine by radiologists with unknown awareness of IIH-MRI-features with the ratings of a junior neuroradiologist aware of features but without special IIH training and a senior neuroradiologist with experience in IIH imaging (gold-standard).

*Methods:* For comparing the 3 settings, we included patients from the Vienna-Idiopathic-Intracranial-Hypertension (VIIH) database with definitive IIH according to Friedman criteria and routine cranial MRI performed for suspected IIH and assessed frequencies of empty sella (ES), optic nerve sheath distension (ONSD), optic nerve tortuosity (ONT), posterior globe flattening (PGF) and transverse sinus stenosis (TSS).

*Results:* We evaluated MRI scans of 84 IIH patients (88% female, mean age 33.5 years). By gold-standard, 78.6% had ≥1 IIH-MRI-feature and 52.9% had ≥3 features with ONSD most frequent (64.3%) followed by TSS (60.0%), ONT (46.4%), ES (44.0%) and PGF (25.0%). Compared to gold standard, IIH features were described significantly less frequently in routine MRI reports (≥1 feature 64.3%, p=0.04; ≥3 features 15.7%, p<0.001; ONSD 28.6%, p<0.001; TSS 42.9%, p=0.04; ONT 13.1%, p<0.001; PGF 4.8%, p<0.001) except for ES (42.9%, p=0.9). Contrary, rating by a neuroradiologist without special training produced significantly higher frequencies of ≥1 / ≥3 MRI features (95.2%, p=0.001; 72.5%, p=0.017), ONSD (81.0%, p=0.015) and ONT (60.7%, p=0.049), but not ES (47.6%, p=0.6), TSS (68.1%, p=0.3) and PGF (29.8%, p=0.5).

*Conclusions:* IIH-MRI-features are underestimated in routine MRI reports and partly overestimated by less experienced neuroradiologists, driven by features less well known or methodologically difficile. Reevaluation of MRI scans by an experienced rater improves diagnostic accuracy.

**Fig. 1 (abstract P213).**
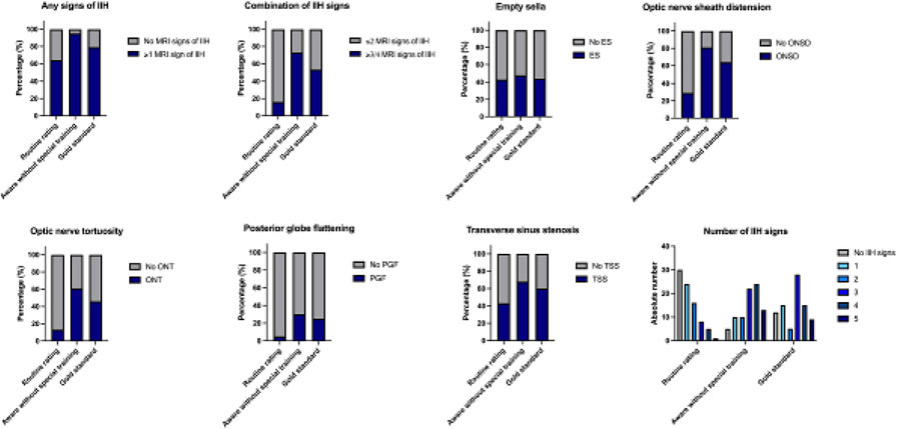
See text for description.

## P214 MRI features of idiopathic intracranial hypertension are not prognostic of headache outcome

### G. Bsteh^1^, W. Marik^2^, N. Krajnc^1^, S. Macher^1^, P. Pruckner^1^, C. Mitsch^3^, K. Novak^4^, B. Pemp^3^, C. Wöber^1^

#### ^1^Medical University of Vienna, Neurology, Vienna, Austria; ^2^Medical University of Vienna, Neuroradiology, Vienna, Austria; ^3^Medical University of Vienna, Ophthalmology, Vienna, Austria; ^4^Medical University of Vienna, Neurosurgery, Vienna, Austria

##### **Correspondence:** N. Krajnc

*Question:* In idiopathic intracranial hypertension (IIH), certain MRI features are promising diagnostic markers, but whether these have prognostic value is controversially discussed.

*Methods:* We analyzed patients from the Vienna-Idiopathic-Intracranial-Hypertension (VIIH) database with definitive IIH according to Friedman criteria and cranial MRI performed at diagnosis. Presence of empty sella (ES), optic nerve sheath distension (ONSD), optic nerve tortuosity (ONT), posterior globe flattening (PGF) and transverse sinus stenosis (TSS) was assessed by a senior neuroradiologist with experience in IIH imaging.

Two endpoints of headache outcome were defined 12 months after IIH diagnosis: headache improvement (reduction of headache severity and/or frequency by ≥50%) and freedom of headache (Multivariate regression models were calculated regarding headache improvement/freedom with IIH MRI features as independent variables adjusted for sex, age at diagnosis, symptom duration, body mass index [BMI], and chronic headache at baseline (>15 days/month for ≥3 months).

*Results:* We included 84 IIH patients (88% female, mean age 33.5 years, median BMI 30.8, 6% IIH without papilledema). At baseline, headache was present in 84.5% (54.8% chronic). Headache improvement was achieved in 83.8%, freedom of headache in 25.7%. At least one IIH MRI feature was found in 78.6% and 52.9% had ≥3 features with ONSD most frequent (64.3%) followed by TSS (60.0%), ONT (46.4%), ES (44.0%) and PGF (25.0%).

In multivariate models, neither any single IIH MRI feature nor ≥1, ≥3 or a combination of features were associated with headache improvement or freedom. Chronic headache at baseline was significantly associated with lower likelihood of headache freedom (odds ratio 0.23, p<0.001), but not headache improvement.

*Conclusions:* IIH MRI features are not prognostic of headache outcome. Chronic headache at diagnosis is an unfavourable predictor of headache outcome.

**Fig. 1 (abstract P214).**
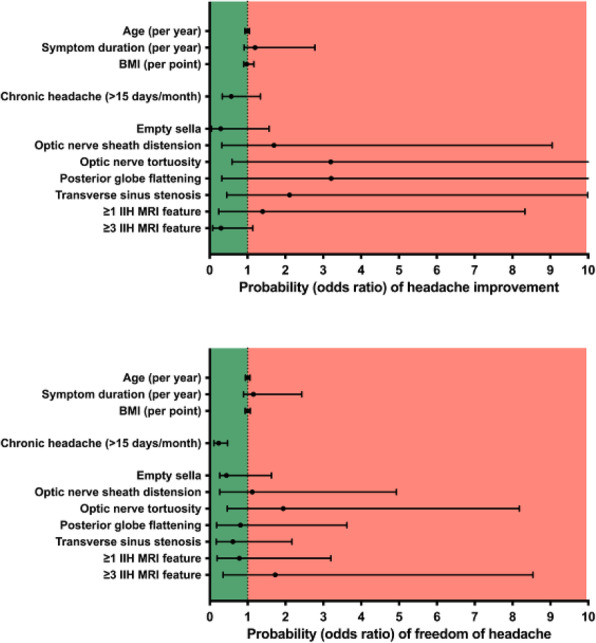
See text for description.

## P215 First telemetric real-time evidence of intracranial pressure increases during spontaneous headache in idiopathic intracranial hypertension: a case series

### A. Yiangou^1^, J. Mitchell^1^, H. Lyons^1^, J. Walker^1^, O. Grech^1^, Z. Alimajstorovic^1^, G. Tsermoulas^2^, S. Mollan^1^, A. Sinclair^1^

#### ^1^University of Birmingham, Institute of Metabolism and Systems Research, Birmingham, United Kingdom; ^2^University Hospitals Birmingham, Department of Neurosurgery, Birmingham, United Kingdom

##### **Correspondence:** A. Yiangou

**Objective**

The use of telemetric intracranial pressure (ICP) monitors has been increasing. The mechanisms of idiopathic intracranial hypertension (IIH) headache have not been fully elucidated. We describe a case series of seven patients with active IIH that a spontaneous headache occurred during real-time telemetric monitoring of ICP.

**Methods**

Patients with active IIH (>25 cmCSF lumbar puncture opening pressure and papilloedema) were enrolled in a prospective, randomized, placebo controlled, double blind, parallel group exploratory trial (IIH Pressure Trial: ISRCTN12678718). Following insertion of an intraparenchymal ICP monitor (Raumedic™ Neurovent p-Tel, Hembrechts, Germany) participants were randomized to receive Exenatide (10 mcg BD subcutaneous) or placebo for 12 weeks. They underwent assessments including ICP monitoring, and headache phenotyping using a paper diary and a semi-structured interview.

**Results**

Participants (n=15) had a mean (SD) age of 28(9) years, BMI 38.1(6.2) kg/m2, supine ICP 23.5 (3.9) mmHg and a converted lumbar puncture-position ICP of 32.2(5.6) cmCSF. Seven patients suffered from an acute spontaneous headache attack during the research visits. During the headache we recorded a significant increase in mean (SD) ICP of 12(6)mmHg, p=0.001 in these patients. A maximum ICP of 104 mmHg was recorded at the peak of a headache attack (severity numeric rating scale 10 out of 10) in a participant which then returned baseline (26 mmHg) as pain settled. We also noted an increased ICP waveform fluctuation as the pain severity score escalated and during the duration of the headache attack.

**Conclusions**

This is the first report to demonstrate real-time evidence of rising ICP and increased fluctuations during an IIH headache. It provides unique insights into the mechanisms of headache in IIH. It further provides future direction to drive research to investigate acute ICP lowering agents for IIH headache.

## P216 MRI features of idiopathic intracranial hypertension are not prognostic of visual outcome

### G. Bsteh^1^, W. Marik^2^, S. Macher^1^, N. Krajnc^1^, P. Pruckner^1^, C. Mitsch^3^, K. Novak^4^, C. Wöber^1^, B. Pemp^3^

#### ^1^Medical University of Vienna, Neurology, Vienna, Austria; ^2^Medical University of Vienna, Neuroradiology, Vienna, Austria; ^3^Medical University of Vienna, Ophthalmology, Vienna, Austria; ^4^Medical University of Vienna, Neurosurgery, Vienna, Austria

##### **Correspondence:** G. Bsteh

*Question:* In idiopathic intracranial hypertension (IIH), certain MRI features are promising diagnostic markers, but their prognostic value is controversial.

*Methods:* We analyzed patients from the Vienna-Idiopathic-Intracranial-Hypertension (VIIH) database with definitive IIH according to Friedman criteria and cranial MRI performed at diagnosis. Presence of empty sella (ES), optic nerve sheath distension (ONSD), optic nerve tortuosity (ONT), posterior globe flattening (PGF) and transverse sinus stenosis (TSS) was assessed by a senior neuroradiologist with experience in IIH imaging. Impaired visual outcome was defined as a combined endpoint of visual acuity ≥0.1 logMAR and/or mean deviation <-2.0 dB in static threshold perimetry 12 months after diagnosis. Visual worsening was defined as worsening by ≥0.2 logMAR and/or ≥2.0 dB. Multivariate binary logistic regression models were calculated regarding poor visual outcome/visual worsening with IIH MRI features as independent variables adjusted for sex, age at diagnosis, symptom duration, BMI and visual dysfunction at baseline.

*Results:* We included 84 patients (88% female, mean age 33.5 years, median body mass index 30.8, 6% IIH without papilledema). At baseline, visual impairment was present in 70.2%. Impaired visual outcome occurred in 57.1% and visual worsening in 11.9%. At least one IIH MRI feature was found in 78.6% and 52.9% had ≥3 features with ONSD most frequent (64.3%) followed by TSS (60.0%), ONT (46.4%), ES (44.0%) and PGF (25.0%).Neither any single IIH MRI feature nor ≥1, ≥3 or a combination of features were associated with impaired visual outcome or visual worsening. Visual dysfunction at baseline predicted impaired visual outcome (odds ratio 7.6, p=0.001), but not visual worsening.

*Conclusions:* IIH MRI features are neither prognostic of impaired visual outcome nor further visual worsening from the time of IIH diagnosis. Visual impairment at diagnosis remains the only established predictor of visual outcome.

**Fig. 1 (abstract 216).**
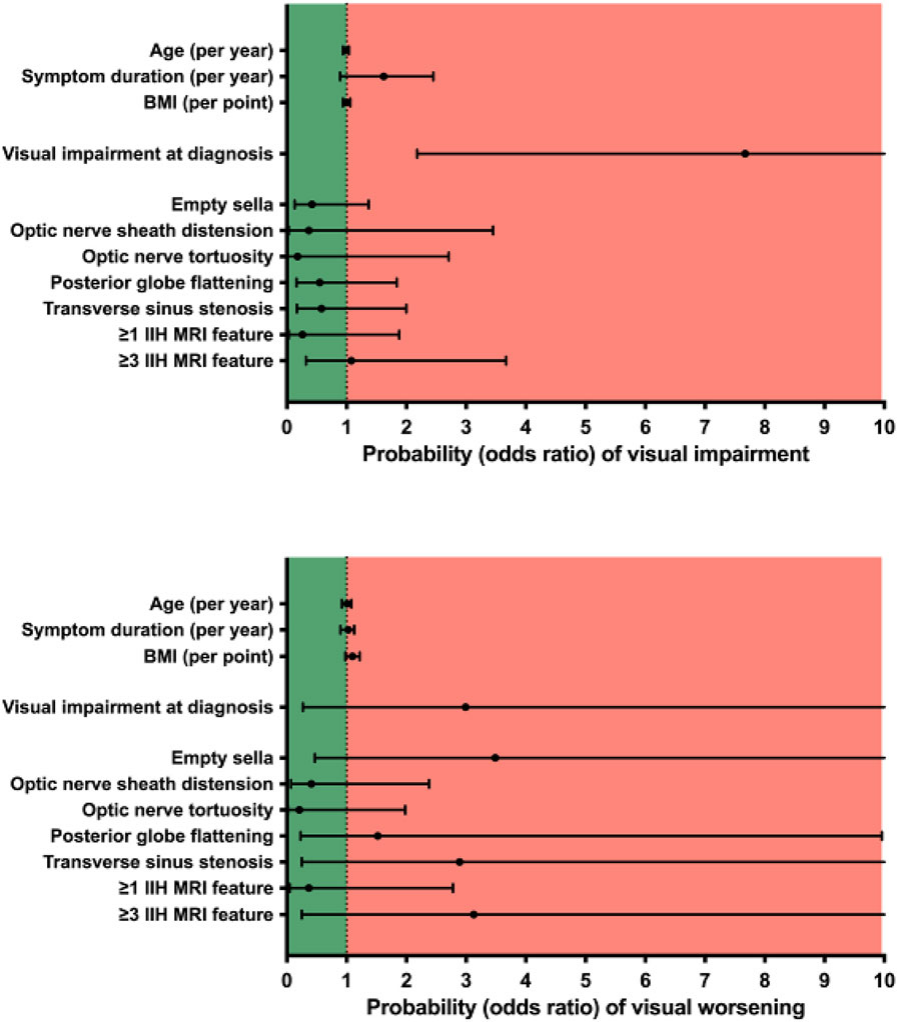
See text for description.

## P217 Idiopathic intracranial hypertension presenting with migraine phenotype is associated with unfavorable headache outcome

### G. Bsteh^1,2^, S. Macher^1,2^, N. Krajnc^1,2^, P. Pruckner^1,2^, W. Marik^3^, C. Mitsch^4^, K. Novak^5,2^, B. Pemp^4^, C. Wöber^1,2^

#### ^1^Medical University of Vienna, Neurology, Vienna, Austria; ^2^Medical University of Vienna, Comprehensive Center for Clinical Neurosciences & Mental Health, Vienna, Austria; ^3^Medical University of Vienna, Neuroradiology, Vienna, Austria; ^4^Medical University of Vienna, Ophthalmology, Vienna, Austria; ^5^Medical University of Vienna, Neurosurgery, Vienna, Austria

##### **Correspondence:** G. Bsteh

*Q:* Migrainous headache is common in idiopathic intracranial hypertension (IIH), but its prognostic impact is unclear. Thus, we compared IIH with and without migraine phenotype.

*M:* We analyzed patients from the Vienna-Idiopathic-Intracranial-Hypertension (VIIH) database with definitive IIH according to Friedman criteria. We recorded CSF opening pressure and ophthalmologic findings and classified headache (HA) according to ICHD-3beta as migraine (IIH-MIG) or non-migrainous and absent (IIH-noMIG). Parameters were defined 12 months after IIH diagnosis comprising HA improvement (≥50% reduction of HA severity and/or frequency), freedom of HA (<1 HA day/month), impaired visual outcome (visual acuity ≥0.1 logMAR and/or mean deviation <-2.0 dB in static threshold perimetry) and visual worsening (≥0.2 logMAR and/or ≥2.0 dB worsening from baseline).

*R:* We included 97 patients (88.7% female, mean age 32.9 years, median BMI 32.0, 6.2% IIH-WOP, median CSF opening pressure 31cmH2O). IIH-MIG comprised 46.4% and IIH-noMIG 53.6% of the patients (11.3% tension-type HA, 25.8% unclassifiable HA, 16.5% no HA).

At baseline, IIH-MIG differed from IIH-noMIG with respect to monthly HA days (22 vs. 15, p=0.003) and HA severity (6.5 vs. 4.5; p<0.001). Age, BMI, CSF opening pressure, proportion of IIH-WOP, and visual acuity did not significantly differ between groups.

At follow-up, IIH-MIG compared to IIH-noMIG showed significantly lower rates for improvement and freedom of HA in all patients (66.7% vs 88.5%, p=0.009; 11.1% vs 42.3%, p=0.006) as well as in those with resolution of papilledema (n=40; 63.2% vs 95.2%, p=0.011; 5.3% vs 61.9%, p<0.001). Persistent visual impairment did not differ in the two groups (55.6% vs 57.7%), and visual worsening even tended to be less common in IIH-MIG than in IIH-noMIG (11.5% vs 24.4%, p=0.093).

*C:* In IIH, migrainous headache is associated with adverse outcomes for headache even when papilledema has resolved, but possibly with favorable visual outcome.

**Fig. 1 (abstract P217).**
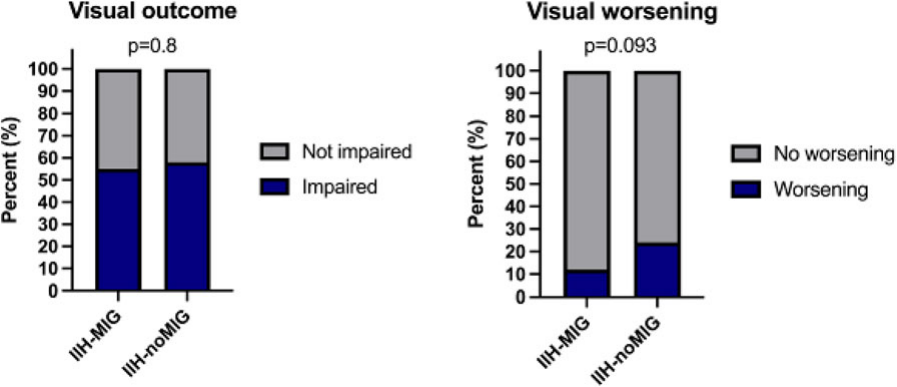
See text for description.

**Fig. 2 (abstract P217).**
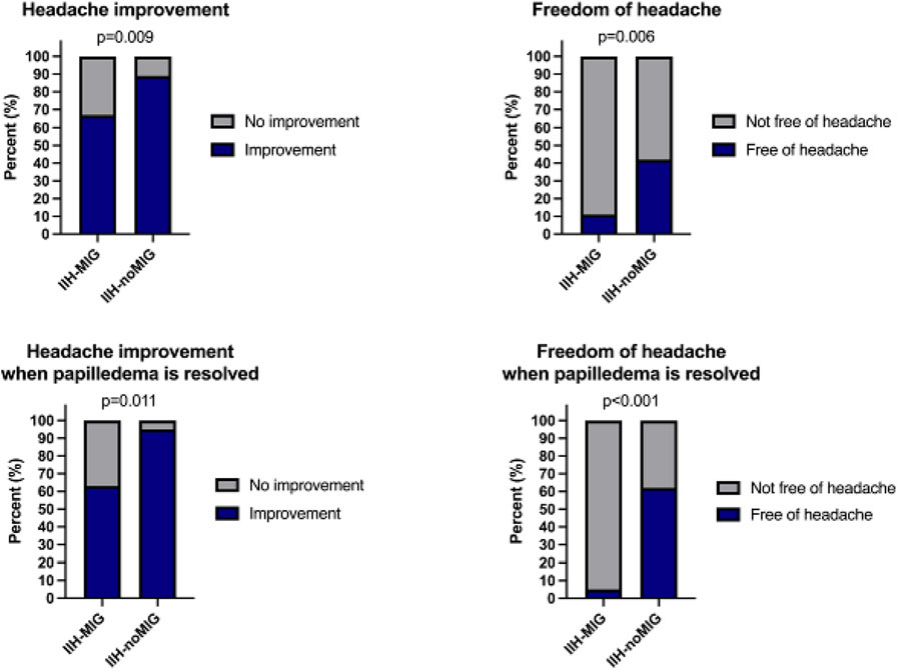
See text for description.

## P218 The Acute and Chronic Effects of Acetazolamide and Topiramate on Intracranial Pressure and CSF Secretion

### C. Westgate^1^, C. Kamp-Jensen^1^, I. Israelsen^1^, T. Toft-Bertelsen^2^, J. Wardman^2^, C. Jensen^1^, B. Styrishave^3^, N. MacAulay^2^, R. H. Jensen^1^, S. Eftekhari^1^

#### ^1^University of Copenhagen, Rigshospitalet, Danish Headache Center, Copenhagen, Denmark; ^2^University of Copenhagen, Rigshospitalet, Department of Neuroscience, Copenhagen, Denmark; ^3^University of Copenhagen, Rigshospitalet, Department of Pharmacy, Copenhagen, Denmark

##### **Correspondence:** C. Westgate

Question

Lowering intracranial pressure (ICP) is the primary rationale of pharmacotherapy in Idiopathic intracranial hypertension (IIH). There is limited evidence behind the use of the leading therapeutics, acetazolamide and topiramate, and how they lower ICP. We assessed the ICP lowering capacity of these drugs in rats and the molecular consequences hereof.

Methods

In a randomized, blinded cross-over study, we assessed the capacity of acetazolamide and topiramate to modulate ICP in female Sprague Dawley rats (N=10) using continuous telemetric ICP monitoring. We assessed single supra-clinical doses over 24 hours, and twice-daily clinically equivalent doses over 10 days. Drugs were delivered via oral gavage. The effects on CSF secretion rates and gene expression at the choroid plexus (CP) for CSF secretory genes were separately evaluated.

Results

Over 24 hours, both acetazolamide and topiramate lower ICP compared to vehicle with peak reduction of 25% at 2 hours. Administering these drugs in combination doubles the ICP lowering effect. Over 10 days, both acetazolamide and topiramate lower daily ICP compared to vehicle with no evidence of tachyphylaxis. Over the course of the day, the effect of acetazolamide wore off overnight whereas the ICP lowering effect of topiramate was sustained overnight relative to control. In accordance, topiramate reduced CSF secretion by 40%. Paradoxically, topiramate increased the expression of CSF secretory genes *Slc12a2* and *Slc4a10* at CP, whereas acetazolamide has no such effect.

Conclusion

We demonstrate that both acetazolamide and topiramate rapidly lower ICP following administration and that there is an additive lowering ICP effect with combination of the drugs. The ICP lowering effect of these drugs persists with no tachyphylaxis with long term administration. These data thus provides the functional rationale for the clinical use of the combination of acetazolamide and topiramate in IIH and other conditions of raised ICP.

## P219 The Idiopathic Intracranial Hypertension Life Long Normal BMI study: Evaluation of the impact of a normal BMI at baseline on outcomes.

### M. Thaller^1,2,3^, V. Homer^4^, S. Mollan^1,5^, A. Sinclair^1,2,3^

#### ^1^University of Birmingham, Institute of metabolism and systems research, Birmingham, United Kingdom; ^2^University Hospitals Birmingham NHS Foundation Trust, Neurology, Birmingham, United Kingdom; ^3^Birmingham Health Partners, Centre for Endocrinology, Birmingham, United Kingdom; ^4^University of Birmingham, Cancer Research (UK) Clinical Trials Unit, Birmingham, United Kingdom; ^5^University Hospitals Birmingham NHS Foundation Trust, Birmingham Neuro-Ophthalmology, Birmingham, United Kingdom

##### **Correspondence:** M. Thaller

Question

Atypical Idiopathic Intracranial Hypertension is a term for cases that do not fit into the classical phenotype of reproductive aged females with obesity. Normal BMI at presentation is uncommon, with potentially different underlying mechanism and management, given that weight loss is currently the only disease-modifying therapy in IIH. Therefore, the prognosis for this subset of atypical IIH needs to be investigated.

Aims and methods

Through a prospectively collected cohort within the IIH Life database (2012-2021), based on baseline BMI. Evaluate visual and headache outcomes. These would include LogMAR visual acuity; Humphrey visual field perimetric mean deviation (PMD) and optical coherence tomography (OCT).

Results

Visual and headache outcomes are not significantly different by baseline BMI with comparable outcomes.

Conclusions

Patients with a normal body mass index make up a small proportion of IIH patients but appear to have similar visual and headache outcomes than more typical IIH. The metabolic phenotype may be different in these patients; however they may also be more susceptible to sequelae of IIH at lower weights.

## P220 The Vienna Idiopathic Intracranial Hypertension (VIIH) database – an Austrian real-world cohort

### P. Pruckner^1^, C. Mitsch^2^, S. Macher^1^, N. Krajnc^1^, W. Marik^3^, K. Novak^4^, C. Wöber^1^, B. Pemp^2^, G. Bsteh^1^

#### ^1^Medical University of Vienna, Neurology, Vienna, Austria; ^2^Medical University of Vienna, Ophthalmology, Vienna, Austria; ^3^Medical University of Vienna, Neuroradiology, Vienna, Austria; ^4^Medical University of Vienna, Neurosurgery, Vienna, Austria

##### **Correspondence:** G. Bsteh

*Background:* Idiopathic intracranial hypertension (IIH) is an increasingly prevalent disease bearing the risk of visual impairment and affecting quality of life. Clinical presentation and outcome are heterogeneous. Large, well-characterized cohorts are scarce.

*Objective:* To describe the Vienna-Idiopathic-Intracranial-Hypertension (VIIH) database aiming to characterize the clinical spectrum, diagnostic findings, therapeutic management and outcome of IIH.

*Methods:* Applying the modified Dandy criteria we identified 113 IIH-patients treated at our center between 2014 and 2021.

*Results:* Of 113 patients, 89% were female (mean age 32.3 years). Median body mass index (BMI) was 31.8, with 85% overweight (BMI>25). Papilledema was found in 95% with 5% classified as IIH without papilledema. Headache was present in 84% and showed migrainous features in 36%. Median opening pressure in lumbar puncture was 31cmH2O.

Pharmacotherapy (predominantly acetazolamide) was established in 99%, 56% required at least one therapeutic lumbar puncture and 13% surgical intervention.

After a median follow-up of 3.7 years, 43% had not achieved significant weight loss, papilledema was present in 49% and headache in 76% (58% improved).

Comparing initial presentation to follow-up, perimetry was abnormal in 67% vs. 50% (8% worsened, 24% improved) and transorbital sonography in 87% vs 65% with a median optic-nerve-sheath-diameter of 5.4mm vs. 4.9mm. Medianperipapillary-retinal-nerve-fiber-layer thickness had decreased from 199μm to 94μm and ganglion-cell-layer thickness from 40μm to 36μm.

*Conclusions:* The VIIH database constitutes a large representative and well-characterized cohort and emphasizes substantial long-term sequelae of IIH. Future analyses will aim to refine phenotyping and identify factors predicting outcome.

## P221 Impact of valsalva and exercise in idiopathic intracranial hypertension: a case series

### A. Yiangou^1^, M. Thaller^1^, S. Weaver^2^, J. Mitchell^1^, H. Lyons^1^, G. Tsermoulas^3^, S. Mollan^1^, S. Lucas^2^, A. Sinclair^1^

#### ^1^University of Birmingham, Institute of Metabolism and Systems Research, Birmingham, United Kingdom; ^2^University of Birmingham, School of Sport, Exercise and Rehabilitation Sciences, Birmingham, United Kingdom; ^3^University Hospitals Birmingham, Department of Neurosurgery, Birmingham, United Kingdom

##### **Correspondence:** A. Yiangou

**Objective**

The impact of straining and exercise on intracranial pressure (ICP) regulation and headache is poorly understood in idiopathic intracranial hypertension (IIH). We sought to investigate whether straining and exercise change ICP and cerebrovascular dynamics in IIH.

**Methods**

Patients with IIH were enrolled in a prospective exploratory trial (IIH Pressure Trial: ISRCTN12678718). After insertion of an intraparenchymal ICP monitor (Raumedic™ Neurovent p-Tel, Hembrechts, Germany) three participants underwent continuous real-time ICP monitoring coupled with cerebrovascular assessments (heart rate, blood pressure, beat-by-beat middle cerebral artery (MCA) blood flow velocity and prefrontal cerebral haemodynamics (near infrared spectroscopy)). During these assessments participants undertook valsalva maneuvers (VMs) and moderate exercise.

**Results**

The age of the three participants was mean(SD) 40.7(14.4) years, BMI of 38.3(2.1)kg/m2, a supine ICP 15.3(8.7)mmHg and a sitting (upright) ICP of 2.8 (8.0)mmHg. A substantial increase in mean(SD) ICP was noted during VMs of 28.5(14.9)mmHg with an accompanied reduction of -9.1(3.3)mmHg at the end of the VM before returning to baseline. There was an initial reduction of MCA blood flow -20.0(7.4)cm/s followed by an increase of 14.8(5.6)cm/s before returning to baseline. Similar trends were noted in the cerebral pre-frontal cortex perfusion dynamics. The ICP was the fastest to reach its peak during the VMs and within 19(5.6) seconds it returned to baseline. There were no substantial differences in the ICP measures during exercise compared to baseline.

**Discussion**

These initial observations of ICP changes during VMs and rapid return to baseline are of relevance in a population of patients in whom valsalva is a function of daily activities as well as repeated VMs occurring during labour. The observations during exercise are important in a disease that is driven by obesity that can be modified by increased energy expenditure.

## P222 Sex-dependent differences in expression of membrane proteins involved in cerebrospinal fluid secretion at choroid plexus.

### I. M. E. Israelsen, C. Kamp-Jensen, C. S. J. Westgate, R. Højland Jensen, S. Eftekhari

#### Danish Headache Center Glostrup Research Institue Rigshospitalet Glostrup, Neurology, Glostrup, Denmark

##### **Correspondence:** C. Kamp-Jensen

**Question**

Idiopathic intracranial hypertension (IIH) is a disease characterized by an elevated intracranial pressure (ICP). As IIH predominantly occurs in obese women in the reproductive age it has been hypothesized that an altered hormonal composition could affect the activity of transporters involved in cerebrospinal fluid (CSF) secretion, thus affecting ICP. We aimed to investigate if gene expression of various transporters involved in CSF secretion at choroid plexus (CP) were different between males and females and between females in different estrous cycle states.

**Method**

10 metestrus (MET), 10 estrous (ES) and 10 male, all Sprague Dawley rats (11-13 weeks) were used. Female rats during MET have higher hormonal levels compared to ES. The estrous cycle stage was determined by wet vaginal smear and microscopy before euthanasia. CP from lateral and 4th ventricles were collected. CP was subjected to RT-qPCR analysis.

**Results**

We found difference between males and females during estrous cycle stage. Gene expression of the water transporter Aqp1 was higher in males compared to ES females (P<0.01). Expression of the gene encoding for NKCC1 (water transporter) and carbonic anhydrase II was higher in males and MET females compared to ES females (P<0.0001). Further, the gene encoding for carbonic anhydrase III was lower in males and MET females compared to ES females (P<0.05). Furthermore, when comparing gene expression in females at different cycle states the expression of sodium-bicarbonate cotransporter, NBCe2, were higher in MET females compared to ES females (P<0.05). There were no differences in expression of Aqp4 and NCBE.

**Conclusion**

This study demonstrates that gene expression at CP is affected by the estrous cycle in rats. Further, expression of some transporters was sex-dependent during estrous stage. This opens the possibility that the expression of transporters involved in CSF may be regulated by hormones and be linked to the pathophysiology of IIH.

## P223 IIH in the elderly: A rare entity

### A. Dubey^1^, S. Dubey^2^

#### ^1^GMC & Hamidia Hospital, Bhopal, India; ^2^All India Institute of Medical Sciences, Neurology, Bhopal, India

##### **Correspondence:** A. Dubey

Question

Idiopathic Intracranial Hypertension (IIH) is a rare cause of headache. It's usual age of presentation is in 3rd and 4th decades of life. However, it is seen in elderly population also, but with different clinical presentation. We present such a case here.

Methods

An 80 year old female with no previous comorbidities, presented to Ophthalmology department with complaints of blurring of vision with transient visual obscurations for around one year with mild generalized headache since 6 months. Examination revealed BP of 184/98 mmHg with papilloedema. She was subjected to brain imaging.

Results

MRI brain with MR venography was normal. Patient denied any history of prolonged drug intake. Hence, lumbar puncture was performed which showed opening pressure of 35cm H_2_O with normal chemical composition. Thus, diagnosis of IIH was established as per modified Dandy's criteria. She was started on oral acetazolamide therapy with good response.

Conclusion

IIH is rarely reported in very elderly patients. In this age group, it generally presents along with hypertension and with visual symptoms being more dominant when compared to headache predominant presentation in the young. A careful history and meticulous examination is needed to diagnose this rare entity in the elderly age group. Consent to publish had been obtained.

## P224 The diagnostic validity of the detailed history and clinical findings in cervicogenic headache: a systematic review and meta-analysis of diagnostic studies

### A. Demont^1^, S. Lafrance^2,3^, J. Mawet^4^

#### ^1^Paris University, 1123 ECEVE, Paris, France; ^2^Montréal University, School of Rehabilitation, Faculty of Medicine, Montreal, Canada; ^3^Maisonneuve-Rosemont Hospital Research Center, Orthopaedic Clinical Research Unit, Montreal, Canada; ^4^Lariboisiere Hospital, Neurology, Paris, France

##### **Correspondence:** A. Demont

**Objective:** To update and evaluate available evidence of the prevalence and the diagnostic accuracy of the detailed history and clinical findings for cervicogenic headache in adults with headache.

**Methods**: CINAHL, Cochrane Central, Embase, PEDro and PubMed were searched for studies before March 2022 that reported detailed history and/or clinical findings related to the diagnosis of cervicogenic headache. Study selection, risk of bias assessment (QUADAS-2 and PROBAST), and data extraction were performed. Meta-analyses for the cervical flexion-rotation test was performed. Certainty of the evidence was assessed with the GRADE approach.

**Results:** Eleven studies were included. Moderate certainty evidence indicated that the cervical flexion-rotation test differentiated cervicogenic headache from lower cervical facet-induced headache, migraine, concomitant headaches or asymptomatic subjects (Se 83.0% [95%CI:70.0%-92.0%]; Sp 83.0% [95%CI:71.0%-91.0%]; positive LR 5.0 [95%CI:2.6-9.5]; negative LR 0.2 [95%CI:0.1-0.4]; n=4 studies; n=182 participants). Several diagnostic classifications and test clusters based on headache history and clinical findings can be useful, despite uncertain accuracy, in formulating the diagnosis of cervicogenic headache.

**Conclusion**: Evidence support to undertake a subjective evaluation of headache history and signs and symptoms and a physical examination of the patient neck to diagnose cervicogenic headache. During the physical examination, a positive or negative cervical flexion-rotation test has small to moderate effect on the probability of a patient having a cervicogenic headache. The diagnostic value of the other findings remains unclear.

## P225 Efficacy of Non-Pharmacological interventions in patients with cervicogenic headache. A Systematic Review

### M. Shahien^1^, A. Elaraby^1^, M. Gamal^2^, A. Azam^3^, Y. Samir^3^

#### ^1^Cairo University, Cairo, Egypt; ^2^Tanta University, Cairo, Egypt; ^3^Misr University for Science and Technology, Cairo, Egypt

##### **Correspondence:** M. Shahien

Question

Are non-pharmacological interventions effective in improving symptoms in patients suffering from cervicogenic headach?

Methods

We performed an electronic search through various databases, and following PRISMA statement guidelines: PubMed, Cochrane central register of clinical trials, Web of science and Embase. We examined articles against the inclusion criteria to include only randomized clinical trials. Data of included articles were extracted and reviewed.

Results

14 randomized clinical trial Met our criteria and included in the study. Due to the significant heterogeneity, we could not perform meta-analysis for the included studies. Various modalities found to be used to manage cervicogenic headache as spinal manipulation, mobilization, ischemic compression, myofascial release and exercises. Our results revealed that these interventions showed significant improvement in relation to headache frequency and intensity, range of movement of cervical rotation, pressure pain threshold (P < 0.001, p < 0.05) respectively.

Conclusion

Non-pharmacological interventions show promising results in reducing the severity of symptoms and improving quality of life in patients with cervicogenic headache. However, there is no supporting evidence regarding the use of such modalities because of the wide variability among interventions parameters. In addition, more high quality randomized clinical trials with larger sample size and objective measurement tools are required to synthesize a clear evidence.

## P226 Characterising the extended phenotype of paediatric migraine: a prospective study

### N. Karsan^1,2^, H. Gosalia^1^, P. Goadsby^1^, P. Prabhakar^2^

#### ^1^King's College London, London, United Kingdom; ^2^Great Ormond Street Hospital for Children, Department of Neurology, London, United Kingdom

##### **Correspondence:** N. Karsan

**Question**

We set out to perform prospective extended phenotyping of children presenting to a tertiary headache service.

**Methods**

Consecutive new migraineurs presenting to the Children"s Headache Clinic at Great Ormond Street Hospital for Children between 6th January- 6th September 2022 were included (*n*=51). A detailed headache history was taken at the first consultation by a trained headache physician. A questionnaire was used to ensure complete symptomatic capture. Data were tabulated and analysed (IBM SPSS v 28). Descriptive statistics, Chi-square and Pearson correlation analyses were used. Significance was assessed at *P* < 0.05.

**Results**

Patients were 69% female and aged 8-16 years (mean 13, SD 2), with mean disease duration 5 years (SD 3). Baseline monthly headache frequency was 1-30 days (median 30, IQR 10-30). Chronic migraine was the most common diagnosis (61%). Aura was present in 45%. At least one infantile migraine marker was present in 71%; the most common were travel sickness (45%) and colic (41%). At least one premonitory symptom (PS) was reported by 94%, at least one cranial autonomic symptom (CAS) by 71% and premonitory CAS by 18%. Vertigo, allodynia and neck stiffness were also reported. The most common perceived triggers were stress (43%), concentration (22%) and bright lights (22%). CAS and headache lateralities co-associated (휒2 15, *P*=0.005). There was a positive correlation between disease duration and the number of PS reported (Pearson correlation coefficient 0.3, *P*=0.026). There was a negative correlation between gestational age at birth and number of PS reported (Pearson correlation coefficient -0.4, *P*=0.009).

**Conclusion**

The extended paediatric migraine phenotype includes several non-canonical migraine symptoms. Similarly to in adults, CAS can occur prior to headache and tend to lateralise with headache. There may be an association of PS with disease chronicity and a suggestion that prematurity is associated with more PS.

**Table 1 (abstract 226).**
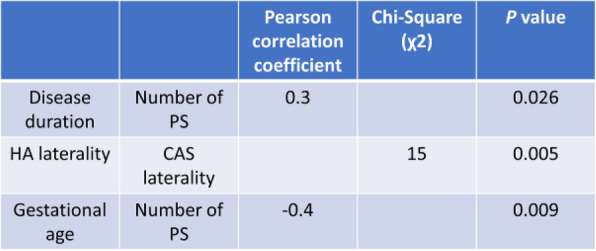
See text for description.

## P227 Sleep Disorders in Pediatric Migraine: a questionnaire-based study

### A. Voci^1^, O. Bruni^2^, M. A. N. Ferilli^3^, L. Papetti^3^, S. Tarantino^3^, F. Ursitti^3^, G. Sforza^3^, F. Vigevano^3^, L. Mazzone^1^, M. Valeriani^3,4^, R. Moavero^1,3^

#### ^1^Tor Vergata University of Rome, Child Neurology and Psychiatry Unit, Systems Medicine Department, Rome, Italy; ^2^Department of Developmental and Social Psychology, Sapienza University, Rome, Italy; ^3^Headache Center, Child Neurology Unit, Neuroscience Department, Bambino Gesù Children’s Hospital, IRCCS, Rome, Italy; ^4^Center for Sensory-Motor Interaction, Aalborg, Denmark

##### **Correspondence:** A. Voci

Migraine and sleep disorders are frequently comorbid and linked by a mutual dependance, possibly representing the expression of a common pathogenic process. This study aimed to analyze the relationship between headache features (migraine frequency and severity, presence of migraine equivalents, use and efficacy of medications) and sleep in pediatric migraine.

Parents of children and adolescents with migraine completed two standardized sleep assessment questionnaires, the Children"s Sleep Habits Questionnaire (CSHQ) and the Epworth Sleepiness Scale for Children and Adolescents (ESS-CHAD), and answered questions about headache characteristics in their children. The presence of sleep disorders was defined according to CSHQ"s total score.

The CSHQ revealed a sleep disturbance in 72.9% of 140 subjects, but only 5.0% had already received a diagnosis. Patients with sleep disturbances presented statistically significant higher headache frequency (*p*=0.031) and higher prevalence of migraine equivalents (*p*=0.007). A higher CSHQ"s total score was associated with higher frequency of severe attacks (*p*=0.012) and lower efficacy of acute medications (*p*=0.003). Significant positive correlations of sleep onset delay (*p*=0.006), sleep duration (*p*=0.005) and nightwakings (*p*=0.047) subscales with migraine frequency also emerged. Only 2.8% of patients reached a clinically significant score in the ESS-CHAD.

Our findings indicate that sleep disorders are highly prevalent in pediatric migraine and frequently associated with higher headache severity and lower response to acute therapy, but often remain underdiagnosed. Given the relationship between sleep and migraine characteristics, improving sleep quality, through sleep hygiene interventions and specific treatments, could help to reduce migraine intensity and disability and vice versa.

## P228 Interictal cognitive performance in children and adolescents with primary headache: a narrative review

### S. Tarantino^1^, M. Proietti Checchi^1^, L. Papetti^1^, F. Ursitti^1^, G. Sforza^1^, M. A. N. Ferilli^1^, R. Moavero^1,2^, G. Monte^1^, T. Grimaldi Capitello^1^, F. Vigevano^1^, M. Valeriani^1,3^

#### ^1^Bambino Gesù Children's Hospital, Neuroscience, Rome, Italy; ^2^Tor Vergata University of Rome, Child Neurology and Psychiatry Unit, Rome, Italy; ^3^Aalborg University, Center for Sensory-Motor Interaction, Aalborg, Italy

##### **Correspondence:** S. Tarantino

Question. Primary headache is a very common and disabling disease. The burden of pain and recurrent attacks may lead to a poor quality of life, anxiety and depression. An increased risk of low functioning and curricular performances in young patients with primary headache has been described. The mechanisms underlying the relationship between migraine and poor school achievement may be various and could be a reflection of weak cognitive skills. Data concerning the cognitive functioning in the free pain interval in pediatric age are under-investigated and results are far from conclusive. Methods. Suitable studies were identified using MEDLINE and Web of Science. Search terms included "Pediatric migraine" or "Pediatric headache" and "Cognitive performance", "Cognitive impairment" or "Neuropsychology", "Intelligence", "Attention", "ADHD", "Memory", "Language", "Visuo-spatial", "Coordination" and "Difficulties" or "Problems". We considered papers involving subjects of an age ranging from 0 to 18 years. We also included articles that, though focusing on adults, included subjects < 18 years old. Results. The present review article suggests that, though considered a benign disease, pediatric migraine may be associated to altered neuropsychological functioning in the interictal phase. Although children and adolescents with migraine generally have a normal intelligence, they may show a not homogeneous cognitive profile, characterized by possible difficulties in verbal skills, in particular comprehension abilities. Pediatric primary headache may present altered neuropsychological functioning involving attentional resources, processing speed and memory, particularly verbal memory. Conclusions. Given the impact that this disease can have on school performance and the tendency to persist from childhood to adulthood, a cognitive screening in young patients affected by primary headache is pivotal. Additional neuropsychological research using more homogenous methods is needed.

## P229 Severe abrupt (thunderclap) non-traumatic headache at the pediatric emergency department

### T. Eidlitz Markus^1^, Y. Levinsky^2,1^

#### ^1^Tel Aviv University, Sackler Faculty of Medicine, Tel Aviv, Israel; ^2^Schneider Children's Medical Center, Day Hospitalization Department, Petach Tikva, Israel

##### **Correspondence:** T. Eidlitz Markus

**Backgroun**d: Adult abrupt severe non-traumatic headache (thunderclap) is often related to serious underlying etiologies such as subarachnoid hemorrhage. However, data are sparse regarding thunderclap headache in the pediatric population.

**Objective**: The aim of the study was to evaluate the prevalence, characteristics and causes of thunderclap headache in the pediatric and adolescent population, aged 6–18 years, presenting to a pediatric emergency department.

**Methods**: The electronic database of a tertiary care pediatric emergency department was searched for children presenting with acute headache during 2016–2018. Headache severity was defined by pain scales, either a visual analogue scale or by the Faces Pain Scale–Revised. The study was approved by the Research Ethics Board of Rabin Medical Center (approval no. RMC-19-704). Due to the retrospective study design, the committee waived the need for informed consent.

Results: During the three-year study period, 104,086 children and adolescents aged 0-18 years were admitted to the pediatric emergency department; of them, A total of 2290 children, aged 6-18 years (mean 13.3 ± 3.26) were admitted with a chief complaint of headache, and reported their level of pain according to one of the two scales used 3112 (3%) presented with acute headaches Thunderclap headache was diagnosed in 19/2290 (0.8%) of the included patients, all of them with a pain score of 10/10. All the patients had a benign course. Primary headache was diagnosed in 15/19 (78.9%), six patients had migraine and eight were diagnosed with primary thunderclap headache. Four of the 19 patients were diagnosed with secondary headache: three with infectious causes and one with malignant hypertension. **Conclusions**: Thunderclap headache is rare among children and adolescents presenting to the emergency department. This headache is generally of a primary origin. Extensive evaluation is still needed to rule out severe diagnosis problems.

**Fig. 1 (abstract P229).**
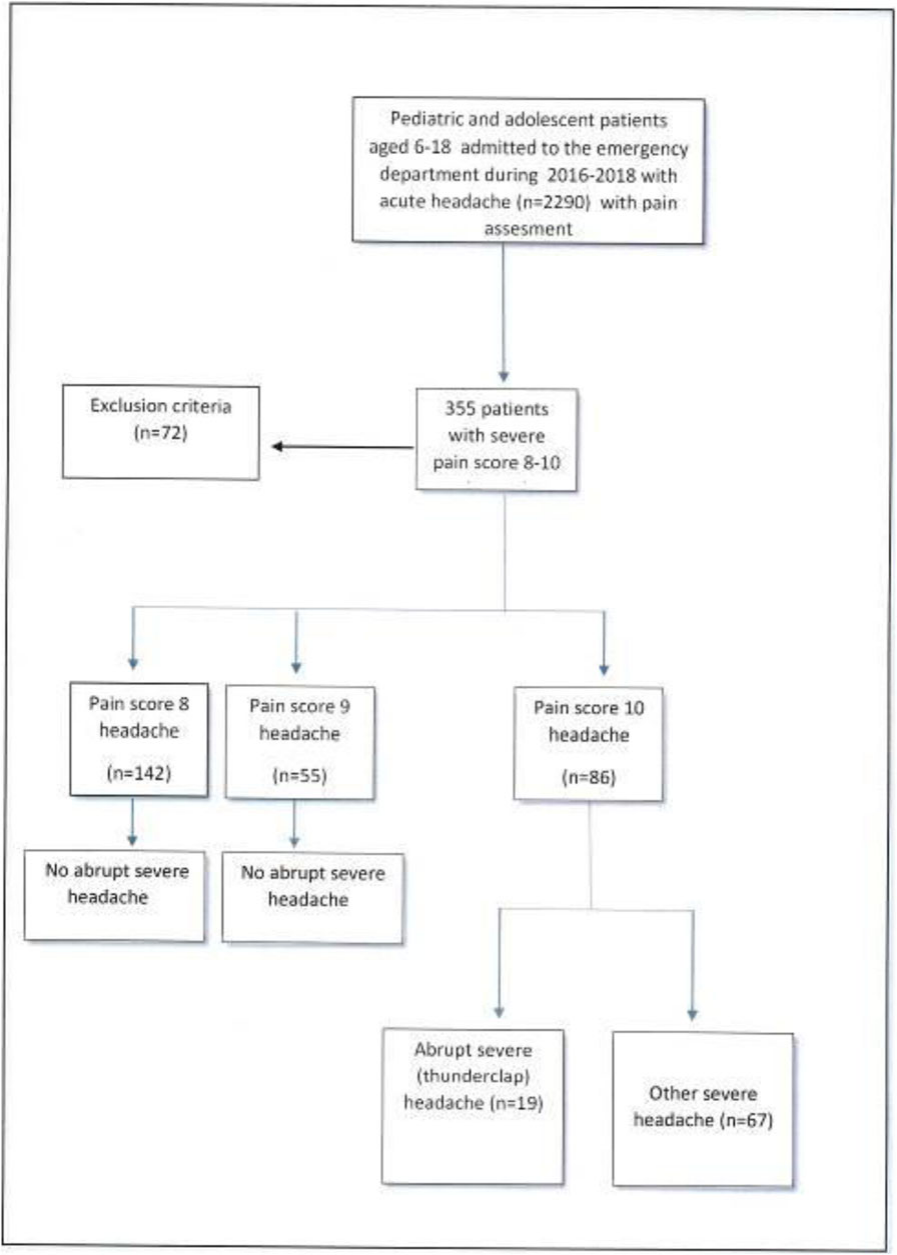
Flowchart of the study depicts the distribution of the cohort

**Fig. 2 (abstract P229).**
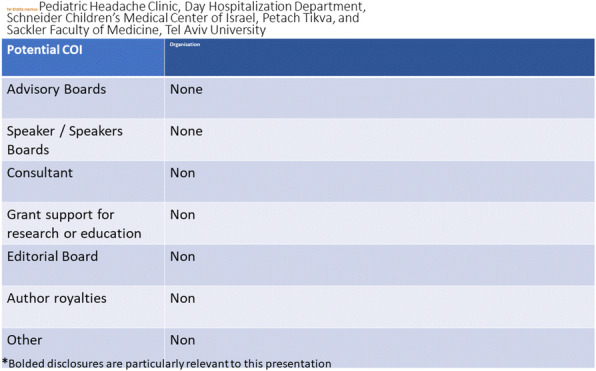
See text for description.

## P230 Cutaneous allodynia in pediatric and adolescent patients and their mothers – a comparative study

### T. Eidlitz Markus^1,2^, K. Raibin^1,2^

#### ^1^Tel Aviv University, Sackler Faculty of Medicine, Tel Aviv, Israel; ^2^Schneider Children's Medical Center, Day Hospitalization Department, Petach Tikva, Israel

##### **Correspondence:** T. Eidlitz Markus

**Background:** Allodynia in adults with migraine is related to disease duration. In pediatric patients with migraine, the same proportion reported allodynia in the first six months of migraine presentation as in prolonged disease. This study examined a possible association of migraine pediatric allodynia with maternal allodynia.

**Methods:** We interviewed children with migraine first, and then their mothers, regarding allodynia and headache symptoms. We reviewed hospital charts on pediatric medical background and headache symptoms. Mothers and children older than 11 years filled the Strengths and Difficulties Questionnaire.

Mothers gave their informed consent to their participation and their children's participation in the study. of the pediatric participants were collected from the patients" files.

The study was approved by the Research Ethics Committee of Rabin Medical Center (approval no. RMC-0294-18RMC).

**Results:** Ninety-eight children with migraine, mean age 13.49±3.1 years, and their mothers, mean age 43.5±6.2 years were recruited to the study. Pediatric allodynia was associated with maternal allodynia; the latter was reported in 82.8% of children with allodynia versus 35.3% of children without allodynia (p<0.001). Maternal migraine was reported in 44 (68.7%) of children with allodynia versus 16.3% without allodynia, p

**Conclusions:** Pediatric allodynia is associated with maternal migraine. Genetic and environmental factors such as maternal behavior may contribute to reduced pain threshold.

**Fig. 1 (abstract 230).**
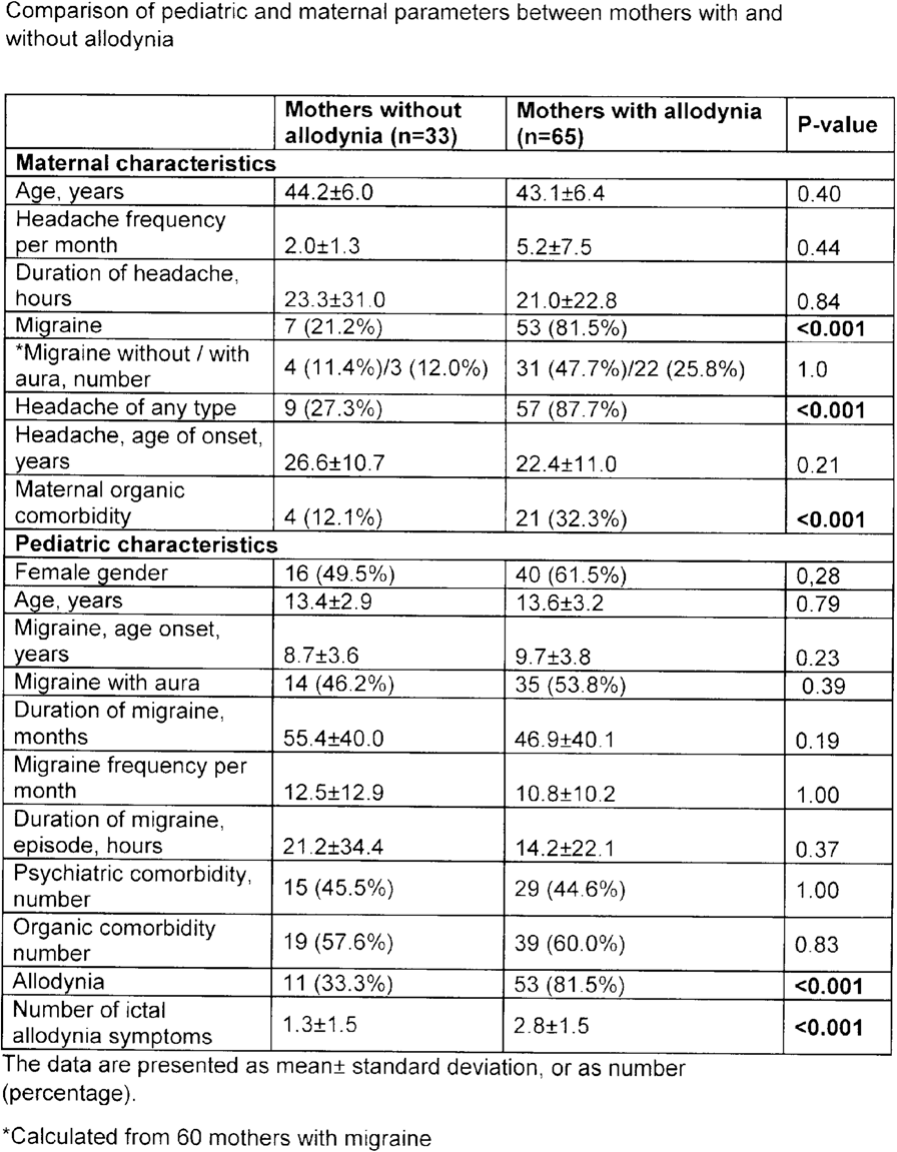
See text for description.

**Fig. 2 (abstract 230).**
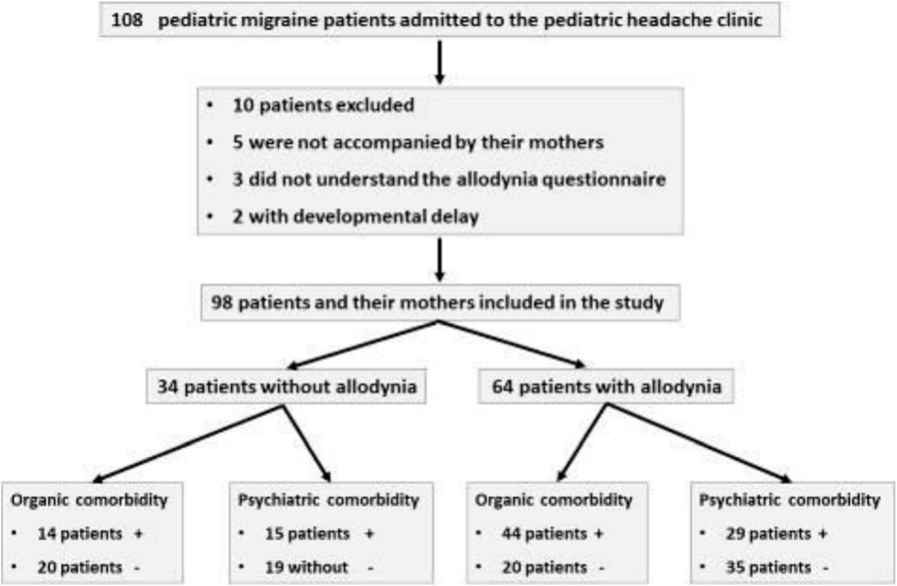
See text for description.

## P232 Long-term effects of pandemic of Covid-19 on clinical features and psychological symptoms in adolescents with migraine

### M. Proietti Checchi^1^, S. Tarantino^2^, L. Papetti^1^, F. Ursitti^1^, G. Sforza^1^, R. Moavero^1,3^, G. Monte^1^, F. Vigevano^1^, T. Grimaldi Capitello^2^, M. Valeriani^1,4^

#### ^1^Bambino Gesù Children's Hospital, Neurology, Rome, Italy; ^2^Bambino Gesù Children's Hospital, Unit of Clinical Psychology, Rome, Italy; ^3^Tor Vergata University of Rome, Child Neurology and Psychiatry Unit, Rome, Italy; ^4^Center for Sensory-Motor Interaction, Neurology, Aalborg, Denmark

##### **Correspondence:** M. Proietti Checchi and S. Tarantino

*Question:* We aimed to compare the clinical characteristics of migraine, use of prophylaxis, and psychological symptoms between patients who referred to our Headache Centre before the COVID-19 pandemic and those who were evaluated during the pandemic, with a further distinction between the first phase and the prolonged second phase.

*Methods:* We studied 418 adolescents with migraine (m.a. 14±1.7; 110 M and 308 F). Based on the pandemic period, patients were grouped into 'Pre Covid' or 'Covid'. Moreover, the second group was divided into 'Covid 1' (March to October 2020, characterised by lockdown) and 'Covid 2' (November 2020 to January 2022, characterised by prolonged restrictions). Patients were grouped into: (1) high frequency (weekly to daily episodes) and low frequency (≤3 episodes per month); (2) mild and severe pain; (3) need for prophylactic treatment or not. The PHQ-9 and GAD-7 questionnaires were used to assess anxiety and depression symptoms.

*Results*: We did not find a significant difference in migraine frequency between the "Pre Covid" and "Covid" periods (p=0.295). In the "Covid 2" period, frequency of the attacks was increased, compared to both the "Pre Covid" period (p=0.038) and the "Covid 1" period (p=0.005). Furthermore, more patients needed prophylactic treatment in the 'Covid' period, especially in the 'Covid 2' period (p=<0.001), than in the 'Pre Covid' period (p=<0.001). Our patients showed higher levels of anxiety and depression during the 'Covid' period (GAD-7, p=0.013 and PHQ-9, p=<0.001), especially during the 'Covid 2' period (p=<0.001).

*Discussion:* Our results show a long-term negative impact of the Covid-19 pandemic on clinical parameters and psychological symptoms of adolescents with migraine. Considering the relationship between migraine severity and emotional symptomatology, our results suggest that monitoring the emotional status of pediatric patients with migraine is mandatory in the near future of COVID-19 pandemic.

## P233 From the new diagnostic criteria to COVID 19 pandemic passing throught the placebo effect. What have we learned in the management of pediatric migraine over the past 5 years? The opinion of Italian pediatric headache centers

### L. Papetti, S. Tarantino, F. Ursitti, R. Moavero, M. Checchi Proietti, G. Sforza, G. Monte, M. A. N. Ferilli, M. Balestri, M. Valeriani

#### ^1^Bambino Gesù Children's Hospital, Neuroscience, Rome, Italy

##### **Correspondence:** L. Papetti

**Questions.** What have we learned in the management of pediatric migraine over the past 5 years?

**Methods.** We interviewed the heads of the Italian pediatric headache centers and reviewed literature data from the last 5 years.

**Results.** *Diagnostic Criteria***.** The ICHD-3 still does not have a section dedicated to childhood migraine and continue to have limits when applied to developmental age. One of the most discussed points of the ICHD-3 is having again brought the minimum duration of the migraine attack to 2 hours and no longer to 1 hour as in the previous version.

*Therapy.* Preliminary results showed that the use of CGRP monoclonal appears to benefit adolescents with chronic refractory headaches. Pending monoclonal antibodies, pediatric migraine prophylaxis is still based on the use of traditional drugs and non-pharmacological therapies of which we have few controlled data. In 2017 the results of CHAMP trial were published. This study showed that there were not differences in reducing frequency of attacks in the amitriptyline, topiramate, and placebo groups. The study had limitations such as the long follow-up period, the behavioural interventions and the exclusion of severe migraines and younger children. Then it did not consider an untreated group to separate a real placebo effect from a placebo response.

*The Covid 19 pandemic.* The rest from school and reduction of stress during the lockdown of march 2020 led to a significant improvement in the headache trend also in chronic and drug resistant children. These results suggested that the management of emotional and psychological factors is mandatory for the management of headache in children and adolescents.

**Conclusions.** Few steps forward have been made for the management of pediatric headache in terms of facilitating the diagnostic process and drug treatment. The era of the Covid 19 pandemic showed us how much t management of anxiety, stress and depression are fundamental to reach the goal of a migraine control in children.

## P234 The influence of anxiety and depression on headache in adolescent migraineurs: A case-control study

### E. Jafari, M. Togha, H. Kazemizadeh

#### Tehran University of Medical Sciences, Neurology, Tehran, Iran

##### **Correspondence:** E. Jafari

**Introduction:** Migraine is a highly prevalent and disabling neurological disorder which is commonly linked to a range of psychiatric comorbidities, primarily anxiety and depression. The present study compared the frequency of anxiety and depression disorders in migraine and non-migraine adolescents and evaluated the effect of depression and anxiety on migraine characteristics.

**Methods:** In this case-control study, 234 adolescents (112 cases with migraine and 122 non-migraine adolescents) aged 13-18 years were evaluated for the presence of anxiety and depression and the influence of these factors on migraine headache. A headache questionnaire as well as the Beck Anxiety Inventory (BAI) and Children"s Depression Inventory (CDI) were completed to investigate the prevalence of headache, anxiety, and depression.

**Results:** The average age of participants was 15.77 ± 2 years in the case group and 15.39 ± 1.79 years in the control group. Among them, 19.8% were boys and 80.2% were girls. We found significantly higher levels of mild, moderate, and severe anxiety (38.4%, 23.2% and 23.2%, respectively) in the migraine group compared to the control group (24.2%, 5.8% and 10.0%. respectively). Non-depressive CDI scores were significantly lower in the migraine group (10.1%) than in the control group (29.6%). There was a significant difference between patients with moderate and severe anxiety in terms of attack frequency and duration. In addition, depressed migraineurs recorded higher attack severities and frequencies than migraineurs without depression.

**Conclusion:** Anxiety and depression are common in adolescents with migraine and can be associated with more burdensome attacks. This makes it necessary to consider anxiety and depression among adolescent migraineurs and provide a therapeutic protocol for this issue.

**Keywords**: Headache, Migraine, Depression, Anxiety, Adolescents

## P235 Abnormal eye movements in a girl with Vestibular migraine: a case report and review of the literature

### F. Farham, A. A. Okhovat

#### Tehran University of Medical Sciences, Headache Department, Tehran, Iran

##### **Correspondence:** F. Farham

Vestibular migraine (VM) is a complex disorder with an estimated prevalence of 1-3%.^1^ VM is most common cause of recurrent spontaneous vertigo.^2^ During acute VM attacks spontaneous and positional nystagmus can be seen.^3^ This case of vertigo and nystagmus may be misdiagnosed as BPPV or Meniere disease.

A 10 years old girl visited our clinic with her parents complaining of abnormal eye movement with vertigo. She had a history of headache from 3 years ago that were unilateral, in temporal region. She has photophobia. Headache were aggravated by activity and had severe intensity. After starting the headache patient had a vertigo that caused imbalance which lasts 10-60 minutes. During the vertigo her parents reported abnormal eye movement, which documented by camera. Videos showed pendular nystagmus. Patient was fully awake and aware during the attacks. Attacks occur about three times in the month and between them patient is symptom free. She had cyclic nausea and vomiting until 6 years old, that improve spontaneously. All neurologic exams was normal. In MRI she has a few nonspecific bilateral asymmetrical lesions. According to the history and IHC-3, vestibular migraine was made as diagnosis. Patient undergone treatment with Cinarizin 25 mg which resulted in reduction of attacks in 3 months follow-up (only one which was following an emotional stress).

This case illustrates that Observation of the nystagmus during a vertigo attacks is helpful for correct diagnosis. exact history taking is important for VM diagnosis, differentiate VM from BPPV, and exclusion CNS consideration.

1.Systematic review of prevalence studies and familial aggregation in vestibular migraine, Ana Paz-Tamayo et al, Frontiers in genetics, 2020

2.Diagnosis and treatment in vestibular migraine, Pinar Yalinay Dikmen, Turkish journal of Neurology,2020

3.Acute migrainous vertigo: clinical and oculographic finding, Michael von Brevern et al, Brain,2005

## P236 Allodynia and muscle sensitization of cervical muscles are not associated with neck range of motion in children and adolescents diagnosed with migraine

### J. Pradela^1^, M. Santos^1^, N. Silva^1^, F. Dach^2^, D. Bevilaqua Grossi^1^

#### ^1^University of São Paulo, Health Sciences, Ribeirão Preto, Brazil; ^2^University of São Paulo, Neuroscience and Behavior Science, Ribeirão Preto, Brazil

##### **Correspondence:** J. Pradela

**Question:** To correlate allodynia and sensitization of cervical muscles with neck mobility in children and adolescents diagnosed with migraine. **Methods:** Fifty children (CH) and adolescents (AD) diagnosed with migraine by ICHD-III were screened, of both sexes, aged between 6 and 17 years at the tertiary headache outpatient clinic. Allodynia was assessed by the adapted allodynia questionnaire based on the ICHD-III and the sensitivity of the cervical muscles by the pressure pain threshold (PPT) using a digital algometer. The active mobility of the cervical spine (ROM) was evaluated in the movements of flexion, extension, lateral flexion, and rotation by the Flexion Rotation Test (FRT), using the CROM®. **Results:** The mean age of patients was 11.7 years (SD=3.0), most of them female (n=31/62%), with a diagnosis of episodic migraine (n=32/64%), of low intensity (2.4; SD=0.6)), pulsatile quality and duration in hours (18.0; SD=22.7). More than 70% of the sample had some comorbidity associated with the diagnosis of migraine, and neurological diseases, such as epilepsy, were more prevalent in both children (25.2%) and adolescents (26.3%), followed by respiratory diseases in children (20.1%) and psychological conditions in adolescents (19.8%). Pearson's correlation values between PPT and ROM for flexion and extension (rho=0.019 to 0.550) and lateral flexion (rho=0.002 to 0.136) and between PPT and left FRT (rho=0.043 to 0.336) and right FRT (rho=0.051 to 0.336) were classified as weak to moderate and not significant. Correlations between cutaneous allodynia and ROM for flexion and extension (rho=-0.024), lateral flexion (rho=0.278) and rotation (rho=-0.038) and for right FRT (rho=-0.085) and left FRT (rho=0.182), had p>0.05. **Conclusion:** Cervical sensitization represented by allodynia and LDP appears not to be associated with cervical mobility in the infant population.

## P237 Neck-Tongue syndrome: an underrecognized and peculiar form of headache

### B. Martins, A. Costa

#### Centro Hospitalar Universitário de São João, Neurology, Porto, Portugal

##### **Correspondence:** B. Martins

**Question**: Neck-tongue syndrome (NTS) is a rare and underrecognized headache disorder, characterized by paroxysms of neck and/or occipital pain brought out by abrupt head-turning and accompanied by ipsilateral tongue symptoms.

**Methods:** Case-report

**Results:** A 31-year-old woman, hotel receptionist, with a history of alopecia and active smoking, was referred to the Neurology consultation due to monthly episodes of sharp and shooting headache since age 15, characterized as an explosive headache, intensity 10/10, lasting 3-5 seconds, in the occipital region (right predominance), precipitated by ipsilateral neck rotation. During these paroxysms, she used to have a concomitant posterior "tongue-pulling" movement, followed by numbness of the hemi-tongue ipsilateral to the headache, usually lasting less than 1 minute. Episodes of involuntary tongue movements caused discomfort and anxiety due to their social impact. She had a history of minor trauma, after headache debut; no positive family history. On general examination, she showed signs of ligamentous hyperlaxity; normal neurological examination, with no trigger points. The referred episodes were not possible to induce. The blood cell count, erythrocyte sedimentation rate, and chemistry profile were normal. Rheumatoid factor and antinuclear antibody were negative. Cerebral and cervical MRI, and skull base X-ray, were normal. Her clinical features fulfilled the ICHD-3 criteria for a diagnosis of NTS.

**Conclusions**: Our case illustrates the diagnostic delay of this underrecognized condition and the possible etiological link with ligamentous laxity leading to transient subluxation of the atlanto-axis joint. There are currently no consensus treatment guidelines; conservative management, including physiotherapy and minor cervical adjustment, is the preferred initial treatment. Consent to publish had been obtained.

## P238 Does symptomatic treatment help children and adolescents with chronic migraine?

### M. A. N. Ferilli^1^, L. Papetti^1^, F. Ursitti^1^, G. Sforza^1^, G. Monte^1^, R. Moavero^2,1^, S. Tarantino^1^, M. Proietti Checchi^1^, F. Vigevano^1^, M. Valeriani^1,3^

#### ^1^Bambino Gesù Children's Hospital, Department of Neuroscience, Rome, Italy; ^2^Tor Vergata University Hospital of Rome, Child Neurology Unit, Systems Medicine Department, Rome, Italy; ^3^Aalborg University, Neurology, Rome, Italy

##### **Correspondence:** M. A. N. Ferilli

**Background and objective**. Chronic migraine (CM) is defined in the third edition of the International Classification of Headache Disorders (ICHD-3) as the presence of headaches on 15 days or more in a month, at least 8 days showing the migraine phenotype, for more than 3 months. CM affects from 0.6% to 1.8% of children and adolescents and determines a decrease of the quality of life. Aim of this study is to analyze the type of symptomatic drugs used and their efficacy for the treatment of acute migraine attacks in pediatric patients with CM.

**Methods**. We conducted a prospective study by selecting pediatric patients diagnosed with CM in our Department. We administered a questionnaire to the parents of all our pediatric patients with CM according to ICHD-3; questions were focused on symptomatic drugs used for acute migraine attacks and their effectiveness.

**Results**. For the final analysis we considered 91 patients with CM. Only two patients responded to the initial therapy with acetaminophen and only 31 % improved with ibuprofen. Fiftythree % of patients had relief with second-line NSAIDs drugs like ketoprofen, indomethacin, naproxen. Fifty one % of patients did not respond to more than three drugs and 16 % were resistant to all acute treatments. All patients underwent prophylaxis therapy.

**Conclusions.** In our study we have shown that the drugs for acute attack are not very effective in patients with CM and that some patients do not respond to any acute treatment.

## P239 Angelman syndrome and cyclic vomiting syndrome: a new potential association - *Not all the vomiting are only vomiting!*

### G. Sforza^1^, G. Racioppi^2^, M. Armando^1^, L. Papetti^1^, F. Ursitti^1^, G. Monte^1^, M. Valeriani^1^

#### ^1^Bambino Gesù Children's Hospital, Department of Neuroscience, Rome, Italy; ^2^Bambino Gesù Children's Hospital, University Hospital Paediatric Department, Rome, Italy

##### **Correspondence:** G. Sforza and G. Racioppi

**BACKGROUND:** We well know about clinical manifestations of Angelman Syndrome (AS), including gastrointestinal issues, like vomiting, gastroesophageal reflux disease (GERD) and constipation; we report the case of a pediatric patient suffering from AS and cyclic vomiting syndrome (CVS) together. This association is not yet included among multisystemic features of the syndrome, nor put in differential diagnosis with gastrointestinal issues described.

**OBJECTIVE:** The aim of this case is to open our mind to new possible association with CVS in AS, rarely described before, and put migraine disorders and equivalents in differential diagnosis with gastrointestinal symptoms.

**METHODS:** We report a patient affected by AS who came to our Headache Centre at 8 years of age: from one year,she reported repeated episodes of general complaint with touching her head and eyes, photophobia and phonophobia, retching and vomiting for hours, with recurrence of one attack per month, rarely during sleep. Her history showed recurrent gastrointestinal disturbance; plus, familiar history was positive for migraine from paternal line. Due to her predisposition to epilepsy, she was undergone to EEG registrations, showing no epileptic alterations.

**RESULTS:** In order to explain her vomiting, we had to exclude organic causes often related to AS and, in general, to neurodevelopmental disorders. She was asymptomatic among 2 episodes, with a regular periodicity and more episodes in a brief time.

So, we were able to make diagnosis of CVS and its possible progression to migraine without aura, in accordance with ICHD-3 criteria. She was discharged with acute therapy for migraine and vomiting (ibuprofen and ondansetron).

**DISCUSSION:** We experienced diagnosis of CVS in a patient affected by AS. It seems clear that not all the repeated vomiting in AS is not necessarily part of gastrointestinal manifestation of the syndrome; even if it is only a case, we need to take into account CVS among neurological features of AS.

## P240 Clinical characteristics of children and adolescents with primary and secondary headaches in the tertiary-level of a public hospital in Brazil

### J. Pradela^1^, N. Silva^1^, M. Santos^1^, F. Dach^2^, D. Bevilaqua Grossi^1^

#### ^1^University of São Paulo, Health Sciences, Ribeirão Preto, Brazil; ^2^University of São Paulo, Neuroscience and Behavior Science, Ribeirão Preto, Brazil

##### **Correspondence:** J. Pradela

**Question:** To analyze the clinical characteristics of children (CH) and adolescents (AD) with primary and secondary headaches of tertiary-level headache outpatient clinic. **Methods:** Retrospective study, based on review of medical records of CH and AD with primary or secondary headaches between the years 2016 and 2021. Study data were obtained between the years 2016 and 2021. Sociodemographic data, medical history, clinical history, and daily routine of the child were obtained. The proportion of primary and secondary headaches in the CH and AD groups was also calculated. For continuous variables and categorical data, the chi-square test was used, considering p<0.05. **Results:** A total of 386 medical records were included, of which 206 were CH (n=112; 54.8% girls) and 178 AD (n=118; 66.8% girls). Headaches were episodic in CH (57.3%) and chronic in AD (49.7%), [X2(2)=10.001; p=0.007], of mild intensity (CR:64.1%); and strong (AD:48.5%), [X2(3)=25.802; p=0.000] in pressure (CR:64.3%) and pulsatile (AD: 52.8%), [X2(5)=14.595; p=0.012]. The chi-square test of independence showed that there is a significant association between CH and the presence of migraine [X2(5)=12,746; p=0.026], type of cesarean delivery [X2(2)=7.299;p=0.026], the use of common analgesics [X2(6)=36.690; p=0.000], discharge from the clinic after migraine treatment [X2(5)=22.225;p=0.000] and between AD and worsening of pain during physical activity [X2(3)=10.671;p=0.014], or pulsatile [X2(5)=14.595;p=0.012], and worsens during menstruation period [X2(6)=21.108;p=0.002]. **Conclusion:** In both groups, migraine was more prevalent, and females were the most affected. By associating the CH and AD groups, these clinical patterns are significantly different in several aspects.

## P241 Sars-Cov-2 and Headache in Children and adolescents

### N. Kapanadze^1^, S. Bakhtadze^1,2^, N. Geladze^1,2^, N. Khachapuridze^1,2^, T. Nadiradze^1,2^

#### ^1^Tbilisi State Mediacal University, Pediatric Neurology, Tbilisi, Georgia; ^2^Tbilisi State Mediacal University, Tbilisi, Georgia

##### **Correspondence:** N. Kapanadze

**Introduction:** Headache is identified as a common post-COVID symptom experienced by COVID-19 survivors. Post-COVID headache can be presented in a broad spectrum like headache attributed to systemic infection, increasing intensively and frequency of already existing primary headache and also late-onset new daily persistent headache.

**Objectives:** Accumulating evidence suggests that that headache onset during the presymptomatic or symptomatic phase of COVID-19 may resemble tension-type or migraine headache. Headache itself associated with a shorter symptomatic period. Our objectives were to determine correlation between Covid 19 and headache in children and adolescents.

**Methods:** We have observed 59 patients with post-COVID headache, 21 boys and 38 girls. Migraine was diagnosed in 19 patients, cluster type headache in 4 and stress-related (tension) headaches in 31. Also, Study included 46 patients with headache, but they had no Sars-Cov-2. Children"s age was 6–17 years in both group. Sars-Cov-2 was identified by *PCR test and* Headache was assessed by daily dairies and clinical examination.

**Results:** Children with SARS-CoV-2 illness and pre-existing Headaches were three-and-a-half times more likely to develop worsening of Headaches than those without pre-existing Headaches. Compared with the control group, on a daily basis the number and intensity of headache were almost more than a third in patients who had Covid 19 in past.

**Conclusions:**Cases of long coronavirus disease (COVID) headache have already been documented in adults, but literature on similar cases in children and adolescents is scant. Although the association between new daily persistent headache and COVID-19 remains unclear, these cases highlight the importance of awareness of the neurological sequelae of novel coronavirus infection in children and adolescents.

## P242 Prevalence of neck pain in migraine: A systematic review and meta-analysis

### H. M. Al-Khazali, S. Younis, S. Ashina, Z. Al-Sayegh, M. Ashina, H. W. Schytz

#### Danish Headache Center, Neurology, Glostrup, Denmark

##### **Correspondence:** H. M. Al-Khazali

**Background**: Neck pain is a frequent complaint among patients with migraine and seems to be correlated with the headache frequency. Neck pain is more common in patients with chronic migraine compared to episodic migraine. However, prevalence of neck pain in patients with migraine varies among studies.

**Objective**: To estimate the prevalence of neck pain in patients with migraine and non-headache controls in observational studies.

**Methods**: A systematic literature search on PubMed and Embase was conducted to identify studies reporting prevalence of neck pain in migraine patients. This review was conducted following the Preferred Reporting Items for Systematic Reviews and Meta-Analyses guidelines. Data was extracted by two independent investigators and results were pooled using random-effects meta-analysis. The protocol was registered with PROSPERO (CRD42021264898).

**Results**: The search identified 2490 citations of which 30 contained relevant original population based and clinic-based data. Among these, 24 studies provided data eligible for the analysis. The meta-analysis for clinic-based studies demonstrated that the pooled relative frequency of neck pain was 77.0% (95% CI: 69.0–86.4) in the migraine group and 23.2% (95% CI:18.6–28.5) in the non-headache control group. Neck pain was more frequent in patients with chronic migraine (87.0%, 95% CI: 77.0–93.0) compared to episodic migraine (77.0%, 95% CI: 69.0–84.0). Neck pain was 12 times more prevalent in migraine patients compared to non-headache controls and two times more prevalent in patients with chronic migraine compared to episodic migraine. The calculated heterogeneity (I2 values) ranged from 61.3% to 72.0%.

**Conclusion**: Neck pain is a frequent complaint among patients with migraine. The heterogeneity among the studies emphasize important aspects to consider in future research of neck pain in migraine to improve our understanding of the driving mechanisms of neck pain in a major group of migraine patients.

## P243 Characterizing Neck Pain With Headache in People With and Without Migraine: Results From the Chronic Migraine Epidemiology and Outcomes – International (CaMEO-I) Study

### M. Matharu^1^, Z. Katsarava^2^, D. C. Buse^3^, K. Sommer^4^, M. Reed^5^, K. Fanning^6^, R. Lipton^3^

#### ^1^Institute of Neurology, London, United Kingdom; ^2^Evangelical Hospital Unna, Unna, Germany; ^3^Albert Einstein College of Medicine, Bronx, NY, United States; ^4^AbbVie, Irvine, CA, United States; ^5^Vedanta Research, Chapel Hill, NC, United States; ^6^MIST Research, Wilmington, NC, United States

##### **Correspondence:** M. Matharu

**Objective:** To characterize the frequency and burden of neck pain with headache (NPWH) among individuals with and without migraine.

**Methods:** CaMEO-I was a prospective web-based survey conducted during 2021-2022 in Canada, France, Germany, Japan, United Kingdom, and United States. Respondents with ≥1 headache in the past year were divided into two groups: those meeting ICHD-3 migraine criteria based on a validated questionnaire and those with non-migraine headache (NMH). In both groups, NPWH was defined as reporting NPWH more than rarely. Within the migraine group, monthly headache days (MHDs), migraine-related disability (MIDAS), allodynia (ASC-12), depression and anxiety symptoms (PHQ-4), work productivity and activity impairment (WPAI), and acute treatment optimization (mTOQ) were evaluated in those with and without NPWH using validated measures.

**Results:** NPWH was reported by a higher proportion of respondents with migraine (63.4% [UK]–75.0% [Germany]) versus NMH (31.4% [UK]–44.8% [Germany]; **Figure 1A**). Migraine respondents with NPWH were more likely to have higher MHDs than those without NPWH in all countries. Among migraine respondents, moderate to severe MIDAS scores (MIDAS ≥11) were reported more frequently among those with NPWH (35.6% [Japan]–55.3% [Germany]) than those without NPWH (19.8% [Japan]–42.3% [Germany]; **Figure 1B**). Depression and anxiety symptoms, and allodynia were greater in respondents with NPWH versus those without (**Table**). Impairment in daily activities and while at work, work time missed, and overall work impairment were higher among those with versus without NPWH. "Poor" to "very poor" treatment optimization was reported more frequently in those with versus without NPWH.

**Conclusions:** NPWH occurred more frequently among respondents with migraine versus those with NMH. Among those with migraine, NPWH was associated with more frequent headaches and greater disability compared to those without NPWH. International differences will be discussed.

**Fig. 1 (abstract P243).**
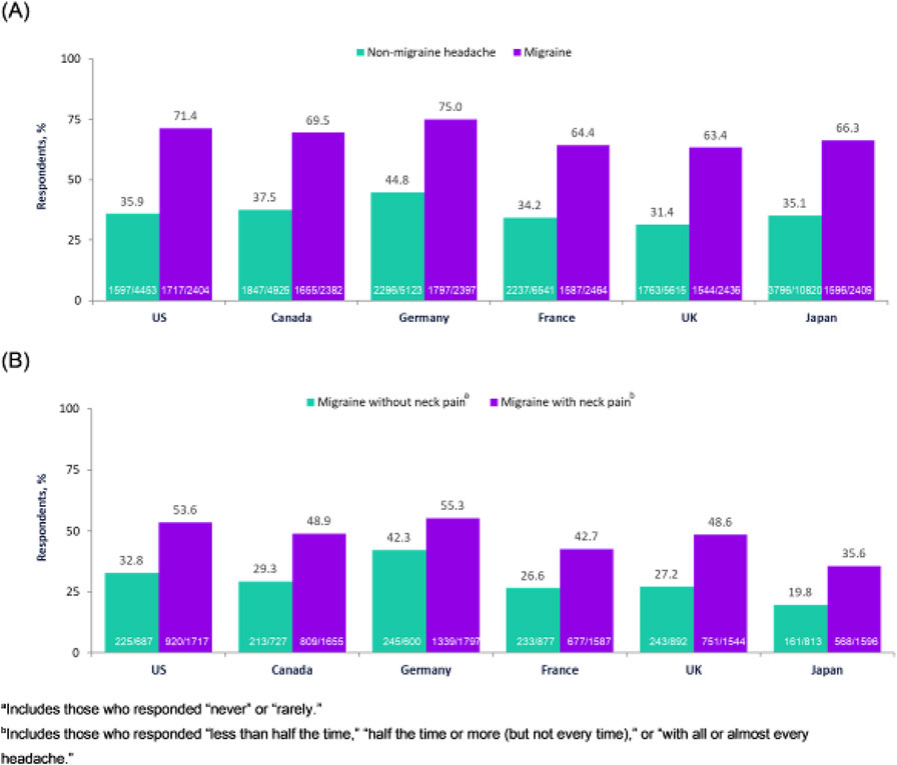
(A) Frequency of Neck Pain More Than Rarely Among Individuals With Migraine versus Non-migraine Headache; (B) Frequency of Moderate to Severe Disability Among Migraine Respondents With and Without Neck Pain With Headache

**Table 1 (abstract P243).**
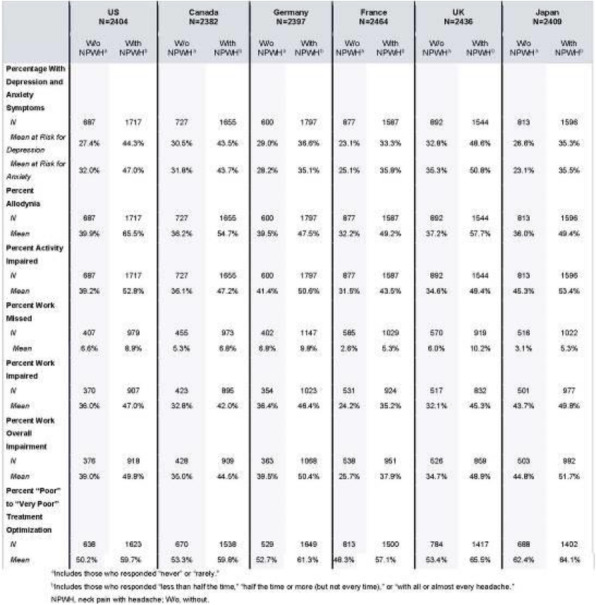
Frequency of Depression, Anxiety, Allodynia, Impairment in Daily Activity, Work Time Missed, and “Poor or Very Poor” Treatment Optimization Among Migraine Respondents With and Without Neck Pain

## P244 Interest in headache and other neurological subspecialties among neurology residents in Denmark: a nationwide survey

### M. Kristensen^1^, T. P. Do^2,1^, P. Pozo-Rosich^3,4^, F. M. Amin^5,1^

#### ^1^Danish Headache Center, Glostrup, Denmark; ^2^Danish Headache Center, Glostrup, Denmark; ^3^Vall d'Hebron University Hospital, Barcelona, Spain; ^4^Autonomous University of Barcelona, Barcelona, Spain; ^5^University of Copenhagen, Rigshospitalet, Department of Neurorehabilitation/Traumatic Brain Injury, Copenhagen, Denmark

##### **Correspondence:** T. P. Do

**Introduction:** Headache disorders constitute a leading cause of disability worldwide, but there is a consistent absence of awareness and educational activities for healthcare providers across regions. Thus, we found it timely to identify potential structural challenges and factors that may affect acquisition of knowledge of headache disorders and their management during residency.

**Methods:** We conducted a nationwide cross-sectional survey of residents in neurology in Denmark including, but not limited to, questions on interest in neurological subspecialties and disorders, adequacy of training in headache disorders, exposure to headache disorders during training including time spent on headache disorders, exposure to specialist outpatient clinics, whether their hospital have a tertiary headache clinic, training in specific procedures (anesthetic blockade, e.g., greater occipital nerve blockade, and onabotulinumtoxinA for headache), and an estimate of proportion of cases with headache amongst patients managed in the last week.

**Results:** The survey was distributed to 127 residents in Denmark between March 2022 to April 2022. Of these, 59 (47%) completed all questions of the survey. Headache disorders were the fourth most popular subspecialties amongst respondents (n= 15 [25%]). The mean number of hours spent in a course or a structured educational activity in headache disorders during residency was 12.1 (±12.9) hours. Half of respondents (n=27 [46%]) reported that they perceived their training in headache disorders to be inadequate.

**Conclusions:** Even in Denmark, a country with excellent headache services, half of residents in neurology report an inadequate training despite a higher-than-average number of hours of structured educational activities.

## P245 Characterizing opioid use in a Dutch cohort with migraine

### R. van Welie, F. van Welie, S. D. Lentsch, G. Terwindt

#### Leiden University Medical Center, Neurology, Leiden, Netherlands

##### **Correspondence:** R. van Welie

**Objective:** To investigate opioids use among migraine subjects for treatment of their headache.

**Methods**: We performed a cross-sectional study using a web-based questionnaire to assess opioid use in individuals with migraine. Primary outcome was to assess opioid use among migraine subjects for treatment of attacks in a large Dutch cohort. We also quantified opioid use (duration, type of opioids, prescriber) and compared between persons with episodic (EM) versus chronic (CM). Covariates were entered as categorical or continuous variables. Descriptive statistics, unpaired T-tests, Chi-square and Mann-Whitney U tests were used.

**Results:** The E-questionnaire was sent to our large Dutch cohort of n=6577 migraine subjects, of whom n=4047 responded, and n=3712 completed the questionnaire (response rate 56%). In total 13% of participants reported to ever have used opioids for headache. In 46% of those who used an opioid this was on one occasion, but in 27% this was for >1 month, and in 11% for >1 year. The opioids were prescribed by physicians but 2% of opioid-users indicated that they had used without a prescription. The majority of prescribing physicians were general practitioners (46%), followed by neurologists (35%), other specialists (9%), and doctors at a first aid (8%). Opioids were reported to be prescribed for acute treatment in 63% of cases, in 16% as preventive treatment and in 21% as both acute and preventive medication. Opioid use was more often in those with CM compared with EM (22% and 12%, p<0.001). Prolonged use was more often in those with CM versus EM (> 1 month: 34% and 24%; p<0.003).

**Conclusion:** Despite the fact that opioids are not effective in migraine these medications are still prescribed, even up to 22% in those who suffer from chronic migraine. Of all opioid-users 2% did so without prescription. Education for doctors and migraine subjects, and providing multimodal pain management strategies are needed to reduce opioid use in persons with migraine.

## P246 The association of innate and adaptive immunity with migraine in The Rotterdam Study: a population-based cohort study

### C. Acarsoy^1^, D. Bos^1,2^, M. K. Ikram^1,3^

#### ^1^Erasmus Medical Center, Department of Epidemiology, Rotterdam, Netherlands; ^2^Erasmus Medical Center, Department of Radiology & Nuclear Medicine, Rotterdam, Netherlands; ^3^Erasmus Medical Center, Neurology, Rotterdam, Netherlands

##### **Correspondence:** C. Acarsoy

**Objective:** Within the multifactorial etiology of migraine, accumulating evidence suggests a role for the immune system. However, the specific contribution of innate and adaptive immunity to migraine remains unclear. Hence, we investigated the association of innate and adaptive immunity with migraine. Additionally, we explored the role of the balance between the two components in migraine. **Methods:** We measured white-blood-cell type based immunity markers and calculated their derived ratios using blood samples collected during interictal periods and assessed the prevalence of migraine using a structured interview with participants of the prospective population-based Rotterdam Study. We assessed neutrophil and platelet counts as a proxy for innate and lymphocyte count as a proxy for adaptive immunity. The balance between the two components was assessed by the neutrophil-to-lymphocyte ratio (NLR), platelet-to-lymphocyte ratio (PLR) and systemic immune-inflammation index (SII). We investigated associations of blood cell counts, and their derived ratios with migraine using logistic regression models adjusting for age, sex and other variables. **Results:** Among 6593 participants (mean age 65.6 ± 11.2 years, 56.7 % female), 995 (15.1%) had migraine. We found no association between neutrophil (Odds Ratio [OR] per standard deviation increase 1.02 95% Confidence Interval [CI] 0.94-1.10), platelet (OR 1.01 CI 0.93 – 1.09) or lymphocyte counts (OR 1.01 CI 0.93 – 1.09) and migraine status. Similarly, no associations were observed between NLR (OR 1.01 CI 0.94 – 1.09), PLR (OR 1.00 CI 0.93 – 1.08) and SII (OR 1.01 CI 0.94 – 1.09) and migraine status. In the analyses with migraine subgroups, a significant association was observed between the platelet count and migraine with aura (OR 1.17, CI 1.01 – 1.35). **Conclusion:** Our results do not support the involvement of innate and adaptive immunity in migraine. Platelet count and migraine with aura relationship needs further investigation.

## P247 Headache-related disability measured with Headache impact test-6 (HIT-6) Results from an Estonian population-based survey

### M. Vaikjärv^1^, K. Braschinsky^2^, A. Raidvee^3^, M. Braschinsky^1,2^

#### ^1^Univeristy of Tartu, Department of Neurology and Neurosurgery, Tartu, Estonia; ^2^Tartu University Hospital, Neurology, Tartu, Estonia; ^3^University of Tartu, Institute of Psychology, Tartu, Estonia

##### **Correspondence:** M. Vaikjärv

Objective: The objective of this study was to use the HIT-6 questionnaire to measure the level of disability caused by various forms of headache disorders in Estonian adults, as well as to describe the relationship between HIT-6 score and characteristics including age, sex, BMI, education, urban vs rural habitat, smoking, and physical activity.

Methods: From January 2016 to May 2017, a population-based random sample study was undertaken in Estonia. The HIT-6 questionnaire was used to assess headache-related burden.

Results: The HIT-6 scores of 475 subjects were evaluated. Subjects with chronic headaches had higher HIT-6 scores than episodic headaches (60.7±9.7 and 52.6±8.9, respectively), and subjects with migrainous headaches had higher HIT-6 scores than non-migrainous headaches (56.3±8.4 and 50.1±8.8, respectively). Both differences were statistically significant. Age, sex, BMI, education, urban vs rural habitat, smoking, and physical activity had no significant effect of HIT-6 scores.

Conclusion: The HIT-score is influenced by the diagnosed headache - episodic vs chronic and migrainous vs non-migrainous - and less so by socio-demographic factors.

## P248 Headache characteristics during COVID-19 pandemic and the armed conflict

### J. Azimova^1^, K. Skorobogatykh^1^, U. Clinic^1^, A. Uzhakhov^1^, D. Korobkova^1^, M. Kukushkin^1,2^, S. Kornienko^1^, E. Mamkhegov^1^, V. Osipova^1^

#### ^1^University Headache Clinic, Moscow, Russian Federation; ^2^Institute of General Pathology and Pathophysiology, Moscow, Russian Federation

##### **Correspondence:** U. Clinic

Stress is the most significant trigger for many types of headaches. In the las two years people faced two major stressful event: Covid-19 pandemic and the armed conflict between Russia and Ukraine. The purpose of the study was to evaluate the influence of the pandemic and the war on the condition of patients who visit a specialized headache clinic.

Methods. We performed retrospective analysis of medical records of all headache patients ≥16 yo who visited the University Headache Clinic from April 01 to June 30 in 2019, 2021 and 2022 was carried out. The diagnosis of the headache and comorbid mental disorders, was made according to the criteria of ICHD-3 (2018) and ICD-10.

Results. The analysis included 849 people: 153 patients in 2019, 264 in 2021 and 432 in 2022. The migraine and tension type headache (TTH) characteristics did not change in 2021 and 2022 years compared with 2019 year. In 2021, the proportion of patients with generalized anxiety disorder has increased from 28,8% (24 patients) to 44,9% (119 patients), p=0,001. Proportion of patients with anxiety remains high in 2022 (43,6%, 179 patients). Proportion of patients with depression didn"t differ significantly between 2019 (32%, 49 patients) and 2021 (28,7%, 76 patients) , p=0,5. In 2022, the proportion of patients with depression significantly increased from 28,7% (76 patients) to 43,9% (188 patients), p=0,0001, The main symptoms of depression were anhedonia, lack of internal energy, and feeling of guilt. The proportion of patients with a first depressive episode has increased significantly from 2,7% (7 patients) to 21,3% (31 patients), p=0,0001.

Conclusions. Significant stressful events had an impact on comorbid psychiatric disorders in primary headache disorders. Covid-19 pandemic led to increase of comorbid anxiety and the war resulted in significantly increased comorbid depression among Russian headache patients

## P249 Visual aura in non-migrainous headache: a population study

### S. J. Kim^1^, S. H. Lee^1^, H. J. Lee^1^, S. Cho^1^, W. Lee^2^, M. K. Chu^1^

#### ^1^Yonsei University College of Medicine, Neurology, Seoul, South Korea; ^2^Yongin Severance Hospital, Neurolohy, Yongin, South Korea

##### **Correspondence:** M. K. Chu

**Question:** Although aura symptoms usually have been described in association with migraine, their occurrence has been observed with other types of headaches. Studies analyzing the aura in migraine show that visual aura (VA) represents 98% of the symptoms. Nevertheless, no study has reported VA in non-migrainous headache in a population-based setting.

**Methods:** The present study used the baseline data of the Circannual Change in Headache and Sleep study, which was a nation-wide and population-based survey. We defined migraine and probable migraine as migrainous headache by combining them. Therefore, non-migrainous headache was defined as a headache case other than migrainous headache. VA was assessed using the self-administered visual aura rating scale which is a validated instrument for assessing VA.

**Results:** Of 3,030 participants, 1,431 (47.2%) and 507 (16.7%) were classified as having non-migrainous and migrainous headaches during the previous year, respectively. VA was reported in 406 (20.9%) participants with headache. The prevalence of VA was significantly lower in non-migrainous headache than that in migrainous headache (26.0% [132/507] vs. 14.5% [207/1431], *p*<0.001). The prevalence of VA did not significantly differ between women and men (14.6% [110/751] vs. 14.3% [97/680], *p*=0.837). Headache days/month (median and 25-75% percentiles, 2.0 [0.4-5.0] vs 2.0 [1.0-3.0], *p*<0.001) and disability (migraine disability assessment, 6.0 [3.0-16.0] vs. 2.0 [0.0-7.0], *p*<0.001) were significantly higher in non-migrainous headache with VA than those without VA.

**Conclusions:** VA was prevalent among participants with non-migrainous headache. Some clinical characteristics of non-migrainous headache with VA were more severe than those in non-migrainous headache without VA

## P250 Barriers and gaps in headache education: a national cross-sectional survey of neurology residents in Denmark

### T. P. Do^1,2^, M. Dømgaard^2^, S. Stefansen^2^, E. S. Kristoffersen^3^, M. Ashina^1,2^, J. M. Hansen^2^

#### ^1^Danish Headache Center, Glostrup, Denmark; ^2^Danish Headache Center, Glostrup, Denmark; ^3^Akershus University Hospital, Lørenskog, Norway

##### **Correspondence:** T. P. Do

**Background:** A major barrier to adequate headache care is the relative lack of formal education and training of healthcare professionals. Concerted efforts should be made to pinpoint major gaps in knowledge in healthcare professionals to facilitate better educational policies in headache training. The aim of this study was to identify deficiencies and barriers in headache training among residents in neurology in Denmark.

**Methods:** We conducted a national cross-sectional survey of residents in neurology in Denmark from April 2019 to September 2019. The survey included questions on participant demographics, knowledge of and barriers in headache disorders, guidelines and diagnostic tools usage, contact with primary and tertiary care, medication overuse, and non-pharmacological interventions. Furthermore, respondents were asked to provide a ranked list from most to least interesting for six sub-specializations/disorders, i.e., cerebrovascular disease, dementia, epilepsy, headache, multiple sclerosis, Parkinson's disease.

**Results:** Sixty (40%) out of estimated a population of ~150 resident across Denmark accepted the invitation. Of these, 54/60 (90%) completed the survey. Although two-thirds, 35/54 (65%), of the respondents had prior formalized training in headache disorders, we identified gaps in all explored domains including diagnosis, management, and referral patterns. Particularly, there was an inconsistent use of guidelines and diagnostic criteria from the Danish Headache Society (2.74 (± 1.14)), the Danish Neurological Society (3.15 (± 0.86)), and the International Classification of Headache Disorders (2.33 (± 1.08)); 1: never/have not heard of, 4: always. Headache was ranked second to last out of six sub-specializations in interest.

**Conclusions:** Overall knowledge on headache disorders amongst neurology residents in Denmark do not meet the expectations set out by national and international recommendations.

## P251 Post Covid Headache, in 510 cases presented in Regional Hospital Durres, Albania in period November 2020 - May 2022

### E. Harizi (Shemsi)^1^, K. Shemsi^2^, F. Domi^3^

#### ^1^Regional Hospital Durres, Neurology, Durres, Albania; ^2^University Of Medicine Tirana, Medicine, Tirana, Albania; ^3^Regional Hospital Durres, Emergency, Durres, Albania

##### **Correspondence:** E. Harizi (Shemsi)

**Background:** SARSCoV2 the virus responsible for the COVID19 pandemic had not only respiratory symptoms,but also neurological symptoms, and headache is a frequent complaint. Pathophysiology of headache in the context of COVID 19 has some mechanisms that can be involved in persistence of headache after acute stage of the disease. These mechanisms include systemic inflammation that can stimulate cytokine storm,can activate trigeminovascular system at the meninges,and in some patients this inflammatory response may be sustained after infection and can play role at post Covid headache. **Methods:**We have seen 510 patients that have been presented at emergency department and neurology consult at SRDwith headache after covid 19(2-10months after infections).15%of patients had severe covid infections with respiratory insuficience and have been recovered in hospital (76 patients)and85%(434 patients) have been treated ambulatory.the most of patients had bilateral frontal headache (52%)and holocranic headache (22%), and hemicranic migraine type (26%). Most of patients had oppressive pain,72% (367 patients) had moderate headache and 28%(143patients) had severe continous headache. Middle age of patients was52years old and65%were female (331) and 35%male (179) and mean time of headache was 3.6month from all patients 30%(153 patients) have been known with primary headache, and76%had migraine (116 patients,78 female,38 male),22%tensive type headache (34 patients, 20 female and 14 male) and2% had cluster headache (3patients were mens) .From all 510 of patients 45%(230) had also other post covid symptoms like dizziness,memory problems, insomnia,brain fog,depression and anxiouse state etc. C**onclusions:**The mechanisms of persistent headache for months after covid 19 infections means to be stimulate by inflammatory mechanisms with stimulation of the trigeminovascular system,andCGRP (calcitoni gene-related peptide) released by pulmonary endings nerve during viral infections may stimulate migraine.

## P252 Inpatient headache referrals to acute neurology service – a review of referral patterns

### S. Kannan^1^, S. Chhetri^2^

#### ^1^University of Central Lancashire, Preston, United Kingdom; ^2^Lancashire Teaching Hospitals NHS Foundation Trust, Neurology, Preston, United Kingdom

##### **Correspondence:** S. Kannan

**Objective:** Headache disorders constitute 20-30% of inpatient referrals to neurology services in UK. The aim of this study was to identify the headache phenotypes referred for inpatient neurology opinion.

**Methodology:** All inpatient referrals to the neurology on call service at the Royal Preston Hospital in the year 2019 were reviewed. Cases were identified from the clinical database and categorised into primary and secondary types, according to the International Headache Society classification.

**Results:** A total of 1114 inpatient referrals were made to neurology services in 2019, of which 255 cases were for a headache disorder (22.9%). Primary headache syndromes made up 158 cases (61.9%), with the remaining cases diagnosed as secondary headache (38.1%). Most primary headaches were diagnosed as migraines (82.3%) followed by tension-type headache (7.6%); 70 cases (44.3%) had a pre-existing diagnosis of primary headache disorder. The major cause of secondary headache was TIA/stroke and idiopathic intracranial hypertension (16.5% each) followed by subarachnoid haemorrhage (11.3%) and infection (7.2%). Both primary and secondary headaches were higher in females (70.0% & 57.7%) compared to males (30.0% & 42.3%).

Majority of patients (74.2%) had been investigated with neuroimaging prior to the referral. Majority received both CT & MRI scans (39.8%), followed by CT only (33.3%) and MRI only (15.7%); 11.2% of patients received no scan.

**Conclusion:** These findings indicate that a significant proportion of patients with headache referred for inpatient neurology opinion have a primary headache disorder.

## P253 Primary Headaches - Underestimated problem in Armenia

### H. Vekilyan^1,2^, H. Manvelyan^2^

#### ^1^2nd Medical Union, Neurology, Yerevan, Armenia; ^2^YSMU, Neurology, Yerevan, Armenia

##### **Correspondence:** H. Vekilyan

Objectives

According to data of WHO primary headaches have substantial burden on patients and their family life and also, they consist serious social-economic problem for all world. This is why many studies addressed toward investigation of different types of primary headaches.

Aim

Aim of our study was investigation of prevalence of different types of primary headaches (tension type, migraine, trigeminal-autonomic cephalalgias) between patient who addressed to neurologist because of headache.

Material and Methods

We included 150 patients (113 women/ 37 men) with different primary headaches (migraine, tension type and trigeminal-autonomic cephalgia"s) who addressed at Neurology department of 2-nd Medical Union. Age of participants was 18-60 years All patients was estimated by neurologist specialized in headache field and diagnosis was done according to ICHD 3 diagnostic criteria.

Results

Data analysis revealed that 90 (60%) has migraine, 47 (31%) tension type, 7 (5%) cluster headache, 4 (3%) paroxysmal hemicrania, 1 (1%) hemicrania continua and 1 (1%) SUNCT –syndrom. Main part of patient (91 patient) was adressed to doctors previously (68 from which adressed to neurologists) and unfortunatly main part of them was misdiagnosed.

Conclusion

All together our data a little bit different from such data of international studies, but we suppose that mean cause of this , that we used choosen population and not a random sample. By analyzing our data, we conclude that in our region there is serious underestimation of all types of primary headaches as among different specialists and also among people. Main part of patients didn"t receive correct diagnosis and further corresponding treatment for many years. We need to increase attention toward headaches and spread knowledges among people and specialists and realize more detailed investigations for estimation of real prevalence of different type of primary headaches in Armenia.

## P254 Epidemiological Investigation of Headache among Medical Students

### O. Yaremchuk, I. Yaremchuk

#### Bukovinian State Medical University, Department of Nervous Diseases, Psychiatry and Medical Psychology, Chernivtsi, Ukraine

##### **Correspondence:** O. Yaremchuk

**Objective:** to investigate associations between lifestyle, physical activity and headache in medical students.

**Methods:** We conducted a survey of 192 students of Bukovinian State Medical University aged 19 to 26 years by using specially designed questionnaires on intake of meals, coffee, nonalcoholic and alcoholic drinks, smoking, and physical activity. By making a diagnosis were used the classification and diagnostic criterions created by International Headache Society, 2018. The intensity of headache was investigated by visual-analog scale. Results are expressed in numbers and percentage.

**Results:** The availability of headache was founded by 157 (81.8%) students. Tension headache was evidenced most commonly (by 58.6% students), migraines – by 9.6% and other types of headaches (vascular, liquor-dynamic, neuropathological etc.) – by 31.8% students. The intensity of headache-syndrome was 50-60% (5-6 points) in accordance with visual-analogical scale. Most of students (89.2%) noticed negative influence of headache over their professional and daily activity. Level of physical activity assessment showed that only 6.3% of students did daily exercise, 26.0% did regular exercise twice-three times a week, 35.4% did not have any form of exercise; and the rest did mild to moderate form of exercise infrequently. The most common exercise was aerobic workouts: walking followed by running (38.7%), fitness (25.8%%), dances (19.3%) and swimming (6.5%). Low physical activity was associated with higher prevalence of tension headache.

**Conclusion:** By means of the research we found high prevalence of headaches among medical students. Students with any type of headache might benefit from regular physical activity and low consumption of alcoholic drinks, while for migraine patients a low consumption of coffee should additionally be recommended. This problem needs further inquiry and active correction for improvement in quality of life, professional and personal succeeding of medical students.

## P255 Patients with headaches are common in Danish chiropractic practice

### K. Dissing^1^, R. Jensen^1,2^, H. Christensen^1^, H. Lauridsen^2^

#### ^1^Chiropractic Knowledge Hub, Odense M, Denmark; ^2^University Of Southern Denmark, Department of Sports Science and Clinical Biomechanics, Odense, Denmark

##### **Correspondence:** K. Dissing

Question

In Denmark, chiropractors treat patients with headaches but there is limited knowledge of how often patients seek care for headaches and what types of headaches are treated. The aims of this study were therefore i) to estimate the prevalence of consultations with headaches in chiropractic practice in Denmark, and ii) to investigate the headache types and diagnoses used.

Methods

Data were collected by Danish chiropractors who were invited by e-mail, social media, and a booklet. Participating chiropractors registered diagnosis on consecutive headache patients for 20 workdays during May and June 2022. For all headache patients, a novel logbook system was used to register the headache diagnoses as predefined by the International Headache Society together with age, and reason for consultation.

Results

In total, 67 chiropractors agreed to participate. The participants had an average of 18 headache and non-headache consultations per day and of these, 16% (SD 10) were patients with headaches. The mean age of the patients with headaches was 41.4 years (SD 14.4, range 5.5-90.3 years). Of the headache patients, 14% were classified as migraine, 62% as tension-type headaches, and 42% as cervicogenic headaches. Among patients with headaches, it was the primary reason for 42% of the consultations and the secondary reason for 54% (it was not reported for 4%).

Conclusions

On average, 16% of the consultations in Danish chiropractic practice concern patients with headaches, and for almost half of the headache patients, it was the primary reason for care seeking. There is limited knowledge of the types of treatments provided and the clinical course of headache patients in primary care. Therefore, it is paramount to explore the management and treatment provided in chiropractic practice and to investigate the clinical course and treatment pathways to the benefit of the patients.

## P256 Migraine Should Be First Focus on Headache Education in the Middle East, Asia, and Africa: A subgroup analysis of the Head-MENAA Study

### H. GENÇ^1^, H. Bolay^2^, D. Uludüz^3^, B. Baykan^4^, N. Kissani^5^, O. Luvsannorov^6^, I. Ü. Çevik^7^, M. Togha^8^, A. A. Özdemir^9^, A. Özge^10^

#### ^1^SBU Van Education and Research Hospital, Neurology, Van, Turkey; ^2^Gazi University, Neurology, Ankara, Turkey; ^3^Istanbul University, Cerrahpaşa Medicine of school, Istanbul, Turkey; ^4^Istanbul University, Istanbul Medicine of school, Neurology, Istanbul, Turkey; ^5^Mohamed VI Teaching Hospital, Neurology, Marrakech, Morocco; ^6^Mongolian National University of Medical Sciences, Neurology, Ulaanbaatar, Mongolia; ^7^Hacettepe University, Neurology, Ankara, Turkey; ^8^Tehran University of Medical Sciences, Neurology, Tehran, Iran; ^9^Mersin University, Biostatistics and Medical Informatics, Mersin, Turkey; ^10^Mersin University, Neurology, Mersin, Turkey and the Head-MENAA study group

##### **Correspondence:** H. GENÇ

**Question**

Migraine is one of the most common disorders that cause disability and affects 12-15% of the general population. Migraine alone is responsible for almost 3 % of disabilities attributable to a specific disease worldwide. We aim that determine how much the global burden of migraine is reflected in headache clinics in Turkey, the Middle East, Asia, and Africa and to define the differences between regions in this study.

**Methods**

In this cross-sectional multicenter international study, sixty-eight headache specialists from 12 countries evaluated headache patients who applied to neurology clinics. Researchers recruited patients on different weekdays selected by the research randomizer program for five consecutive weeks in April and May. Researchers used the Head-MENAA study questionnaire and ICHD-3 criteria when they evaluated patients.

**Results**

A total of 3454 headache patients were admitted out of 12043 who applied to neurology clinics on chosen days. While 15.6% of the patients who applied to the outpatient clinic had migraine, 53.2% of the headache patients had migraine. 82.5% of migraineurs were female; the mean age was 39.8±12.57 years. Migraine with aura was observed in 10.9%, chronic migraine in 26.3%, complications of migraine in 0.6%, probable migraine in 4.8%, and episodic syndromes that may be associated with migraine in 2% of migraineurs. The mean total pain duration of the patients was 6.27±8.76 years and the pain duration in the last month was 12.02±9.97 days. The differences by region are also summarized in **Table 1**.

**Conclusion**

According to our study, migraine is the most common primary headache type among the patients who applied to neurology clinics. This burden of disease; is an entity that needs to be resolved and be faced by neurologists and the health system. The directive of our study is substantial in terms of giving a status report to plan the issues that should be done at every step, from education to medical applications in the control process of the disease.

**Keywords: Head-MENAA study, migraine, global burden, neurology clinics, education**

**Table 1 (abstract A256).**
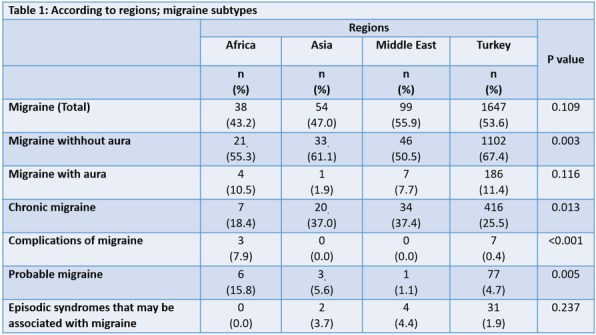
According to regions; migraine subtypes

## P257 Switching from anti-CGRP-receptor mAb (erenumab) to an anti-CGRP-molecule mAbs (fremanezumab) in chronic migraine: results of a real-world study

### V. Caponnetto^1,2^, B. Hill^2^, M. Murphy^2^, O. Ighavini^2^, J. Briscoe^2^, R. Arruda^3^, A. P. Andreou^2,4,5^, G. Lambru^2,4^

#### ^1^University of L'Aquila, Department of Applied Clinical Sciences and Biotechnology, L'Aquila, Italy; ^2^Guy's and St Thomas' NHS Foundation Trust, Headache Centre, London, United Kingdom; ^3^University of São Paulo, MSc Department of Neuroscience and Behavioural Science, Ribeirao Preto, São Paulo, Brazil; ^4^King's College London, Institute of Psychiatry, Psychology and Neuroscience, London, United Kingdom; ^5^King's College London, Headache Research-Wolfson CARD, Institute of Psychology, Psychiatry and Neuroscience, London, United Kingdom

##### **Correspondence:** V. Caponnetto

**Question.** Is a monoclonal antibody (MAB) against circulating CGRP (calcitonin gene related peptide) effective in chronic migraine (CM) patients who did not respond at all or sufficiently to a MAB against CGRP receptor?

**Methods.** This is a prospective audit conducted at the Headache Centre at Guy"s and St Thomas" Hospital.

We collected demographic and clinical data on patients who were exposed and eventually discontinued erenumab and were subsequently switched to fremanezumab. The main outcomes of this analysis include percentage of responders to Fremanezumab (reduction of monthly migraine days of at least 30% compared to baseline) as per NICE UK guidance at month 3 and 6.

**Results.** Their demographic and clinical details of the 33 patients included are reported in Table 1. Patients received a median of 15 (IQR = 7-21) erenumab injections. After discontinuing erenumab, patients received the first dose of fremanezumab after a median of 14 (IQR 6-54) weeks. After 3 months of treatment with fremanezumab, 25 patients (75.8%) were not responders, while 8 patients (24.2%) were responders. Their response was sustained at month 6. Of the non-responders at month 3, 16 patients discontinued the treatment, nine patients continued until month 6 and two of them became responders at month 6. Non-responders to erenumab and fremanezumab tried further treatments (Figure 1).

**Conclusions.** Our preliminary analysis showed that 24.2% of CM patients who fail to respond to erenumab, can respond to fremanezumab. Furthermore, a small proportion of patients who did not respond to Fremanezumab at month 3 but continued to month 6, became responders (22%), increasing the overall percentage of responders to 30.3% after 6 months of treatment. Our initial data suggests that switching from a MAB against CGRP receptor to a MAB against circulating CGRP may have a beneficial effect in a subgroup of otherwise refractory patients.

**Table 1 (abstract A257).**
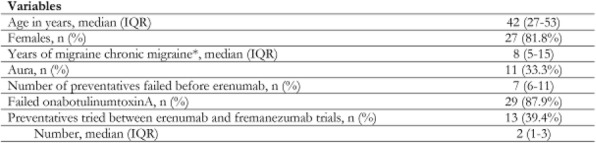
Sample characteristics (n=33). *missing data

**Fig. 1 (abstract A257).**
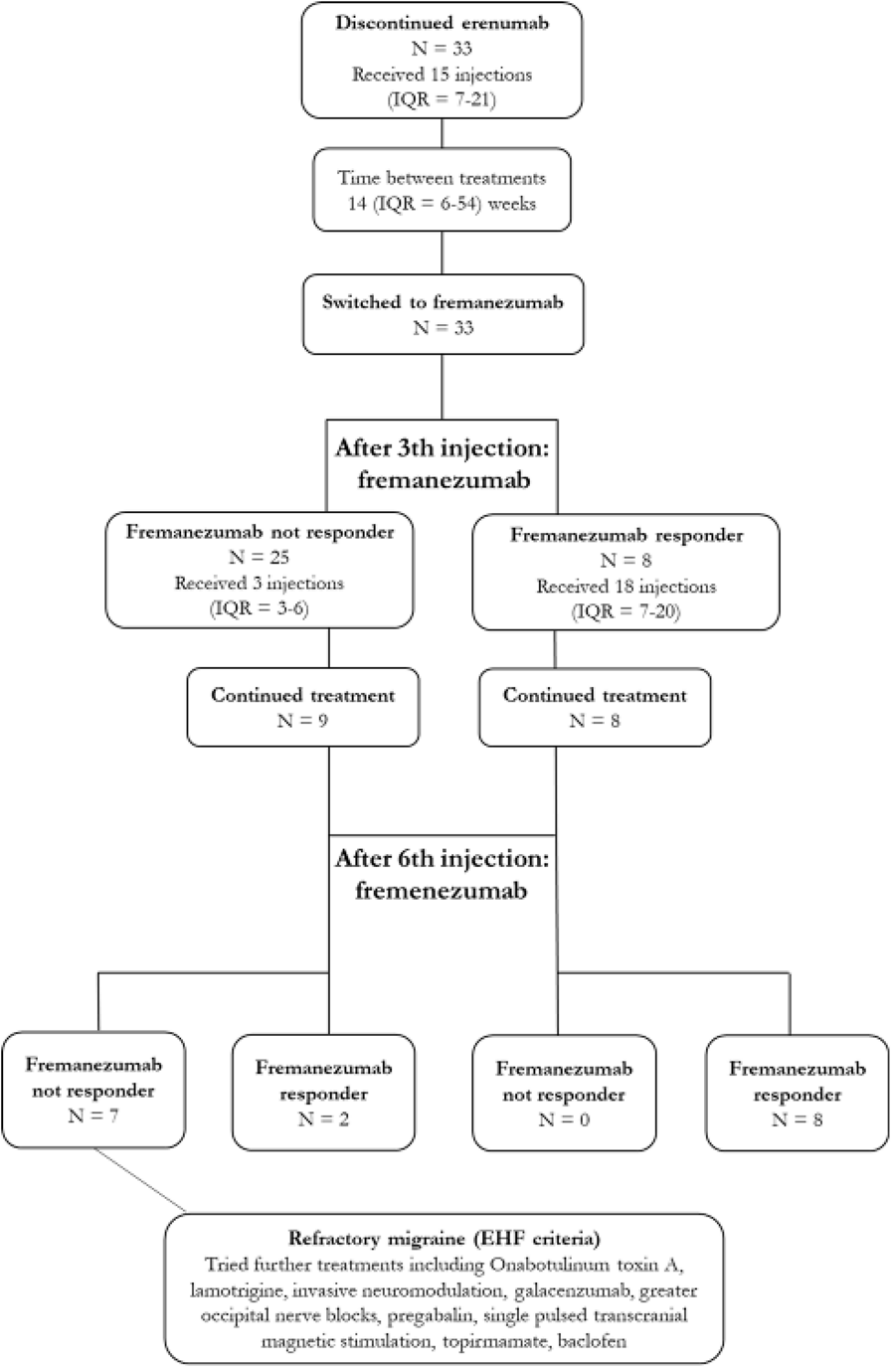
Patients’ pathway with MABS. Number of infections and time between treatments are expressed in median

## P258 Real-World fremanezumab experience from headache clinic in Moscow

### J. Azimova^1^, K. Skorobogatykh^1^, A. Uzhakhov^1^, N. Vashchenko^1^, M. Kukushkin^1,2^, D. Korobkova^1^, S. Kornienko^1^, E. Mamkhegov^1^

#### ^1^University Headache Clinic, Moscow, Russian Federation; ^2^Institute of General Pathology and Pathophysiology, Moscow, Russian Federation

##### **Correspondence:** A. Uzhakhov

Fremanezumab has demonstrated to be effective and safe for migraine prevention in randomized, placebo-controlled trials. Real-life studies are needed to evaluate effects in patients in routine practice.

Methods. This retrospective, observational study included 1-month baseline and 6- and 12-month follow-up. Patients with migraine (M) (ICHD-3. 2018) visited University Headache Clinic from 01.09.2020 till 01.09.2022 and were given subcutaneous fremanezumab were included. All included patients were keeping E-headache diary "Migrebot" 1 month prior the first injection and then all the treatment period. Primary study endpoint was the change in monthly migraine days (MMD) from baseline to 12 weeks of the treatment.

Results. We included 113 patients (38,5±11,0 y.o. 91,2% - women). 36,3% had episodic M, 63,7% - chronic. 52% had medication overuse headache. 32,7% previously failed ≥2 classes of preventive treatment. 31.1% were naïve (fremanezumab was the first prevention). Only 1 patient preferred 675mg once per 3 months". 47,3% had depression, 45,5% had somatic disorders including allergy and arterial hypertension. The change MDD was -3,7 days to 12 weeks (р<0,001), and -9.0 to 12 month. ≥50% response rate was 82,3%. 76,6% of the responders had ≥50% MDD reduction at the first treatment month. Predictors of efficacy were episodic M, response to previous therapy, and good response to triptans. 20% of the responders had significant MDD increasing during the treatment, usually on the 4th or 6th month. They were advised to continue fremanezumab or other preventives were added. After continuing with fremanezumab, MDD became less frequent again after 3 months. 11,5% had adverse events: local allergic reactions and triptans efficacy reduction.

Conclusions. Our study reproduces data from studies in other countries. However, in Russia, when prescribing fremanezumab, ≥2 treatment fails are not required. Therefore, the ≥50% response rate is slightly better than in other countries.

## P259 Discontinuation after one-year of treatment with anti-CGRP antibodies did not provide long-term sustained response without therapy

### L. F. Iannone, A. Chiarugi, D. Fattori, F. De Cesaris, P. Geppetti

#### Università degli Studi di Firenze, Health Sciences, Florence, Italy

##### **Correspondence:** L. F. Iannone

**Question:** To assess the long-term effects of discontinuation and retreatment of anti-CGRP mAbs in resistant chronic migraine (CM) patients.

**Methods:** A monocentric prospective cohort study, enrolling 53 severe (resistant to ≥3 preventive treatments) CM patients (96.2% with medication-overuse [MO]), treated with erenumab, galcanezumab or fremanezumab for 12-months, who discontinued and re-started treatment (fig. 1). The primary outcome was the percentage of patients that maintained a sustained clinical response after six months of discontinuation. The clinical effectiveness was evaluated using monthly migraine days (MMDs), response rates and acute medications use. Secondary outcomes were the effect of re-treatment up to three months, using the same parameters reported for the primary outcome.

**Results:** After 6 months of discontinuation only 8 patients (15.1%) achieved a sustained response without treatment. At month-3 after discontinuation, most patients (38, 71.7%) had already restarted treatment, mainly after the mandatory period of discontinuation (1 to 3 months [34, 64.2%]) (fig. 2). Patients with a sustained response compared to patients who restarted therapy showed less MO at baseline (75% vs 100% p=0.02) and reduced MMDs (10.6±7.8 vs 3.8±2.4, p=0.010), days with analgesic use (9.8±7.7 vs 3.6 ±2.6, p=0.014) and lower MIDAS score (24.2±24.6 vs 7.8±16.3, p=0.001) at month-12 of treatment, respectively. Patients re-treated for 3-months (n=39, 73.5%), reported an amelioration in all outcome measures regaining a response similar to that observed at the end of treatment. However, 6 patients (15.3%) did not show any amelioration during retreatment, and one patient withdrawn treatment.

**Conclusions:** Discontinuing treatment after 12 months did not provide long term benefits and appeared unnecessary in most patients. Two small subgroups of patients reported sustained benefit during discontinuation or, contrariwise, a worsening in MMDs during the second treatment cycle.

**Fig. 1 (abstract P259).**
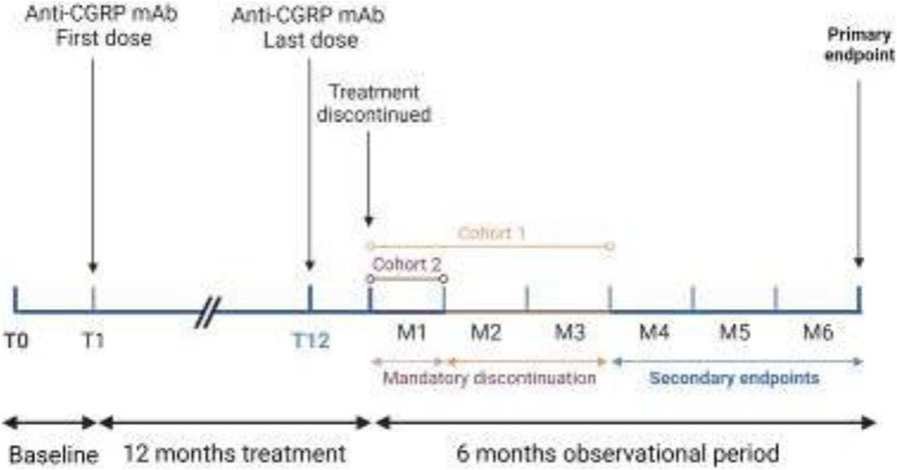
See text for description.

**Fig. 2 (abstract P259).**
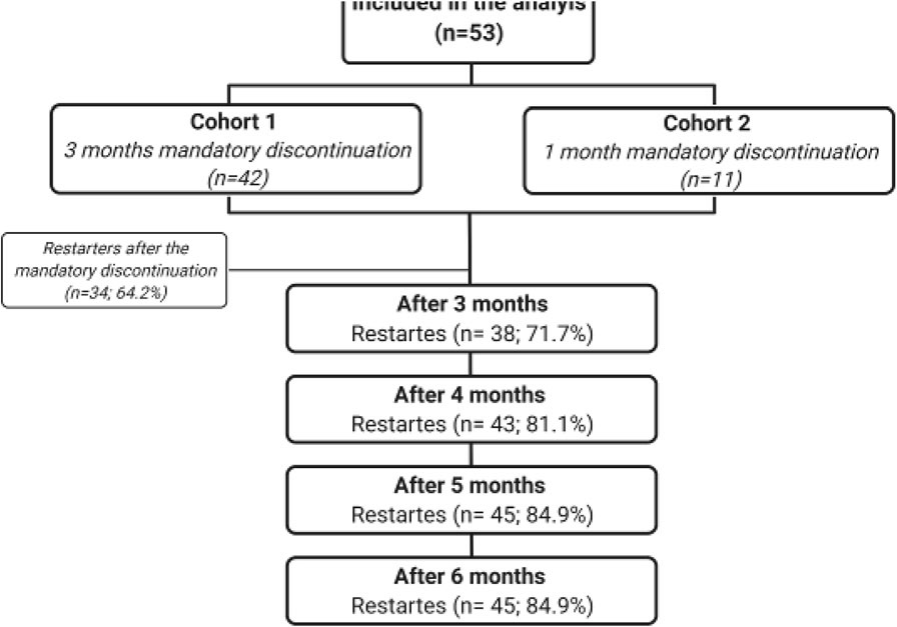
See text for description.

## P260 Anti-CGRP monoclonal antibodies and peripheral sensitization: a study on Pressure Pain Threshold in migraineurs

### G. Garascia^1^, M. Deodato^2,3,4^, L. D'Acunto^1^, A. Granato^1^, F. Biaduzzini^1^, P. Manganotti^5^

#### ^1^University of Trieste, Neurology Unit, Headache Centre, Department of Medical, Surgical and Health Sciences, Cattinara University Hospital, Trieste, Italy; ^2^University of Trieste, Department of Life Science, Trieste, Italy; ^3^University of Trieste, Department of Medical, Surgical and Health Sciences, Trieste, Italy; ^4^Azienda Sanitaria Universitaria Giuliano Isontina, Trieste, Italy; ^5^University of Trieste, Department of Medical, Surgical and Health Sciences, Cattinara University Hospital, Trieste, Italy

##### **Correspondence:** G. Garascia

**Objective**: Peripheral sensitization consisting of reduction of Pressure Pain Threshold (PPT) is related to migraine. Aim of this study was to investigate the neurophysiological effect of anti-CGRP monoclonal antibodies (mAbs) on PPT as modulation of peripheral sensitization in migraineurs.

**Methods:** An observational cohort study in migraineurs without aura treated with anti-CGRP mAbs was performed. Type and doses of anti-CGRP mAbs were chosen by a headache expert. All patients underwent a PPT analysis, conducted according to the Andersen"s standardized guidelines of the PPT assessment over craniofacial muscles. We tested five muscles of the trigemino-cervical-complex and one far from this area. PPT values of all muscles of each area were measured at baseline (t0) and after 3 (t1) and 4 (t2) months after the first injection of the anti-CGRP mAb. Data were compared with PPT in healthy controls. Data were analysed with GraphPad InStat 3.06.

**Results:** Eleven migraineurs and 11 healthy controls (mean age 43±15; F=63.6%) were enrolled. Patients were diagnosed as high-frequency episodic migraine (45.5%) or chronic migraine (54.5%), and they were treated with erenumab 140 mg (63.6%), fremanezumab (27.3%) or galcanezumab (9.1%). All migraine outcomes improved at t1 and t2 (migraine days/month: 18.2±6.9 t0 vs 8±4.7 t1 vs 6.1±3.8 t2; severe hours/month: 28.1±40.7 t0 vs 1.6±2.9 t1 vs 3.7±5.6 t2; MIDAS: 100±25 t0 vs 21±15.4 t1). At t0, migraineurs showed a significant lower PPT respect to controls in all muscles, except in the left temporalisandprocerus. PPT increased in all migraineurs" muscles at t1 and t2 without significant differences between migraineurs and healthy controls.

**Conclusion**: High-frequency episodic migraine and chronic migraine have lower PPT in cephalic and extra-cephalic muscles compared to healthy controls. Treatment with anti-CGRP mAbs normalizes migraineurs" PPT, that is related to the improvement of headache.

## P261 Clinical characteristics, efficacy and safety of patients older than 65 years with the use of anti-CGRP drugs in the central region of Spain

### A. Gonzalez-Martinez^1^, J. Pagán^2^, A. Sanz^3^, D. García-Azorín^4^, J. Rodríguez-Vico^5^, A. Jaimes^5^, A. Gómez García^5^, J. Díaz de Terán^6^, M. Sastre Real^6^, N. González-García^7^, J. Porta-Etessam^7^, S. Quintas^8^, P. Heredia^8^, J. Casas Limón^9^, G. Latorre^10^, C. Calle^10^, Á. Sierra-Mencía^11^, Á. L. Guerrero Peral^11^, C. Trevino-Peinado^1^, A. B. Gago-Veiga^8^

#### ^1^Hospital Severo Ochoa, Neurology, Leganés, Spain; ^2^Universidad Politécnica de Madrid, Electronic Engineering Department, Madrid, Spain; ^3^Unidad de Análisis de datos, Instituto de Investigación Sanitaria (IIS-Princesa), Hospital Universitario de la Princesa, Madrid, Spain; ^4^University Hospital of Valladolid, Neurology, Madrid, Spain; ^5^Fundación Jiménez Díaz, Neurology, Madrid, Spain; ^6^University Hospital La Paz, Neurology, Madrid, Spain; ^7^Hospital Universitario Clínico San Carlos, Neurology, Madrid, Spain; ^8^Hospital Universitario de La Princesa & Instituto de Investigación Sanitaria Princesa (IIS-Princesa), Neurology, Madrid, Spain; ^9^Hospital Universitario Fundación de Alcorcón, Neurology, Alcorcón, Spain; ^10^Hospital Universitario de Fuenlabrada, Neurology, Fuenlabrada, Spain; ^11^University Hospital of Valladolid, Neurology, Valladolid, Spain

##### **Correspondence:** A. Gonzalez-Martinez

**Objectives:** Most clinical trials evaluating anti-CGRP antibodies have demonstrated their effectiveness and safety in chronic and high-frequency episodic migraine in patients under 65 years of age. The main objective of this study is to describe the clinical characteristics, effectiveness and safety of antiCGRP in patients older than 65 years.

**Material and methods:** Retrospective observational study nested in a prospectively collected multicenter cohort of patients older than 65 years with chronic migraine or high-frequency episodic migraine (CIC-3) treated with anti-CGRP (erenumab, galcanezumab or fremanezumab). Demographic and clinical variables are collected, response as a reduction in the number of days of headache (DCM) and/or migraine (DMM) monthly at 3, 6 and 12 months, and the presence of adverse effects.

**Results:** 43 patients, mean age 70(SD:3.6) years, 39/43(90.7%) women, 29/43(67%) chronic migraine, migraine evolution time of 45(SD:15.2) years. , years of chronicity 14(10.4), 9.5(SD:3.8) preventive treatments, 17/43(39.5%) psychiatric comorbidity and 24/43(55.8%) excessive use of medication. A reduction of 5 DCM at 3 months, 7 DCM at 6 months and 6 DCM at 12 months (p<0.05), and 11 DMM at 3 months, 13 DMM at 6 months and 11 DMM at 3 months was observed. 12 months (p<0.05); we found that 11/43 (25.6%) of the patients had some adverse effect.

**Conclusions:** According to our series, the use of antiCGRP drugs seems effective and safe in patients older than 65 years. Future studies with a larger number of patients and a longer follow-up period are necessary to corroborate these findings.

## P262 Is it worth switching monoclonal antibodies?

### E. Parreira

#### Hospital Professor Dr Fernando Fonseca, Neurology, Amadora, Portugal

**Question:** Is it worth switching monoclonal antibodies for migraine prevention when there are side effects or efficacy failure?

**Methods:** Prospective study of patients that changed monoclonal antibody for migraine prevention due to lack of efficacy or side effects, in a one-year period, from a single headache centre. Data regarding demographic and clinical features, comorbid diseases, and monoclonal therapy (first and second monoclonal used, length of therapy, reason for change, profile of side effects and clinical response) were prospectively collected. Response to therapy was evaluated through headache diaries, patient interview and clinical validated scales.

**Results:** From a total of 82 patients treated at our centre, 16 changed monoclonal therapy (15 women, one male, median age of 45 years, median duration of migraine 23.3 years), 12 of them due to ineffectiveness (initial or latter loss of efficacy after initial response) in 4 of these also because of adverse events) and in 4 exclusively to adverse events. All except two patients changed from erenumab to anti-ligand antibodies (fremanezumab or galcanezumab). First monoclonal therapy lasted from 4 to 24 months (median 12 months) and the second one 1 to 8 months (median 4,6). All the patients that suffered adverse events (high blood pressure, constipation, and local reactions) with the first treatment didn´t experience them with the second one. In the case of lack of efficiency of the first monoclonal 66% responded to the second one; the remaining 33% were mostly cases of chronic migraine with analgesic overuse.

**Conclusions:** In the case of adverse events switching monoclonal seems to be worthwhile as happened in our patients (no longer having unwanted secondary effects), pointing to a real different meaningful effect in the individual patient. On what concerns efficacy it may be advantageous as 66% improved but the short time of follow-up doesn´t allow to exclude latter loss of effect in the long run.

## P263 Study on the presence of comorbidities in a series of 200 patients with migraine and their influence on the effectiveness of fremanezumab

### N. Morollón Sánchez-Mateo^1^, M. P. Navarro^2^, S. Santos^2^, C. Nieves Castellanos^3^, S. Díaz Insa^3^, R. Belvís Nieto^1^

#### ^1^Hospital de la Santa Creu i Sant Pau, Neurology, Barcelona, Spain; ^2^Hospital Lozano Blesa, Neurology, Zaragoza, Spain; ^3^Hospital la Fe de Valencia, Neurology, Valencia, Spain

##### **Correspondence:** N. Morollón Sánchez-Mateo

Introduction: The results of clinical trials may differ from daily clinical practice because they include patients without comorbidities.

Methods: Prospective observational cohort study to analyze whether the presence of comorbidities influences the response to preventive treatment with fremane-zumab.

Results: We included 200 patients on treatment with fremanezumab for at least 3 months, 165 with chronic migraine (CM), 35 with high frequency episodic migraine (HFEM). Seventy-three point three percent of patients with CM and 45.7% with HFM had comorbidities, the most frequent being depression (21.2%) and insomnia (11.6%). Response rate at 3 months (reduction >50% DMM): MEAF 65.7%, MC 62.8%, and at 6 months: MEAF 64%, MC 73.8%, with no statistically significant differences between the two groups. Not having comorbidity is a protective factor for being a responder patient (p = 0.003), highlighting generalized anxiety (67.2% vs 36.0%, p = 0.003) and fibromyalgia (65.7% vs 42.9%, p = 0.044) as risk factors.

Conclusion:

The presence of comorbidities, especially anxiety and fibromyalgia, may predict lack of response to fremanezumab in patients with high-frequency episodic migraine and chronic migraine.

## P264 Predictors of galcanezumab response in Korean patients with migraine

### S. A. Kim^1^, H. Jang^2^, M. Lee^1^

#### ^1^Seoul National University Hospital, Neurology, Seoul, South Korea; ^2^Samsung Medical Center, Department of Neurology, Seoul, South Korea

##### **Correspondence:** M. Lee

Question: The objective of this study was to assess predictors of galcanezumab response in Korean patients with migraine.

Methods: We prospectively recruited and followed up patients from June 2020 to October 2021 who received monthly galcanezumab treatment in Samsung Medical Center. We defined the treatment response with ≥ 50% reduction in monthly migraine days. Demographics, migraine characteristics, comorbid medication overuse, disease duration, triptan response, previous response to botulinum toxin treatment, monthly headache days, headache impact, depression (Patient Health Questionnaire-9 score ≥ 8), anxiety (Generalized Anxiety Disorder-7 score ≥ 5), number of previously failed preventive medication classes, and the presence of pain-free day were tested by using the univariable logistic regression analysis. Variables with univariable p <0.2 were include in the multivariable analysis.

Results: Among 104 patients (81.7% female; mean age 42.0 ± 13.02; 76.9% chronic migraine; and 45.5% medication overuse headache) recruited, 58 (55.7%) were responders. From the univariable logistic regression analysis, chronic migraine, medication overuse headache, nausea or vomiting, triptan response, monthly headache days, depression, the number of previously failed preventive medication classes, and the presence of pain-free day were included in the multivariable logistic regression analysis. The multivariable analysis showed chronic migraine (OR 0.05 [95% CI 0.00–0.82], p=0.036) and the number of previously failed preventive medication classes (OR 0.55 [95% CI 0.33–0.92], p=0.024] were independently associated with treatment response.

Conclusion: Chronic migraine and multiple failures from preventive medication are associated with poor galcanezumab response. Further studies are needed to investigate if earlier treatment before disease chronification may lead to a greater therapeutic gain from anti-CGRP(-receptor) monoclonal antibody treatments.

## P265 Can We Descriminate Migraine Susceptibile to CGRP Antagonism?

### M. Zaletel^1,2^, G. Požlep^2^, B. Žvan^1^

#### ^1^University Clinical Centre of Ljubljana, Vascular Neurology, Grosuplje, Slovenia; ^2^University Clinical Centre of Ljubljana, Pain clinic, Grosuplje, Slovenia

##### **Correspondence:** M. Zaletel

*Background:* Migraine is recognized as a disorder of calcitonin gene-related peptide (CGRP) pathway. CGRP test can discriminate migraine from non-migraine using CGRP-induced headache (CGRP-IH) and cerebral hemodynamic changes. We test hypothesis that hemodynamic changes related to cerebral vascular responses could discriminate between migraineurs prone to CGRP effects.

*Methods:* We included two groups of participants. Group A consisted of twenty healthy participants (nine females aged 37.0 ± 2.8 years, 11 males aged 41.8 ± 7.6 years). Group B consisted of twenty participants with migraine (15 females aged 41.9 ± 9.9 years, 5 males aged 38.2 ± 9.2 years). Responses in middle cerebral artery (R MCA) and posterior cerebral artery (R PCA) were determined by measuring mean arterial velocity (vm) and calculating difference between vm during CGRP stimulation and basal vm. The Et-CO2 was measured by an infrared capnograph. CGRP-IH, MO and MA were detected according to the International Classification of Headache Disorders third edition. We calculated the responses (R) of vm MCA, vm PCA, Et-CO2, HR, and MAP as differences between measuring points.

*Results:* We found significant differences in the frequency of CGRP-IH between migraineurs and non-migraineurs (p=0.001). We found linear positive relationship between responses R2 MCA and R2 Et-CO2 (r=0.477, p=0.002) and significant difference between product of R2 MCA Et-CO2 of migraineurs (30.3±8.7 cms-1 mmHg) and controls (4.9±2.8 cms-1 mmHg) (p=0.008). The AUC for product R2 MCA Et-CO2 is 0.750 (95% CI 0.598-0.902 p=0.007).

*Conclusions:* CGRP- test might discriminate between migraine susceptible to CGRP from non-susceptible CGRP migraineurs and non-migraineurs. It could be used to predict response to anti-CGRP monoclonal antibodies.

## P266 Interim Analysis on the Effectiveness of Fremanezumab for the Preventive Treatment of Migraine: The Observational PEARL Study

### M. Ashina^1^, D. Mitsikostas^2^, F. M. Amin^1,3^, P. Kokturk^4^, G. Sahin^5^, C. Schankin^6^, P. Dorman^7^, P. Pozo-Rosich^8^, L. Lyras^4^, C. Myers^9^, A. Ahn^9^, C. Tassorelli^10,11^

#### ^1^University of Copenhagen, Rigshospitalet, Danish Headache Center, Copenhagen, Denmark; ^2^Aeginition Hospital, National and Kapodistrian University of Athens, Athens, Greece; ^3^University of Copenhagen, Rigshospitalet, Copenhagen, Denmark; ^4^Teva Pharmaceutical B.V., Amsterdam, Netherlands; ^5^Lund University, Department of Clinical Sciences, Lund, Sweden; ^6^Inselspital, University Hospital Bern, University of Bern, Bern, Switzerland; ^7^The Newcastle upon Tyne Hospitals NHS Foundation Trust, Newcastle upon Tyne, United Kingdom; ^8^Vall d’Hebron University Hospital and Autonomous University of Barcelona, Barcelona, Spain; ^9^Teva Branded Pharmaceutical Products R&D, Inc., West Chester, PA, United States; ^10^IRCCS Mondino Foundation, Pavia, Italy; ^11^University of Pavia, Pavia, Italy

##### **Correspondence:** M. Ashina

**Objective:** Fremanezumab, a humanised monoclonal antibody, that selectively targets calcitonin gene-related peptide (CGRP), is approved in Europe for migraine prevention in adults with ≥4 monthly migraine days (MMD). PEARL study aims to provide real-world data on effectiveness, acute medication use, and disability in patients (pts) initiating fremanezumab treatment. Here we present the second interim analysis.

**Methods:** PEARL is a 24-month, pan-European, prospective, observational study. Eligible pts are adults (≥18 years) diagnosed with episodic or chronic migraine (EM, CM) and starting fremanezumab treatment. The primary endpoint is the proportion of pts with ≥50% reduction from baseline (BL) in average MMD (≥50% response) during the 6 months post fremanezumab initiation. Secondary endpoints were assessed at multiple timepoints from Months 1 to 24 and include mean change from BL in: MMD; average monthly days of acute migraine medication use; disability scores (Migraine Disability Assessment [MIDAS] and 6-item Headache Impact Test [HIT-6]).

**Results:** 574 pts (EM, 26%; CM, 74%) were evaluated in this interim analysis; 65% had used prior anticonvulsants, 61% beta-blockers, and 51% tricyclic antidepressants for preventive treatment. For patients with data for the primary endpoint (n = 313), 56% (EM, 69%; CM, 52%) achieved ≥50% MMD response during the 6 months after fremanezumab initiation. For secondary endpoints, the mean±SD change from BL at Month 6 was –8.0±7.1 for MMD, –6.7±6.2 for average monthly days of acute migraine medication use, –52.7±58.5 for MIDAS score, –9.5±8.8 for HIT-6 score. One pt experienced a drug-related serious adverse event of dysphonia.

**Conclusion:** The real-world effectiveness of fremanezumab is supported by this interim analysis, with more than half of pts reporting a ≥50% reduction in MMD during the 6 months after fremanezumab initiation, improvement in other secondary outcome measures, and favorable safety profile.

## P267 Response patterns in migraine patients in prophylactic treatment with different anti-CGRP pharmacological blockade systems

### F. Schiano di Cola^1,2^, M. Bolchini^1,2^, G. Ceccardi^1,2^, S. Caratozzolo^1^, P. Liberini^1^, A. Padovani^1,2^, R. Rao^1^

#### ^1^ASST Spedali Civili Brescia, Brescia, Italy; ^2^Università degli Studi di Brescia, Brescia, Italy

##### **Correspondence:** F. Schiano di Cola

*INTRODUCTION:* aim of the present study was to compare anti-CGRP versus anti-CGRP-receptor monoclonal antibodies on migraine prevention in patients with high frequency episodic and chronic migraine.

*MATERIALS AND METHODS:* this observational study was conducted at the Headache Centre – ASST Spedali Civili Brescia. All patients in monthly treatment with an anti-CGRP (either molecule or receptor) monoclonal antibody (mAb) with an available 6 months follow-up were included. Clinical and demographical characteristics were gathered at baseline (T0) for all patients. Data regarding efficacy outcome were collected following one (T1), three (T3) and six (T6) months of treatment.

*RESULTS:* one hundred and thirty-three consecutive patients were enrolled, of whom 49 patients in treatment with an anti-CGRP (galcanezumab 120 mg) and 84 with the anti-CGRP-receptor (erenumab 140 mg). Both treatments showed a significant clinical improvement at T3 and T6. At T3, a significantly higher percentage of super responders (47.6% vs 23.1%) and a significantly lower percentage of non responders (14.3% vs 29.8%) was found in patients in treatment with galcanezumab compared to erenumab (p=0.02). Similarly, at T6, a significantly higher percentage of super responders (44.1% vs 26.6%) and a significantly lower percentage of partial responders (5.9% vs 25.4%) was found in patients on galcanezumab compared to erenumab (p=0.05).

*CONCLUSIONS:* the present study on a real-world sample confirms the beneficial effect of both monoclonal antibodies. The comparison of these treatments displayed no difference in term of adverse events while arguing for a more favorable clinical outcome for anti-CGRP antibodies.

## P268 Effect of Galcanezumab in Migraine and Concomitant Medication Overuse Headache Without Prior Drug Withdrawal

### J. A. Membrilla^1^, M. J. Ruíz Castrillo^1^, L. Sánchez Casado^1^, C. Corral^1^, M. Sastre Real^2^, J. Díaz de Terán^1^

#### ^1^University Hospital La Paz, Neurology, Madrid, Spain; ^2^University Hospital Severo Ochoa, Neurology, Leganés, Spain

##### **Correspondence:** J. A. Membrilla

Introduction- Medication overuse headache (MOH) is a disabling and usual comorbidity in migraine patients. We aim to evaluate the effect of Galcanezumab (anti-CGRP monoclonal antibody) in migraine patients with concomitant MOH without prior drug withdrawal.

Methods- Prospective observational study of migraine and MOH patients treated with Galcanezumab in the Headache Unit of a tertiary hospital. Patients received education about MOH, but no detoxification was performed prior to starting therapy. The evaluated outcomes were: monthly headache days (MHD), reduction of symptomatic medication and disability associated with migraine (HIT6 and MIDAS) after 3, 6 and 12 months.

Results- 74 patients were treated with Galcanezumab for at least 6 months. The drug was discontinued in 5/74 (6.8%) due to lack of effect. 53/74 (71.6%) completed 12 months of treatment. The responder rate was 64.7%. Baseline median (p25-75) MHD was 20.9 (14.0-30.0), reduced to 7.0 (4.0-15.0) at 3 months, 6.0 (3.0-12.5) at 6 months, and 10.0 (3.5-15.0) in 12 months. Monthly NSAID consumption decreased from 15.5 (1.5-30.5) to 4.0 (0.0-10.0) at 3 months, to 3.0 (0.0-7.0) at 6 months, and to 4.0 (0.0-14.0) at 12 months. Monthly triptan use decreased from 14.0 (8.0-20.0) to 4.0 (0.0-8.0) at 3 months, to 4.5 (2.0-9.8) at 6 months, and to 5.0 (1.5-10.0) at 12 months. Likewise, a reduction in MIDAS and HIT6 was recorded. All these differences were statistically significant (p<0.001).

Conclusion- Galcanezumab is a valid therapeutic option in migraine patients with comorbid MOH, being able to decrease headache burden, drug intake and disability without prior drug withdrawal.

## P269 Factors associated with favorable outcome in galcanezumab treatment for chronic migraine: A clinic-based prospective study

### B. K. Kim^1^, H. C. Lee^1^, S. Cho^2^

#### ^1^Nowon Eulji Medical Center, Neurology, Seoul, South Korea; ^2^Uijeongbu Eulji Medical Center, Neurology, Seoul, South Korea

##### **Correspondence:** B. K. Kim

**Objective:** Although migraine-specific monoclonal antibody galcanezumab is a very effective and well tolerated preventive treatment of migraine, there is limited ability to predict a clinical meaningful response to galcanezumab. To identify clinical predictors of good response to galcanezumab in patients with CM. **Methods:** This is a prospective study conducted in patients with CM treated with monthly galcanezumab injections. Treatment response was determined after 12 weeks of follow-up. The variables included were age, sex, duration of CM, characteristics of headache, accompanying symptoms of migraine, monthly headache days (MHD), response to triptans, medication overuse, depression and anxiety. **Results:** In 238 patients with CM, 64.3% showed more than 50% reduction in MHD. The mean age was 43.2 years. 83.2% were female. The MHD was 25 days. Medication overuse was presented in 58.0%. Compared to the non-responder group, the responder group was younger (41.6±13.3 years vs 46.3±13.4 years, *p* = 0.039). In the headache characteristics, responder group had lower frequency of baseline headache days (24.0±6.4 vs 26.0±6.2, *p* = 0.012), more nausea (70.0% vs 53.2%, *p* = 0.019), more vomiting (73% vs 60.0%, *p*= 0.033), and more photophobia (68.3% vs 57.4%, *p* = 0.025) compared to the non-responder group. The responder group also showed better triptan response (73.1% vs 52.1%, *p* = 0.047) and no depression in PHQ-9 (78.3% vs 58.9%, *p* = 0.008) compared to the non-responder group. Multivariable regression analysis revealed that absence of depression (OR = 0.439, 95% CI = 0.216–0.896, *p =* 0.024) and presence of accompanying symptoms (OR =3.0, 95% CI = 1.139–7.899, *p* = 0.026) were significantly associated with better response to galcanezumab treatment. **Conclusion:** Our real-world data shows the efficacy of galcanezumab in patient with CM regardless of medication overuse. Depression and presence of accompanying symptoms of migraine were significant response predictors.

## P270 Past Preventive Migraine Treatment in Patients Initiating Fremanezumab in Clinical Practice: Interim Data from the PEARL Study

### M. Ashina^1^, D. Mitsikostas^2^, F. M. Amin^1,3^, P. Kokturk^4^, A. C. Poole^5^, G. Sahin^6^, C. Schankin^7^, T. Nezadal^8^, I. Pavão Martins^9^, P. Pozo-Rosich^10^, P. Dorman^11^, M. L. Sumelahti^12^, L. Lyras^4^, C. Tassorelli^13,14^

#### ^1^University of Copenhagen, Rigshospitalet, Neurology, Copenhagen, Denmark; ^2^Aeginition Hospital, National and Kapodistrian University of Athens, Neurology, Athens, Greece; ^3^University of Copenhagen, Rigshospitalet, Department of Neurorehabilitation/Traumatic Brain Injury, Copenhagen, Denmark; ^4^Teva Pharmaceutical B.V., Amsterdam, Netherlands; ^5^Oslo Headache Center, Oslo, Norway; ^6^Lund University, Department of Clinical Sciences, Lund, Sweden; ^7^Department of Neurology, Inselspital, University Hospital Bern, University of Bern, Bern, Switzerland; ^8^Charles University, Institute of Neuropsychiatric Care, Prague, Czech Republic; ^9^University of Lisbon and Hospital de Santa Maria, Centro Estudos Egas Moniz, Faculty of Medicine, Lisbon, Portugal; ^10^Vall d’Hebron University Hospital and Autonomous University of Barcelona, Barcelona, Spain; ^11^The Newcastle upon Tyne Hospitals NHS Foundation Trust, Newcastle upon Tyne, United Kingdom; ^12^University of Tampere, Faculty of Medicine and Health, Tampere, Finland; ^13^Headache Science and Neurorehabilitation Center, IRCCS Mondino Foundation, Pavia, Italy; ^14^Department of Brain and Behavioral Sciences, University of Pavia, Pavia, Italy

##### **Correspondence:** M. Ashina

**Objective:** Fremanezumab, a humanised monoclonal antibody selectively targeting calcitonin gene-related peptide (CGRP), is approved for preventive treatment of episodic and chronic migraine (EM, CM) in adults with ≥4 monthly migraine days. This interim analysis of the ongoing PEARL study aims to provide real-world data on preventive migraine medication use in patients initiating fremanezumab treatment.

**Methods:** PEARL is a 24-month, pan-European, prospective, observational study in patients (≥18 years) diagnosed with EM or CM and initiating fremanezumab. This study analysed past preventive treatment classes (PPT) used before fremanezumab. Medical history of patients was documented at baseline, including PPT class, duration of PTT, and reason for discontinuation.

**Results:** 574 pts (EM, 26%; CM, 74%) were included in analysis. The proportion of patients taking each PPT was as follows; anticonvulsants, 64.8% (mean±standard deviation [SD] duration of treatment, 9.7±13.00 months); beta-blockers, 60.8% (8.6±10.88 months); tricyclic antidepressants (TCA), 50.9% (9.4±11.37 months); onabotulinumtoxinA, 38.0% (19.4±17.07 months); calcium channel blockers, 28.2% (7.1±8.75 months); angiotensin II receptor blockers, 23.0% (8.7±12.25 months); erenumab 11.5% (11.0±10.42 months); valproic acid 11.1% (7.1±10.35 months); galcanezumab 0.3% (4.5±2.12 months). PPT discontinuation was most commonly due to lack of efficacy across all classes (42.4%-83.9%), while discontinuation due to lack of tolerability ranged from 0% to 39.2% across all classes.

**Conclusion:** In this interim analysis, most patients had received PPTs before fremanezumab initiation, most commonly anticonvulsants, beta-blockers, or TCA, with treatment durations of approximately 9 to 10 months. PPT discontinuation was generally due to lack of efficacy.

## P271 Fremanezumab in patients with refractory migraine and medication-overuse headache: the experience of a tertiary Portuguese hospital

### R. Costa^1,2^, A. L. Neves^1^, A. Costa^1,2^, M. Pinto^1^

#### ^1^Centro Hospitalar Universitário de São João, Neurology, Porto, Portugal; ^2^University of Porto, Department of Clinical Neurosciences and Mental Health, Porto, Portugal

##### **Correspondence:** R. Costa

Question: The monoclonal antibodies anti-CGRP/CGRPr have a promising role in the treatment of migraine. We aimed to evaluate the efficacy, safety, and side effect profile of fremanezumab in patients with refractory migraine and medication-overuse headache in a Portuguese tertiary hospital.

Methods: A longitudinal observational study of a consecutive sample of patients with refractory migraine and medication-overuse headache treated with fremanezumab.

Results: Fifteen patients were included: fourteen were female, with a mean age of 46 years. All patients had chronic migraine and were previously treated with ≥4 preventive drugs. Nine patients had 3 administrations of fremanezumab, six patients had ≥4 administrations. The analgesic drugs being abused were triptans and NSAIDs in 10 patients, only triptans in 2 patients, only NSAIDs in 1 patient, ergotamine and acetaminophen in 1 patient and opioids in 1 patient. The mean headache frequency at 12 weeks was significantly lower than at the baseline (p=0.005). The Headache Impact Test score at 12 weeks was significantly lower (p=0.019). The reduction in monthly headache frequency was ≥50% in 8 patients, ≥75% in 6 patients and <30% in 4 patients. All the patients with a reduction in monthly headache frequency ≥30% suspended the medication over-use and continued the treatment the fremanezumab for another 12 weeks. Of the patients with a reduction in headache frequency <30%, 2 over-used both triptans and NSAIDs, 1 over-used opioids and 1 over-used ergotamine and acetaminophen compounds: 2 patients switched treatment to erenumab, 1 stoped anti-CGRP/CGRPr drugs and 1 waits the group decision. Reported side effects were mild and didn"t lead to discontinuation of the drug.

Conclusions: In our cohort of patients with refractory migraine and medication over-use headache, the use of fremanezumab lead to a significant decrease in both the frequency and impact of the headache, and suspension of the medication overuse in most patients.

## P272 Descriptive study on the presence, treatment and evolution of comorbidities in a series of 200 migraine patients treated with fremanezumab

### N. Morollón Sánchez-Mateo^1^, M. P. Navarro^2^, S. Santos^2^, C. Nieves Castellanos^3^, S. Díaz Insa^3^, R. Belvís Nieto^1^

#### ^1^Hospital de la Santa Creu i Sant Pau, Neurology, Barcelona, Spain; ^2^Hospital Lozano Blesa, Neurology, Zaragoza, Spain; ^3^Hospital la Fe de Valencia, Neurology, Valencia, Spain

##### **Correspondence:** N. Morollón Sánchez-Mateo

Introduction: Clinical trials do not yield information on whether mo-noclonal antibodies against CGRP (MABS) interact with other drugs or influence the evolution of patients' comorbidities.

Methods: Prospective observational study aimed to describe the relationship of frema-nezumab with comorbidities and their respective treatments.

Results: We included 200 patients on fremanezumab treatment for at least 3 months, 165 with chronic migraine (CM), 35 with high-frequency episodic migraine (HFM). Comorbidities were present in 73.3% of patients with CM and 45.7% with HFM, the most frequent being depression (21.2%), insomnia (11.6%) and anxiety (9.7%). HT was present in 7.3% of the sample. After 6 months of treatment 28.7% improved and 15% worsened anxiety and depression, the rest were stable. Only 1 case reported the appearance of HT as an adverse effect. 16 patients with oral contraceptives: no interaction with fremanezumab; 8 patients with immunosuppressants: 87.5% no influence on evolution of comorbidity (myasthenia gravis, asthma), 12.5% worsens polyarthritis; 2 patients with other antibodies: no influence on evolution of comorbidity (rheumatoid arthritis).

Conclusion:

The use of MABS can help to improve comorbidities such as depression or anxiety in patients with migraine.

In our sample there were no complications derived from the combination of frema-nezumab with other monoclonal antibodies, immunosuppressants or oral contraceptives.

## P273 Response to fremanezumab in migraine patients with and without prior aCGRP mAbs - preliminary data from the FINESSE study

### C. Schankin^1^, G. Broessner^2^, C. Gaul^3^, T. Kraya^4^, X. Hamann^5^, B. Haertel^6^, L. Neeb^7^, A. Straube^8^

#### ^1^Inselspital University Hospital, Neurology, Bern, Switzerland; ^2^Medical University, Neurology, Innsbruck, Austria; ^3^Headache Center, Frankfurt a. M., Germany; ^4^St. Georg Hospital, Leipzig, Germany; ^5^Teva GmbH, Ulm, Germany; ^6^Teva Pharma AG, Basel, Switzerland; ^7^Charité University Hospital Berlin, Berlin, Germany; ^8^Ludwig-Maximilians-University, Munich, Germany

##### **Correspondence:** C. Schankin

**Objective:** According to guidelines for health insurance reimbursement, ineffective use of one aCGRP mAb (monoclonal antibody targeting the CGRP pathway), defined by not reaching a reduction of monthly migraine days (MMD) by 50% compared to baseline after 6 months of treatment, excludes reimbursement of another aCGRP mAb. The aim of this preliminary analysis is to quantify the effectiveness of switching to another aCGRP mAb.

**Methods:** FINESSE is a multicenter, two-country (Germany, Austria) prospective, non-interventional study in which effectiveness and tolerability of fremanezumab in adults with episodic (EM) or chronic migraine (CM) are evaluated in clinical practice. Medical history is documented at baseline, including past preventive treatment (PPT) with another aCGRP mAb. A subgroup analysis (14.05.21) focused on PPT with other aCGRP mAbs prior to initiation of fremanezumab. Distribution of patients with and without ineffective PPT with another aCGRP mAb was analysed for the primary endpoint of FINESSE, the proportion of patients reaching ≥50% reduction in monthly migraine days (MMD) that was evaluated during the 6-month period after the first dose of fremanezumab.

**Results:** 308 patients had completed the 6-month visit, 241 without and 67 with prior exposure to another aCGRP mAb. The main reason for discontinuation of prior aCGRP mAb therapy was low efficacy (LoE) in 57 patients (85.1%). In total 150 of 308 patients (48.7%) achieved the primary endpoint of ≥50% reduction in MMD. Eighteen of 57 (31.6%) patients with LoE of prior aCGRP mAbs reached the primary endpoint compared to 129 of 241 (53.5%) patients without prior aCGRP mAb treatment.

**Conclusion:** This first data provides prospective real-world evidence that treatment with fremanezumab is effective in about 30% of patients with prior LoE under aCGRP mAb treatment. For these patients, a switch should not be withheld.

## P274 Could Symptom Severity Predict the Response to Anti-Cgrp Monoclonal Antibodies in Migraine?

### J. A. Membrilla^1^, M. J. Ruíz Castrillo^1^, M. Sastre Real^2^, L. Sánchez Casado^1^, E. Mariño Trillo^1^, C. Corral^1^, C. Estebas^1^, R. González Sarmiento^1^, M. Lorenzo^1^, J. Díaz de Terán^1^

#### ^1^University Hospital La Paz, Neurology, Madrid, Spain; ^2^University Hospital Severo Ochoa, Neurology, Leganés, Spain

##### **Correspondence:** J. A. Membrilla

Introduction- Little is known about predictors of response to monoclonal antibodies against CGRP and its receptor (anti-CGRP/r) in migraine patients. This study aims to find a clinical responsiveness predictor.

Methods- Prospective cohort study of migraine patients treated with anti-CGRP galcanezumab or anti-CGRPr erenumab with 6 months of follow-up at a Headache Clinic of a third level hospital. Symptoms severity was assessed using the Migraine Severity Symptom Score (MSSS). MIDAS and HIT-6 were used for disability assessment. The primary endpoint was the responder rate (RR, defined as ≥50% monthly headache days decrease after 6 months). Exploratory stepwise multiple logistic regression analysis was used for independent predictors of response identification.

Results- 126 patients were recruited. Diagnosis was chronic migraine in 75.4% (95/126) and high frequency episodic migraine in 24.6% (31/126). Baseline monthly headache days, MIDAS and HIT-6 had a mean (SD) of 20.2 (7.2), 75.0 (63.0) and 65.8 (8.5) respectively. After 6 months, the RR was 61.1% (77/126) and monthly headache days, MIDAS and HIT-6 showed a decrease of 9.4 (10.2), 40.1 (61.3) and 9.1 (13.4) respectively. Responder status was associated with MSSS total score (OR 0.87, standard error 0.06, B= -0.613, p=0.015) and severity of worsening pain intensity with usual activities (OR 0.54, standard error 0.24, B= -0.140, p=0.012).

Conclusion- Among the clinical features, worsening with activities severity could be a predictor of response to anti-CGRP/r monoclonal antibodies. These therapies are effective showing frequent but mild AE in real clinical practice.

## P275 Influence of sex hormones on Calcitonin Gene-Related Peptide (CGRP) in tear fluid and plasma across different female hormonal states

### B. Raffaelli, E. Storch, L. H. Overeem, M. Terhart, M. Fitzek, K. S. Lange, U. Reuter

#### Charité - Universitätsmedizin Berlin

##### **Correspondence:** B. Raffaelli

Objective: Sex hormones may have an influence on CGRP release in the trigeminovascular system. We aimed to assess CGRP concentrations in tear fluid and plasma in three groups of women with episodic migraine: A. With a regular menstrual cycle, B. Under combined oral contraception (COC), C. In the postmenopause. For control, we studied three respective groups of age-matched healthy women.

Methods: The study protocol for women with a regular menstrual cycle consisted of two visits on menstrual cycle day 2 ± 2 (during menstruation) and 13 ± 2 (periovulatory period). Participants with COC were examined at day 4 ± 2 of the hormone-free interval (HFI) and between days 7-14 of hormone intake (HI). Postmenopausal women were assessed once at a variable time point. All women were migraine-free and free of pain medication for more than 12 hours. We collected tear fluid and plasma samples at each visit and measured CGRP levels with an enzyme-linked immunosorbent assay (ELISA, Cusabio Biotech, Wuhan). Data within and among groups were compared using nonparametric procedures.

Results: A total of 180 women (n=30 per group) completed study protocol. In general, CGRP concentrations in tear fluid were 80.5x higher than in plasma (IQR 27.8 – 260.7). During menstruation, women with migraine showed significantly higher CGRP concentrations in both tear fluid and plasma compared to healthy controls [tear fluid: 1.2 ng/ml (IQR 0.4 – 2.5) vs. 0.2 ng/ml (IQR 0.1 – 1.2), p=0.005; plasma: 5.9 pg/ml (IQR 4.4 – 10.8) vs. 4.6 pg/ml (IQR 2.8 – 6.9), p=0.020]. In migraine patients without hormonal contraception, tear fluid CGRP concentrations during menstruation were significantly higher compared to patients under COC (p=0.015 vs. HFI and p=0.029 vs. HI). CGRP levels did not differ between migraine and control groups under COC and during postmenopause.

Conclusion: Sex hormone fluctuations across the female lifespan have an influence on CGRP concentrations in women with migraine.

## P276 Prevalence of migraine according to Migraine Screening-Questionnaire (MS-Q) and headache characteristics in patients with inflammatory bowel disease (IBD)

### A. Gonzalez-Martinez, I. Muro García, S. Quintas, M. Chaparro, J. P. Gisbert, A. Sanz, M. J. Casanova, C. Rubín de Célix, J. Vivancos, A. B. Gago-Veiga

#### Hospital Universitario de La Princesa & Instituto de Investigación Sanitaria Princesa (IIS-Princesa)

##### **Correspondence:** A. Gonzalez-Martinez

Objective: The gut-brain axis describes a complex bidirectional association between neurological and gastrointestinal (GI) disorders. In patients with migraine, GI comorbidities are common. We aimed to evaluate the prevalence of migraine according to Migraine Screening Questionnaire (MS-Q) among patients with inflammatory bowel disease (IBD) and describe the headache characteristics compared to a control group. Additionally, we explored the relationship between migraine and IBD activity.

Methods: We performed a cross-sectional study through an online survey including patients with IBD from the IBD Unit at our tertiary hospital. Clinical and demographic variables were collected. MS-Q was used for migraine prevalence evaluation. Scale scores from HIT-6, HADS, ISI, Harvey-Bradshaw and Mayo were also included.

Results: We evaluated 66 patients with IBD and 47 controls. Among patients with IBD, 28/66 (42%) were women, mean age 42 years and 23/66 (64%) had ulcerative colitis and 43/66 (65.15%) Crohn"s disease. MS-Q was positive in 13/66 (23%) of IBD patients and 4 (13%) controls. Among patients with IBD, headache was unilateral in 5/13 (38%) and throbbing in 10/13 (77%). We found that a female sex (p= 0.006), weight (p=0.002), height (p=0.003) and anti-TNF use (0.0035) were associated with MS-Q positive diagnosis. We did not find any association between HIT-6 and IBD activity scales scores.

Conclusions: Migraine prevalence according to MS-Q is higher in patients with IBD than in the control group, which might be explained by the relationship between these entities through the gut-brain axis. Some sociodemographic characteristics of patients with IBD could predict the presence of migraine. These results highlight the importance of migraine detection among patients with IBD which could improve management of this condition and the quality of life of these patients.

## P277 Influence of migraine and migraine aura on the prescription of hormonal contraception in German outpatient gynecological clinics

### M. Fitzek*,* P. Kull, K. S. Lange, L. H. Overeem, E. Storch, M. Terhart, U. Reuter, B. Raffaelli

#### Charité - Universitätsmedizin Berlin

##### **Correspondence:** M. Fitzek

Objective: Hormonal fluctuations during the menstrual cycle affect migraine frequency, duration and intensity. Use of combined oral contraception (COC) containing new generation of estrogen, COC with extended cycle as well as gestagen monotherapy (GM) can reduce the burden of disease. Access to hormonal contraception (HC) to women with migraine especially with aura (MA), has often been denied due to an increased risk of ischemic stroke. However, the absolute risk of ischemic stroke with the use of HC in migrane is minor and the prescription after informed consent is possible. We assessed the influence of migraine without aura (MO) and MA on the prescribing behavior of HC among German gynecologists and investigated potential factors influencing gynecologist"s decision-making process.

Method: In this descriptive observational study of practicing German gynecologists, prescription of HC in migraine was investigated using a self-administered online-based survey with up to 29 items from October 2021 to March 2022. The survey was distributed via mail and e-mail.

Results: A total of n=851 gynecologists responded to the questionnaire. Gynecologists regularly ask for MO (94.5%) and MA (91.8%) prior to prescribing HC. In the presence of MO, 75% of participants reported to prescribe COC only under certain conditions, with cardiovascular risk factors and other comorbidities particularly influencing the decision. In contrast, GM is prescribed in MO by 82% of participating gynecologists without restrictions. In MA, 90% of gynecologists do not prescribe COC at all and 47% of gynecologists stated to not prescribe GM or do so only under certain conditions. Almost all gynecologist reported to have initiated (80%), discontinued (95.8%) or changed (99.2%) a HC due to migraine.

Conclusion: Although HC is not generally contraindicated in migraine patients, presence of MA but also MO hinder German gynecologists to prescribe HC.

## P278 Sex differences in migraine attack characteristics: a longitudinal E-diary study

### I. Verhagen, B. van der Arend, D. van Casteren, S. Le Cessie, A. Maassen van den Brink, G. Terwindt

#### Leiden University Medical Center

##### **Correspondence:** I. Verhagen

Background Women retrospectively report longer migraine attacks and more accompanying symptoms than men, but this has not been confirmed in longitudinal studies (1). Supposed differences could result from more refractory perimenstrual migraine attacks, or migraine attacks in women in general could be associated with different characteristics.

Methods We assessed differences in migraine attack characteristics between men and women, who were prospectively followed with previously validated E-diaries at the Leiden Headache Center. The primary outcome was attack duration. Secondary outcomes comprised recurrence risk and prevalence of accompanying symptoms. Differences between men and women were assessed with linear and logistic models using GEE, corrected for chronic migraine, medication-overuse headache and age dichotomized at 50 as a proxy for menopause.

Results A total of 1,347 women and 284 men were included. Both perimenstrual (62% (47-79%), p<0.001) and non-perimenstrual (15% (5-25%), p=0.03) migraine attacks had longer duration than migraine attacks in men. Recurrence risk was greater for perimenstrual (OR (95%CI): 2.39 (1.93-2.95), p<0.001), but not for non-perimenstrual (1.18 (0.97-1.45), p=0.06) attacks. Migraine attacks in women in general were more often accompanied by photophobia, phonophobia and nausea, but less often by aura symptoms.

Conclusion Not only perimenstrual, but also non-perimenstrual migraine attacks have longer duration than migraine attacks in men, although to a lesser extent. In general, women more often experience accompanying symptoms than men. These results highlight the need for sex-specific approaches in migraine treatment and research.

1. Buse DC, Loder EW, Gorman JA, et al. Sex differences in the prevalence, symptoms, and associated features of migraine, probable migraine and other severe headache: results of the American Migraine Prevalence and Prevention (AMPP) Study. Headache 2013;53:1278-1299.

## P279 Ketogenic diet for migraine prevention: an effective option beyond weight loss

### M. Valente^1^, R. Garbo^1^, F. Filippi^1^, A. Antonutti^1^, V. Ceccarini^1^, Y. Tereshko^1^, C. Di Lorenzo^2^, G. L. Gigli^1^

#### ^1^Clinical Neurology Unit of Udine; ^2^Sapienza University of Rome

##### **Correspondence:** M. Valente

Question: ketogenic diet (KD) is gaining attention as a possible non pharmacological approach for migraine prevention, supported by many pre-clinical and clinical observations. KD is also used for weight loss purposes, and there is a well defined relationship between migraine and weight excess. However, it is not known if the effect of KD on migraine is only due to weight reduction or if it depends on mechanisms which are specific for the ketogenic nature of the intervention.

Methods: we conducted a retrospective observational study on patients suffering from migraine who received a KD as a preventive treatment. All the patients were evaluated both from a neurological and a nutritional point of view, including bioimpedance analysis.

Results: 23 migraine patients were considered in the analysis, 10 (43.5%) were affected by chronic migraine and 6 (26.1%) were suffering from medication overuse headache. The number of previously failed preventive treatments was 1.78±2.21. After KD, we observed a reduction in monthly headache days (12.5±0.5 v.s. 6.7±8.6; p<0.001) and in days of acute medication intake (11.06±9.37 v.s. 4.93±7.99; p=0.008). We also observed a reduction of patients" weight (73.8±15.2 v.s. 68.4±14.6; p<0.001) and BMI (26.9±6.2 v.s. 23.7±8.1; p<0.001), with decrement of the fat mass (28.6±12.5 v.s. 20.6±9.8; p<0.001). Responders and non-respondersto KD did not differ for weight loss (5.6±2.7 v.s. 6.2±5.1; p=0.299) or fat mass loss (6.1±2.1 v.s. 5.0±4.1; p=0.120). In addition, we observed no significant difference in the reduction of headache days between patients who had normal BMI or who were overweight or obese at baseline (9.2±11.5 v.s. 3.7±3.2; p=0.545).

Conclusions: these data corroborate the use of KD as a preventive treatment for migraine. Moreover, since KD improved migraine independently from weight or fat mass loss, its action is probably mediated by mechanisms specific for this kind of nutritional intervention.

## P280 The association between Alternative Healthy Eating Index and odds of migraine headaches: a case-control study

### D. Fotros^1^*,* M. Noormohammadi^2^, S. Razeghi Jahromi^1^, M. Togha^2^

#### ^1^Shahid Beheshti University of Medical Sciences; ^2^Tehran University of Medical Sciences

##### **Correspondence:** D. Fotros

Background: Migraine is the most common disabling primary headache globally. Although the pathogenesis of migraine headache is not fully understood, the possible role of inflammation and disturbed immune system has been proposed; thus, adherence to a dietary pattern that follows the Alternative Healthy Eating Index (AHEI) might reduce the risk of migraine. The current study aimed to investigate the association between the AHEI and odds of migraine headaches.

Methods: This case-control research was conducted on a total of 501 patients with migraine headaches (94.2% were women) and 576 sex-matched healthy controls (94.4% were women). A valid and reliable semi-quantitative food frequency questionnaire was used to record participants' dietary intakes. AHEI-2010 score was measured based on the dietary records, and regression models were used to determine the association between AHEI and migraine headaches odds.

Results: In the multivariable-adjusted model, the odds of migraine headaches was 76% lower for the patients in the last tertile of the AHEI score (aOR: 0.24, 95%CI: 0.16, 0.35, P for trend < 0.001). In the both base and adjusted models, odds of migraine headaches was significantly lower in patients in the last tertile of Whole grains (aOR: 0.60, 95%CI: 0.43, 0.82, P for trend: 0.001), Legumes (aOR: 0.30, 95%CI: 0.25, 0.48, P for trend < 0.001) and PUFA (aOR: 0.12, 95%CI: 0.08, 0.18, P for trend < 0.001). Patients in the last tertile of Fruits (aOR: 1.77, 95%CI: 1.27, 2.45, P for trend < 0.001), and sodium (aOR: 2.57, 95%CI: 1.82, 3.64, P for trend < 0.001), had a higher odds of migraine headaches in the both base and adjusted models

Conclusion: Following a dietary pattern, which is in adherence to the healthy eating index, may be protective against migraine headaches.

## P281 The association between Mediterranean diet adherence and odds of migraine headaches: a case-control study

### D. Fotros^1^*,* M. Noormohammadi^2^, S. Razeghi Jahromi^1^, M. Togha^2^

#### ^1^Shahid Beheshti University of Medical Sciences; ^2^Tehran University of Medical Sciences

##### **Correspondence:** D. Fotros and M. Togha

Background: Migraine is a chronic neurological disorder characterized by attacks of moderate or severe headache and reversible neurological and systemic symptoms. Individuals with migraines may benefit from a nutritional approach incorporating a Mediterranean pattern. The current study aimed to investigate the association between the Mediterranean diet and odds of migraine headaches.

Methods: This case-control research was conducted on a total of 501 patients with migraine headaches (94.2% were women) and 576 sex-matched healthy controls (94.4% were women). A valid and reliable semi-quantitative food frequency questionnaire was used to record participants' dietary intakes. Adherence to the Mediterranean diet was assessed using MEDI-LITE (literature-derived Mediterranean diet) score to determine the association between the Mediterranean diet and migraine headaches odds.

Results: In the multivariable-adjusted model, the odds of migraine headaches was 38% lower for the patients in the last tertile of the MEDI_LITE score (aOR: 0.62, 95%CI: 0.45, 0.86, P for trend: 0.003). In both base and adjusted models, odds of migraine headaches were significantly lower in patients in the last tertile of Whole grains (aOR: 0.60, 95%CI: 0.43, 0.82, P for trend: 0.001), and Legumes (aOR: 0.30, 95%CI: 0.22, 0.42, P for trend < 0.001). Patients in the last tertile of Fruits and nuts (aOR: 1.84, 95%CI: 1.32, 2.56, P for trend < 0.001), Mono-unsaturated/saturated fatty acids ratio (aOR: 1.54, 95%CI: 1.13, 2.11, P for trend: 0.006), and dairy (aOR: 2.06, 95%CI: 1.45, 2.91, P for trend < 0.001), had a higher odds of migraine headaches in the both base and adjusted models.

Conclusions: A higher MEDI_LITE score, indicating greater adherence to the Mediterranean diet, is associated with a lower odds of migraine headaches. Therefore, adherence to the Mediterranean diet pattern is associated with reducing the odds of migraine headaches.

## P282 Evaluation of Serum Leptin Level in a Sample of Egyptian Patients with Migraine

### W. Osman Amer, M. Haddad Hemida, I. El Metwaly Ibrahim

#### Al-Azhar University

##### **Correspondence:** W. Osman Amer

Background: Obesity is a risk factor for multiple neurological disorders including stroke, dementia and migraine. In addition, several cytokines and adipocytokines associated with migraine are modulated by body mass, which also act in the neurogenic inflammation in migraine. The aim of the study: was to throw light and asses leptin levels, one of the adipcytokines, in headache – free period of migraine patients and investigate its relation to vascular risk factors. Material and methods: Sixty – three patients with episodic migraine and 33 control subjects were enrolled in the study. All participants were subjected to full history taking and clinical examination, anthropometric measurements, Body mass index and fat mass values were calculated,fasting blood glucose , lipid profiles and serum leptin assay by ELISA technique ,CT brain to exclude any brain lesions. Results: Leptin levels were found significantly lower in migraineurs than controls (21.16 ± 2.4 ng/ml, 35.48 ± 8.1 ng/ml ; p < 0.001 ) . Body mass index and fat mass were not differ between 2 groups. Conclusion: Migraine patients have lower leptin levels, which may be related to the pathogenesis of migraine. The importance of these findings on the prevalence, pathogenesis and treatment of migraine needs to be investigated in further detailed studies.

## P283 Onabotulinum toxin A block of the sphenopalatine ganglion in patients with persistent idiopathic facial pain: a randomized, triple-blind, placebo-controlled cross–over study.

### K. A. Jamtøy^1^, W. M. Thorstensen^1^, L. J. Stovner^1^, A. Rosén^2^, S. Maarbjerg^3^, D. Dodick^4^, M. R. Simpson^1^, E. Tronvik^1^

#### ^1^Norwegian University of Science and Technology (NTNU); ^2^Haukelands University Hospital; ^3^Danish Headache Center; ^4^Mayo Clinic

##### **Correspondence:** K. A. Jamtøy

**Introduction:** Patients with Persistent idiopathic facial pain (PIFP) experiences a high degree of suffering with scarcity of therapeutic options. Many patients experience diagnostic delay, misdiagnoses and treated by several specialties without success or with inflicted harm. It has been suggested that the sphenopalatine ganglion (SPG) play a role in modulating the pain in PIFP. Parasympathetic nerves can be blocked by onabotulinum toxin A (BTA). Our group has developed an instrument (MultiGuide) to deliver medicines with high precision to the SPG with CT- guided navigation. **Objectives:** The aim was to investigate the efficacy and safety of injecting BTA towards the SPG in patients with PIFP. **Methods:** This cross-over study included a four-week baseline period, followed by an injection of 25 units BTA/placebo and a 12-week diary registration. The primary endpoint was the change from baseline to weeks 5-8 in diary average pain. **Results:** For the primary endpoint there was no statistically significant difference between BTA vs placebo (-0.00; 95 % CI: -0.57 to 0.57) (*P=* 0.996). Exploring a potential carry-over effect, we analysed separately the sequence where placebo came before BTA. With this, there was a significant difference between BTA and placebo in weeks 1-4, weeks 13-16 and weeks 17-20. When analysing the average daily pain, it was lower during the 20 weeks following the first injection among participants who had received BTA compared to those who had received placebo, which was a 15% reduction from baseline. **Conclusion:** This trial did not meet the primary efficacy endpoint, possibly because of a carry-over effect. Injection of BTA toward the SPG using the MultiGuide appears to be safe and well-tolerated in patients with PIFP. The post hoc analyses, considering the study as a parallel group study after the first injection to avoid the potential carry over effect, gave evidence of an effect that started within the first 4 weeks and lasted for at least 20 weeks.

**Fig. 1 (abstract P283).**
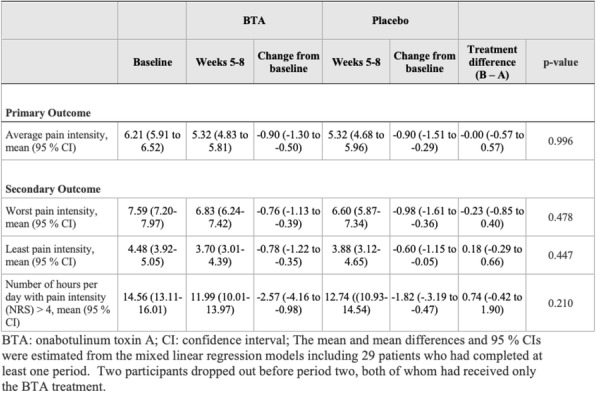
Pain severity outcomes and number of hours in BTA versus placebo group at baseline and at weeks 5-8

**Fig. 2 (abstract P283).**
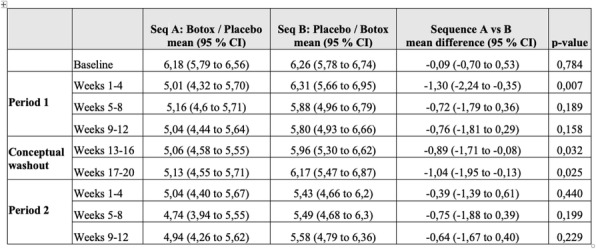
Primary outcome (pain intensity measured on a numerical rating scale) demonstrated by treatment sequence A and B

**Fig. 3 (abstract P283).**
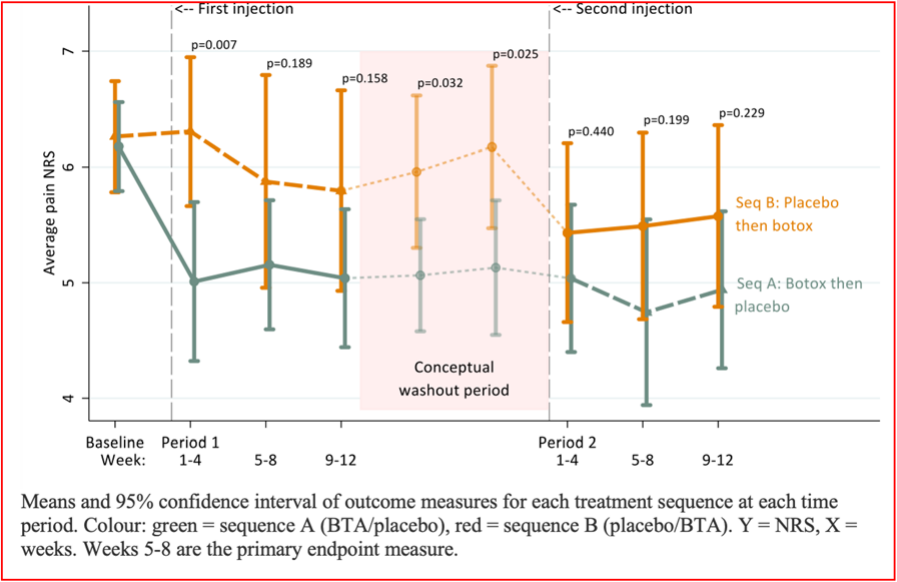
Primary outcome demonstrated by treatment sequence A and B

## P284 nVNS treatment instead of microvascular decompression

### D. Moreno Ajona, M. D. Villar-Martínez, P. J. Goadsby

#### King's College London

##### Correspondence: D. Moreno Ajona

Question: Non-invasive Vagal nerve stimulation (nVNS) with the GammaCore device is a safe and well tolerated treatment. Randomised sham-controlled trials have shown nVNS is efficacious for the treatment of cluster headache. There is also some evidence of the efficacy of nVNS for other trigeminal autonomic cephalalgias, namely hemicrania continua and paroxysmal hemicrania. On the other hand, patients who fail standard treatment for SUNCT/SUNA and are found to have trigeminal neurovascular conflict may be considered for microvascular decompression.

Methods: We report the case of a 58-year old woman who had a history of chronic SUNA. She had initially responded to pregabalin and then added lamotrigine, which was not sufficiently efficacious. She also failed gabapentin. Following funding the patient was started on nVNS. A previous MRI 10 years ago did not include trigeminal views. A recent brain MRI with 3D-CISS sequence showed neurovascular conflict and thinning of the of the trigeminal nerve compared to the contralateral, to the pain, side.

Results: The patient, who had been referred to neurosurgery given the new MRI findings, became attack-free after 2 months on nVNS, which she was taking at 4 treatments TDS. After 1 year of follow-up the benefit persisted and a procedure is no longer being contemplated.

Conclusions: The utility of nVNS as a treatment for refractory SUNCT/ SUNA, even in the presence of neurovascular conflict, could open the doors for its use before considering surgery, given the well-recognised risks of a procedure.

**Fig. 1 (abstract P284).**
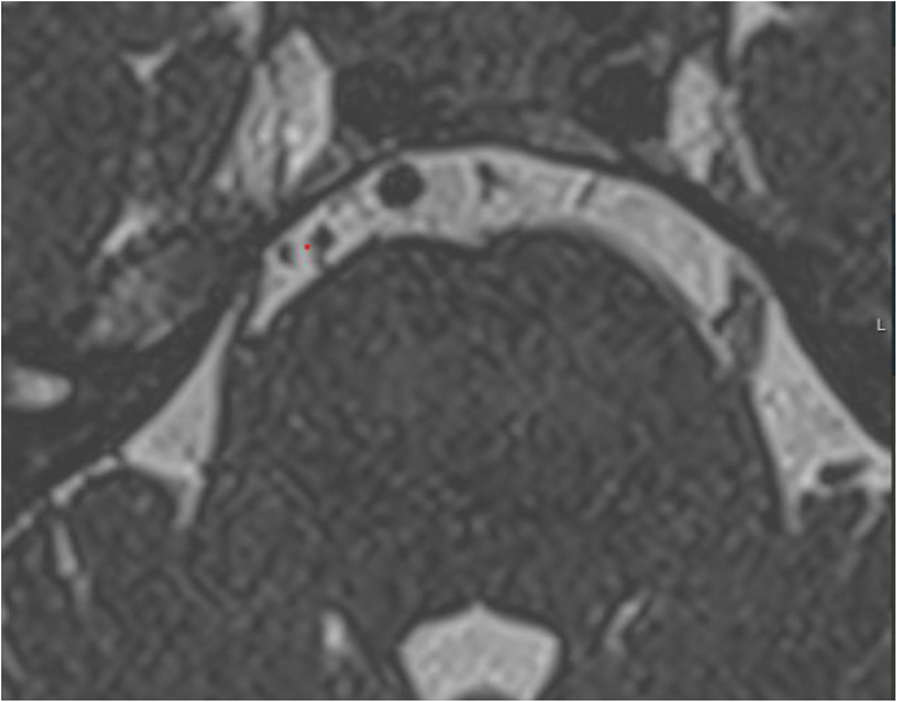
See text for description.

## P285 Effectiveness and safety of pulsed radiofrequency in patients with chronic and refractory trigeminal neuralgia and cluster headache

### A. Gonzalez-Martinez^1^, M. Domínguez Gallego^2^, S. Quinta^2^, C. Montero Grande^3^, E. Rojo^4^, M. Muñoz^4^, D. Ochoa^4^, C. Pérez^2^, J.Vivancos^2^, A. B Gago-Veiga^2^

#### ^1^University Hospital Severo Ochoa; ^2^Hospital Universitario de La Princesa & Instituto de Investigación Sanitaria Princesa (IIS-Princesa); ^3^Universidad Autónoma de Madrid; ^4^Hospital Universitario de La Princesa

##### **Correspondence:** A. Gonzalez-Martinez

Objective: Pulsed radiofrequency (PRF) is a neuromodulation technique used in chronic pain conditions which does not cause irreversible nerve damage. We aimed to evaluate the effectiveness and safety of Gasser and sphenopalatine ganglion PRF in patients with chronic and refractory trigeminal neuralgia (TN) and cluster headache (CH), respectively.

Methods: We performed a retrospective study including patients with TN and CH according to the International Classification of Headache Disorders-ICHD-3, attended at the national reference Pain Unit for refractory pain of our tertiary hospital. We evaluated clinical and demographic variables. The primary endpoint was the reduction in the number of attacks. Secondary objectives were 50% response rate, change in Numerical Rating Scale (NRS), Patient Global Impression (PGI) at 3 months, percentage of recurrence and major adverse events.

Results: Among 293 patients who received radiofrequency in our center, 19 had a headache diagnosis of chronic and refractory TN and 6 CH according to the ICDH-3, therefore a total of 25 patients were analyzed. A reduction in the number of attacks was observed in 14/19(73.68%) of patients with NT and in 5/6(83.3%) of patients with CH. A 50% response rate was observed in 11/19(58%) of patients with TN and there were differences between the NRS at baseline and at 3 months, statistically significant in NT(p<0.05). Among patients with CCH, 4/6 (66.6%) showed a 50% response rate. Pain recurrence occurred in 13/14(92,86%) patients with TN and 5/5(100%) patients with CH, half of them more than one year after RFP.

Conclusions: According to our study, PRF of Gasser and sphenopalatine ganglion is an effective and safe choice for patients with NT and CCH, even considering recurrence, especially in fragile patients not candidates for aggressive procedures.

## P286 Ultrasound Guided Percutaneous Electrical Nerve Stimulation for the Third Occipital Nerve inPatients with Suboccipital Headache Pain. Treatment Proposal

### E. Anarte-Lazo^1^, C. Bernal-Utrera^1^, J. J Gonzalez-Gerez^2^, C. Rodriguez-Blanco^1^

#### ^1^University of Seville; ^2^University of Almeria

##### **Correspondence:** E. Anarte-Lazo

Background

Suboccipital headache is one of the main symptoms in various pathologies such as cervicogenic headache, acute and chronic whiplash. Sensitization of the third occipital nerve has been identified as one of the main causes of pain specific to the suboccipital region. Classically transcutaneous stimulation (TENS) has been used to treat the symptoms, however, the inclusion of ultrasound in the physiotherapeutic field allows a more specific invasive treatment, guaranteeing the safety of the intervention.

Objective

To perform a specific, safe and effective percutaneous approach to the third occipital nerve (TON).

Methods

Treatment Parameters with Asymmetrical Biphasic Current:

15 minutes

Low Frecuencies: 2-4Hz

Pulse Amplitude: 150-300 useg

Materials:

Ultrasound

Disinfection and Asectic Material

Dry Needle (0,30/0,40x0,25)

Electrotherapy equitment with clamp connectors.

Results

Approximately 1-2 mm superficial to the CII-CIII facet joint in longitudinal view with the probe perpendicular to all planes and about 2-3 cm posterior to the mastoid process and 0.5 cm posterior and inferior to the vertebral artery. A linear probe in longitudinal view is used for this purpose. The out-of-plane or in-plane access will depend on the ergonomics offered by the patient. Doppler mode should be used to locate vascular structures such as the inferior branch of the occipital artery. Sonographically, the TON appears hypoechoic with an internal architecture with a hyperechoic outer rim.

Conclusion

Ultrasound-guided intervention allows a safe physiotherapeutic approach. Percutaneous intervention with electrotherapy allows a physiotherapeutic specific approach. The implementation of electrotherapy is a non-pharmacological alternative, less expensive and more accessible.

**Index**

**Table:** 

**A**	
Abed, E.	P65
Abramchuk, O.	P204
Abreu, Â.	A22, P172
Abreu, P.	P87
Acarsoy, C.	P139, P246
Adamczyk, W. M.	P164
Afridi, S.	P50
Agustinho Rodrigues, R.	P22
Ahmed, F.	A42
Ahmed, Z.	P59
Ahn, A.	P266
Aibar Duran, J. A.	A25, A27
Ailani, J.	P103, P142, P143, P148, P149
Akcicek, H.	P50
Al-Hamaid, F.	P77
Al-Hassany, L.	P139
Al-Karagholi, M. A.-M.	A3, P116, P91
Al-Khazali, H. M.	P242
Al-Mahdi Al-Karagholi, M.	A37
Al-Sayegh, Z.	P242
Alba-Delgado, C.	A29
Albertí Vall, B.	A25, A26, A27
Ali, D.	P98
Alimajstorovic, Z.	A17, P210, P215
Allena, M.	A6, P113, P121, P193, P60, P94
Alonso Pérez, J. L.	P46
Alonso Redondo, R.	P86
Alpuente, A.	A24, P131, P133, P145, P168, P73
Altamura, C.	A42
Aluoja, A.	P196, P39
Alvarez, F.	P185
Alvarez Escudero, R.	P56
Álvarez Fernández, L.	P86
Alves da Silva, V.	P22
Amand-Bourdon, C.	P192
Ambrosini, A.	A39
Amin, F. M.	P20, P244, P266, P270, P53
Amirguliyev, S.	A3
Anarte-Lazo, E.	P100, P102, P286
Andersen, A. S. S.	P82
Andreou, A. P.	A42, P257
Antoli Martinez, H.	P55
Antonutti, A.	P279
Apulente, A.	A42
Aranceta, S.	P56
Arkuszewski, M.	P156
Armando, M.	P239
Armesto Rivas, J.	P86
Arruda, R.	P257
Aschauer, K.	P165
Ashina, H.	P20
Ashina, M.	A13, A3, A37, P103, P111, P112
	P116, P117, P129, P137a, P141, P20, P242
	P250, P266, P270, P38, P40, P91
Ashina, S.	P242
Asskour, L.	A24
Audacio Ramos Fernandez, I.	P22
Audouze, K.	P1
Ayala, J. L.	P180, P190
Ayer, D.	P137a
Azam, A.	P225
Azimova, J.	P152, P248, P258
Azzahra Elbahi, F.	A37, P116
**B**	
Babaei, M.	P101
Babiano-Nodal, M.	P72
Bagdy, G.	A19
Bainomugisa, C.	A21
Bakhtadze, S.	P241
Baksa, D.	P62
Balestri, M.	P233
Bandeira Rocha, L.	P22
Baptista, M. V.	P179, P26
Baraldi, C.	A42
Barbanti, P.	P109, P119
Barbosa Mokdeci Surerus, R.	P22
Barloese, M.	A23
Batista Aveaneda, M. P.	P22
Baviskar, R.	P162
Bayar, D.	A41
Baykan, B.	P256
Bazo Diniz, C. E.	P22
Bazo Diniz, S.	P22
Becker, W.	P120a
Belascoaín, R.	P191
Belin, A.	A5
Belvís, R.	P83, P88, P99
Belvís Nieto, R.	A25, A26, A27, P263, P272
Bendtsen, L.	P82
Bernal-Utrera, C.	P100, P102, P286
Bernardes da Silva Neves, L.	P22
Best, P.	P106
Bevilaqua Grossi, D.	P107, P147, P164
	P182, P236, P240
Bhandari, G.	P169

**Index**

**Table:** 

Biaduzzini, F.	P260
Bigal, L.	P147
Bigal, M.	P147
Bighiani, F.	A6, P60, P94
Biglia Diniz, I.	P22
Bilchik, T.	P105, P110
Bivol, S.	P96
Blumenfeld, A.	P106, P114, P115
Bobi, J.	A33
Boinpally, R.	P103
Boix Moreno, A.	P200
Bolay, H.	P256
Bolchini, M.	P267
Bömers, J.	P126
Boon, E.	P154
Borges Nager, G.	P151
Borrell Pichot, M.	A25, A26, A27, P83, P88, P99
Bos, D.	P139, P246
Boskovic, Z.	P24
Bottiroli, S.	P113, P121
Boucherie, D.	A33, P10
Boudreau, G. P.	P150
Bračić, M.	P58
Bradley, C.	P21
Brameli, A.	P163
Brañas Fernández, F.	P86
Brandt, P.	P15
Brandt, R.	P13, P15, P18
Braschinsky, K.	P247
Braschinsky, M.	A41, P196, P247, P39
Brevig, T.	A14
Brimble, M.	A1
Briscoe, J.	P257
Broadhurst, S.	P140
Broessner, G.	P273
Bron, C.	A21
Bronstein, A.	P188
Brown, P.	P98
Brunelli, N.	A42
Bruni, O.	P227
Bsteh, G.	P213, P214, P216, P217, P220
Budrewicz, S.	P153, P177
Burcin, C.	P29
Buse, D. C.	P118, P124, P243
Bussiere, J.	A11
Bustamante Rocha, R.	P151
Butera, C.	A42
**C**	
Cabral, G.	P179, P26
Cabral, J.	P63
Cabrera Fuentes, A.	P46
Cady, R.	A14, P135
Caetano, A.	P179, P26
Caetano, G.	P59a, P63, P67
Calabria Gallego, M. D.	P33, P35
Calle, C.	P191, P261
Callesen, I.	A23
Callø, K.	P1
Camiña Muñiz, J.	P200
Cammarota, F.	A6, P79
Campoy, S.	P84
Candela, N. C.	P16, P51
Cao, Y.	P166, P69
Caponnetto, V.	A41, P175, P257
Carapinha, D.	A22
Caratozzolo, S.	P267
Carbone, J.	P36
Carlos Soares, R.	P22
Carnovali, M.	A41
Caronna, E.	A24, P131, P133, P145, P73
Carvalho, G. F.	P130, P164
Casanova, M. J.	P276
Casas Limón, J.	P191, P261
Castellazzi, G.	P193
Castro Sanchez, M. V.	P55
Cebola, P.	P44
Ceccardi, G.	P267
Ceccarini, V.	P279
Cerboneschi, M.	P89
Cerdá-Company, X.	P131, P133
Çevik, I. Ü.	P256
Cevoli, S.	A42
Chalermpalanupap, T.	P105, P110
Chalmer, M. A.	A23, A5, P189, P37
Chaparro, M.	P276
Chaudhry, B. A.	P20
Checchi Proietti, M.	P233
Chen, J.	A43
Chen, L.	P4
Chen, S.	A43, P166, P69
Chen, X.	P166, P69
Cheng, S.	P173, P52
Chernenko, I.	P31
Chhetri, S.	P252
Chiarugi, A.	A8, P259

**Index**

**Table:** 

Cho, C.	P25
Cho, S.-J.	P137, P57
Cho, S.	P269, P27, P161, P249
Choi, S. Y.	P27
Chowdhury, D.	P162, P183
Chowdhury, S.	P54
Christensen, C. G.	P189
Christensen, H.	P255
Christensen, R.	A37
Christensen, S. L.	P1
Christensen, S. S.	P40
Christiansen, I.	A34
Christie, S.	P142
Christoffersen, C. L.	P109, P111, P112, P119
Chu, M. K.	P137, P161, P249
Cirillo, M.	A20, P157
Clinic, U.	P248
Cloquell, J.	P168
Colby, C.	P192
Collet Vidiella, R.	A25, A26, A27, P83, P88, P99
Colombo, B.	A42
Condés-Lara, M.	P12
Constantin, L.	P192
Conway, C. M.	P122
Cooke, S.	P5
Coric, V.	P104, P115, P122, P123, P136a
	P148, P149
Corrado, M.	A6, P60, P94
Corral, C.	P268, P274
Correia da Cruz, L.	P22
Corti, L.	P29
Corujo Suarez, M. J.	P200
Coskun, H.	A37
Costa, A.	P237, P271, P87, P92
Costa, D.	P78
Costa, J.	A22
Costa, R.	P271
Cowan, R.	A14
Crespo, C.	P144
Crestani, A. S.	P22
Croop, R.	P104, P115, P122, P123, P136a
	P148, P149
Cruz, A.	P185
Cuadrado, E.	P56
Cuéllar-Partida, G.	P96
Cullum, C. K.	P53
Curvelo Bernardes Silva, G.	P22
Cyntia Lima Fonseca Rodrigues, A.	P22
Czapińska-Ciepiela, E. K.	P153, P177
**D**	
D'Acunto, L.	P260
D'Amico, D.	P174
da Silva, B. A.	P22
Da Silva França, C. F.	P86
Dabruzzo, B.	P105, P106, P110, P120, P124, P141
Dach, F.	P107, P182, P236, P240
Dalic, L.	P52
Dallel, R.	A29
Dalmasso, M. C.	A5
Danser, A. H. J.	A11, A2, A33, P10, P2
Datta, D.	P183
Dave, J.	P202
De Abreu Ferreira, R.	P106, P141
de Boer, I.	A21, A36, A38
de Castro Olyntho Júnior, M. A.	P22
De Cesaris, F.	A8, P259
De Icco, R.	A6, P113, P121, P193, P60, P79, P94
de la Torre Suñe, A.	P131, P73
De Santis, N.	A41
de Vries, T.	A11, P10, P2
Debruyne, F.	P154
Delgado, S.	A22
Delgobbo Pereira, M. V.	P22
Deligianni, C.	A3
Dell Agnello, G.	P138, P155
Della Pietra, A.	A35
Delmotte, K.	P154
Demartini, C.	P94
Demont, A.	P224
Dénes, Á.	A19
Deodato, M.	P260
Deorari, V.	P183
Dhiraj, D.	P50
Di Lorenzo, C.	P279
Díaz de Terán, J.	P17, P191, P261, P268, P274
Díaz Insa, S.	A15, P125, P263, P272
Díaz-Torres, S.	P96
Diener, H.-C.	A14
Dionísio, J.	A22
Dissing, K.	P255
Djamandi, P.	P74
Do, T. P.	A13, A3, P20, P244, P250, P53
do Couto Soares Caversan, R.	P22
Dobos, D.	P62

**Index**

**Table:** 

Dodick, D.	P105, P110, P120, P135, P142
	P143, P283
Domi, F.	P251
Domingos, C.	P59a
Domínguez Gallego, M.	A4, P285
Dong, L.	P64
Dong, Z.	A43, P166, P69
Doporto Fernández, A.	P86
Dorman, P.	P266, P270
Driessen, M. T.	P50
Dubey, A.	P223
Dubey, S.	P223
Ducros, A.	P29
Dueland, A. N.	P108
Dufek, M.	P120
Duflos, C.	P29
Duggal, A.	P183
Duraníková, O.	P134
Dømgaard, M.	A13, P250
Echavarría-Íñiguez, A.	P158, P76
Echeverria, A.	P56
Edvinsson, L.	A7, P3, P9
Eftekhari, S.	P218, P222, P6
Eidlitz Markus, T.	P163, P229, P230
El Metwaly Ibrahim, I.	P282
Elaraby, A.	P225
Elosua-Bayes, I.	P73
Erikstrup, C.	P37
Ernstsen, C.	P40
Esposito, F.	A20, P157
Estebas, C.	P274
Esteves, I.	P59a, P63, P67
Ettrup, A.	P109, P111, P112, P119, P135
Evers, S.	P138, P155, P176
**F**	
Fabregat, N.	P56
Fabrich Marín, M. I.	A15, P125
Falla, D.	P100, P102
Fan, X.	P4
Fang, X.	A31, A32
Fanning, K.	P118, P124, P243
Farah Sabe, F.	P22
Farham, F.	P235, P30
Fattori, D.	A8, P259
Fawsitt, C.	P178
Ferilli, M. A. N.	P227, P228, P233, P238, P49
Ferkingstad, E.	A5
Fernandes, A.	P92
Fernandes, C.	P128
Fernández Cabreira, A.	P86
Fernandez Fernandez, S.	P56
Fernández Vidal, J. M.	A25, A26, A27
Fernández-de-las-Peñas, C.	P182
Fernandez-Vidal, J. M.	P83, P88, P99
Ferrari, M.	P13, P15, P18
Ferreira Bomtempo, F.	P151
Figueiredo, P.	P59a, P63, P67
Filatova, E.	A42
Filippi, F.	P279
Filippi, M.	A42
Finkelstein, I.	P120a
Finnegan, M.	P103, P105, P110, P120
Fitzek, M.	P275, P277
Fleischmann, R.	P156
Florencio, L. L.	P107
Fonseca, C.	P63
Forster, O.	P29
Fortes Garib Batista, S.	P22
Fotros, D.	P280, P281
Fouto, A.	P59a, P63, P67
Frattale, I.	A42
Frederiksen, J. L.	P82
Friedman, D. I.	P143
Fronczek, R.	P13, P15, P18
**G**	
Gago-Veiga, A. B.	A4, P180, P190, P191
	P261, P276, P285
Galambos, A.	P62
Gallardo, V. J.	A24, P131, P133, P168
Gálvez-Goicurría, J.	P180, P190
Gamal, M.	P225
Gandhi, P.	P142, P143
Gao, X.	A43
Garascia, G.	P260
Garbo, R.	P279
García Cebrián, M.	A4
García Domínguez, G.	P46
García Iglesias, C.	P76
Garcia Monco, J. C.	P56
García Pazos, O.	P86
Garcia Trujillo, L.	P55
García-Azorín, D.	P158, P167, P191, P261, P76
Garcia-Azorin, D.	P56
García-Ruiz, C.	P76
Garelja, M.	A12
Gaul, C.	P176, P273

**Index**

**Table:** 

Ge, Z.	P166
Gecse, K.	A19, P62
Geladze, N.	P241
Genç, H.	P256
Gendolla, A.	P176
Genizi, J.	P186
Gentile, M.	A41
Geppetti, P.	A8, P259
Gerring, Z. F.	P96
Gervasio, L.	P79
Ghadiri-Sani, M.	P140, P21
Ghanizada, H.	A37
Ghiotto, N.	P113, P121
Gigli, G. L.	P279
Gil-Gouveia, R.	A41, P53, P59a, P63, P67
Gilev, D. V.	A44
Gillard, P.	P114
Giniatullin, R.	A35
Giniatullina, R.	A35
Gisbert, J. P.	P276
Giuliani Schmitt, L.	P22
Goadsby, P. J.	P103, P109, P115, P119, P129
	P188, P192, P226, P284
Gobbi, C.	A39
Gomes, A.	P87
Gomes Lomba, V. E.	P22
Gómez, C.	P167
Gómez García, A.	P191, P261
Gómez-Pilar, J.	P167
Gonçalves, C.	P179
Gonçalves Alencar Rodrigues Furtado Rocha, S.	P22
Gonderten, S.	P137a, P138, P155
Gong, Z.	A43
Gonzalez Gerez, J. J.	P286
González Oria, C.	P14
González Sarmiento, R.	P274
González-Celestino, A.	P76
Gonzalez-Fernandez, L.	P56
González-García, N.	P191, P261
González-Hernández, A.	P12
Gonzalez-Martinez, A.	A4, P180, P190, P191
	P261, P276, P285
González-Osorio, Y.	P158, P76
Gosalia, H.	P226
Graboski, C.	P120a
Grabova, S.	P70
Granato, A.	P260
Grasby, K. L.	P96
Gray, R.	P195
Grazzi, L.	P174
Grech, O.	P210, P215
Greco, R.	P113, P94
Griffiths, L.	A21, A36
Grillo, V.	A6, P60, P94
Grimaldi Capitello, T.	P228, P232
Grozeva, V.	P28
Gryglas-Dworak, A.	A42, P153, P177
Grøntveit Winnberg, I.	A39
Gu, Q.	P166
Guaschino, E.	P113, P121
Guerrero Peral, Á. L.	P158, P167, P191, P261, P76
Guerzoni, S.	A42
Gui, W.	P69
Guidotti, G.	P80
Guijarro del Amo, M.	P86
Guindani, B.	P79
Guisado, D.	P56
Gulišija, J.	P23
Guo, H.	P103, P142
Guo, S.	A3, P40
Gupta, A.	P146, P159, P171
Gupta, S.	P45, P47, P48
Gurary, N. M.	A44
Gutiérrez-de Pablo, V.	P167
**H**	
Ha, H.	P129
Haan, J.	P18
Haanes, K. A.	P1, P126, P3, P9
Haddad Hemida, M.	P282
Haertel, B.	P273
Hakkinen, I.	P202
Hamann, X.	P273
Han, X.	A43, P166, P69
Hanna, M.	P122
Hansen, J. M.	P250
Hansen, M. B.	A23
Hansen, T. F.	A23, A5, P189, P37
Harder, A.	A36, A5, P127
Harizi (Shemsi), E.	P251
Harriott, A.	P106
Harris, P.	A1
Haselgruber, N.	P165
Haupt, L.	A21
Hay, D.	A1, A12, P90
Hay-Schmidt, A.	P40

**Index**

**Table:** 

He, M.	A43, P69
Heinskou, T. B.	P82
Hendrikse, E.	A12
Heo, K.	P137
Heredia, P.	P261
Hill, B.	P257
Hirman, J.	A14, P135
Hirtz, C.	P61
Hoffmann, J.	A10, A30, P41, P43
Holland, P.	A10, A30, P5
Holm, A.	P126
Homer, V.	A16, P208, P209, P211, P212, P219
Hori, S.	A18
Hornero, R.	P167
Horváthová, S.	P134
Hougaard, A.	A39
Hould, J.	P104
Hu, D.	A43, P166, P69
Huerta-Villanueva, M.	P84
Hussain, H.	P173
Hutchinson, S.	P122, P123
Hutton, E.	P173, P52
Huygen, F.	P13, P15
Hwang, H.	P137
Hyder, Y.	P208
**I**	
Iaccarino, G.	A41
Iannone, L. F.	A8, P259
Iba, C.	A18
Ibrahim, O.	A36
Ighavini, O.	P257
Ikram, M. K.	P139, P246
Ikumi, N.	P131, P133
Imai, N.	P66
Irimia, P.	P144
Isadora Cenci, G.	P151
Isaza-Jaramillo, S.	P38
Ishizuchi, K.	A18
Israelsen, I. M. E.	P218, P222, P6
Ivanov, K.	A35
**J**	
Jafari, E.	P132, P234
Jaimes, A.	P191, P261
Jamtøy, K. A.	P283
Jang, H.	P264
Jang, K.	P137
Jens, K.	P156
Jensen, C.	P218
Jensen, C. M.	P115, P122, P123, P148, P149
Jensen, R. H.	A23, P218, P222, P6, P80
Jensen, R.	P255
Jia, Z.	A43, P69
Josiassen, M. K.	P109, P111, P112, P119
Jovanovski, A.	P24
Juhasz, G.	A19, P62
Juranek, B.	P165
**K**	
Kachynska, T.	P204
Kamen, L.	P123, P148, P149
Kamitaki, B.	P194
Kamm, K.	A42
Kamp-Jensen, C.	P218, P222
Kannan, S.	P252
Kapanadze, N.	P241
Karsan, N.	P226
Kassburg Mello, L.	P22
Katsarava, Z.	A41, P118, P243
Kazantzi, S.	P126, P3, P9
Kazemizadeh, H.	P234
Khachapuridze, N.	P241
Khachidze, I.	P204
Kim, B.-K.	P269, P57
Kim, D. W.	P25
Kim, E. H.	P137
Kim, E. Y.	P27
Kim, K. M.	P137
Kim, S. A.	P264
Kim, S. J.	P161, P249
Kingston, W.	P19
Kislyak, N. V.	A44
Kissani, N.	P256
Kitamura, E.	P66
Klein, D.	P176
Klærke, D.	P1
Knievel, K.	P114
Koch, M.	P156
Kocsel, N.	P62
Kogelman, L. J. A.	P189
Kokonyei, G.	P62
Kokoti, L.	P91
Kokturk, P.	P266, P270
Komori, M.	P137a
Koppen, H.	A40
Kornienko, S.	P152, P248, P258
Környei, Z.	A19

**Index**

**Table:** 

Korobkova, D.	P152, P248, P258
Kortazar, I.	P56
Košta, V.	P23
Koul, A.	P183
Krajnc, N.	P213, P214, P216, P217, P220
Krasenbaum, L. J.	P50
Kraya, T.	P273
Krege, J.	P137a
Krishnan, A.	P183
Kristensen, D. M.	P1
Kristensen, M.	P244
Kristoffersen, E. S.	P250
Krivoshein, G.	A35
Kruja, J.	P70, P74, P75
Kudrow, D.	P148, P149
Kuehn, G.	P176
Kukushkin, M.	P248, P258
Kull, P.	P277
Kuqo, A.	P70
Kuznietsov, I.	P204
Kymes, S.	P178
**L**	
Laas, K.	P196, P39
Labastida-Ramirez, A.	A10
Lafrance, S.	P224
Lakhotia, S.	P169
Lamas Pérez, R.	P14
Lambru, G.	A42, P109, P119, P257
Lampl, C.	P165
Lange, K. S.	P275, P277, P29
Lanteri-Minet, M.	P111, P112, P118, P181
Latorre, G.	P191, P261
Latysheva, N.	A42
Lauridsen, H.	P255
Lavery, G.	P210
Le Cessie, S.	P278
Lea, R.	A36
Lebedeva, E. R.	A44
Lee, C. H.	P160
Lee, H. J.	P161, P249
Lee, H. C.	P269
Lee, H.-S.	P34
Lee, J.	P114
Lee, J. H.	P7
Lee, M. J.	P160
Lee, M.	P264, P27, P137
Lee, S. H.	P161, P249
Lee, W.	P137, P161, P249
Leheste, A.-R.	A41
Lehtonen, M.	A35
Leinonen, V.	A35
Lelleck, V.	P176
Lentsch, S. d.	P245
Leone, M.	P80
Leroux, E.	P118
Levinsky, Y.	P163, P229
Li, H.	P64, P69
Li, K.	A43, P166
Li, L.	A43
Li, L. Q.	P137a
Li, Y.	P166
Lian, Y.	P166
Liberini, P.	P267
Lima Florencio, L.	P147, P182
Lin, J.	A31, A32
Lin, X.	A43
Lipton, R. B.	P103, P104, P105, P110, P115
	P117, P118, P120, P122, P123, P124
	P136a, P142, P143, P148, P149
	P153, P177, P243
Liu, H.	A43, P69
Liu, J.	A43, P166, P69
Liu, Y.	A43
Ljubisavljevic, S.	P207
Lopes, J.	P128
Lopes Penido de Mendonça, L.	P22
Lopez, C. L.	A3
Lopez Bravo, A.	P56
López Guerrero, A.	A4
Lopez-Gonzalez, R.	P38
Lorenzo, M.	P274
Lorini Rodrigues, M.	P22
Lovegren, M.	P104, P122, P136a
Lucas, S.	P221
Ludwig, C.	P210
Luedtke, K.	P130, P164
Luminati Picolo de Oliveira, L.	P22
Lund, N.	A23
Luvsannorov, O.	P256
Luzeiro, I.	P128

**Index**

**Table:** 

Lyons, H.	P215, P221
Lyras, L.	P266, P270
L’Italien, G.	P115
**M**	
Ma, D.	P8
Ma, J.	P105, P106, P110, P120, P120a, P141
Maagdenberg, A. M. J. M. v. d.	A35
Maarbjerg, S.	P283, P82
Maassen van den Brink, A.	A11, A2, A33
	P10, P136, P139, P2, P278
MacAulay, N.	P218
Macedo Goedert, G.	P22
Machado, S.	P172
Macher, S.	P213, P214, P216, P217, P220
Maddahi, A.	A7
Madonia, J.	P104, P136a
Madureira, B.	A22
Magna Garib, A.	P22
Maher, M.	P19
Mahović, D.	P58
Maia, M.	P11
Makovac, E.	P41, P43
Maksemous, N.	A36
Maleska, A.	P156
Malgarejo, L.	P73
Malheiro, S.	P78
Mamkhegov, E.	P152, P248, P258
Manack Adams, A.	P118
Manganotti, P.	P260
Manvelyan, H.	P253
Marazzi, M. C.	P80
Marco, T.	P56
Margolis, M. K.	P192
Marhamati, H.	P30
Maribel, F. M.	P16
Marik, W.	P213, P214, P216, P217, P220
Marina Alejandra, O.	P16, P51
Mariño Trillo, E.	P274
Markovic, A.	P24
Marmura, M.	A14, P141
Marone Barros Lopes, V.	P22
Martelletti, P.	A41, A42
Marti-Marca, A.	P131, P133
Martin, I.	P56
Martin, N. G.	P96
Martin, V.	P117
Martin Bujanda, M.	P56
Martín Pérez, I. M.	P46
Martín Pérez, S.	P46
Martín Ramos, C.	A4
Martín Rivero, A.	P46
Martinelli, D.	P108, P193, P60, P80
Martínez, M.	P144
Martínez Rodríguez, B.	P158
Martinez Viguera, A.	A25, A26, A27
Martínez-Lorenzana, G.	P12
Martinez-Viguera, A.	P83, P88, P99
Martinez-Yelamos, S.	P84
Martins, B.	P237, P92
Martins, J.	P107, P182
Mata, I.	P59
Matamala-Gomez, M.	P121
Mateos Salas, T.	P200
Matharu, M.	A5, P118, P124, P173, P195
	P243, P80
Matthew, H.	P41, P43
Mavlanov, M.	P170, P184
Mawet, J.	P224, P29
May, A.	P164
Mazetto Rocha, N.	P22
Mazzanti, C.	A41
Mazzone, L.	P227
McAllister, P.	P105, P110, P120
McCusker, E.	P103
McVige, J.	P114, P141
Mechtler, L.	P120
Mederer, T.	P83, P88
Mederer Fernandez, T. I.	A25, A26, A27
Mederer-Fernandez, T.	P99
Medland, S. E.	P96
Mehta, D.	A21
Meira, B.	P26
Meise, R.	P130
Membrilla, J. A.	P17, P268, P274
Mendes Bragatto Scornavacca, M.	P147, P182
Mengi, A.	P85
Meshref, M.	P77
Mikkelsen, S.	P37
Millán Vázquez, M.	P14
Minguez, A.	P56
Miscio, A.	A42
Mitchell, B. L.	P96
Mitchell, J.	P210, P215, P221
Mitsch, C.	P213, P214, P216, P217, P220
Mitsikostas, D.	P266, P270
Miyazaki, N.	A18

**Index**

**Table:** 

Mo, H.	P57
Moavero, R.	P227, P228, P232, P233, P238, P49
Moisset, X.	P129
Moleirinho-Alves, P.	P44
Molina Martinez, F. J.	P200
Mollan, S.	A16, P208, P209, P210, P211
	P212, P215, P219, P221
Moniz Dionísio, J.	P172
Monte, G.	P228, P232, P233, P238, P239, P49
Montero Grande, C.	P285
Montheith, T.	P106
Montisano, D. A.	P174
Moon, H.-S.	P57
Moots, R.	P140
Morais Pereira de Medeiros, F.	P22
Morales Caballero, Á.	A4
Moreira, A.	P56
Moreira Craveiro, J. F.	P11
Moreno Ajona, D.	P284
Moreno Rojas, A. J.	P200
Moreno-Ajona, D.	P188
Moriya, A.	P66
Morollón, N.	P83, P88, P99
Morollón Sánchez-Mateo, N.	A25, A26, A27
	P263, P272
Morris, B.	P115
Mosher, L.	P104, P136a
Mota Telles, J. P.	P151
Mrad, Y.	A29
Muharremi, E.	P74
Mulleners, W.	P13, P15
Mullin, K.	P123, P136a
Mundra, A.	P183
Muñoz, M.	P285
Muñoz-Vendrell, A.	A41, P84
Muro García, I.	P276
Murphy, M.	P257
Musialowicz, B.	P194
Mwinyi, J.	P127
Myers, C.	P266
**N**	
Naber, W.	P18
Nachev, P.	P195
Nadiradze, T.	P241
Nagy, K.	P117, P129, P142
Nagy, T.	A19
Nahas, S.	P105, P106, P110, P120
Nair, J.	P140
Nakahara, J.	A18
Narasimhan, R. L.	P146, P159, P171
Naser Moghadasi, A.	P30
Navarro, M. P.	P263, P272
Navarro Muñoz, M. J.	P16, P51
Neeb, L.	P273
Negro, A.	A42
Nematgorgani, S.	P132
Neves, A. L.	P271, P87
Neves, P.	A22
Nezadal, T.	P270
Nicolodi, M.	P89, P95
Nie, L.	P8
Nielsen, K.	P37
Nieves Castellanos, C.	A15, P125, P263, P272
Niiberg-Pikksööt, T.	P196, P39
Nissen, J.	P189
Nizoli de Campos, T.	P22
Noormohammadi, M.	P280, P281
Noory, N.	P82
Norato, A. C. C.	P107
Nordahl, K. M. L.	P9
Novak, K.	P213, P214, P216, P217, P220
Novick, D.	P138, P155
Nowaczewska, M.	P153, P177
Nunes, R.	P59a, P63, P67
Nyholt, D.	A21, P96
**O**	
O'Daly, O.	P43
Obach, V.	P56
Ochoa, D.	P285
of the CCG, o. b.	A5
Ohtani, S.	A18
Okhovat, A. A.	P235
Olesen, A.	P37
Olesen, J.	A44, P1, P189, P37, P40
Olet, S.	P98
Oliveira, R.	A41, P44
Olivier, M.	A15, P125
Olmedo Saura, G.	A25, A26, A27, P83, P88, P99
Ong-Lam, M.	P120
Onofri, A.	P175
Ornello, R.	A41, A42, P108, P175
Osipova, V.	P248
Osman Amer, W.	P282
Ostrowski, S. R.	A23, P37

**Index**

**Table:** 

Ouwehand, R.-L.	P18
Overeem, L. H.	P108, P156, P275, P277
Özdemir, A. A.	P256
Ozge, A.	A41
Özge, A.	P256
**P**	
Padovani, A.	P267
Pagán, J.	P180, P190, P191, P261
Pan, Q.	P4
Panni, T.	P138, P155
Paños Basterra, P.	A4
Pantovic, A.	P24
Papetti, L.	P227, P228, P232, P233, P238, P239, P49
Park, B.-y.	P160
Parreira, E.	A22, P172, P262
Pascual, J.	P138, P155
Pavão Martins, I.	A41, P270, P59a, P63
Pavlovic, J.	P106, P123, P136a
Pedersen, A. S.	A23
Pedersen, O. B.	A23, P37
Pegahi, R.	P181
Pego Reigosa, R.	P86
Peixoto Leal, T.	P59
Pelejão, R.	P179
Pelzer, N.	A36
Pemp, B.	P213, P214, P216, P217, P220
Pérez, C.	P285
Perez Garcia, J.	P16, P51
Perrotta, A.	A39
Petersen, A. S.	A23
Pezarat-Correia, P.	P44
Pho Do, T.	A39
Phul, R.	P109, P111, P112, P119
Piccus, R.	P208
Pijpers, J.	A21
Pinheiro, A. R.	A22, P172
Pinheiro-Araujo, C. F.	P107
Pinnaro, M. S.	P89, P95
Pinto, M.	P271, P92
Pinto, M. J.	P87
Planchuelo-Gómez, Á.	P76
Plaut, S.	P71
Pocora, M. M.	P108, P193
Polivka, V.	P192
Poole, A. C.	P270
Porta-Etessam, J.	P191, P261
Posas III, J.	P98
Poza, J.	P167
Požlep, G.	P265
Pozo-Rosich, P.	A24, A41, A42, P103, P105, P110, P111, P112, P120, P129, P131, P133, P135, P142, P143, P144, P145, P168, P244, P266, P270, P73
Prabhakar, P.	P226
Pradela, J.	P236, P240
Prat, J.	P84
Prewett, M.	P21
Proietti Checchi, M.	P228, P232, P238
Pruckner, P.	P213, P214, P216, P217, P220
Prudenzano, M. P.	A41
Putortì, A.	A6, P60
**Q**	
Qin, G.	P4
Quigley, S.	P81
Quintas, S.	P180, P190, P191, P261, P276, P285
Quka, A.	P70
**R**	
Racioppi, G.	P239
Raffaelli, B.	P108, P156, P275, P277
Raggi, A.	P174
Raibin, K.	P230
Raidvee, A.	P247
Rajpal, N.	P54
Ramirez, A.	A5
Ramiro, C. A.	P190
Ramos Rúa, L.	P86
Ran, C.	A5
Ran, Y.	P166, P69
Ranchero, B.	P68
Ranchon-Cole, I.	A29
Rao, R.	P267
Raoelina, A.	A29
Rask-Andersen, M.	P127
Rasmussen, R. H.	P1
Ray, J.	P173, P52
Razeghi Jahromi, S.	P132, P280, P281
Recio García, A.	P158, P76
Reducha, P.	P126
Reed, M.	P118, P124, P243
Rees, T.	A12, P90
Regnier, S.	A9, P178
Rego, A.	A22
Rehfeld, A.	P1
Rekeda, L.	P141
Renteria, M. E.	P96

**Index**

**Table:** 

Renton, T.	P41, P43
Reuter, U.	P103, P108, P129, P156, P275, P277
Ribeiro Basseto, Y.	P22
Ribeiro Lodo, M. L.	P22
Riesco Perez, N.	P56
Rivera- Mancilla, E.	A2
Rizzoli, P.	P141
Roa, J.	P17
Robblee, J.	P141
Robustelli Della Cuna, F. S.	P79
Rochat, P.	P82
Rodrigues, A.	P147, P182
Rodrigues Simoes, J. A.	P11
Rodríguez Ares, T.	P86
Rodriguez Jimenez, L.	P55
Rodriguez Rodríguez, M.	P86
Rodríguez Rodríguez, R.	A25, A27
Rodriguez-Blanco, C.	P100, P102, P286
Rodríguez-Vico, J.	P191, P261
Rojo, E.	P285
Rojo Prieto, N.	P86
Roncero, N.	P56
Roos, C.	P137a, P29
Rosa, A.	P63
Rosén, A.	P283
Rosignoli, C.	A41, P175
Rroji, A.	P75
Rubín de Célix, C.	P276
Rubio-Beltran, E.	A10
Ruff, D.	P137a
Ruibal, M.	P56
Ruisanchez, A.	P56
Ruíz Castrillo, M. J.	P268, P274
Ruiz-Tagle, A.	P59a, P63, P67
Rukh, G.	P127
Rundblad, L.	P53
Ruprecht, K.	P156
Ruscheweyh, R.	A42
Russo, A.	A20, A42, P157, P175
Russo, M.	A42
Ryan, A. M.	P81
Ryliskiene, K.	A41
**S**	
Sacco, S.	A41, A42, P108, P141, P175, P53
Sahin, G.	P266, P270
Saidvaliyev, F.	P170, P184
Sainz-Torres, R.	A25, A26, A27, P83, P88, P99
Sakai, F.	P118, P124
Salim, A.	P59
Sambini, F. D.	P107
Samir, Y.	P225
Samuel, D. I.	P16, P51
Sances, G.	A6, P113, P121, P174, P193, P60
	P79, P94
Sánchez, S.	P144
Sánchez Casado, L.	P268, P274
Sanchez del Rio, M.	A41
Sanchez Guijo Benavente, A.	P55
Santamaria Montero, P.	P86
Santoro, A.	A42
Santos, D.	P185
Santos, M.	P236, P240
Santos, S.	P263, P272
Sanz, A.	P180, P191, P261, P276
Saraiva, M.	A22
Sastre Real, M.	P191, P261, P268, P274
Sava, S. L.	P154
Savinainen, J.	A35
Schankin, C.	P266, P270, P273
Schiano di Cola, F.	P267
Schim, J.	A14, P122
Schiöth, H.	P127
Schmidbauer, V.	P213
Schoenen, J.	P154
Schroeder, T.	P176
Schulz, F.	P176
Schutter, D.	P2
Schwarz, A.	P164
Schwedt, T.	P114
Schwefel, B.	P103, P117, P129, P143
Schytz, H. W.	P242, P37
Sekiguchi, K.	A18
Sellebjerg, F. T.	A23
Semprini, M.	A6, P60
Seneviratne, S.	P210
Seong, M.	P27
Serôdio, M.	P26
Serra-Mecína, A.	P158
Severt, L.	P120
Sforza, G.	P227, P228, P232, P233, P238
	P239, P49
Shah, D.	P114
Shahien, M.	P225
Sharma, J.	P146, P159, P171
Shemsi, K.	P251
Shen, J.	A31, A32

**Index**

**Table:** 

Shibata, M.	A18
Sicuteri Di Puccio, L.	P89, P95
Sierra-Mencía, Á.	P167, P191, P261, P76
Silva, B.	P128
Silva, N.	P236, P240
Silva, N. A.	P67
Silva, S.	P87
Silvestro, M.	A20, A42, P157, P175
Simpson, M. R.	P283
Sina, C.	P176
Sinclair, A.	A16, P208, P209, P210, P211
	P212, P215, P219, P221
Singh, P.	P169
Singh, S.	P146, P159, P171
Singhvi, S.	P169
Siow, A.	A1
Skorobogatykh, K.	P152, P248, P258
Smilkov, E. A.	P82
Smith, J.	P106, P143
Smith, M.	P68
Smith, T.	P136a, P148, P149, P210
Snellman, J.	A11, A3
Sobrado, M.	P180, P190
Sommer, K.	P118, P120a, P243
Somovilla, A.	A4
Soto-Faraco, S.	P131
Souto Faria Navarro, J.	P22
Spaggiari Marra, J.	P22
Sperling, B.	P109, P111, P112, P119
Springborg, J. B.	P82
Stanyer, E.	A30
Stark, R.	P173
Starling, A.	A14
Stefansen, S.	A13, P250
Steiner, T.	A13
Stewart, A.	P192
Stock, E. G.	P122
Stokes, J.	P142, P143
Storch, E.	P275, P277
Stovner, L. J.	P283
Straburzyński, M.	A42, P153, P177
Straube, A.	P273
Stubberud, A.	P195
Styrishave, B.	P218
Su, W.	P156
Suarez, A.	P56
Sumelahti, M.-L.	P270
Sun, R.	P50
Sun, S.	A43
Sundar, U.	P146, P159, P171
Sutherland, H.	A21, A36
Szabo, E.	P62
Szikszay, T. M.	P164
Søborg, M. L. K.	A23
Søndergaard, H. B.	A23
Sørensen, E.	P189
**T**	
Taboureau, O.	P1
Taha, A.	A33
Takemura, R.	A18
Takizawa, T.	A18
Tan, H. L.	P41, P43
Tana, J.	P70
Tarantino, S.	P227, P228, P232, P233, P238
Tartaglione, L.	P175
Tasma, Z.	A1, A12, P90
Tassorelli, C.	A6, P108, P113, P117, P121
	P174, P193, P266, P270
	P60, P79, P80, P94
Tavy, D.	A40
Taylor-Clark, T. E.	P1
Techlo, T. R.	P189
Tedeschi, G.	A20, P157
Tena-Cucala, R.	P84
Tenenbaum, N.	P156
Terasawa, E.	P50
Tereshko, Y.	P279
Terhart, M.	P275, P277
Terwindt, G.	A21, A36, A38, P136, P245
	P278, P93
Tessitore, A.	A20, P157
Thaci, D.	P176
Thakur, S.	P146, P159, P162, P171
Thaller, M.	A16, P208, P209, P211, P212
	P219, P221
Thiel, C.	P130
Thiry, A. C.	P115, P122, P123, P148, P149
Thole, D.	P80
Thom, H.	P178
Thorat, A.	P146, P159, P162, P171
Thorstensen, W. M.	P283
Thuraiaiyah, J.	P116
Thørner, L. W.	P189
Tian, R.	P4
Toft-Bertelsen, T.	P218

**Index**

**Table:** 

Togha, M.	P101, P132, P187, P234, P256
	P280, P281
Tolentino, G. A.	P107
Tolner, E.	P93
Tolno, V.	P80
Tomar, A.	P183
Topholm, M.	P37
Torelli, P.	A42
Torphy, B.	P68
Torralba, M.	P131
Torre-Suñe, A.	P133, P145, P168
Torres-Ferrús, M.	A24, P131, P145, P73
Toscano Prat, C.	A25, A26, A27
Toscano Prats, C.	P83, P88
Toscano-Prat, C.	P99
Totev, T.	P50
Toyokytty Yoshida, A.	P22
Trevino-Peinado, C.	P191, P261, P72
Tronvik, E.	A39, P195, P283
Troy, E.	P81
Trugman, J.	P103, P117, P120, P129
Tsan, K.	P173
Tsermoulas, G.	P208, P215, P221
Tuloup, G.	P29
Tuñas Gesto, C.	P86
Turk, B. G.	P85
**U**	
Uludüz, D.	P256, P53
Ursitti, F.	P227, P228, P232, P233, P238, P239, P49
Ushenin, A. V.	A44
Uygunoglu, U.	P85
Uzhakhov, A.	P152, P248, P258
**V**	
Vaghi, G.	P174, P60, P79
Vaikjärv, M.	P247
Vainauskienė, J.	A41
Valente, M.	P279
Valeriani, M.	P227, P228, P232, P233, P238
	P239, P49
van Beusekom, H. M. M.	A33
van Casteren, D.	P136, P278
van de Ruit, M.	P93
van den Bogaerdt, A.	A11, A2, P2
van den Hoek, T.	A38, P93
van den Maagdenberg, A.	A21, A36, A5, P127, P93
van der Arend, B.	P136, P278, P93
van der Zwet, R.	A40
van Drie, R.	P10
Van Humbeeck, C.	P154
van Welie, F.	P245
van Welie, R.	P245
van Zwet, E.	P13, P15
Varela, R.	P78
Vase, L.	P178
Vashchenko, N.	P152, P258
Vehagen, I.	P136
Vekilyan, H.	P253
Velasco, F.	P56
Verhagen, I.	A38, P278
Vernieri, F.	A41, A42
Versijpt, J.	P154
Viana, M.	A39
Vicente Peracho, G.	P86
Vieira, A.	P180, P190
Vieira Nascimento, E.	P22
Vieira Nogueira, B.	P22
Vigevano, F.	P227, P228, P232, P238
Viguier, A.	P61
Vijfhuizen, L.	A36
Vilà-Balló, A.	P131, P133
Vilela, P.	P67
Villalón, C. M.	A2
Villar-Martinez, M. D.	P188, P284
Vivancos, J.	A4, P180, P190, P276, P285
Voci, A.	P227
**W**	
Waldorff Nielsen, C. A.	P91
Waliszewska-Prosół, M.	P153, P177
Walker, C.	A1, A12, P90
Walker, J.	P215
Wang, D.	A43
Wang, M.	P8
Wang, R.	P166, P69
Wang, S.	A43
Wang, W.	P114
Wang, X.	P166, P69
Wang, Y.	A43, P4, P166
Wardman, J.	P218
Warfvinge, K.	A7
Watanabe, N.	A18
Weaver, S.	P221
Wei, D.	P69

**Index**

**Table:** 

Welander, N.	P127
Wen, Q.	P4
Westgate, C.	P218
Westgate, C. S. J.	P222, P6
Wilbrink, L.	P13, P15
Winsvold, B. S.	A23, A5
Witt, O.	P176
Wöber, C.	P213, P214, P216, P217, P220
Worm, J.	P82
Wu, Z.	P114
**X**	
Xavier, M.	P63
Xhelili, M.	P75
Xie, W.	P166
Xu, Y.	P166
Xue, Z.	A43
**Y**	
Yakkaphan, P.	P42
Yan, F.	P166, P69
Yang, H. B.	P34
Yaremchuk, I.	P254
Yaremchuk, O.	P254
Yiangou, A.	P210, P215, P221
Yilma, B.	P50
Yin, Z.	P166
Ying Lee, X.	A9, P178
Younis, H.	P77
Younis, S.	P242
Yu, R.	P69
Yu, S.	A43, P166, P69
Yu, S. Y.	P117
Yu, Z.	P166
Yun Yu, S.	P103
**Z**	
Zago Mazzini, M.	P22
Zaletel, M.	P265
Zamanian, S.	P97
Zanaboni, A.	P113, P94
Zecca, C.	A39
Zekja, I.	P75
Zhang, D.	P4
Zhang, M.	A43, P166, P69
Zhang, P.	P194, P198, P199, P201, P202
	P203, P205, P206
Zhang, S.	A43, P69
Zhang, Y.	P4
Zhao, H.	P69
Zhao, W.	A43
Zhou, J.	P4, P64
Zhou Chen, H.	P5
Zhuang, Z.	A. P116
Zhuravlov, O.	P204
Zsombok, T.	P62
Žvan, B.	P265
Zwart, J.-A.	A23, A5

